# Nickel-catalyzed asymmetric hydrogenation of β-acylamino nitroolefins: an efficient approach to chiral amines†
†Electronic supplementary information (ESI) available. See DOI: 10.1039/c7sc02669b



**DOI:** 10.1039/c7sc02669b

**Published:** 2017-07-04

**Authors:** Wenchao Gao, Hui Lv, Tonghuan Zhang, Yuhong Yang, Lung Wa Chung, Yun-Dong Wu, Xumu Zhang

**Affiliations:** a Key Laboratory of Biomedical Polymers of Ministry of Education , College of Chemistry and Molecular Sciences , Wuhan University , Wuhan , 430072 , China . Email: huilv@whu.edu.cn; b Department of Chemistry , South University of Science and Technology of China , Shenzhen , 518055 , China . Email: zhangxm@sustc.edu.cn ; Email: oscarchung@sustc.edu.cn; c Lab of Computational Chemistry and Drug Design , Laboratory of Chemical Genomics , Peking University Shenzhen Graduate School , Shenzhen 518055 , China; d Engineering Research Center of Organosilicon Compounds & Materials , Ministry of Education , Wuhan , 430072 , China

## Abstract

An efficient approach for synthesizing chiral β-amino nitroalkanes has been developed *via* the Ni-catalyzed asymmetric hydrogenation of challenging β-amino nitroolefins under mild conditions, affording the desired products in excellent yields and with high enantioselectivities. This protocol had good compatibility with the wide substrate scope and a range of functional groups. The synthesis of chiral β-amino nitroalkanes on a gram scale has also been achieved. In addition, the reaction mechanism was elucidated using a combined experimental and computational study, and it involved acetate-assisted heterolytic H_2_ cleavage followed by 1,4-hydride addition and protonation to achieve the nitroalkanes.

## Abstract 2

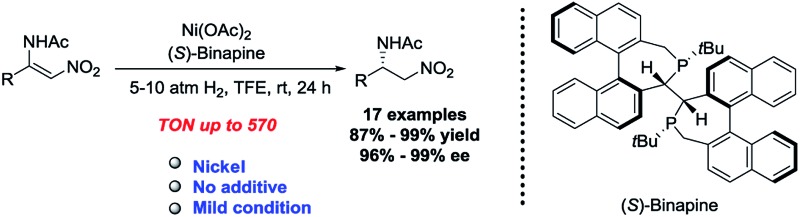
The Ni-catalyzed asymmetric hydrogenation of challenging β-acylamino nitroolefins was achieved under mild conditions, affording β-acylamino nitroalkanes in excellent yields and with high enantioselectivities.

## 

The development of new protocols for synthesizing chiral compounds in an environmentally-friendly and cost-effective manner is an important subject in both academic research and industrial applications.^1^ In this context, asymmetric hydrogenation, one of the most effective approaches for constructing chiral compounds, has been rapidly developed and has achieved remarkable progress. However, almost all of the catalytic systems for asymmetric hydrogenation heavily rely on noble transition metal catalysts based on Ru, Rh, Ir or Pd.^1^ In contrast, catalysts based on the cheap, earth-abundant first-row transition metals have potential advantages in terms of cost and sustainability. Therefore, Fe-, Co- and Ni-catalyzed asymmetric hydrogenation has attracted great attention.^2^

Recently, the Fe-catalyzed asymmetric hydrogenation of ketones and imines and the Co-catalyzed asymmetric hydrogenation of ketones and olefins have been reported.^2–4^ These methods exhibited the great potential of first-row transition metals in asymmetric hydrogenation. However, seminal studies on Ni-catalyzed asymmetric hydrogenation are rare, although heterogeneous nickel catalysts have long played a prominent role in reduction reactions. In 2008, Hamada *et al.* reported the Ni-catalyzed asymmetric hydrogenation of α-amino-β-ketoesters *via* dynamic kinetic resolution.^5^ Subsequently, Zhou and coworkers disclosed a series of studies on the Ni-catalyzed asymmetric transfer hydrogenation of enamides and hydrazones, and the asymmetric reductive amination of ketones (Scheme 1a).^6^ Recently, Chirik and coworkers developed the first example of the asymmetric hydrogenation of α,β-unsaturated esters using Ni catalysts and H_2_ gas (Scheme 1b).^7^ Given that the studies on Ni-catalyzed asymmetric hydrogenation are in their infancy, exploring a wide substrate scope, increasing the enantioselectivities of the products and improving the TON values of the catalysts are highly desirable.

**Scheme 1:**
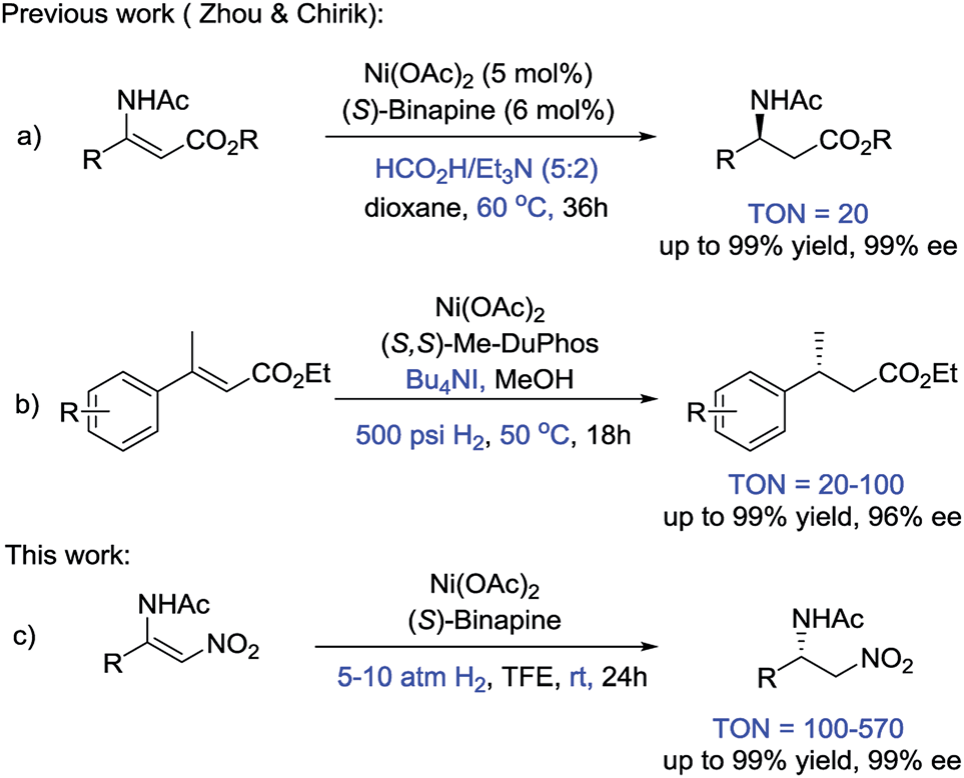
Reports on Ni-catalyzed asymmetric hydrogenation.

Generally, β-acylamino nitroolefins are challenging substrates for asymmetric hydrogenation due to the weak binding affinity of the olefin with an electron-poor nitro group. There are only a few examples on the asymmetric hydrogenation of β-acylamino nitroolefins that employed precious transition metal catalysts.^8^ To the best of our knowledge, cheap transition metals have never been used in the asymmetric hydrogenation of β-acylamino nitroolefins. Herein, we report an efficient access route to chiral β-amino nitroalkanes *via* the Ni-catalyzed asymmetric hydrogenation of β-acylamino nitroolefins under mild conditions (Scheme 1c).

Initially, (*Z*)-*N*-(2-nitro-1-(*p*-tolyl)vinyl)acetamide **1b** was chosen as a model substrate for optimizing the reaction conditions. Some chiral diphosphine ligands were evaluated. When the reaction was carried out in the presence of 5 mol% Ni(OAc)_2_ and 5.6 mol% ligand under 50 atm of H_2_ at 50 °C in MeOH for 24 h, using Bu_4_NI as the additive, all of the *P*-chiral diphosphine ligands could catalyze the reaction with different conversions and enantioselectivities (Fig. 1). (*S*)-Binapine was found to give the best results (>99% conversion, 98% ee). Axial chiral and planar chiral diphosphine ligands had no activity for this reaction. The solvent screening experiments indicated that CF_3_CH_2_OH was the best choice (Table 1, entries 1–6). Further investigation showed that the Bu_4_NI additive had no effect on this reaction (Table 1, entries 7 and 8). When the catalyst loading was reduced from 5 mol% to 1 mol%, the reaction completed smoothly with similar results (Table 1, entry 9). Decreasing the hydrogen pressure to 5 atm did not affect the reaction. The excellent enantioselectivity was maintained when further decreasing the hydrogen pressure to 1 atm, but the yield dramatically decreased (Table 1, entries 10–14).

**Fig. 1:**
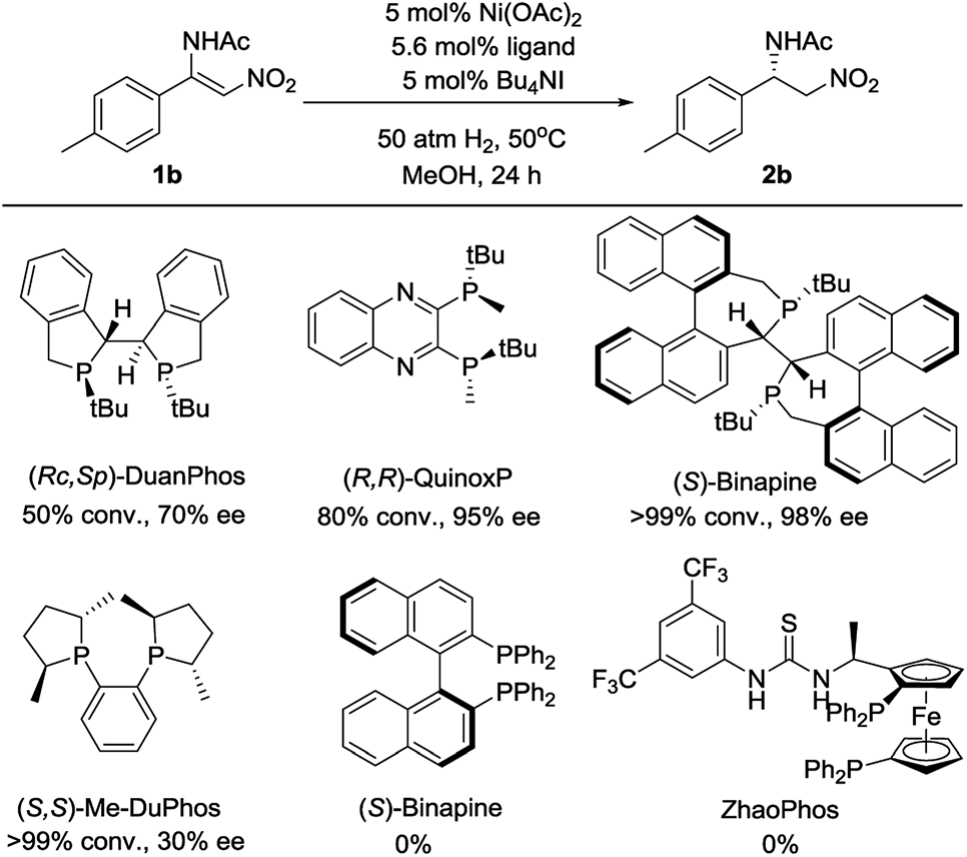
The performance of chiral phosphines in the asymmetric hydrogenation of **1b**.

**Table 1:** Optimization of the reaction conditions^*a*^

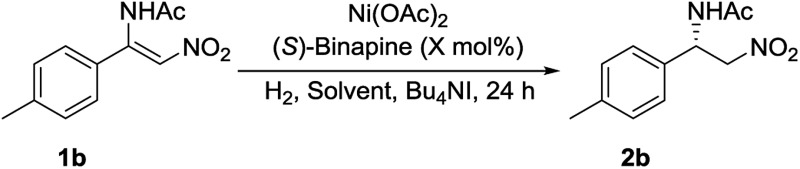						
Entry	*X* (mol%)	Solvent	H_2_ (atm)	Temp. (°C)	Conv.^*b*^ (%)	ee^*c*^ (%)
1	5	MeOH	50	50	>99	98
2	5	EtOH	50	50	38	90
3	5	iPrOH	50	50	40	92
4	5	DCM	50	50	26	89
5	5	THF	50	50	Trace	60
6	5	Toluene	50	50	12	78
7	5	TFE	50	50	>99	>99
8^*d*^	5	TFE	50	50	>99	>99
9^*d*^	1	TFE	50	50	>99	>99
10^*d*^	1	TFE	50	40	>99	>99
11^*d*^	1	TFE	50	rt	>99	>99
12^*d*^	1	TFE	10	rt	>99	>99
13^*d*^	1	TFE	5	rt	>99	>99
14^*d*^	1	TFE	1	rt	72	>99

^*a*^Conditions: Ni(OAc)_2_ : (*S*)-binapine : Bu_4_NI = 1 : 1.1 : 1, and **1b** (0.1 mmol) in 1 ml of solvent.

^*b*^The conversion was determined using ^1^H NMR and HPLC analysis.

^*c*^The ee values were determined using HPLC analysis with a chiral stationary phase.

^*d*^Without using Bu_4_NI as an additive.

Under the optimized reaction conditions, the substrate scope was examined. As shown in Scheme 2, various electron-rich or electron-poor aromatic group substituted β-acylamino nitroolefins could be hydrogenated smoothly to afford the corresponding β-amino nitroalkanes in high yields and with excellent enantioselectivities. The position of the substituents on the benzene ring had no influence on the reactivity and enantioselectivity. In addition, a heteroaromatic group substituted β-acylamino nitroolefin was also well-tolerated, albeit with a slight decrease in the yield and enantioselectivity. It is worth noting that alkyl substituted β-acylamino nitroolefins, which are challenging substrates for Rh or Ir catalysts, could also be hydrogenated in high yields and with excellent enantioselectivities.

**Scheme 2:**
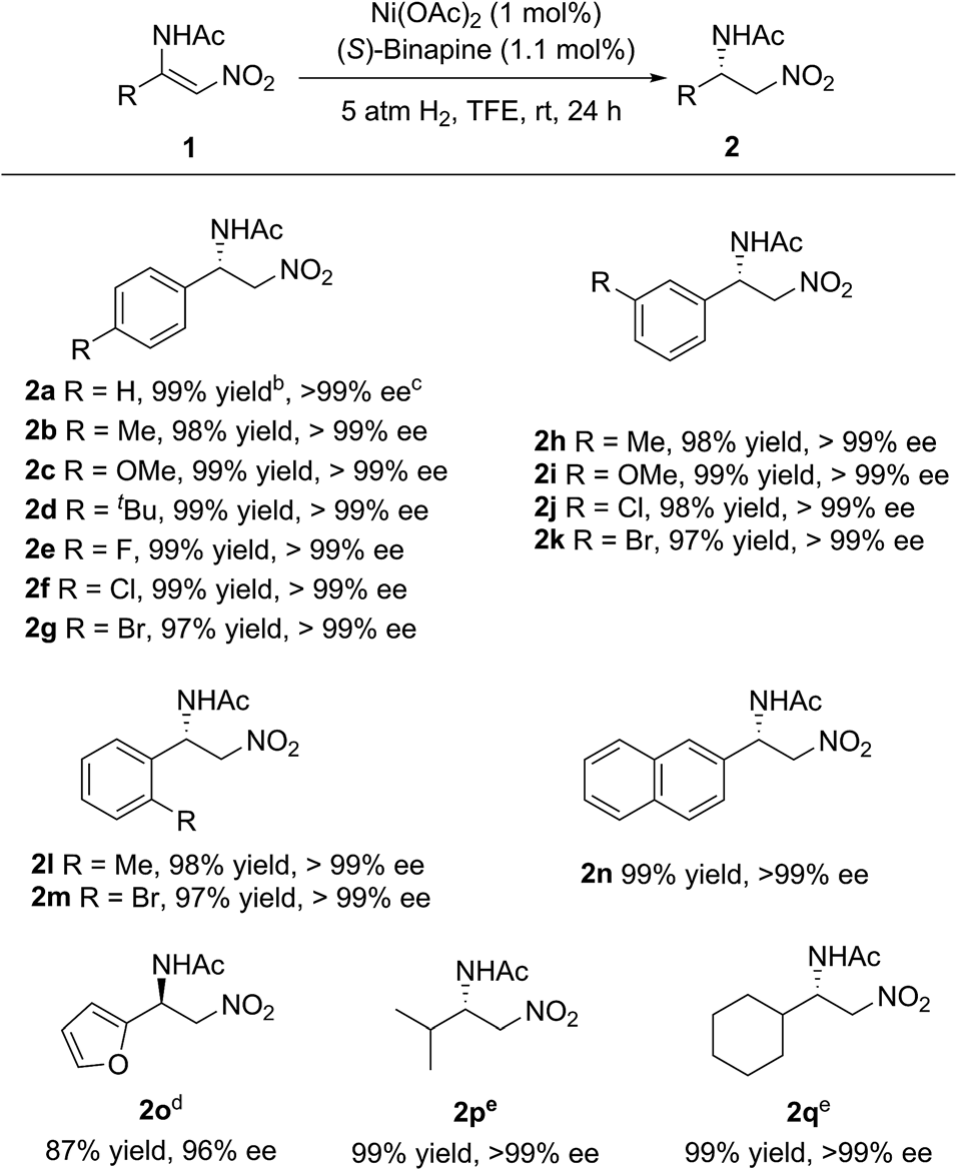
Substrate scope. ^*a*^ The general reaction conditions: the ratio **1** (0.1 mmol) : Ni(OAc)_2_ : (*S*)-binapine = 100 : 1 : 1.1, 1 ml of TFE as the solvent under 5 atm of H_2_ at rt for 24 h. ^*b*^ Isolated yield. ^*c*^ Determined using HPLC analysis with a chiral stationary phase. ^*d*^ 6 mol% catalyst, 50 °C, 50 atm of H_2_. ^*e*^ 10 atm of H_2_.

To explore the potential application of this methodology, the synthesis of the chiral β-acylamino nitroalkane on a gram scale was examined. The reaction was carried out under 5 atm of hydrogen pressure at room temperature in the presence of 1 mol% Ni(OAc)_2_/(*S*)-binapine complex, affording the desired compound **2a** in 99% yield and with 99% ee. When the catalyst loading was decreased to 0.1 mol%, we achieved **2a** in 57% yield and with 99% ee (Scheme 3).

**Scheme 3:**
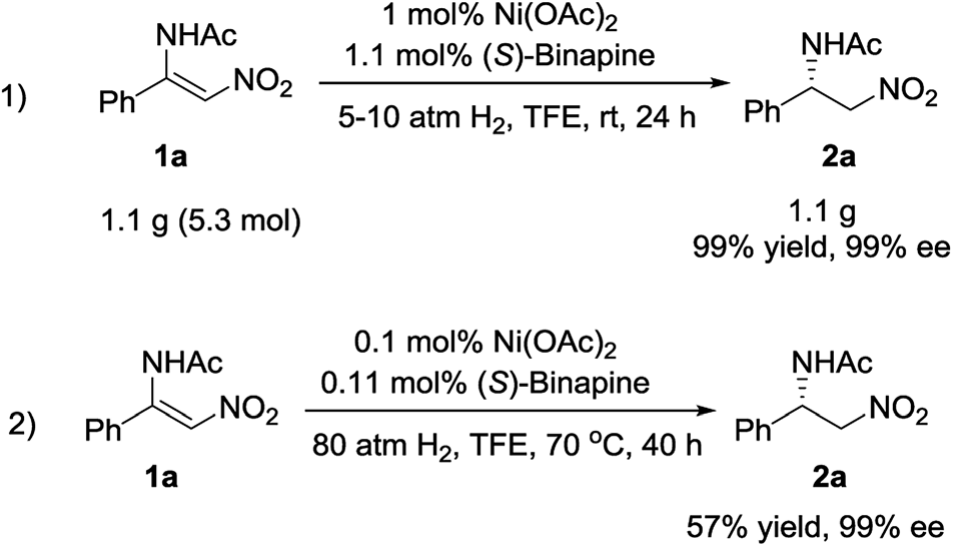
Gram-scale reaction and S/C evaluation.

To decipher the possible reaction mechanism for the Ni-catalyzed asymmetric hydrogenation of β-acylamino nitroolefins, a series of isotopic labeling studies were conducted. Firstly, when **1a** was hydrogenated with 10 atm of D_2_ in TFE solution, the deuterium atom was solely added at the β position (Scheme 4a). When the experiment was repeated with H_2_ and CD_3_OD, the deuterium atoms were incorporated at the α position (Scheme 4b). Performing the hydrogenation reaction under 30 atm of D_2_ in CD_3_OD solution gave the expected compound with the deuterium atoms at both the α and β positions (Scheme 4c). Finally, when **2a** was dissolved in CD_3_OD solution and stirred, the deuterium atoms were found to be incorporated at the α position, showing the H/D scrambling of the product (Scheme 4d).^9^

**Scheme 4:**
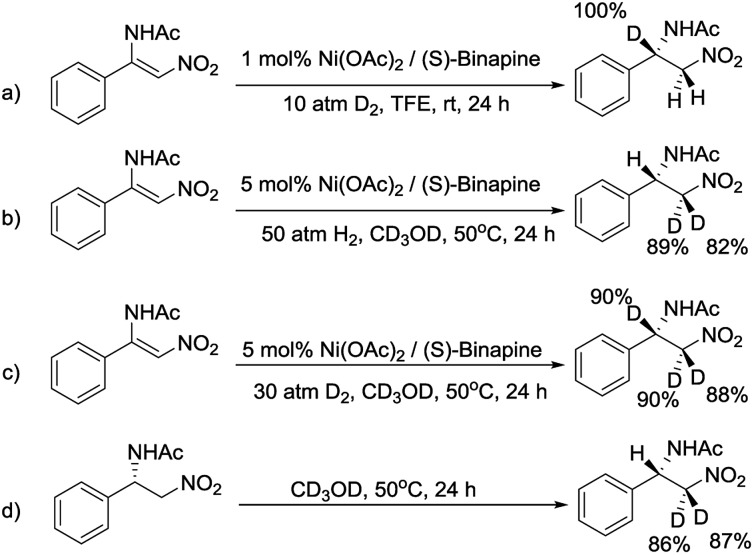
Deuterium labeling studies for the hydrogenation of **1a**.

To gain further insight into the reaction mechanism, DFT calculations using M06-L-D3 and B3LYP-D3 methods have been performed (Scheme 5).^10,11^ Our favored computed catalytic cycle starts with the acetate-assisted heterolytic cleavage of H_2_ to give a Ni(ii)–H intermediate (**III**) with a barrier of ∼23.3–24.1 kcal mol^–1^ in solution. Then, ligand exchange with the nitroolefin substrate takes place, followed by the regio-determining 1,4-addition of the hydride to the β position of the nitroolefin to preferentially form a Ni(ii) intermediate **VI_S1_***via***TSII_S1_**. Such a major pathway requires a lower barrier than the minor pathway by 4.0 kcal mol^–1^, and it forms the (*S*)-product. Subsequently, an AcOH molecule can re-coordinate to the Ni metal and undergo protonation to afford the desired (*S*)-product **2a***via***TSIII_S1_**. These computational results are qualitatively consistent with the observed enantioselectivity and isotope labeling. **2a** could undergo H exchange at the α position with TFE (or AcOH) to give the compound **2a′**. This reaction mechanism for the Ni catalyst is different to that for the Rh-dihydride catalysts, in which alcohol solvents play a critical role in the catalytic system.^12^

**Scheme 5:**
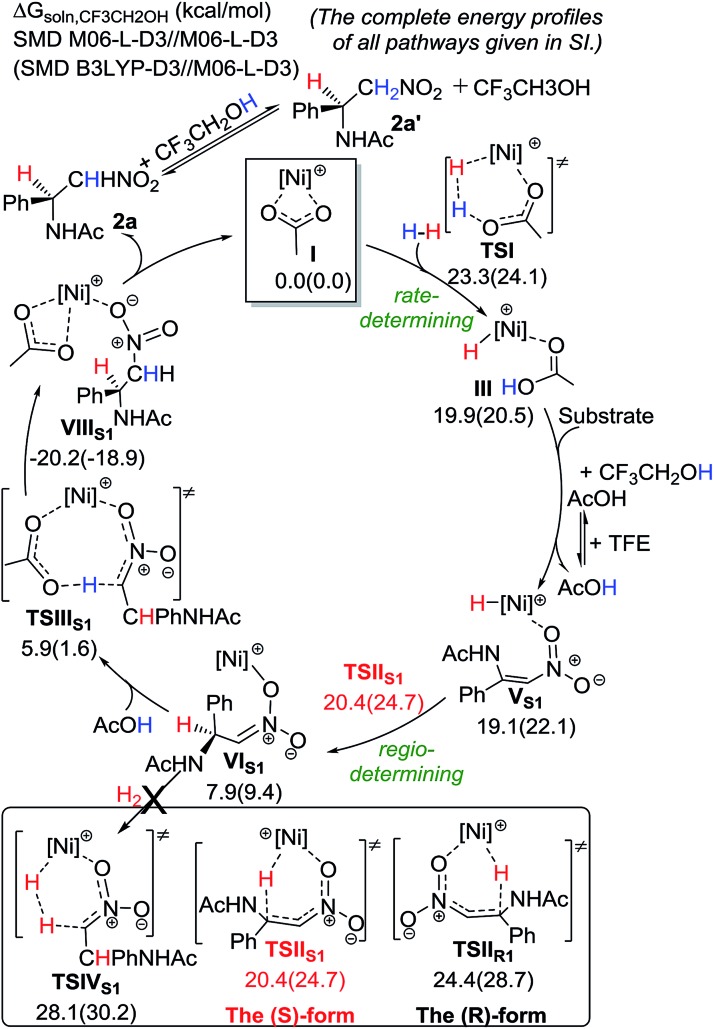
Computed energetics of the proposed catalytic cycle for the Ni-catalyzed hydrogenation of **1a** with the (*S*)-binapine ligand.

## Conclusions

In conclusion, the Ni-catalyzed asymmetric hydrogenation of β-acylamino nitroolefins using H_2_ as the reductant has been achieved, affording chiral β-amino nitroalkanes in high yields and with excellent enantioselectivities. Notably, this catalytic system was carried out under mild conditions and higher turnover numbers were achieved. Compared to noble metal catalysts, such as Rh species, the Ni catalyst is more attractive in the synthesis of chiral β-amino nitroalkanes. Moreover, deuterium labeling and computational studies were performed to reveal a possible mechanism for the Ni-catalyzed asymmetric hydrogenation. A further investigation on Ni-catalyzed asymmetric hydrogenation is ongoing in our laboratory.
