# Supplementary material for: Nickel-catalyzed asymmetric hydrogenation of β-acylamino nitroolefins: an efficient approach to chiral amines
Source: Chem Sci. 2017 Jul 4;8(9):6419–false. doi: 10.1039/c7sc02669b (PMC5632792; doi:10.1039/c7sc02669b)
Supplement: Supplementary file 1 [file SC-008-C7SC02669B-s001.pdf]

This journal is © The Royal Society of Chemistry 2017

*Supporting Information*

# Nickel-Catalyzed Asymmetric Hydrogenation of ß-Acylamino Nitroolefins: An Efficient Approach to Chiral Amines

Table of Contents

1. General Information ........................................ S2

2. General Procedure for Asymmetric Hydrogenation of (Z)-β-Acylamino Nitroolefins 1 ........................................ S2

3. Gram-scale Reaction and S/C Evaluation ........................................ S9

4. The Influence of additive $Bu_4NI$ ........................................ S10

5. Deuterium Labeling Studies ........................................ S11

6. Computational Details ........................................ S11

7. References ........................................ S114

8. NMR and HPLC ........................................ S115

## 1. General Information

All the reactions dealing with air- or moisture-sensitive compounds were carried out in a dry reaction vessel under a positive pressure of nitrogen or in the nitrogen-filled glovebox. Unless otherwise noted, all reagents and solvents were purchased from commercial suppliers and used without further purification. NMR spectra were recorded on Bruker ADVANCE III (400 MHz) spectrometers for $^1H$ NMR and $^{13}C$ NMR. $CDCl_3$ was the solvent used for the NMR analysis, with tetramethylsilane as the internal standard. Chemical shifts were reported upfield to TMS (0.00 ppm) for $^1H$ NMR and relative to $CDCl_3$ (77.0 ppm) for $^{13}C$ NMR. Optical rotation was determined using a Perkin Elmer 343 polarimeter. HPLC analysis was conducted on an Agilent 1260 Series instrument. Column Chromatography was performed with silica gel Merck 60 (300-400 mesh). All new products were further characterized by HRMS. A positive ion mass spectrum of sample was acquired on a Thermo LTQ-FT mass spectrometer with an electrospray ionization source.

All the (*Z*)-β-Acylamino Nitroolefins were pepared according the literature.[1] The absolute configuration of products were determined by comparison of analytical data with the literature.

## 2. General Procedure for Asymmetric Hydrogenation of (Z)-β-Acylamino Nitroolefins 1

In a nitrogen-filled glovebox, the $Ni(OAc)_2$ (0.01 mmol) and (*S*)-Bianpine (0.011 mmol) were dissolved in 1.0 mL anhydrous MeOH and THF (1:1), and the solution was stirred for 30 min at rt. Then the solution was equally divided into 10 vials charged with substrates (0.1 mmol) in anhydrous TFE solution (1.0 mL). The resulting vials were transferred to an autoclave, which was charged with 5-10 atm $H_2$, and the reaction was stirred at rt for 24h. When the reaction was completed, the $H_2$ was released slowly and the solution was passed through a short column of silica gel

with EA. The solution was concentrated under reduced pressure to got the target compound. The chiral compounds were the analyzed by using HPLC on a chiral stationary phase to determine the ee.

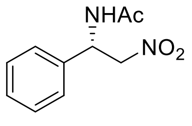

**(*S*)-N-(2-nitro-1-phenylethyl)acetamide 2a**

White solid; 20.7 mg, 99% yield; 99% ee; $[\alpha]_D^{20}$ = +34.20 (c = 0.5, $CHCl_3$); The enantiomeric excess was determined by HPLC on Chiralpak AD-H column, hexane: isopropanol = 90: 10; flow rate = 1.0 mL/min; UV detection at 210 nm; $t_R$ = 11.5 min (major), 20.1 min (minor); $^1$H NMR (400 MHz, $CDCl_3$) δ 7.48 – 7.33 (m, 3H), 7.33 – 7.28 (m, 2H), 6.37 (brs, 1H), 5.70-5.65 (m, 1H), 4.91 (dd, *J* = 13.0, 6.5 Hz, 1H), 4.73 (dd, *J* = 13.0, 5.6 Hz, 1H), 2.05 (s, 3H); $^{13}$C NMR (101 MHz, $CDCl_3$) δ 169.8, 136.3, 129.3, 128.9, 126.4, 78.2, 51.2, 23.3.

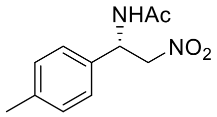

**(*S*)-N-(2-nitro-1-(p-tolyl)ethyl)acetamide 2b**

White solid; 22.0 mg, 99% yield; 99% ee; $[\alpha]_D^{20}$ = +72.30 (c = 1.0, $CHCl_3$); The enantiomeric excess was determined by HPLC on Chiralpak AD-H column, hexane: isopropanol = 90: 10; flow rate = 1.0 mL/min; UV detection at 210 nm; $t_R$ = 11.9 min (major), 16.7 min (minor); $^1$H NMR (400 MHz, $CDCl_3$) δ 7.19 (s, 4H), 6.32 (brs, 1H), 5.67-5.58 (m, 1H), 4.89 (dd, *J* = 12.8, 6.4, 1H), 4.70 (dd, *J* = 12.8, 5.7, 1H), 2.34 (s, 3H), 2.04 (s, 3H); $^{13}$C NMR (101 MHz, $CDCl_3$) δ 169.8, 138.8, 133.3, 129.9, 126.4, 78.2, 51.1, 23.3, 21.1.

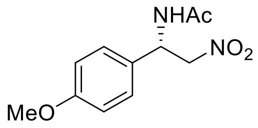

**(*S*)-N-(1-(4-methoxyphenyl)-2-nitroethyl)acetamide 2c**

White solid; 23.7 mg, 99% yield; 99% ee; $[\alpha]_D^{20}$ = +59.00 (c = 0.5, $CHCl_3$); The enantiomeric excess was determined by HPLC on Chiralpak AD-H column, hexane: isopropanol = 85:15; flow rate = 1.0 mL/min; UV detection at 210 nm; $t_R$ = 9.9 min

(major), 15.3 min (minor); $^1$H NMR (400 MHz, $CDCl_3$) δ 7.25 – 7.19 (m, 2H), 7.03 – 6.62 (m, 2H), 6.31 (brs, 1H), 5.63-5.58 (m, 1H), 4.89 (dd, *J* = 12.8, 6.5 Hz, 1H), 4.68 (dd, *J* = 12.8, 5.9 Hz, 1H), 3.79 (s, 3H), 2.03 (s, 3H); $^{13}$C NMR (101 MHz, $CDCl_3$) δ 169.8, 159.8, 128.3, 127.8, 114.6, 78.2, 55.4, 50.9, 23.3.

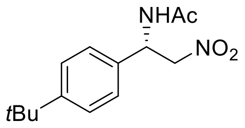


**(*S*)-N-(1-(4-(tert-butyl)phenyl)-2-nitroethyl)acetamide 2d**

White solid; 26.3 mg, 99% yield; 99% ee; $[\alpha]_D^{20}$ = +58.20 (c = 0.5, $CHCl_3$); The enantiomeric excess was determined by HPLC on Chiralpak AD-H column, hexane: isopropanol = 90: 10; flow rate = 1.0 mL/min; UV detection at 210 nm; $t_R$ = 9.0 min (major), 13.4 min (minor); $^1$H NMR (400 MHz, $CDCl_3$) δ 7.44 – 7.35 (m, 2H), 7.26 – 7.20 (m, 2H), 6.34 (brs, 1H), 5.70-5.60 (m, 1H), 4.90 (dd, *J* = 12.9, 6.6 Hz, 1H), 4.71 (dd, *J* = 12.9, 5.8 Hz, 1H), 2.03 (s, 3H), 1.30 (s, 9H); $^{13}$C NMR (101 MHz, $CDCl_3$) δ 169.8, 151.9, 133.3, 126.2, 78.1, 51.0, 34.6, 31.2, 23.3.

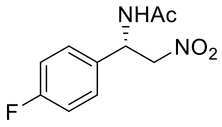


**(*S*)-N-(1-(4-fluorophenyl)-2-nitroethyl)acetamide 2e**

White solid; 22.5 mg, 99% yield; 99% ee; $[\alpha]_D^{20}$ = +43.20 (c = 0.5, $CHCl_3$); The enantiomeric excess was determined by HPLC on Chiralpak AD-H column, hexane: isopropanol = 90: 10; flow rate = 1.0 mL/min; UV detection at 210 nm; $t_R$ = 12.2 min (major), 24.0 min (minor); $^1$H NMR (400 MHz, $CDCl_3$) δ 7.32-7.28 (m, 2H), 7.18 – 6.99 (m, 2H), 6.44 (brs, 1H), 5.68-5.66 (m, 1H), 4.90 (dd, *J* = 13.0, 6.5 Hz, 1H), 4.71 (dd, *J* = 13.0, 5.5 Hz, 1H), 2.06 (s, 3H); $^{13}$C NMR (101 MHz, $CDCl_3$) δ 169.8, 164.0, 161.5, 132.2, 132.2, 128.4, 128.3, 116.4, 116.2, 78.2, 50.7, 23.3.

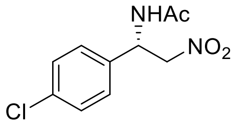


**(*S*)-N-(1-(4-chlorophenyl)-2-nitroethyl)acetamide 2f**

White solid; 24.1 mg, 99% yield; 99% ee; $[\alpha]_D^{20}$ = +52.00 (c = 0.5, $CHCl_3$); The enantiomeric excess was determined by HPLC on Chiralpak AD-H column, hexane:

isopropanol = 90: 10; flow rate = 1.0 mL/min; UV detection at 210 nm; $t_R$ = 13.3 min (major), 25.0 min (minor); $^1$H NMR (400 MHz, $CDCl_3$) δ 7.43 – 7.32 (m, 2H), 7.27-7.23 (m, 2H), 6.51 (brs, 1H), 5.71 – 5.47 (m, 1H), 4.89 (dd, *J* = 13.1, 6.5 Hz, 1H), 4.71 (dd, *J* = 13.1, 5.3 Hz, 1H), 2.06 (s, 3H); $^{13}$C NMR (101 MHz, $CDCl_3$) δ 169.9, 134.9, 134.8, 129.5, 127.9, 78.0, 50.7, 23.3.

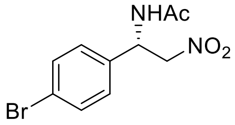


**(*S*)-N-(1-(4-bromophenyl)-2-nitroethyl)acetamide 2g**

White solid; 28.0 mg, 97% yield; 99% ee; $[\alpha]_D^{20}$ = +48.80 (c = 0.5, $CHCl_3$); The enantiomeric excess was determined by HPLC on Chiralpak AD-H column, hexane: isopropanol = 85:15; flow rate = 1.0 mL/min; UV detection at 210 nm; $t_R$ = 8.3 min (major), 15.2 min (minor); $^1$H NMR (400 MHz, $CDCl_3$) δ 7.52-7.49 (m, 2H), 7.24 – 7.10 (m, 2H), 6.50 (brs, 1H), 5.66-5.61 (m, 1H), 4.88 (dd, *J* = 13.1, 6.5 Hz, 1H), 4.71 (dd, *J* = 13.1, 5.3 Hz, 1H), 2.06 (s, 3H); $^{13}$C NMR (101 MHz, $CDCl_3$) δ 169.9, 135.4, 132.4, 128.2, 122.9, 78.0, 50.7, 23.3.

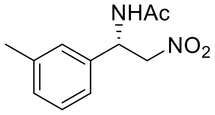


**(*S*)-N-(2-nitro-1-(m-tolyl)ethyl)acetamide 2h**

White solid; 22.1 mg, 99% yield; 99% ee; $[\alpha]_D^{20}$ = +47.00 (c = 0.5, $CHCl_3$); The enantiomeric excess was determined by HPLC on Chiralpak AD-H column, hexane: isopropanol = 85:15; flow rate = 1.0 mL/min; UV detection at 210 nm; $t_R$ = 6.3 min (major), 9.9 min (minor); $^1$H NMR (400 MHz, $CDCl_3$) δ 7.30-7.22 (m, 1H),7.19-7.05 (m, 3H), 6.41 (brs, 1H), 5.70-5.60 (m, 1H), 4.88 (dd, *J* = 12.9, 6.7, 1H), 4.70 (dd, *J* = 12.9, 5.7 Hz, 1H), 2.35 (s, 3H), 2.04 (s, 3H); $^{13}$C NMR (101 MHz, $CDCl_3$) δ 169.8, 139.1, 136.3, 129.6, 129.2, 127.3, 123.4, 78.2, 51.3, 23.3, 21.5.

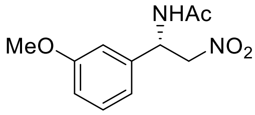


**(*S*)-N-(1-(3-methoxyphenyl)-2-nitroethyl)acetamide 2i**

White solid; 23.6 mg, 99% yield; 99% ee; $[\alpha]_D^{20}$ = +44.00 (c = 0.5, $CHCl_3$); The enantiomeric excess was determined by HPLC on Chiralpak AD-H column, hexane:

isopropanol = 85: 15; flow rate = 1.0 mL/min; UV detection at 210 nm; $t_R$ = 8.8 min (major), 12.9 min (minor); $^1$H NMR (400 MHz, $CDCl_3$) δ 7.38-7.22 (m,, 1H), 7.05 – 6.70 (m, 3H), 6.38 (brs, 1H), 5.70-5.60 (m, 1H), 4.89 (dd, *J* = 12.9, 6.6 Hz, 1H), 4.71 (dd, *J* = 12.9, 5.6 Hz, 1H), 3.80 (s, 3H), 2.05 (s, 3H); $^{13}$C NMR (101 MHz, $CDCl_3$) δ 169.8, 160.1, 137.9, 130.4, 118.4, 113.8, 112.7, 78.1, 55.4, 51.2, 23.3.

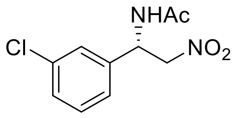


**(*S*)-N-(1-(3-chlorophenyl)-2-nitroethyl)acetamide 2j**

White solid; 23.7 mg, 98% yield; 99% ee; $[\alpha]_D^{20}$ = +46.80 (c = 0.5, $CHCl_3$); The enantiomeric excess was determined by HPLC on Chiralpak AD-H column, hexane: isopropanol = 85: 15; flow rate = 1.0 mL/min; UV detection at 210 nm; $t_R$ = 6.5 min (major), 9.3 min (minor); $^1$H NMR (400 MHz, $CDCl_3$) δ 7.37-7.29 (m, 3H), 7.24-7.16 (m, 1H), 6.61 (brs, 1H), 5.72-5.62 (m, 1H), 4.88 (dd, *J* = 13.2, 6.7 Hz, 1H), 4.71 (dd, *J* = 13.2, 5.2 Hz, 1H), 2.06 (s, 3H); $^{13}$C NMR (101 MHz, $CDCl_3$) δ 169.9, 138.4, 135.2, 130.5, 129.0, 126.7, 124.7, 78.0, 50.7, 23.2.

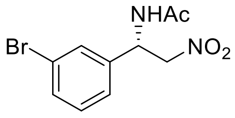


**(*S*)-N-(1-(3-bromophenyl)-2-nitroethyl)acetamide 2k**

White solid; 28.0 mg, 97% yield; 99% ee; $[\alpha]_D^{20}$ = +99.40 (c = 0.5, $CHCl_3$); The enantiomeric excess was determined by HPLC on Chiralpak AD-H column, hexane: isopropanol = 85: 15; flow rate = 1.0 mL/min; UV detection at 210 nm; $t_R$ = 6.8 min (major), 9.6 min (minor); $^1$H NMR (400 MHz, $CDCl_3$) δ 7.58 – 7.33 (m, 2H), 7.32 – 7.09 (m, 2H), 6.63 (brs, 1H), 5.86 – 5.33 (m, 1H), 4.87 (dd, *J* = 13.2, 6.8 Hz, 1H), 4.71 (dd, *J* = 13.2, 5.2 Hz, 1H), 2.06 (s, 3H); $^{13}$C NMR (101 MHz, $CDCl_3$) δ 170.0, 138.7, 132.0, 130.8, 129.6, 125.2, 123.3, 78.0, 50.7, 23.2.

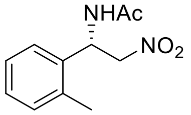


**(*S*)-N-(2-nitro-1-(o-tolyl)ethyl)acetamide 2l**

White solid; 22.1 mg, 99% yield; 99% ee; $[\alpha]_D^{20}$ = +62.60 (c = 0.5, $CHCl_3$); The enantiomeric excess was determined by HPLC on Chiralpak AD-H column, hexane: isopropanol = 85:15; flow rate = 1.0 mL/min; UV detection at 210 nm; $t_R$ = 5.8 min

(major), 6.8 min (minor); $^1$H NMR (400 MHz, $CDCl_3$) δ 7.26 – 7.18 (m, 4H), 6.19 (brs, 1H), 5.95-5.90 (m, 1H), 4.84 (dd, *J* = 12.7, 7.1 Hz, 1H), 4.69 (dd, *J* = 12.7, 6.4 Hz, 1H), 2.45 (s, 3H), 2.01 (s, 3H); $^{13}$C NMR (101 MHz, $CDCl_3$) δ 169.7, 136.4, 134.7, 131.4, 128.9, 126.8, 125.0, 77.3, 47.9, 23.1, 19.3.

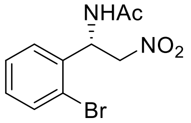


**(*S*)-N-(1-(2-bromophenyl)-2-nitroethyl)acetamide 2m**

White solid; 28.6 mg, 99% yield; 99% ee; $[\alpha]_D^{20}$ = +13.20 (c = 0.5, $CHCl_3$); The enantiomeric excess was determined by HPLC on Chiralpak AD-H column, hexane: isopropanol = 90: 10; flow rate = 1.0 mL/min; UV detection at 210 nm; $t_R$ = 11.1 min (major), 13.9 min (minor); $^1$H NMR (400 MHz, $CDCl_3$) δ 7.64-7.56 (m, 1H), 7.40 – 7.29 (m, 2H), 7.23-7.18 (m, 1H), 6.66 (brs, 1H), 6.02-5.93 (m, 1H), 4.97 (dd, *J* = 13.0, 6.7 Hz, 1H), 4.81 (dd, *J* = 13.0, 4.8 Hz, 1H), 2.07 (s, 3H); $^{13}$C NMR (101 MHz, $CDCl_3$) δ 170.0, 136.3, 133.2, 129.8, 128.1, 128.0, 122.9, 76.8, 51.1, 22.8.

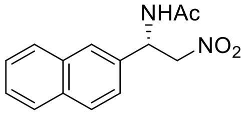


**(*S*)-N-(1-(naphthalen-2-yl)-2-nitroethyl)acetamide 2n**

White solid; 25.7 mg, 99% yield; 99% ee; $[\alpha]_D^{20}$ = +82.80 (c = 0.5, $CHCl_3$); The enantiomeric excess was determined by HPLC on Chiralpak AD-H column, hexane: isopropanol = 85: 15; flow rate = 1.0 mL/min; UV detection at 210 nm; $t_R$ = 9.3 min (major), 16.9 min (minor); $^1$H NMR (400 MHz, $CDCl_3$) δ 7.99 – 7.67 (m, 4H), 7.59 – 7.44 (m, 2H), 7.41-7.35 (m, 1H), 6.51 (brs, 1H), 5.88-5.80 (m, 1H), 4.99 (dd, *J* = 13.0, 6.6 Hz, 1H), 4.81 (dd, *J* = 13.0, 5.6 Hz, 1H), 2.08 (s, 3H); $^{13}$C NMR (101 MHz, $CDCl_3$) δ 168.8, 132.6, 132.1, 128.3, 127.0, 126.7, 125.8, 125.7, 124.7, 122.8, 77.1, 50.4, 22.3.

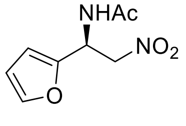


**(*S*)-N-(1-(furan-2-yl)-2-nitroethyl)acetamide 2o**

White solid; 14.0 mg, 87% yield; 96% ee; $[\alpha]_D^{20}$ = +45.40 (c = 0.5, $CHCl_3$); The enantiomeric excess was determined by HPLC on Chiralpak AD-H column, hexane:

isopropanol = 90: 10; flow rate = 1.0 mL/min; UV detection at 210 nm; $t_R$ = 12.1 min (major), 13.3 min (minor); $^1$H NMR (400 MHz, $CDCl_3$) δ 7.39 (m, 1H), 6.43 – 6.27 (m, 3H), 5.77 (dd, *J* = 13.2, 5.5 Hz, 1H), 4.90 (dd, *J* = 13.2, 5.5 Hz, 1H), 4.73 (dd, *J* = 13.2, 5.7 Hz, 1H), 2.07 (s, 3H); $^{13}$C NMR (101 MHz, $CDCl_3$) δ 169.7, 148.9, 143.0, 110.8, 108.1, 76.1, 45.5, 23.2.

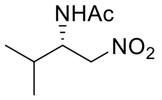


**(*S*)-N-(3-methyl-1-nitrobutan-2-yl)acetamide 2p**

White solid; 17.4 mg, 99% yield; 99% ee; $[\alpha]_D^{20}$ = -50.40 (c = 1.0, $CHCl_3$); The enantiomeric excess was determined by HPLC on Chiralpak AD-H column, hexane: isopropanol = 97: 3; flow rate = 0.9 mL/min; UV detection at 210 nm; $t_R$ = 38.0 min (major), 40.6 min (minor); $^1$H NMR (400 MHz, $CDCl_3$) δ 5.92 (brs, 1H), 4.63 (dd, *J* = 12.8, 5.8 Hz, 1H), 4.54 (dd, *J* = 12.8, 3.9 Hz, 1H), 4.30 – 4.16 (m, 1H), 2.04 (s, 3H), 1.97 – 1.81 (m, 1H), 1.02 (d, *J* =6.7 Hz, 3H), 0.99 (d, *J*=6.7 Hz, 3H ); $^{13}$C NMR (101 MHz, $CDCl_3$) δ 170.1, 76.6, 53.2, 29.7, 23.3, 19.5, 19.1.

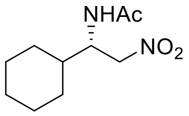


**(*S*)-N-(1-cyclohexyl-2-nitroethyl)acetamide 2q**

White solid; 21.3 mg, 99% yield; 99% ee; $[\alpha]_D^{20}$ = -31.70 (c = 1.0, $CHCl_3$); The enantiomeric excess was determined by HPLC on Chiralpak AD-H column, hexane: isopropanol = 90: 10; flow rate = 1.0 mL/min; UV detection at 210 nm; $t_R$ = 7.6 min (major), 10.4 min (minor); $^1$H NMR (400 MHz, $CDCl_3$) δ 5.96 (brs, 1H), 4.65 (dd, *J* = 13.0, 5.7 Hz, 1H), 4.54 (dd, *J* = 13.0, 3.8 Hz, 1H), 4.23 (m, 1H), 2.03 (s, 3H), 1.84 – 1.61 (m, 5H), 1.62 – 1.46 (m, 1H), 1.31 – 1.11 (m, 3H), 1.10 – 0.77 (m, 2H); $^{13}$C NMR (101 MHz, $CDCl_3$) δ 170.0, 76.3, 52.3, 38.9, 29.8, 29.5, 25.9, 25.7, 23.3.

## 3. Gram-scale Reaction and S/C Evaluation

1) 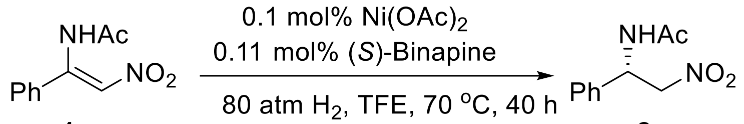


**1a** **2a**

57.4% conv, 99% ee

In a nitrogen-filled glovebox, the $Ni(OAc)_2$ (0.01 mmol) and (*S*)-Bianpine (0.011 mmol) were dissolved in 1.0 mL anhydrous MeOH and THF (1:1), and the solution was stirred for 30 min at rt. Then the catalyst solution (0.2 mL) was transferred by syringe into the vials charged with substrates **1a** (0.414g, 2 mmol) in anhydrous TFE solution (5.0 mL). The resulting vials were transferred to an autoclave, which was charged with 80 atm $H_2$, and the reaction was stirred at 70°C for 40h. When the reaction was completed, the $H_2$ was released slowly and the solution was passed through a short column of silica gel with EA. The solution was concentrated under reduced pressure to got the target compound. The conversion of **1a** was confirmed by $^1$H NMR. The chiral compounds were the analyzed by using HPLC on a chiral stationary phase to determine the ee.

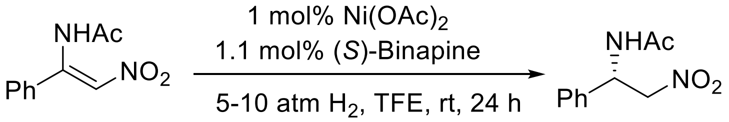


**1a** **2a**

1.1 g (5.3 mol)

1.1 g.
99% yield, 99% ee

In a nitrogen-filled glovebox, the $Ni(OAc)_2$ (0.05 mmol) and (*S*)-Bianpine (0.055 mmol) were dissolved in 1.0 mL anhydrous MeOH and THF (1:1), and the solution was stirred for 30 min at rt. Then the catalyst solution was transferred by syringe into the vials charged with substrates **1a** (1.1g, 5.3 mmol) in anhydrous TFE solution (30.0 mL). The resulting vials were transferred to an autoclave, which was charged with 10 atm $H_2$, and the reaction was stirred at rt for 24h. When the reaction was completed, the $H_2$ was released slowly and the solution was passed through a short column of silica gel with EA. The solution was concentrated under reduced pressure to got the

target compound. The chiral compounds were the analyzed by using HPLC on a chiral stationary phase to determine the ee. The yield of **2a** is 99%, ee is 99%.

## 4. The Influence of additive $Bu_4NI$

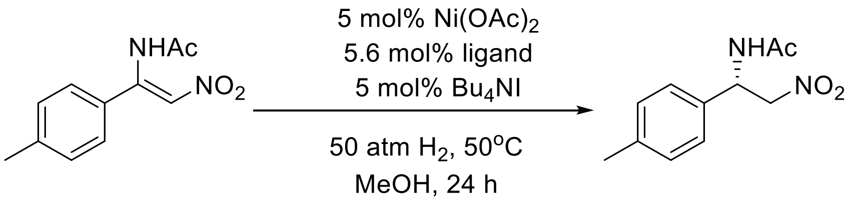


| Entry | Ligand | Additive | Conv. (%) | Ee (%) |
|---|---|---|---|---|
| 1 | (*Rc,Sp*)-DuanPhos | $Bu_4NI$ | 71 | 68 |
| 2 | (*Rc,Sp*)-DuanPhos | - | 85 | 65 |
| 3 | (*R,R*)-QuinoxP* | $Bu_4NI$ | 80 | 95 |
| 4 | (*R,R*)-QuinoxP* | - | >99 | 97 |
| 5 | (*S*)-DuPhos | $Bu_4NI$ | >99 | 30 |
| 6 | (*S*)-DuPhos | - | >99 | 37 |

## 5. Deuterium Labeling Studies

(1) Asymmetric hydrogenation of **1a** in TFE solution under $D_2$: In a nitrogen-filled glovebox, the $Ni(OAc)_2$ (0.01 mmol) and (*S*)-Bianpine (0.011 mmol) were dissolved in 1.0 mL anhydrous MeOH and THF (1:1), and the solution was stirred for 30 min at rt. Then the solution was equally divided into 10 vials charged with **1a** (0.1 mmol) in anhydrous TFE solution (1.0 mL). The resulting vials were transferred to an autoclave, which was charged with 5-10 atm $D_2$, and the reaction was stirred at rt for 24h. When the reaction was completed, the $D_2$ was released slowly and the solution was passed through a short column of silica gel with EA. The solution was concentrated under reduced pressure to got the target compound. The chiral compounds were the analyzed by using HPLC on a chiral stationary phase to determine the ee.

(2) Asymmetric hydrogenation of **1a** in $CD_3OD$ solution under $H_2$ ($D_2$): In a nitrogen-filled glovebox, the $Ni(OAc)_2$ (0.01 mmol) and (*S*)-Bianpine (0.011 mmol) were dissolved in 1.0 mL anhydrous $CD_3OD$ and THF (1:1), and the solution was stirred for 30 min at rt. Then the solution was equally divided into 10 vials charged with **1a** (0.1 mmol) in anhydrous $CD_3OD$ solution (1.0 mL). The resulting vials were transferred to an autoclave, which was charged with 30-50 atm $H_2$ ($D_2$), and the reaction was stirred at 50 °C for 24h. When the reaction was completed, the $D_2$ was released slowly and the solution was passed through a short column of silica gel with EA. The solution was concentrated under reduced pressure to got the target compound. The chiral compounds were the analyzed by using HPLC on a chiral stationary phase to determine the ee.

(3) Asymmetric hydrogenation of **2a** in $CD_3OD$ solution under $H_2$: A vials charged with **2a** (0.1 mmol) in anhydrous $CD_3OD$ solution (1.0 mL). The resulting vials were transferred to an autoclave, which was charged with 30 $H_2$, and the reaction was stirred at 50 °C for 24h. When the reaction was completed, the $H_2$ was released slowly and the solution was passed through a short column of silica gel with EA. The

solution was concentrated under reduced pressure to got the target compound. The chiral compounds were the analyzed by using HPLC on a chiral stationary phase to determine the ee.

$^{1}$H NMR spectrum of **2a**: asymmetric hydrogenation of **1a** under TFE and 10atm $D_2$

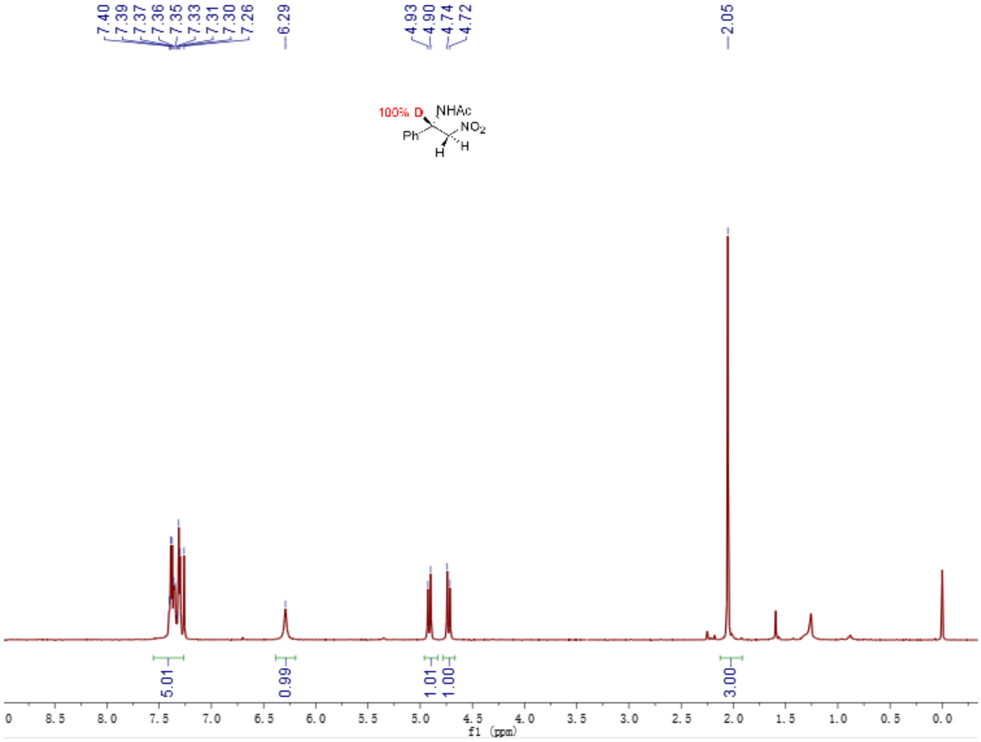


$^{1}$H NMR spectrum of **2a:** asymmetric hydrogenation of **1a** $CD_3OD$ and 50 atm $H_2$

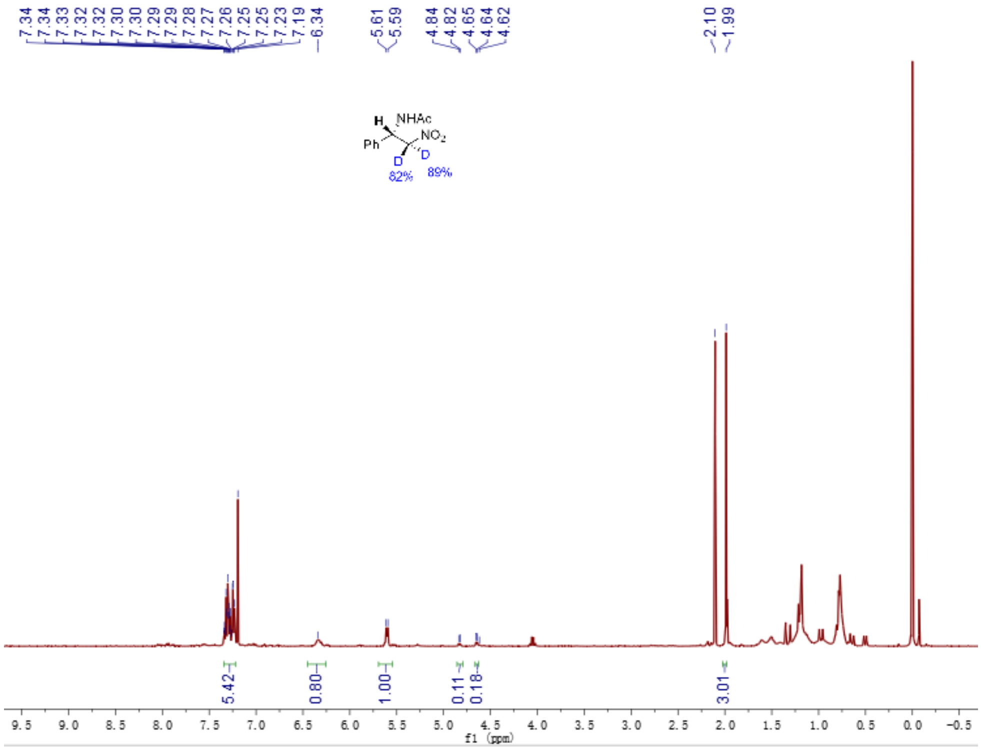

$^{1}$H NMR spectrum of **2a:** asymmetric hydrogenation of **1a** $CD_3OD$ and 30 atm $D_2$

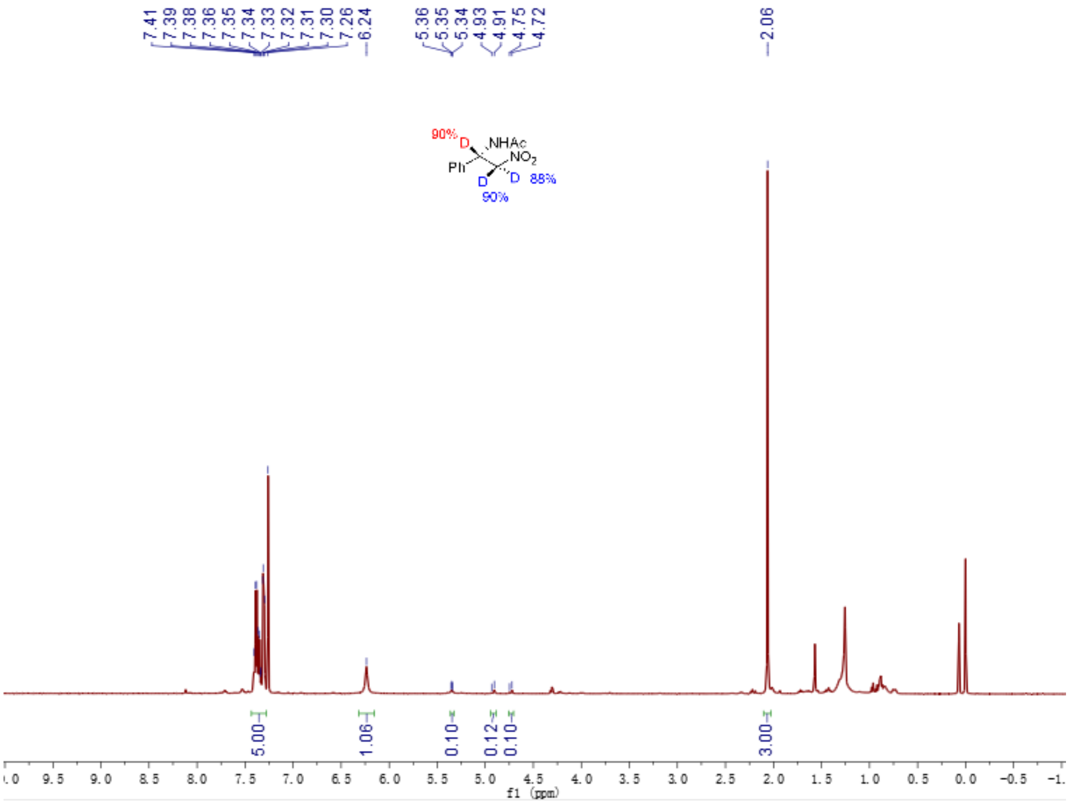


$^{1}$H NMR spectrum of **2a**: **2a** was stirred in $CD_3OD$ and 30 atm $H_2$

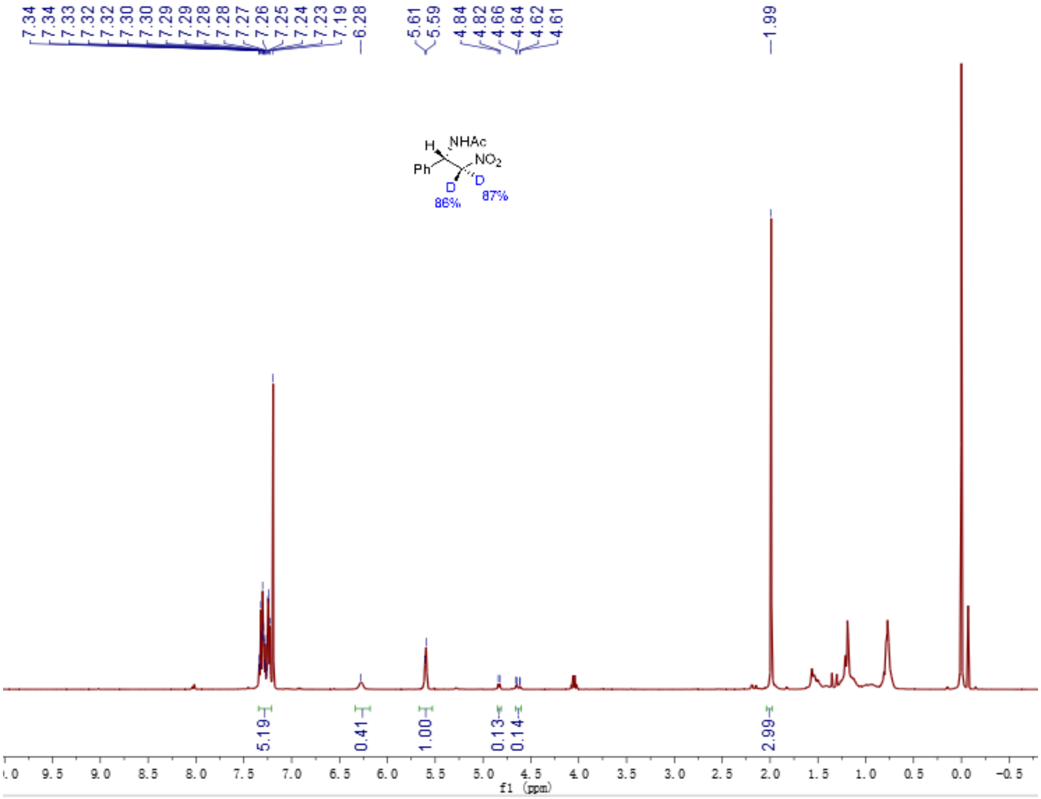

## 6. Computational Details

We carried out density functional theory (DFT) calculations to understand the mechanism of the Ni-catalyzed asymmetric hydrogenation of (*Z*)-*N*-(2-nitro-1-(p-tolyl)vinyl)acetamide **1b** using a (S)-Binapine ligand. M06-L[3] method combined with 6-31G* basis set[4] and Grimme's dispersion correction[5] was used to optimize all the structures in gas phase. The vibrational frequency calculations were computed on the optimized structures at the same level of theory to check whether every optimized structure is either an energy minimum or a transition state. The effect of the solvent (2,2,2-TriFluoroEthanol, TFE) was then included by single-point calculations with an implicit solvent model SMD[6] (by the M06L-D3 and B3LYP-D3[7] methods). All the computations were carried out by Gaussian 09 package.[8] All 3D images of the optimized structures were illustrated by CYLview.[9]

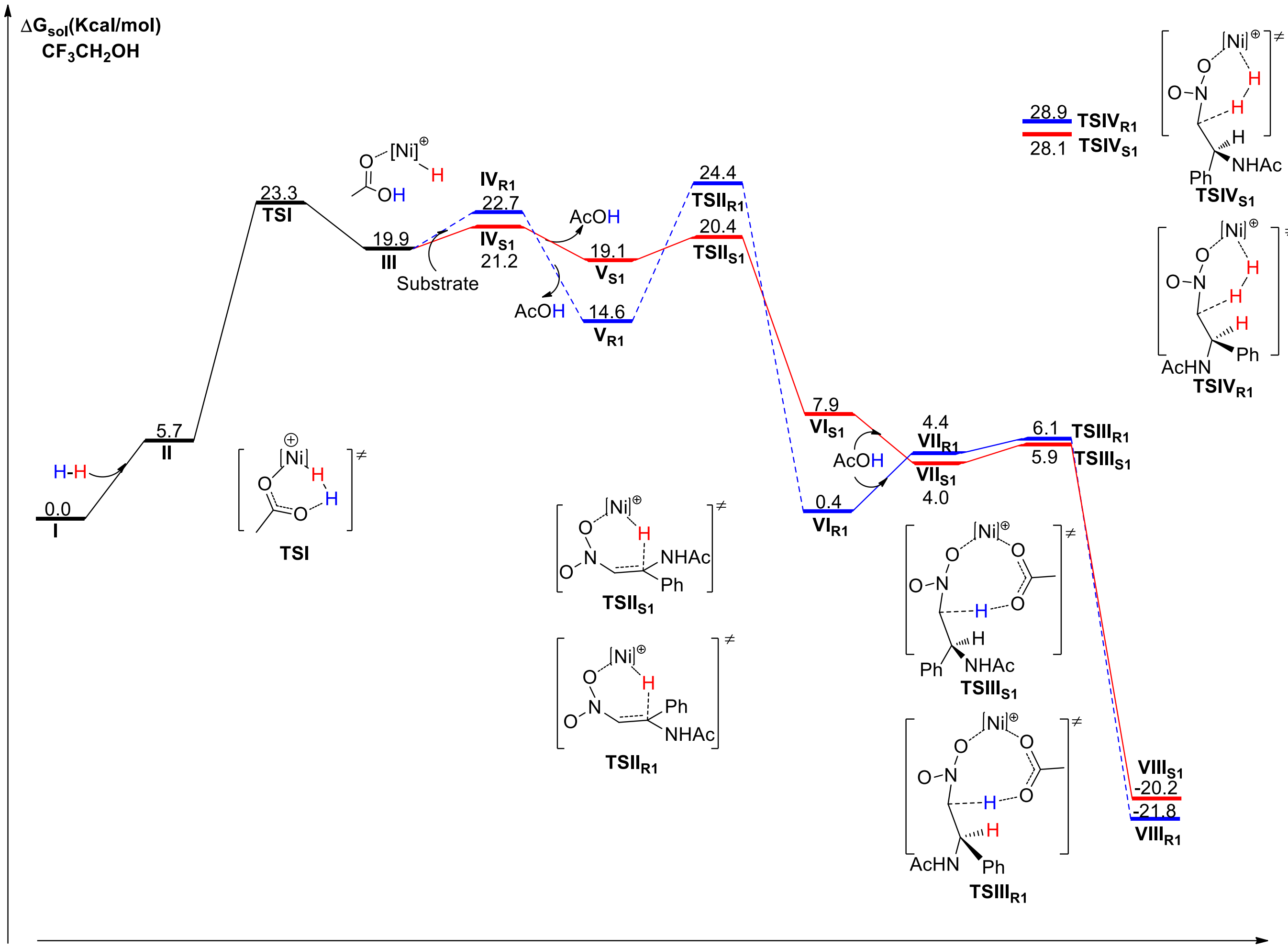


**Figure S1.** Free energy profiles of the of the Ni-catalyzed asymmetric hydrogenation reaction pathways for the S1, & R1 (most favorable 1,4-addition) isomers in solution by the M06L/6-31G* method.

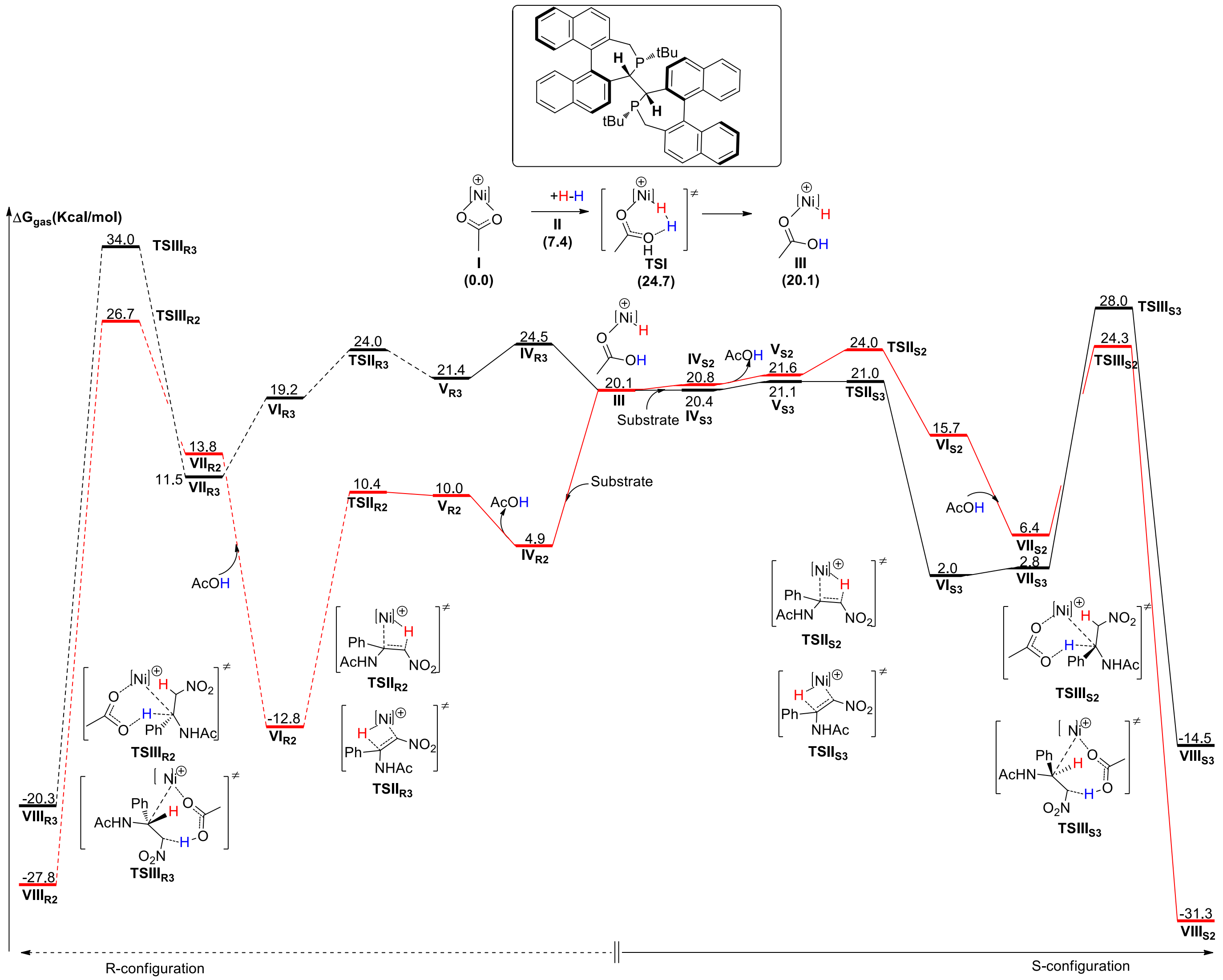


**Figure S2.** Free energy profiles of the of the Ni-catalyzed asymmetric hydrogenation reaction pathways for the S2, S3 & R2, R3 (less favorable) isomers in gas phase by the M06L/6-31G* method.

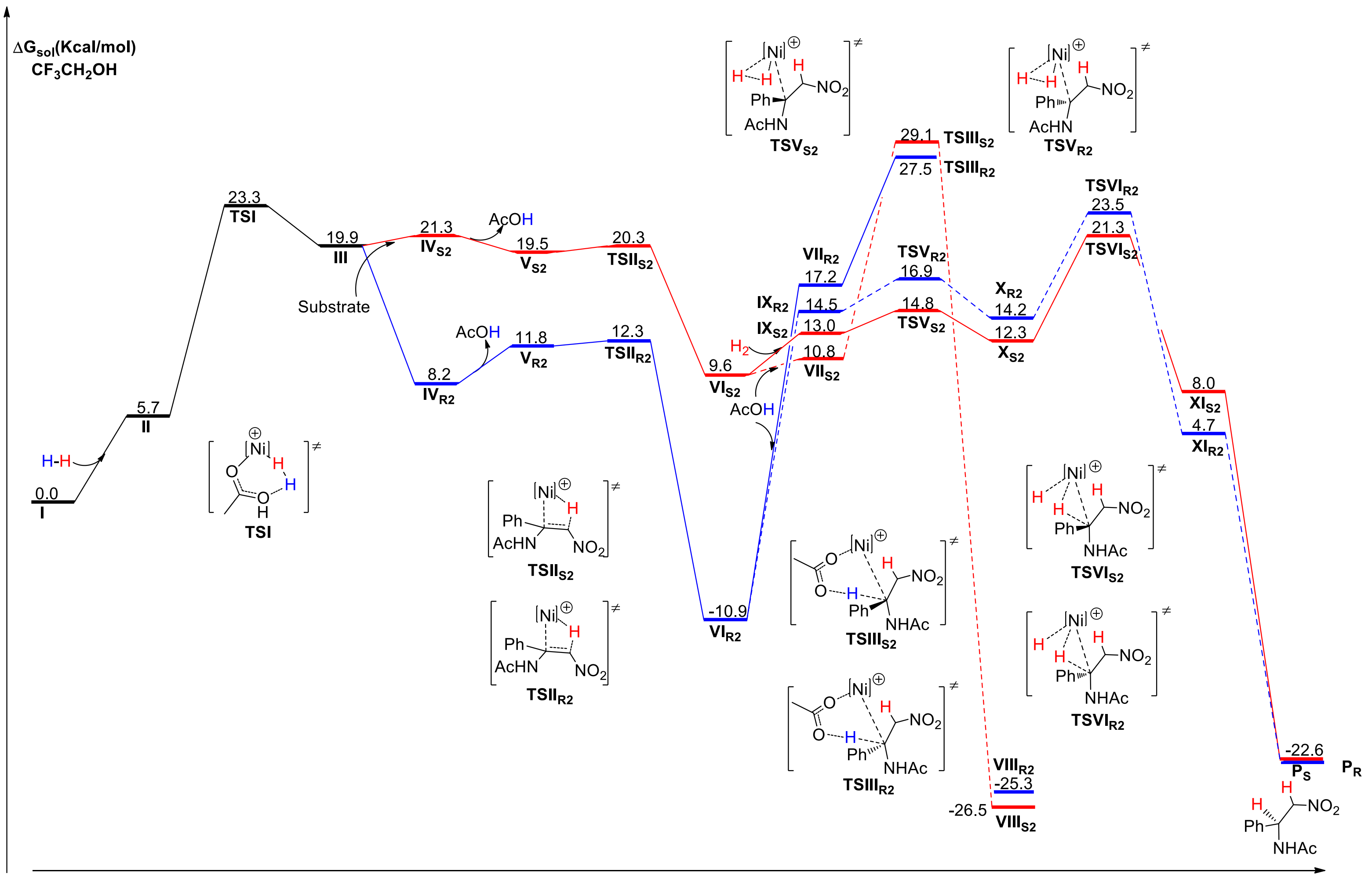


**Figure S3.** Free energy profiles of the of the Ni-catalyzed asymmetric hydrogenation reaction pathways for the S2, S3 & R2, R3 (less favorable) isomers in solution by the M06L/6-31G* method.

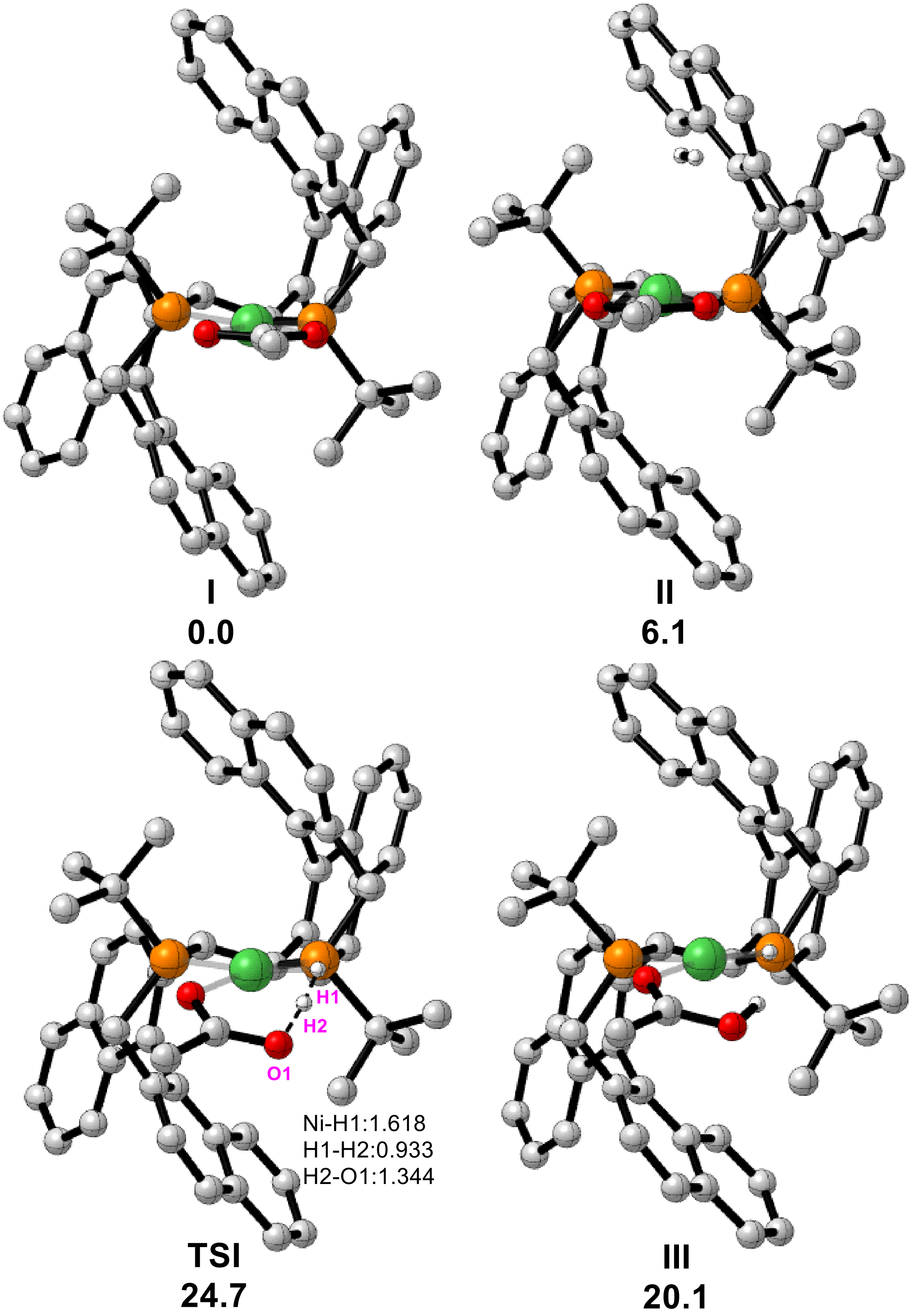


**Figure S4.** Optimized structures of the key intermediates and transition states in the cationic form. The key bond lengths (in angstrom) and relative free energies (in kcal/mol) in gas phase are given. Unimportant hydrogen atoms are not shown for clarity.

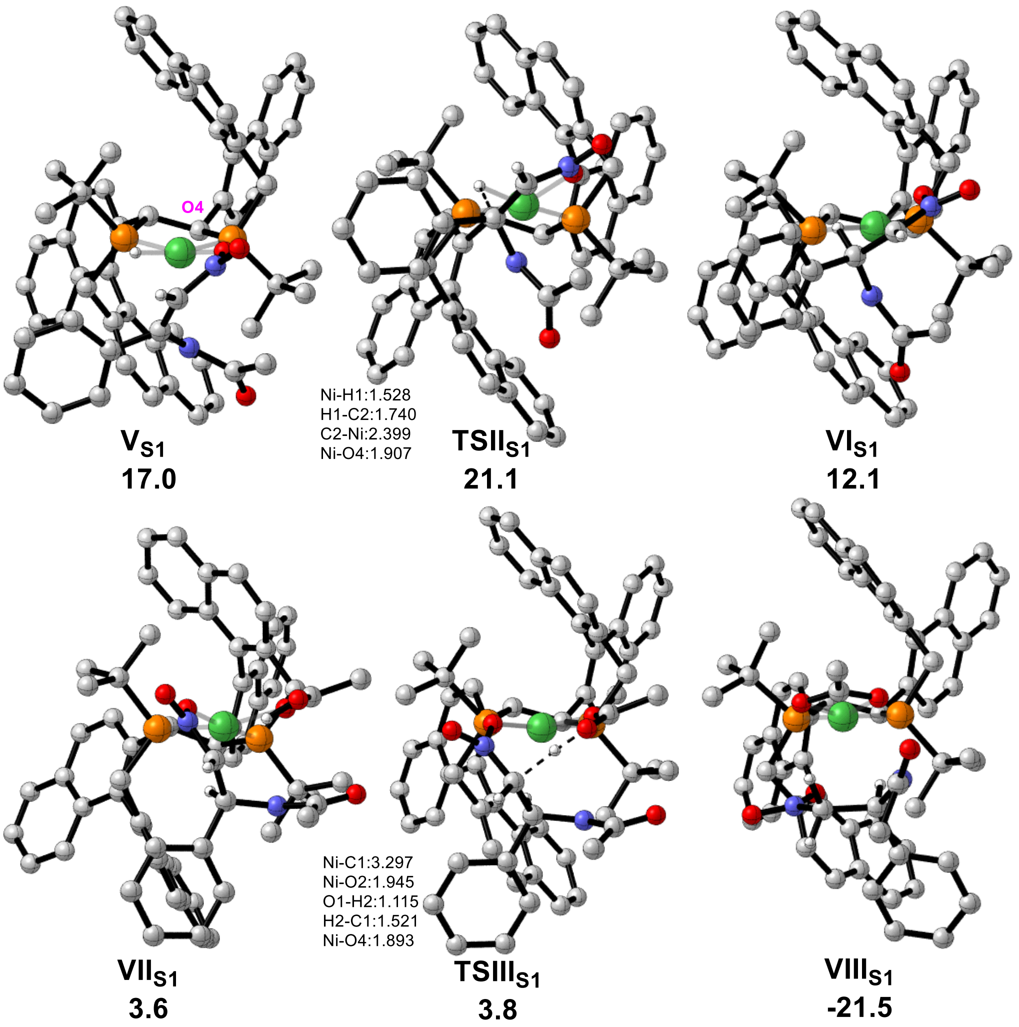


**Figure S5.** Optimized structures of the key intermediates and transition states for the S1 isomer. The key bond lengths (in angstrom) and relative free energies (in kcal/mol) in gas phase are given. Unimportant hydrogen atoms are not shown for clarity.

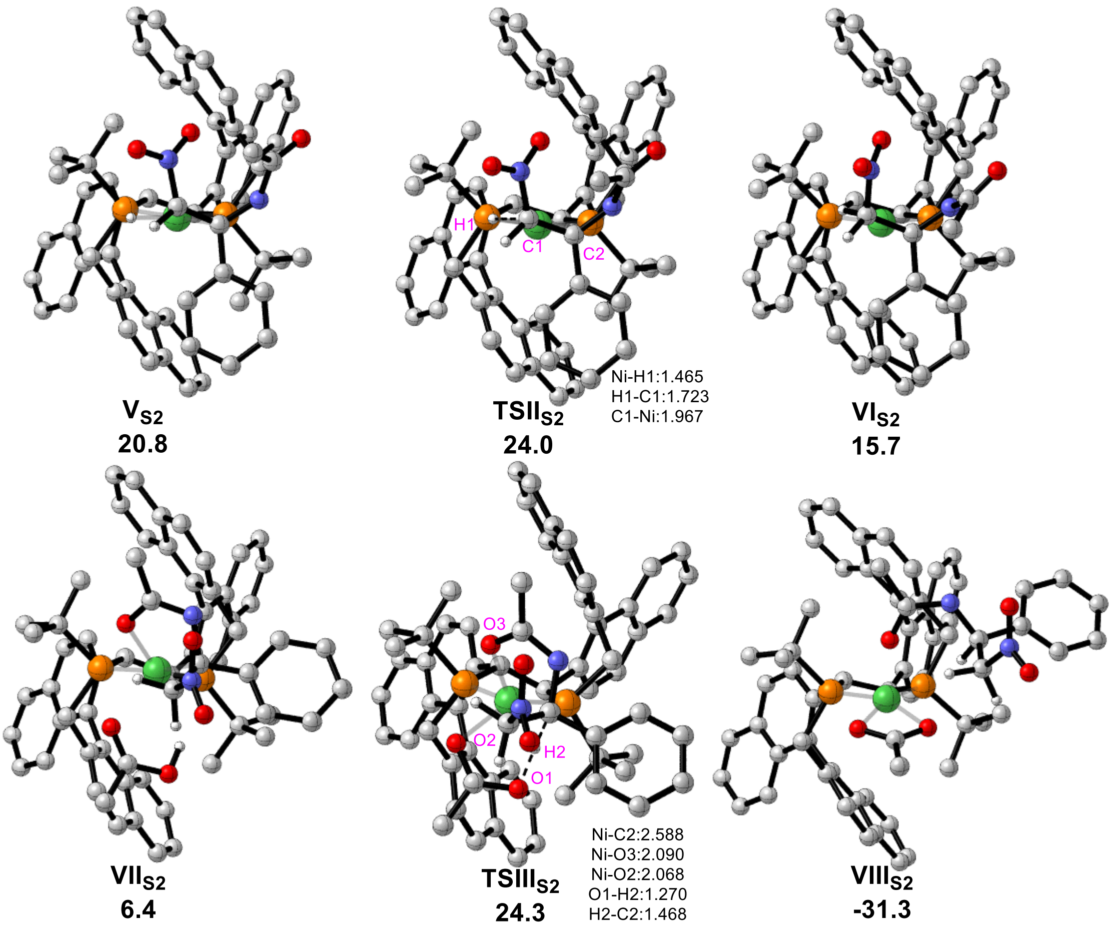


**Figure S6.** Optimized structures of the key intermediates and transition states for the S2 isomer. The key bond lengths (in angstrom) and relative free energies (in kcal/mol) in gas phase are given. Unimportant hydrogen atoms are not shown for clarity.

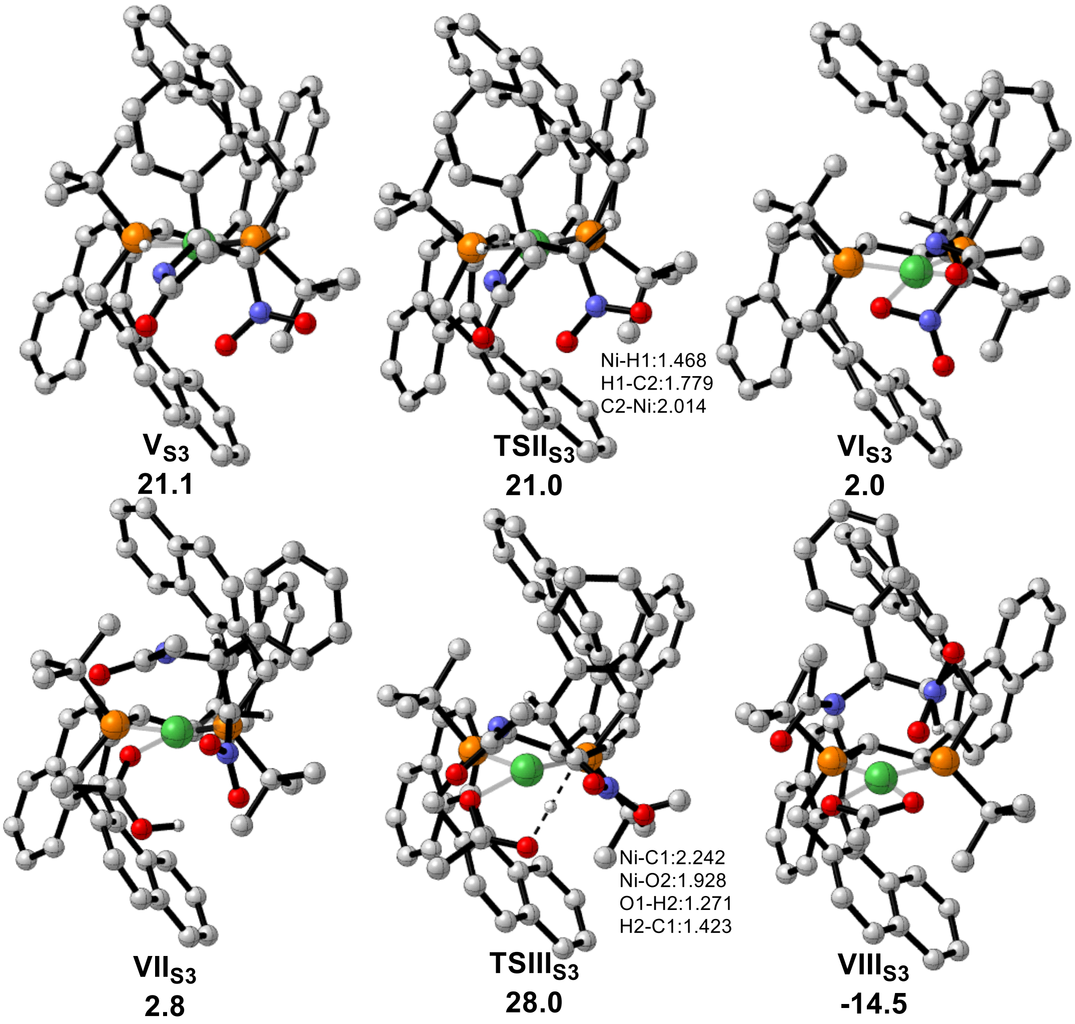


**Figure S7.** Optimized structures of the key intermediates and transition states for the S3 isomer. The key bond lengths (in angstrom) and relative free energies (in kcal/mol) in gas phase are given. Unimportant hydrogen atoms are not shown for clarity.

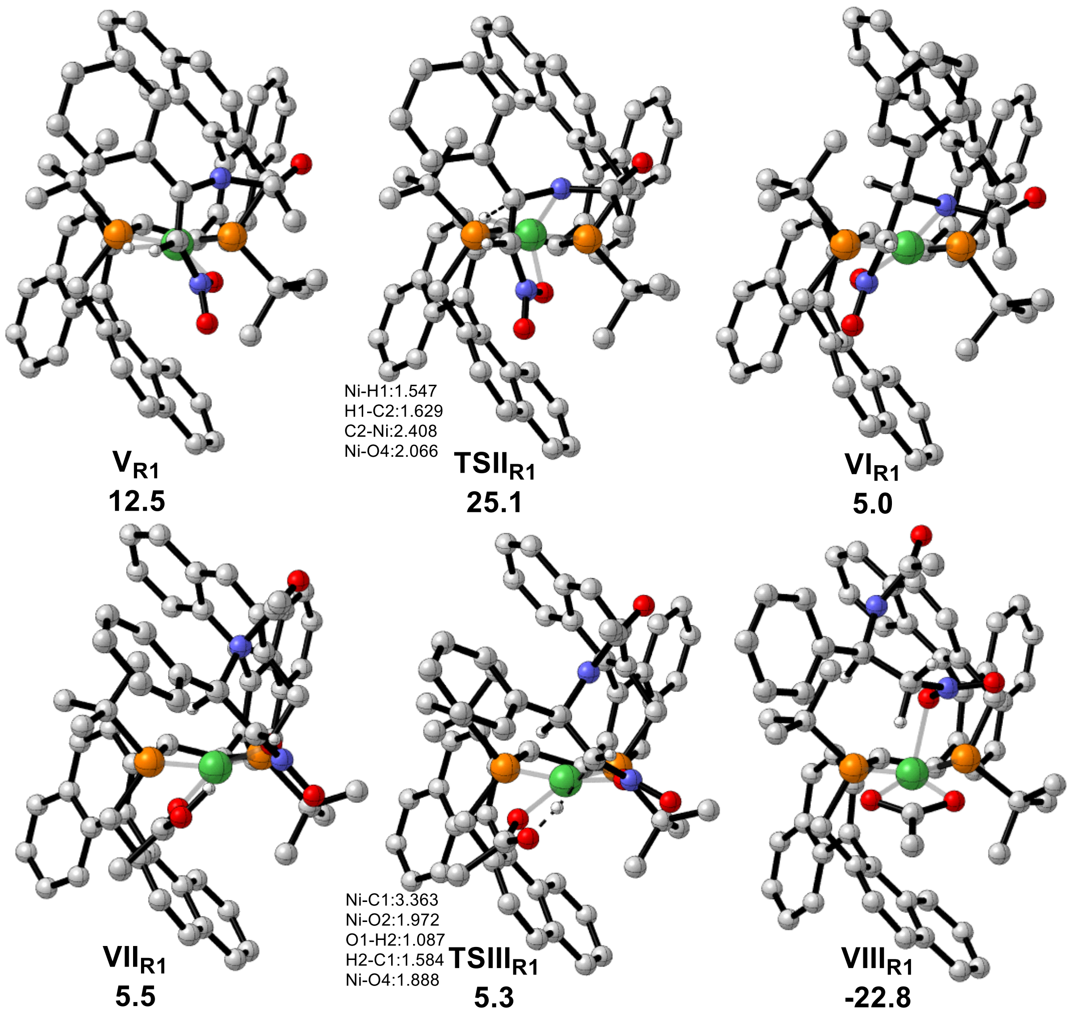


**Figure S8.** Optimized structures of the key intermediates and transition states for the R1 isomer. The key bond lengths (in angstrom) and relative free energies (in kcal/mol) in gas phase are given. Unimportant hydrogen atoms are not shown for clarity.

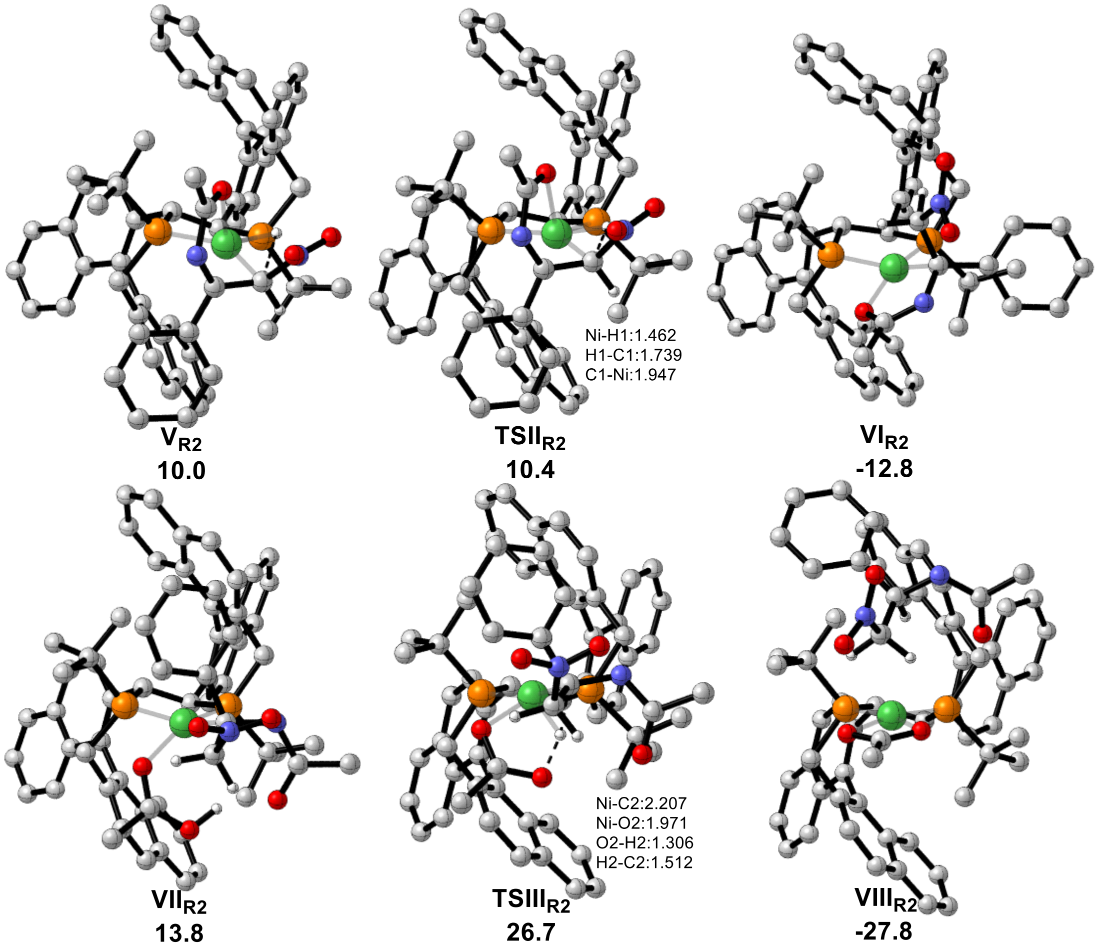


**Figure S9.** Optimized structures of the key intermediates and transition states for the R2 isomer. The key bond lengths (in angstrom) and relative free energies (in kcal/mol) in gas phase are given. Unimportant hydrogen atoms are not shown for clarity.

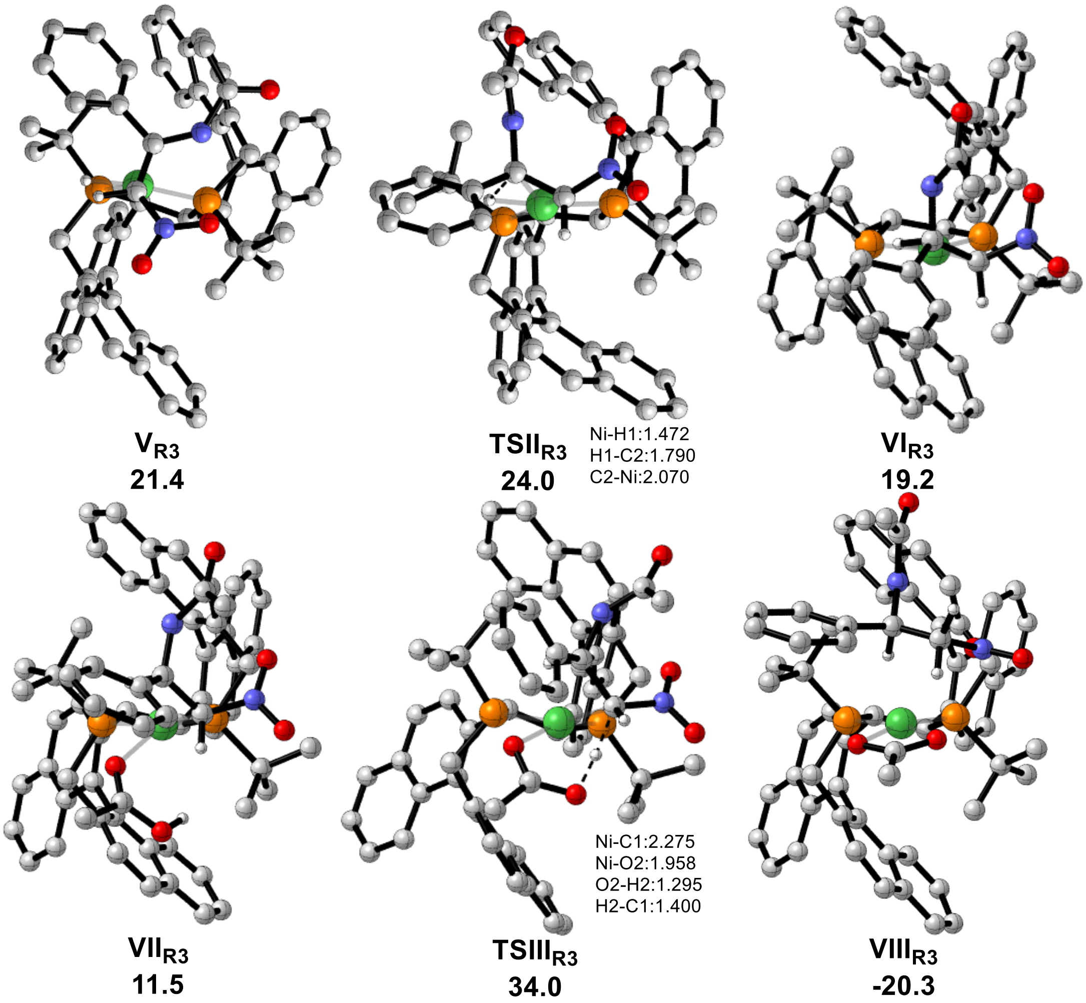


**Figure S10.** Optimized structures of the key intermediates and transition states for the R3 isomer. The key bond lengths (in angstrom) and relative free energies (in kcal/mol) in gas phase are given. Unimportant hydrogen atoms are not shown for clarity.

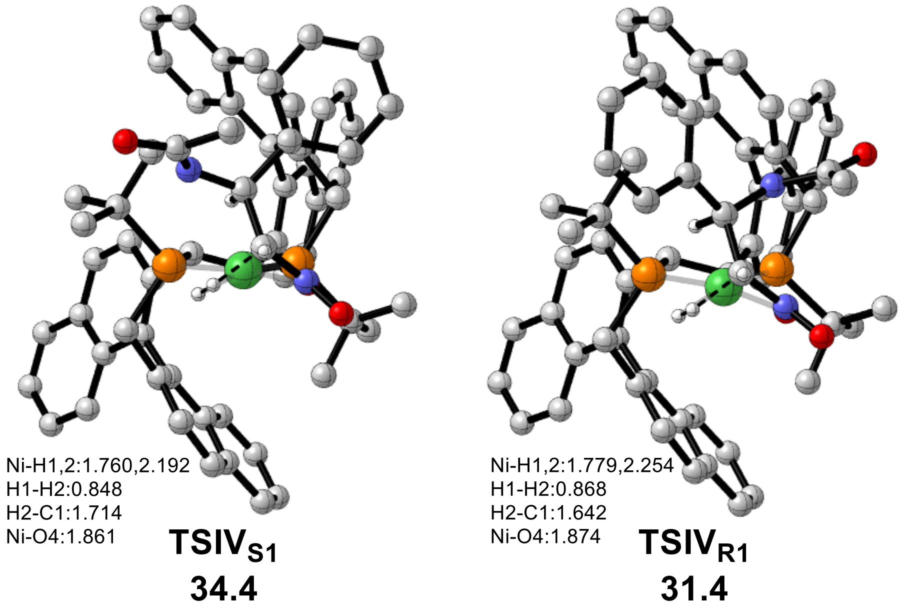


**Figure S11.** Optimized structures of the transition states involving $H_2$ splitting for the S1 & R1 isomers. The key bond lengths (in angstrom) and relative free energies (in kcal/mol) in gas phase are given. Unimportant hydrogen atoms are not shown for clarity.

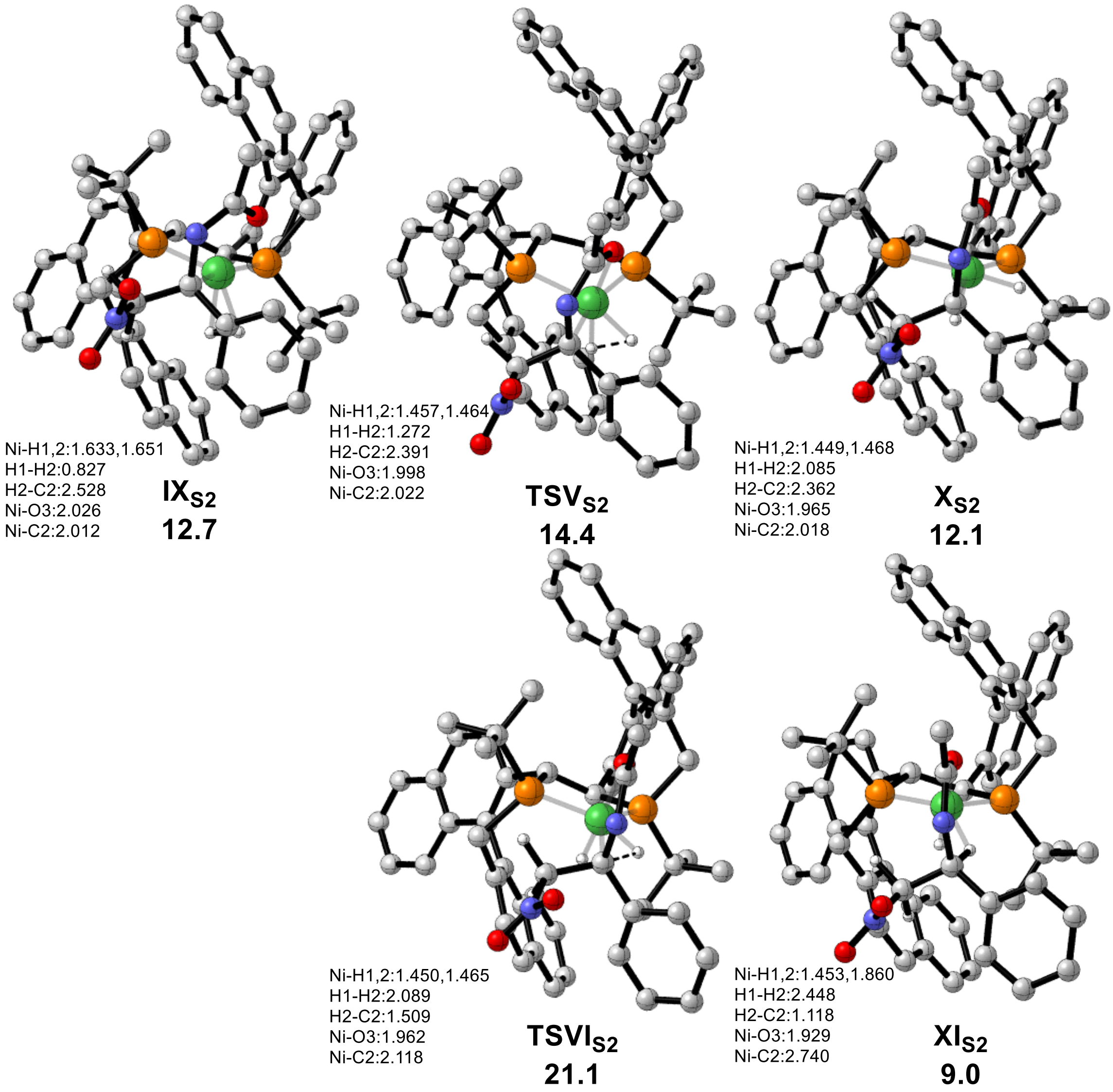


**Figure S12.** Optimized structures of the transition states involving $H_2$ splitting for the S2 isomer. The key bond lengths (in angstrom) and relative free energies (in kcal/mol) in gas phase are given. Unimportant hydrogen atoms are not shown for clarity.

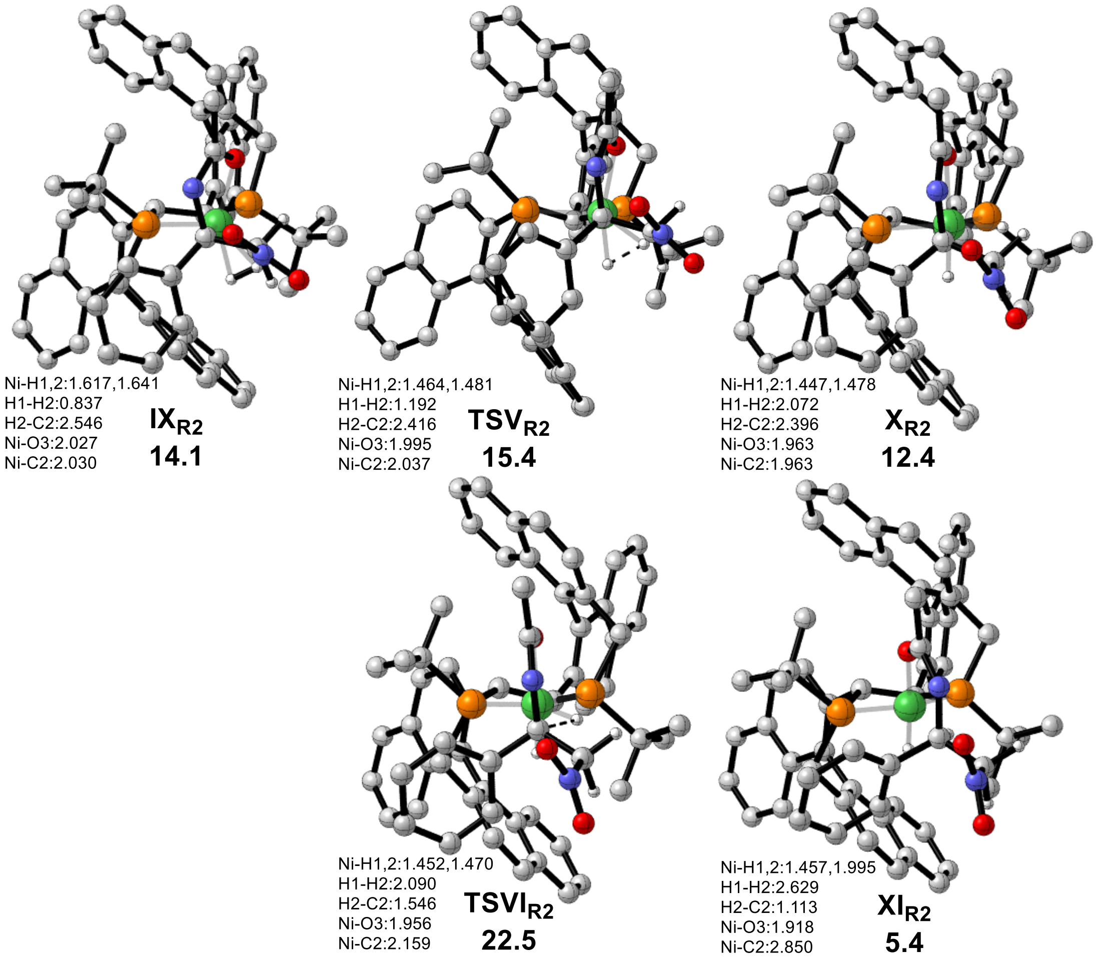


**Figure S13.** Optimized structures of the transition states involving $H_2$ splitting for the R2 isomer. The key bond lengths (in angstrom) and relative free energies (in kcal/mol) in gas phase are given. Unimportant hydrogen atoms are not shown for clarity.

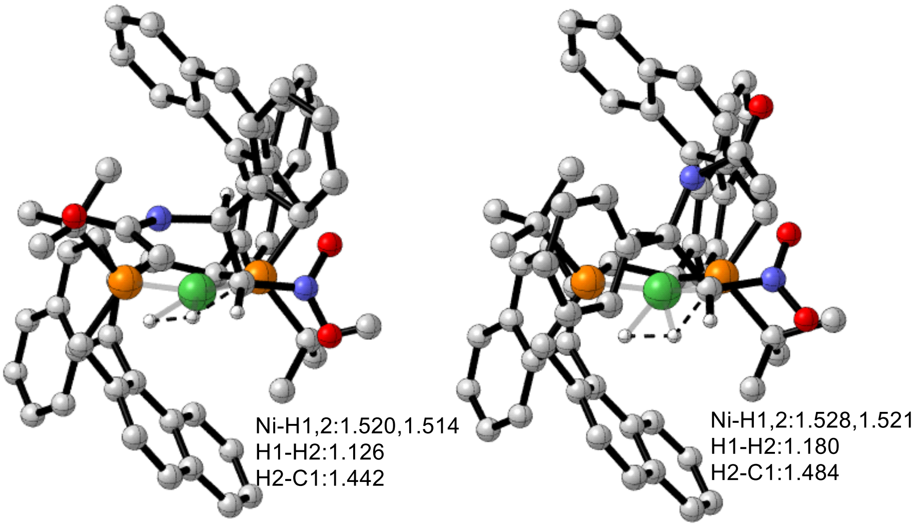


**$TSIV_{S3}$**
**47.0**

**$TSIV_{R3}$**
**49.7**

**Figure S14.** Optimized structures of the transition states involving $H_2$ splitting for the S3 & R3 isomers. The key bond lengths (in angstrom) and relative free energies (in kcal/mol) in gas phase are given. Unimportant hydrogen atoms are not shown for clarity.

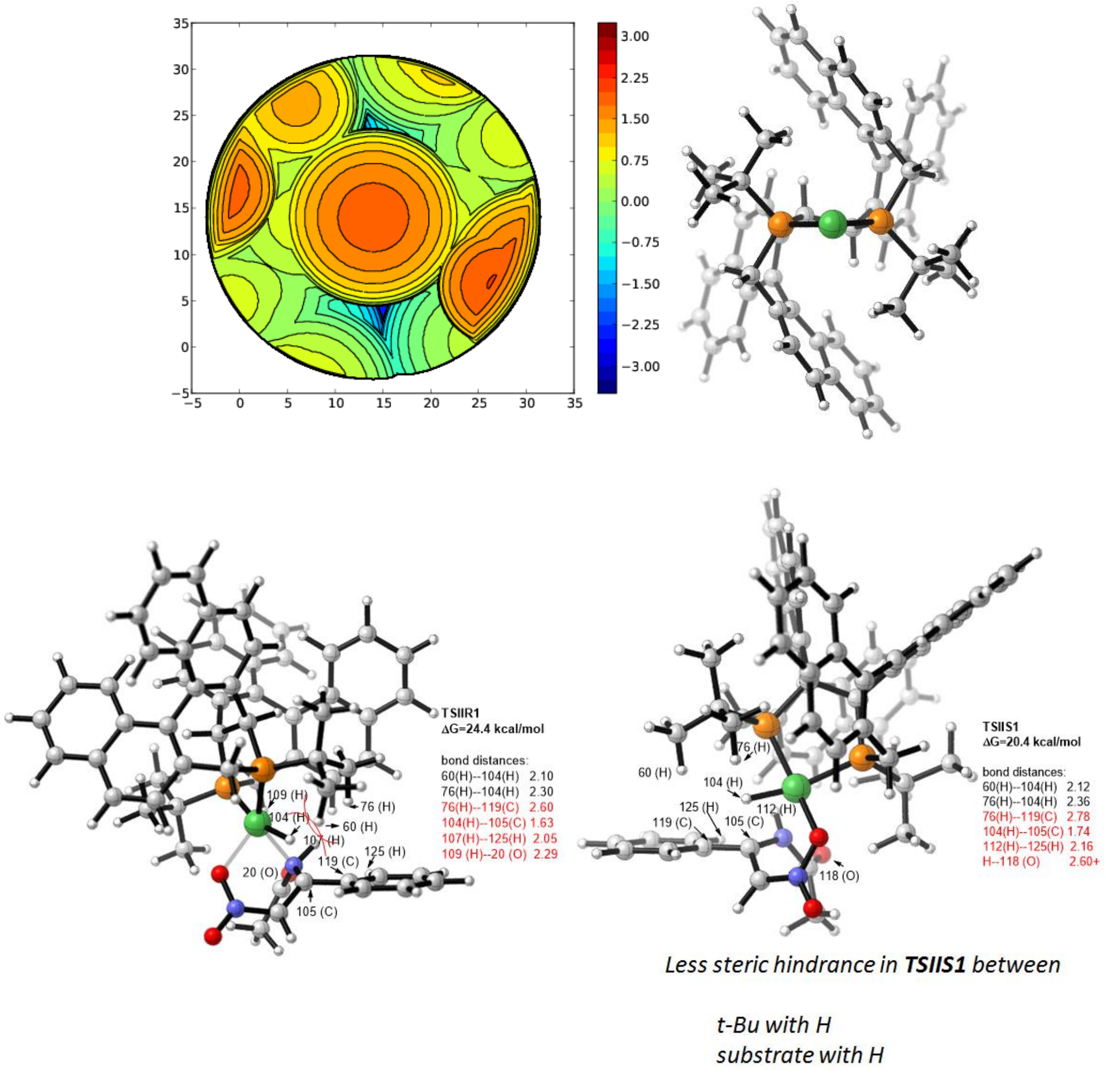


**Figure S15**. (top) The steric map[10] for **TSIIS1** and (bottom) The regio-determining transition states **TSIIR1** (left) and **TSIIS1** (right) to illustrate the larger steric effect on the stereoselectivity in **TSIIR1**.

**Table S1.** The absolute (in Hartree) and relative (in kcal/mol) energies for S isomer in gas phase by M06L/6-31G* method.

| | E | E+ZPE | G | $\Delta E_{gas}$ | $\Delta E_{gas+ZPE}$ | $\Delta G_{gas}$ |
|---|---|---|---|---|---|---|
| **$H_2$** | -1.167913 | -1.158003 | -1.169494 | | | |
| **Sub** | -722.083326 | -721.890567 | -721.931901 | | | |
| **AcOH** | -229.056125 | -228.993765 | -229.020702 | | | |
| **I** | -4429.445753 | -4428.534385 | -4428.61447 | 0.0 | 0.0 | 0.0 |
| **II** | -4430.616853 | -4429.691448 | -4429.774171 | -2.0 | 0.6 | 6.1 |
| **TSI** | -4430.589486 | -4429.664058 | -4429.744656 | 15.2 | 17.8 | 24.7 |
| **III** | -4430.599646 | -4429.669984 | -4429.751874 | 8.8 | 14.1 | 20.1 |
| **S1 isomer** | | | | | | |
| **$IV_{S1}$** | -5152.712677 | -5151.587988 | -5151.685241 | -9.8 | -3.2 | 19.2 |
| **$V_{S1}$** | -4923.638698 | -4922.577172 | -4922.668052 | 1.4 | 7.5 | 17.0 |
| **$TSII_{S1}$** | -4923.629306 | -4922.571295 | -4922.66147 | 7.3 | 11.2 | 21.1 |
| **$VI_{S1}$** | -4923.645825 | -4922.583676 | -4922.675879 | -3.1 | 3.5 | 12.1 |
| **$VII_{S1}$** | -5152.741101 | -5151.61341 | -5151.710159 | -27.7 | -19.1 | 3.6 |
| **$TSIII_{S1}$** | -5152.740304 | -5151.615022 | -5151.709737 | -27.2 | -20.1 | 3.8 |
| **$VIII_{S1}$** | -5152.784518 | -5151.65355 | -5151.750064 | -54.9 | -44.3 | -21.5 |
| the second step as the $H_2$ splitting | | | | | | |
| **$TSIV_{S1}$** | -4924.795065 | -4923.717246 | -4923.809842 | 8.6 | 18.8 | 34.4 |
| **S2 isomer** | | | | | | |
| **$IV_{S2}$** | -5152.715133 | -5151.587572 | -5151.682768 | -11.4 | -2.9 | 20.8 |
| **$V_{S2}$** | -4923.629605 | -4922.569423 | -4922.660809 | 7.1 | 12.4 | 21.6 |
| **$TSII_{S2}$** | -4923.628109 | -4922.567982 | -4922.656934 | 8.0 | 13.3 | 24.0 |
| **$VI_{S2}$** | -4923.642711 | -4922.579463 | -4922.670268 | -1.2 | 6.1 | 15.6 |
| **$VII_{S2}$** | -5152.74026 | -5151.609546 | -5151.705656 | -27.2 | -16.7 | 6.4 |
| **$TSIII_{S2}$** | -5152.706214 | -5151.581461 | -5151.677208 | -5.8 | 0.9 | 24.3 |
| **$VIII_{S2}$** | -5152.797434 | -5151.667395 | -5151.765762 | -63.0 | -53.0 | -31.3 |
| the second step as the $H_2$ splitting | | | | | | |
| **$IX_{S2}$** | -4924.836817 | -4923.754403 | -4923.844465 | -17.6 | -4.5 | 12.7 |
| **$TSV_{S2}$** | -4924.828402 | -4923.749646 | -4923.841781 | -12.3 | -1.5 | 14.4 |
| **$X_{S2}$** | -4924.834676 | -4923.753899 | -4923.845382 | -16.2 | -4.2 | 12.1 |
| **$TSVI_{S2}$** | -4924.817377 | -4923.738884 | -4923.831062 | -5.4 | 5.2 | 21.1 |
| **$XI_{S2}$** | -4924.840318 | -4923.756624 | -4923.850435 | -19.8 | -5.9 | 8.9 |
| **S3 isomer** | | | | | | |
| **$IV_{S3}$** | -5152.713277 | -5151.586546 | -5151.683473 | -10.2 | -2.1 | 21.6 |
| **$V_{S3}$** | -4923.63086 | -4922.57056 | -4922.661549 | 6.3 | 11.7 | 21.1 |
| **$TSII_{S3}$** | -4923.630155 | -4922.570663 | -4922.661768 | 6.7 | 11.6 | 21.0 |
| **$VI_{S3}$** | -4923.665269 | -4922.601476 | -4922.69209 | -15.3 | -7.7 | 1.9 |
| **$VII_{S3}$** | -5152.747765 | -5151.616745 | -5151.711467 | -31.9 | -21.2 | 2.8 |
| **$TSIII_{S3}$** | -5152.700612 | -5151.575417 | -5151.671329 | -2.3 | 4.7 | 27.9 |
| **$VIII_{S3}$** | -5152.771223 | -5151.641609 | -5151.738986 | -46.6 | -36.8 | -14.5 |
| the second step as the $H_2$ splitting | | | | | | |

| | | | | | | |
|---|---|---|---|---|---|---|
| **TSIV**$_{S3}$ | -4924.779819 | -4923.699488 | -4923.789807 | 18.2 | 29.9 | 47.0 |

**Table S2.** The absolute (in Hartree) and relative (in kcal/mol) energies for R isomer in gas phase by M06L/6-31G* method.

| | **E** | **E+ZPE** | **G** | **ΔE**$_{gas}$ | **ΔE**$_{gas+ZPE}$ | **ΔG**$_{gas}$ |
|---|---|---|---|---|---|---|
| **R1 isomer** | | | | | | |
| **IV**$_{R1}$ | -5152.712189 | -5151.586983 | -5151.684316 | -9.5 | -2.5 | 19.8 |
| **V**$_{R1}$ | -4923.643563 | -4922.583527 | -4922.67531 | -1.7 | 3.6 | 12.5 |
| **TSII**$_{R1}$ | -4923.626688 | -4922.566498 | -4922.655212 | 8.9 | 14.2 | 25.1 |
| **VI**$_{R1}$ | -4923.661337 | -4922.596433 | -4922.687133 | -12.8 | -4.5 | 5.0 |
| **VII**$_{R1}$ | -5152.737764 | -5151.609726 | -5151.707086 | -25.6 | -16.8 | 5.5 |
| **TSIII**$_{R1}$ | -5152.736818 | -5151.611313 | -5151.707482 | -25.0 | -17.8 | 5.3 |
| **VIII**$_{R1}$ | -5152.7828 | -5151.653866 | -5151.752251 | -53.8 | -44.5 | -22.8 |
| the second step as the $H_2$ splitting | | | | | | |
| **TSIV**$_{R1}$ | -4924.804037 | -4923.72435 | -4923.814598 | 3.0 | 14.3 | 31.4 |
| **R2 isomer** | | | | | | |
| **IV**$_{R2}$ | -5152.736332 | -5151.611563 | -5151.708054 | -24.7 | -18.0 | 4.9 |
| **V**$_{R2}$ | -4923.649559 | -4922.588797 | -4922.679268 | -5.5 | 0.2 | 10.0 |
| **TSII**$_{R2}$ | -4923.649149 | -4922.58939 | -4922.678632 | -5.2 | -0.1 | 10.4 |
| **VI**$_{R2}$ | -4923.689092 | -4922.624351 | -4922.715696 | -30.3 | -22.1 | -12.9 |
| **VII**$_{R2}$ | -5152.731704 | -5151.599999 | -5151.693859 | -21.8 | -10.7 | 13.8 |
| **TSIII**$_{R2}$ | -5152.70137 | -5151.575156 | -5151.6677 | -2.1 | 3.3 | 26.6 |
| **VIII**$_{R2}$ | -5152.789277 | -5151.659551 | -5151.760284 | -57.9 | -48.1 | -27.9 |
| the second step as the $H_2$ splitting | | | | | | |
| **IX**$_{R2}$ | -4924.834189 | -4923.751484 | -4923.842223 | -15.9 | -2.7 | 14.1 |
| **TSV**$_{R2}$ | -4924.828498 | -4923.749549 | -4923.840072 | -12.4 | -1.5 | 15.4 |
| **X**$_{R2}$ | -4924.834929 | -4923.754273 | -4923.844887 | -16.4 | -4.4 | 12.4 |
| **TSVI**$_{R2}$ | -4924.816815 | -4923.737779 | -4923.828779 | -5.0 | 5.9 | 22.5 |
| **XI**$_{R2}$ | -4924.844795 | -4923.761682 | -4923.856024 | -22.6 | -9.1 | 5.4 |
| **R3 isomer** | | | | | | |
| **IV**$_{R3}$ | -5152.709277 | -5151.58168 | -5151.676831 | -7.7 | 0.8 | 24.5 |
| **V**$_{R3}$ | -4923.633023 | -4922.571734 | -4922.66116 | 4.9 | 11.0 | 21.3 |
| **TSII**$_{R3}$ | -4923.627854 | -4922.567266 | -4922.657002 | 8.2 | 13.8 | 23.9 |
| **VI**$_{R3}$ | -4923.637926 | -4922.574043 | -4922.6646 | 1.8 | 9.5 | 19.2 |
| **VII**$_{R3}$ | -5152.734004 | -5151.603138 | -5151.697629 | -23.2 | -12.7 | 11.4 |
| **TSIII**$_{R3}$ | -5152.693515 | -5151.567422 | -5151.661795 | 2.2 | 9.7 | 33.9 |
| **VIII**$_{R3}$ | -5152.780877 | -5151.650022 | -5151.748343 | -52.6 | -42.1 | -20.4 |
| the second step as the $H_2$ splitting | | | | | | |
| **TSIV**$_{R3}$ | -4924.774419 | -4923.695431 | -4923.785588 | 21.6 | 32.5 | 49.6 |

**Table S3.** The absolute (in Hartree) and relative (in kcal/mol) single-point energies for the S- and R isomers in gas phase and in $CF_3CH_2OH$ as solution with SMD model by B3LYP/6-31G* method.

| | $E_{gas}$ | $E_{SMD}$ | $\Delta E_{gas}$ | $\Delta E_{SMD}$ | $\Delta G_{gas}$ | $\Delta G_{SMD}$ |
|---|---|---|---|---|---|---|
| **$H_2$** | -1.175481 | -1.174926 | | | | |
| **Sub2** | -722.17706 | -722.193087 | | | | |
| **AcOH** | -229.08468 | -229.090818 | | | | |
| **I** | -4429.963459 | -4430.045596 | 0.0 | 0.0 | 0.0 | 0.0 |
| **TSI** | -4431.113795 | -4431.197205 | 15.8 | 14.6 | 25.3 | 24.1 |
| **III** | -4431.124908 | -4431.20592 | 8.8 | 9.2 | 20.1 | 20.5 |
| **S isomer** | | | | | | |
| **$TSII_{S2}$** | -4924.209696 | -4924.307447 | 13.6 | 9.6 | 29.6 | 25.6 |
| **$VI_{S2}$** | -4924.225869 | -4924.327816 | 3.4 | -3.2 | 20.2 | 13.6 |
| **$VII_{S2}$** | -5153.355856 | | -25.0 | | 8.6 | |
| **$TSIII_{S2}$** | -5153.32496 | -5153.415593 | -5.6 | -1.2 | 24.5 | 28.8 |
| **$TSII_{S3}$** | -4924.212606 | | 11.7 | | 26.0 | |
| **$TSIII_{S3}$** | -5153.323868 | | -4.9 | | 25.3 | |
| **$V_{S1}$** | -4924.224794 | -4924.312552 | 4.1 | 6.4 | 19.7 | 22.1 |
| **$TSII_{S1}$** | -4924.212285 | -4924.305562 | 11.9 | 10.8 | 25.8 | 24.7 |
| **$VI_{S1}$** | -4924.232134 | -4924.332039 | -0.5 | -5.8 | 14.7 | 9.4 |
| **$VII_{S1}$** | -5153.366738 | -5153.46545 | -31.8 | -32.5 | -0.5 | -1.3 |
| **$TSIII_{S1}$** | -5153.365022 | -5153.460532 | -30.8 | -29.4 | 0.3 | 1.6 |
| **$VIII_{S1}$** | -5153.401124 | -5153.497112 | -53.4 | -52.4 | -20.0 | -18.9 |
| the second step as the $H_2$ splitting | | | | | | |
| **$TSV_{S2}$** | -4925.416275 | -4925.506765 | -5.9 | -5.7 | 20.8 | 21.0 |
| **$X_{S2}$** | -4925.422182 | -4925.513019 | -9.7 | -9.6 | 18.7 | 18.7 |
| **$TSVI_{S2}$** | -4925.407793 | -4925.498404 | -0.6 | -0.4 | 25.9 | 26.0 |
| **$TSIV_{S3}$** | -4925.372863 | | 21.3 | | 50.1 | |
| **$TSIV_{S1}$** | -4925.388136 | -4925.490619 | 11.7 | 4.5 | 37.5 | 30.2 |
| **R isomer** | | | | | | |
| **$TSII_{R2}$** | -4924.227475 | | 2.4 | | 18.0 | |
| **$VI_{R2}$** | -4924.278604 | | -29.7 | | -11.2 | |
| **$VII_{R2}$** | -5153.344705 | | -18.0 | | 17.6 | |
| **$TSIII_{R2}$** | -5153.314029 | -5153.410567 | 1.2 | 1.9 | 30.0 | 30.6 |
| **$TSII_{R3}$** | -4924.213814 | | 11.0 | | 26.8 | |
| **$TSIII_{R3}$** | -5153.31257 | | 2.1 | | 33.9 | |
| **$V_{R1}$** | -4924.228664 | -4924.316291 | 1.7 | 4.1 | 15.8 | 18.2 |
| **$TSII_{R1}$** | -4924.209907 | -4924.302834 | 13.4 | 12.5 | 29.6 | 28.7 |
| **$VI_{R1}$** | -4924.254676 | -4924.302578 | -14.7 | 12.7 | 3.2 | 27.3 |
| **$VII_{R1}$** | -5153.364893 | -4924.355739 | -30.7 | -20.7 | 0.4 | -2.8 |
| **$TSIII_{R1}$** | -5153.362527 | -5153.466083 | -29.2 | -32.9 | 1.1 | -1.8 |
| **$VIII_{R1}$** | -5153.398934 | -5153.460171 | -52.0 | -29.2 | -21.0 | 1.0 |
| the second step as the $H_2$ splitting | | | | | | |

| | | | | | | |
|---|---|---|---|---|---|---|
| **TSV$_{R2}$** | -4925.417482 | -4925.505922 | -6.7 | -5.1 | 21.1 | 22.7 |
| **TSVI$_{R2}$** | -4925.408586 | -4925.497282 | -1.1 | 0.3 | 26.4 | 27.8 |
| **TSIV$_{R3}$** | -4925.360961 | | 28.8 | | 56.9 | |
| **TSIV$_{R1}$** | -4925.395769 | -4925.492032 | 6.9 | 3.6 | 35.4 | 32.0 |

**Table S4.** The absolute (in Hartree) and relative (in kcal/mol) single-point energies for the S- and R isomers in $CF_3CH_2OH$ as solution with SMD model by M06L/6-31G* method.

| | **E$_{SMD}$** | **ΔE$_{SMD}$** | **ΔG$_{SMD}$** |
|---|---|---|---|
| **H$_2$** | -1.167359 | | |
| **Sub** | -722.099159 | | |
| **AcOH** | -229.06254 | | |
| **I** | -4429.526063 | 0.0 | 0.0 |
| **II** | -4430.697295 | -2.4 | 5.8 |
| **TSII** | -4430.671398 | 13.8 | 23.4 |
| **III** | -4430.679782 | 8.6 | 19.9 |
| **S1 isomer** | | | |
| **IV$_{S1}$** | -5152.805026 | -7.8 | 21.3 |
| **V$_{S1}$** | -4923.724541 | 3.5 | 19.1 |
| **TSII$_{S1}$** | -4923.719647 | 6.5 | 20.4 |
| **VI$_{S1}$** | -4923.741717 | -7.3 | 7.9 |
| **VII$_{S1}$** | -5152.836036 | -27.3 | 4.0 |
| **TSIII$_{S1}$** | -5152.832691 | -25.2 | 5.9 |
| **VIII$_{S1}$** | -5152.87809 | -53.7 | -20.2 |
| the second step as the $H_2$ splitting | | | |
| **TSIV$_{S1}$** | -4924.89379 | 2.3 | 28.1 |
| **S2 isomer** | | | |
| **IV$_{S2}$** | -5152.810001 | -10.9 | 21.3 |
| **V$_{S2}$** | -4923.722199 | 4.9 | 19.5 |
| **TSII$_{S2}$** | -4923.723261 | 4.3 | 20.3 |
| **VI$_{S2}$** | -4923.741585 | -7.2 | 9.6 |
| **VII$_{S2}$** | -5152.828906 | -22.8 | 10.8 |
| **TSIII$_{S2}$** | -5152.794223 | -1.0 | 29.1 |
| **VIII$_{S2}$** | -5152.88546 | -58.3 | -24.1 |
| the second step as the $H_2$ splitting | | | |
| **IX$_{S2}$** | -4924.924895 | -17.3 | 13.0 |
| **TSV$_{S2}$** | -4924.916361 | -11.9 | 14.8 |
| **X$_{S2}$** | -4924.922993 | -16.1 | 12.3 |
| **TSVI$_{S2}$** | -4924.905759 | -5.2 | 21.3 |
| **XI$_{S2}$** | -4924.930417 | -20.7 | 8.0 |
| **R1 isomer** | | | |

| | | | |
|---|---|---|---|
| **IV$_{R1}$** | -5152.803219 | -6.7 | 22.7 |
| **V$_{R1}$** | -4923.729393 | 0.4 | 14.6 |
| **TSII$_{R1}$** | -4923.716948 | 8.2 | 24.4 |
| **VI$_{R1}$** | -4923.757888 | -17.5 | 0.4 |
| **VII$_{R1}$** | -5152.835193 | -26.7 | 4.4 |
| **TSIII$_{R1}$** | -5152.831081 | -24.2 | 6.1 |
| **VIII$_{R1}$** | -5152.876807 | -52.9 | -21.8 |
| the second step as the $H_2$ splitting | | | |
| **TSIV$_{R1}$** | -4924.896708 | 0.4 | 28.9 |
| **R2 isomer** | | | |
| **IV$_{R2}$** | -5152.826749 | -21.4 | 8.2 |
| **V$_{R2}$** | -4923.735822 | -3.6 | 11.8 |
| **TSII$_{R2}$** | -4923.735302 | -3.3 | 12.3 |
| **VI$_{R2}$** | -4923.775239 | -28.4 | -10.9 |
| **VII$_{R2}$** | -5152.821959 | -18.4 | 17.2 |
| **TSIII$_{R2}$** | -5152.794575 | -1.3 | 27.5 |
| **VIII$_{R2}$** | -5152.880789 | -55.4 | -25.3 |
| the second step as the $H_2$ splitting | | | |
| **IX$_{R2}$** | -4924.922067 | -15.5 | 14.5 |
| **TSV$_{R2}$** | -4924.914832 | -10.9 | 16.9 |
| **X$_{R2}$** | -4924.920749 | -14.7 | 14.2 |
| **TSVI$_{R2}$** | -4924.903839 | -4.0 | 23.5 |
| **XI$_{R2}$** | -4924.934486 | -23.3 | 4.7 |

# Cartesian coordinates of all optimized structures

**$H_2$**

| | | | |
|---|---|---|---|
| H | 0.000000 | 0.000000 | 0.372098 |
| H | 0.000000 | 0.000000 | -0.372098 |

**Sub**

| | | | |
|---|---|---|---|
| C | 3.497754 | 0.872800 | 0.867963 |
| C | 2.110785 | 0.922997 | 0.796686 |
| C | 1.410801 | 0.067204 | -0.063633 |
| C | 2.138176 | -0.836178 | -0.851360 |
| C | 3.525939 | -0.878773 | -0.783265 |
| C | 4.210477 | -0.027044 | 0.079298 |
| H | 4.023722 | 1.535601 | 1.552203 |
| H | 1.555471 | 1.607241 | 1.435836 |
| H | 1.621831 | -1.480927 | -1.563597 |
| H | 4.074446 | -1.575787 | -1.413818 |
| H | 5.296187 | -0.064757 | 0.137162 |
| C | -0.063688 | 0.116692 | -0.147536 |
| C | -0.680508 | 1.321662 | -0.131640 |
| H | -0.119253 | 2.233772 | 0.027500 |
| N | -2.063029 | 1.584229 | -0.402765 |
| N | -0.712051 | -1.106913 | -0.230038 |
| H | -0.117685 | -1.890148 | -0.472030 |
| O | -2.725149 | 0.752886 | -1.027528 |
| O | -2.475690 | 2.691653 | -0.044910 |
| C | -1.870524 | -1.443346 | 0.496685 |
| O | -2.428012 | -0.664574 | 1.235972 |
| C | -2.347082 | -2.844204 | 0.230404 |
| H | -1.536309 | -3.556840 | 0.041491 |
| H | -2.946927 | -3.189515 | 1.074314 |
| H | -2.987893 | -2.830433 | -0.659322 |

**AcOH**

| | | | |
|---|---|---|---|
| C | -1.387709 | -0.098415 | -0.000010 |
| H | -1.910216 | 0.858955 | -0.000688 |
| H | -1.683160 | -0.681705 | -0.878229 |
| H | -1.683258 | -0.680407 | 0.879044 |
| C | 0.092058 | 0.126793 | -0.000020 |
| O | 0.655543 | 1.197154 | 0.000003 |
| O | 0.762429 | -1.053419 | -0.000002 |
| H | 1.706765 | -0.816997 | 0.000040 |

**I**

| | | | |
|---|---|---|---|
| P | -0.413424 | 1.517202 | -1.462356 |
| P | 0.419508 | 1.513195 | 1.466394 |
| C | 0.805150 | -1.304261 | 1.340776 |
| C | 2.990568 | -0.364420 | 0.477888 |
| C | 4.300319 | -2.590410 | 1.830292 |
| H | 4.896315 | -1.800451 | 1.380179 |
| C | 3.049029 | 0.950446 | 0.928356 |
| C | -2.992692 | -0.349473 | -0.477525 |
| C | -2.197762 | -1.376225 | -1.211980 |
| C | -0.595767 | 1.746934 | 3.010627 |
| C | 3.755064 | -0.736872 | -0.676478 |
| C | -0.811684 | -1.298314 | -1.341628 |
| C | -2.896965 | -2.474363 | -1.820135 |
| C | -0.100865 | -2.338355 | -1.987851 |
| H | 0.986088 | -2.265088 | -2.044692 |
| C | -0.000306 | -0.157772 | 0.775726 |
| H | -1.046489 | -0.295168 | 1.089078 |
| C | -3.045310 | 0.966579 | -0.925130 |
| C | -2.151094 | 1.424526 | -2.034769 |
| H | -2.436710 | 2.420087 | -2.394471 |
| H | -2.177335 | 0.739483 | -2.895779 |
| C | 2.137336 | -3.529608 | 2.461268 |
| C | 2.190782 | -1.389047 | 1.210053 |
| C | 4.654535 | 0.211351 | -1.264430 |
| C | -4.653451 | 0.229900 | 1.266589 |
| C | -0.000903 | -0.156581 | -0.774521 |
| H | 1.044608 | -0.298276 | -1.088195 |
| C | 2.819622 | -4.623121 | 3.046517 |
| H | 2.231063 | -5.404671 | 3.525746 |
| C | -2.842295 | -4.602802 | -3.056061 |
| H | -2.257595 | -5.386337 | -3.536753 |
| C | 2.156753 | 1.410297 | 2.038705 |
| H | 2.447669 | 2.403176 | 2.401485 |
| H | 2.178768 | 0.722475 | 2.897620 |
| C | -2.154646 | -3.514264 | -2.467861 |
| C | 5.391144 | -0.153827 | -2.416691 |
| H | 6.075282 | 0.576217 | -2.848173 |
| C | -3.642816 | -1.997628 | 1.284479 |
| H | -2.974523 | -2.734996 | 0.843158 |
| C | -4.358608 | -2.312661 | 2.417039 |
| H | -4.244769 | -3.295012 | 2.871090 |
| C | 4.757194 | 1.505323 | -0.701494 |
| H | 5.459414 | 2.215932 | -1.135718 |
| C | 2.884705 | -2.492155 | 1.815410 |

| | | | |
|---|---|---|---|
| C | -0.745715 | -3.423336 | -2.518948 |
| H | -0.182206 | -4.221920 | -2.999753 |
| C | 0.728948 | -3.431402 | 2.513621 |
| H | 0.161622 | -4.228080 | 2.993110 |
| C | -3.758484 | -0.721071 | 0.676198 |
| C | 4.348603 | -2.327119 | -2.420844 |
| H | 4.230505 | -3.307915 | -2.877156 |
| C | 0.089388 | -2.341973 | 1.985276 |
| H | -0.997130 | -2.263302 | 2.043016 |
| C | -5.391306 | -0.134627 | 2.418273 |
| H | -6.071999 | 0.597495 | 2.851689 |
| C | -4.750223 | 1.525659 | 0.706664 |
| H | -5.448839 | 2.238616 | 1.142841 |
| C | 0.053073 | 2.847236 | 3.852615 |
| H | -0.610256 | 3.091775 | 4.691940 |
| H | 0.217424 | 3.767127 | 3.280018 |
| H | 1.009563 | 2.527347 | 4.282422 |
| C | -4.951865 | -3.629679 | -2.427710 |
| H | -6.039191 | -3.669175 | -2.430590 |
| C | 3.633808 | -2.011449 | -1.287813 |
| H | 2.962052 | -2.746846 | -0.848504 |
| C | -4.313050 | -2.565149 | -1.836024 |
| H | -4.905049 | -1.773134 | -1.384276 |
| C | -3.935957 | 1.893774 | -0.333739 |
| H | -3.978135 | 2.907077 | -0.734043 |
| C | 5.240071 | -1.394059 | -2.989703 |
| H | 5.806137 | -1.658715 | -3.880092 |
| C | 4.933870 | -3.659677 | 2.419057 |
| H | 6.020972 | -3.704883 | 2.421188 |
| C | -1.990758 | 2.167735 | 2.561531 |
| H | -2.410588 | 1.442621 | 1.855803 |
| H | -1.983902 | 3.151234 | 2.070869 |
| H | -2.671128 | 2.221599 | 3.422110 |
| C | -0.040013 | 2.854166 | -3.846057 |
| H | 0.625122 | 3.097534 | -4.684294 |
| H | -0.201194 | 3.773558 | -3.271762 |
| H | -0.997463 | 2.539202 | -4.277362 |
| C | -5.245616 | -1.376847 | 2.988373 |
| H | -5.812531 | -1.640976 | 3.878377 |
| C | -4.213868 | -4.665869 | -3.034668 |
| H | -4.732252 | -5.504407 | -3.494584 |
| C | 2.000163 | 2.162210 | -2.555571 |
| H | 2.680999 | 2.215019 | -3.415844 |
| H | 2.416377 | 1.433514 | -1.851434 |
| H | 1.997837 | 3.144488 | -2.062333 |
| C | 4.190829 | -4.693406 | 3.024046 |
| H | 4.705107 | -5.535753 | 3.481639 |
| C | 0.603247 | 1.749222 | -3.005844 |
| C | 3.944286 | 1.874750 | 0.339487 |
| H | 3.991417 | 2.886716 | 0.742605 |
| C | -0.692526 | 0.450624 | 3.816609 |
| H | 0.284439 | 0.005697 | 4.044230 |
| H | -1.301006 | -0.307374 | 3.307425 |
| H | -1.184235 | 0.667221 | 4.773920 |
| C | 0.693948 | 0.454060 | -3.814413 |
| H | -0.285165 | 0.014500 | -4.043202 |
| H | 1.298500 | -0.308010 | -3.306667 |
| H | 1.186962 | 0.670208 | -4.771156 |
| Ni | 0.003772 | 3.013232 | 0.003711 |
| O | -0.361143 | 4.604452 | -1.021116 |
| C | 0.007399 | 5.279604 | 0.002980 |
| O | 0.364545 | 4.604695 | 1.031083 |
| C | 0.045469 | 6.761046 | -0.011989 |
| H | 1.030832 | 7.091538 | -0.361579 |
| H | -0.701769 | 7.160304 | -0.701580 |
| H | -0.102288 | 7.163384 | 0.992750 |

**II**

| | | | |
|---|---|---|---|
| P | -0.639489 | -1.392863 | 1.494044 |
| P | 0.245775 | -1.595668 | -1.407709 |
| C | 1.042175 | 1.135205 | -1.348296 |
| C | 3.051030 | -0.082108 | -0.405444 |
| C | 4.697967 | 1.823708 | -1.859323 |
| H | 5.157553 | 0.975763 | -1.356790 |
| C | 2.938416 | -1.414446 | -0.789890 |
| C | -2.874038 | 0.834387 | 0.425629 |
| C | -1.948766 | 1.730624 | 1.175539 |
| C | -0.699695 | -1.671473 | -3.016265 |
| C | 3.838344 | 0.245146 | 0.747086 |
| C | -0.593120 | 1.442487 | 1.326805 |
| C | -2.488146 | 2.915164 | 1.781704 |
| C | 0.252805 | 2.361112 | 1.995254 |
| H | 1.315812 | 2.128009 | 2.070818 |
| C | 0.069545 | 0.139415 | -0.763527 |
| H | -0.941050 | 0.413825 | -1.103262 |
| C | -3.130849 | -0.454898 | 0.877770 |
| C | -2.352208 | -1.022856 | 2.024413 |
| H | -2.796524 | -1.958650 | 2.383184 |

| | | | |
|---|---|---|---|
| H | -2.300158 | -0.326156 | 2.874432 |
| C | 2.713708 | 3.059323 | -2.567519 |
| C | 2.422009 | 1.008100 | -1.205939 |
| C | 4.594211 | -0.780454 | 1.402671 |
| C | -4.543206 | 0.486818 | -1.370730 |
| C | 0.041819 | 0.181099 | 0.786399 |
| H | 1.090204 | 0.165264 | 1.119778 |
| C | 3.564030 | 3.993196 | -3.206478 |
| H | 3.109423 | 4.831107 | -3.733920 |
| C | -2.144595 | 4.993492 | 3.054634 |
| H | -1.460720 | 5.675462 | 3.558850 |
| C | 2.004579 | -1.808108 | -1.891196 |
| H | 2.140070 | -2.858984 | -2.174902 |
| H | 2.161043 | -1.198260 | -2.794532 |
| C | -1.613042 | 3.824109 | 2.460125 |
| C | 5.345152 | -0.456804 | 2.558333 |
| H | 5.916640 | -1.247176 | 3.044030 |
| C | -3.234615 | 2.554869 | -1.350223 |
| H | -2.482850 | 3.191193 | -0.885738 |
| C | -3.865402 | 2.963942 | -2.502727 |
| H | -3.604522 | 3.921271 | -2.949756 |
| C | 4.547604 | -2.099618 | 0.893325 |
| H | 5.153169 | -2.871394 | 1.367216 |
| C | 3.284985 | 1.953432 | -1.858197 |
| C | -0.234424 | 3.523443 | 2.531820 |
| H | 0.433773 | 4.222986 | 3.032702 |
| C | 1.307437 | 3.188802 | -2.620303 |
| H | 0.874201 | 4.042543 | -3.140041 |
| C | -3.544747 | 1.307155 | -0.749779 |
| C | 4.599568 | 1.831713 | 2.427618 |
| H | 4.604464 | 2.842274 | 2.831594 |
| C | 0.501194 | 2.245662 | -2.041110 |
| H | -0.584017 | 2.341843 | -2.100290 |
| C | -5.185248 | 0.947520 | -2.545472 |
| H | -5.943659 | 0.313944 | -3.004471 |
| C | -4.830034 | -0.785184 | -0.820658 |
| H | -5.599252 | -1.400150 | -1.286518 |
| C | -0.224324 | -2.896067 | -3.801591 |
| H | -0.865969 | -3.021471 | -4.682832 |
| H | -0.284602 | -3.818671 | -3.214309 |
| H | 0.802512 | -2.777942 | -4.167765 |
| C | -4.361805 | 4.353450 | 2.367545 |
| H | -5.430871 | 4.555149 | 2.349497 |
| C | 3.875128 | 1.555138 | 1.290764 |
| H | 3.317856 | 2.348636 | 0.795909 |
| C | -3.875088 | 3.215161 | 1.769298 |
| H | -4.567049 | 2.526272 | 1.290428 |
| C | -4.113681 | -1.256424 | 0.249496 |
| H | -4.308350 | -2.252657 | 0.647958 |
| C | 5.344425 | 0.819516 | 3.067778 |
| H | 5.918915 | 1.053221 | 3.961456 |
| C | 5.495941 | 2.741774 | -2.500563 |
| H | 6.576410 | 2.613836 | -2.491677 |
| C | -2.175046 | -1.802215 | -2.656318 |
| H | -2.492402 | -0.979127 | -2.006650 |
| H | -2.386094 | -2.746439 | -2.137426 |
| H | -2.795761 | -1.762696 | -3.561558 |
| C | -0.489299 | -2.774409 | 3.874877 |
| H | 0.116570 | -3.097380 | 4.730777 |
| H | -0.752315 | -3.666936 | 3.295525 |
| H | -1.411766 | -2.341092 | 4.279067 |
| C | -4.853098 | 2.157584 | -3.105248 |
| H | -5.348954 | 2.495764 | -4.012538 |
| C | -3.491311 | 5.258950 | 3.008143 |
| H | -3.889733 | 6.159314 | 3.471027 |
| C | 1.647666 | -2.352142 | 2.625113 |
| H | 2.290039 | -2.522913 | 3.499224 |
| H | 2.182881 | -1.671834 | 1.953650 |
| H | 1.520559 | -3.311334 | 2.103813 |
| C | 4.928768 | 3.843205 | -3.173239 |
| H | 5.572666 | 4.565250 | -3.670863 |
| C | 0.309945 | -1.761635 | 3.053101 |
| C | 3.712945 | -2.411235 | -0.149366 |
| H | 3.655143 | -3.434679 | -0.517523 |
| C | -0.491771 | -0.404027 | -3.845172 |
| H | 0.565108 | -0.154155 | -4.003800 |
| H | -0.981086 | 0.467185 | -3.393321 |
| H | -0.945393 | -0.551960 | -4.833930 |
| C | 0.551683 | -0.487401 | 3.864074 |
| H | -0.367348 | 0.073886 | 4.075284 |
| H | 1.255368 | 0.190286 | 3.364251 |
| H | 0.997974 | -0.761552 | 4.828891 |
| Ni | -0.580241 | -2.943870 | 0.032114 |
| O | -1.442383 | -4.336974 | 1.044765 |
| C | -1.321410 | -5.089083 | 0.014099 |
| O | -0.775151 | -4.561741 | -1.014866 |
| C | -1.756554 | -6.505799 | 0.028304 |
| H | -0.903090 | -7.137551 | 0.303292 |

| | | | |
|---|---|---|---|
| H | -2.543771 | -6.664520 | 0.768526 |
| H | -2.090566 | -6.818435 | -0.963976 |
| H | 1.836495 | -5.444770 | 0.099843 |
| H | 1.920433 | -4.779235 | 0.431087 |

**TSI**

| | | | |
|---|---|---|---|
| P | -0.847207 | 1.355167 | -1.343879 |
| P | -0.026291 | 1.452110 | 1.608617 |
| C | 1.376634 | -1.005477 | 1.261696 |
| C | 3.033749 | 0.750167 | 0.507726 |
| C | 5.110264 | -0.928528 | 1.669557 |
| H | 5.358185 | 0.059396 | 1.288931 |
| C | 2.620280 | 1.950499 | 1.075911 |
| C | -2.558554 | -1.360706 | -0.510831 |
| C | -1.449170 | -2.005037 | -1.273525 |
| C | -1.021050 | 1.137697 | 3.161601 |
| C | 3.843987 | 0.783347 | -0.673947 |
| C | -0.192963 | -1.412116 | -1.409811 |
| C | -1.707251 | -3.256352 | -1.930153 |
| C | 0.830323 | -2.075830 | -2.128189 |
| H | 1.807437 | -1.596124 | -2.200209 |
| C | 0.193320 | -0.199777 | 0.779735 |
| H | -0.721015 | -0.736197 | 1.075265 |
| C | -3.077013 | -0.138434 | -0.928216 |
| C | -2.398386 | 0.648780 | -2.004506 |
| H | -3.021013 | 1.485712 | -2.340612 |
| H | -2.148665 | 0.034319 | -2.881950 |
| C | 3.467547 | -2.651728 | 2.211897 |
| C | 2.689967 | -0.555383 | 1.141144 |
| C | 4.319658 | 2.040373 | -1.169814 |
| C | -4.316006 | -1.448583 | 1.232621 |
| C | 0.161264 | -0.091026 | -0.765397 |
| H | 1.181142 | 0.177256 | -1.081067 |
| C | 4.522292 | -3.454527 | 2.708597 |
| H | 4.279394 | -4.434703 | 3.117548 |
| C | -0.899714 | -5.156535 | -3.271328 |
| H | -0.079651 | -5.643515 | -3.797757 |
| C | 1.636345 | 1.948943 | 2.203623 |
| H | 1.546485 | 2.940959 | 2.660581 |
| H | 1.924032 | 1.239916 | 2.994772 |
| C | -0.650986 | -3.913800 | -2.640275 |
| C | 5.087986 | 2.069110 | -2.358890 |
| H | 5.445323 | 3.031821 | -2.723210 |
| C | -2.586237 | -3.177787 | 1.205747 |
| H | -1.704640 | -3.625091 | 0.748467 |
| C | -3.141876 | -3.749150 | 2.328340 |
| H | -2.689039 | -4.639302 | 2.760833 |
| C | 3.973773 | 3.226996 | -0.480520 |
| H | 4.360378 | 4.179264 | -0.841467 |
| C | 3.759556 | -1.363376 | 1.657902 |
| C | 0.617606 | -3.297153 | -2.711712 |
| H | 1.420859 | -3.801562 | -3.247189 |
| C | 2.125566 | -3.092118 | 2.256643 |
| H | 1.907249 | -4.079690 | 2.661202 |
| C | -3.145271 | -2.009589 | 0.625600 |
| C | 4.897832 | -0.325439 | -2.566150 |
| H | 5.123035 | -1.237917 | -3.114750 |
| C | 1.114198 | -2.284586 | 1.809244 |
| H | 0.078716 | -2.624022 | 1.858609 |
| C | -4.874515 | -2.074442 | 2.372428 |
| H | -5.766558 | -1.636691 | 2.819801 |
| C | -4.860749 | -0.251762 | 0.710566 |
| H | -5.761148 | 0.161266 | 1.163996 |
| C | -0.780218 | 2.281515 | 4.147556 |
| H | -1.468181 | 2.173712 | 4.995079 |
| H | -0.963818 | 3.268480 | 3.705134 |
| H | 0.238036 | 2.270803 | 4.553626 |
| C | -3.199655 | -5.071116 | -2.567667 |
| H | -4.193252 | -5.514704 | -2.558793 |
| C | 4.166402 | -0.390975 | -1.402166 |
| H | 3.822198 | -1.354389 | -1.028827 |
| C | -2.987576 | -3.868736 | -1.934811 |
| H | -3.819242 | -3.374812 | -1.438942 |
| C | -4.230599 | 0.405263 | -0.314850 |
| H | -4.627423 | 1.348820 | -0.691024 |
| C | 5.366368 | 0.913861 | -3.049538 |
| H | 5.949784 | 0.951390 | -3.966878 |
| C | 6.111097 | -1.728418 | 2.168938 |
| H | 7.137558 | -1.367479 | 2.169441 |
| C | -2.490102 | 1.080862 | 2.751762 |
| H | -2.644587 | 0.355799 | 1.945069 |
| H | -2.868522 | 2.053417 | 2.407468 |
| H | -3.111427 | 0.763120 | 3.599990 |
| C | -0.980833 | 2.900018 | -3.632867 |
| H | -0.445643 | 3.463147 | -4.407759 |
| H | -1.539288 | 3.616504 | -3.020099 |
| H | -1.698202 | 2.248149 | -4.145674 |
| C | -4.298297 | -3.196790 | 2.917178 |

| | | | |
|---|---|---|---|
| H | -4.731756 | -3.662081 | 3.799876 |
| C | -2.146274 | -5.730811 | -3.232839 |
| H | -2.326650 | -6.684910 | -3.723435 |
| C | 1.108016 | 3.019456 | -2.241442 |
| H | 1.740195 | 3.422636 | -3.044045 |
| H | 1.759681 | 2.470247 | -1.553914 |
| H | 0.673355 | 3.866375 | -1.690741 |
| C | 5.820769 | -3.007597 | 2.684994 |
| H | 6.623480 | -3.632394 | 3.070824 |
| C | 0.032061 | 2.102625 | -2.809872 |
| C | 3.112262 | 3.183649 | 0.586043 |
| H | 2.803895 | 4.104604 | 1.081245 |
| C | -0.627165 | -0.190833 | 3.810466 |
| H | 0.451422 | -0.283660 | 3.989761 |
| H | -0.948884 | -1.052809 | 3.213276 |
| H | -1.129796 | -0.270648 | 4.783097 |
| C | 0.679874 | 1.021464 | -3.676893 |
| H | -0.018510 | 0.224952 | -3.963349 |
| H | 1.544499 | 0.555483 | -3.186206 |
| H | 1.049928 | 1.483267 | -4.601600 |
| Ni | -1.102436 | 2.733539 | 0.290781 |
| O | -2.339277 | 3.738239 | -0.747505 |
| C | -3.180436 | 4.465607 | -0.102597 |
| O | -3.145441 | 4.621921 | 1.149336 |
| C | -4.225155 | 5.175506 | -0.899650 |
| H | -5.096203 | 5.405626 | -0.283758 |
| H | -3.809493 | 6.122703 | -1.262290 |
| H | -4.513744 | 4.587572 | -1.774574 |
| H | -1.964495 | 4.051121 | 1.441025 |
| H | -1.084675 | 3.758050 | 1.543250 |

**III**

| | | | |
|---|---|---|---|
| P | -0.871082 | -1.337341 | 1.371877 |
| P | -0.061720 | -1.453635 | -1.596132 |
| C | 1.358194 | 1.003217 | -1.259963 |
| C | 3.012847 | -0.757141 | -0.517772 |
| C | 5.087643 | 0.909372 | -1.704621 |
| H | 5.334122 | -0.079264 | -1.324912 |
| C | 2.580242 | -1.955157 | -1.076191 |
| C | -2.560531 | 1.400487 | 0.522528 |
| C | -1.441029 | 2.041822 | 1.272214 |
| C | -1.055374 | -1.122962 | -3.146514 |
| C | 3.834575 | -0.792856 | 0.655878 |
| C | -0.190061 | 1.437474 | 1.405103 |
| C | -1.683884 | 3.301979 | 1.917478 |
| C | 0.845564 | 2.104536 | 2.102652 |
| H | 1.820891 | 1.620173 | 2.167949 |
| C | 0.174512 | 0.202695 | -0.769978 |
| H | -0.738100 | 0.743440 | -1.062811 |
| C | -3.083681 | 0.185904 | 0.954966 |
| C | -2.403774 | -0.586410 | 2.040455 |
| H | -3.042476 | -1.395529 | 2.413734 |
| H | -2.133325 | 0.052491 | 2.893832 |
| C | 3.448496 | 2.640311 | -2.231696 |
| C | 2.671213 | 0.548646 | -1.150983 |
| C | 4.299439 | -2.052677 | 1.154948 |
| C | -4.322775 | 1.481618 | -1.216262 |
| C | 0.145887 | 0.103459 | 0.776838 |
| H | 1.165082 | -0.168521 | 1.092361 |
| C | 4.502074 | 3.436779 | -2.740929 |
| H | 4.259859 | 4.417665 | -3.148714 |
| C | -0.851143 | 5.209323 | 3.232410 |
| H | -0.022864 | 5.695700 | 3.746511 |
| C | 1.595119 | -1.947752 | -2.202378 |
| H | 1.500134 | -2.937807 | -2.661498 |
| H | 1.884551 | -1.235359 | -2.989541 |
| C | -0.616574 | 3.959057 | 2.610722 |
| C | 5.081893 | -2.083480 | 2.334520 |
| H | 5.430984 | -3.048522 | 2.700871 |
| C | -2.585621 | 3.203253 | -1.208470 |
| H | -1.700691 | 3.650145 | -0.757478 |
| C | -3.142257 | 3.768636 | -2.333485 |
| H | -2.686955 | 4.653543 | -2.774046 |
| C | 3.923511 | -3.240010 | 0.482627 |
| H | 4.296896 | -4.195244 | 0.849845 |
| C | 3.739309 | 1.351208 | -1.679214 |
| C | 0.648417 | 3.334289 | 2.674057 |
| H | 1.461510 | 3.839183 | 3.194073 |
| C | 2.108223 | 3.087005 | -2.262725 |
| H | 1.890320 | 4.075564 | -2.665232 |
| C | -3.147656 | 2.041945 | -0.617549 |
| C | 4.922368 | 0.314058 | 2.530148 |
| H | 5.163263 | 1.226856 | 3.071552 |
| C | 1.097925 | 2.283912 | -1.805419 |
| H | 0.063683 | 2.628087 | -1.845345 |
| C | -4.881474 | 2.100448 | -2.359826 |
| H | -5.776910 | 1.663257 | -2.801136 |
| C | -4.872018 | 0.292760 | -0.680590 |

| | | | |
|---|---|---|---|
| H | -5.776761 | -0.118924 | -1.126847 |
| C | -0.814498 | -2.254925 | -4.145267 |
| H | -1.508332 | -2.144171 | -4.987847 |
| H | -0.984672 | -3.245193 | -3.704415 |
| H | 0.201267 | -2.234092 | -4.557188 |
| C | -3.158245 | 5.132758 | 2.551944 |
| H | -4.149075 | 5.582698 | 2.548617 |
| C | 4.178158 | 0.381427 | 1.374498 |
| H | 3.838909 | 1.346597 | 1.001224 |
| C | -2.959946 | 3.923162 | 1.928250 |
| H | -3.799720 | 3.429617 | 1.445607 |
| C | -4.242619 | -0.356328 | 0.350612 |
| H | -4.645104 | -1.292678 | 0.739774 |
| C | 5.382608 | -0.927743 | 3.014825 |
| H | 5.976134 | -0.966943 | 3.925591 |
| C | 6.087387 | 1.703045 | -2.216010 |
| H | 7.111861 | 1.336496 | -2.227113 |
| C | -2.523292 | -1.072444 | -2.737327 |
| H | -2.682737 | -0.346184 | -1.933133 |
| H | -2.883709 | -2.047696 | -2.380647 |
| H | -3.151538 | -0.769339 | -3.585891 |
| C | -0.995249 | -2.846874 | 3.678890 |
| H | -0.461789 | -3.395088 | 4.465807 |
| H | -1.527506 | -3.582303 | 3.063752 |
| H | -1.736085 | -2.208991 | 4.175776 |
| C | -4.302359 | 3.216251 | -2.914945 |
| H | -4.736460 | 3.676317 | -3.800083 |
| C | -2.094248 | 5.791477 | 3.200961 |
| H | -2.263561 | 6.751175 | 3.684527 |
| C | 1.113856 | -2.932454 | 2.319960 |
| H | 1.737214 | -3.323263 | 3.135799 |
| H | 1.769800 | -2.372216 | 1.645570 |
| H | 0.708442 | -3.787850 | 1.759542 |
| C | 5.798362 | 2.982986 | -2.730932 |
| H | 6.600075 | 3.602866 | -3.126772 |
| C | 0.010166 | -2.032681 | 2.863141 |
| C | 3.051992 | -3.191932 | -0.575433 |
| H | 2.720918 | -4.111817 | -1.057856 |
| C | -0.667240 | 0.212065 | -3.785833 |
| H | 0.411150 | 0.309954 | -3.963294 |
| H | -0.992620 | 1.069329 | -3.183741 |
| H | -1.168702 | 0.297257 | -4.758658 |
| C | 0.624982 | -0.935912 | 3.732313 |
| H | -0.092183 | -0.151977 | 4.007425 |
| H | 1.482828 | -0.454553 | 3.245035 |
| H | 0.998183 | -1.382242 | 4.663598 |
| Ni | -1.061160 | -2.746337 | -0.281363 |
| O | -2.235032 | -3.937956 | 0.694213 |
| C | -3.067161 | -4.675425 | 0.126804 |
| O | -3.142458 | -4.787069 | -1.176517 |
| C | -4.038690 | -5.498158 | 0.887206 |
| H | -5.043286 | -5.381152 | 0.470504 |
| H | -3.777136 | -6.557049 | 0.781961 |
| H | -4.030317 | -5.229338 | 1.943731 |
| H | -2.362490 | -4.260736 | -1.536069 |
| H | -0.969935 | -3.626344 | -1.533721 |

**IV$_{S2}$**

| | | | |
|---|---|---|---|
| P | -0.607459 | 0.596351 | 1.410536 |
| P | -0.408871 | 0.036506 | -1.826017 |
| C | 2.217450 | -0.944314 | -1.339914 |
| C | 0.809535 | -2.977923 | -0.884983 |
| C | 2.979454 | -4.550277 | -2.071384 |
| H | 2.054366 | -5.038903 | -1.775234 |
| C | -0.391771 | -2.689673 | -1.526944 |
| C | 2.146834 | 2.576095 | 0.841850 |
| C | 2.737942 | 1.413202 | 1.564576 |
| C | -0.081035 | 1.219970 | -3.255102 |
| C | 0.818430 | -3.914797 | 0.198791 |
| C | 2.189913 | 0.133246 | 1.493561 |
| C | 3.887610 | 1.638681 | 2.398085 |
| C | 2.826007 | -0.943262 | 2.158089 |
| H | 2.387217 | -1.937359 | 2.069080 |
| C | 1.158284 | 0.001552 | -0.813726 |
| H | 1.549397 | 1.018233 | -0.934196 |
| C | 0.855036 | 3.004583 | 1.135601 |
| C | -0.005094 | 2.221056 | 2.077279 |
| H | -0.870994 | 2.807872 | 2.407943 |
| H | 0.571165 | 1.972528 | 2.977584 |
| C | 4.321971 | -2.532259 | -2.364356 |
| C | 2.064585 | -2.330200 | -1.358345 |
| C | -0.395700 | -4.585095 | 0.563061 |
| C | 2.394159 | 4.510305 | -0.690883 |
| C | 0.936288 | -0.193237 | 0.712495 |
| H | 0.728573 | -1.261806 | 0.858958 |
| C | 5.357089 | -3.337454 | -2.897253 |
| H | 6.274892 | -2.850776 | -3.225884 |
| C | 5.656569 | 0.749253 | 3.862293 |

| | | | |
|---|---|---|---|
| H | 6.122564 | -0.109862 | 4.343777 |
| C | -0.444978 | -1.636391 | -2.588728 |
| H | -1.348261 | -1.725075 | -3.202750 |
| H | 0.425880 | -1.711115 | -3.251549 |
| C | 4.514875 | 0.530658 | 3.054461 |
| C | -0.390862 | -5.488476 | 1.653816 |
| H | -1.321821 | -5.992758 | 1.913746 |
| C | 4.198212 | 2.868233 | -0.558936 |
| H | 4.622551 | 1.961314 | -0.132418 |
| C | 4.898631 | 3.563524 | -1.518120 |
| H | 5.869924 | 3.197733 | -1.845613 |
| C | -1.574745 | -4.315152 | -0.168606 |
| H | -2.489493 | -4.853961 | 0.079210 |
| C | 3.107975 | -3.145770 | -1.913601 |
| C | 3.964423 | -0.760409 | 2.897026 |
| H | 4.447017 | -1.604946 | 3.387989 |
| C | 4.447619 | -1.127098 | -2.292111 |
| H | 5.369679 | -0.660166 | -2.637699 |
| C | 2.921889 | 3.306254 | -0.119672 |
| C | 1.953086 | -5.066419 | 2.019234 |
| H | 2.861294 | -5.258310 | 2.587452 |
| C | 3.417125 | -0.361394 | -1.813558 |
| H | 3.512444 | 0.724599 | -1.784539 |
| C | 3.149499 | 5.213001 | -1.660235 |
| H | 2.734113 | 6.130169 | -2.076969 |
| C | 1.122919 | 4.966638 | -0.275573 |
| H | 0.741987 | 5.910494 | -0.665474 |
| C | -1.433782 | 1.537109 | -3.896826 |
| H | -1.281411 | 2.185900 | -4.769711 |
| H | -2.101630 | 2.052593 | -3.195928 |
| H | -1.953571 | 0.635138 | -4.234994 |
| C | 5.524714 | 3.113424 | 3.434491 |
| H | 5.908910 | 4.117934 | 3.600004 |
| C | 1.985150 | -4.191596 | 0.957533 |
| H | 2.917860 | -3.698273 | 0.689902 |
| C | 4.422817 | 2.932089 | 2.631913 |
| H | 3.945908 | 3.797443 | 2.178451 |
| C | 0.375426 | 4.222017 | 0.599970 |
| H | -0.587712 | 4.596750 | 0.941026 |
| C | 0.756161 | -5.723832 | 2.372669 |
| H | 0.745613 | -6.416138 | 3.211784 |
| C | 3.999215 | -5.303194 | -2.604125 |
| H | 3.870752 | -6.377681 | -2.717367 |
| C | 0.559925 | 2.496972 | -2.711237 |
| H | 1.630867 | 2.367118 | -2.509327 |
| H | 0.089268 | 2.850636 | -1.784892 |
| H | 0.482919 | 3.296613 | -3.462014 |
| C | -0.998603 | -1.864833 | 2.635314 |
| H | -1.531138 | -2.425201 | 3.414962 |
| H | 0.021056 | -2.270095 | 2.585524 |
| H | -1.492599 | -2.091162 | 1.681893 |
| C | 4.374592 | 4.748876 | -2.074025 |
| H | 4.942246 | 5.292711 | -2.825924 |
| C | 6.158477 | 2.013940 | 4.047694 |
| H | 7.033521 | 2.171794 | 4.674438 |
| C | -0.015671 | -0.130699 | 4.127005 |
| H | -0.233298 | -0.852980 | 4.924570 |
| H | -0.126434 | 0.865779 | 4.569172 |
| H | 1.031964 | -0.269799 | 3.839348 |
| C | 5.205583 | -4.697587 | -3.011287 |
| H | 6.005972 | -5.306852 | -3.425799 |
| C | -0.999070 | -0.373463 | 2.978191 |
| C | -1.575071 | -3.380960 | -1.174002 |
| H | -2.489642 | -3.184296 | -1.734276 |
| C | 0.840589 | 0.607966 | -4.312964 |
| H | 0.363812 | -0.217409 | -4.852708 |
| H | 1.787954 | 0.246677 | -3.892862 |
| H | 1.085296 | 1.378329 | -5.056552 |
| C | -2.386396 | 0.088738 | 3.430608 |
| H | -3.156468 | -0.126673 | 2.686326 |
| H | -2.401693 | 1.165990 | 3.650525 |
| H | -2.668437 | -0.432069 | 4.355097 |
| Ni | -2.103318 | 0.501501 | -0.320343 |
| H | -1.854274 | -0.895520 | -0.105910 |
| C | -4.506545 | -2.979011 | 1.996072 |
| C | -4.068333 | -2.045119 | 1.071864 |
| C | -4.682734 | -0.789635 | 0.955044 |
| C | -5.774640 | -0.522653 | 1.798002 |
| C | -6.222182 | -1.463889 | 2.720635 |
| C | -5.584618 | -2.693128 | 2.834709 |
| H | -3.992236 | -3.937057 | 2.069901 |
| H | -3.230674 | -2.295056 | 0.427552 |
| H | -6.298110 | 0.427733 | 1.726633 |
| H | -7.076075 | -1.227010 | 3.351569 |
| H | -5.925044 | -3.425322 | 3.563613 |
| C | -4.199111 | 0.282737 | 0.048397 |
| C | -3.752312 | 0.152980 | -1.305176 |
| N | -4.748316 | 1.565828 | 0.320323 |

| | | | |
|---|---|---|---|
| H | -4.583625 | 1.935081 | 1.249918 |
| N | -3.945910 | -1.037201 | -2.128820 |
| O | -4.573179 | -1.996168 | -1.691256 |
| O | -3.462564 | -0.985178 | -3.266109 |
| H | -3.836818 | 1.023909 | -1.951458 |
| C | -5.797678 | 2.212682 | -0.322852 |
| O | -6.150796 | 3.312276 | 0.082391 |
| C | -6.457232 | 1.514292 | -1.479678 |
| H | -6.452830 | 0.423214 | -1.381797 |
| H | -7.486637 | 1.873113 | -1.541155 |
| H | -5.972588 | 1.771570 | -2.429171 |
| C | -2.517005 | 3.622625 | -0.460525 |
| O | -2.182404 | 2.481201 | -0.786884 |
| C | -3.116096 | 4.054648 | 0.826360 |
| H | -4.211669 | 4.094327 | 0.714834 |
| H | -2.869313 | 3.341906 | 1.618444 |
| H | -2.780106 | 5.059196 | 1.095583 |
| O | -2.362279 | 4.626316 | -1.317510 |
| H | -1.973960 | 4.267481 | -2.138159 |

**$V_{S2}$**

| | | | |
|---|---|---|---|
| P | -1.058829 | 0.118270 | 1.144772 |
| P | -0.378340 | -0.443379 | -1.880052 |
| C | 2.383849 | -0.451048 | -1.190176 |
| C | 1.669272 | -2.816398 | -0.665949 |
| C | 4.252129 | -3.664001 | -1.723201 |
| H | 3.491637 | -4.404397 | -1.485796 |
| C | 0.458995 | -3.010768 | -1.320524 |
| C | 0.622739 | 3.011346 | 0.663720 |
| C | 1.598486 | 2.274463 | 1.513087 |
| C | -0.334696 | 0.653300 | -3.395990 |
| C | 1.976415 | -3.613118 | 0.485171 |
| C | 1.692493 | 0.885377 | 1.501497 |
| C | 2.463843 | 3.028763 | 2.377531 |
| C | 2.731657 | 0.245772 | 2.221438 |
| H | 2.822170 | -0.838602 | 2.147566 |
| C | 1.041152 | 0.133744 | -0.811133 |
| H | 1.073132 | 1.211518 | -1.031925 |
| C | -0.746666 | 2.853082 | 0.845862 |
| C | -1.275930 | 1.826886 | 1.801338 |
| H | -2.341221 | 2.010375 | 1.994674 |
| H | -0.754533 | 1.867780 | 2.769538 |
| C | 4.954926 | -1.337820 | -1.963541 |
| C | 2.659185 | -1.816936 | -1.158095 |
| C | 1.088203 | -4.668270 | 0.874696 |
| C | 0.146482 | 4.751394 | -1.031608 |
| C | 0.759757 | 0.000630 | 0.700128 |
| H | 0.991290 | -1.041663 | 0.966519 |
| C | 6.223789 | -1.798919 | -2.387778 |
| H | 6.982141 | -1.061820 | -2.649529 |
| C | 4.327088 | 3.097174 | 3.983517 |
| H | 5.098534 | 2.565391 | 4.539511 |
| C | 0.079964 | -2.121982 | -2.466461 |
| H | -0.770482 | -2.529983 | -3.025348 |
| H | 0.914887 | -2.008419 | -3.172419 |
| C | 3.480504 | 2.354218 | 3.126863 |
| C | 1.389200 | -5.438452 | 2.024404 |
| H | 0.707616 | -6.241095 | 2.305104 |
| C | 2.463275 | 4.050784 | -0.674612 |
| H | 3.196509 | 3.444243 | -0.145296 |
| C | 2.876266 | 4.913690 | -1.663882 |
| H | 3.932711 | 4.976743 | -1.917580 |
| C | -0.092526 | -4.882063 | 0.124852 |
| H | -0.760895 | -5.693683 | 0.412174 |
| C | 3.943032 | -2.284699 | -1.601573 |
| C | 3.609726 | 0.953023 | 2.997437 |
| H | 4.405313 | 0.442147 | 3.538380 |
| C | 4.658252 | 0.042090 | -1.895200 |
| H | 5.435947 | 0.763458 | -2.143049 |
| C | 1.091760 | 3.935336 | -0.327544 |
| C | 3.373371 | -4.125462 | 2.410462 |
| H | 4.253409 | -3.914535 | 3.014533 |
| C | 3.405787 | 0.465950 | -1.540948 |
| H | 3.177557 | 1.532347 | -1.508327 |
| C | 0.609213 | 5.645487 | -2.027383 |
| H | -0.121763 | 6.263065 | -2.548610 |
| C | -1.231045 | 4.618901 | -0.740131 |
| H | -1.946479 | 5.256912 | -1.257828 |
| C | -1.274792 | 0.069474 | -4.450931 |
| H | -1.274088 | 0.723824 | -5.331198 |
| H | -2.315528 | 0.011213 | -4.106132 |
| H | -0.961471 | -0.926113 | -4.788569 |
| C | 3.159210 | 5.121895 | 3.404883 |
| H | 3.027241 | 6.194600 | 3.529844 |
| C | 3.122877 | -3.374227 | 1.285834 |
| H | 3.811249 | -2.580397 | 1.002789 |
| C | 2.330101 | 4.430214 | 2.552899 |
| H | 1.546575 | 4.962157 | 2.018334 |

| | | | |
|---|---|---|---|
| C | -1.665609 | 3.676912 | 0.156282 |
| H | -2.726498 | 3.565785 | 0.370014 |
| C | 2.504140 | -5.171515 | 2.782460 |
| H | 2.718425 | -5.764478 | 3.668855 |
| C | 5.490600 | -4.080202 | -2.153010 |
| H | 5.697914 | -5.144450 | -2.244653 |
| C | -0.787510 | 2.051713 | -2.988227 |
| H | -0.138239 | 2.480543 | -2.216145 |
| H | -1.814285 | 2.081953 | -2.609251 |
| H | -0.735020 | 2.722152 | -3.856185 |
| C | -1.216469 | -2.429880 | 2.179006 |
| H | -1.184912 | -3.117143 | 3.035519 |
| H | -0.339877 | -2.647306 | 1.559020 |
| H | -2.109659 | -2.665966 | 1.589226 |
| C | 1.944015 | 5.724539 | -2.343769 |
| H | 2.285216 | 6.410142 | -3.116523 |
| C | 4.173576 | 4.455198 | 4.121637 |
| H | 4.825398 | 5.016503 | 4.787694 |
| C | -0.019644 | -0.797479 | 3.615702 |
| H | -0.244797 | -1.314723 | 4.557464 |
| H | 0.194768 | 0.250177 | 3.859717 |
| H | 0.897636 | -1.245309 | 3.219519 |
| C | 6.491895 | -3.142889 | -2.478942 |
| H | 7.469190 | -3.486637 | -2.810822 |
| C | -1.202133 | -0.984708 | 2.662798 |
| C | -0.412551 | -4.051662 | -0.919770 |
| H | -1.332664 | -4.207867 | -1.483998 |
| C | 1.085273 | 0.742922 | -3.961555 |
| H | 1.557058 | -0.232209 | -4.134514 |
| H | 1.746443 | 1.327175 | -3.312906 |
| H | 1.045025 | 1.261299 | -4.927780 |
| C | -2.495646 | -0.668819 | 3.412216 |
| H | -3.381963 | -0.924522 | 2.822635 |
| H | -2.559553 | 0.377054 | 3.740210 |
| H | -2.547086 | -1.288086 | 4.316585 |
| Ni | -2.200436 | -0.371853 | -0.732596 |
| H | -2.212560 | -0.727709 | -2.163118 |
| C | -4.128106 | -4.145884 | 0.038352 |
| C | -3.873246 | -2.828241 | -0.315190 |
| C | -4.411959 | -1.765431 | 0.431776 |
| C | -5.212007 | -2.064492 | 1.541822 |
| C | -5.460854 | -3.385892 | 1.896808 |
| C | -4.920504 | -4.429827 | 1.149840 |
| H | -3.708250 | -4.956125 | -0.554944 |
| H | -3.234795 | -2.614264 | -1.175402 |
| H | -5.655312 | -1.250553 | 2.112422 |
| H | -6.091270 | -3.600748 | 2.756749 |
| H | -5.123468 | -5.461976 | 1.426040 |
| C | -4.141821 | -0.358060 | 0.042927 |
| C | -4.076186 | -0.140274 | -1.359752 |
| N | -4.461744 | 0.641905 | 0.972687 |
| H | -3.921479 | 0.644145 | 1.829801 |
| C | -5.216705 | 1.813578 | 0.861023 |
| O | -5.007122 | 2.707713 | 1.661060 |
| C | -6.328596 | 1.842749 | -0.145687 |
| H | -6.453751 | 0.910155 | -0.704678 |
| H | -7.257892 | 2.048667 | 0.394268 |
| H | -6.178375 | 2.667992 | -0.847940 |
| N | -4.131276 | 1.169223 | -1.992814 |
| O | -3.692446 | 2.147663 | -1.374934 |
| O | -4.569926 | 1.204348 | -3.137727 |
| H | -4.473772 | -0.893025 | -2.032886 |

**TSII$_{S2}$**

| | | | |
|---|---|---|---|
| P | -0.997297 | 0.101804 | 1.194361 |
| P | -0.345378 | -0.402096 | -1.842981 |
| C | 2.416599 | -0.432498 | -1.190295 |
| C | 1.689617 | -2.798934 | -0.673989 |
| C | 4.241091 | -3.657151 | -1.793078 |
| H | 3.478104 | -4.393153 | -1.550204 |
| C | 0.466738 | -2.994769 | -1.305421 |
| C | 0.641381 | 3.017498 | 0.688846 |
| C | 1.646064 | 2.297166 | 1.519511 |
| C | -0.299064 | 0.666283 | -3.378462 |
| C | 2.020465 | -3.600219 | 0.467384 |
| C | 1.745284 | 0.906524 | 1.518840 |
| C | 2.522383 | 3.062369 | 2.362316 |
| C | 2.789596 | 0.276488 | 2.239338 |
| H | 2.880990 | -0.808604 | 2.178390 |
| C | 1.081778 | 0.155958 | -0.791679 |
| H | 1.115515 | 1.235554 | -1.003103 |
| C | -0.718475 | 2.822548 | 0.898680 |
| C | -1.185701 | 1.796953 | 1.883206 |
| H | -2.230969 | 1.981103 | 2.158681 |
| H | -0.597955 | 1.830156 | 2.812339 |
| C | 4.962425 | -1.335384 | -2.019524 |
| C | 2.675905 | -1.801253 | -1.180205 |
| C | 1.149529 | -4.667414 | 0.861985 |

| | | | | | | | |
|---|---|---|---|---|---|---|---|
| C | 0.077340 | 4.726378 | -1.010032 | H | -2.736466 | 3.422595 | 0.411616 |
| C | 0.817123 | 0.010319 | 0.724159 | C | 2.597392 | -5.167398 | 2.746384 |
| H | 1.065652 | -1.030004 | 0.984304 | H | 2.830544 | -5.763867 | 3.625592 |
| C | 6.218757 | -1.803790 | -2.472016 | C | 5.467692 | -4.080277 | -2.249588 |
| H | 6.979504 | -1.070981 | -2.738871 | H | 5.662899 | -5.145371 | -2.356610 |
| C | 4.410728 | 3.151906 | 3.938459 | C | -0.751618 | 2.066272 | -2.981285 |
| H | 5.190765 | 2.627285 | 4.489115 | H | -0.091625 | 2.503348 | -2.223699 |
| C | 0.054259 | -2.096434 | -2.431724 | H | -1.770857 | 2.084833 | -2.579241 |
| H | -0.830056 | -2.486407 | -2.950858 | H | -0.721830 | 2.731414 | -3.854339 |
| H | 0.855484 | -1.999405 | -3.179230 | C | -1.200555 | -2.455127 | 2.170264 |
| C | 3.550878 | 2.397821 | 3.104891 | H | -1.173197 | -3.161252 | 3.011424 |
| C | 1.475027 | -5.441928 | 2.001857 | H | -0.334918 | -2.675234 | 1.535517 |
| H | 0.806854 | -6.254399 | 2.286307 | H | -2.105025 | -2.659315 | 1.586947 |
| C | 2.422191 | 4.098470 | -0.694299 | C | 1.819679 | 5.746726 | -2.360446 |
| H | 3.183727 | 3.517532 | -0.176303 | H | 2.124366 | 6.437122 | -3.144120 |
| C | 2.789255 | 4.967205 | -1.696201 | C | 4.258882 | 4.511457 | 4.061570 |
| H | 3.837824 | 5.059385 | -1.972635 | H | 4.920736 | 5.081582 | 4.709935 |
| C | -0.038489 | -4.889877 | 0.126222 | C | 0.043543 | -0.883915 | 3.633308 |
| H | -0.692929 | -5.712363 | 0.414461 | H | -0.181707 | -1.431737 | 4.557616 |
| C | 3.947381 | -2.276543 | -1.651045 | H | 0.270612 | 0.152038 | 3.913302 |
| C | 3.676445 | 0.994865 | 2.995403 | H | 0.953143 | -1.327442 | 3.213238 |
| H | 4.477461 | 0.491193 | 3.535030 | C | 6.471999 | -3.149112 | -2.583386 |
| C | 4.683102 | 0.046788 | -1.926584 | H | 7.439732 | -3.498497 | -2.936531 |
| H | 5.464607 | 0.762642 | -2.178342 | C | -1.148189 | -1.021985 | 2.684908 |
| C | 1.062280 | 3.946595 | -0.317844 | C | -0.385409 | -4.050956 | -0.902729 |
| C | 3.449273 | -4.108601 | 2.370178 | H | -1.314418 | -4.210511 | -1.450789 |
| H | 4.334626 | -3.891255 | 2.964176 | C | 1.115158 | 0.745676 | -3.957748 |
| C | 3.442007 | 0.479107 | -1.543515 | H | 1.584248 | -0.233213 | -4.117037 |
| H | 3.224496 | 1.547239 | -1.492316 | H | 1.780915 | 1.337655 | -3.320797 |
| C | 0.494177 | 5.629469 | -2.017896 | H | 1.070479 | 1.249114 | -4.931703 |
| H | -0.265168 | 6.219583 | -2.530213 | C | -2.431341 | -0.669847 | 3.435896 |
| C | -1.292186 | 4.537105 | -0.709240 | H | -3.318241 | -0.738548 | 2.796899 |
| H | -2.038088 | 5.133610 | -1.233520 | H | -2.393236 | 0.328700 | 3.889096 |
| C | -1.248709 | 0.069693 | -4.418046 | H | -2.579840 | -1.388051 | 4.252336 |
| H | -1.268605 | 0.720719 | -5.300467 | Ni | -2.170442 | -0.292328 | -0.670757 |
| H | -2.285768 | 0.000139 | -4.060610 | H | -2.443088 | -0.393208 | -2.107111 |
| H | -0.932528 | -0.924545 | -4.756328 | C | -4.157769 | -4.127436 | 0.102662 |
| C | 3.232111 | 5.168389 | 3.353299 | C | -3.876413 | -2.824427 | -0.283413 |
| H | 3.101200 | 6.242444 | 3.467172 | C | -4.366131 | -1.730569 | 0.452924 |
| C | 3.175108 | -3.353067 | 1.253765 | C | -5.149796 | -1.986068 | 1.584413 |
| H | 3.850136 | -2.548889 | 0.967129 | C | -5.430679 | -3.292938 | 1.969655 |
| C | 2.389491 | 4.465737 | 2.523968 | C | -4.935996 | -4.367203 | 1.234491 |
| H | 1.597331 | 4.991069 | 1.995864 | H | -3.769920 | -4.960594 | -0.481122 |
| C | -1.680563 | 3.586905 | 0.199753 | H | -3.251061 | -2.650739 | -1.163025 |

| | | | |
|---|---|---|---|
| H | -5.548841 | -1.149023 | 2.154727 |
| H | -6.048640 | -3.471696 | 2.846912 |
| H | -5.161449 | -5.387523 | 1.536043 |
| C | -4.048878 | -0.338752 | 0.034880 |
| C | -3.994756 | -0.160087 | -1.395234 |
| N | -4.427638 | 0.721962 | 0.890629 |
| H | -3.806829 | 0.948615 | 1.656596 |
| C | -5.482746 | 1.630152 | 0.810208 |
| O | -5.487153 | 2.584152 | 1.565493 |
| C | -6.583367 | 1.340282 | -0.170902 |
| H | -6.546392 | 0.339348 | -0.613132 |
| H | -7.536962 | 1.459129 | 0.350661 |
| H | -6.571815 | 2.084409 | -0.974471 |
| N | -4.274257 | 1.144600 | -2.014153 |
| O | -3.777238 | 2.150569 | -1.505203 |
| O | -4.984259 | 1.126005 | -3.012930 |
| H | -4.418368 | -0.946794 | -2.015198 |

**VI$_{S2}$**

| | | | |
|---|---|---|---|
| P | -0.959123 | 0.201791 | 1.176175 |
| P | -0.337865 | -0.371957 | -1.849719 |
| C | 2.430915 | -0.524570 | -1.198054 |
| C | 1.593504 | -2.848226 | -0.664121 |
| C | 4.119707 | -3.834542 | -1.734028 |
| H | 3.322176 | -4.532573 | -1.490347 |
| C | 0.372697 | -2.981073 | -1.314960 |
| C | 0.820140 | 3.015806 | 0.663109 |
| C | 1.789080 | 2.246366 | 1.493070 |
| C | -0.263751 | 0.699380 | -3.382180 |
| C | 1.860110 | -3.658992 | 0.488401 |
| C | 1.814225 | 0.852423 | 1.502538 |
| C | 2.699896 | 2.971214 | 2.334513 |
| C | 2.814170 | 0.172575 | 2.240970 |
| H | 2.845996 | -0.916624 | 2.190929 |
| C | 1.120208 | 0.125485 | -0.813510 |
| H | 1.205005 | 1.201130 | -1.030887 |
| C | -0.547028 | 2.903396 | 0.890539 |
| C | -1.062152 | 1.898873 | 1.872872 |
| H | -2.100302 | 2.108846 | 2.158380 |
| H | -0.471489 | 1.899833 | 2.800942 |
| C | 4.945071 | -1.550480 | -1.986879 |
| C | 2.632808 | -1.903187 | -1.164182 |
| C | 0.914986 | -4.662611 | 0.883221 |
| C | 0.341738 | 4.749037 | -1.038663 |
| C | 0.845378 | 0.005394 | 0.705476 |
| H | 1.030779 | -1.046446 | 0.971677 |
| C | 6.184960 | -2.080797 | -2.416744 |
| H | 6.980162 | -1.386649 | -2.686742 |
| C | 4.575276 | 2.972883 | 3.928219 |
| H | 5.319860 | 2.412162 | 4.492330 |
| C | 0.026037 | -2.071555 | -2.452724 |
| H | -0.853817 | -2.436539 | -2.997153 |
| H | 0.852164 | -2.000595 | -3.175684 |
| C | 3.683180 | 2.258154 | 3.093797 |
| C | 1.172531 | -5.440252 | 2.038550 |
| H | 0.445553 | -6.199800 | 2.325207 |
| C | 2.647335 | 3.985396 | -0.741796 |
| H | 3.377914 | 3.362277 | -0.228283 |
| C | 3.054692 | 4.825813 | -1.752596 |
| H | 4.103993 | 4.855048 | -2.039206 |
| C | -0.275412 | -4.817762 | 0.134296 |
| H | -0.989771 | -5.586794 | 0.428801 |
| C | 3.886281 | -2.440210 | -1.614772 |
| C | 3.730891 | 0.849112 | 3.000230 |
| H | 4.497361 | 0.307992 | 3.553710 |
| C | 4.722735 | -0.156508 | -1.923311 |
| H | 5.535609 | 0.520782 | -2.182568 |
| C | 1.284608 | 3.914757 | -0.352407 |
| C | 3.230593 | -4.243319 | 2.412682 |
| H | 4.123657 | -4.081888 | 3.013044 |
| C | 3.497152 | 0.334715 | -1.560064 |
| H | 3.324814 | 1.411855 | -1.531717 |
| C | 0.799600 | 5.620363 | -2.056503 |
| H | 0.071390 | 6.252947 | -2.563338 |
| C | -1.032264 | 4.648452 | -0.718466 |
| H | -1.746358 | 5.290053 | -1.233632 |
| C | -1.237263 | 0.118914 | -4.408928 |
| H | -1.307153 | 0.793905 | -5.270723 |
| H | -2.256935 | 0.007153 | -4.012907 |
| H | -0.913327 | -0.858155 | -4.786790 |
| C | 3.516939 | 5.045620 | 3.309681 |
| H | 3.445167 | 6.126507 | 3.410474 |
| C | 3.020028 | -3.482439 | 1.286344 |
| H | 3.750904 | -2.729669 | 0.997464 |
| C | 2.644096 | 4.381462 | 2.480323 |
| H | 1.887195 | 4.944069 | 1.938991 |
| C | -1.464509 | 3.727671 | 0.200727 |
| H | -2.526926 | 3.645315 | 0.427349 |

| | | | |
|---|---|---|---|
| C | 2.302231 | -5.234426 | 2.792935 |
| H | 2.483560 | -5.833274 | 3.682778 |
| C | 5.331312 | -4.318300 | -2.169316 |
| H | 5.479944 | -5.392437 | -2.259013 |
| C | -0.687794 | 2.105512 | -2.974206 |
| H | -0.015854 | 2.522674 | -2.215327 |
| H | -1.703187 | 2.139562 | -2.560480 |
| H | -0.651471 | 2.779802 | -3.840374 |
| C | -1.351162 | -2.336801 | 2.119084 |
| H | -1.425666 | -3.049204 | 2.952409 |
| H | -0.473484 | -2.620062 | 1.528224 |
| H | -2.235157 | -2.462546 | 1.485170 |
| C | 2.126337 | 5.657240 | -2.412758 |
| H | 2.463515 | 6.324477 | -3.203126 |
| C | 4.498453 | 4.340130 | 4.035401 |
| H | 5.184986 | 4.879305 | 4.684649 |
| C | -0.012836 | -0.880424 | 3.612047 |
| H | -0.288715 | -1.422015 | 4.526453 |
| H | 0.284715 | 0.132862 | 3.908986 |
| H | 0.866966 | -1.384408 | 3.195057 |
| C | 6.379904 | -3.437595 | -2.503952 |
| H | 7.335209 | -3.834205 | -2.840684 |
| C | -1.201516 | -0.916731 | 2.651109 |
| C | -0.549712 | -3.975262 | -0.912311 |
| H | -1.480818 | -4.075906 | -1.469676 |
| C | 1.143704 | 0.753066 | -3.977597 |
| H | 1.586295 | -0.235900 | -4.149375 |
| H | 1.830502 | 1.322209 | -3.340993 |
| H | 1.103140 | 1.267033 | -4.946802 |
| C | -2.461593 | -0.447666 | 3.379958 |
| H | -3.298997 | -0.265919 | 2.697193 |
| H | -2.283385 | 0.473674 | 3.947794 |
| H | -2.779246 | -1.216831 | 4.095880 |
| Ni | -2.153638 | -0.132885 | -0.609427 |
| H | -2.965779 | 0.098195 | -2.066661 |
| C | -4.157661 | -3.886756 | -0.272428 |
| C | -3.957368 | -2.552077 | -0.604488 |
| C | -4.327740 | -1.518888 | 0.280304 |
| C | -4.925167 | -1.880519 | 1.498531 |
| C | -5.132472 | -3.214646 | 1.822496 |
| C | -4.742730 | -4.225278 | 0.944873 |
| H | -3.868473 | -4.666474 | -0.975020 |
| H | -3.509318 | -2.319833 | -1.574799 |
| H | -5.226382 | -1.102700 | 2.196520 |
| H | -5.607489 | -3.467150 | 2.768317 |
| H | -4.911305 | -5.269040 | 1.199799 |
| C | -3.984500 | -0.114048 | -0.037739 |
| C | -3.997306 | 0.162496 | -1.548091 |
| N | -4.512080 | 0.902570 | 0.803344 |
| H | -3.874419 | 1.642199 | 1.074214 |
| C | -5.845538 | 1.321284 | 0.949751 |
| O | -6.070666 | 2.342312 | 1.567386 |
| C | -6.923879 | 0.460898 | 0.351772 |
| H | -6.576068 | -0.252396 | -0.400389 |
| H | -7.422239 | -0.103604 | 1.147762 |
| H | -7.673067 | 1.118011 | -0.096458 |
| N | -4.457512 | 1.544381 | -1.843925 |
| O | -3.704297 | 2.453912 | -1.502944 |
| O | -5.567573 | 1.661802 | -2.339395 |
| H | -4.610771 | -0.537510 | -2.120804 |

**VII$_{S2}$**

| | | | |
|---|---|---|---|
| P | -0.668032 | -0.255976 | 0.807921 |
| P | 0.618275 | -1.071055 | -1.801702 |
| C | 3.112000 | -0.020524 | -0.940533 |
| C | 3.100219 | -2.324392 | 0.095650 |
| C | 5.963251 | -2.421597 | -0.484439 |
| H | 5.454155 | -3.303540 | -0.103483 |
| C | 2.127373 | -3.029470 | -0.606571 |
| C | 0.099357 | 2.920286 | -0.072779 |
| C | 1.042711 | 2.702205 | 1.059937 |
| C | 0.449279 | -0.402664 | -3.538628 |
| C | 3.446688 | -2.749149 | 1.420612 |
| C | 1.557423 | 1.446204 | 1.372943 |
| C | 1.424477 | 3.835715 | 1.856325 |
| C | 2.543079 | 1.321124 | 2.382555 |
| H | 2.964169 | 0.332984 | 2.574010 |
| C | 1.615917 | 0.137296 | -0.809519 |
| H | 1.349755 | 1.106656 | -1.250870 |
| C | -1.165883 | 2.342817 | -0.075071 |
| C | -1.553621 | 1.351644 | 0.975394 |
| H | -2.630303 | 1.143837 | 0.944793 |
| H | -1.332031 | 1.734694 | 1.982858 |
| C | 5.911358 | -0.125034 | -1.310839 |
| C | 3.797077 | -1.165177 | -0.533869 |
| C | 2.878463 | -3.955338 | 1.946588 |
| C | -0.489953 | 4.126185 | -2.156122 |
| C | 1.138310 | 0.180470 | 0.662117 |

| | | | |
|---|---|---|---|
| H | 1.621867 | -0.663492 | 1.176283 |
| C | 7.309889 | -0.202390 | -1.516748 |
| H | 7.821277 | 0.666386 | -1.930259 |
| C | 2.781343 | 4.794034 | 3.672199 |
| H | 3.534603 | 4.653201 | 4.446546 |
| C | 1.680995 | -2.556201 | -1.954855 |
| H | 1.105333 | -3.326081 | -2.481727 |
| H | 2.541880 | -2.283875 | -2.579898 |
| C | 2.404663 | 3.677605 | 2.888415 |
| C | 3.225949 | -4.373829 | 3.253652 |
| H | 2.792659 | -5.298267 | 3.634563 |
| C | 1.797834 | 4.275837 | -1.307598 |
| H | 2.547414 | 4.019444 | -0.561064 |
| C | 2.142469 | 5.063798 | -2.382507 |
| H | 3.164703 | 5.424173 | -2.480181 |
| C | 1.946671 | -4.676954 | 1.163625 |
| H | 1.522029 | -5.596740 | 1.564796 |
| C | 5.213860 | -1.248134 | -0.757944 |
| C | 2.969672 | 2.401184 | 3.107654 |
| H | 3.735167 | 2.285459 | 3.873908 |
| C | 5.181881 | 1.033326 | -1.659351 |
| H | 5.714422 | 1.888462 | -2.074255 |
| C | 0.477259 | 3.779076 | -1.157652 |
| C | 4.618256 | -2.426802 | 3.529773 |
| H | 5.284156 | -1.832649 | 4.152477 |
| C | 3.822280 | 1.072121 | -1.496206 |
| H | 3.260133 | 1.961886 | -1.786863 |
| C | -0.107831 | 4.956555 | -3.236796 |
| H | -0.858063 | 5.214752 | -3.983788 |
| C | -1.799849 | 3.604993 | -2.052027 |
| H | -2.547128 | 3.904162 | -2.787620 |
| C | -0.500388 | -1.334728 | -4.295713 |
| H | -0.575447 | -0.996847 | -5.337847 |
| H | -1.502927 | -1.324305 | -3.855084 |
| H | -0.148340 | -2.373213 | -4.311378 |
| C | 1.218405 | 6.180526 | 2.476360 |
| H | 0.748305 | 7.151024 | 2.331417 |
| C | 4.318342 | -2.003465 | 2.255335 |
| H | 4.754553 | -1.081390 | 1.875103 |
| C | 0.841006 | 5.118030 | 1.688440 |
| H | 0.071049 | 5.260230 | 0.933714 |
| C | -2.112643 | 2.712699 | -1.059057 |
| H | -3.115147 | 2.289649 | -0.997032 |
| C | 4.076809 | -3.627505 | 4.033211 |

| | | | |
|---|---|---|---|
| H | 4.330165 | -3.956849 | 5.038656 |
| C | 7.319235 | -2.469467 | -0.708649 |
| H | 7.866891 | -3.385061 | -0.494475 |
| C | -0.164706 | 0.989697 | -3.443911 |
| H | 0.540216 | 1.735504 | -3.054816 |
| H | -1.057449 | 0.991360 | -2.807855 |
| H | -0.463280 | 1.330382 | -4.445233 |
| C | -2.383546 | -1.109073 | 2.789124 |
| H | -2.543455 | -1.629456 | 3.742640 |
| H | -2.931141 | -1.664673 | 2.019240 |
| H | -2.831777 | -0.112056 | 2.890706 |
| C | 1.182361 | 5.414579 | -3.353781 |
| H | 1.466601 | 6.043896 | -4.194276 |
| C | 2.203993 | 6.024296 | 3.471836 |
| H | 2.497206 | 6.874729 | 4.083500 |
| C | -0.285765 | -2.453316 | 2.430789 |
| H | 0.772845 | -2.420608 | 2.146180 |
| H | -0.808213 | -3.097121 | 1.715114 |
| H | -0.339374 | -2.925544 | 3.421267 |
| C | 8.005445 | -1.348056 | -1.217543 |
| H | 9.079463 | -1.398029 | -1.383561 |
| C | -0.883604 | -1.051512 | 2.491970 |
| C | 1.551654 | -4.201041 | -0.060186 |
| H | 0.806941 | -4.737189 | -0.649302 |
| C | 1.800506 | -0.330357 | -4.251400 |
| H | 2.215029 | -1.321386 | -4.467000 |
| H | 2.548209 | 0.240664 | -3.686862 |
| H | 1.662523 | 0.175565 | -5.216193 |
| C | -0.165909 | -0.263472 | 3.589119 |
| H | -0.422946 | 0.802553 | 3.614693 |
| H | 0.923005 | -0.345359 | 3.503868 |
| H | -0.446472 | -0.687608 | 4.562304 |
| Ni | -1.183916 | -1.589830 | -0.792971 |
| H | -5.228343 | -2.565586 | 0.662904 |
| C | -6.230824 | 0.364904 | 3.455416 |
| C | -5.834909 | -0.361340 | 2.336563 |
| C | -5.298653 | 0.285606 | 1.217980 |
| C | -5.181281 | 1.678539 | 1.247683 |
| C | -5.573239 | 2.405450 | 2.368139 |
| C | -6.097526 | 1.750236 | 3.477901 |
| H | -6.652475 | -0.158219 | 4.311105 |
| H | -5.951426 | -1.444336 | 2.349765 |
| H | -4.780154 | 2.204939 | 0.382097 |
| H | -5.473659 | 3.488970 | 2.367339 |

| | | | |
|---|---|---|---|
| H | -6.408680 | 2.316781 | 4.352661 |
| C | -4.781710 | -0.507210 | 0.035032 |
| C | -5.442919 | -1.865226 | -0.146182 |
| N | -4.814768 | 0.263871 | -1.200030 |
| H | -5.702234 | 0.695932 | -1.431081 |
| N | -6.945252 | -1.761426 | -0.243968 |
| O | -7.405940 | -0.725106 | -0.720620 |
| O | -7.592423 | -2.724927 | 0.133967 |
| H | -5.116103 | -2.326091 | -1.084088 |
| C | -3.112740 | -4.838810 | -1.169432 |
| H | -3.591498 | -4.858525 | -2.154135 |
| H | -2.420860 | -5.687894 | -1.142112 |
| H | -3.864602 | -4.967489 | -0.387608 |
| C | -2.357071 | -3.569993 | -0.992576 |
| O | -1.479658 | -3.207674 | -1.845608 |
| O | -2.550239 | -2.804246 | 0.010935 |
| H | -3.715759 | -0.738137 | 0.191472 |
| C | -3.886904 | 0.086345 | -2.170853 |
| C | -4.095504 | 0.847035 | -3.447031 |
| H | -4.082615 | 0.152869 | -4.293351 |
| H | -5.025796 | 1.422299 | -3.471879 |
| H | -3.252626 | 1.534393 | -3.587820 |
| O | -2.897358 | -0.643769 | -2.012536 |

**TSIII$_{S2}$**

| | | | |
|---|---|---|---|
| P | 0.807401 | -0.074469 | 1.071626 |
| P | -0.082557 | 0.737611 | -1.723117 |
| C | -2.779672 | 0.274964 | -1.076172 |
| C | -2.462094 | 2.640078 | -0.231495 |
| C | -5.044416 | 3.245553 | -1.438631 |
| H | -4.419230 | 4.050722 | -1.059783 |
| C | -1.255994 | 3.108171 | -0.745255 |
| C | -0.662310 | -3.052938 | 0.491057 |
| C | -1.647239 | -2.474890 | 1.446653 |
| C | -0.136164 | -0.003061 | -3.451482 |
| C | -2.989641 | 3.229156 | 0.964385 |
| C | -1.840302 | -1.101359 | 1.559592 |
| C | -2.419466 | -3.364713 | 2.267737 |
| C | -2.871128 | -0.602799 | 2.394420 |
| H | -3.027975 | 0.476177 | 2.435739 |
| C | -1.371023 | -0.139210 | -0.728582 |
| H | -1.243400 | -1.185543 | -1.039037 |
| C | 0.697683 | -2.795831 | 0.628257 |
| C | 1.190475 | -1.795298 | 1.628140 |
| H | 2.278282 | -1.864652 | 1.757115 |
| H | 0.727976 | -1.947006 | 2.613011 |
| C | -5.385634 | 0.889217 | -1.978474 |
| C | -3.248877 | 1.577653 | -0.921802 |
| C | -2.335141 | 4.364217 | 1.544149 |
| C | -0.153997 | -4.550471 | -1.416600 |
| C | -1.039954 | -0.086107 | 0.776276 |
| H | -1.339131 | 0.908374 | 1.132463 |
| C | -6.671127 | 1.221065 | -2.468312 |
| H | -7.293283 | 0.423726 | -2.873562 |
| C | -4.225477 | -3.713824 | 3.902884 |
| H | -5.010144 | -3.290624 | 4.529291 |
| C | -0.657553 | 2.470550 | -1.959589 |
| H | 0.202878 | 3.037829 | -2.331025 |
| H | -1.403048 | 2.432297 | -2.765071 |
| C | -3.452062 | -2.835402 | 3.107744 |
| C | -2.864360 | 4.942596 | 2.724055 |
| H | -2.357941 | 5.808104 | 3.150411 |
| C | -2.489352 | -4.112972 | -0.847297 |
| H | -3.234936 | -3.631580 | -0.217545 |
| C | -2.892402 | -4.904521 | -1.898487 |
| H | -3.954544 | -5.041984 | -2.092312 |
| C | -1.166339 | 4.868554 | 0.927108 |
| H | -0.682761 | 5.748030 | 1.351414 |
| C | -4.550692 | 1.915100 | -1.428554 |
| C | -3.667432 | -1.438999 | 3.130043 |
| H | -4.463855 | -1.035557 | 3.754738 |
| C | -4.899298 | -0.436768 | -2.031147 |
| H | -5.542746 | -1.220536 | -2.429491 |
| C | -1.113264 | -3.904686 | -0.570785 |
| C | -4.623989 | 3.296657 | 2.766604 |
| H | -5.507316 | 2.882533 | 3.249068 |
| C | -3.627151 | -0.724918 | -1.614686 |
| H | -3.242067 | -1.742331 | -1.696912 |
| C | -0.601786 | -5.372989 | -2.477905 |
| H | 0.142892 | -5.861741 | -3.105710 |
| C | 1.222510 | -4.342302 | -1.171956 |
| H | 1.952086 | -4.879788 | -1.778003 |
| C | 1.012279 | 0.591757 | -4.274088 |
| H | 0.919672 | 0.236642 | -5.308750 |
| H | 1.996085 | 0.304251 | -3.902909 |
| H | 0.960005 | 1.687439 | -4.303102 |
| C | -2.949492 | -5.590098 | 3.098511 |
| H | -2.745596 | -6.658861 | 3.112458 |

| | | | |
|---|---|---|---|
| C | -4.144286 | 2.720168 | 1.614010 |
| H | -4.651826 | 1.853934 | 1.194682 |
| C | -2.188758 | -4.763660 | 2.304353 |
| H | -1.388060 | -5.188947 | 1.703753 |
| C | 1.629941 | -3.472296 | -0.192965 |
| H | 2.690728 | -3.314956 | -0.001523 |
| C | -3.984724 | 4.422199 | 3.325823 |
| H | -4.377733 | 4.873271 | 4.234464 |
| C | -6.292318 | 3.538132 | -1.936871 |
| H | -6.640647 | 4.568909 | -1.940476 |
| C | -0.031285 | -1.527063 | -3.318476 |
| H | -0.994373 | -1.972195 | -3.033170 |
| H | 0.720922 | -1.846102 | -2.589626 |
| H | 0.242744 | -1.959412 | -4.289875 |
| C | 2.426830 | 0.907770 | 3.118205 |
| H | 2.520039 | 1.497113 | 4.039910 |
| H | 3.119598 | 1.339915 | 2.391687 |
| H | 2.759471 | -0.110520 | 3.352836 |
| C | -1.943143 | -5.546265 | -2.720418 |
| H | -2.274863 | -6.175916 | -3.543115 |
| C | -3.985852 | -5.066317 | 3.897797 |
| H | -4.584235 | -5.731766 | 4.516243 |
| C | 0.604813 | 2.402051 | 2.305611 |
| H | -0.446636 | 2.494092 | 2.010715 |
| H | 1.228520 | 2.817195 | 1.506817 |
| H | 0.741437 | 3.030697 | 3.195987 |
| C | -7.121637 | 2.518473 | -2.446063 |
| H | -8.110082 | 2.763207 | -2.828972 |
| C | 0.981330 | 0.964293 | 2.638648 |
| C | -0.628709 | 4.236252 | -0.165320 |
| H | 0.286050 | 4.614171 | -0.622678 |
| C | -1.443503 | 0.331314 | -4.185784 |
| H | -1.500770 | 1.383311 | -4.485273 |
| H | -2.345763 | 0.075799 | -3.622272 |
| H | -1.459909 | -0.258646 | -5.111651 |
| C | 0.058186 | 0.466545 | 3.753066 |
| H | 0.213740 | -0.584617 | 4.021598 |
| H | -1.000957 | 0.603131 | 3.514093 |
| H | 0.263330 | 1.059154 | 4.654607 |
| Ni | 1.839976 | 0.550900 | -0.722756 |
| H | 5.259897 | 2.030283 | -1.000845 |
| C | 6.411449 | 1.435577 | 2.851593 |
| C | 5.745979 | 1.408124 | 1.633570 |
| C | 5.065967 | 0.256777 | 1.206068 |
| C | 5.083831 | -0.864201 | 2.050783 |
| C | 5.752182 | -0.836256 | 3.270323 |
| C | 6.419193 | 0.314259 | 3.677879 |
| H | 6.936911 | 2.339509 | 3.151844 |
| H | 5.754532 | 2.299550 | 1.012071 |
| H | 4.554704 | -1.772667 | 1.764290 |
| H | 5.751988 | -1.722578 | 3.901583 |
| H | 6.947551 | 0.335590 | 4.628469 |
| C | 4.334142 | 0.237866 | -0.107181 |
| C | 5.101497 | 0.977339 | -1.225765 |
| N | 4.186268 | -1.164239 | -0.518917 |
| H | 5.010707 | -1.726601 | -0.315414 |
| N | 6.480492 | 0.400173 | -1.404259 |
| O | 6.548909 | -0.726937 | -1.902948 |
| O | 7.429460 | 1.060163 | -1.006669 |
| H | 4.586992 | 0.867193 | -2.184227 |
| C | 3.083871 | 4.504636 | -1.150773 |
| H | 2.518272 | 4.804911 | -2.035384 |
| H | 2.799820 | 5.113293 | -0.287143 |
| H | 4.146508 | 4.702454 | -1.336669 |
| C | 2.901126 | 3.052938 | -0.850648 |
| O | 2.414447 | 2.273057 | -1.712506 |
| O | 3.306322 | 2.636638 | 0.297856 |
| H | 3.422880 | 1.378603 | 0.170043 |
| C | 3.584168 | -1.465073 | -1.690775 |
| C | 4.020073 | -2.690369 | -2.419020 |
| H | 4.873577 | -2.426000 | -3.055461 |
| H | 4.355593 | -3.484544 | -1.743434 |
| H | 3.210477 | -3.058003 | -3.053341 |
| O | 2.710458 | -0.705132 | -2.148204 |

**VIII$_{S2}$**

| | | | |
|---|---|---|---|
| P | -0.836581 | 0.068190 | 1.139996 |
| P | -0.012090 | -0.563207 | -1.832150 |
| C | 2.727659 | -0.305029 | -1.065380 |
| C | 2.219799 | -2.691985 | -0.370878 |
| C | 4.905916 | -3.357238 | -1.274415 |
| H | 4.210134 | -4.148626 | -1.006454 |
| C | 1.059172 | -3.044735 | -1.055702 |
| C | 0.641401 | 3.065572 | 0.463246 |
| C | 1.669689 | 2.477860 | 1.366838 |
| C | 0.118603 | 0.293888 | -3.508002 |
| C | 2.575462 | -3.422405 | 0.811438 |
| C | 1.831781 | 1.098651 | 1.489386 |

| | | | |
|---|---|---|---|
| C | 2.500168 | 3.354455 | 2.144220 |
| C | 2.874761 | 0.581104 | 2.297592 |
| H | 2.993168 | -0.502376 | 2.357331 |
| C | 1.323007 | 0.179865 | -0.786633 |
| H | 1.274123 | 1.242028 | -1.063699 |
| C | -0.706301 | 2.782332 | 0.658186 |
| C | -1.123737 | 1.799707 | 1.706222 |
| H | -2.181040 | 1.903999 | 1.970186 |
| H | -0.536008 | 1.940976 | 2.621397 |
| C | 5.398413 | -1.002828 | -1.679039 |
| C | 3.126926 | -1.629987 | -0.896933 |
| C | 1.800750 | -4.562524 | 1.202522 |
| C | 0.015096 | 4.577282 | -1.397726 |
| C | 0.998226 | 0.095916 | 0.721233 |
| H | 1.320924 | -0.903426 | 1.037751 |
| C | 6.719990 | -1.372579 | -2.025887 |
| H | 7.414407 | -0.588456 | -2.326363 |
| C | 4.393331 | 3.673180 | 3.684588 |
| H | 5.196970 | 3.235800 | 4.276193 |
| C | 0.594443 | -2.258634 | -2.238405 |
| H | -0.215993 | -2.777010 | -2.761681 |
| H | 1.421422 | -2.127936 | -2.948282 |
| C | 3.556453 | 2.806579 | 2.941731 |
| C | 2.141000 | -5.266542 | 2.382254 |
| H | 1.543803 | -6.135115 | 2.658215 |
| C | 2.382455 | 4.169323 | -0.955761 |
| H | 3.167790 | 3.692104 | -0.373209 |
| C | 2.718836 | 4.983144 | -2.013707 |
| H | 3.767126 | 5.137035 | -2.262244 |
| C | 0.703351 | -4.955981 | 0.403980 |
| H | 0.148345 | -5.855170 | 0.671337 |
| C | 4.465457 | -2.008266 | -1.266264 |
| C | 3.727204 | 1.404709 | 2.983771 |
| H | 4.535622 | 0.988491 | 3.583968 |
| C | 4.972091 | 0.342060 | -1.747469 |
| H | 5.686641 | 1.109287 | -2.043798 |
| C | 1.026124 | 3.938050 | -0.608525 |
| C | 3.950095 | -3.732522 | 2.798167 |
| H | 4.780806 | -3.411275 | 3.423501 |
| C | 3.671589 | 0.670770 | -1.471703 |
| H | 3.336832 | 1.705788 | -1.562486 |
| C | 0.395300 | 5.426723 | -2.464012 |
| H | -0.388816 | 5.910516 | -3.046111 |
| C | -1.344998 | 4.315799 | -1.113930 |
| H | -2.111835 | 4.826258 | -1.697428 |
| C | -0.905192 | -0.344843 | -4.453729 |
| H | -0.850689 | 0.162104 | -5.426425 |
| H | -1.930916 | -0.272702 | -4.090473 |
| H | -0.674812 | -1.401938 | -4.637337 |
| C | 3.127418 | 5.573777 | 2.923301 |
| H | 2.951179 | 6.647532 | 2.935872 |
| C | 3.657954 | -3.039100 | 1.645292 |
| H | 4.264499 | -2.179619 | 1.366163 |
| C | 2.306145 | 4.759347 | 2.179022 |
| H | 1.486201 | 5.198854 | 1.616225 |
| C | -1.689890 | 3.422303 | -0.132668 |
| H | -2.738550 | 3.212605 | 0.074446 |
| C | 3.190474 | -4.860703 | 3.170619 |
| H | 3.438267 | -5.406164 | 4.078660 |
| C | 6.192196 | -3.686567 | -1.633418 |
| H | 6.496926 | -4.730987 | -1.638213 |
| C | -0.121593 | 1.792684 | -3.310332 |
| H | 0.766284 | 2.282287 | -2.888936 |
| H | -0.970494 | 2.015829 | -2.659015 |
| H | -0.305753 | 2.266194 | -4.284648 |
| C | -2.295841 | -1.188119 | 3.166554 |
| H | -2.309420 | -1.733021 | 4.119961 |
| H | -2.843260 | -1.801205 | 2.444953 |
| H | -2.857984 | -0.258014 | 3.311240 |
| C | 1.719464 | 5.626635 | -2.771829 |
| H | 1.998964 | 6.274216 | -3.600038 |
| C | 4.190578 | 5.031471 | 3.673725 |
| H | 4.836840 | 5.687713 | 4.252630 |
| C | -0.155100 | -2.274182 | 2.492536 |
| H | 0.934717 | -2.192708 | 2.576236 |
| H | -0.380573 | -2.691387 | 1.501169 |
| H | -0.475059 | -3.014528 | 3.237434 |
| C | 7.115988 | -2.687327 | -2.000430 |
| H | 8.132787 | -2.960413 | -2.274229 |
| C | -0.853191 | -0.938675 | 2.752297 |
| C | 0.338172 | -4.201529 | -0.680790 |
| H | -0.486771 | -4.518384 | -1.316500 |
| C | 1.500203 | 0.138232 | -4.154944 |
| H | 1.729195 | -0.891135 | -4.450626 |
| H | 2.315337 | 0.505233 | -3.524430 |
| H | 1.507229 | 0.735840 | -5.076384 |
| C | -0.156948 | -0.219414 | 3.909886 |
| H | -0.725121 | 0.648612 | 4.262277 |

| | | | |
|---|---|---|---|
| H | 0.861744 | 0.103689 | 3.674135 |
| H | -0.088743 | -0.917591 | 4.755069 |
| Ni | -2.006073 | -0.340206 | -0.672668 |
| H | -4.650603 | -2.063237 | -0.432629 |
| C | -5.711811 | -0.746147 | 3.246482 |
| C | -5.175555 | -0.909463 | 1.975122 |
| C | -4.571012 | 0.161352 | 1.294273 |
| C | -4.573581 | 1.410369 | 1.936540 |
| C | -5.098520 | 1.574625 | 3.214132 |
| C | -5.667646 | 0.493042 | 3.880126 |
| H | -6.175443 | -1.596783 | 3.741770 |
| H | -5.229643 | -1.896034 | 1.518419 |
| H | -4.164435 | 2.277411 | 1.419351 |
| H | -5.077645 | 2.557617 | 3.680884 |
| H | -6.090178 | 0.618747 | 4.874390 |
| C | -4.017546 | 0.014035 | -0.086754 |
| C | -4.762987 | -1.073554 | -0.867283 |
| N | -4.235693 | 1.288105 | -0.798767 |
| H | -5.137323 | 1.741562 | -0.662966 |
| N | -6.274367 | -0.863605 | -0.887345 |
| O | -6.691795 | 0.201517 | -1.342879 |
| O | -6.971009 | -1.769855 | -0.454260 |
| H | -4.469757 | -1.098077 | -1.919865 |
| C | -3.148679 | -4.760718 | -1.370858 |
| H | -2.855265 | -4.930526 | -2.408108 |
| H | -2.746316 | -5.541144 | -0.718985 |
| H | -4.242408 | -4.822153 | -1.308656 |
| C | -2.714770 | -3.409694 | -0.921078 |
| O | -2.435051 | -2.476648 | -1.667023 |
| O | -2.706497 | -3.281517 | 0.408739 |
| H | -2.411810 | -2.353393 | 0.591117 |
| C | -3.668645 | 1.386004 | -1.999920 |
| C | -4.180155 | 2.363699 | -2.996962 |
| H | -5.001334 | 1.906245 | -3.563343 |
| H | -4.576181 | 3.264564 | -2.517323 |
| H | -3.388547 | 2.636132 | -3.699565 |
| O | -2.715106 | 0.609661 | -2.267764 |

**IV$_{S3}$**

| | | | |
|---|---|---|---|
| P | 0.612332 | 0.133299 | -1.574665 |
| P | 0.603182 | -0.349894 | 1.509656 |
| C | -2.207224 | -0.285686 | 1.519067 |
| C | -1.800013 | -2.709740 | 0.942545 |
| C | -3.909257 | -3.356235 | 2.844722 |
| H | -3.314551 | -4.148631 | 2.395898 |
| C | -0.451536 | -2.955274 | 1.183993 |
| C | -1.345901 | 2.864218 | -0.942211 |
| C | -2.311838 | 1.945799 | -1.605523 |
| C | 0.776844 | 0.687247 | 3.078907 |
| C | -2.505330 | -3.526386 | -0.001101 |
| C | -2.190949 | 0.562606 | -1.493873 |
| C | -3.365072 | 2.496900 | -2.409593 |
| C | -3.155200 | -0.281381 | -2.096165 |
| H | -3.057510 | -1.359461 | -1.967996 |
| C | -1.007485 | 0.196368 | 0.740710 |
| H | -0.962369 | 1.286368 | 0.842598 |
| C | -0.000283 | 2.839793 | -1.299487 |
| C | 0.494297 | 1.829621 | -2.293353 |
| H | 1.475001 | 2.112274 | -2.691433 |
| H | -0.202262 | 1.760819 | -3.138277 |
| C | -4.382589 | -0.983781 | 3.184745 |
| C | -2.530953 | -1.632395 | 1.666800 |
| C | -1.841098 | -4.644656 | -0.601521 |
| C | -0.864298 | 4.721237 | 0.621335 |
| C | -1.052612 | -0.113727 | -0.765217 |
| H | -1.210850 | -1.192913 | -0.853436 |
| C | -5.439588 | -1.352115 | 4.050824 |
| H | -6.025129 | -0.561960 | 4.519482 |
| C | -5.365677 | 2.167120 | -3.804528 |
| H | -6.090955 | 1.485768 | -4.247951 |
| C | 0.327222 | -2.072634 | 2.113961 |
| H | 1.310476 | -2.506495 | 2.332924 |
| H | -0.206960 | -1.974777 | 3.067737 |
| C | -4.326275 | 1.622086 | -3.012928 |
| C | -2.528016 | -5.432014 | -1.557251 |
| H | -2.006936 | -6.278071 | -2.004337 |
| C | -3.127440 | 3.799109 | 0.543661 |
| H | -3.847084 | 3.092139 | 0.135166 |
| C | -3.516276 | 4.673637 | 1.531992 |
| H | -4.543357 | 4.656562 | 1.891874 |
| C | -0.505558 | -4.924270 | -0.233524 |
| H | -0.019434 | -5.806779 | -0.648981 |
| C | -3.602881 | -2.003100 | 2.546992 |
| C | -4.202724 | 0.228209 | -2.817642 |
| H | -4.941398 | -0.438302 | -3.262440 |
| C | -4.069316 | 0.372292 | 2.938937 |
| H | -4.675101 | 1.150203 | 3.403238 |
| C | -1.793329 | 3.787182 | 0.058654 |

| | | | |
|---|---|---|---|
| C | -4.476353 | -4.031392 | -1.335873 |
| H | -5.496942 | -3.793523 | -1.629754 |
| C | -3.001073 | 0.705121 | 2.148555 |
| H | -2.749085 | 1.753448 | 1.987365 |
| C | -1.301185 | 5.617218 | 1.627087 |
| H | -0.582799 | 6.325353 | 2.039024 |
| C | 0.470811 | 4.722672 | 0.157098 |
| H | 1.161190 | 5.473492 | 0.541486 |
| C | 2.200318 | 0.539882 | 3.621012 |
| H | 2.269087 | 1.056171 | 4.587039 |
| H | 2.951909 | 0.984580 | 2.963041 |
| H | 2.466237 | -0.511004 | 3.801847 |
| C | -4.490898 | 4.383449 | -3.455154 |
| H | -4.548704 | 5.453092 | -3.646084 |
| C | -3.842255 | -3.255843 | -0.393497 |
| H | -4.362645 | -2.404873 | 0.041596 |
| C | -3.477700 | 3.887429 | -2.669016 |
| H | -2.741770 | 4.569940 | -2.250322 |
| C | 0.893664 | 3.798510 | -0.765737 |
| H | 1.919345 | 3.814072 | -1.127044 |
| C | -3.817301 | -5.130978 | -1.924304 |
| H | -4.331752 | -5.737484 | -2.666609 |
| C | -4.934368 | -3.680915 | 3.701708 |
| H | -5.143309 | -4.726409 | 3.918534 |
| C | 0.486175 | 2.147589 | 2.715204 |
| H | -0.591724 | 2.342254 | 2.643419 |
| H | 0.944570 | 2.459950 | 1.768439 |
| H | 0.879846 | 2.806179 | 3.500326 |
| C | 2.051499 | -1.299134 | -3.495645 |
| H | 2.041707 | -1.902224 | -4.413212 |
| H | 2.602201 | -1.862648 | -2.733994 |
| H | 2.602313 | -0.379086 | -3.723076 |
| C | -2.598532 | 5.594583 | 2.078768 |
| H | -2.921652 | 6.285715 | 2.854371 |
| C | -5.451395 | 3.520428 | -4.021521 |
| H | -6.250362 | 3.927690 | -4.637546 |
| C | 0.002804 | -2.376686 | -2.604870 |
| H | 0.242677 | -3.150111 | -3.347068 |
| H | -1.091128 | -2.341333 | -2.531887 |
| H | 0.407287 | -2.707433 | -1.638879 |
| C | -5.715200 | -2.673691 | 4.304806 |
| H | -6.528367 | -2.945721 | 4.974359 |
| C | 0.610569 | -1.047235 | -3.053287 |
| C | 0.176903 | -4.085685 | 0.611168 |
| H | 1.215579 | -4.288289 | 0.865391 |
| C | -0.185656 | 0.246046 | 4.189802 |
| H | 0.117365 | -0.701790 | 4.648446 |
| H | -1.228296 | 0.164004 | 3.868012 |
| H | -0.150877 | 1.002731 | 4.984008 |
| C | -0.186432 | -0.483250 | -4.230759 |
| H | 0.315287 | 0.369209 | -4.702543 |
| H | -1.204008 | -0.181144 | -3.954174 |
| H | -0.276089 | -1.265039 | -4.996379 |
| Ni | 2.161151 | -0.138727 | 0.012414 |
| H | 2.544112 | -0.445999 | 1.404383 |
| C | 4.150685 | 0.217669 | 0.280074 |
| C | 3.755429 | 0.147941 | -1.099950 |
| H | 4.007305 | -0.784926 | -1.595131 |
| N | 3.959706 | 1.247036 | -2.037244 |
| O | 4.518290 | 0.947403 | -3.093415 |
| O | 3.594529 | 2.384688 | -1.740902 |
| N | 4.896220 | -0.943858 | 0.683470 |
| H | 4.446728 | -1.609893 | 1.298355 |
| C | 5.986954 | -1.453105 | 0.027345 |
| C | 6.729657 | -0.545418 | -0.909681 |
| H | 6.346003 | -0.648759 | -1.932426 |
| H | 6.655210 | 0.513180 | -0.637214 |
| H | 7.778549 | -0.848354 | -0.923429 |
| O | 6.347680 | -2.610388 | 0.247341 |
| C | 4.452292 | 1.453301 | 1.045203 |
| C | 5.599869 | 1.504507 | 1.850666 |
| C | 3.578910 | 2.549327 | 1.063735 |
| C | 5.878650 | 2.629946 | 2.618169 |
| H | 6.274762 | 0.652782 | 1.879487 |
| C | 3.850303 | 3.665819 | 1.840854 |
| H | 2.670522 | 2.514355 | 0.467956 |
| C | 5.006708 | 3.714795 | 2.617941 |
| H | 6.779862 | 2.652337 | 3.226655 |
| H | 3.147733 | 4.497982 | 1.846254 |
| H | 5.222437 | 4.590822 | 3.225753 |
| C | 3.435286 | -3.939166 | -0.863088 |
| O | 3.037040 | -2.852839 | -0.443298 |
| C | 2.677516 | -4.807508 | -1.818223 |
| H | 1.625539 | -4.522668 | -1.851545 |
| H | 3.107092 | -4.690717 | -2.820716 |
| H | 2.782254 | -5.863584 | -1.552896 |
| O | 4.601620 | -4.477629 | -0.545872 |
| H | 5.165738 | -3.829417 | -0.039701 |

**V$_{S3}$**

| | | | |
|---|---|---|---|
| P | 0.569373 | 0.123405 | -1.639239 |
| P | 0.719566 | -0.388508 | 1.449263 |
| C | -2.122147 | -0.610756 | 1.474070 |
| C | -1.363241 | -2.919439 | 0.792050 |
| C | -3.588797 | -3.946729 | 2.381009 |
| H | -2.864765 | -4.632575 | 1.948010 |
| C | -0.029063 | -2.977964 | 1.177826 |
| C | -1.043229 | 2.958111 | -0.725166 |
| C | -2.170307 | 2.173913 | -1.306058 |
| C | 1.057680 | 0.799708 | 2.844033 |
| C | -1.817008 | -3.776044 | -0.264225 |
| C | -2.202781 | 0.781397 | -1.291781 |
| C | -3.266230 | 2.893878 | -1.895391 |
| C | -3.365369 | 0.098371 | -1.726421 |
| H | -3.384392 | -0.990058 | -1.649242 |
| C | -0.964246 | 0.066100 | 0.772944 |
| H | -1.016744 | 1.141901 | 1.001226 |
| C | 0.243850 | 2.864978 | -1.244350 |
| C | 0.570990 | 1.846666 | -2.290185 |
| H | 1.559017 | 2.036721 | -2.728460 |
| H | -0.158109 | 1.873129 | -3.113344 |
| C | -4.357072 | -1.673558 | 2.839854 |
| C | -2.305067 | -1.994676 | 1.485509 |
| C | -0.911810 | -4.732228 | -0.831597 |
| C | -0.249518 | 4.751489 | 0.792866 |
| C | -1.067489 | -0.066492 | -0.761041 |
| H | -1.317073 | -1.118730 | -0.968530 |
| C | -5.447754 | -2.221796 | 3.556302 |
| H | -6.162054 | -1.539231 | 4.015321 |
| C | -5.503812 | 2.887331 | -2.920984 |
| H | -6.375294 | 2.323310 | -3.251399 |
| C | 0.491389 | -2.031146 | 2.214617 |
| H | 1.452076 | -2.364992 | 2.623411 |
| H | -0.211048 | -1.913709 | 3.051816 |
| C | -4.420193 | 2.177895 | -2.350479 |
| C | -1.356629 | -5.567791 | -1.884611 |
| H | -0.658069 | -6.292673 | -2.300980 |
| C | -2.528628 | 3.902038 | 1.051501 |
| H | -3.332236 | 3.240692 | 0.731787 |
| C | -2.724065 | 4.753930 | 2.114115 |
| H | -3.679371 | 4.755353 | 2.635406 |
| C | 0.427545 | -4.774490 | -0.378833 |
| H | 1.117867 | -5.488670 | -0.825768 |
| C | -3.409484 | -2.548748 | 2.216070 |
| C | -4.449098 | 0.771064 | -2.222830 |
| H | -5.339167 | 0.228733 | -2.539180 |
| C | -4.172489 | -0.276661 | 2.736898 |
| H | -4.905443 | 0.388782 | 3.191300 |
| C | -1.290511 | 3.866595 | 0.358054 |
| C | -3.514814 | -4.495532 | -1.852148 |
| H | -4.519367 | -4.401487 | -2.259877 |
| C | -3.078113 | 0.233393 | 2.090709 |
| H | -2.935893 | 1.313210 | 2.026314 |
| C | -0.491601 | 5.629085 | 1.876925 |
| H | 0.309570 | 6.297434 | 2.191362 |
| C | 1.011035 | 4.696386 | 0.153447 |
| H | 1.796423 | 5.383201 | 0.469207 |
| C | 2.183959 | 0.231335 | 3.706542 |
| H | 2.496720 | 0.988012 | 4.437300 |
| H | 3.073845 | -0.029852 | 3.117992 |
| H | 1.869795 | -0.656650 | 4.267697 |
| C | -4.310020 | 4.959742 | -2.648829 |
| H | -4.263370 | 6.038325 | -2.784346 |
| C | -3.123509 | -3.685983 | -0.809965 |
| H | -3.824364 | -2.965984 | -0.390566 |
| C | -3.246475 | 4.300891 | -2.077391 |
| H | -2.368609 | 4.866598 | -1.774424 |
| C | 1.254990 | 3.753116 | -0.810868 |
| H | 2.236334 | 3.687723 | -1.277402 |
| C | -2.629063 | -5.451837 | -2.390190 |
| H | -2.954038 | -6.091475 | -3.207916 |
| C | -4.651656 | -4.447474 | 3.095220 |
| H | -4.760614 | -5.523492 | 3.213138 |
| C | 1.466938 | 2.119876 | 2.203499 |
| H | 0.673613 | 2.493157 | 1.547698 |
| H | 2.380986 | 2.025392 | 1.603500 |
| H | 1.643385 | 2.883723 | 2.973130 |
| C | 0.526253 | -2.375580 | -2.795923 |
| H | 0.437173 | -3.013155 | -3.685300 |
| H | -0.286730 | -2.660228 | -2.118623 |
| H | 1.469327 | -2.622027 | -2.298822 |
| C | -1.700414 | 5.630250 | 2.529915 |
| H | -1.870867 | 6.304409 | 3.366448 |
| C | -5.455725 | 4.252290 | -3.065743 |
| H | -6.291960 | 4.786984 | -3.510952 |
| C | -0.950168 | -0.712636 | -3.874826 |

| | | | |
|---|---|---|---|
| H | -0.926342 | -1.228539 | -4.843502 |
| H | -1.204433 | 0.335956 | -4.073609 |
| H | -1.768415 | -1.155134 | -3.297213 |
| C | -5.597660 | -3.581337 | 3.680915 |
| H | -6.437078 | -3.992082 | 4.237868 |
| C | 0.407038 | -0.909536 | -3.194060 |
| C | 0.863794 | -3.894472 | 0.577544 |
| H | 1.903683 | -3.912526 | 0.903212 |
| C | -0.192101 | 1.011345 | 3.699217 |
| H | -0.638740 | 0.074523 | 4.054939 |
| H | -0.965170 | 1.574483 | 3.164064 |
| H | 0.079199 | 1.601880 | 4.583376 |
| C | 1.507888 | -0.496335 | -4.173401 |
| H | 2.518114 | -0.569596 | -3.760305 |
| H | 1.358330 | 0.522307 | -4.551448 |
| H | 1.480467 | -1.166828 | -5.041423 |
| Ni | 2.165486 | -0.358844 | -0.150487 |
| H | 2.579331 | -0.667667 | 1.232903 |
| C | 4.175961 | -0.366768 | -0.048603 |
| C | 3.690157 | -0.561468 | -1.384531 |
| N | 4.811241 | -1.426483 | 0.670354 |
| H | 4.246647 | -2.242560 | 0.869731 |
| C | 6.157398 | -1.800281 | 0.481958 |
| C | 7.049650 | -0.821248 | -0.228675 |
| H | 6.606419 | -0.449062 | -1.160757 |
| H | 7.256465 | 0.052060 | 0.400840 |
| H | 7.993952 | -1.318446 | -0.454509 |
| O | 6.531202 | -2.868022 | 0.919663 |
| C | 4.549805 | 1.002324 | 0.390239 |
| C | 5.038545 | 1.190216 | 1.695086 |
| C | 4.391590 | 2.133670 | -0.425269 |
| C | 5.339211 | 2.459126 | 2.169866 |
| H | 5.187830 | 0.319905 | 2.330196 |
| C | 4.699533 | 3.403038 | 0.051174 |
| H | 4.039573 | 2.032509 | -1.451372 |
| C | 5.166406 | 3.574367 | 1.351810 |
| H | 5.718442 | 2.576865 | 3.182968 |
| H | 4.582960 | 4.264179 | -0.604845 |
| H | 5.409080 | 4.568898 | 1.719734 |
| N | 3.832570 | -1.893421 | -1.941952 |
| O | 4.289391 | -1.984781 | -3.077276 |
| O | 3.480163 | -2.851469 | -1.239645 |
| H | 3.837228 | 0.195291 | -2.149718 |

**TSII$_{S3}$**

| | | | |
|---|---|---|---|
| P | 0.538989 | 0.089953 | -1.658023 |
| P | 0.689559 | -0.415410 | 1.424414 |
| C | -2.152594 | -0.548186 | 1.478119 |
| C | -1.475602 | -2.879416 | 0.773011 |
| C | -3.715139 | -3.842823 | 2.375468 |
| H | -3.015317 | -4.548043 | 1.934173 |
| C | -0.139612 | -2.990452 | 1.141765 |
| C | -0.926281 | 2.988167 | -0.716147 |
| C | -2.097028 | 2.263378 | -1.288430 |
| C | 1.090134 | 0.725201 | 2.844143 |
| C | -1.974743 | -3.719665 | -0.277271 |
| C | -2.195401 | 0.873405 | -1.283939 |
| C | -3.162921 | 3.039029 | -1.861551 |
| C | -3.391778 | 0.248932 | -1.714397 |
| H | -3.461438 | -0.837893 | -1.645087 |
| C | -0.978407 | 0.095975 | 0.772821 |
| H | -0.995503 | 1.172342 | 1.004667 |
| C | 0.347923 | 2.836303 | -1.253179 |
| C | 0.609017 | 1.809953 | -2.309581 |
| H | 1.595795 | 1.954943 | -2.767224 |
| H | -0.135183 | 1.871153 | -3.117396 |
| C | -4.408721 | -1.550562 | 2.854918 |
| C | -2.377233 | -1.925988 | 1.482110 |
| C | -1.116846 | -4.716644 | -0.848046 |
| C | -0.029031 | 4.739359 | 0.792381 |
| C | -1.097682 | -0.027783 | -0.763959 |
| H | -1.396344 | -1.067029 | -0.972754 |
| C | -5.511944 | -2.069464 | 3.574032 |
| H | -6.201671 | -1.368067 | 4.042202 |
| C | -5.408545 | 3.145784 | -2.863886 |
| H | -6.308838 | 2.625844 | -3.189560 |
| C | 0.439065 | -2.066097 | 2.167324 |
| H | 1.404847 | -2.431171 | 2.536862 |
| H | -0.225260 | -1.940519 | 3.034795 |
| C | -4.354083 | 2.381725 | -2.309429 |
| C | -1.605012 | -5.536650 | -1.893910 |
| H | -0.940779 | -6.292245 | -2.312045 |
| C | -2.339470 | 3.993204 | 1.086017 |
| H | -3.176358 | 3.367503 | 0.780186 |
| C | -2.480068 | 4.852693 | 2.151446 |
| H | -3.425649 | 4.895554 | 2.688409 |
| C | 0.222772 | -4.816416 | -0.406098 |
| H | 0.876915 | -5.563361 | -0.853938 |

| | | | |
|---|---|---|---|
| C | -3.492956 | -2.450106 | 2.218396 |
| C | -4.447608 | 0.976543 | -2.193617 |
| H | -5.365102 | 0.479720 | -2.506628 |
| C | -4.180915 | -0.159415 | 2.760867 |
| H | -4.889199 | 0.525275 | 3.225640 |
| C | -1.115760 | 3.904205 | 0.372541 |
| C | -3.713694 | -4.371805 | -1.850353 |
| H | -4.716172 | -4.235764 | -2.251280 |
| C | -3.076594 | 0.320790 | 2.108734 |
| H | -2.899729 | 1.396069 | 2.051238 |
| C | -0.214941 | 5.628058 | 1.878453 |
| H | 0.620315 | 6.259399 | 2.180163 |
| C | 1.220023 | 4.623728 | 0.139037 |
| H | 2.040793 | 5.272213 | 0.445206 |
| C | 2.198826 | 0.086559 | 3.679496 |
| H | 2.560908 | 0.813887 | 4.417248 |
| H | 3.067728 | -0.209983 | 3.074845 |
| H | 1.849895 | -0.794167 | 4.231389 |
| C | -4.114518 | 5.157061 | -2.592083 |
| H | -4.017623 | 6.232944 | -2.721928 |
| C | -3.280033 | -3.575592 | -0.814497 |
| H | -3.946136 | -2.824221 | -0.393247 |
| C | -3.077950 | 4.444607 | -2.035913 |
| H | -2.171165 | 4.966913 | -1.740323 |
| C | 1.407482 | 3.670835 | -0.828769 |
| H | 2.378861 | 3.561943 | -1.308329 |
| C | -2.874911 | -5.368162 | -2.390619 |
| H | -3.233864 | -5.996970 | -3.202550 |
| C | -4.789178 | -4.315314 | 3.092124 |
| H | -4.930691 | -5.388332 | 3.203110 |
| C | 1.553996 | 2.040740 | 2.232071 |
| H | 0.773997 | 2.465780 | 1.591725 |
| H | 2.458645 | 1.917671 | 1.622810 |
| H | 1.771270 | 2.777517 | 3.017488 |
| C | 0.398539 | -2.409754 | -2.804383 |
| H | 0.260221 | -3.043890 | -3.689951 |
| H | -0.410636 | -2.657058 | -2.108200 |
| H | 1.341481 | -2.697664 | -2.329215 |
| C | -1.412506 | 5.683739 | 2.549557 |
| H | -1.540086 | 6.365639 | 3.387533 |
| C | -5.297133 | 4.507909 | -3.000732 |
| H | -6.111762 | 5.084713 | -3.433293 |
| C | -1.012951 | -0.691277 | -3.885988 |
| H | -1.011923 | -1.211704 | -4.852447 |
| H | -1.223692 | 0.366298 | -4.087963 |
| H | -1.848006 | -1.096748 | -3.304555 |
| C | -5.704579 | -3.424403 | 3.689180 |
| H | -6.553621 | -3.812295 | 4.247907 |
| C | 0.335596 | -0.940921 | -3.205322 |
| C | 0.705705 | -3.949525 | 0.539212 |
| H | 1.746821 | -4.009331 | 0.854872 |
| C | -0.142623 | 0.971513 | 3.714658 |
| H | -0.624161 | 0.046526 | 4.056037 |
| H | -0.895949 | 1.573190 | 3.192638 |
| H | 0.156470 | 1.535421 | 4.607188 |
| C | 1.456964 | -0.572310 | -4.179442 |
| H | 2.460155 | -0.669898 | -3.752481 |
| H | 1.341829 | 0.446571 | -4.568431 |
| H | 1.418593 | -1.250945 | -5.040618 |
| Ni | 2.125245 | -0.408861 | -0.189651 |
| H | 2.757593 | -0.593103 | 1.122431 |
| C | 4.129532 | -0.473190 | -0.004092 |
| C | 3.665881 | -0.643931 | -1.364495 |
| N | 4.745550 | -1.564373 | 0.696338 |
| H | 4.148936 | -2.348423 | 0.929021 |
| C | 6.058678 | -2.011072 | 0.449783 |
| C | 6.973361 | -1.081599 | -0.297904 |
| H | 6.520499 | -0.698637 | -1.221054 |
| H | 7.238242 | -0.211758 | 0.314543 |
| H | 7.885879 | -1.624956 | -0.546724 |
| O | 6.390124 | -3.098604 | 0.873235 |
| C | 4.610446 | 0.879448 | 0.409710 |
| C | 5.144678 | 1.044220 | 1.697699 |
| C | 4.502960 | 2.008511 | -0.413163 |
| C | 5.552711 | 2.293248 | 2.146512 |
| H | 5.247014 | 0.172217 | 2.340120 |
| C | 4.917046 | 3.257960 | 0.036294 |
| H | 4.100835 | 1.923805 | -1.421814 |
| C | 5.439265 | 3.408014 | 1.318135 |
| H | 5.969090 | 2.394799 | 3.146522 |
| H | 4.839039 | 4.118408 | -0.626172 |
| H | 5.766536 | 4.385569 | 1.665104 |
| N | 3.739702 | -1.987252 | -1.905474 |
| O | 4.210059 | -2.126374 | -3.029768 |
| O | 3.309912 | -2.910253 | -1.196471 |
| H | 3.898378 | 0.093879 | -2.127452 |

**$VI_{S3}$**

| | | | |
|---|---|---|---|
| P | 0.787904 | -0.124208 | -1.267359 |
| P | 0.092962 | -0.964164 | 1.624863 |
| C | -2.584361 | -0.132278 | 1.237097 |
| C | -2.540931 | -2.493777 | 0.339373 |
| C | -5.214163 | -2.803390 | 1.455190 |
| H | -4.705340 | -3.653740 | 1.007566 |
| C | -1.418279 | -3.091873 | 0.902292 |
| C | -0.034508 | 2.986484 | -0.292681 |
| C | -1.210781 | 2.664504 | -1.150773 |
| C | 0.636862 | -0.125706 | 3.195224 |
| C | -3.065041 | -3.011972 | -0.888535 |
| C | -1.662403 | 1.359366 | -1.340752 |
| C | -1.888744 | 3.744012 | -1.813102 |
| C | -2.843481 | 1.122253 | -2.086195 |
| H | -3.199454 | 0.095605 | -2.181056 |
| C | -1.157261 | 0.134579 | 0.815318 |
| H | -0.884359 | 1.136408 | 1.181782 |
| C | 1.233634 | 2.517750 | -0.617464 |
| C | 1.412572 | 1.536633 | -1.733145 |
| H | 2.469288 | 1.439970 | -2.005990 |
| H | 0.857569 | 1.840541 | -2.634140 |
| C | -5.233776 | -0.466140 | 2.163141 |
| C | -3.216980 | -1.357931 | 1.031011 |
| C | -2.481301 | -4.187154 | -1.463717 |
| C | 0.972014 | 4.265192 | 1.575708 |
| C | -0.983566 | 0.157103 | -0.724224 |
| H | -1.487393 | -0.745243 | -1.104768 |
| C | -6.546066 | -0.660409 | 2.656498 |
| H | -7.051620 | 0.180012 | 3.130803 |
| C | -3.752126 | 4.542148 | -3.209688 |
| H | -4.657870 | 4.317155 | -3.771787 |
| C | -0.781071 | -2.492136 | 2.118851 |
| H | -0.041466 | -3.171859 | 2.556982 |
| H | -1.520462 | -2.242585 | 2.893768 |
| C | -3.076949 | 3.477069 | -2.567654 |
| C | -2.990880 | -4.684053 | -2.687689 |
| H | -2.539789 | -5.580712 | -3.112026 |
| C | -1.463481 | 4.225268 | 1.340560 |
| H | -2.354750 | 3.909581 | 0.800928 |
| C | -1.585223 | 4.998175 | 2.472293 |
| H | -2.573674 | 5.283278 | 2.827303 |
| C | -1.384856 | -4.802383 | -0.813790 |
| H | -0.949998 | -5.702137 | -1.247740 |
| C | -4.548223 | -1.552879 | 1.530385 |
| C | -3.543365 | 2.146931 | -2.664783 |
| H | -4.458286 | 1.946315 | -3.220993 |
| C | -4.575158 | 0.776552 | 2.295409 |
| H | -5.102284 | 1.606871 | 2.763641 |
| C | -0.189752 | 3.828108 | 0.857758 |
| C | -4.599901 | -2.890511 | -2.773151 |
| H | -5.420323 | -2.392540 | -3.285986 |
| C | -3.286033 | 0.929788 | 1.858154 |
| H | -2.778297 | 1.888342 | 1.977094 |
| C | 0.810728 | 5.076330 | 2.724996 |
| H | 1.702967 | 5.403520 | 3.258234 |
| C | 2.250013 | 3.839938 | 1.141303 |
| H | 3.134855 | 4.187724 | 1.675032 |
| C | 1.423237 | -1.134172 | 4.032943 |
| H | 1.821754 | -0.630488 | 4.922617 |
| H | 2.274027 | -1.558269 | 3.484448 |
| H | 0.798339 | -1.963388 | 4.384351 |
| C | -2.085053 | 6.091712 | -2.425733 |
| H | -1.693507 | 7.106228 | -2.388341 |
| C | -4.136839 | -2.388443 | -1.578254 |
| H | -4.599456 | -1.501487 | -1.148016 |
| C | -1.413406 | 5.080558 | -1.778901 |
| H | -0.495095 | 5.307924 | -1.242525 |
| C | 2.371740 | 2.959271 | 0.097191 |
| H | 3.352933 | 2.600757 | -0.211309 |
| C | -4.025895 | -4.050476 | -3.333578 |
| H | -4.404865 | -4.441836 | -4.275090 |
| C | -6.486514 | -2.962372 | 1.952302 |
| H | -6.971462 | -3.934148 | 1.886365 |
| C | 1.523132 | 1.049409 | 2.799580 |
| H | 0.999779 | 1.735195 | 2.124945 |
| H | 2.444035 | 0.728382 | 2.289563 |
| H | 1.814988 | 1.625240 | 3.688054 |
| C | 0.474061 | -2.534736 | -2.544662 |
| H | 0.267062 | -3.116410 | -3.453027 |
| H | -0.380396 | -2.668940 | -1.871688 |
| H | 1.356709 | -2.978023 | -2.062690 |
| C | -0.439487 | 5.433113 | 3.170370 |
| H | -0.548365 | 6.050270 | 4.059554 |
| C | -3.271557 | 5.826789 | -3.139357 |
| H | -3.795818 | 6.637257 | -3.640819 |
| C | -0.499534 | -0.575589 | -3.735054 |
| H | -0.425357 | -1.026867 | -4.732902 |
| H | -0.521627 | 0.513525 | -3.865384 |

| | | | |
|---|---|---|---|
| H | -1.463685 | -0.887032 | -3.315618 |
| C | -7.165579 | -1.881894 | 2.551938 |
| H | -8.172771 | -2.021299 | 2.938791 |
| C | 0.676169 | -1.062746 | -2.886518 |
| C | -0.844471 | -4.247418 | 0.319189 |
| H | 0.024908 | -4.702228 | 0.794561 |
| C | -0.572457 | 0.392492 | 3.975577 |
| H | -1.318050 | -0.385295 | 4.181287 |
| H | -1.077062 | 1.214215 | 3.452073 |
| H | -0.233128 | 0.785473 | 4.942736 |
| C | 1.980148 | -0.870237 | -3.657598 |
| H | 2.849928 | -1.198312 | -3.076902 |
| H | 2.141175 | 0.170130 | -3.964264 |
| H | 1.955610 | -1.479263 | -4.569820 |
| Ni | 1.726417 | -1.331327 | 0.247123 |
| H | 3.892920 | -0.770379 | 1.413959 |
| C | 4.475397 | -1.032428 | 0.518077 |
| C | 3.533572 | -1.800988 | -0.422380 |
| N | 5.554427 | -1.856558 | 1.051073 |
| H | 5.379521 | -2.298249 | 1.946807 |
| C | 6.679014 | -2.382458 | 0.429003 |
| C | 7.006329 | -1.895544 | -0.957379 |
| H | 6.141922 | -1.912869 | -1.631577 |
| H | 7.371907 | -0.861916 | -0.931674 |
| H | 7.791186 | -2.533672 | -1.366483 |
| O | 7.371161 | -3.179958 | 1.034471 |
| C | 4.959979 | 0.275117 | -0.074582 |
| C | 5.577663 | 1.190961 | 0.784766 |
| C | 4.852242 | 0.596976 | -1.427701 |
| C | 6.075817 | 2.395545 | 0.301667 |
| H | 5.679577 | 0.941245 | 1.841188 |
| C | 5.352453 | 1.803196 | -1.917382 |
| H | 4.395255 | -0.104473 | -2.124659 |
| C | 5.965781 | 2.705725 | -1.054277 |
| H | 6.555073 | 3.094066 | 0.984640 |
| H | 5.268833 | 2.029838 | -2.978898 |
| H | 6.360328 | 3.645041 | -1.435029 |
| N | 3.041852 | -3.021292 | 0.121326 |
| O | 3.016761 | -4.107646 | -0.419361 |
| O | 2.419199 | -2.781472 | 1.271740 |
| H | 3.881383 | -1.997897 | -1.436277 |

**VII$_{S3}$**

| | | | |
|---|---|---|---|
| P | -0.478373 | 0.158949 | 1.376223 |
| P | 0.040347 | -0.925282 | -1.450160 |
| C | 2.793602 | -0.202344 | -1.208971 |
| C | 2.671920 | -2.510949 | -0.187042 |
| C | 5.326387 | -2.974263 | -1.310743 |
| H | 4.784685 | -3.790729 | -0.839604 |
| C | 1.508394 | -3.077475 | -0.699068 |
| C | 0.398598 | 3.167742 | 0.192326 |
| C | 1.613558 | 2.845446 | 0.994029 |
| C | -0.499414 | -0.121981 | -3.054056 |
| C | 3.202279 | -3.009022 | 1.048190 |
| C | 2.034264 | 1.539863 | 1.233284 |
| C | 2.379923 | 3.938113 | 1.529604 |
| C | 3.267577 | 1.304238 | 1.889219 |
| H | 3.596499 | 0.271729 | 2.015254 |
| C | 1.388197 | 0.162084 | -0.782950 |
| H | 1.162274 | 1.144203 | -1.222925 |
| C | -0.872032 | 2.798665 | 0.622515 |
| C | -1.042256 | 1.865634 | 1.782795 |
| H | -2.094730 | 1.814645 | 2.093247 |
| H | -0.463698 | 2.195361 | 2.659195 |
| C | 5.434807 | -0.667587 | -2.101885 |
| C | 3.382621 | -1.437571 | -0.940255 |
| C | 2.587772 | -4.141223 | 1.675570 |
| C | -0.645178 | 4.405273 | -1.686349 |
| C | 1.272534 | 0.328285 | 0.750276 |
| H | 1.752997 | -0.560338 | 1.187618 |
| C | 6.741761 | -0.926229 | -2.578705 |
| H | 7.278801 | -0.121982 | -3.080064 |
| C | 4.381199 | 4.748453 | 2.711483 |
| H | 5.323758 | 4.524346 | 3.209942 |
| C | 0.861571 | -2.495104 | -1.917635 |
| H | 0.102741 | -3.170461 | -2.329998 |
| H | 1.595011 | -2.287572 | -2.709220 |
| C | 3.617154 | 3.672195 | 2.200432 |
| C | 3.101792 | -4.617607 | 2.905268 |
| H | 2.625128 | -5.481853 | 3.367088 |
| C | 1.790357 | 4.204243 | -1.606277 |
| H | 2.694258 | 3.861203 | -1.106111 |
| C | 1.884834 | 4.890450 | -2.795268 |
| H | 2.863114 | 5.074929 | -3.234678 |
| C | 1.450110 | -4.730025 | 1.075457 |
| H | 0.993365 | -5.599441 | 1.547091 |
| C | 4.706968 | -1.703132 | -1.431187 |
| C | 4.047515 | 2.333611 | 2.341743 |

| | | | | | | | |
|---|---|---|---|---|---|---|---|
| H | 5.001389 | 2.133271 | 2.828109 | C | 7.315890 | -2.165024 | -2.427676 |
| C | 4.821584 | 0.591034 | -2.290615 | H | 8.319337 | -2.354698 | -2.802603 |
| H | 5.380534 | 1.382688 | -2.787884 | C | -0.270450 | -0.691833 | 3.047976 |
| C | 0.529525 | 3.931287 | -1.015485 | C | 0.905885 | -4.187164 | -0.060492 |
| C | 4.765696 | -2.874631 | 2.909180 | H | 0.016767 | -4.633084 | -0.507894 |
| H | 5.606694 | -2.384137 | 3.395264 | C | 0.720967 | 0.219217 | -3.912830 |
| C | 3.535669 | 0.804646 | -1.873133 | H | 1.343268 | -0.653331 | -4.143590 |
| H | 3.061531 | 1.773487 | -2.037293 | H | 1.360048 | 0.979200 | -3.447163 |
| C | -0.511065 | 5.130550 | -2.894557 | H | 0.373684 | 0.632249 | -4.868870 |
| H | -1.414320 | 5.488375 | -3.387954 | C | -1.526308 | -0.535958 | 3.906965 |
| C | -1.914493 | 4.113059 | -1.135037 | H | -2.371299 | -1.106558 | 3.510166 |
| H | -2.807067 | 4.514809 | -1.614085 | H | -1.821735 | 0.511358 | 4.049362 |
| C | -1.418024 | -1.075687 | -3.820277 | H | -1.318638 | -0.943356 | 4.904450 |
| H | -1.745268 | -0.586654 | -4.746493 | Ni | -1.597235 | -1.050640 | 0.017723 |
| H | -2.325048 | -1.357261 | -3.270134 | H | -3.392767 | 1.117008 | -0.076896 |
| H | -0.897437 | -1.996223 | -4.114153 | C | -4.106250 | 0.284132 | 0.067396 |
| C | 2.709314 | 6.310464 | 1.964194 | C | -3.309749 | -0.660389 | 0.957616 |
| H | 2.351224 | 7.335328 | 1.891229 | N | -4.299881 | -0.220713 | -1.297037 |
| C | 4.303183 | -2.399126 | 1.703011 | H | -3.410583 | -0.442961 | -1.737326 |
| H | 4.791588 | -1.545653 | 1.235616 | C | -5.257407 | -1.069344 | -1.834827 |
| C | 1.950602 | 5.287750 | 1.445002 | C | -6.680119 | -0.968494 | -1.370814 |
| H | 0.998263 | 5.517162 | 0.972742 | H | -7.256201 | -1.750576 | -1.868439 |
| C | -2.021150 | 3.309525 | -0.028802 | H | -6.776443 | -1.076006 | -0.288449 |
| H | -3.003040 | 3.092791 | 0.392825 | H | -7.099229 | 0.007726 | -1.640971 |
| C | 4.165262 | -3.997021 | 3.515882 | O | -4.920500 | -1.795743 | -2.765096 |
| H | 4.544998 | -4.368170 | 4.465197 | C | -5.338975 | 0.906944 | 0.683457 |
| C | 6.593495 | -3.197981 | -1.796546 | C | -5.874514 | 0.471125 | 1.895732 |
| H | 7.040403 | -4.184911 | -1.696145 | C | -5.943823 | 1.985640 | 0.026970 |
| C | -1.231256 | 1.172171 | -2.708551 | C | -7.004301 | 1.083816 | 2.432297 |
| H | -0.610885 | 1.840134 | -2.101685 | H | -5.423685 | -0.360666 | 2.434904 |
| H | -2.164660 | 1.005403 | -2.156217 | C | -7.071893 | 2.597258 | 0.560405 |
| H | -1.489948 | 1.713573 | -3.628190 | H | -5.528740 | 2.325977 | -0.923559 |
| C | -0.003990 | -2.167122 | 2.768159 | C | -7.608124 | 2.144145 | 1.765243 |
| H | 0.231836 | -2.693921 | 3.702855 | H | -7.414767 | 0.724507 | 3.373572 |
| H | 0.853704 | -2.297738 | 2.099041 | H | -7.535952 | 3.429927 | 0.035394 |
| H | -0.874766 | -2.655544 | 2.313638 | H | -8.492944 | 2.620204 | 2.182572 |
| C | 0.726370 | 5.364428 | -3.444900 | C | -2.739020 | -3.702663 | -1.105806 |
| H | 0.815417 | 5.913536 | -4.379771 | O | -2.357774 | -2.523116 | -1.002825 |
| C | 3.942064 | 6.044414 | 2.593583 | O | -2.952353 | -4.505822 | -0.089670 |
| H | 4.535796 | 6.863960 | 2.992826 | C | -2.983963 | -4.315004 | -2.435185 |
| C | 0.925468 | -0.097941 | 3.799085 | H | -3.832755 | -3.788362 | -2.889509 |
| H | 0.933448 | -0.512008 | 4.815503 | H | -2.125350 | -4.151301 | -3.094237 |
| H | 0.891001 | 0.994521 | 3.892438 | H | -3.203328 | -5.379958 | -2.353268 |
| H | 1.882354 | -0.368880 | 3.339977 | H | -2.952244 | -4.013662 | 0.775745 |

| | | | |
|---|---|---|---|
| N | -3.886253 | -1.963475 | 1.203238 |
| O | -3.254147 | -2.735321 | 1.974315 |
| O | -4.870065 | -2.337934 | 0.571431 |
| H | -3.121254 | -0.215915 | 1.936176 |

**TSIII$_{S3}$**

| | | | |
|---|---|---|---|
| P | -0.371191 | -0.067525 | 1.397213 |
| P | 0.050524 | -0.871435 | -1.539778 |
| C | 2.771804 | -0.115817 | -1.343697 |
| C | 2.741397 | -2.448726 | -0.377504 |
| C | 5.376445 | -2.809338 | -1.589743 |
| H | 4.885869 | -3.642381 | -1.092484 |
| C | 1.573635 | -3.019461 | -0.876216 |
| C | 0.397729 | 3.047663 | 0.358233 |
| C | 1.642629 | 2.716676 | 1.107956 |
| C | -0.545685 | -0.060456 | -3.123075 |
| C | 3.295190 | -2.957280 | 0.842428 |
| C | 2.076160 | 1.403539 | 1.290869 |
| C | 2.397178 | 3.791010 | 1.693481 |
| C | 3.289395 | 1.146469 | 1.974133 |
| H | 3.609364 | 0.110191 | 2.087078 |
| C | 1.387757 | 0.193329 | -0.822309 |
| H | 1.117735 | 1.205404 | -1.154736 |
| C | -0.836771 | 2.585628 | 0.802460 |
| C | -0.913781 | 1.607892 | 1.929531 |
| H | -1.930387 | 1.547399 | 2.337500 |
| H | -0.249686 | 1.893823 | 2.759060 |
| C | 5.366888 | -0.500551 | -2.386724 |
| C | 3.406082 | -1.339836 | -1.124241 |
| C | 2.679083 | -4.081735 | 1.482730 |
| C | -0.779092 | 4.346024 | -1.392800 |
| C | 1.342562 | 0.207951 | 0.724963 |
| H | 1.872431 | -0.695161 | 1.053053 |
| C | 6.657914 | -0.715661 | -2.925620 |
| H | 7.143370 | 0.104963 | -3.452408 |
| C | 4.384735 | 4.564229 | 2.925003 |
| H | 5.323297 | 4.324589 | 3.423736 |
| C | 0.887211 | -2.414414 | -2.059605 |
| H | 0.129704 | -3.084215 | -2.482183 |
| H | 1.602227 | -2.153667 | -2.850502 |
| C | 3.629820 | 3.505131 | 2.366180 |
| C | 3.202679 | -4.556146 | 2.709569 |
| H | 2.722537 | -5.412629 | 3.181994 |
| C | 1.666135 | 4.265603 | -1.414415 |
| H | 2.603824 | 3.926314 | -0.977092 |
| C | 1.679717 | 5.036940 | -2.554782 |
| H | 2.629390 | 5.299128 | -3.017227 |
| C | 1.546901 | -4.683372 | 0.885493 |
| H | 1.098784 | -5.554991 | 1.361352 |
| C | 4.709821 | -1.560270 | -1.680714 |
| C | 4.056579 | 2.163082 | 2.477092 |
| H | 4.996729 | 1.947728 | 2.983609 |
| C | 4.701231 | 0.734611 | -2.550151 |
| H | 5.204906 | 1.539428 | -3.083844 |
| C | 0.444221 | 3.893453 | -0.797419 |
| C | 4.896107 | -2.842141 | 2.673223 |
| H | 5.755510 | -2.364948 | 3.140213 |
| C | 3.435301 | 0.912584 | -2.055913 |
| H | 2.918096 | 1.863889 | -2.197184 |
| C | -0.727033 | 5.163585 | -2.547305 |
| H | -1.665261 | 5.507912 | -2.981404 |
| C | -2.011755 | 3.926976 | -0.839897 |
| H | -2.942149 | 4.290770 | -1.275385 |
| C | -1.804837 | -0.774701 | -3.614272 |
| H | -2.072243 | -0.381424 | -4.603118 |
| H | -2.657585 | -0.587293 | -2.949877 |
| H | -1.681275 | -1.859017 | -3.705510 |
| C | 2.708384 | 6.145195 | 2.230752 |
| H | 2.343484 | 7.169747 | 2.198987 |
| C | 4.418245 | -2.362438 | 1.474249 |
| H | 4.907055 | -1.514136 | 0.997812 |
| C | 1.958927 | 5.140436 | 1.665613 |
| H | 1.007217 | 5.384901 | 1.200803 |
| C | -2.036927 | 3.040427 | 0.206610 |
| H | -2.990449 | 2.704039 | 0.615569 |
| C | 4.285880 | -3.949078 | 3.298682 |
| H | 4.675470 | -4.320399 | 4.243863 |
| C | 6.627322 | -2.989043 | -2.132659 |
| H | 7.113748 | -3.958890 | -2.051466 |
| C | -0.879351 | 1.399388 | -2.836930 |
| H | 0.014212 | 2.013493 | -2.676734 |
| H | -1.537338 | 1.517200 | -1.964271 |
| H | -1.405467 | 1.827217 | -3.700404 |
| C | -0.570293 | -2.511239 | 2.717162 |
| H | -0.308022 | -3.132726 | 3.584391 |
| H | -0.019237 | -2.911679 | 1.857116 |
| H | -1.645274 | -2.642742 | 2.546008 |
| C | 0.474604 | 5.499842 | -3.122818 |

| | | | |
|---|---|---|---|
| H | 0.499530 | 6.118647 | -4.016993 |
| C | 3.939696 | 5.861496 | 2.855615 |
| H | 4.525113 | 6.667326 | 3.293024 |
| C | 1.202843 | -0.966732 | 3.575466 |
| H | 1.213558 | -1.476802 | 4.547598 |
| H | 1.534700 | 0.065547 | 3.742555 |
| H | 1.947062 | -1.472229 | 2.946935 |
| C | 7.282125 | -1.932545 | -2.798241 |
| H | 8.273235 | -2.087455 | -3.218883 |
| C | -0.215269 | -1.052953 | 3.008708 |
| C | 0.993671 | -4.146339 | -0.248686 |
| H | 0.105007 | -4.594237 | -0.694088 |
| C | 0.547999 | -0.143021 | -4.192505 |
| H | 0.690822 | -1.163491 | -4.564402 |
| H | 1.516034 | 0.237515 | -3.842078 |
| H | 0.245143 | 0.472819 | -5.049230 |
| C | -1.198031 | -0.495896 | 4.040478 |
| H | -2.231473 | -0.485185 | 3.677087 |
| H | -0.923998 | 0.506633 | 4.386563 |
| H | -1.190578 | -1.156108 | 4.916814 |
| Ni | -1.500400 | -1.204619 | -0.072304 |
| H | -3.198603 | 0.275949 | -0.711376 |
| C | -4.083765 | -0.192602 | -0.219422 |
| C | -3.454061 | -0.902063 | 0.986020 |
| N | -4.618962 | -1.078215 | -1.245873 |
| H | -3.899617 | -1.635746 | -1.695078 |
| C | -5.822355 | -1.781052 | -1.322488 |
| C | -7.095939 | -1.118525 | -0.888839 |
| H | -7.867304 | -1.884295 | -0.789470 |
| H | -7.010677 | -0.562087 | 0.045461 |
| H | -7.407553 | -0.411814 | -1.668598 |
| O | -5.805589 | -2.866206 | -1.887991 |
| C | -4.976624 | 0.989113 | 0.100511 |
| C | -5.309952 | 1.375898 | 1.399634 |
| C | -5.403269 | 1.783443 | -0.970940 |
| C | -6.072773 | 2.522275 | 1.621319 |
| H | -4.989988 | 0.790291 | 2.260553 |
| C | -6.161508 | 2.925392 | -0.750470 |
| H | -5.137681 | 1.483944 | -1.985960 |
| C | -6.500427 | 3.298619 | 0.550220 |
| H | -6.335159 | 2.801711 | 2.639388 |
| H | -6.490041 | 3.528035 | -1.595198 |
| H | -7.097048 | 4.191042 | 0.726136 |
| C | -2.720209 | -3.578224 | -0.662025 |
| O | -2.082490 | -2.623955 | -1.240724 |
| O | -3.106771 | -3.473291 | 0.538819 |
| C | -3.070830 | -4.783562 | -1.452904 |
| H | -4.065125 | -4.595835 | -1.881565 |
| H | -2.366118 | -4.946942 | -2.271659 |
| H | -3.141612 | -5.664875 | -0.812410 |
| H | -2.986631 | -2.227136 | 0.759554 |
| N | -4.448645 | -1.557141 | 1.916437 |
| O | -4.053639 | -1.806856 | 3.052432 |
| O | -5.557783 | -1.823935 | 1.477721 |
| H | -2.985784 | -0.174013 | 1.651691 |

**VIII$_{S3}$**

| | | | |
|---|---|---|---|
| P | 0.018468 | -0.490387 | 1.645209 |
| P | 0.193887 | -1.030602 | -1.357585 |
| C | 2.700218 | 0.279885 | -1.456818 |
| C | 3.312969 | -2.054266 | -0.704543 |
| C | 5.719420 | -1.775297 | -2.334448 |
| H | 5.492126 | -2.725964 | -1.858796 |
| C | 2.223649 | -2.849127 | -1.050069 |
| C | -0.054194 | 2.749025 | 0.792684 |
| C | 1.314464 | 2.659169 | 1.380100 |
| C | -0.723015 | -0.294357 | -2.808844 |
| C | 4.186981 | -2.491967 | 0.343707 |
| C | 2.061831 | 1.482004 | 1.374697 |
| C | 1.870965 | 3.837718 | 1.986309 |
| C | 3.390728 | 1.484053 | 1.864249 |
| H | 3.963228 | 0.556889 | 1.811731 |
| C | 1.377926 | 0.248001 | -0.729559 |
| H | 0.858100 | 1.192888 | -0.941121 |
| C | -1.108239 | 2.011246 | 1.320046 |
| C | -0.861055 | 0.957131 | 2.350392 |
| H | -1.801151 | 0.599789 | 2.788563 |
| H | -0.234961 | 1.331177 | 3.175361 |
| C | 5.118592 | 0.523661 | -2.895302 |
| C | 3.593836 | -0.790873 | -1.446668 |
| C | 3.994426 | -3.784260 | 0.931488 |
| C | -1.666358 | 3.854686 | -0.733253 |
| C | 1.539408 | 0.180445 | 0.810987 |
| H | 2.285437 | -0.601333 | 1.019812 |
| C | 6.326854 | 0.626127 | -3.626520 |
| H | 6.546783 | 1.564443 | -4.134664 |
| C | 3.773568 | 4.978340 | 3.055119 |
| H | 4.802978 | 4.940118 | 3.409638 |

| | | | |
|---|---|---|---|
| C | 1.233663 | -2.364609 | -2.063849 |
| H | 0.571585 | -3.171772 | -2.400022 |
| H | 1.741734 | -1.952766 | -2.946101 |
| C | 3.218092 | 3.813272 | 2.474677 |
| C | 4.851666 | -4.203790 | 1.977557 |
| H | 4.695530 | -5.190484 | 2.412511 |
| C | 0.721674 | 4.314764 | -0.991937 |
| H | 1.751913 | 4.174027 | -0.668326 |
| C | 0.445637 | 5.124436 | -2.070787 |
| H | 1.261000 | 5.615815 | -2.597825 |
| C | 2.922715 | -4.592709 | 0.483987 |
| H | 2.787141 | -5.580052 | 0.923925 |
| C | 4.808752 | -0.694649 | -2.207034 |
| C | 3.962782 | 2.616922 | 2.377520 |
| H | 4.992725 | 2.605139 | 2.731811 |
| C | 4.200712 | 1.596140 | -2.843102 |
| H | 4.440161 | 2.521972 | -3.364878 |
| C | -0.317086 | 3.648787 | -0.292494 |
| C | 6.035958 | -2.106506 | 1.877681 |
| H | 6.825953 | -1.458775 | 2.252793 |
| C | 3.020101 | 1.467575 | -2.159210 |
| H | 2.306045 | 2.293414 | -2.135080 |
| C | -1.916630 | 4.710466 | -1.832629 |
| H | -2.949543 | 4.852770 | -2.151280 |
| C | -2.717932 | 3.170773 | -0.081932 |
| H | -3.749009 | 3.353015 | -0.385526 |
| C | -1.859032 | -1.250752 | -3.172616 |
| H | -2.356951 | -0.893708 | -4.083358 |
| H | -2.613601 | -1.293599 | -2.378619 |
| H | -1.517610 | -2.275233 | -3.361456 |
| C | 1.693454 | 6.146480 | 2.737393 |
| H | 1.098167 | 7.048973 | 2.860096 |
| C | 5.231949 | -1.675532 | 0.847517 |
| H | 5.395765 | -0.694025 | 0.406198 |
| C | 1.129670 | 5.035990 | 2.153832 |
| H | 0.092840 | 5.073883 | 1.829387 |
| C | -2.434648 | 2.252900 | 0.895888 |
| H | -3.248253 | 1.721746 | 1.389613 |
| C | 5.849563 | -3.383545 | 2.446667 |
| H | 6.495696 | -3.715173 | 3.256588 |
| C | 6.880202 | -1.646507 | -3.060111 |
| H | 7.558299 | -2.493319 | -3.142570 |
| C | -1.301323 | 1.052909 | -2.388971 |
| H | -0.534158 | 1.831660 | -2.303633 |
| H | -1.842219 | 1.001220 | -1.432798 |
| H | -2.017682 | 1.399674 | -3.146331 |
| C | 1.103871 | -2.796194 | 2.644090 |
| H | 1.533621 | -3.371063 | 3.475385 |
| H | 1.893263 | -2.669196 | 1.895495 |
| H | 0.301069 | -3.394293 | 2.194977 |
| C | -0.883809 | 5.330327 | -2.494624 |
| H | -1.087502 | 5.977574 | -3.344845 |
| C | 3.031008 | 6.126418 | 3.181951 |
| H | 3.466127 | 7.015265 | 3.633816 |
| C | 1.741403 | -0.716672 | 3.849702 |
| H | 1.965772 | -1.243693 | 4.786248 |
| H | 1.502326 | 0.322522 | 4.108001 |
| H | 2.661816 | -0.713622 | 3.252808 |
| C | 7.196963 | -0.433170 | -3.703974 |
| H | 8.121606 | -0.344282 | -4.270175 |
| C | 0.600509 | -1.445865 | 3.138715 |
| C | 2.037860 | -4.118021 | -0.451188 |
| H | 1.187757 | -4.724426 | -0.765588 |
| C | 0.205182 | -0.105355 | -4.011713 |
| H | 0.511911 | -1.057856 | -4.457620 |
| H | 1.107716 | 0.468775 | -3.766779 |
| H | -0.336768 | 0.451618 | -4.787068 |
| C | -0.585703 | -1.624243 | 4.088968 |
| H | -1.455317 | -2.069461 | 3.592693 |
| H | -0.888454 | -0.678283 | 4.553260 |
| H | -0.291919 | -2.299727 | 4.902224 |
| Ni | -0.976962 | -1.796769 | 0.259896 |
| H | -3.331034 | -0.254683 | -0.066015 |
| C | -4.415202 | -0.487091 | 0.016001 |
| C | -4.575232 | -0.747287 | 1.531316 |
| N | -4.581693 | -1.679304 | -0.795111 |
| H | -3.754545 | -2.242238 | -0.957846 |
| C | -5.727161 | -2.355540 | -1.191712 |
| C | -7.048433 | -1.646050 | -1.106280 |
| H | -7.842371 | -2.392730 | -1.160334 |
| H | -7.182454 | -1.041444 | -0.205341 |
| H | -7.151707 | -0.964828 | -1.960272 |
| O | -5.603576 | -3.478235 | -1.658905 |
| C | -5.131456 | 0.727513 | -0.544480 |
| C | -5.909351 | 1.592093 | 0.227332 |
| C | -4.924770 | 1.028975 | -1.895998 |
| C | -6.463872 | 2.738570 | -0.343203 |
| H | -6.123091 | 1.363762 | 1.269484 |

| | | | |
|---|---|---|---|
| C | -5.468593 | 2.174878 | -2.461992 |
| H | -4.333824 | 0.343392 | -2.504415 |
| C | -6.241031 | 3.037664 | -1.683534 |
| H | -7.080724 | 3.393717 | 0.268180 |
| H | -5.295168 | 2.393381 | -3.514512 |
| H | -6.677040 | 3.931828 | -2.124666 |
| C | -2.382026 | -3.592147 | 0.438819 |
| O | -1.829715 | -3.248904 | -0.673072 |
| O | -2.097994 | -2.847278 | 1.444157 |
| C | -3.321585 | -4.726200 | 0.511947 |
| H | -4.171422 | -4.526275 | -0.160037 |
| H | -2.839324 | -5.636270 | 0.139782 |
| H | -3.684160 | -4.876003 | 1.529940 |
| H | -3.789460 | -1.429407 | 1.864348 |
| N | -5.862077 | -1.429225 | 1.881672 |
| O | -5.884170 | -2.648445 | 1.745693 |
| O | -6.804696 | -0.730215 | 2.243505 |
| H | -4.546470 | 0.183654 | 2.103183 |

**IV$_{S1}$**

| | | | |
|---|---|---|---|
| P | 0.151170 | -1.074219 | -1.496531 |
| P | -0.571745 | -0.267304 | 1.403049 |
| C | 1.583380 | 1.643303 | 1.344824 |
| C | -0.359350 | 2.907244 | 0.340945 |
| C | 0.702781 | 5.285025 | 1.642061 |
| H | -0.259007 | 5.324059 | 1.136515 |
| C | -1.494486 | 2.242182 | 0.786347 |
| C | 3.179875 | -1.961192 | -0.222408 |
| C | 3.539700 | -0.751908 | -1.018013 |
| C | -0.235207 | -1.137540 | 3.030919 |
| C | -0.439734 | 3.702623 | -0.848608 |
| C | 2.640610 | 0.291052 | -1.226560 |
| C | 4.847937 | -0.679474 | -1.608345 |
| C | 3.054733 | 1.449273 | -1.930137 |
| H | 2.333593 | 2.258011 | -2.061133 |
| C | 1.109802 | 0.312837 | 0.804880 |
| H | 1.785372 | -0.460291 | 1.199440 |
| C | 2.162379 | -2.813499 | -0.643608 |
| C | 1.320084 | -2.460215 | -1.831977 |
| H | 0.719258 | -3.318949 | -2.155072 |
| H | 1.959353 | -2.161516 | -2.673673 |
| C | 2.642524 | 4.033947 | 2.433782 |
| C | 0.904028 | 2.841133 | 1.127734 |
| C | -1.700425 | 3.869035 | -1.509723 |
| C | 3.676807 | -3.526558 | 1.632198 |
| C | 1.212391 | 0.261012 | -0.738000 |
| H | 0.727580 | 1.178552 | -1.097220 |
| C | 3.141916 | 5.230800 | 3.000942 |
| H | 4.089947 | 5.193619 | 3.536808 |
| C | 6.529079 | 0.573375 | -2.899394 |
| H | 6.806627 | 1.482828 | -3.431613 |
| C | -1.419957 | 1.280039 | 1.925512 |
| H | -2.421314 | 1.008036 | 2.280058 |
| H | -0.860265 | 1.704165 | 2.770423 |
| C | 5.239395 | 0.499807 | -2.321029 |
| C | -1.765467 | 4.624214 | -2.704392 |
| H | -2.732245 | 4.738944 | -3.193614 |
| C | 4.875947 | -1.398864 | 1.526815 |
| H | 5.074133 | -0.448887 | 1.033702 |
| C | 5.550645 | -1.721638 | 2.681700 |
| H | 6.275286 | -1.023178 | 3.095636 |
| C | -2.851767 | 3.265581 | -0.951264 |
| H | -3.820663 | 3.446246 | -1.420451 |
| C | 1.404382 | 4.053204 | 1.714606 |
| C | 4.317712 | 1.562867 | -2.447685 |
| H | 4.619782 | 2.464107 | -2.980351 |
| C | 3.333238 | 2.809105 | 2.572122 |
| H | 4.282836 | 2.794480 | 3.106267 |
| C | 3.917445 | -2.281899 | 0.964737 |
| C | 0.609344 | 5.020407 | -2.605051 |
| H | 1.503484 | 5.465405 | -3.037562 |
| C | 2.807463 | 1.653556 | 2.058622 |
| H | 3.340143 | 0.709775 | 2.184150 |
| C | 4.399931 | -3.834653 | 2.809539 |
| H | 4.208306 | -4.788270 | 3.301398 |
| C | 2.705236 | -4.409091 | 1.105990 |
| H | 2.546220 | -5.369727 | 1.595904 |
| C | -1.565350 | -1.382279 | 3.744724 |
| H | -1.374518 | -1.918470 | 4.683730 |
| H | -2.240801 | -1.997960 | 3.139351 |
| H | -2.079274 | -0.447662 | 4.006947 |
| C | 7.016466 | -1.655076 | -2.132866 |
| H | 7.703191 | -2.497520 | -2.077914 |
| C | 0.703004 | 4.306724 | -1.431964 |
| H | 1.667575 | 4.194995 | -0.940022 |
| C | 5.774644 | -1.752559 | -1.549142 |
| H | 5.493333 | -2.673696 | -1.044127 |
| C | 1.950584 | -4.045365 | 0.020560 |

| | | | |
|---|---|---|---|
| H | 1.178701 | -4.710032 | -0.361893 |
| C | -0.634656 | 5.185537 | -3.247807 |
| H | -0.695329 | 5.755784 | -4.172171 |
| C | 1.209312 | 6.428840 | 2.213202 |
| H | 0.646159 | 7.357651 | 2.145930 |
| C | 0.445442 | -2.464729 | 2.718462 |
| H | 1.399558 | -2.312306 | 2.201829 |
| H | -0.175172 | -3.105891 | 2.086077 |
| H | 0.668182 | -3.001700 | 3.650905 |
| C | -0.934225 | 0.959023 | -3.007142 |
| H | -1.490651 | 1.241807 | -3.908840 |
| H | -0.182749 | 1.739578 | -2.832450 |
| H | -1.650191 | 0.985127 | -2.171483 |
| C | 5.316916 | -2.952427 | 3.328955 |
| H | 5.863213 | -3.200355 | 4.236666 |
| C | 7.405847 | -0.479496 | -2.806501 |
| H | 8.393347 | -0.413704 | -3.258496 |
| C | 0.904457 | -0.291719 | -4.143175 |
| H | 0.571892 | 0.210258 | -5.061820 |
| H | 1.302865 | -1.267691 | -4.443921 |
| H | 1.726201 | 0.300814 | -3.726596 |
| C | 2.445394 | 6.409419 | 2.890012 |
| H | 2.837792 | 7.323423 | 3.330849 |
| C | -0.291694 | -0.416260 | -3.197880 |
| C | -2.739439 | 2.459425 | 0.153472 |
| H | -3.621543 | 2.009107 | 0.610705 |
| C | 0.693961 | -0.316649 | 3.927113 |
| H | 0.326769 | 0.694503 | 4.140107 |
| H | 1.700495 | -0.229243 | 3.502158 |
| H | 0.798449 | -0.831685 | 4.891332 |
| C | -1.298745 | -1.387375 | -3.815886 |
| H | -2.222335 | -1.436631 | -3.235835 |
| H | -0.895966 | -2.405605 | -3.896694 |
| H | -1.556155 | -1.049774 | -4.828573 |
| Ni | -1.488806 | -1.416695 | -0.073746 |
| H | -2.451492 | -1.263243 | 1.075174 |
| C | -4.977532 | -0.599727 | -0.230490 |
| C | -4.889767 | -1.967647 | -0.117374 |
| H | -5.510116 | -2.533224 | 0.566267 |
| N | -3.962777 | -2.747233 | -0.800544 |
| O | -4.148826 | -3.966936 | -0.899976 |
| O | -2.903912 | -2.205414 | -1.276489 |
| N | -4.545204 | 0.107710 | -1.321021 |
| H | -4.228753 | 1.060937 | -1.152393 |
| C | -4.531606 | -0.189699 | -2.704578 |
| C | -5.161575 | -1.459566 | -3.186350 |
| H | -4.402114 | -2.243876 | -3.287886 |
| H | -5.950328 | -1.837880 | -2.529591 |
| H | -5.568640 | -1.268955 | -4.181592 |
| O | -4.067280 | 0.653954 | -3.444095 |
| C | -5.526703 | 0.185859 | 0.871560 |
| C | -5.243873 | -0.194203 | 2.194104 |
| C | -6.251135 | 1.365107 | 0.631882 |
| C | -5.675110 | 0.593709 | 3.253363 |
| H | -4.639006 | -1.084443 | 2.369963 |
| C | -6.689784 | 2.141724 | 1.695229 |
| H | -6.483764 | 1.652762 | -0.393029 |
| C | -6.397975 | 1.760171 | 3.005393 |
| H | -5.435972 | 0.305934 | 4.275048 |
| H | -7.261909 | 3.046850 | 1.505208 |
| H | -6.732657 | 2.377300 | 3.836397 |
| C | -1.871431 | -4.549185 | 0.542403 |
| O | -2.632848 | -4.171934 | 1.592712 |
| H | -3.184516 | -4.916858 | 1.882788 |
| O | -1.143894 | -3.726624 | 0.018118 |
| C | -1.954556 | -5.985200 | 0.131432 |
| H | -1.638184 | -6.641196 | 0.951346 |
| H | -2.983474 | -6.247573 | -0.136372 |
| H | -1.314836 | -6.163277 | -0.733533 |

**$V_{S1}$**

| | | | |
|---|---|---|---|
| P | -0.025381 | -1.178580 | -1.474235 |
| P | -0.741618 | -0.275107 | 1.394644 |
| C | 1.414566 | 1.606387 | 1.279740 |
| C | -0.507189 | 2.828055 | 0.185854 |
| C | 0.500554 | 5.245101 | 1.466066 |
| H | -0.451195 | 5.262875 | 0.940017 |
| C | -1.662089 | 2.179953 | 0.603022 |
| C | 2.883514 | -2.176612 | -0.096028 |
| C | 3.340045 | -1.025426 | -0.927554 |
| C | -0.452332 | -1.062045 | 3.066614 |
| C | -0.532244 | 3.572866 | -1.038334 |
| C | 2.516803 | 0.063645 | -1.203778 |
| C | 4.673986 | -1.048548 | -1.461051 |
| C | 3.025797 | 1.171780 | -1.924699 |
| H | 2.362686 | 2.020654 | -2.103755 |
| C | 0.944773 | 0.258851 | 0.781955 |
| H | 1.603912 | -0.506901 | 1.217180 |

| | | | |
|---|---|---|---|
| C | 1.836580 | -2.993275 | -0.513453 |
| C | 1.056777 | -2.640776 | -1.741340 |
| H | 0.412773 | -3.471598 | -2.054744 |
| H | 1.733108 | -2.407963 | -2.575241 |
| C | 2.437496 | 4.029667 | 2.321151 |
| C | 0.728711 | 2.791830 | 1.019697 |
| C | -1.761097 | 3.707408 | -1.765046 |
| C | 3.210524 | -3.663497 | 1.856972 |
| C | 1.076398 | 0.135760 | -0.756943 |
| H | 0.647992 | 1.058106 | -1.175140 |
| C | 2.916586 | 5.243720 | 2.868481 |
| H | 3.855625 | 5.228071 | 3.420870 |
| C | 6.482544 | 0.064657 | -2.706530 |
| H | 6.836695 | 0.938645 | -3.252332 |
| C | -1.638825 | 1.278060 | 1.793875 |
| H | -2.654419 | 1.003078 | 2.108673 |
| H | -1.132312 | 1.743801 | 2.652598 |
| C | 5.164427 | 0.082670 | -2.190826 |
| C | -1.770884 | 4.420410 | -2.987556 |
| H | -2.714204 | 4.516926 | -3.524268 |
| C | 4.524506 | -1.605719 | 1.698531 |
| H | 4.801316 | -0.701537 | 1.158953 |
| C | 5.118357 | -1.891107 | 2.906032 |
| H | 5.856499 | -1.206585 | 3.319398 |
| C | -2.932038 | 3.099416 | -1.255493 |
| H | -3.871103 | 3.219587 | -1.796060 |
| C | 1.210142 | 4.021825 | 1.582785 |
| C | 4.313455 | 1.192924 | -2.390861 |
| H | 4.692388 | 2.058034 | -2.934016 |
| C | 3.139597 | 2.815874 | 2.495643 |
| H | 4.082834 | 2.824054 | 3.040885 |
| C | 3.549982 | -2.471636 | 1.137569 |
| C | 0.598607 | 4.816315 | -2.797746 |
| H | 1.512931 | 5.240183 | -3.208366 |
| C | 2.631095 | 1.642723 | 2.004941 |
| H | 3.168865 | 0.706293 | 2.160388 |
| C | 3.852514 | -3.934453 | 3.089666 |
| H | 3.584519 | -4.845023 | 3.625120 |
| C | 2.212311 | -4.520098 | 1.334937 |
| H | 1.969559 | -5.436395 | 1.872325 |
| C | -1.756019 | -1.037398 | 3.863073 |
| H | -1.622924 | -1.618218 | 4.784595 |
| H | -2.585966 | -1.488140 | 3.304540 |
| H | -2.049820 | -0.022365 | 4.157252 |
| C | 6.806577 | -2.158581 | -1.843312 |
| H | 7.442191 | -3.033986 | -1.726021 |
| C | 0.638518 | 4.147353 | -1.595569 |
| H | 1.582117 | 4.049000 | -1.061385 |
| C | 5.534773 | -2.168082 | -1.320296 |
| H | 5.177985 | -3.053014 | -0.798357 |
| C | 1.517877 | -4.170912 | 0.204074 |
| H | 0.714723 | -4.807735 | -0.167938 |
| C | -0.616137 | 4.963064 | -3.498534 |
| H | -0.635167 | 5.502614 | -4.442939 |
| C | 0.987009 | 6.406054 | 2.020088 |
| H | 0.418326 | 7.328549 | 1.921000 |
| C | -0.010137 | -2.497305 | 2.818776 |
| H | 0.896902 | -2.524104 | 2.207324 |
| H | -0.784170 | -3.077082 | 2.297899 |
| H | 0.220591 | -2.998990 | 3.768522 |
| C | -1.157164 | 0.737339 | -3.074645 |
| H | -1.670429 | 0.989847 | -4.011173 |
| H | -0.443783 | 1.544862 | -2.869040 |
| H | -1.917990 | 0.746612 | -2.282750 |
| C | 4.783127 | -3.066606 | 3.609669 |
| H | 5.263266 | -3.284112 | 4.561478 |
| C | 7.293039 | -1.030351 | -2.534464 |
| H | 8.303596 | -1.034702 | -2.937548 |
| C | 0.754558 | -0.456807 | -4.128842 |
| H | 0.405931 | -0.088751 | -5.103134 |
| H | 1.272535 | -1.404544 | -4.314921 |
| H | 1.487238 | 0.264553 | -3.750863 |
| C | 2.210856 | 6.412468 | 2.719457 |
| H | 2.587228 | 7.339525 | 3.146461 |
| C | -0.451808 | -0.610023 | -3.203302 |
| C | -2.871849 | 2.342624 | -0.112186 |
| H | -3.778183 | 1.902241 | 0.309245 |
| C | 0.638946 | -0.326766 | 3.847307 |
| H | 0.468417 | 0.755176 | 3.920458 |
| H | 1.632834 | -0.485110 | 3.410483 |
| H | 0.673932 | -0.723748 | 4.870023 |
| C | -1.394072 | -1.672487 | -3.771575 |
| H | -2.213902 | -1.890811 | -3.080951 |
| H | -0.872558 | -2.617328 | -3.969637 |
| H | -1.829777 | -1.323792 | -4.716656 |
| Ni | -1.636367 | -1.515321 | -0.028811 |
| H | -2.439548 | -1.668084 | 1.232144 |
| C | -4.624213 | -1.455924 | -0.046428 |

| | | | |
|---|---|---|---|
| C | -4.547147 | -2.827339 | -0.001605 |
| H | -5.227049 | -3.438297 | 0.578886 |
| N | -3.559525 | -3.574977 | -0.649212 |
| O | -3.759123 | -4.754379 | -0.915196 |
| O | -2.413596 | -3.037444 | -0.908871 |
| N | -4.131827 | -0.724979 | -1.116848 |
| H | -3.926111 | 0.255172 | -0.920970 |
| C | -4.438402 | -0.873942 | -2.501841 |
| C | -4.966164 | -2.187112 | -2.985995 |
| H | -4.174918 | -2.945376 | -3.021651 |
| H | -5.765291 | -2.583357 | -2.350988 |
| H | -5.342372 | -2.041243 | -3.999718 |
| O | -4.260453 | 0.095932 | -3.207960 |
| C | -5.307664 | -0.712534 | 1.007587 |
| C | -5.285534 | -1.197056 | 2.326177 |
| C | -5.950427 | 0.507871 | 0.737784 |
| C | -5.883175 | -0.475728 | 3.349238 |
| H | -4.758187 | -2.126409 | 2.539019 |
| C | -6.547922 | 1.226337 | 1.764972 |
| H | -6.004874 | 0.877120 | -0.286903 |
| C | -6.511301 | 0.738775 | 3.070890 |
| H | -5.848257 | -0.852150 | 4.369191 |
| H | -7.051789 | 2.165052 | 1.546583 |
| H | -6.975711 | 1.306118 | 3.874404 |

**TSII$_{S1}$**

| | | | |
|---|---|---|---|
| P | 0.116157 | 0.928158 | -1.634591 |
| P | 0.911406 | 0.130835 | 1.229505 |
| C | -1.494053 | -1.407153 | 1.412915 |
| C | 0.143210 | -2.994785 | 0.320248 |
| C | -1.117862 | -5.116944 | 1.863459 |
| H | -0.210854 | -5.319762 | 1.298655 |
| C | 1.416537 | -2.510084 | 0.600158 |
| C | -2.495184 | 2.448139 | -0.183333 |
| C | -3.179146 | 1.316949 | -0.876798 |
| C | 0.838854 | 1.043477 | 2.863035 |
| C | -0.046509 | -3.831870 | -0.826349 |
| C | -2.543035 | 0.100961 | -1.123892 |
| C | -4.526510 | 1.506442 | -1.336278 |
| C | -3.250181 | -0.956821 | -1.746996 |
| H | -2.730987 | -1.904179 | -1.907005 |
| C | -0.869634 | -0.188206 | 0.772638 |
| H | -1.374769 | 0.698892 | 1.185315 |
| C | -1.380720 | 3.058168 | -0.752196 |
| C | -0.765609 | 2.492778 | -1.994216 |
| H | -0.037998 | 3.185126 | -2.432723 |
| H | -1.530672 | 2.274883 | -2.751733 |
| C | -2.801625 | -3.559082 | 2.699536 |
| C | -1.006696 | -2.698275 | 1.221690 |
| C | 1.086858 | -4.232319 | -1.607532 |
| C | -2.441471 | 4.140074 | 1.622525 |
| C | -1.101852 | -0.161742 | -0.757595 |
| H | -0.843098 | -1.163274 | -1.128510 |
| C | -3.425631 | -4.641083 | 3.364621 |
| H | -4.320585 | -4.440371 | 3.952604 |
| C | -6.547705 | 0.610869 | -2.419041 |
| H | -7.057806 | -0.227824 | -2.891632 |
| C | 1.619086 | -1.507624 | 1.691635 |
| H | 2.686370 | -1.361261 | 1.911361 |
| H | 1.133861 | -1.818061 | 2.629310 |
| C | -5.219770 | 0.423112 | -1.967205 |
| C | 0.889298 | -5.029474 | -2.761193 |
| H | 1.762122 | -5.326974 | -3.341205 |
| C | -4.053305 | 2.305118 | 1.764374 |
| H | -4.501367 | 1.406563 | 1.342841 |
| C | -4.507771 | 2.796426 | 2.967062 |
| H | -5.306485 | 2.278581 | 3.494304 |
| C | 2.374460 | -3.790635 | -1.230115 |
| H | 3.238876 | -4.097094 | -1.816411 |
| C | -1.629570 | -3.794225 | 1.910402 |
| C | -4.553120 | -0.810053 | -2.143392 |
| H | -5.085169 | -1.635913 | -2.614036 |
| C | -3.307211 | -2.243612 | 2.801008 |
| H | -4.211329 | -2.067774 | 3.382680 |
| C | -3.008283 | 2.952510 | 1.055842 |
| C | -1.493242 | -4.994846 | -2.399498 |
| H | -2.492403 | -5.286785 | -2.717141 |
| C | -2.659886 | -1.202177 | 2.191776 |
| H | -3.046605 | -0.186680 | 2.288375 |
| C | -2.944142 | 4.627356 | 2.853005 |
| H | -2.506606 | 5.534376 | 3.269611 |
| C | -1.364834 | 4.774976 | 0.958993 |
| H | -0.945464 | 5.685491 | 1.385986 |
| C | 2.191476 | 0.910158 | 3.557609 |
| H | 2.199999 | 1.538797 | 4.457171 |
| H | 3.019102 | 1.244940 | 2.921345 |
| H | 2.401155 | -0.117651 | 3.878078 |
| C | -6.489711 | 2.902670 | -1.684574 |

| | | | |
|---|---|---|---|
| H | -6.980723 | 3.869274 | -1.592136 |
| C | -1.334318 | -4.240018 | -1.260071 |
| H | -2.206761 | -3.941856 | -0.681448 |
| C | -5.202423 | 2.749037 | -1.225773 |
| H | -4.689153 | 3.598624 | -0.782103 |
| C | -0.823234 | 4.223664 | -0.174527 |
| H | 0.036756 | 4.685696 | -0.659382 |
| C | -0.373427 | -5.400790 | -3.155334 |
| H | -0.513411 | -6.003721 | -4.049894 |
| C | -1.741398 | -6.145478 | 2.530852 |
| H | -1.323850 | -7.149063 | 2.480921 |
| C | 0.535850 | 2.504453 | 2.558486 |
| H | -0.387272 | 2.600458 | 1.976835 |
| H | 1.339933 | 2.980549 | 1.981651 |
| H | 0.396719 | 3.068830 | 3.490603 |
| C | 0.929453 | -1.274560 | -3.067602 |
| H | 1.395635 | -1.670381 | -3.979433 |
| H | 0.097597 | -1.943785 | -2.816429 |
| H | 1.682323 | -1.345796 | -2.270970 |
| C | -3.953506 | 3.971000 | 3.516377 |
| H | -4.326173 | 4.354527 | 4.463689 |
| C | -7.176209 | 1.824206 | -2.279041 |
| H | -8.195544 | 1.959021 | -2.634678 |
| C | -0.787050 | 0.147862 | -4.198197 |
| H | -0.554944 | -0.409942 | -5.114845 |
| H | -1.092258 | 1.154547 | -4.505006 |
| H | -1.645901 | -0.343080 | -3.725348 |
| C | -2.912027 | -5.912130 | 3.280821 |
| H | -3.399063 | -6.736118 | 3.797717 |
| C | 0.453753 | 0.156344 | -3.303377 |
| C | 2.525033 | -2.936238 | -0.167417 |
| H | 3.524604 | -2.607445 | 0.124938 |
| C | -0.259520 | 0.485129 | 3.770343 |
| H | -0.206413 | -0.602808 | 3.903520 |
| H | -1.262254 | 0.731797 | 3.402060 |
| H | -0.162620 | 0.942118 | 4.763564 |
| C | 1.559017 | 0.978533 | -3.968518 |
| H | 2.481399 | 0.961539 | -3.379549 |
| H | 1.279286 | 2.029958 | -4.106659 |
| H | 1.781900 | 0.556355 | -4.956600 |
| Ni | 1.850078 | 1.219684 | -0.341540 |
| H | 2.784342 | 1.506023 | 0.832655 |
| C | 4.234669 | 1.215811 | -0.083564 |
| C | 4.505323 | 2.499150 | -0.612881 |
| H | 5.400324 | 3.061831 | -0.383321 |
| N | 3.606376 | 3.146287 | -1.405847 |
| O | 3.895189 | 4.131533 | -2.081220 |
| O | 2.359124 | 2.718541 | -1.405632 |
| N | 3.820821 | 0.184322 | -0.983949 |
| H | 3.504579 | -0.673791 | -0.531075 |
| C | 4.441673 | -0.186031 | -2.213653 |
| C | 5.257004 | 0.824021 | -2.958205 |
| H | 4.668773 | 1.692304 | -3.275670 |
| H | 6.079332 | 1.208056 | -2.344092 |
| H | 5.666186 | 0.332499 | -3.842447 |
| O | 4.258260 | -1.325298 | -2.594244 |
| C | 5.049609 | 0.785886 | 1.073728 |
| C | 5.341084 | 1.716629 | 2.081833 |
| C | 5.541526 | -0.520986 | 1.190564 |
| C | 6.104737 | 1.350960 | 3.180851 |
| H | 4.933575 | 2.725261 | 2.004335 |
| C | 6.300827 | -0.887068 | 2.297965 |
| H | 5.372839 | -1.246582 | 0.394749 |
| C | 6.580205 | 0.044328 | 3.294601 |
| H | 6.321211 | 2.081708 | 3.956749 |
| H | 6.686840 | -1.901038 | 2.374131 |
| H | 7.173303 | -0.245477 | 4.158933 |

**VI$_{S1}$**

| | | | |
|---|---|---|---|
| P | -0.165581 | -0.911999 | -1.622690 |
| P | -0.933736 | 0.034183 | 1.176098 |
| C | 1.641821 | 1.218458 | 1.479545 |
| C | 0.293853 | 3.033689 | 0.351671 |
| C | 1.778347 | 4.937840 | 1.986278 |
| H | 0.950765 | 5.278600 | 1.368897 |
| C | -1.040175 | 2.714524 | 0.586940 |
| C | 2.313042 | -2.625881 | -0.192174 |
| C | 3.075372 | -1.558480 | -0.902057 |
| C | -1.113563 | -0.953572 | 2.763085 |
| C | 0.623141 | 3.852379 | -0.776699 |
| C | 2.536836 | -0.293932 | -1.132828 |
| C | 4.396011 | -1.853722 | -1.381580 |
| C | 3.310877 | 0.705144 | -1.772253 |
| H | 2.866214 | 1.690458 | -1.923322 |
| C | 0.885309 | 0.107793 | 0.786030 |
| H | 1.253512 | -0.848837 | 1.184992 |
| C | 1.149788 | -3.155948 | -0.743110 |
| C | 0.564003 | -2.551359 | -1.982370 |

| | | | | | | | |
|---|---|---|---|---|---|---|---|
| H | -0.235330 | -3.174388 | -2.399214 | C | 1.964511 | 4.119879 | -1.155348 |
| H | 1.330104 | -2.405585 | -2.756028 | H | 2.776023 | 3.721490 | -0.548669 |
| C | 3.154167 | 3.139360 | 2.899633 | C | 4.974819 | -3.145385 | -1.278184 |
| C | 1.357464 | 2.569807 | 1.287411 | H | 4.402751 | -3.952218 | -0.826074 |
| C | -0.427738 | 4.388498 | -1.591965 | C | 0.504920 | -4.260090 | -0.136481 |
| C | 2.117335 | -4.244089 | 1.670061 | H | -0.391181 | -4.663461 | -0.607607 |
| C | 1.128454 | 0.082301 | -0.742683 | C | 1.213228 | 5.397138 | -3.070511 |
| H | 0.944553 | 1.103177 | -1.101754 | H | 1.454638 | 5.989027 | -3.950616 |
| C | 3.878958 | 4.109718 | 3.632100 | C | 2.494331 | 5.854698 | 2.719768 |
| H | 4.690919 | 3.773271 | 4.275995 | H | 2.229796 | 6.908744 | 2.667437 |
| C | 6.465456 | -1.116139 | -2.491371 | C | -1.098167 | -2.434600 | 2.400402 |
| H | 7.033161 | -0.319404 | -2.970525 | H | -0.199704 | -2.697475 | 1.832068 |
| C | -1.408054 | 1.743769 | 1.663296 | H | -1.967154 | -2.726337 | 1.791595 |
| H | -2.488893 | 1.754611 | 1.861315 | H | -1.105805 | -3.048139 | 3.311418 |
| H | -0.901291 | 1.969109 | 2.614164 | C | -0.803111 | 1.372257 | -3.036362 |
| C | 5.162351 | -0.826010 | -2.022322 | H | -1.208914 | 1.817605 | -3.954293 |
| C | -0.097212 | 5.165579 | -2.728911 | H | 0.096687 | 1.944419 | -2.777069 |
| H | -0.908570 | 5.566866 | -3.335316 | H | -1.552996 | 1.528898 | -2.248943 |
| C | 3.897034 | -2.567135 | 1.738033 | C | 3.644710 | -4.143206 | 3.556594 |
| H | 4.424849 | -1.730076 | 1.284668 | H | 3.982996 | -4.521722 | 4.518719 |
| C | 4.307770 | -3.052021 | 2.958275 | C | 6.999007 | -2.375086 | -2.359101 |
| H | 5.155436 | -2.592750 | 3.463284 | H | 8.000334 | -2.588160 | -2.727108 |
| C | -1.771556 | 4.102109 | -1.263659 | C | 0.690996 | -0.256814 | -4.217370 |
| H | -2.573512 | 4.507516 | -1.878542 | H | 0.502971 | 0.340112 | -5.119109 |
| C | 2.088559 | 3.553939 | 2.036501 | H | 0.838996 | -1.292451 | -4.542823 |
| C | 4.590947 | 0.455787 | -2.189968 | H | 1.627504 | 0.105372 | -3.775116 |
| H | 5.178434 | 1.237558 | -2.669992 | C | 3.561959 | 5.442891 | 3.543081 |
| C | 3.450365 | 1.762601 | 3.010876 | H | 4.123547 | 6.180124 | 4.112819 |
| H | 4.272045 | 1.448348 | 3.653500 | C | -0.507172 | -0.106064 | -3.276292 |
| C | 2.789633 | -3.137473 | 1.057688 | C | -2.062903 | 3.272986 | -0.211274 |
| C | 2.250610 | 4.864396 | -2.277033 | H | -3.104748 | 3.053685 | 0.022886 |
| H | 3.287729 | 5.049206 | -2.550406 | C | 0.034176 | -0.662211 | 3.731237 |
| C | 2.704410 | 0.835214 | 2.333831 | H | 0.181710 | 0.408426 | 3.920547 |
| H | 2.928251 | -0.226584 | 2.440540 | H | 0.986106 | -1.078563 | 3.378088 |
| C | 2.575172 | -4.727025 | 2.920443 | H | -0.185769 | -1.139405 | 4.695226 |
| H | 2.055816 | -5.571661 | 3.372330 | C | -1.738644 | -0.782313 | -3.884941 |
| C | 0.995659 | -4.812104 | 1.020484 | H | -2.632359 | -0.588675 | -3.280172 |
| H | 0.499505 | -5.668120 | 1.476949 | H | -1.634352 | -1.869853 | -3.975571 |
| C | -2.447092 | -0.584380 | 3.416073 | H | -1.913190 | -0.371664 | -4.887256 |
| H | -2.631326 | -1.257252 | 4.262946 | Ni | -1.916931 | -1.005917 | -0.433976 |
| H | -3.302236 | -0.692165 | 2.734633 | H | -3.388209 | -1.220925 | 0.610238 |
| H | -2.453680 | 0.439145 | 3.808398 | C | -4.306838 | -0.938484 | -0.029584 |
| C | 6.239783 | -3.397778 | -1.754782 | C | -4.672387 | -2.103059 | -0.848799 |
| H | 6.656995 | -4.398872 | -1.667654 | H | -5.697019 | -2.425858 | -0.974572 |

| | | | |
|---|---|---|---|
| N | -3.775613 | -2.751168 | -1.594127 |
| O | -4.035682 | -3.615493 | -2.430692 |
| O | -2.454293 | -2.489192 | -1.395781 |
| N | -3.841429 | 0.230509 | -0.819130 |
| H | -3.468175 | 0.985544 | -0.243113 |
| C | -4.490275 | 0.828144 | -1.923718 |
| C | -5.431606 | 0.013822 | -2.759912 |
| H | -4.962520 | -0.882237 | -3.180673 |
| H | -6.290815 | -0.324383 | -2.168706 |
| H | -5.788894 | 0.649359 | -3.572107 |
| O | -4.230306 | 1.994150 | -2.165063 |
| C | -5.365631 | -0.600895 | 0.997780 |
| C | -5.808100 | -1.619097 | 1.849405 |
| C | -5.878831 | 0.688925 | 1.145487 |
| C | -6.757536 | -1.354147 | 2.828026 |
| H | -5.398327 | -2.624131 | 1.737025 |
| C | -6.835754 | 0.950505 | 2.124602 |
| H | -5.556025 | 1.493765 | 0.484896 |
| C | -7.274017 | -0.066386 | 2.966575 |
| H | -7.097625 | -2.153643 | 3.482595 |
| H | -7.241428 | 1.954995 | 2.223644 |
| H | -8.019855 | 0.141783 | 3.730419 |

**VII$_{S1}$**

| | | | |
|---|---|---|---|
| P | -0.281749 | -0.022620 | 1.590820 |
| P | -0.026737 | -1.057052 | -1.289731 |
| C | 2.634802 | -0.045885 | -1.390161 |
| C | 2.895365 | -2.302685 | -0.283279 |
| C | 5.388212 | -2.570565 | -1.760975 |
| H | 4.987474 | -3.417159 | -1.208455 |
| C | 1.743122 | -3.020371 | -0.583643 |
| C | 0.049238 | 3.030374 | 0.223911 |
| C | 1.358441 | 2.895972 | 0.924655 |
| C | -0.733456 | -0.423660 | -2.904058 |
| C | 3.647632 | -2.646841 | 0.886412 |
| C | 1.976399 | 1.663896 | 1.131444 |
| C | 2.008780 | 4.091779 | 1.386830 |
| C | 3.284323 | 1.611942 | 1.672582 |
| H | 3.761694 | 0.637080 | 1.777044 |
| C | 1.264456 | 0.177514 | -0.788331 |
| H | 0.872773 | 1.124017 | -1.191922 |
| C | -1.115542 | 2.481751 | 0.750651 |
| C | -1.059527 | 1.596454 | 1.956393 |
| H | -2.060837 | 1.396477 | 2.361080 |
| H | -0.465750 | 2.048780 | 2.765593 |
| C | 5.175744 | -0.289410 | -2.607316 |
| C | 3.371601 | -1.209037 | -1.176377 |
| C | 3.265883 | -3.789156 | 1.660808 |
| C | -1.309276 | 4.065958 | -1.572803 |
| C | 1.335497 | 0.349253 | 0.748459 |
| H | 1.981558 | -0.459780 | 1.121419 |
| C | 6.436408 | -0.439917 | -3.233953 |
| H | 6.831406 | 0.395256 | -3.811454 |
| C | 3.970418 | 5.188265 | 2.392091 |
| H | 4.974055 | 5.103419 | 2.807371 |
| C | 0.874390 | -2.592774 | -1.725588 |
| H | 0.121050 | -3.350937 | -1.963120 |
| H | 1.461607 | -2.397714 | -2.634535 |
| C | 3.324900 | 4.010217 | 1.946634 |
| C | 3.997260 | -4.108340 | 2.830478 |
| H | 3.696791 | -4.981268 | 3.409099 |
| C | 1.128836 | 4.236239 | -1.681637 |
| H | 2.110012 | 4.030805 | -1.257083 |
| C | 1.027100 | 4.922707 | -2.869756 |
| H | 1.929570 | 5.249301 | -3.382744 |
| C | 2.135664 | -4.542770 | 1.263911 |
| H | 1.852673 | -5.417338 | 1.848337 |
| C | 4.639856 | -1.367343 | -1.830986 |
| C | 3.949645 | 2.746970 | 2.049504 |
| H | 4.961238 | 2.687965 | 2.449197 |
| C | 4.426823 | 0.902237 | -2.732981 |
| H | 4.843577 | 1.730652 | -3.304545 |
| C | -0.027667 | 3.784326 | -0.994371 |
| C | 5.427586 | -2.199070 | 2.484027 |
| H | 6.264688 | -1.584998 | 2.810163 |
| C | 3.188181 | 1.008629 | -2.157349 |
| H | 2.609093 | 1.926937 | -2.269944 |
| C | -1.377895 | 4.795369 | -2.784265 |
| H | -2.359800 | 5.006240 | -3.207083 |
| C | -2.473068 | 3.569217 | -0.940740 |
| H | -3.450604 | 3.797468 | -1.365341 |
| C | -1.531504 | -1.536465 | -3.586541 |
| H | -1.967602 | -1.144136 | -4.513683 |
| H | -2.364707 | -1.927101 | -2.991799 |
| H | -0.894429 | -2.385031 | -3.865318 |
| C | 2.036088 | 6.491008 | 1.795778 |
| H | 1.532054 | 7.454551 | 1.756517 |
| C | 4.750075 | -1.872672 | 1.331952 |

| | | | |
|---|---|---|---|
| H | 5.061723 | -1.008247 | 0.748519 |
| C | 1.387739 | 5.366800 | 1.340764 |
| H | 0.375618 | 5.454827 | 0.952896 |
| C | -2.372399 | 2.769066 | 0.168509 |
| H | -3.273336 | 2.374754 | 0.638035 |
| C | 5.052573 | -3.328603 | 3.240248 |
| H | 5.600206 | -3.580992 | 4.145651 |
| C | 6.603731 | -2.689774 | -2.392737 |
| H | 7.153581 | -3.626453 | -2.328258 |
| C | -1.607478 | 0.786635 | -2.592579 |
| H | -1.031512 | 1.572768 | -2.093273 |
| H | -2.466444 | 0.560210 | -1.948796 |
| H | -2.000441 | 1.216062 | -3.523730 |
| C | 0.446470 | -2.223495 | 3.081455 |
| H | 0.863415 | -2.647457 | 4.004981 |
| H | 1.189102 | -2.379034 | 2.290612 |
| H | -0.458309 | -2.782834 | 2.823327 |
| C | -0.236437 | 5.213568 | -3.424612 |
| H | -0.303109 | 5.766176 | -4.359315 |
| C | 3.343442 | 6.407757 | 2.316629 |
| H | 3.846755 | 7.306810 | 2.665955 |
| C | 1.442129 | -0.064105 | 3.800734 |
| H | 1.601865 | -0.409986 | 4.830029 |
| H | 1.381658 | 1.031164 | 3.830714 |
| H | 2.335436 | -0.345563 | 3.230845 |
| C | 7.142141 | -1.613125 | -3.126452 |
| H | 8.109098 | -1.718641 | -3.613537 |
| C | 0.168658 | -0.736518 | 3.273321 |
| C | 1.372320 | -4.142053 | 0.195974 |
| H | 0.474036 | -4.696421 | -0.077704 |
| C | 0.394531 | 0.029627 | -3.836831 |
| H | 1.160535 | -0.736724 | -4.005359 |
| H | 0.893513 | 0.933353 | -3.469462 |
| H | -0.039553 | 0.276170 | -4.814464 |
| C | -0.951530 | -0.503831 | 4.289771 |
| H | -1.891440 | -0.980417 | 4.005452 |
| H | -1.125347 | 0.563181 | 4.475681 |
| H | -0.637509 | -0.942730 | 5.245320 |
| Ni | -1.406505 | -1.395897 | 0.354911 |
| H | -3.306849 | -0.156945 | 0.333507 |
| C | -4.352038 | -0.502079 | 0.187342 |
| C | -4.618053 | -1.529138 | 1.251258 |
| H | -5.621452 | -1.638968 | 1.654473 |
| N | -3.689577 | -1.862464 | 2.176043 |
| O | -3.960703 | -2.382892 | 3.259804 |
| O | -2.380948 | -1.720324 | 1.928610 |
| N | -4.333396 | -1.036457 | -1.182363 |
| H | -3.407576 | -1.135629 | -1.580120 |
| C | -5.216928 | -1.910626 | -1.773484 |
| C | -6.598436 | -2.000759 | -1.191394 |
| H | -6.652175 | -2.875910 | -0.531507 |
| H | -6.891428 | -1.116001 | -0.617029 |
| H | -7.314327 | -2.166176 | -1.999887 |
| O | -4.848681 | -2.580383 | -2.733819 |
| C | -5.236052 | 0.724501 | 0.336568 |
| C | -5.562470 | 1.190340 | 1.615094 |
| C | -5.637782 | 1.467203 | -0.776863 |
| C | -6.277257 | 2.373287 | 1.776508 |
| H | -5.251894 | 0.622976 | 2.493345 |
| C | -6.357979 | 2.647640 | -0.614020 |
| H | -5.380336 | 1.112416 | -1.773722 |
| C | -6.677250 | 3.106169 | 0.661631 |
| H | -6.526233 | 2.720109 | 2.777419 |
| H | -6.672517 | 3.210897 | -1.490895 |
| H | -7.240133 | 4.028727 | 0.786948 |
| C | -2.898893 | -3.878423 | -0.766327 |
| O | -2.074890 | -2.950111 | -0.647815 |
| O | -3.994426 | -4.000160 | -0.066221 |
| C | -2.715003 | -4.944607 | -1.784644 |
| H | -3.320715 | -4.662479 | -2.655760 |
| H | -1.670662 | -5.025034 | -2.092461 |
| H | -3.087694 | -5.905320 | -1.422346 |
| H | -4.190941 | -3.163986 | 0.467417 |

**$TSIII_{S1}$**

| | | | |
|---|---|---|---|
| P | -0.264028 | -0.009168 | 1.596017 |
| P | -0.018000 | -1.065379 | -1.274345 |
| C | 2.645675 | -0.063798 | -1.385738 |
| C | 2.895373 | -2.314054 | -0.261211 |
| C | 5.387914 | -2.603418 | -1.733971 |
| H | 4.983095 | -3.444025 | -1.175411 |
| C | 1.742279 | -3.032055 | -0.558165 |
| C | 0.051103 | 3.029293 | 0.201908 |
| C | 1.365092 | 2.906265 | 0.896459 |
| C | -0.723062 | -0.437399 | -2.891197 |
| C | 3.646246 | -2.652993 | 0.910880 |
| C | 1.986983 | 1.678363 | 1.117537 |
| C | 2.014401 | 4.109517 | 1.340528 |

| | | | | | | | |
|---|---|---|---|---|---|---|---|
| C | 3.295196 | 1.635908 | 1.658651 | C | -1.512496 | -1.552669 | -3.579023 |
| H | 3.774387 | 0.663288 | 1.775869 | H | -1.962441 | -1.157105 | -4.498048 |
| C | 1.276817 | 0.170357 | -0.785496 | H | -2.332109 | -1.961957 | -2.979695 |
| H | 0.888223 | 1.113765 | -1.198909 | H | -0.866116 | -2.389599 | -3.870899 |
| C | -1.108472 | 2.483079 | 0.742613 | C | 2.039304 | 6.514606 | 1.714344 |
| C | -1.041245 | 1.610531 | 1.956775 | H | 1.533605 | 7.476668 | 1.662698 |
| H | -2.039569 | 1.412603 | 2.369735 | C | 4.747426 | -1.876007 | 1.354750 |
| H | -0.442875 | 2.071952 | 2.757334 | H | 5.058808 | -1.013452 | 0.768195 |
| C | 5.186164 | -0.328159 | -2.598534 | C | 1.391001 | 5.382771 | 1.278648 |
| C | 3.376804 | -1.228651 | -1.161953 | H | 0.377241 | 5.463583 | 0.893754 |
| C | 3.264452 | -3.792903 | 1.688781 | C | -2.370972 | 2.761348 | 0.168324 |
| C | -1.325039 | 4.047448 | -1.591302 | H | -3.266520 | 2.367156 | 0.648569 |
| C | 1.350023 | 0.357828 | 0.748681 | C | 5.050089 | -3.325849 | 3.267637 |
| H | 1.999108 | -0.445828 | 1.127682 | H | 5.597675 | -3.575220 | 4.173912 |
| C | 6.446712 | -0.489132 | -3.222800 | C | 6.603564 | -2.732901 | -2.363558 |
| H | 6.845852 | 0.339687 | -3.806571 | H | 7.149110 | -3.671513 | -2.291227 |
| C | 3.977595 | 5.223691 | 2.323199 | C | -1.599072 | 0.771547 | -2.581397 |
| H | 4.982784 | 5.146505 | 2.736210 | H | -1.022541 | 1.558355 | -2.084118 |
| C | 0.872342 | -2.608507 | -1.700485 | H | -2.455096 | 0.546656 | -1.933586 |
| H | 0.111353 | -3.361979 | -1.928265 | H | -1.994854 | 1.198936 | -3.512195 |
| H | 1.456272 | -2.422825 | -2.613589 | C | 0.452100 | -2.208121 | 3.090288 |
| C | 3.332257 | 4.038152 | 1.897649 | H | 0.859898 | -2.637330 | 4.015438 |
| C | 3.995624 | -4.107767 | 2.859749 | H | 1.196328 | -2.371655 | 2.302683 |
| H | 3.695904 | -4.979360 | 3.440759 | H | -0.456178 | -2.760010 | 2.827034 |
| C | 1.111859 | 4.219258 | -1.723966 | C | -0.270533 | 5.181574 | -3.461780 |
| H | 2.096967 | 4.018722 | -1.306167 | H | -0.346593 | 5.726363 | -4.400342 |
| C | 0.998410 | 4.895952 | -2.916625 | C | 3.348712 | 6.441117 | 2.231345 |
| H | 1.895679 | 5.218811 | -3.440889 | H | 3.852141 | 7.345952 | 2.565164 |
| C | 2.134765 | -4.548471 | 1.293802 | C | 1.471149 | -0.056234 | 3.800078 |
| H | 1.852758 | -5.422033 | 1.880208 | H | 1.632940 | -0.401168 | 4.829386 |
| C | 4.644943 | -1.397575 | -1.814146 | H | 1.419237 | 1.039467 | 3.827651 |
| C | 3.959059 | 2.777529 | 2.018228 | H | 2.359803 | -0.345753 | 3.226777 |
| H | 4.971411 | 2.726076 | 2.417028 | C | 7.147306 | -1.664433 | -3.105227 |
| C | 4.442895 | 0.865897 | -2.734276 | H | 8.114210 | -1.777889 | -3.590620 |
| H | 4.863985 | 1.687900 | -3.311845 | C | 0.189453 | -0.718066 | 3.279594 |
| C | -0.037755 | 3.772170 | -1.022286 | C | 1.370940 | -4.151257 | 0.224824 |
| C | 5.424452 | -2.198081 | 2.508404 | H | 0.472486 | -4.705828 | -0.047678 |
| H | 6.260772 | -1.582259 | 2.833257 | C | 0.407671 | 0.018799 | -3.820064 |
| C | 3.204228 | 0.982403 | -2.160546 | H | 1.182611 | -0.741326 | -3.975916 |
| H | 2.629279 | 1.902391 | -2.280513 | H | 0.895038 | 0.930867 | -3.457954 |
| C | -1.405616 | 4.767568 | -2.807493 | H | -0.022615 | 0.251991 | -4.802570 |
| H | -2.391549 | 4.974002 | -3.223059 | C | -0.924273 | -0.469730 | 4.299588 |
| C | -2.482565 | 3.552734 | -0.946078 | H | -1.874877 | -0.926023 | 4.016578 |
| H | -3.463905 | 3.775802 | -1.364707 | H | -1.077890 | 0.599579 | 4.489543 |

| | | | |
|---|---|---|---|
| H | -0.617184 | -0.918235 | 5.252900 |
| Ni | -1.401248 | -1.388224 | 0.370099 |
| H | -3.300659 | -0.221660 | 0.261731 |
| C | -4.350856 | -0.554767 | 0.121034 |
| C | -4.595099 | -1.648815 | 1.144826 |
| H | -5.602757 | -1.721805 | 1.552387 |
| N | -3.691655 | -1.813103 | 2.173643 |
| O | -4.016994 | -2.199759 | 3.291608 |
| O | -2.399592 | -1.677073 | 1.952642 |
| N | -4.371265 | -1.021232 | -1.266486 |
| H | -3.455847 | -1.120778 | -1.687167 |
| C | -5.272629 | -1.876171 | -1.866996 |
| C | -6.639978 | -1.981432 | -1.254169 |
| H | -6.678334 | -2.873081 | -0.616194 |
| H | -6.919614 | -1.110631 | -0.652161 |
| H | -7.374778 | -2.126617 | -2.049365 |
| O | -4.929566 | -2.508344 | -2.859037 |
| C | -5.221051 | 0.666134 | 0.362698 |
| C | -5.513597 | 1.062614 | 1.672916 |
| C | -5.645858 | 1.470821 | -0.698080 |
| C | -6.216147 | 2.238970 | 1.916755 |
| H | -5.193097 | 0.446820 | 2.514420 |
| C | -6.353464 | 2.644402 | -0.452348 |
| H | -5.416529 | 1.168945 | -1.718822 |
| C | -6.637966 | 3.034226 | 0.854074 |
| H | -6.439754 | 2.530132 | 2.940963 |
| H | -6.686850 | 3.255992 | -1.289030 |
| H | -7.192017 | 3.951026 | 1.043921 |
| C | -2.940844 | -3.815486 | -0.769066 |
| O | -2.070514 | -2.914573 | -0.633415 |
| O | -4.042482 | -3.897148 | -0.111101 |
| C | -2.743507 | -4.873620 | -1.800707 |
| H | -3.339965 | -4.585051 | -2.675990 |
| H | -1.695856 | -4.957881 | -2.097868 |
| H | -3.123596 | -5.836242 | -1.450874 |
| H | -4.275100 | -2.965987 | 0.455642 |

**VIII$_{S1}$**

| | | | |
|---|---|---|---|
| P | -0.076094 | -0.139009 | -1.829672 |
| P | 0.321269 | -0.804927 | 1.104522 |
| C | -2.402026 | -0.188355 | 1.628366 |
| C | -2.486653 | -2.587640 | 0.845819 |
| C | -4.605221 | -2.989210 | 2.815349 |
| H | -4.198356 | -3.839164 | 2.272705 |
| C | -1.191265 | -3.073500 | 0.985401 |
| C | -0.732287 | 2.985892 | -0.779305 |
| C | -2.092695 | 2.545748 | -1.205706 |
| C | 1.243207 | 0.139983 | 2.420909 |
| C | -3.364618 | -3.207718 | -0.103199 |
| C | -2.499908 | 1.213602 | -1.175275 |
| C | -3.013997 | 3.545691 | -1.672512 |
| C | -3.838326 | 0.872409 | -1.488575 |
| H | -4.136489 | -0.174463 | -1.413500 |
| C | -1.226335 | 0.143169 | 0.737568 |
| H | -0.929982 | 1.181423 | 0.951829 |
| C | 0.407790 | 2.578420 | -1.466839 |
| C | 0.323169 | 1.516782 | -2.518192 |
| H | 1.266575 | 1.421309 | -3.065446 |
| H | -0.470560 | 1.739007 | -3.247602 |
| C | -4.582142 | -0.612813 | 3.376432 |
| C | -2.971633 | -1.459602 | 1.691727 |
| C | -2.931534 | -4.379557 | -0.805840 |
| C | 0.687344 | 4.477741 | 0.600042 |
| C | -1.585183 | 0.083589 | -0.767474 |
| H | -2.127854 | -0.861006 | -0.927071 |
| C | -5.653113 | -0.847084 | 4.271715 |
| H | -6.047324 | -0.004998 | 4.839587 |
| C | -5.272253 | 4.160317 | -2.437044 |
| H | -6.293610 | 3.856760 | -2.664385 |
| C | -0.214321 | -2.373897 | 1.880247 |
| H | 0.679593 | -2.984645 | 2.049565 |
| H | -0.653728 | -2.133577 | 2.859359 |
| C | -4.361051 | 3.174282 | -1.989805 |
| C | -3.795897 | -4.979132 | -1.753278 |
| H | -3.453740 | -5.871866 | -2.276124 |
| C | -1.678483 | 4.237075 | 1.169130 |
| H | -2.660604 | 3.812238 | 0.967863 |
| C | -1.505780 | 5.082509 | 2.241093 |
| H | -2.351755 | 5.317084 | 2.884227 |
| C | -1.629470 | -4.881382 | -0.571289 |
| H | -1.307544 | -5.777893 | -1.100256 |
| C | -4.050181 | -1.699681 | 2.609095 |
| C | -4.749974 | 1.821985 | -1.863063 |
| H | -5.779540 | 1.544819 | -2.085925 |
| C | -4.015149 | 0.672789 | 3.230287 |
| H | -4.431562 | 1.502215 | 3.800692 |
| C | -0.593922 | 3.901369 | 0.317426 |
| C | -5.455593 | -3.279995 | -1.346269 |

| | | | | | | | |
|---|---|---|---|---|---|---|---|
| H | -6.432917 | -2.854410 | -1.565243 | C | 0.292881 | 0.587234 | 3.534084 |
| C | -2.947956 | 0.869849 | 2.395223 | H | -0.297311 | -0.233936 | 3.959923 |
| H | -2.505847 | 1.862504 | 2.297684 | H | -0.404114 | 1.361590 | 3.190388 |
| C | 0.825548 | 5.359239 | 1.698451 | H | 0.884570 | 1.024253 | 4.349153 |
| H | 1.806314 | 5.791416 | 1.895217 | C | 0.484173 | -0.980900 | -4.388246 |
| C | 1.790815 | 4.132032 | -0.214635 | H | 1.481757 | -1.222367 | -4.003638 |
| H | 2.756379 | 4.600440 | -0.024475 | H | 0.510497 | 0.029515 | -4.812832 |
| C | 2.328926 | -0.774365 | 2.989839 | H | 0.274422 | -1.672065 | -5.214377 |
| H | 2.973539 | -0.195176 | 3.663150 | Ni | 1.396057 | -1.242380 | -0.684194 |
| H | 2.969517 | -1.200897 | 2.207124 | H | 3.827131 | -0.577078 | 0.339619 |
| H | 1.909619 | -1.605568 | 3.569538 | C | 4.913500 | -0.525504 | 0.179886 |
| C | -3.545027 | 5.831923 | -2.320073 | C | 5.081780 | -0.402193 | -1.336183 |
| H | -3.226547 | 6.861598 | -2.469639 | H | 5.974039 | 0.144271 | -1.657154 |
| C | -4.648148 | -2.683617 | -0.405268 | N | 3.926779 | 0.307640 | -1.995928 |
| H | -4.993223 | -1.794169 | 0.118774 | O | 4.057090 | 0.635601 | -3.165296 |
| C | -2.639477 | 4.900321 | -1.868341 | O | 2.895268 | 0.485269 | -1.328580 |
| H | -1.613984 | 5.205206 | -1.673928 | N | 5.386329 | -1.810079 | 0.671175 |
| C | 1.657799 | 3.187343 | -1.198473 | H | 4.654639 | -2.466373 | 0.923874 |
| H | 2.512604 | 2.904294 | -1.809532 | C | 6.591859 | -2.427129 | 0.404818 |
| C | -5.031604 | -4.441917 | -2.023342 | C | 7.730739 | -1.550597 | -0.047144 |
| H | -5.682828 | -4.907797 | -2.759667 | H | 7.924055 | -1.718363 | -1.113480 |
| C | -5.638362 | -3.185911 | 3.701347 | H | 7.567889 | -0.481120 | 0.121673 |
| H | -6.041223 | -4.186669 | 3.843124 | H | 8.635504 | -1.858956 | 0.482998 |
| C | 1.853195 | 1.369166 | 1.757122 | O | 6.701701 | -3.636378 | 0.545708 |
| H | 1.081836 | 1.987130 | 1.282808 | C | 5.401930 | 0.682390 | 0.957099 |
| H | 2.589243 | 1.112366 | 0.986143 | C | 5.386796 | 1.959230 | 0.386838 |
| H | 2.352810 | 1.995371 | 2.509286 | C | 5.766562 | 0.550708 | 2.299913 |
| C | -0.753570 | -2.583777 | -2.890819 | C | 5.725658 | 3.078436 | 1.142657 |
| H | -1.175078 | -3.180296 | -3.711248 | H | 5.108210 | 2.101442 | -0.658287 |
| H | -1.437690 | -2.674581 | -2.039031 | C | 6.104788 | 1.669026 | 3.055031 |
| H | 0.205753 | -3.029014 | -2.603117 | H | 5.791221 | -0.442237 | 2.746505 |
| C | -0.245194 | 5.655569 | 2.507496 | C | 6.081995 | 2.937678 | 2.480525 |
| H | -0.124285 | 6.329741 | 3.352752 | H | 5.718147 | 4.063157 | 0.678533 |
| C | -4.877987 | 5.465865 | -2.596454 | H | 6.394519 | 1.546738 | 4.097021 |
| H | -5.585301 | 6.214177 | -2.947395 | H | 6.350758 | 3.811324 | 3.070230 |
| C | -1.948543 | -0.638668 | -3.868547 | C | 2.881065 | -2.966752 | -1.108111 |
| H | -2.143287 | -1.143140 | -4.824207 | O | 2.515529 | -2.647011 | 0.083632 |
| H | -1.979973 | 0.441433 | -4.058205 | O | 2.453797 | -2.224401 | -2.052131 |
| H | -2.778878 | -0.888576 | -3.196854 | C | 3.750433 | -4.144325 | -1.353508 |
| C | -6.177662 | -2.106438 | 4.430392 | H | 4.625989 | -4.146666 | -0.689559 |
| H | -6.998390 | -2.275974 | 5.124026 | H | 3.189057 | -5.059233 | -1.128824 |
| C | -0.605921 | -1.129078 | -3.323817 | H | 4.070519 | -4.179819 | -2.397145 |
| C | -0.770993 | -4.220375 | 0.269256 | H | 5.091562 | -1.384304 | -1.822902 |
| H | 0.244221 | -4.587813 | 0.423196 | | | | |

**IV$_{R2}$**

| | | | |
|---|---|---|---|
| P | -0.322946 | 0.591718 | 1.367967 |
| P | -0.201356 | -0.342741 | -1.555793 |
| C | 2.601611 | -0.853518 | -1.218522 |
| C | 1.507243 | -2.964970 | -0.363722 |
| C | 3.710323 | -4.421472 | -1.588511 |
| H | 2.858773 | -4.960683 | -1.180528 |
| C | 0.210964 | -2.984877 | -0.866416 |
| C | 1.765742 | 3.048090 | 0.347828 |
| C | 2.680372 | 2.242578 | 1.209375 |
| C | -0.145558 | 0.554358 | -3.198805 |
| C | 1.805781 | -3.696782 | 0.831824 |
| C | 2.506450 | 0.871553 | 1.394392 |
| C | 3.749031 | 2.912445 | 1.897537 |
| C | 3.425435 | 0.142460 | 2.188579 |
| H | 3.276603 | -0.933979 | 2.294722 |
| C | 1.456778 | 0.023345 | -0.764910 |
| H | 1.663617 | 1.043123 | -1.122790 |
| C | 0.416033 | 3.165738 | 0.666950 |
| C | -0.158723 | 2.374571 | 1.798636 |
| H | -1.153821 | 2.742801 | 2.079911 |
| H | 0.489017 | 2.446686 | 2.682384 |
| C | 4.818214 | -2.327475 | -2.176022 |
| C | 2.601703 | -2.237686 | -1.066960 |
| C | 0.803960 | -4.537217 | 1.414785 |
| C | 1.415954 | 4.632622 | -1.524972 |
| C | 1.368034 | 0.093657 | 0.779187 |
| H | 1.450514 | -0.942933 | 1.132970 |
| C | 5.900596 | -3.081798 | -2.688399 |
| H | 6.745431 | -2.547330 | -3.121870 |
| C | 5.719611 | 2.810933 | 3.371365 |
| H | 6.405793 | 2.210773 | 3.968170 |
| C | -0.162517 | -2.120265 | -2.030486 |
| H | -1.156854 | -2.374937 | -2.420401 |
| H | 0.556171 | -2.225631 | -2.857287 |
| C | 4.663399 | 2.152429 | 2.697361 |
| C | 1.098351 | -5.243997 | 2.605548 |
| H | 0.329075 | -5.889693 | 3.029549 |
| C | 3.597593 | 3.555367 | -1.274372 |
| H | 4.262351 | 2.884322 | -0.732916 |
| C | 4.045709 | 4.201510 | -2.403453 |
| H | 5.062338 | 4.033193 | -2.753440 |
| C | -0.467954 | -4.610767 | 0.801197 |
| H | -1.214840 | -5.284414 | 1.222506 |
| C | 3.697736 | -3.002823 | -1.593839 |
| C | 4.480818 | 0.755957 | 2.810426 |
| H | 5.183319 | 0.177434 | 3.409455 |
| C | 4.813762 | -0.915450 | -2.230133 |
| H | 5.677185 | -0.399673 | -2.649182 |
| C | 2.273160 | 3.741344 | -0.799878 |
| C | 3.299896 | -4.266020 | 2.667472 |
| H | 4.262844 | -4.156550 | 3.162734 |
| C | 3.731828 | -0.208221 | -1.779517 |
| H | 3.726015 | 0.880810 | -1.842311 |
| C | 1.915670 | 5.296034 | -2.671409 |
| H | 1.251167 | 5.970438 | -3.211120 |
| C | 0.074841 | 4.792468 | -1.103842 |
| H | -0.570166 | 5.482808 | -1.646669 |
| C | 1.227491 | 0.415233 | -3.863266 |
| H | 1.165176 | 0.811803 | -4.884815 |
| H | 1.578237 | -0.621804 | -3.936402 |
| H | 1.997285 | 0.995475 | -3.341495 |
| C | 4.958322 | 4.930872 | 2.525558 |
| H | 5.066379 | 6.012440 | 2.474486 |
| C | 3.055503 | -3.588605 | 1.494380 |
| H | 3.827138 | -2.950172 | 1.067020 |
| C | 3.927418 | 4.319593 | 1.850590 |
| H | 3.230238 | 4.927857 | 1.279641 |
| C | -0.415935 | 4.046822 | -0.063172 |
| H | -1.456419 | 4.154175 | 0.246395 |
| C | 2.317617 | -5.109897 | 3.225899 |
| H | 2.527686 | -5.651393 | 4.146019 |
| C | 4.771927 | -5.126428 | -2.105388 |
| H | 4.750242 | -6.214334 | -2.095440 |
| C | -1.213785 | -0.013912 | -4.129186 |
| H | -2.216867 | 0.015021 | -3.690936 |
| H | -1.011434 | -1.052765 | -4.415031 |
| H | -1.233199 | 0.580348 | -5.051784 |
| C | -0.343219 | -1.728920 | 2.849918 |
| H | -0.745270 | -2.281141 | 3.711628 |
| H | 0.726055 | -1.970434 | 2.800574 |
| H | -0.821438 | -2.114129 | 1.940600 |
| C | 3.200975 | 5.083696 | -3.108329 |
| H | 3.568620 | 5.591970 | -3.997213 |
| C | 5.871434 | 4.173133 | 3.286445 |
| H | 6.684960 | 4.669629 | 3.810951 |
| C | 0.348146 | 0.294715 | 4.133204 |
| H | 0.215149 | -0.320871 | 5.033296 |

| | | | |
|---|---|---|---|
| H | 0.119337 | 1.327691 | 4.418581 |
| H | 1.407940 | 0.241509 | 3.857005 |
| C | 5.883855 | -4.454674 | -2.652662 |
| H | 6.719605 | -5.024703 | -3.053204 |
| C | -0.578021 | -0.230893 | 3.035837 |
| C | -0.767398 | -3.827212 | -0.284407 |
| H | -1.760320 | -3.853841 | -0.732780 |
| C | -0.401319 | 2.031731 | -2.932860 |
| H | 0.304191 | 2.431059 | -2.196691 |
| H | -1.413017 | 2.218502 | -2.551656 |
| H | -0.275587 | 2.611970 | -3.857360 |
| C | -2.030655 | 0.044864 | 3.436248 |
| H | -2.739960 | -0.338383 | 2.696157 |
| H | -2.225706 | 1.118968 | 3.552599 |
| H | -2.242283 | -0.433463 | 4.402483 |
| Ni | -1.743702 | 0.187481 | -0.244257 |
| H | -2.418795 | 0.093389 | -1.604311 |
| C | -5.439466 | -0.911448 | -0.216141 |
| C | -4.924407 | -0.969565 | -1.472846 |
| N | -4.123811 | -2.021919 | -2.021420 |
| O | -3.976873 | -3.083373 | -1.404804 |
| O | -3.648348 | -1.803894 | -3.142140 |
| H | -5.120463 | -0.197369 | -2.205717 |
| N | -5.049554 | -1.770817 | 0.807862 |
| H | -5.784096 | -2.107898 | 1.418575 |
| C | -3.756421 | -2.162852 | 1.089940 |
| O | -2.779721 | -1.614873 | 0.591176 |
| C | -3.648594 | -3.314536 | 2.038060 |
| H | -3.723955 | -4.241039 | 1.453583 |
| H | -2.675580 | -3.302651 | 2.533930 |
| H | -4.446500 | -3.332498 | 2.788186 |
| C | -6.435041 | 0.102441 | 0.151232 |
| C | -6.552059 | 0.513056 | 1.490271 |
| C | -7.286876 | 0.675153 | -0.808186 |
| C | -7.496571 | 1.461752 | 1.860206 |
| H | -5.869352 | 0.108219 | 2.236276 |
| C | -8.219040 | 1.634465 | -0.436795 |
| H | -7.238176 | 0.339812 | -1.842501 |
| C | -8.331303 | 2.026624 | 0.898132 |
| H | -7.573516 | 1.770165 | 2.900678 |
| H | -8.878683 | 2.062958 | -1.187896 |
| H | -9.073554 | 2.767657 | 1.187012 |
| C | -3.851391 | 2.133214 | -0.083069 |
| O | -3.158121 | 1.361778 | 0.601901 |
| C | -4.731678 | 3.173725 | 0.508212 |
| H | -4.410155 | 4.162666 | 0.163176 |
| H | -5.762839 | 3.038225 | 0.161471 |
| H | -4.699098 | 3.132270 | 1.597584 |
| O | -3.841905 | 2.111541 | -1.399073 |
| H | -3.203122 | 1.366537 | -1.638266 |

**V$_{R2}$**

| | | | |
|---|---|---|---|
| P | -0.968999 | -0.086033 | 1.311166 |
| P | -0.365833 | -0.900791 | -1.663925 |
| C | 2.348219 | -0.115498 | -1.256146 |
| C | 2.300816 | -2.490524 | -0.423177 |
| C | 4.968616 | -2.787032 | -1.567527 |
| H | 4.451959 | -3.649558 | -1.153197 |
| C | 1.149143 | -3.033630 | -0.979423 |
| C | 0.002245 | 3.025559 | 0.345803 |
| C | 1.147371 | 2.628508 | 1.213126 |
| C | -0.795764 | 0.006661 | -3.243502 |
| C | 2.825757 | -3.047064 | 0.785802 |
| C | 1.534908 | 1.299469 | 1.366298 |
| C | 1.856986 | 3.651954 | 1.929907 |
| C | 2.694791 | 0.985541 | 2.116193 |
| H | 3.000112 | -0.060071 | 2.180776 |
| C | 0.928567 | 0.153903 | -0.803396 |
| H | 0.656026 | 1.164907 | -1.144304 |
| C | -1.288507 | 2.597416 | 0.635055 |
| C | -1.535329 | 1.616228 | 1.736474 |
| H | -2.605401 | 1.562541 | 1.968807 |
| H | -1.010145 | 1.919560 | 2.651892 |
| C | 5.003834 | -0.424506 | -2.187036 |
| C | 2.982402 | -1.346940 | -1.089419 |
| C | 2.197070 | -4.198609 | 1.361019 |
| C | -0.914997 | 4.382832 | -1.514170 |
| C | 0.793748 | 0.142368 | 0.732975 |
| H | 1.274390 | -0.784454 | 1.079514 |
| C | 6.316860 | -0.607713 | -2.682790 |
| H | 6.829413 | 0.247039 | -3.123046 |
| C | 3.710989 | 4.313513 | 3.408158 |
| H | 4.591091 | 4.028134 | 3.983430 |
| C | 0.552832 | -2.398524 | -2.198038 |
| H | -0.154146 | -3.070197 | -2.699973 |
| H | 1.327533 | -2.111300 | -2.921473 |
| C | 3.013061 | 3.305537 | 2.701270 |
| C | 2.713487 | -4.739966 | 2.563517 |

| | | | |
|---|---|---|---|
| H | 2.229692 | -5.620450 | 2.986101 |
| C | 1.510105 | 4.268590 | -1.214107 |
| H | 2.377472 | 3.913871 | -0.660095 |
| C | 1.684797 | 5.067137 | -2.320733 |
| H | 2.690549 | 5.336006 | -2.637893 |
| C | 1.041404 | -4.732307 | 0.743277 |
| H | 0.558692 | -5.601101 | 1.189999 |
| C | 4.310980 | -1.530138 | -1.597343 |
| C | 3.424513 | 1.954824 | 2.750596 |
| H | 4.317279 | 1.693613 | 3.317640 |
| C | 4.350061 | 0.825045 | -2.270767 |
| H | 4.882108 | 1.672689 | -2.701340 |
| C | 0.213203 | 3.891121 | -0.778334 |
| C | 4.396034 | -3.015620 | 2.644518 |
| H | 5.242949 | -2.556835 | 3.151074 |
| C | 3.059018 | 0.965520 | -1.834749 |
| H | 2.559451 | 1.931353 | -1.917750 |
| C | -0.699219 | 5.214294 | -2.639789 |
| H | -1.567220 | 5.580690 | -3.187541 |
| C | -2.216868 | 4.002969 | -1.110937 |
| H | -3.076846 | 4.401815 | -1.649663 |
| C | -1.503203 | -0.962426 | -4.190546 |
| H | -1.880824 | -0.406338 | -5.057845 |
| H | -2.352149 | -1.481417 | -3.730378 |
| H | -0.821470 | -1.731445 | -4.572835 |
| C | 2.130264 | 5.961719 | 2.648227 |
| H | 1.781084 | 6.992261 | 2.644400 |
| C | 3.932532 | -2.477069 | 1.466362 |
| H | 4.420849 | -1.600400 | 1.044386 |
| C | 1.438527 | 5.007522 | 1.938882 |
| H | 0.546321 | 5.295058 | 1.387725 |
| C | -2.391552 | 3.110449 | -0.084976 |
| H | -3.390693 | 2.798252 | 0.211078 |
| C | 3.788321 | -4.162285 | 3.196252 |
| H | 4.169658 | -4.584551 | 4.123491 |
| C | 6.241591 | -2.934841 | -2.066003 |
| H | 6.721027 | -3.911202 | -2.035642 |
| C | -1.687541 | 1.189704 | -2.885715 |
| H | -1.203666 | 1.838151 | -2.147825 |
| H | -2.654873 | 0.891775 | -2.461543 |
| H | -1.882672 | 1.799112 | -3.778436 |
| C | -0.296662 | -2.326795 | 2.826905 |
| H | -0.280215 | -2.830278 | 3.803716 |
| H | 0.739197 | -2.316609 | 2.466774 |
| H | -0.890361 | -2.933738 | 2.137454 |
| C | 0.571920 | 5.547549 | -3.041580 |
| H | 0.723300 | 6.182765 | -3.911718 |
| C | 3.282745 | 5.618278 | 3.383407 |
| H | 3.823157 | 6.384480 | 3.935117 |
| C | 0.108928 | -0.170838 | 3.958051 |
| H | 0.019467 | -0.632756 | 4.950473 |
| H | -0.117201 | 0.894818 | 4.080314 |
| H | 1.156626 | -0.259970 | 3.655399 |
| C | 6.928776 | -1.835953 | -2.621791 |
| H | 7.936868 | -1.966493 | -3.009617 |
| C | -0.831320 | -0.907294 | 3.000889 |
| C | 0.513442 | -4.146271 | -0.379764 |
| H | -0.396630 | -4.540105 | -0.833145 |
| C | 0.467890 | 0.547931 | -3.918151 |
| H | 1.249738 | -0.208443 | -4.055954 |
| H | 0.900726 | 1.388433 | -3.363014 |
| H | 0.199344 | 0.925827 | -4.913154 |
| C | -2.238251 | -0.914577 | 3.603339 |
| H | -2.985907 | -1.343208 | 2.927424 |
| H | -2.569938 | 0.096496 | 3.873576 |
| H | -2.239844 | -1.510615 | 4.525471 |
| Ni | -2.036795 | -1.197259 | -0.334842 |
| H | -1.774963 | -1.991924 | -1.541021 |
| C | -4.130905 | -0.975702 | -0.030541 |
| C | -3.728056 | -1.482519 | -1.284481 |
| N | -4.016800 | -2.890083 | -1.698641 |
| O | -5.022903 | -3.431066 | -1.256055 |
| O | -3.245799 | -3.398088 | -2.510584 |
| H | -3.757599 | -0.852729 | -2.170532 |
| N | -4.480888 | -1.918974 | 0.987468 |
| H | -5.449625 | -1.934266 | 1.287719 |
| C | -3.700383 | -2.997899 | 1.233949 |
| O | -2.558900 | -3.061785 | 0.745245 |
| C | -4.254069 | -4.074332 | 2.103451 |
| H | -4.551713 | -4.920000 | 1.472166 |
| H | -3.475327 | -4.432594 | 2.782973 |
| H | -5.126042 | -3.755502 | 2.681848 |
| C | -4.751094 | 0.351046 | 0.135945 |
| C | -4.986414 | 0.857004 | 1.424930 |
| C | -5.198786 | 1.103512 | -0.961706 |
| C | -5.641474 | 2.069258 | 1.610480 |
| H | -4.648825 | 0.284584 | 2.289883 |
| C | -5.849402 | 2.317193 | -0.774687 |

| | | | |
|---|---|---|---|
| H | -5.075811 | 0.720984 | -1.973656 |
| C | -6.075488 | 2.807179 | 0.510536 |
| H | -5.815151 | 2.438813 | 2.619192 |
| H | -6.199354 | 2.875310 | -1.640865 |
| H | -6.592912 | 3.752959 | 0.654358 |

**TSII$_{R2}$**

| | | | |
|---|---|---|---|
| P | -0.987015 | -0.068203 | 1.300922 |
| P | -0.325995 | -0.873518 | -1.659071 |
| C | 2.376209 | -0.088806 | -1.214835 |
| C | 2.320224 | -2.466065 | -0.373951 |
| C | 4.982335 | -2.774388 | -1.516408 |
| H | 4.456129 | -3.636408 | -1.113128 |
| C | 1.174320 | -3.026903 | -0.925782 |
| C | -0.072264 | 3.048200 | 0.330445 |
| C | 1.084733 | 2.684004 | 1.196779 |
| C | -0.698940 | 0.015832 | -3.265408 |
| C | 2.856800 | -3.014991 | 0.834488 |
| C | 1.496770 | 1.364298 | 1.367790 |
| C | 1.784407 | 3.731170 | 1.888245 |
| C | 2.668912 | 1.082355 | 2.111790 |
| H | 2.993782 | 0.043248 | 2.189260 |
| C | 0.948208 | 0.184054 | -0.791615 |
| H | 0.686283 | 1.193763 | -1.143418 |
| C | -1.352526 | 2.595741 | 0.628012 |
| C | -1.573077 | 1.620776 | 1.740252 |
| H | -2.638897 | 1.552650 | 1.988199 |
| H | -1.037450 | 1.933501 | 2.646262 |
| C | 5.039543 | -0.408381 | -2.118656 |
| C | 3.000914 | -1.323938 | -1.044806 |
| C | 2.256382 | -4.184404 | 1.403586 |
| C | -1.025273 | 4.365108 | -1.540369 |
| C | 0.781643 | 0.186017 | 0.742991 |
| H | 1.273761 | -0.727358 | 1.108619 |
| C | 6.355270 | -0.597324 | -2.604726 |
| H | 6.877382 | 0.257092 | -3.034312 |
| C | 3.649965 | 4.451058 | 3.323959 |
| H | 4.543359 | 4.190900 | 3.890711 |
| C | 0.555270 | -2.408483 | -2.141923 |
| H | -0.174023 | -3.083275 | -2.606007 |
| H | 1.312562 | -2.156016 | -2.897763 |
| C | 2.957545 | 3.418291 | 2.648318 |
| C | 2.783842 | -4.718614 | 2.604186 |
| H | 2.322041 | -5.613536 | 3.020982 |
| C | 1.403190 | 4.297838 | -1.254570 |
| H | 2.279866 | 3.968125 | -0.700033 |
| C | 1.557090 | 5.083810 | -2.373233 |
| H | 2.555965 | 5.364560 | -2.701622 |
| C | 1.118501 | -4.746653 | 0.779005 |
| H | 0.662503 | -5.635003 | 1.215375 |
| C | 4.333554 | -1.512856 | -1.542681 |
| C | 3.390922 | 2.075231 | 2.718078 |
| H | 4.295443 | 1.839048 | 3.277446 |
| C | 4.396365 | 0.847124 | -2.197716 |
| H | 4.939430 | 1.694656 | -2.614459 |
| C | 0.116055 | 3.904624 | -0.804588 |
| C | 4.422901 | -2.953707 | 2.696557 |
| H | 5.256928 | -2.476121 | 3.206958 |
| C | 3.101748 | 0.992259 | -1.775266 |
| H | 2.607604 | 1.961085 | -1.857680 |
| C | -0.831279 | 5.187504 | -2.676513 |
| H | -1.709019 | 5.530501 | -3.223904 |
| C | -2.317310 | 3.956171 | -1.132857 |
| H | -3.186868 | 4.326849 | -1.676358 |
| C | -1.398973 | -0.948704 | -4.222438 |
| H | -1.690441 | -0.403994 | -5.129351 |
| H | -2.308179 | -1.394662 | -3.801675 |
| H | -0.745392 | -1.771416 | -4.535190 |
| C | 2.029553 | 6.058231 | 2.559387 |
| H | 1.662684 | 7.082524 | 2.542318 |
| C | 3.948232 | -2.421337 | 1.519785 |
| H | 4.415689 | -1.530565 | 1.103428 |
| C | 1.342463 | 5.079274 | 1.879906 |
| H | 0.436620 | 5.341454 | 1.338276 |
| C | -2.469140 | 3.069687 | -0.097943 |
| H | -3.459568 | 2.734163 | 0.204147 |
| C | 3.843065 | -4.117804 | 3.241694 |
| H | 4.233249 | -4.535087 | 4.167525 |
| C | 6.258051 | -2.928080 | -2.006273 |
| H | 6.729765 | -3.908324 | -1.979636 |
| C | -1.594849 | 1.207425 | -2.943833 |
| H | -1.136093 | 1.859052 | -2.192626 |
| H | -2.578578 | 0.912792 | -2.554717 |
| H | -1.756716 | 1.812717 | -3.845968 |
| C | -0.281142 | -2.323227 | 2.779943 |
| H | -0.257543 | -2.842700 | 3.748123 |
| H | 0.754203 | -2.294251 | 2.420164 |
| H | -0.866663 | -2.926348 | 2.080198 |

| | | | |
|---|---|---|---|
| C | 0.431324 | 5.536880 | -3.091653 |
| H | 0.565566 | 6.163180 | -3.971015 |
| C | 3.200144 | 5.748059 | 3.280768 |
| H | 3.736775 | 6.533520 | 3.808439 |
| C | 0.090517 | -0.173363 | 3.938353 |
| H | 0.026843 | -0.658213 | 4.921707 |
| H | -0.166344 | 0.881950 | 4.085394 |
| H | 1.137097 | -0.225001 | 3.622926 |
| C | 6.957884 | -1.830376 | -2.548167 |
| H | 7.968099 | -1.965420 | -2.928901 |
| C | -0.839264 | -0.916692 | 2.976292 |
| C | 0.573640 | -4.164130 | -0.337247 |
| H | -0.325367 | -4.578281 | -0.792658 |
| C | 0.584845 | 0.535785 | -3.916629 |
| H | 1.360116 | -0.231865 | -4.029746 |
| H | 1.016720 | 1.373730 | -3.357639 |
| H | 0.345023 | 0.910006 | -4.920257 |
| C | -2.240746 | -0.969238 | 3.586024 |
| H | -2.967259 | -1.461514 | 2.930168 |
| H | -2.622127 | 0.032038 | 3.825266 |
| H | -2.207606 | -1.533855 | 4.527090 |
| Ni | -2.040848 | -1.125331 | -0.368491 |
| H | -2.027452 | -1.750931 | -1.689787 |
| C | -4.073647 | -1.057392 | -0.009120 |
| C | -3.703953 | -1.474429 | -1.318092 |
| N | -3.990571 | -2.879759 | -1.786860 |
| O | -5.036215 | -3.393235 | -1.414968 |
| O | -3.185627 | -3.385053 | -2.563978 |
| H | -3.874503 | -0.807165 | -2.162033 |
| N | -4.379786 | -2.082885 | 0.945511 |
| H | -5.339052 | -2.142222 | 1.270317 |
| C | -3.561357 | -3.151449 | 1.100638 |
| O | -2.439978 | -3.154694 | 0.565895 |
| C | -4.057781 | -4.291881 | 1.924665 |
| H | -4.360796 | -5.107674 | 1.257679 |
| H | -3.244800 | -4.669111 | 2.551872 |
| H | -4.912931 | -4.029617 | 2.554422 |
| C | -4.765049 | 0.225651 | 0.231903 |
| C | -4.984727 | 0.662940 | 1.547693 |
| C | -5.273858 | 1.008396 | -0.816085 |
| C | -5.674100 | 1.842094 | 1.807256 |
| H | -4.603044 | 0.063540 | 2.374612 |
| C | -5.963422 | 2.186934 | -0.556040 |
| H | -5.162123 | 0.685122 | -1.849675 |
| C | -6.165001 | 2.612828 | 0.755351 |
| H | -5.831834 | 2.158088 | 2.836638 |
| H | -6.359092 | 2.768958 | -1.386126 |
| H | -6.709710 | 3.532377 | 0.956693 |

**VI$_{R2}$**

| | | | |
|---|---|---|---|
| P | -0.979279 | -0.072863 | 1.078381 |
| P | -0.105615 | -0.769518 | -1.819248 |
| C | 2.596419 | -0.193504 | -1.136496 |
| C | 2.283179 | -2.588234 | -0.389995 |
| C | 5.069642 | -3.022710 | -1.159601 |
| H | 4.457017 | -3.858109 | -0.829323 |
| C | 1.192867 | -3.041392 | -1.126257 |
| C | 0.132215 | 3.025321 | 0.409653 |
| C | 1.257049 | 2.562348 | 1.269405 |
| C | -0.396989 | 0.176995 | -3.411793 |
| C | 2.595594 | -3.231839 | 0.853240 |
| C | 1.575914 | 1.212872 | 1.409821 |
| C | 1.999682 | 3.542379 | 2.013808 |
| C | 2.680183 | 0.829058 | 2.209614 |
| H | 2.922287 | -0.231717 | 2.282012 |
| C | 1.157219 | 0.175113 | -0.849139 |
| H | 1.009176 | 1.216758 | -1.166198 |
| C | -1.174030 | 2.644413 | 0.700763 |
| C | -1.441162 | 1.588593 | 1.726282 |
| H | -2.497123 | 1.559936 | 2.021680 |
| H | -0.847011 | 1.749746 | 2.637640 |
| C | 5.318993 | -0.663424 | -1.735814 |
| C | 3.120684 | -1.467875 | -0.912728 |
| C | 1.824596 | -4.364757 | 1.274459 |
| C | -0.741069 | 4.478432 | -1.398144 |
| C | 0.845461 | 0.117418 | 0.666419 |
| H | 1.251640 | -0.842581 | 1.009195 |
| C | 6.672667 | -0.919507 | -2.060565 |
| H | 7.284409 | -0.092009 | -2.418523 |
| C | 3.856051 | 4.092515 | 3.534217 |
| H | 4.705468 | 3.755876 | 4.127638 |
| C | 0.744954 | -2.304484 | -2.348334 |
| H | 0.034005 | -2.899234 | -2.933112 |
| H | 1.590648 | -2.035723 | -2.994371 |
| C | 3.114656 | 3.127327 | 2.811525 |
| C | 2.105979 | -4.972867 | 2.521460 |
| H | 1.509327 | -5.832949 | 2.824303 |
| C | 1.678171 | 4.227309 | -1.138185 |

| | | | |
|---|---|---|---|
| H | 2.532170 | 3.814837 | -0.602848 |
| C | 1.878257 | 5.030684 | -2.237554 |
| H | 2.891634 | 5.243864 | -2.572324 |
| C | 0.778400 | -4.836621 | 0.447849 |
| H | 0.210229 | -5.710840 | 0.764175 |
| C | 4.491868 | -1.729387 | -1.251314 |
| C | 3.439980 | 1.753973 | 2.874673 |
| H | 4.295077 | 1.440983 | 3.472586 |
| C | 4.756303 | 0.621631 | -1.900840 |
| H | 5.387310 | 1.432557 | -2.262894 |
| C | 0.369483 | 3.918030 | -0.685777 |
| C | 3.866344 | -3.379195 | 2.925697 |
| H | 4.657540 | -3.001086 | 3.570288 |
| C | 3.432664 | 0.839692 | -1.624695 |
| H | 2.998104 | 1.830112 | -1.771899 |
| C | -0.498779 | 5.322333 | -2.508782 |
| H | -1.353851 | 5.745976 | -3.034911 |
| C | -2.054903 | 4.152130 | -0.990517 |
| H | -2.902311 | 4.611411 | -1.498584 |
| C | -1.479145 | -0.551302 | -4.210816 |
| H | -1.622343 | -0.046376 | -5.174671 |
| H | -2.443554 | -0.546994 | -3.687798 |
| H | -1.223305 | -1.597419 | -4.418823 |
| C | 2.391337 | 5.831450 | 2.745398 |
| H | 2.101439 | 6.880051 | 2.737701 |
| C | 3.625025 | -2.770424 | 1.714558 |
| H | 4.231577 | -1.920942 | 1.405888 |
| C | 1.658198 | 4.919663 | 2.022485 |
| H | 0.793000 | 5.260084 | 1.459399 |
| C | -2.258587 | 3.247131 | 0.019981 |
| H | -3.275785 | 3.011891 | 0.335016 |
| C | 3.101840 | -4.490915 | 3.336109 |
| H | 3.304093 | -4.964196 | 4.294569 |
| C | 6.385296 | -3.240971 | -1.494571 |
| H | 6.799387 | -4.244351 | -1.418332 |
| C | -0.869916 | 1.586563 | -3.066762 |
| H | -0.065921 | 2.197589 | -2.641265 |
| H | -1.706755 | 1.593225 | -2.353690 |
| H | -1.208026 | 2.099472 | -3.977109 |
| C | -1.365581 | -2.607665 | 2.132027 |
| H | -1.353855 | -3.305513 | 2.981402 |
| H | -0.637658 | -2.975014 | 1.399631 |
| H | -2.364453 | -2.649405 | 1.678201 |
| C | 0.782416 | 5.589440 | -2.927625 |
| H | 0.955246 | 6.230674 | -3.789251 |
| C | 3.508394 | 5.420778 | 3.500402 |
| H | 4.082975 | 6.153732 | 4.062731 |
| C | 0.320805 | -1.238966 | 3.349082 |
| H | 0.185773 | -1.821166 | 4.270733 |
| H | 0.672367 | -0.242145 | 3.641200 |
| H | 1.115982 | -1.736149 | 2.780593 |
| C | 7.201323 | -2.181165 | -1.939903 |
| H | 8.241899 | -2.368062 | -2.196306 |
| C | -1.017325 | -1.200510 | 2.610451 |
| C | 0.454190 | -4.171895 | -0.706011 |
| H | -0.381108 | -4.505249 | -1.321649 |
| C | 0.884785 | 0.264903 | -4.242965 |
| H | 1.207727 | -0.710095 | -4.625383 |
| H | 1.718123 | 0.704112 | -3.680287 |
| H | 0.703658 | 0.909055 | -5.113490 |
| C | -2.078467 | -0.694953 | 3.588997 |
| H | -3.053608 | -0.539761 | 3.124044 |
| H | -1.775946 | 0.240995 | 4.072920 |
| H | -2.210150 | -1.442221 | 4.382364 |
| Ni | -1.939645 | -1.019290 | -0.626754 |
| H | -3.706348 | 0.202465 | -2.149280 |
| C | -3.910418 | -0.846615 | -0.249913 |
| C | -4.025224 | 0.387979 | -1.116098 |
| N | -5.469321 | 0.906567 | -1.284160 |
| O | -5.613880 | 2.120038 | -1.315528 |
| O | -6.359405 | 0.069328 | -1.393049 |
| H | -3.474512 | 1.252529 | -0.741772 |
| N | -4.484540 | -1.995562 | -0.967219 |
| H | -5.495658 | -2.099272 | -0.983684 |
| C | -3.713917 | -2.676485 | -1.798452 |
| O | -2.470283 | -2.414340 | -1.816637 |
| C | -4.273130 | -3.713369 | -2.705053 |
| H | -4.221308 | -3.357695 | -3.740776 |
| H | -3.663930 | -4.620157 | -2.649036 |
| H | -5.312154 | -3.961174 | -2.475813 |
| C | -4.565278 | -0.723230 | 1.080438 |
| C | -4.909536 | -1.876266 | 1.804191 |
| C | -4.888172 | 0.520645 | 1.646975 |
| C | -5.521242 | -1.792142 | 3.049140 |
| H | -4.682752 | -2.855165 | 1.382518 |
| C | -5.497628 | 0.606032 | 2.895207 |
| H | -4.684693 | 1.443951 | 1.104832 |
| C | -5.813216 | -0.548899 | 3.605793 |

| | | | |
|---|---|---|---|
| H | -5.772906 | -2.704104 | 3.587152 |
| H | -5.740848 | 1.584204 | 3.305363 |
| H | -6.294588 | -0.480132 | 4.578606 |

**VII$_{R2}$**

| | | | |
|---|---|---|---|
| P | -0.687708 | 0.258037 | 1.358815 |
| P | -0.379064 | -0.453327 | -1.654226 |
| C | 2.463235 | -0.538832 | -1.335159 |
| C | 1.754712 | -2.867680 | -0.685276 |
| C | 4.233008 | -3.802092 | -1.915292 |
| H | 3.491108 | -4.520379 | -1.574881 |
| C | 0.496297 | -3.024031 | -1.257479 |
| C | 1.255588 | 2.991029 | 0.695117 |
| C | 2.249983 | 2.135695 | 1.392631 |
| C | -0.532404 | 0.693700 | -3.121702 |
| C | 2.111944 | -3.663029 | 0.454103 |
| C | 2.178586 | 0.747051 | 1.377697 |
| C | 3.326227 | 2.780176 | 2.096342 |
| C | 3.251194 | -0.008748 | 1.912071 |
| H | 3.203587 | -1.093447 | 1.833075 |
| C | 1.181870 | 0.087986 | -0.823474 |
| H | 1.229924 | 1.160876 | -1.058294 |
| C | -0.082711 | 2.990219 | 1.075381 |
| C | -0.605201 | 1.978394 | 2.050116 |
| H | -1.610241 | 2.245194 | 2.402005 |
| H | 0.042064 | 1.912652 | 2.938013 |
| C | 4.934424 | -1.501371 | -2.328794 |
| C | 2.727024 | -1.905875 | -1.274350 |
| C | 1.193240 | -4.643744 | 0.951017 |
| C | 0.767662 | 4.812841 | -0.912852 |
| C | 1.058539 | -0.023478 | 0.708333 |
| H | 1.203567 | -1.087358 | 0.950838 |
| C | 6.151255 | -2.000118 | -2.851734 |
| H | 6.886506 | -1.286983 | -3.222846 |
| C | 5.447298 | 2.624681 | 3.333469 |
| H | 6.244302 | 2.003787 | 3.741625 |
| C | 0.032639 | -2.108209 | -2.351707 |
| H | -0.863620 | -2.498192 | -2.846932 |
| H | 0.809786 | -1.969777 | -3.116160 |
| C | 4.382208 | 1.990278 | 2.651777 |
| C | 1.525763 | -5.389325 | 2.107535 |
| H | 0.811586 | -6.127112 | 2.472083 |
| C | 3.003356 | 3.829827 | -0.888652 |
| H | 3.727151 | 3.136174 | -0.466073 |
| C | 3.371170 | 4.643007 | -1.936118 |
| H | 4.380550 | 4.579122 | -2.338095 |
| C | -0.032427 | -4.838996 | 0.274305 |
| H | -0.713920 | -5.615524 | 0.621319 |
| C | 3.953285 | -2.413893 | -1.823526 |
| C | 4.329169 | 0.585391 | 2.508857 |
| H | 5.147636 | -0.019054 | 2.898849 |
| C | 4.655208 | -0.117059 | -2.306009 |
| H | 5.405031 | 0.580609 | -2.677415 |
| C | 1.690584 | 3.873379 | -0.350294 |
| C | 3.631505 | -4.232172 | 2.270740 |
| H | 4.579005 | -4.078817 | 2.783744 |
| C | 3.450322 | 0.340599 | -1.843450 |
| H | 3.237221 | 1.409582 | -1.848688 |
| C | 1.183492 | 5.649161 | -1.976615 |
| H | 0.466746 | 6.358417 | -2.389390 |
| C | -0.543715 | 4.874545 | -0.390905 |
| H | -1.229266 | 5.631951 | -0.770946 |
| C | -1.635492 | 0.140786 | -4.025560 |
| H | -1.870560 | 0.870340 | -4.811380 |
| H | -2.560685 | -0.069774 | -3.479184 |
| H | -1.317017 | -0.783873 | -4.523376 |
| C | 4.419711 | 4.771110 | 2.971675 |
| H | 4.428484 | 5.849788 | 3.114631 |
| C | 3.342044 | -3.498943 | 1.142884 |
| H | 4.066264 | -2.777981 | 0.770408 |
| C | 3.378424 | 4.184475 | 2.290101 |
| H | 2.573126 | 4.806818 | 1.906501 |
| C | -0.951892 | 3.979644 | 0.563360 |
| H | -1.956939 | 4.041660 | 0.969746 |
| C | 2.715955 | -5.184930 | 2.762757 |
| H | 2.957634 | -5.759839 | 3.654017 |
| C | 5.420397 | -4.256191 | -2.440205 |
| H | 5.605669 | -5.326700 | -2.502148 |
| C | -0.875165 | 2.078909 | -2.570195 |
| H | -0.025919 | 2.511847 | -2.026921 |
| H | -1.732101 | 2.073588 | -1.887410 |
| H | -1.108298 | 2.766685 | -3.394787 |
| C | -1.631379 | -0.215368 | 3.949831 |
| H | -1.955710 | -0.993951 | 4.651936 |
| H | -2.519003 | 0.222822 | 3.492922 |
| H | -1.151034 | 0.577286 | 4.536203 |
| C | 2.456202 | 5.563589 | -2.486864 |
| H | 2.761153 | 6.204405 | -3.311436 |

| | | | |
|---|---|---|---|
| C | 5.471255 | 3.989235 | 3.490478 |
| H | 6.292119 | 4.467088 | 4.020861 |
| C | -0.993799 | -2.225981 | 2.508157 |
| H | -0.338416 | -2.559706 | 1.692419 |
| H | -2.028099 | -2.310696 | 2.174346 |
| H | -0.852376 | -2.921196 | 3.346829 |
| C | 6.396393 | -3.350429 | -2.903275 |
| H | 7.334001 | -3.723766 | -3.309323 |
| C | -0.647963 | -0.803185 | 2.931553 |
| C | -0.367158 | -4.041745 | -0.789625 |
| H | -1.295250 | -4.218892 | -1.327430 |
| C | 0.764807 | 0.799782 | -3.925159 |
| H | 1.152859 | -0.168711 | -4.263070 |
| H | 1.557598 | 1.308000 | -3.365272 |
| H | 0.571787 | 1.401799 | -4.823169 |
| C | 0.720916 | -0.844690 | 3.613053 |
| H | 1.159360 | 0.146177 | 3.780915 |
| H | 1.446176 | -1.458482 | 3.067997 |
| H | 0.588396 | -1.314425 | 4.597274 |
| Ni | -2.164646 | -0.420371 | -0.254854 |
| H | -5.466730 | -1.661949 | 0.652549 |
| C | -4.053999 | 0.002903 | 0.392392 |
| C | -5.228614 | -0.876679 | -0.068811 |
| N | -6.505614 | -0.052157 | -0.183603 |
| O | -7.092397 | -0.087580 | -1.255576 |
| O | -6.854292 | 0.596476 | 0.801865 |
| H | -5.124155 | -1.308682 | -1.059457 |
| C | -3.749806 | -4.022098 | -1.939148 |
| H | -3.232638 | -3.893587 | -2.891966 |
| H | -3.580449 | -5.023029 | -1.534293 |
| H | -4.828283 | -3.930940 | -2.122622 |
| C | -3.347074 | -2.972011 | -0.963429 |
| O | -2.877766 | -1.886345 | -1.354992 |
| O | -3.587305 | -3.293357 | 0.280112 |
| H | -3.519770 | -2.531362 | 0.931509 |
| N | -4.278189 | 0.348205 | 1.783421 |
| C | -4.475506 | -0.652726 | 2.688522 |
| O | -4.138457 | -1.821483 | 2.457951 |
| C | -5.139849 | -0.256968 | 3.971121 |
| H | -4.909267 | 0.772124 | 4.266019 |
| H | -6.227838 | -0.328835 | 3.846161 |
| H | -4.847666 | -0.943374 | 4.768409 |
| H | -4.855564 | 1.171269 | 1.940571 |
| C | -3.958007 | 1.193182 | -0.494583 |
| C | -4.214370 | 1.059867 | -1.879387 |
| C | -3.781319 | 2.496635 | 0.001122 |
| C | -4.301559 | 2.169355 | -2.709070 |
| H | -4.391346 | 0.075838 | -2.306933 |
| C | -3.883436 | 3.604425 | -0.829428 |
| H | -3.586830 | 2.639630 | 1.063258 |
| C | -4.150113 | 3.451205 | -2.187988 |
| H | -4.509536 | 2.027103 | -3.767933 |
| H | -3.770568 | 4.601532 | -0.407028 |
| H | -4.244908 | 4.323164 | -2.831034 |

**TSIII$_{R2}$**

| | | | |
|---|---|---|---|
| P | -0.677005 | 0.215929 | 1.322629 |
| P | -0.298917 | -0.458933 | -1.680715 |
| C | 2.525224 | -0.526843 | -1.307044 |
| C | 1.815858 | -2.852664 | -0.649906 |
| C | 4.316889 | -3.786194 | -1.833260 |
| H | 3.575509 | -4.504587 | -1.491905 |
| C | 0.567795 | -3.023019 | -1.240034 |
| C | 1.224064 | 2.982780 | 0.677457 |
| C | 2.221439 | 2.153366 | 1.403135 |
| C | -0.464337 | 0.674386 | -3.154396 |
| C | 2.154563 | -3.624371 | 0.510351 |
| C | 2.166664 | 0.762987 | 1.418092 |
| C | 3.275155 | 2.825087 | 2.115922 |
| C | 3.222841 | 0.029078 | 2.011046 |
| H | 3.180973 | -1.057669 | 1.968718 |
| C | 1.234595 | 0.094359 | -0.810759 |
| H | 1.278562 | 1.168832 | -1.040242 |
| C | -0.120662 | 2.958884 | 1.036966 |
| C | -0.638930 | 1.939144 | 2.004153 |
| H | -1.648747 | 2.188445 | 2.352639 |
| H | 0.004752 | 1.875996 | 2.895143 |
| C | 5.009833 | -1.485464 | -2.265540 |
| C | 2.792815 | -1.892595 | -1.233439 |
| C | 1.227023 | -4.594827 | 1.010435 |
| C | 0.724549 | 4.789930 | -0.944192 |
| C | 1.083563 | -0.031497 | 0.718045 |
| H | 1.246848 | -1.092223 | 0.956362 |
| C | 6.234497 | -1.982979 | -2.770794 |
| H | 6.969765 | -1.270158 | -3.142326 |
| C | 5.373014 | 2.718039 | 3.398370 |
| H | 6.165487 | 2.113859 | 3.838828 |
| C | 0.115886 | -2.119802 | -2.348692 |

| | | | | | | | |
|---|---|---|---|---|---|---|---|
| H | -0.773959 | -2.509356 | -2.853224 | H | -1.033536 | 2.742837 | -3.449958 |
| H | 0.902733 | -1.976104 | -3.102740 | C | -1.643906 | -0.189772 | 3.927332 |
| C | 4.324773 | 2.058069 | 2.714563 | H | -1.967042 | -0.936720 | 4.663524 |
| C | 1.538522 | -5.315066 | 2.188992 | H | -2.539201 | 0.256422 | 3.491738 |
| H | 0.818239 | -6.045475 | 2.556017 | H | -1.110936 | 0.599461 | 4.471166 |
| C | 2.975106 | 3.841918 | -0.890445 | C | 2.417976 | 5.557196 | -2.504910 |
| H | 3.704582 | 3.162454 | -0.454887 | H | 2.721629 | 6.197778 | -3.330082 |
| C | 3.341393 | 4.654297 | -1.939049 | C | 5.385528 | 4.086274 | 3.518736 |
| H | 4.355961 | 4.604315 | -2.329692 | H | 6.193562 | 4.583854 | 4.050492 |
| C | 0.015783 | -4.808472 | 0.313617 | C | -1.275313 | -2.231703 | 2.481081 |
| H | -0.670925 | -5.577469 | 0.666131 | H | -0.751162 | -2.624254 | 1.598395 |
| C | 4.028409 | -2.398663 | -1.761566 | H | -2.346129 | -2.217403 | 2.268531 |
| C | 4.281270 | 0.649209 | 2.617294 | H | -1.112092 | -2.947063 | 3.298947 |
| H | 5.090642 | 0.062766 | 3.051040 | C | 6.487443 | -3.332472 | -2.804326 |
| C | 4.723634 | -0.102258 | -2.258717 | H | 7.431028 | -3.704882 | -3.197040 |
| H | 5.473970 | 0.595422 | -2.628860 | C | -0.741081 | -0.861846 | 2.887001 |
| C | 1.655955 | 3.867582 | -0.367217 | C | -0.304286 | -4.032313 | -0.770595 |
| C | 3.639355 | -4.150537 | 2.367595 | H | -1.228241 | -4.214207 | -1.313478 |
| H | 4.576242 | -3.983953 | 2.895873 | C | 0.861826 | 0.820191 | -3.903962 |
| C | 3.512419 | 0.354251 | -1.811572 | H | 1.304301 | -0.136220 | -4.207663 |
| H | 3.294359 | 1.421974 | -1.827385 | H | 1.607711 | 1.371940 | -3.320847 |
| C | 1.138890 | 5.626693 | -2.008200 | H | 0.679495 | 1.398466 | -4.819348 |
| H | 0.416305 | 6.323296 | -2.432090 | C | 0.632660 | -1.050214 | 3.531584 |
| C | -0.593093 | 4.835032 | -0.435754 | H | 1.150347 | -0.108006 | 3.747275 |
| H | -1.284769 | 5.582813 | -0.823360 | H | 1.297225 | -1.690968 | 2.939759 |
| C | -1.520252 | 0.089931 | -4.093045 | H | 0.477119 | -1.562816 | 4.490470 |
| H | -1.718470 | 0.804688 | -4.902157 | Ni | -2.058431 | -0.459875 | -0.355826 |
| H | -2.468848 | -0.112758 | -3.585870 | H | -5.621251 | -1.741645 | 0.772649 |
| H | -1.177191 | -0.842483 | -4.558281 | C | -4.110684 | -0.207570 | 0.414507 |
| C | 4.336958 | 4.845933 | 2.962455 | C | -5.368761 | -1.009180 | 0.001156 |
| H | 4.334005 | 5.927771 | 3.078818 | N | -6.569818 | -0.106682 | -0.156955 |
| C | 3.370456 | -3.441415 | 1.219334 | O | -7.171309 | -0.164336 | -1.220405 |
| H | 4.099288 | -2.724921 | 0.846948 | O | -6.861296 | 0.621763 | 0.791027 |
| C | 3.312213 | 4.234075 | 2.278116 | H | -5.273171 | -1.503240 | -0.963398 |
| H | 2.508731 | 4.841419 | 1.868318 | C | -3.678594 | -3.961520 | -1.854932 |
| C | -0.999756 | 3.934185 | 0.513586 | H | -3.170921 | -4.000393 | -2.821665 |
| H | -2.009664 | 3.984548 | 0.910885 | H | -3.532430 | -4.892315 | -1.301253 |
| C | 2.716187 | -5.094898 | 2.861715 | H | -4.755328 | -3.867707 | -2.045381 |
| H | 2.941939 | -5.650447 | 3.769334 | C | -3.234572 | -2.787476 | -1.046293 |
| C | 5.512537 | -4.238972 | -2.340205 | O | -2.779818 | -1.759672 | -1.651092 |
| H | 5.705430 | -5.308824 | -2.387218 | O | -3.395530 | -2.807872 | 0.211741 |
| C | -0.881705 | 2.041489 | -2.618217 | H | -3.389502 | -1.533732 | 0.498068 |
| H | -0.107916 | 2.473430 | -1.971789 | N | -4.278647 | 0.307281 | 1.763553 |
| H | -1.810849 | 1.998640 | -2.041447 | C | -4.644296 | -0.546689 | 2.789576 |

| | | | |
|---|---|---|---|
| O | -4.464440 | -1.754509 | 2.720663 |
| C | -5.266419 | 0.136477 | 3.974599 |
| H | -4.841300 | 1.128949 | 4.161637 |
| H | -6.339988 | 0.267486 | 3.789966 |
| H | -5.147525 | -0.487751 | 4.862300 |
| H | -4.790897 | 1.185197 | 1.789161 |
| C | -3.940484 | 0.942137 | -0.563466 |
| C | -4.285149 | 0.788970 | -1.926252 |
| C | -3.714195 | 2.254639 | -0.111285 |
| C | -4.415167 | 1.885272 | -2.767911 |
| H | -4.475735 | -0.200759 | -2.331283 |
| C | -3.861487 | 3.351026 | -0.952771 |
| H | -3.435794 | 2.413883 | 0.927974 |
| C | -4.228092 | 3.176919 | -2.283144 |
| H | -4.692370 | 1.723163 | -3.807794 |
| H | -3.706769 | 4.352919 | -0.556739 |
| H | -4.366819 | 4.036783 | -2.934453 |

**VIII$_{R2}$**

| | | | |
|---|---|---|---|
| P | -0.076704 | -0.396561 | 1.631249 |
| P | 0.038236 | -1.053715 | -1.329777 |
| C | 2.600680 | 0.153958 | -1.529387 |
| C | 3.121113 | -2.187144 | -0.740482 |
| C | 5.499648 | -2.059375 | -2.425677 |
| H | 5.236687 | -2.987513 | -1.924366 |
| C | 1.988347 | -2.935092 | -1.044168 |
| C | 0.074991 | 2.837748 | 0.700825 |
| C | 1.422947 | 2.651615 | 1.310475 |
| C | -0.953103 | -0.305080 | -2.713225 |
| C | 3.988048 | -2.632231 | 0.310825 |
| C | 2.087367 | 1.427427 | 1.301155 |
| C | 2.051859 | 3.784422 | 1.932619 |
| C | 3.413308 | 1.339542 | 1.791816 |
| H | 3.924481 | 0.377746 | 1.731377 |
| C | 1.288630 | 0.198047 | -0.780223 |
| H | 0.805780 | 1.158125 | -1.015963 |
| C | -1.039878 | 2.172396 | 1.201305 |
| C | -0.888973 | 1.121630 | 2.254753 |
| H | -1.857429 | 0.820729 | 2.668367 |
| H | -0.257567 | 1.480461 | 3.082247 |
| C | 5.003467 | 0.257278 | -3.015714 |
| C | 3.444120 | -0.956883 | -1.518564 |
| C | 3.728856 | -3.882890 | 0.959597 |
| C | -1.408031 | 4.076211 | -0.847151 |
| C | 1.471931 | 0.159261 | 0.758759 |
| H | 2.166636 | -0.667383 | 0.970935 |
| C | 6.197152 | 0.284470 | -3.776384 |
| H | 6.453393 | 1.201251 | -4.306451 |
| C | 4.007258 | 4.779579 | 3.049897 |
| H | 5.024927 | 4.666511 | 3.422388 |
| C | 0.998870 | -2.434068 | -2.053561 |
| H | 0.290493 | -3.219892 | -2.340141 |
| H | 1.494565 | -2.068633 | -2.964426 |
| C | 3.386751 | 3.664106 | 2.439196 |
| C | 4.574649 | -4.306314 | 2.013226 |
| H | 4.365477 | -5.261218 | 2.494783 |
| C | 1.012996 | 4.347494 | -1.058460 |
| H | 2.022584 | 4.117079 | -0.721345 |
| C | 0.822953 | 5.196105 | -2.124952 |
| H | 1.684443 | 5.629212 | -2.629662 |
| C | 2.604001 | -4.644222 | 0.563594 |
| H | 2.416061 | -5.600202 | 1.051496 |
| C | 4.646871 | -0.932313 | -2.301805 |
| C | 4.053973 | 2.424040 | 2.326941 |
| H | 5.077746 | 2.339295 | 2.689624 |
| C | 4.145713 | 1.377588 | -2.953834 |
| H | 4.423412 | 2.285917 | -3.487303 |
| C | -0.089142 | 3.757524 | -0.387294 |
| C | 5.879797 | -2.291298 | 1.803492 |
| H | 6.712823 | -1.676155 | 2.138072 |
| C | 2.973823 | 1.317435 | -2.246154 |
| H | 2.311237 | 2.184079 | -2.210246 |
| C | -1.568537 | 4.968523 | -1.934355 |
| H | -2.579747 | 5.198944 | -2.270201 |
| C | -2.519127 | 3.460236 | -0.226814 |
| H | -3.520019 | 3.725103 | -0.564204 |
| C | -1.867612 | -1.390763 | -3.282282 |
| H | -2.510134 | -0.949856 | -4.055334 |
| H | -2.519806 | -1.823850 | -2.513774 |
| H | -1.307815 | -2.210717 | -3.747654 |
| C | 2.017347 | 6.090411 | 2.712058 |
| H | 1.483114 | 7.030337 | 2.835975 |
| C | 5.087675 | -1.859212 | 0.764508 |
| H | 5.303249 | -0.908042 | 0.281159 |
| C | 1.391283 | 5.028575 | 2.101449 |
| H | 0.366089 | 5.140161 | 1.756084 |
| C | -2.336547 | 2.504242 | 0.739963 |
| H | -3.192124 | 1.998342 | 1.190862 |

| | | | |
|---|---|---|---|
| C | 5.626475 | -3.527609 | 2.432793 |
| H | 6.262946 | -3.860413 | 3.249896 |
| C | 6.648532 | -2.003554 | -3.179422 |
| H | 7.282114 | -2.884527 | -3.258492 |
| C | -1.778923 | 0.826151 | -2.114061 |
| H | -1.141752 | 1.620275 | -1.709046 |
| H | -2.432249 | 0.475705 | -1.304918 |
| H | -2.423163 | 1.281269 | -2.879057 |
| C | -0.760233 | -1.397573 | 4.092658 |
| H | -0.510111 | -2.039654 | 4.947079 |
| H | -1.627900 | -1.831056 | 3.585925 |
| H | -1.057851 | -0.422195 | 4.494652 |
| C | -0.477971 | 5.516770 | -2.565384 |
| H | -0.613619 | 6.195281 | -3.404932 |
| C | 3.340529 | 5.972623 | 3.183658 |
| H | 3.825005 | 6.822103 | 3.660322 |
| C | 0.964899 | -2.669399 | 2.767207 |
| H | 1.783793 | -2.592189 | 2.041723 |
| H | 0.170407 | -3.280138 | 2.322207 |
| H | 1.356876 | -3.205917 | 3.641990 |
| C | 7.009740 | -0.819885 | -3.854852 |
| H | 7.923338 | -0.788789 | -4.444560 |
| C | 0.463196 | -1.289628 | 3.179160 |
| C | 1.737857 | -4.163199 | -0.385372 |
| H | 0.851759 | -4.733579 | -0.665314 |
| C | -0.041266 | 0.252734 | -3.805868 |
| H | 0.619569 | -0.504989 | -4.243260 |
| H | 0.582092 | 1.078822 | -3.442453 |
| H | -0.663671 | 0.649568 | -4.618663 |
| C | 1.594116 | -0.550598 | 3.895317 |
| H | 1.379457 | 0.508623 | 4.084562 |
| H | 2.539204 | -0.611049 | 3.342843 |
| H | 1.759284 | -1.028394 | 4.870087 |
| Ni | -1.081181 | -1.781941 | 0.326346 |
| H | -4.579326 | -2.227762 | 1.080998 |
| C | -4.792363 | -0.313633 | 0.139334 |
| C | -4.872359 | -1.826170 | 0.104323 |
| N | -6.252090 | -2.368272 | -0.161828 |
| O | -6.318335 | -3.560147 | -0.439626 |
| O | -7.202556 | -1.600439 | -0.064433 |
| H | -4.227129 | -2.276353 | -0.657197 |
| C | -3.160234 | -4.901279 | 0.671546 |
| H | -2.751940 | -5.700239 | 0.046681 |
| H | -3.232998 | -5.225761 | 1.711669 |
| H | -4.173986 | -4.690287 | 0.299904 |
| C | -2.342055 | -3.672075 | 0.552100 |
| O | -1.847049 | -3.309116 | -0.576720 |
| O | -2.140025 | -2.887242 | 1.538390 |
| H | -3.720812 | -0.114869 | 0.338299 |
| N | -5.481153 | 0.223613 | 1.300084 |
| C | -4.897001 | 0.184941 | 2.533143 |
| O | -3.731312 | -0.186466 | 2.679891 |
| C | -5.751619 | 0.641450 | 3.681484 |
| H | -6.746338 | 0.985521 | 3.383255 |
| H | -5.862378 | -0.178631 | 4.398011 |
| H | -5.241050 | 1.452868 | 4.209036 |
| H | -6.482892 | 0.343272 | 1.216067 |
| C | -5.186128 | 0.399916 | -1.133681 |
| C | -5.256086 | -0.258345 | -2.364178 |
| C | -5.408532 | 1.779352 | -1.101237 |
| C | -5.531017 | 0.447541 | -3.532838 |
| H | -5.107137 | -1.336455 | -2.428688 |
| C | -5.682690 | 2.486286 | -2.267316 |
| H | -5.370599 | 2.300452 | -0.144833 |
| C | -5.741470 | 1.822703 | -3.490066 |
| H | -5.592913 | -0.086557 | -4.479236 |
| H | -5.859172 | 3.559893 | -2.218312 |
| H | -5.963647 | 2.371707 | -4.402584 |

**$IV_{R3}$**

| | | | |
|---|---|---|---|
| P | 0.643542 | -0.249235 | -1.574875 |
| P | 0.367244 | -0.744041 | 1.562617 |
| C | -2.344741 | 0.104507 | 1.395763 |
| C | -2.545016 | -2.325490 | 0.764183 |
| C | -5.098319 | -2.320244 | 2.200813 |
| H | -4.698559 | -3.252141 | 1.808559 |
| C | -1.374349 | -2.903864 | 1.247630 |
| C | -0.226071 | 2.986064 | -0.747784 |
| C | -1.495625 | 2.521975 | -1.368448 |
| C | 0.883573 | 0.245299 | 3.083932 |
| C | -3.238614 | -2.955340 | -0.322313 |
| C | -1.880336 | 1.182553 | -1.369922 |
| C | -2.348529 | 3.501972 | -1.986456 |
| C | -3.170351 | 0.832286 | -1.838967 |
| H | -3.470557 | -0.212717 | -1.786907 |
| C | -0.981968 | 0.247217 | 0.756914 |
| H | -0.649849 | 1.278057 | 0.928892 |
| C | 0.999836 | 2.519720 | -1.210211 |

| | | | |
|---|---|---|---|
| C | 1.070021 | 1.431180 | -2.233489 |
| H | 2.059208 | 1.404855 | -2.706521 |
| H | 0.339011 | 1.616151 | -3.034915 |
| C | -4.888080 | 0.073567 | 2.629131 |
| C | -3.075180 | -1.082459 | 1.391770 |
| C | -2.743432 | -4.188631 | -0.856406 |
| C | 0.974565 | 4.512187 | 0.795169 |
| C | -1.036674 | 0.067675 | -0.777538 |
| H | -1.562177 | -0.879957 | -0.968825 |
| C | -6.149883 | 0.037743 | 3.268574 |
| H | -6.543872 | 0.962737 | 3.688162 |
| C | -4.498338 | 4.087330 | -3.032320 |
| H | -5.485318 | 3.773281 | -3.370570 |
| C | -0.530348 | -2.199853 | 2.267019 |
| H | 0.201358 | -2.881549 | 2.719917 |
| H | -1.154537 | -1.803656 | 3.078525 |
| C | -3.645166 | 3.117150 | -2.455104 |
| C | -3.381139 | -4.770625 | -1.977664 |
| H | -2.985062 | -5.706116 | -2.371560 |
| C | -1.460054 | 4.384247 | 0.923977 |
| H | -2.407134 | 3.985475 | 0.567332 |
| C | -1.450610 | 5.282213 | 1.966810 |
| H | -2.390387 | 5.581999 | 2.426717 |
| C | -1.629324 | -4.804743 | -0.241936 |
| H | -1.299729 | -5.779541 | -0.601176 |
| C | -4.348479 | -1.123845 | 2.056232 |
| C | -4.037668 | 1.765251 | -2.338590 |
| H | -5.032379 | 1.471200 | -2.672449 |
| C | -4.132833 | 1.266054 | 2.564967 |
| H | -4.544518 | 2.177876 | 2.996728 |
| C | -0.254108 | 3.959690 | 0.307098 |
| C | -4.981201 | -2.971717 | -2.024697 |
| H | -5.853702 | -2.505356 | -2.478391 |
| C | -2.892674 | 1.269800 | 1.985573 |
| H | -2.303244 | 2.187351 | 1.963972 |
| C | 0.948363 | 5.448300 | 1.856803 |
| H | 1.894304 | 5.860092 | 2.208279 |
| C | 2.189095 | 4.102923 | 0.200993 |
| H | 3.120450 | 4.573476 | 0.520274 |
| C | 2.139544 | -0.383093 | 3.688970 |
| H | 2.387785 | 0.138257 | 4.623302 |
| H | 3.007279 | -0.303692 | 3.025932 |
| H | 1.993959 | -1.443201 | 3.939457 |
| C | -2.795753 | 5.766299 | -2.760945 |

| | | | |
|---|---|---|---|
| H | -2.459506 | 6.791199 | -2.904621 |
| C | -4.386760 | -2.384236 | -0.931068 |
| H | -4.796833 | -1.460602 | -0.527912 |
| C | -1.951085 | 4.850445 | -2.178118 |
| H | -0.954813 | 5.162023 | -1.872992 |
| C | 2.195939 | 3.124313 | -0.759939 |
| H | 3.126031 | 2.835802 | -1.242262 |
| C | -4.472717 | -4.173050 | -2.559828 |
| H | -4.950693 | -4.626980 | -3.425289 |
| C | -6.316157 | -2.324707 | 2.839897 |
| H | -6.866051 | -3.258383 | 2.940215 |
| C | 1.174314 | 1.678988 | 2.635927 |
| H | 0.255475 | 2.266611 | 2.514258 |
| H | 1.729165 | 1.723877 | 1.688646 |
| H | 1.779127 | 2.190875 | 3.396563 |
| C | 0.261461 | -2.683871 | -2.928170 |
| H | -0.161128 | -3.204997 | -3.798031 |
| H | -0.325704 | -2.976395 | -2.047679 |
| H | 1.282257 | -3.047307 | -2.778825 |
| C | -0.237620 | 5.824043 | 2.439166 |
| H | -0.245129 | 6.538029 | 3.259997 |
| C | -4.088132 | 5.389617 | -3.179221 |
| H | -4.750038 | 6.126655 | -3.628863 |
| C | -1.173435 | -0.817783 | -3.702288 |
| H | -1.296831 | -1.274202 | -4.693455 |
| H | -1.330130 | 0.261132 | -3.819661 |
| H | -1.974675 | -1.217076 | -3.068729 |
| C | -6.856268 | -1.135626 | 3.370549 |
| H | -7.823512 | -1.153517 | 3.868352 |
| C | 0.217731 | -1.179741 | -3.183682 |
| C | -0.970158 | -4.174600 | 0.779859 |
| H | -0.122676 | -4.655093 | 1.267179 |
| C | -0.207035 | 0.267925 | 4.160612 |
| H | -0.313702 | -0.698725 | 4.665244 |
| H | -1.188086 | 0.568202 | 3.775178 |
| H | 0.077650 | 0.998207 | 4.929233 |
| C | 1.233786 | -0.806338 | -4.266520 |
| H | 2.269742 | -0.947496 | -3.948958 |
| H | 1.108327 | 0.223886 | -4.618269 |
| H | 1.075287 | -1.461257 | -5.132724 |
| Ni | 1.877518 | -1.232775 | 0.106658 |
| H | 2.324547 | -1.591094 | 1.491039 |
| C | 4.233012 | -0.370970 | -0.326245 |
| N | 4.243785 | 0.453440 | -1.416098 |

| | | | |
|---|---|---|---|
| H | 3.758078 | 0.066251 | -2.225384 |
| C | 5.096170 | 1.512529 | -1.820684 |
| O | 4.806406 | 2.062983 | -2.864431 |
| C | 6.300409 | 1.872638 | -1.006717 |
| H | 6.727591 | 1.031813 | -0.454042 |
| H | 7.042269 | 2.284647 | -1.694284 |
| H | 6.054832 | 2.654775 | -0.279414 |
| C | 3.834162 | -1.719988 | -0.444102 |
| H | 4.134724 | -2.380707 | 0.361331 |
| N | 3.944160 | -2.440254 | -1.692231 |
| O | 3.808822 | -1.836049 | -2.766948 |
| O | 4.142624 | -3.651078 | -1.609297 |
| C | 4.725896 | 0.114530 | 0.969371 |
| C | 5.509078 | -0.704417 | 1.798276 |
| C | 4.414224 | 1.412653 | 1.397084 |
| C | 5.972399 | -0.225728 | 3.018717 |
| H | 5.793343 | -1.702795 | 1.469000 |
| C | 4.867893 | 1.881927 | 2.621293 |
| H | 3.778934 | 2.037409 | 0.772419 |
| C | 5.653853 | 1.065837 | 3.434949 |
| H | 6.594456 | -0.862499 | 3.643281 |
| H | 4.599658 | 2.887137 | 2.943854 |
| H | 6.017862 | 1.435021 | 4.391159 |
| C | 2.109527 | -4.286878 | 0.185598 |
| H | 1.762213 | -5.710997 | -1.347796 |
| H | 3.211988 | -6.019103 | -0.351407 |
| H | 1.616429 | -6.353461 | 0.319522 |
| O | 2.634148 | -4.142801 | 1.405655 |
| H | 2.533824 | -3.179789 | 1.615079 |
| O | 1.599472 | -3.346707 | -0.417383 |
| C | 2.173332 | -5.675846 | -0.337699 |

**$V_{R3}$**

| | | | |
|---|---|---|---|
| P | 0.705688 | -0.394329 | -1.489305 |
| P | 0.344262 | -0.790241 | 1.679060 |
| C | -2.319226 | 0.186864 | 1.390749 |
| C | -2.551089 | -2.241566 | 0.743940 |
| C | -5.174180 | -2.162612 | 2.054471 |
| H | -4.779452 | -3.104992 | 1.682636 |
| C | -1.405621 | -2.837456 | 1.261745 |
| C | 0.397489 | 2.878682 | -0.714446 |
| C | -0.935840 | 2.637275 | -1.335597 |
| C | 0.967626 | 0.153404 | 3.170005 |
| C | -3.209681 | -2.858517 | -0.370110 |
| C | -1.556117 | 1.390381 | -1.318867 |
| C | -1.594690 | 3.741523 | -1.978302 |
| C | -2.881509 | 1.259784 | -1.802633 |
| H | -3.367096 | 0.286327 | -1.721251 |
| C | -0.917876 | 0.291674 | 0.830840 |
| H | -0.551584 | 1.303702 | 1.053037 |
| C | 1.534861 | 2.238966 | -1.193927 |
| C | 1.417288 | 1.148159 | -2.207673 |
| H | 2.393292 | 0.906722 | -2.649653 |
| H | 0.750024 | 1.446736 | -3.030199 |
| C | -4.922473 | 0.226905 | 2.487587 |
| C | -3.079734 | -0.983935 | 1.349810 |
| C | -2.731002 | -4.115688 | -0.864800 |
| C | 1.834574 | 4.187411 | 0.823388 |
| C | -0.916498 | 0.158455 | -0.713236 |
| H | -1.567095 | -0.698689 | -0.942812 |
| C | -6.214267 | 0.225719 | 3.065569 |
| H | -6.604379 | 1.161798 | 3.463436 |
| C | -3.574397 | 4.663055 | -3.113803 |
| H | -4.587221 | 4.515177 | -3.487230 |
| C | -0.650590 | -2.166039 | 2.366470 |
| H | 0.018929 | -2.863758 | 2.881966 |
| H | -1.337513 | -1.737388 | 3.106958 |
| C | -2.923177 | 3.574973 | -2.485765 |
| C | -3.365833 | -4.708452 | -1.982337 |
| H | -2.988657 | -5.663684 | -2.346226 |
| C | -0.594790 | 4.402976 | 0.998135 |
| H | -1.593747 | 4.146091 | 0.648730 |
| C | -0.440392 | 5.270550 | 2.055533 |
| H | -1.319210 | 5.688876 | 2.542504 |
| C | -1.589959 | -4.705562 | -0.270871 |
| H | -1.222374 | -5.654971 | -0.658391 |
| C | -4.386637 | -0.986789 | 1.945957 |
| C | -3.554189 | 2.317667 | -2.351869 |
| H | -4.576798 | 2.198233 | -2.708046 |
| C | -4.138167 | 1.401334 | 2.452636 |
| H | -4.551713 | 2.326022 | 2.853280 |
| C | 0.529700 | 3.829195 | 0.350285 |
| C | -4.886212 | -2.844591 | -2.134577 |
| H | -5.718072 | -2.353423 | -2.635464 |
| C | -2.869978 | 1.371842 | 1.937421 |
| H | -2.260036 | 2.277189 | 1.928357 |
| C | 1.957825 | 5.104333 | 1.894631 |
| H | 2.957808 | 5.375484 | 2.233220 |

| | | | |
|---|---|---|---|
| C | 2.969130 | 3.597466 | 0.220176 |
| H | 3.963018 | 3.913592 | 0.538924 |
| C | 2.068491 | -0.672818 | 3.834141 |
| H | 2.413059 | -0.153917 | 4.738544 |
| H | 2.934112 | -0.808570 | 3.177622 |
| H | 1.714023 | -1.664671 | 4.143281 |
| C | -1.624536 | 6.036314 | -2.790767 |
| H | -1.116598 | 6.988795 | -2.928275 |
| C | -4.303998 | -2.250818 | -1.037830 |
| H | -4.688367 | -1.300884 | -0.670588 |
| C | -0.969632 | 5.001580 | -2.163914 |
| H | 0.052101 | 5.147461 | -1.821232 |
| C | 2.817423 | 2.634145 | -0.746042 |
| H | 3.689223 | 2.193216 | -1.231467 |
| C | -4.417498 | -4.086102 | -2.611636 |
| H | -4.889257 | -4.547409 | -3.476609 |
| C | -6.421267 | -2.133417 | 2.633612 |
| H | -7.000614 | -3.051498 | 2.707338 |
| C | 1.514805 | 1.498408 | 2.695813 |
| H | 0.711480 | 2.208928 | 2.467592 |
| H | 2.146731 | 1.408331 | 1.802406 |
| H | 2.124726 | 1.952116 | 3.488668 |
| C | -0.154703 | -2.745824 | -2.643207 |
| H | -0.650851 | -3.274364 | -3.468164 |
| H | -0.843458 | -2.760873 | -1.791649 |
| H | 0.733599 | -3.322644 | -2.373893 |
| C | 0.846196 | 5.632947 | 2.505344 |
| H | 0.954691 | 6.330672 | 3.332938 |
| C | -2.942294 | 5.873442 | -3.262960 |
| H | -3.449986 | 6.701338 | -3.752999 |
| C | -1.101370 | -0.703398 | -3.664642 |
| H | -1.273948 | -1.186158 | -4.635693 |
| H | -1.024933 | 0.375777 | -3.846422 |
| H | -1.994741 | -0.886792 | -3.056869 |
| C | -6.954753 | -0.928863 | 3.135712 |
| H | -7.945186 | -0.919738 | 3.585809 |
| C | 0.159297 | -1.311608 | -3.046683 |
| C | -0.923433 | -4.061691 | 0.740455 |
| H | -0.022455 | -4.500915 | 1.171463 |
| C | -0.158349 | 0.396567 | 4.178856 |
| H | -0.493333 | -0.527011 | 4.664180 |
| H | -1.031635 | 0.887079 | 3.732129 |
| H | 0.217432 | 1.057793 | 4.970217 |
| C | 1.270559 | -1.237421 | -4.095608 |
| H | 2.251693 | -1.544930 | -3.724610 |
| H | 1.362757 | -0.232462 | -4.522574 |
| H | 1.027551 | -1.920354 | -4.919490 |
| Ni | 1.765379 | -1.458270 | 0.218597 |
| H | 1.966115 | -1.978228 | 1.595725 |
| C | 3.889642 | -1.656862 | -0.313873 |
| N | 4.124677 | -0.983043 | -1.484461 |
| H | 3.550323 | -1.292323 | -2.267155 |
| C | 5.117889 | -0.066003 | -1.901736 |
| O | 4.885341 | 0.556319 | -2.920765 |
| C | 6.413813 | 0.041341 | -1.156890 |
| H | 6.646680 | -0.828686 | -0.538522 |
| H | 7.197816 | 0.185645 | -1.904815 |
| H | 6.417011 | 0.926631 | -0.512044 |
| C | 3.136169 | -2.856561 | -0.274546 |
| H | 3.206486 | -3.479332 | 0.609821 |
| N | 2.933629 | -3.703802 | -1.430337 |
| O | 3.118665 | -3.243167 | -2.566634 |
| O | 2.549582 | -4.846581 | -1.202675 |
| C | 4.504526 | -1.159621 | 0.927461 |
| C | 5.108052 | -2.036250 | 1.841826 |
| C | 4.491876 | 0.215444 | 1.204402 |
| C | 5.707831 | -1.536412 | 2.991221 |
| H | 5.143192 | -3.103407 | 1.628753 |
| C | 5.080899 | 0.708349 | 2.360577 |
| H | 3.992916 | 0.893936 | 0.512813 |
| C | 5.700156 | -0.165777 | 3.252579 |
| H | 6.193812 | -2.220145 | 3.683335 |
| H | 5.049002 | 1.777170 | 2.566650 |
| H | 6.173049 | 0.217957 | 4.153989 |

**TSII$_{R3}$**

| | | | |
|---|---|---|---|
| P | 0.449637 | 0.028554 | -1.678958 |
| P | 0.539037 | -0.736077 | 1.379838 |
| C | -2.292348 | -0.497839 | 1.447119 |
| C | -1.939000 | -2.794599 | 0.466703 |
| C | -4.285205 | -3.635890 | 1.983952 |
| H | -3.695370 | -4.368324 | 1.437383 |
| C | -0.634766 | -3.144130 | 0.797853 |
| C | -0.583586 | 2.977460 | -0.403476 |
| C | -1.874275 | 2.506257 | -0.981335 |
| C | 1.025463 | 0.126692 | 2.963192 |
| C | -2.546124 | -3.385662 | -0.689080 |
| C | -2.144799 | 1.151677 | -1.171313 |

| | | | |
|---|---|---|---|
| C | -2.840135 | 3.481547 | -1.403368 |
| C | -3.394562 | 0.746939 | -1.699857 |
| H | -3.587067 | -0.320983 | -1.813900 |
| C | -1.040731 | 0.046082 | 0.792409 |
| H | -0.916688 | 1.090642 | 1.117075 |
| C | 0.612245 | 2.700072 | -1.056521 |
| C | 0.628560 | 1.775373 | -2.231904 |
| H | 1.553945 | 1.873568 | -2.806332 |
| H | -0.217824 | 1.971004 | -2.905991 |
| C | -4.650395 | -1.346953 | 2.755692 |
| C | -2.699278 | -1.823855 | 1.304733 |
| C | -1.831971 | -4.384809 | -1.427259 |
| C | 0.683351 | 4.329103 | 1.241523 |
| C | -1.182039 | 0.063135 | -0.750233 |
| H | -1.632180 | -0.898382 | -1.024565 |
| C | -5.807248 | -1.795976 | 3.436254 |
| H | -6.388974 | -1.070806 | 4.004282 |
| C | -5.059830 | 4.007056 | -2.329719 |
| H | -6.014335 | 3.654208 | -2.718620 |
| C | 0.075144 | -2.432484 | 1.905725 |
| H | 0.986719 | -2.960975 | 2.207271 |
| H | -0.561170 | -2.327173 | 2.797161 |
| C | -4.100504 | 3.046532 | -1.928224 |
| C | -2.412587 | -4.932421 | -2.596801 |
| H | -1.857700 | -5.693882 | -3.144288 |
| C | -1.706550 | 3.944373 | 1.605141 |
| H | -2.656557 | 3.530208 | 1.271002 |
| C | -1.638771 | 4.641551 | 2.790042 |
| H | -2.533900 | 4.763133 | 3.397050 |
| C | -0.546166 | -4.778919 | -0.989524 |
| H | -0.019037 | -5.559752 | -1.536649 |
| C | -3.871585 | -2.278872 | 1.995918 |
| C | -4.351813 | 1.661696 | -2.051423 |
| H | -5.314201 | 1.331908 | -2.440978 |
| C | -4.238903 | 0.003695 | 2.813689 |
| H | -4.844514 | 0.716854 | 3.371635 |
| C | -0.554282 | 3.756412 | 0.799334 |
| C | -4.345963 | -3.519496 | -2.322067 |
| H | -5.317907 | -3.183442 | -2.677877 |
| C | -3.089034 | 0.407728 | 2.189180 |
| H | -2.770626 | 1.449170 | 2.247949 |
| C | 0.711881 | 5.060070 | 2.454238 |
| H | 1.659456 | 5.490381 | 2.777152 |
| C | 1.858183 | 4.104881 | 0.483916 |
| H | 2.798573 | 4.553061 | 0.802482 |
| C | 1.910112 | -0.823751 | 3.770615 |
| H | 2.385159 | -0.269379 | 4.590096 |
| H | 2.716815 | -1.261742 | 3.164918 |
| H | 1.339169 | -1.644696 | 4.219672 |
| C | -3.539887 | 5.785062 | -1.760980 |
| H | -3.318698 | 6.849508 | -1.717239 |
| C | -3.817975 | -2.980227 | -1.170854 |
| H | -4.377675 | -2.225266 | -0.621496 |
| C | -2.591238 | 4.877678 | -1.351927 |
| H | -1.629307 | 5.236418 | -0.994654 |
| C | 1.826425 | 3.285234 | -0.617856 |
| H | 2.732284 | 3.119919 | -1.202256 |
| C | -3.641350 | -4.506556 | -3.042044 |
| H | -4.071994 | -4.927630 | -3.947967 |
| C | -5.409827 | -4.042085 | 2.663285 |
| H | -5.701143 | -5.090074 | 2.643124 |
| C | 1.805014 | 1.376628 | 2.571406 |
| H | 1.263072 | 1.959417 | 1.817850 |
| H | 2.791440 | 1.133151 | 2.156699 |
| H | 1.954378 | 2.028172 | 3.442227 |
| C | 0.868891 | -2.324404 | -3.105476 |
| H | 0.573629 | -2.987835 | -3.929797 |
| H | 0.578818 | -2.816909 | -2.169266 |
| H | 1.961153 | -2.236171 | -3.135892 |
| C | -0.420252 | 5.207538 | 3.219486 |
| H | -0.381364 | 5.763518 | 4.153817 |
| C | -4.791129 | 5.350941 | -2.244283 |
| H | -5.533556 | 6.080505 | -2.560641 |
| C | -1.294526 | -1.167254 | -3.576173 |
| H | -1.385241 | -1.675335 | -4.545270 |
| H | -1.843091 | -0.220343 | -3.649428 |
| H | -1.798372 | -1.804896 | -2.839037 |
| C | -6.184909 | -3.115856 | 3.390861 |
| H | -7.074125 | -3.450125 | 3.920953 |
| C | 0.190937 | -0.964515 | -3.273522 |
| C | 0.048188 | -4.150543 | 0.075407 |
| H | 1.054095 | -4.432830 | 0.388732 |
| C | -0.195793 | 0.530007 | 3.788743 |
| H | -0.894984 | -0.297250 | 3.963503 |
| H | -0.750353 | 1.352263 | 3.319601 |
| H | 0.142315 | 0.891508 | 4.768407 |
| C | 0.831700 | -0.223099 | -4.452069 |
| H | 1.886770 | 0.023378 | -4.293170 |

| | | | |
|---|---|---|---|
| H | 0.297403 | 0.699292 | -4.704309 |
| H | 0.788492 | -0.873818 | -5.334560 |
| Ni | 1.989794 | -0.644491 | -0.227261 |
| H | 2.833897 | -0.874314 | 0.974736 |
| C | 4.055723 | -0.444726 | -0.107252 |
| N | 4.216703 | 0.933802 | 0.160660 |
| H | 3.405534 | 1.514399 | -0.048460 |
| C | 5.377763 | 1.734651 | 0.069969 |
| O | 5.228993 | 2.938319 | 0.126279 |
| C | 6.703779 | 1.061447 | -0.119158 |
| H | 6.673513 | 0.330256 | -0.935998 |
| H | 7.442788 | 1.830562 | -0.348764 |
| H | 7.019932 | 0.523053 | 0.781112 |
| C | 3.508688 | -0.868132 | -1.382019 |
| H | 3.608549 | -1.929031 | -1.608335 |
| N | 3.767098 | -0.096072 | -2.582566 |
| O | 4.058052 | -0.746150 | -3.587722 |
| O | 3.678741 | 1.136094 | -2.544930 |
| C | 5.006192 | -1.346239 | 0.611491 |
| C | 5.723057 | -2.340379 | -0.058572 |
| C | 5.242531 | -1.135665 | 1.976425 |
| C | 6.651734 | -3.117625 | 0.629287 |
| H | 5.592744 | -2.482158 | -1.130885 |
| C | 6.167536 | -1.910357 | 2.660941 |
| H | 4.702225 | -0.338693 | 2.489858 |
| C | 6.873866 | -2.907591 | 1.986518 |
| H | 7.213558 | -3.880638 | 0.095437 |
| H | 6.344408 | -1.735258 | 3.720003 |
| H | 7.604137 | -3.512641 | 2.519310 |

**VI$_{R3}$**

| | | | |
|---|---|---|---|
| P | 0.494457 | 0.064759 | -1.638799 |
| P | 0.442680 | -0.837203 | 1.367260 |
| C | -2.381236 | -0.450823 | 1.365069 |
| C | -2.136418 | -2.773182 | 0.383438 |
| C | -4.555931 | -3.475570 | 1.849000 |
| H | -3.991255 | -4.245440 | 1.328926 |
| C | -0.862996 | -3.205850 | 0.740153 |
| C | -0.465109 | 3.001786 | -0.357446 |
| C | -1.745022 | 2.589796 | -1.001172 |
| C | 0.899203 | -0.066644 | 3.008503 |
| C | -2.776199 | -3.367811 | -0.754369 |
| C | -2.072403 | 1.249368 | -1.205179 |
| C | -2.647612 | 3.609468 | -1.455477 |
| C | -3.327842 | 0.905705 | -1.763530 |
| H | -3.577243 | -0.151219 | -1.870067 |
| C | -1.080425 | 0.036967 | 0.766777 |
| H | -0.912637 | 1.065111 | 1.124368 |
| C | 0.751674 | 2.692330 | -0.953983 |
| C | 0.795208 | 1.796078 | -2.155213 |
| H | 1.766868 | 1.850513 | -2.655527 |
| H | 0.012521 | 2.051872 | -2.884527 |
| C | -4.822825 | -1.167092 | 2.594373 |
| C | -2.854751 | -1.752139 | 1.200816 |
| C | -2.157300 | -4.481693 | -1.409583 |
| C | 0.764554 | 4.268449 | 1.381636 |
| C | -1.167973 | 0.113791 | -0.778155 |
| H | -1.633776 | -0.826466 | -1.110170 |
| C | -6.022519 | -1.548163 | 3.241339 |
| H | -6.580213 | -0.787644 | 3.786687 |
| C | -4.806261 | 4.241627 | -2.455329 |
| H | -5.764496 | 3.935976 | -2.874029 |
| C | -0.113865 | -2.525369 | 1.841379 |
| H | 0.768923 | -3.104191 | 2.136919 |
| H | -0.741603 | -2.405062 | 2.737441 |
| C | -3.910125 | 3.235960 | -2.020814 |
| C | -2.780544 | -5.052595 | -2.544880 |
| H | -2.299599 | -5.901916 | -3.029286 |
| C | -1.655709 | 3.989439 | 1.604403 |
| H | -2.603169 | 3.619443 | 1.215622 |
| C | -1.624066 | 4.673225 | 2.798685 |
| H | -2.546238 | 4.829914 | 3.355139 |
| C | -0.910152 | -4.955094 | -0.938431 |
| H | -0.452726 | -5.815395 | -1.425774 |
| C | -4.071114 | -2.141775 | 1.860926 |
| C | -4.228550 | 1.864974 | -2.143485 |
| H | -5.196613 | 1.581846 | -2.555081 |
| C | -4.343367 | 0.159921 | 2.664244 |
| H | -4.927951 | 0.906419 | 3.200569 |
| C | -0.469949 | 3.759182 | 0.860687 |
| C | -4.558919 | -3.431109 | -2.409405 |
| H | -5.486167 | -3.023648 | -2.807229 |
| C | -3.152583 | 0.497575 | 2.079935 |
| H | -2.778727 | 1.519888 | 2.150374 |
| C | 0.757176 | 4.989150 | 2.600348 |
| H | 1.703183 | 5.374683 | 2.979063 |
| C | 1.970189 | 3.994375 | 0.692852 |
| H | 2.912001 | 4.388281 | 1.073714 |

| | | | |
|---|---|---|---|
| C | 1.819786 | -1.044886 | 3.741383 |
| H | 2.280339 | -0.539690 | 4.599933 |
| H | 2.639543 | -1.408364 | 3.102816 |
| H | 1.282094 | -1.918178 | 4.127858 |
| C | -3.218831 | 5.943804 | -1.838923 |
| H | -2.946386 | 6.995931 | -1.789937 |
| C | -3.993421 | -2.870046 | -1.286879 |
| H | -4.482057 | -2.029472 | -0.796435 |
| C | -2.330946 | 4.991450 | -1.396638 |
| H | -1.364005 | 5.303733 | -1.010027 |
| C | 1.965811 | 3.199252 | -0.426835 |
| H | 2.898246 | 2.992819 | -0.950249 |
| C | -3.952811 | -4.535768 | -3.042692 |
| H | -4.414859 | -4.975795 | -3.923841 |
| C | -5.719868 | -3.816524 | 2.497478 |
| H | -6.062297 | -4.849054 | 2.477353 |
| C | 1.643144 | 1.231760 | 2.716630 |
| H | 1.112478 | 1.846207 | 1.979821 |
| H | 2.656858 | 1.042873 | 2.340675 |
| H | 1.741804 | 1.834098 | 3.629180 |
| C | 0.211911 | -2.390044 | -2.780366 |
| H | -0.044133 | -3.028779 | -3.635977 |
| H | -0.539882 | -2.565748 | -2.003809 |
| H | 1.182318 | -2.724222 | -2.387651 |
| C | -0.409181 | 5.183696 | 3.300932 |
| H | -0.399385 | 5.732760 | 4.240116 |
| C | -4.473497 | 5.570784 | -2.362938 |
| H | -5.168124 | 6.335215 | -2.704422 |
| C | -1.083824 | -0.557781 | -3.869390 |
| H | -1.141961 | -1.074825 | -4.835682 |
| H | -1.187831 | 0.516489 | -4.066340 |
| H | -1.949728 | -0.881103 | -3.280439 |
| C | -6.468701 | -2.845920 | 3.193325 |
| H | -7.389726 | -3.129303 | 3.698311 |
| C | 0.241046 | -0.924133 | -3.196275 |
| C | -0.262533 | -4.303401 | 0.080034 |
| H | 0.718570 | -4.642305 | 0.413897 |
| C | -0.333243 | 0.236853 | 3.859716 |
| H | -1.014706 | -0.616828 | 3.962889 |
| H | -0.907187 | 1.078638 | 3.454533 |
| H | -0.008390 | 0.523882 | 4.868231 |
| C | 1.400165 | -0.665477 | -4.156367 |
| H | 2.362329 | -0.992261 | -3.750177 |
| H | 1.485259 | 0.387530 | -4.449735 |
| H | 1.235577 | -1.243124 | -5.074534 |
| Ni | 1.946777 | -0.643852 | -0.225840 |
| H | 3.161476 | -0.779479 | 0.878700 |
| C | 4.050533 | -0.531610 | 0.126711 |
| N | 4.342498 | 0.841437 | 0.485095 |
| H | 3.525447 | 1.449523 | 0.518091 |
| C | 5.464738 | 1.616810 | 0.151801 |
| O | 5.369282 | 2.823776 | 0.268438 |
| C | 6.715694 | 0.916159 | -0.290026 |
| H | 6.536943 | 0.245157 | -1.138717 |
| H | 7.442863 | 1.674050 | -0.584947 |
| H | 7.140214 | 0.306386 | 0.516131 |
| C | 3.511712 | -0.775343 | -1.264617 |
| H | 3.607437 | -1.813366 | -1.595352 |
| N | 4.069051 | 0.067865 | -2.313835 |
| O | 4.686367 | -0.507853 | -3.212557 |
| O | 3.912977 | 1.289362 | -2.247735 |
| C | 5.087480 | -1.521390 | 0.618452 |
| C | 5.782002 | -2.362403 | -0.251725 |
| C | 5.397525 | -1.536760 | 1.982697 |
| C | 6.765366 | -3.217547 | 0.239985 |
| H | 5.592122 | -2.323429 | -1.323787 |
| C | 6.377442 | -2.391546 | 2.471735 |
| H | 4.884011 | -0.848645 | 2.655991 |
| C | 7.061380 | -3.237479 | 1.599268 |
| H | 7.310438 | -3.859939 | -0.447940 |
| H | 6.614192 | -2.393237 | 3.533449 |
| H | 7.831656 | -3.904574 | 1.979988 |

**VII$_{R3}$**

| | | | |
|---|---|---|---|
| P | -0.320692 | 0.198553 | 1.561615 |
| P | -0.283824 | -0.682864 | -1.427220 |
| C | 2.556349 | -0.559647 | -1.412903 |
| C | 2.053402 | -2.826823 | -0.433513 |
| C | 4.415273 | -3.795503 | -1.838935 |
| H | 3.766565 | -4.492319 | -1.312459 |
| C | 0.755672 | -3.125157 | -0.829647 |
| C | 1.055376 | 3.043081 | 0.363341 |
| C | 2.280304 | 2.450188 | 0.969577 |
| C | -0.720612 | 0.224671 | -3.000897 |
| C | 2.573612 | -3.420434 | 0.762949 |
| C | 2.402094 | 1.077128 | 1.188663 |
| C | 3.333469 | 3.324485 | 1.408098 |
| C | 3.581070 | 0.554395 | 1.773102 |

| | | | | | | | |
|---|---|---|---|---|---|---|---|
| H | 3.651778 | -0.523752 | 1.919802 | H | -2.165088 | -0.000413 | -4.588671 |
| C | 1.302191 | 0.048030 | -0.820658 | H | -2.649908 | -0.821948 | -3.094879 |
| H | 1.239789 | 1.093409 | -1.161339 | H | -1.396110 | -1.525278 | -4.137971 |
| C | -0.175522 | 2.873708 | 0.990406 | C | 4.271039 | 5.545528 | 1.749824 |
| C | -0.320301 | 1.940966 | 2.147457 | H | 4.166483 | 6.626553 | 1.685255 |
| H | -1.238352 | 2.133370 | 2.707967 | C | 3.847255 | -3.078824 | 1.286122 |
| H | 0.529307 | 2.017964 | 2.841489 | H | 4.476164 | -2.380761 | 0.736992 |
| C | 4.925765 | -1.534805 | -2.612617 | C | 3.238164 | 4.738685 | 1.333749 |
| C | 2.893736 | -1.901492 | -1.245098 | H | 2.327972 | 5.194203 | 0.952813 |
| C | 1.769336 | -4.350282 | 1.500349 | C | -1.318587 | 3.576830 | 0.540650 |
| C | -0.052381 | 4.520726 | -1.290376 | H | -2.248214 | 3.493017 | 1.102265 |
| C | 1.365238 | 0.076745 | 0.726514 | C | 3.493580 | -4.516544 | 3.201919 |
| H | 1.708545 | -0.918794 | 1.032555 | H | 3.857290 | -4.926973 | 4.141270 |
| C | 6.088791 | -2.046964 | -3.236276 | C | 5.547042 | -4.263356 | -2.464020 |
| H | 6.730399 | -1.357552 | -3.784035 | H | 5.785977 | -5.324032 | -2.422090 |
| C | 5.575786 | 3.624942 | 2.381964 | C | -1.264218 | 1.599513 | -2.619840 |
| H | 6.477122 | 3.178137 | 2.800441 | H | -0.642800 | 2.090160 | -1.860849 |
| C | 0.129061 | -2.388382 | -1.971707 | H | -2.290915 | 1.545139 | -2.235322 |
| H | -0.798950 | -2.869789 | -2.299360 | H | -1.284195 | 2.259960 | -3.496686 |
| H | 0.804130 | -2.321392 | -2.836677 | C | -1.021113 | -2.118144 | 2.988424 |
| C | 4.526122 | 2.767124 | 1.974440 | H | -0.877866 | -2.771343 | 3.860084 |
| C | 2.261437 | -4.883963 | 2.716345 | H | -0.669135 | -2.676805 | 2.108753 |
| H | 1.637542 | -5.589793 | 3.264364 | H | -2.099224 | -1.912337 | 2.913748 |
| C | 2.298994 | 3.926435 | -1.615599 | C | 1.165895 | 5.328273 | -3.230528 |
| H | 3.201763 | 3.424427 | -1.270188 | H | 1.196346 | 5.902236 | -4.154222 |
| C | 2.317779 | 4.643558 | -2.790484 | C | 5.458410 | 4.988198 | 2.266345 |
| H | 3.231787 | 4.690968 | -3.379393 | H | 6.270199 | 5.638439 | 2.585358 |
| C | 0.492426 | -4.699454 | 1.001736 | C | 1.211810 | -1.157381 | 3.544519 |
| H | -0.101006 | -5.441209 | 1.536687 | H | 1.215774 | -1.657139 | 4.522502 |
| C | 4.070369 | -2.420021 | -1.880901 | H | 1.839102 | -0.262231 | 3.633067 |
| C | 4.621519 | 1.367532 | 2.136582 | H | 1.688367 | -1.846539 | 2.836515 |
| H | 5.529802 | 0.946042 | 2.565890 | C | 6.398584 | -3.383208 | -3.162934 |
| C | 4.581107 | -0.167124 | -2.700204 | H | 7.293945 | -3.766248 | -3.647966 |
| H | 5.242646 | 0.510673 | -3.238336 | C | -0.234442 | -0.821680 | 3.175609 |
| C | 1.120252 | 3.833591 | -0.831457 | C | -0.003672 | -4.086231 | -0.120859 |
| C | 4.290775 | -3.606312 | 2.477728 | H | -0.986126 | -4.360406 | -0.504250 |
| H | 5.267294 | -3.319565 | 2.862985 | C | 0.512856 | 0.405267 | -3.887378 |
| C | 3.426311 | 0.298206 | -2.128447 | H | 1.054360 | -0.529374 | -4.078854 |
| H | 3.162079 | 1.353414 | -2.208155 | H | 1.220950 | 1.123705 | -3.457575 |
| C | 0.009955 | 5.271547 | -2.488426 | H | 0.196874 | 0.807018 | -4.858977 |
| H | -0.886889 | 5.795940 | -2.817075 | C | -0.853414 | -0.023494 | 4.328307 |
| C | -1.256187 | 4.400900 | -0.554987 | H | -1.878536 | 0.307801 | 4.138376 |
| H | -2.146223 | 4.944656 | -0.871203 | H | -0.252728 | 0.852632 | 4.593593 |
| C | -1.790554 | -0.583547 | -3.737710 | H | -0.887613 | -0.669791 | 5.214657 |

| | | | |
|---|---|---|---|
| Ni | -1.784350 | -0.556692 | 0.172496 |
| H | -3.246360 | 0.102172 | -0.963402 |
| C | -3.992070 | 0.367432 | -0.163893 |
| N | -4.012024 | 1.812207 | -0.260102 |
| H | -3.107959 | 2.239427 | -0.444189 |
| C | -4.860592 | 2.722497 | 0.357364 |
| O | -4.548271 | 3.902292 | 0.361929 |
| C | -6.152256 | 2.212283 | 0.931025 |
| H | -6.012686 | 1.340874 | 1.580513 |
| H | -6.611155 | 3.016196 | 1.508607 |
| H | -6.842744 | 1.911223 | 0.133863 |
| C | -3.445285 | -0.279168 | 1.124433 |
| H | -3.894085 | -1.255768 | 1.298551 |
| N | -3.627448 | 0.420800 | 2.381623 |
| O | -4.023601 | -0.258766 | 3.338008 |
| O | -3.356312 | 1.623512 | 2.463774 |
| C | -5.271381 | -0.283994 | -0.668395 |
| C | -6.127734 | -1.027553 | 0.146941 |
| C | -5.591826 | -0.129723 | -2.021275 |
| C | -7.283985 | -1.601313 | -0.381264 |
| H | -5.921409 | -1.133855 | 1.212567 |
| C | -6.738667 | -0.707777 | -2.551501 |
| H | -4.935843 | 0.468172 | -2.656163 |
| C | -7.588226 | -1.450035 | -1.731075 |
| H | -7.951852 | -2.160169 | 0.271330 |
| H | -6.974053 | -0.576362 | -3.605738 |
| H | -8.490815 | -1.897762 | -2.141687 |
| C | -3.366127 | -3.143371 | -0.392628 |
| C | -4.314013 | -3.911658 | -1.235792 |
| H | -4.268354 | -4.981400 | -1.018672 |
| H | -5.332482 | -3.568229 | -1.009737 |
| H | -4.120582 | -3.714431 | -2.291961 |
| O | -3.214825 | -3.621776 | 0.834637 |
| H | -2.570289 | -3.053889 | 1.312478 |
| O | -2.773766 | -2.136996 | -0.795505 |

**TSIII$_{R3}$**

| | | | |
|---|---|---|---|
| P | 0.296989 | 0.161399 | -1.639027 |
| P | 0.342294 | -0.738514 | 1.334434 |
| C | -2.473908 | -0.453479 | 1.464342 |
| C | -2.162534 | -2.750273 | 0.477653 |
| C | -4.537036 | -3.549323 | 1.981016 |
| H | -3.967403 | -4.290801 | 1.425544 |
| C | -0.865575 | -3.111102 | 0.825965 |
| C | -0.936998 | 3.067107 | -0.400437 |
| C | -2.208942 | 2.505527 | -0.936491 |
| C | 0.971552 | 0.201476 | 2.823906 |
| C | -2.761933 | -3.336719 | -0.685276 |
| C | -2.382535 | 1.136629 | -1.148463 |
| C | -3.260087 | 3.410362 | -1.315440 |
| C | -3.608963 | 0.649114 | -1.660093 |
| H | -3.718512 | -0.425853 | -1.803334 |
| C | -1.222208 | 0.080988 | 0.799743 |
| H | -1.090349 | 1.123221 | 1.127410 |
| C | 0.254931 | 2.858034 | -1.087805 |
| C | 0.305400 | 1.905749 | -2.235275 |
| H | 1.172935 | 2.073366 | -2.873317 |
| H | -0.596687 | 1.982690 | -2.859853 |
| C | -4.843245 | -1.260707 | 2.778725 |
| C | -2.912417 | -1.768502 | 1.310694 |
| C | -2.034497 | -4.317590 | -1.435764 |
| C | 0.290471 | 4.535397 | 1.175778 |
| C | -1.355057 | 0.102142 | -0.739488 |
| H | -1.745357 | -0.883206 | -1.021409 |
| C | -6.008125 | -1.688402 | 3.459203 |
| H | -6.569137 | -0.955450 | 4.037869 |
| C | -5.548847 | 3.775521 | -2.149630 |
| H | -6.486603 | 3.354474 | -2.510397 |
| C | -0.153202 | -2.397762 | 1.933792 |
| H | 0.750563 | -2.928249 | 2.248079 |
| H | -0.797256 | -2.256777 | 2.812337 |
| C | -4.501208 | 2.888112 | -1.805013 |
| C | -2.600941 | -4.851125 | -2.618524 |
| H | -2.033575 | -5.596397 | -3.175282 |
| C | -2.050428 | 3.991610 | 1.634986 |
| H | -2.982451 | 3.510768 | 1.342234 |
| C | -1.986220 | 4.705794 | 2.810026 |
| H | -2.863892 | 4.771562 | 3.450063 |
| C | -0.753924 | -4.717270 | -0.989151 |
| H | -0.217967 | -5.488551 | -1.541221 |
| C | -4.091170 | -2.202431 | 2.003944 |
| C | -4.646308 | 1.491841 | -1.958833 |
| H | -5.590088 | 1.096491 | -2.332499 |
| C | -4.394769 | 0.076752 | 2.856418 |
| H | -4.976312 | 0.796848 | 3.430690 |
| C | -0.919999 | 3.871231 | 0.787207 |
| C | -4.554985 | -3.471735 | -2.326265 |
| H | -5.531873 | -3.145584 | -2.677789 |

| | | | |
|---|---|---|---|
| C | -3.238592 | 0.459414 | 2.230029 |
| H | -2.894529 | 1.491825 | 2.300926 |
| C | 0.311872 | 5.288709 | 2.374257 |
| H | 1.235862 | 5.795897 | 2.650793 |
| C | 1.447237 | 4.386285 | 0.372828 |
| H | 2.358762 | 4.924306 | 0.633682 |
| C | 2.129965 | -0.571261 | 3.455226 |
| H | 2.518855 | 0.003735 | 4.305318 |
| H | 2.960835 | -0.731143 | 2.757547 |
| H | 1.820014 | -1.551110 | 3.837474 |
| C | -4.151006 | 5.657746 | -1.609268 |
| H | -4.010184 | 6.735392 | -1.557261 |
| C | -4.038431 | -2.944020 | -1.164424 |
| H | -4.612168 | -2.208480 | -0.603930 |
| C | -3.118680 | 4.821480 | -1.254459 |
| H | -2.173739 | 5.251849 | -0.934085 |
| C | 1.434628 | 3.548229 | -0.714641 |
| H | 2.327105 | 3.447986 | -1.333234 |
| C | -3.833007 | -4.433818 | -3.062437 |
| H | -4.253867 | -4.843943 | -3.977831 |
| C | -5.668621 | -3.934952 | 2.660697 |
| H | -5.984806 | -4.975512 | 2.631495 |
| C | 1.436831 | 1.582658 | 2.373166 |
| H | 0.681174 | 2.101833 | 1.773934 |
| H | 2.361544 | 1.542247 | 1.787086 |
| H | 1.637678 | 2.211112 | 3.250513 |
| C | 0.827109 | -2.188810 | -3.064123 |
| H | 0.573042 | -2.860195 | -3.895944 |
| H | 0.547750 | -2.705888 | -2.136079 |
| H | 1.915315 | -2.059135 | -3.083533 |
| C | -0.797143 | 5.365727 | 3.183047 |
| H | -0.762723 | 5.938645 | 4.107224 |
| C | -5.384466 | 5.134918 | -2.047790 |
| H | -6.194928 | 5.808169 | -2.318838 |
| C | -1.382531 | -1.122601 | -3.537487 |
| H | -1.450249 | -1.619840 | -4.514233 |
| H | -1.973280 | -0.200294 | -3.597551 |
| H | -1.857036 | -1.793383 | -2.810491 |
| C | -6.418530 | -2.997811 | 3.400733 |
| H | -7.313443 | -3.316074 | 3.931023 |
| C | 0.094290 | -0.860522 | -3.237611 |
| C | -0.180474 | -4.112847 | 0.099678 |
| H | 0.810650 | -4.414915 | 0.435463 |
| C | -0.164989 | 0.376012 | 3.837094 |
| H | -0.628484 | -0.569191 | 4.142738 |
| H | -0.953167 | 1.038502 | 3.461506 |
| H | 0.246390 | 0.843884 | 4.740941 |
| C | 0.704013 | -0.098906 | -4.419061 |
| H | 1.746602 | 0.194857 | -4.260429 |
| H | 0.128633 | 0.796438 | -4.678080 |
| H | 0.686106 | -0.758542 | -5.295902 |
| Ni | 1.778380 | -0.824428 | -0.272448 |
| H | 3.216028 | 0.122062 | 0.713977 |
| C | 4.087535 | 0.238594 | 0.036418 |
| N | 4.180390 | 1.676597 | -0.083665 |
| H | 3.295052 | 2.170722 | -0.029882 |
| C | 5.118741 | 2.419633 | -0.784373 |
| O | 4.896552 | 3.598974 | -1.004575 |
| C | 6.389512 | 1.720955 | -1.184528 |
| H | 6.212389 | 0.738339 | -1.639636 |
| H | 6.928291 | 2.352094 | -1.892808 |
| H | 7.026466 | 1.549987 | -0.308253 |
| C | 3.808434 | -0.570715 | -1.266865 |
| H | 4.693280 | -1.167696 | -1.504717 |
| N | 3.614484 | 0.149994 | -2.537829 |
| O | 3.944314 | -0.465677 | -3.551365 |
| O | 3.101124 | 1.270864 | -2.547388 |
| C | 5.230810 | -0.341103 | 0.866418 |
| C | 5.858171 | -1.563880 | 0.618926 |
| C | 5.617980 | 0.385022 | 2.000972 |
| C | 6.860424 | -2.035102 | 1.467692 |
| H | 5.589747 | -2.177906 | -0.239498 |
| C | 6.613020 | -0.084821 | 2.847677 |
| H | 5.138099 | 1.342194 | 2.201613 |
| C | 7.243490 | -1.298910 | 2.582237 |
| H | 7.347896 | -2.982402 | 1.243368 |
| H | 6.902021 | 0.503203 | 3.716325 |
| H | 8.028815 | -1.666293 | 3.239179 |
| C | 3.083525 | -3.208206 | 0.122158 |
| C | 3.513037 | -4.443384 | 0.839883 |
| H | 3.401791 | -5.322463 | 0.200671 |
| H | 4.577647 | -4.347000 | 1.086984 |
| H | 2.966945 | -4.572413 | 1.777464 |
| O | 3.268100 | -3.120482 | -1.130557 |
| H | 3.214701 | -1.838053 | -1.304657 |
| O | 2.587460 | -2.247610 | 0.801049 |

**VIII$_{R3}$**

| | | | |
|---|---|---|---|
| P | 0.022444 | -0.021154 | -1.754849 |
| P | 0.152620 | -0.982794 | 1.143209 |
| C | -2.554289 | -0.236985 | 1.534993 |
| C | -2.714975 | -2.558025 | 0.552438 |
| C | -4.955282 | -3.004600 | 2.358905 |
| H | -4.559375 | -3.826025 | 1.766079 |
| C | -1.470961 | -3.151512 | 0.733354 |
| C | -0.553205 | 3.012098 | -0.435402 |
| C | -1.916897 | 2.703419 | -0.955701 |
| C | 1.010480 | -0.220228 | 2.609023 |
| C | -3.565956 | -3.025878 | -0.500565 |
| C | -2.380760 | 1.398193 | -1.118486 |
| C | -2.760062 | 3.798477 | -1.350786 |
| C | -3.703153 | 1.168075 | -1.570006 |
| H | -4.051357 | 0.137425 | -1.651491 |
| C | -1.302686 | 0.087878 | 0.753221 |
| H | -0.960162 | 1.083931 | 1.072445 |
| C | 0.573857 | 2.605557 | -1.142873 |
| C | 0.437688 | 1.678917 | -2.307751 |
| H | 1.352975 | 1.630029 | -2.901294 |
| H | -0.387191 | 1.984512 | -2.968032 |
| C | -4.853239 | -0.685000 | 3.117705 |
| C | -3.187604 | -1.475652 | 1.461465 |
| C | -3.174647 | -4.166862 | -1.272699 |
| C | 0.934284 | 4.215234 | 1.140572 |
| C | -1.552573 | 0.181247 | -0.772028 |
| H | -2.132500 | -0.710018 | -1.056283 |
| C | -5.982154 | -0.936703 | 3.933621 |
| H | -6.369079 | -0.124301 | 4.547940 |
| C | -4.923333 | 4.617615 | -2.195169 |
| H | -5.934741 | 4.396400 | -2.534644 |
| C | -0.507075 | -2.587578 | 1.732027 |
| H | 0.346706 | -3.256690 | 1.888765 |
| H | -0.982269 | -2.410938 | 2.708999 |
| C | -4.091547 | 3.538550 | -1.811387 |
| C | -4.009100 | -4.606758 | -2.328679 |
| H | -3.701572 | -5.478111 | -2.906044 |
| C | -1.467129 | 4.131421 | 1.604604 |
| H | -2.474255 | 3.831696 | 1.319012 |
| C | -1.265752 | 4.837903 | 2.768421 |
| H | -2.115005 | 5.084096 | 3.402916 |
| C | -1.940503 | -4.798252 | -0.986267 |
| H | -1.652761 | -5.678156 | -1.560655 |
| C | -4.329823 | -1.732582 | 2.292388 |
| C | -4.542010 | 2.201941 | -1.889491 |
| H | -5.560276 | 2.005332 | -2.223112 |
| C | -4.220224 | 0.578439 | 3.105838 |
| H | -4.630406 | 1.380758 | 3.718058 |
| C | -0.381003 | 3.788931 | 0.758091 |
| C | -5.556259 | -2.811978 | -1.882205 |
| H | -6.476823 | -2.286657 | -2.129195 |
| C | -3.096118 | 0.785474 | 2.351487 |
| H | -2.601618 | 1.757815 | 2.361627 |
| C | 1.102829 | 4.952657 | 2.337062 |
| H | 2.108507 | 5.270787 | 2.609799 |
| C | 2.040086 | 3.870864 | 0.326051 |
| H | 3.042872 | 4.207807 | 0.590783 |
| C | 1.944748 | -1.265739 | 3.220168 |
| H | 2.546927 | -0.791046 | 4.006251 |
| H | 2.637418 | -1.695801 | 2.486342 |
| H | 1.391496 | -2.087262 | 3.690680 |
| C | -3.145127 | 6.171928 | -1.732701 |
| H | -2.775005 | 7.195078 | -1.722266 |
| C | -4.779119 | -2.371546 | -0.836106 |
| H | -5.089815 | -1.502883 | -0.258220 |
| C | -2.316561 | 5.146702 | -1.340855 |
| H | -1.298674 | 5.372125 | -1.032691 |
| C | 1.859206 | 3.068569 | -0.772057 |
| H | 2.707128 | 2.808660 | -1.405064 |
| C | -5.173252 | -3.942289 | -2.633539 |
| H | -5.799693 | -4.284615 | -3.454427 |
| C | -6.042324 | -3.220319 | 3.172864 |
| H | -6.497279 | -4.208017 | 3.211673 |
| C | 1.799056 | 0.968776 | 2.076805 |
| H | 1.153245 | 1.668327 | 1.534109 |
| H | 2.591494 | 0.644946 | 1.389894 |
| H | 2.271056 | 1.527662 | 2.896636 |
| C | -0.624942 | -2.395091 | -2.968261 |
| H | -0.996811 | -2.953679 | -3.838014 |
| H | -1.359509 | -2.518099 | -2.164153 |
| H | 0.315298 | -2.858085 | -2.644700 |
| C | 0.028848 | 5.255215 | 3.140063 |
| H | 0.173264 | 5.818899 | 4.059339 |
| C | -4.464653 | 5.911352 | -2.154177 |
| H | -5.110269 | 6.733312 | -2.456014 |
| C | -1.749401 | -0.388029 | -3.905944 |
| H | -1.918748 | -0.861145 | -4.882194 |
| H | -1.746931 | 0.696886 | -4.066904 |

| | | | |
|---|---|---|---|
| H | -2.611713 | -0.635284 | -3.274342 |
| C | -6.570102 | -2.177439 | 3.961253 |
| H | -7.434511 | -2.361686 | 4.595479 |
| C | -0.440787 | -0.924083 | -3.324071 |
| C | -1.096142 | -4.278676 | -0.037242 |
| H | -0.128495 | -4.744456 | 0.153400 |
| C | -0.002268 | 0.258885 | 3.650109 |
| H | -0.717470 | -0.518594 | 3.947045 |
| H | -0.571806 | 1.128106 | 3.299359 |
| H | 0.539532 | 0.571075 | 4.552411 |
| C | 0.703499 | -0.745307 | -4.324011 |
| H | 1.672081 | -1.044088 | -3.908866 |
| H | 0.785948 | 0.289291 | -4.676741 |
| H | 0.510948 | -1.373135 | -5.203175 |
| Ni | 1.364843 | -1.274428 | -0.585465 |
| H | 3.754598 | -0.530154 | -0.108763 |
| C | 4.798919 | -0.175041 | -0.186333 |
| N | 4.699283 | 1.249709 | 0.098636 |
| H | 3.786267 | 1.661332 | -0.061890 |
| C | 5.681442 | 2.198134 | 0.296633 |
| O | 5.368166 | 3.377637 | 0.382053 |
| C | 7.104162 | 1.726379 | 0.440625 |
| H | 7.379044 | 0.937157 | -0.268676 |
| H | 7.767118 | 2.582526 | 0.304585 |
| H | 7.269200 | 1.314652 | 1.443614 |
| C | 5.224622 | -0.456064 | -1.619785 |
| H | 6.181150 | 0.001486 | -1.893208 |
| N | 4.235690 | 0.065983 | -2.630499 |
| O | 4.523838 | -0.089448 | -3.803578 |
| O | 3.202081 | 0.594781 | -2.215474 |
| C | 5.605506 | -0.938062 | 0.847324 |
| C | 6.567047 | -1.895777 | 0.519082 |
| C | 5.350263 | -0.681314 | 2.199143 |
| C | 7.252403 | -2.585156 | 1.519233 |
| H | 6.814556 | -2.106217 | -0.520766 |
| C | 6.027457 | -1.371107 | 3.196406 |
| H | 4.624543 | 0.089622 | 2.459455 |
| C | 6.983334 | -2.328263 | 2.858768 |
| H | 8.008081 | -3.318099 | 1.244070 |
| H | 5.815000 | -1.155208 | 4.242036 |
| H | 7.521439 | -2.863368 | 3.637939 |
| C | 2.899473 | -2.934750 | -0.970433 |
| C | 3.808268 | -4.086514 | -1.197821 |
| H | 4.284425 | -4.026007 | -2.179176 |
| H | 4.562099 | -4.132843 | -0.405089 |
| H | 3.235375 | -5.019563 | -1.145014 |
| O | 2.587541 | -2.115213 | -1.896626 |
| H | 5.288659 | -1.524296 | -1.848154 |
| O | 2.390176 | -2.716740 | 0.185840 |

**IV$_{R1}$**

| | | | |
|---|---|---|---|
| P | 0.236440 | -0.350572 | -1.739800 |
| P | 0.347245 | -0.804913 | 1.317377 |
| C | -2.275398 | 0.324076 | 1.560240 |
| C | -2.738527 | -2.100590 | 1.055337 |
| C | -4.969368 | -1.916542 | 2.913999 |
| H | -4.678821 | -2.880754 | 2.503090 |
| C | -1.570024 | -2.790642 | 1.355570 |
| C | -0.180396 | 2.935493 | -0.928641 |
| C | -1.539939 | 2.599778 | -1.440437 |
| C | 1.250040 | 0.150455 | 2.650758 |
| C | -3.660648 | -2.654223 | 0.109564 |
| C | -2.055403 | 1.306730 | -1.377888 |
| C | -2.305173 | 3.633104 | -2.081383 |
| C | -3.351418 | 1.039315 | -1.882887 |
| H | -3.736760 | 0.021104 | -1.807439 |
| C | -1.012338 | 0.338964 | 0.728262 |
| H | -0.549611 | 1.329142 | 0.847430 |
| C | 0.942712 | 2.347944 | -1.503462 |
| C | 0.801867 | 1.236606 | -2.497969 |
| H | 1.750897 | 1.050592 | -3.013811 |
| H | 0.052849 | 1.507030 | -3.253737 |
| C | -4.588660 | 0.479520 | 3.189359 |
| C | -3.056672 | -0.815227 | 1.732857 |
| C | -3.413883 | -3.951061 | -0.443946 |
| C | 1.313799 | 4.326529 | 0.479990 |
| C | -1.292491 | 0.148738 | -0.783233 |
| H | -1.930839 | -0.740153 | -0.864912 |
| C | -5.725185 | 0.530160 | 4.031199 |
| H | -6.006733 | 1.489325 | 4.465037 |
| C | -4.375220 | 4.356988 | -3.201137 |
| H | -5.373228 | 4.110935 | -3.562978 |
| C | -0.516017 | -2.160850 | 2.216479 |
| H | 0.247768 | -2.891350 | 2.507597 |
| H | -0.945247 | -1.731920 | 3.134835 |
| C | -3.616544 | 3.337814 | -2.577065 |
| C | -4.313758 | -4.480707 | -1.399971 |
| H | -4.114816 | -5.471566 | -1.807884 |

| | | | | | | | |
|---|---|---|---|---|---|---|---|
| C | -1.102762 | 4.465834 | 0.818252 | C | 0.394844 | 5.743846 | 2.224877 |
| H | -2.113055 | 4.179574 | 0.531837 | H | 0.536388 | 6.447314 | 3.042713 |
| C | -0.907414 | 5.351108 | 1.853365 | C | -3.864572 | 5.622191 | -3.358995 |
| H | -1.765971 | 5.756476 | 2.385572 | H | -4.454863 | 6.396707 | -3.844120 |
| C | -2.264004 | -4.662455 | -0.029166 | C | -1.055691 | -0.511594 | -4.287227 |
| H | -2.097396 | -5.666847 | -0.419357 | H | -1.485320 | -1.185967 | -5.040186 |
| C | -4.204097 | -0.765163 | 2.594728 | H | -0.360358 | 0.151654 | -4.813996 |
| C | -4.117617 | 2.023047 | -2.450563 | H | -1.877167 | 0.096039 | -3.887104 |
| H | -5.116852 | 1.800297 | -2.823715 | C | -6.451571 | -0.603025 | 4.305364 |
| C | -3.811512 | 1.629662 | 2.925584 | H | -7.321448 | -0.553902 | 4.956894 |
| H | -4.114118 | 2.583247 | 3.357546 | C | -0.361390 | -1.349677 | -3.213350 |
| C | -0.006360 | 3.908143 | 0.110878 | C | -1.362198 | -4.086941 | 0.829015 |
| C | -5.649047 | -2.478055 | -1.282350 | H | -0.471963 | -4.630832 | 1.139920 |
| H | -6.514630 | -1.908438 | -1.615212 | C | 0.299830 | 0.959359 | 3.533524 |
| C | -2.683838 | 1.544124 | 2.153573 | H | -0.497403 | 0.352220 | 3.981347 |
| H | -2.080517 | 2.433464 | 1.973997 | H | -0.170024 | 1.783349 | 2.983895 |
| C | 1.479238 | 5.246056 | 1.543247 | H | 0.870254 | 1.408624 | 4.357038 |
| H | 2.490529 | 5.551749 | 1.811288 | C | 0.894082 | -2.000206 | -3.806817 |
| C | 2.418042 | 3.815669 | -0.242492 | H | 1.347244 | -2.711432 | -3.108866 |
| H | 3.415617 | 4.198651 | -0.022180 | H | 1.657582 | -1.254866 | -4.071931 |
| C | 2.035312 | -0.831774 | 3.518725 | H | 0.628949 | -2.538137 | -4.727073 |
| H | 2.675034 | -0.271139 | 4.213576 | Ni | 1.510779 | -1.408765 | -0.296654 |
| H | 2.682505 | -1.484643 | 2.918035 | H | 2.162428 | -1.822938 | 0.992109 |
| H | 1.374933 | -1.467277 | 4.121433 | C | 5.080803 | -0.327763 | 0.107880 |
| C | -2.561617 | 5.910394 | -2.905039 | N | 4.664864 | 0.434462 | -0.959769 |
| H | -2.147103 | 6.905723 | -3.052095 | H | 4.242802 | 1.333622 | -0.738575 |
| C | -4.803231 | -1.942419 | -0.338243 | C | 4.673211 | 0.145775 | -2.337445 |
| H | -5.003202 | -0.952472 | 0.067584 | O | 4.051653 | 0.876255 | -3.086637 |
| C | -1.803531 | 4.945016 | -2.284963 | C | 5.542181 | -0.967196 | -2.840377 |
| H | -0.796080 | 5.188694 | -1.957217 | H | 4.914307 | -1.807822 | -3.155628 |
| C | 2.232650 | 2.845223 | -1.195074 | H | 6.056025 | -0.600533 | -3.732707 |
| H | 3.062239 | 2.477776 | -1.797469 | H | 6.278355 | -1.329958 | -2.118771 |
| C | -5.408083 | -3.760690 | -1.816240 | C | 4.957041 | -1.697193 | 0.124270 |
| H | -6.088463 | -4.176291 | -2.556437 | H | 5.533659 | -2.325418 | 0.790971 |
| C | -6.059491 | -1.837358 | 3.748576 | N | 4.132058 | -2.431356 | -0.730793 |
| H | -6.623763 | -2.737698 | 3.983371 | O | 4.352483 | -3.641456 | -0.874539 |
| C | 2.194833 | 1.102757 | 1.926266 | O | 3.155445 | -1.863258 | -1.339094 |
| H | 1.643271 | 1.781792 | 1.265872 | C | 5.641762 | 0.397400 | 1.244796 |
| H | 2.909758 | 0.544387 | 1.307419 | C | 5.605888 | -0.155833 | 2.536484 |
| H | 2.763122 | 1.716506 | 2.641064 | C | 6.160339 | 1.692975 | 1.076499 |
| C | -1.325089 | -2.424489 | -2.708073 | C | 6.078094 | 0.564099 | 3.623304 |
| H | -1.479106 | -3.176354 | -3.494597 | H | 5.164282 | -1.139573 | 2.687129 |
| H | -2.315404 | -2.012599 | -2.470924 | C | 6.623861 | 2.413423 | 2.168574 |
| H | -0.949146 | -2.948838 | -1.821103 | H | 6.226034 | 2.121863 | 0.077751 |

| | | | |
|---|---|---|---|
| C | 6.583609 | 1.852392 | 3.443845 |
| H | 6.036390 | 0.127637 | 4.618991 |
| H | 7.029978 | 3.411976 | 2.023828 |
| H | 6.944779 | 2.418870 | 4.299463 |
| C | 1.693738 | -4.545653 | -0.468430 |
| C | 1.943420 | -5.746154 | -1.323121 |
| H | 2.994301 | -5.743918 | -1.635493 |
| H | 1.744173 | -6.684839 | -0.794473 |
| H | 1.321586 | -5.695183 | -2.218639 |
| O | 2.086852 | -4.607075 | 0.819496 |
| H | 2.567277 | -5.435871 | 0.980027 |
| O | 1.145121 | -3.537492 | -0.874095 |

**$V_{R1}$**

| | | | |
|---|---|---|---|
| P | 0.425070 | -0.347192 | -1.697903 |
| P | 0.444413 | -0.792728 | 1.372945 |
| C | -2.273056 | 0.101545 | 1.472363 |
| C | -2.493389 | -2.342801 | 0.883240 |
| C | -4.934880 | -2.347361 | 2.493991 |
| H | -4.551561 | -3.279306 | 2.086480 |
| C | -1.278044 | -2.894431 | 1.272806 |
| C | 0.077683 | 2.875069 | -0.814085 |
| C | -1.301223 | 2.618503 | -1.322840 |
| C | 1.278620 | 0.182874 | 2.734148 |
| C | -3.265355 | -3.007558 | -0.125892 |
| C | -1.867780 | 1.344222 | -1.335855 |
| C | -2.044146 | 3.724363 | -1.862193 |
| C | -3.198823 | 1.170836 | -1.787019 |
| H | -3.634023 | 0.170961 | -1.743354 |
| C | -0.963010 | 0.248944 | 0.729476 |
| H | -0.593384 | 1.269601 | 0.907668 |
| C | 1.173284 | 2.289891 | -1.439921 |
| C | 0.969252 | 1.229758 | -2.475103 |
| H | 1.890053 | 1.041419 | -3.032937 |
| H | 0.189343 | 1.527518 | -3.189979 |
| C | -4.725229 | 0.052504 | 2.882883 |
| C | -2.989300 | -1.095875 | 1.532696 |
| C | -2.824797 | -4.276562 | -0.627555 |
| C | 1.638667 | 4.148648 | 0.630453 |
| C | -1.150313 | 0.123928 | -0.803671 |
| H | -1.801747 | -0.746050 | -0.975141 |
| C | -5.937731 | 0.008258 | 3.611228 |
| H | -6.312712 | 0.932994 | 4.048684 |
| C | -4.121819 | 4.610073 | -2.842307 |
| H | -5.142819 | 4.431593 | -3.178227 |
| C | -0.391364 | -2.172003 | 2.236341 |
| H | 0.376868 | -2.835027 | 2.648158 |
| H | -0.960764 | -1.743407 | 3.074082 |
| C | -3.385331 | 3.519249 | -2.320440 |
| C | -3.573803 | -4.919839 | -1.641250 |
| H | -3.226569 | -5.885406 | -2.008019 |
| C | -0.764380 | 4.293962 | 1.061467 |
| H | -1.787742 | 4.040786 | 0.790275 |
| C | -0.521026 | 5.105482 | 2.146325 |
| H | -1.354282 | 5.479257 | 2.738229 |
| C | -1.609739 | -4.824041 | -0.154552 |
| H | -1.276397 | -5.785216 | -0.544125 |
| C | -4.212328 | -1.143882 | 2.284362 |
| C | -3.942807 | 2.222873 | -2.251480 |
| H | -4.969554 | 2.070708 | -2.582375 |
| C | -3.995152 | 1.253952 | 2.747673 |
| H | -4.390513 | 2.166409 | 3.192710 |
| C | 0.299978 | 3.776137 | 0.279205 |
| C | -5.118403 | -3.071580 | -1.702502 |
| H | -6.001681 | -2.603470 | -2.132954 |
| C | -2.799756 | 1.267399 | 2.080472 |
| H | -2.232159 | 2.194710 | 1.997557 |
| C | 1.852242 | 5.000506 | 1.740758 |
| H | 2.876135 | 5.274549 | 1.994591 |
| C | 2.717170 | 3.628581 | -0.124581 |
| H | 3.731135 | 3.953348 | 0.111172 |
| C | 1.970951 | -0.793085 | 3.684367 |
| H | 2.593828 | -0.228495 | 4.390740 |
| H | 2.628921 | -1.490378 | 3.148863 |
| H | 1.254987 | -1.377863 | 4.273755 |
| C | -2.232240 | 6.063115 | -2.514037 |
| H | -1.780961 | 7.048757 | -2.608582 |
| C | -4.429584 | -2.432284 | -0.696478 |
| H | -4.776010 | -1.467086 | -0.330189 |
| C | -1.494040 | 5.026316 | -1.993474 |
| H | -0.465643 | 5.206894 | -1.690455 |
| C | 2.489819 | 2.699497 | -1.109460 |
| H | 3.312259 | 2.303100 | -1.706817 |
| C | -4.694234 | -4.330659 | -2.175491 |
| H | -5.251616 | -4.828630 | -2.965871 |
| C | -6.104596 | -2.359871 | 3.217309 |
| H | -6.633162 | -3.299395 | 3.366123 |
| C | 2.302894 | 1.079357 | 2.052923 |

| | | | |
|---|---|---|---|
| H | 1.825931 | 1.707754 | 1.294421 |
| H | 3.078856 | 0.478004 | 1.560716 |
| H | 2.796527 | 1.742676 | 2.777737 |
| C | -0.523878 | -2.755268 | -2.581759 |
| H | -0.834101 | -3.426207 | -3.394734 |
| H | -1.380022 | -2.655237 | -1.905827 |
| H | 0.288514 | -3.242259 | -2.029356 |
| C | 0.798587 | 5.462713 | 2.492581 |
| H | 0.978542 | 6.107967 | 3.349785 |
| C | -3.562256 | 5.861006 | -2.933982 |
| H | -4.136193 | 6.691542 | -3.339552 |
| C | -1.277791 | -0.796135 | -3.916184 |
| H | -1.501668 | -1.429242 | -4.785165 |
| H | -1.076945 | 0.213235 | -4.295558 |
| H | -2.188097 | -0.754239 | -3.306370 |
| C | -6.621034 | -1.171772 | 3.773456 |
| H | -7.550060 | -1.195357 | 4.339145 |
| C | -0.107322 | -1.401958 | -3.145833 |
| C | -0.837335 | -4.127888 | 0.738039 |
| H | 0.120364 | -4.530019 | 1.070077 |
| C | 0.280165 | 1.041684 | 3.510698 |
| H | -0.562518 | 0.464713 | 3.912433 |
| H | -0.126356 | 1.857651 | 2.899889 |
| H | 0.795235 | 1.508517 | 4.360680 |
| C | 1.109148 | -1.562207 | -4.058271 |
| H | 1.956825 | -2.003256 | -3.519116 |
| H | 1.434887 | -0.613556 | -4.501380 |
| H | 0.859484 | -2.241746 | -4.883371 |
| Ni | 1.671335 | -1.380877 | -0.209790 |
| H | 2.203339 | -1.947386 | 1.078011 |
| C | 4.705940 | -1.270435 | -0.031443 |
| N | 4.176051 | -0.451629 | -1.018082 |
| H | 3.922869 | 0.494288 | -0.731132 |
| C | 4.337469 | -0.537436 | -2.424050 |
| O | 3.969661 | 0.413466 | -3.087254 |
| C | 4.988579 | -1.742424 | -3.025712 |
| H | 4.246417 | -2.523127 | -3.229654 |
| H | 5.410793 | -1.437101 | -3.984814 |
| H | 5.770514 | -2.181520 | -2.400005 |
| C | 4.620144 | -2.638302 | -0.104687 |
| H | 5.279013 | -3.301059 | 0.442094 |
| N | 3.678056 | -3.323938 | -0.880859 |
| O | 3.879342 | -4.495686 | -1.183422 |
| O | 2.590083 | -2.732939 | -1.236894 |
| C | 5.406961 | -0.608931 | 1.063959 |
| C | 5.482816 | -1.219719 | 2.327310 |
| C | 5.958315 | 0.672957 | 0.891365 |
| C | 6.095812 | -0.566523 | 3.386088 |
| H | 5.020027 | -2.194119 | 2.478201 |
| C | 6.566544 | 1.323896 | 1.955048 |
| H | 5.937101 | 1.147356 | -0.089312 |
| C | 6.635362 | 0.707313 | 3.203561 |
| H | 6.142472 | -1.044667 | 4.361933 |
| H | 7.000294 | 2.310283 | 1.808418 |
| H | 7.112015 | 1.219339 | 4.036485 |

**$TSII_{R1}$**

| | | | |
|---|---|---|---|
| P | 0.488415 | -0.163651 | -1.731399 |
| P | 0.555070 | -0.853297 | 1.244440 |
| C | -2.173283 | -0.039551 | 1.487925 |
| C | -2.374942 | -2.432952 | 0.712352 |
| C | -4.625066 | -2.673759 | 2.554845 |
| H | -4.224120 | -3.558720 | 2.066180 |
| C | -1.132726 | -3.020999 | 0.923642 |
| C | -0.006016 | 2.981744 | -0.660231 |
| C | -1.359044 | 2.685554 | -1.216899 |
| C | 1.265419 | -0.023980 | 2.769400 |
| C | -3.246384 | -2.978355 | -0.285906 |
| C | -1.867096 | 1.388459 | -1.285312 |
| C | -2.134904 | 3.770625 | -1.751537 |
| C | -3.170540 | 1.164242 | -1.791889 |
| H | -3.553938 | 0.141781 | -1.802012 |
| C | -0.891414 | 0.196714 | 0.720730 |
| H | -0.540118 | 1.206833 | 0.980966 |
| C | 1.129955 | 2.465019 | -1.274227 |
| C | 0.996363 | 1.475713 | -2.387017 |
| H | 1.939516 | 1.362702 | -2.932577 |
| H | 0.226008 | 1.793349 | -3.102576 |
| C | -4.516849 | -0.285940 | 3.052200 |
| C | -2.827285 | -1.269413 | 1.525159 |
| C | -2.874495 | -4.184971 | -0.963594 |
| C | 1.463276 | 4.232394 | 0.894863 |
| C | -1.098355 | 0.177083 | -0.812707 |
| H | -1.709187 | -0.709768 | -1.035820 |
| C | -5.664722 | -0.436115 | 3.866180 |
| H | -6.057168 | 0.443001 | 4.376525 |
| C | -4.209131 | 4.583720 | -2.799661 |
| H | -5.205752 | 4.367616 | -3.183213 |

| | | | |
|---|---|---|---|
| C | -0.173493 | -2.411685 | 1.897657 |
| H | 0.652668 | -3.094932 | 2.127229 |
| H | -0.670559 | -2.158977 | 2.846769 |
| C | -3.446018 | 3.515635 | -2.269428 |
| C | -3.736330 | -4.717060 | -1.953258 |
| H | -3.443138 | -5.638539 | -2.455639 |
| C | -0.958006 | 4.299652 | 1.236757 |
| H | -1.963092 | 4.019349 | 0.925719 |
| C | -0.778297 | 5.088467 | 2.349875 |
| H | -1.643150 | 5.420176 | 2.920934 |
| C | -1.629674 | -4.786122 | -0.662741 |
| H | -1.347378 | -5.703155 | -1.178771 |
| C | -3.986463 | -1.421931 | 2.359980 |
| C | -3.945301 | 2.193967 | -2.256202 |
| H | -4.948700 | 2.004164 | -2.635484 |
| C | -3.874442 | 0.964470 | 2.908670 |
| H | -4.293840 | 1.835637 | 3.410808 |
| C | 0.148787 | 3.840345 | 0.477357 |
| C | -5.262513 | -2.877280 | -1.644287 |
| H | -6.183671 | -2.368403 | -1.921740 |
| C | -2.729716 | 1.073384 | 2.166346 |
| H | -2.224269 | 2.036296 | 2.081435 |
| C | 1.610616 | 5.061240 | 2.032849 |
| H | 2.616046 | 5.355199 | 2.333511 |
| C | 2.586108 | 3.753919 | 0.178658 |
| H | 3.582041 | 4.080269 | 0.479317 |
| C | 2.075012 | -1.055925 | 3.552057 |
| H | 2.661570 | -0.547565 | 4.328399 |
| H | 2.784693 | -1.597934 | 2.913587 |
| H | 1.434890 | -1.791943 | 4.052308 |
| C | -2.403197 | 6.112536 | -2.360824 |
| H | -1.994430 | 7.119508 | -2.415826 |
| C | -4.458640 | -2.344483 | -0.663261 |
| H | -4.749782 | -1.419161 | -0.169356 |
| C | -1.640742 | 5.098794 | -1.829817 |
| H | -0.635031 | 5.317251 | -1.479169 |
| C | 2.422484 | 2.867830 | -0.856148 |
| H | 3.282532 | 2.512883 | -1.427456 |
| C | -4.906174 | -4.079110 | -2.289734 |
| H | -5.555553 | -4.495089 | -3.057095 |
| C | -5.731045 | -2.790053 | 3.363964 |
| H | -6.195573 | -3.764203 | 3.501818 |
| C | 2.154733 | 1.115704 | 2.288167 |
| H | 1.606178 | 1.777665 | 1.609269 |
| H | 3.037279 | 0.746413 | 1.750933 |
| H | 2.505027 | 1.720964 | 3.135792 |
| C | -0.518008 | -2.496044 | -2.794258 |
| H | -0.731500 | -3.126465 | -3.667871 |
| H | -1.464424 | -2.376833 | -2.253807 |
| H | 0.181797 | -3.043283 | -2.152535 |
| C | 0.516055 | 5.478051 | 2.751912 |
| H | 0.643736 | 6.108749 | 3.629017 |
| C | -3.703158 | 5.859645 | -2.843173 |
| H | -4.296165 | 6.671805 | -3.258298 |
| C | -0.967028 | -0.435302 | -4.134443 |
| H | -1.223002 | -1.081345 | -4.984585 |
| H | -0.582949 | 0.503175 | -4.550045 |
| H | -1.898896 | -0.216999 | -3.600210 |
| C | -6.265802 | -1.661932 | 4.018767 |
| H | -7.145805 | -1.766680 | 4.649793 |
| C | 0.050678 | -1.159503 | -3.254798 |
| C | -0.767509 | -4.196879 | 0.226729 |
| H | 0.209232 | -4.641325 | 0.417869 |
| C | 0.165806 | 0.548179 | 3.665674 |
| H | -0.621642 | -0.176784 | 3.907973 |
| H | -0.311727 | 1.426638 | 3.215491 |
| H | 0.614498 | 0.875927 | 4.612541 |
| C | 1.349283 | -1.395661 | -4.027433 |
| H | 2.047159 | -1.998457 | -3.433886 |
| H | 1.845327 | -0.464187 | -4.329833 |
| H | 1.129887 | -1.960340 | -4.942551 |
| Ni | 1.903548 | -1.087247 | -0.356835 |
| H | 2.855701 | -1.350311 | 0.833687 |
| C | 4.271200 | -1.297231 | 0.029893 |
| N | 3.787727 | -0.439838 | -1.025931 |
| H | 3.667668 | 0.526480 | -0.710110 |
| C | 4.263503 | -0.388280 | -2.383143 |
| O | 4.126823 | 0.675540 | -2.953095 |
| C | 4.876450 | -1.597784 | -3.000514 |
| H | 4.166360 | -2.429004 | -3.068347 |
| H | 5.204742 | -1.327703 | -4.005472 |
| H | 5.731107 | -1.957072 | -2.417337 |
| C | 4.511898 | -2.662917 | -0.214654 |
| H | 5.323023 | -3.204839 | 0.252165 |
| N | 3.585113 | -3.446787 | -0.859205 |
| O | 3.810341 | -4.631185 | -1.106092 |
| O | 2.450032 | -2.905015 | -1.172287 |
| C | 5.124037 | -0.621822 | 1.035915 |

| | | | |
|---|---|---|---|
| C | 5.371971 | -1.268359 | 2.254743 |
| C | 5.665496 | 0.653628 | 0.821389 |
| C | 6.125957 | -0.650966 | 3.242297 |
| H | 4.947772 | -2.256929 | 2.428858 |
| C | 6.421439 | 1.269415 | 1.813034 |
| H | 5.528555 | 1.165393 | -0.131662 |
| C | 6.645942 | 0.624665 | 3.026772 |
| H | 6.302855 | -1.161977 | 4.186138 |
| H | 6.844449 | 2.254769 | 1.631204 |
| H | 7.231207 | 1.113141 | 3.802550 |

**VI$_{R1}$**

| | | | |
|---|---|---|---|
| P | 0.524861 | -0.099692 | -1.582059 |
| P | 0.231528 | -1.051984 | 1.300790 |
| C | -2.407397 | -0.017754 | 1.402693 |
| C | -2.692804 | -2.353492 | 0.469409 |
| C | -5.118081 | -2.527824 | 2.066916 |
| H | -4.737376 | -3.407800 | 1.553411 |
| C | -1.542222 | -3.072376 | 0.777182 |
| C | 0.129856 | 2.992094 | -0.467852 |
| C | -1.214798 | 2.803461 | -1.087049 |
| C | 0.992944 | -0.318522 | 2.851921 |
| C | -3.494122 | -2.763583 | -0.645219 |
| C | -1.793703 | 1.543252 | -1.236596 |
| C | -1.913940 | 3.958043 | -1.579292 |
| C | -3.095180 | 1.422113 | -1.781464 |
| H | -3.537117 | 0.427094 | -1.851727 |
| C | -1.053699 | 0.169850 | 0.758317 |
| H | -0.646574 | 1.128688 | 1.116080 |
| C | 1.260630 | 2.450203 | -1.069035 |
| C | 1.127478 | 1.506484 | -2.220823 |
| H | 2.090359 | 1.340064 | -2.715985 |
| H | 0.411558 | 1.870220 | -2.971101 |
| C | -4.889749 | -0.191850 | 2.741317 |
| C | -3.136612 | -1.200056 | 1.303973 |
| C | -3.166744 | -3.970127 | -1.344593 |
| C | 1.602701 | 4.067515 | 1.211388 |
| C | -1.122723 | 0.267548 | -0.784081 |
| H | -1.747934 | -0.574756 | -1.117414 |
| C | -6.116650 | -0.306356 | 3.436947 |
| H | -6.492061 | 0.564910 | 3.972746 |
| C | -3.924590 | 4.945236 | -2.602951 |
| H | -4.926460 | 4.807705 | -3.008257 |
| C | -0.612138 | -2.580527 | 1.844250 |
| H | 0.179652 | -3.309110 | 2.051475 |
| H | -1.142712 | -2.363741 | 2.782919 |
| C | -3.229843 | 3.807693 | -2.126729 |
| C | -3.945368 | -4.353312 | -2.463667 |
| H | -3.686563 | -5.274570 | -2.985117 |
| C | -0.823697 | 4.249626 | 1.468380 |
| H | -1.830024 | 4.041872 | 1.108340 |
| C | -0.645342 | 4.961142 | 2.632660 |
| H | -1.512819 | 5.303194 | 3.193546 |
| C | -2.042486 | -4.722316 | -0.927228 |
| H | -1.803698 | -5.648170 | -1.449769 |
| C | -4.376947 | -1.318460 | 2.019691 |
| C | -3.802602 | 2.518615 | -2.196452 |
| H | -4.808385 | 2.407842 | -2.599910 |
| C | -4.150666 | 1.012927 | 2.747882 |
| H | -4.551739 | 1.876410 | 3.277329 |
| C | 0.286001 | 3.777670 | 0.720944 |
| C | -5.314862 | -2.385151 | -2.214972 |
| H | -6.145885 | -1.772953 | -2.560006 |
| C | -2.940353 | 1.086145 | 2.113153 |
| H | -2.363807 | 2.012323 | 2.136669 |
| C | 1.749446 | 4.824071 | 2.398682 |
| H | 2.756370 | 5.042973 | 2.753157 |
| C | 2.726326 | 3.555776 | 0.519701 |
| H | 3.726209 | 3.797769 | 0.879715 |
| C | 1.846666 | -1.379812 | 3.545125 |
| H | 2.351298 | -0.925923 | 4.407416 |
| H | 2.613721 | -1.801847 | 2.885806 |
| H | 1.243216 | -2.213263 | 3.923760 |
| C | -2.039488 | 6.340447 | -2.063586 |
| H | -1.573294 | 7.323690 | -2.058742 |
| C | -4.590693 | -1.993836 | -1.112409 |
| H | -4.857297 | -1.076465 | -0.590347 |
| C | -1.343215 | 5.257316 | -1.580685 |
| H | -0.331804 | 5.398766 | -1.207273 |
| C | 2.554105 | 2.744053 | -0.572811 |
| H | 3.417458 | 2.365036 | -1.124407 |
| C | -4.994400 | -3.577506 | -2.896286 |
| H | -5.577904 | -3.879840 | -3.763130 |
| C | -6.300531 | -2.610634 | 2.763984 |
| H | -6.844413 | -3.552743 | 2.789145 |
| C | 1.840631 | 0.887571 | 2.457882 |
| H | 1.270386 | 1.614085 | 1.867958 |
| H | 2.725550 | 0.613465 | 1.867726 |

| | | | |
|---|---|---|---|
| H | 2.195264 | 1.407088 | 3.358068 |
| C | -0.280436 | -2.512787 | -2.626963 |
| H | -0.573487 | -3.138296 | -3.480877 |
| H | -1.135551 | -2.472900 | -1.945542 |
| H | 0.540300 | -3.023685 | -2.101494 |
| C | 0.651112 | 5.259539 | 3.100524 |
| H | 0.777811 | 5.831984 | 4.016843 |
| C | -3.346618 | 6.190112 | -2.570166 |
| H | -3.887653 | 7.057327 | -2.942670 |
| C | -1.051113 | -0.495894 | -3.871430 |
| H | -1.199533 | -1.057095 | -4.803500 |
| H | -0.879266 | 0.551598 | -4.146683 |
| H | -1.993441 | -0.551763 | -3.313410 |
| C | -6.813409 | -1.489984 | 3.448249 |
| H | -7.753965 | -1.568199 | 3.989330 |
| C | 0.113984 | -1.120477 | -3.102655 |
| C | -1.228250 | -4.262377 | 0.077549 |
| H | -0.327050 | -4.809126 | 0.354563 |
| C | -0.113430 | 0.158363 | 3.799212 |
| H | -0.859341 | -0.612704 | 4.026168 |
| H | -0.641090 | 1.034069 | 3.404826 |
| H | 0.345256 | 0.457864 | 4.750583 |
| C | 1.348786 | -1.210138 | -3.995938 |
| H | 2.169407 | -1.735649 | -3.494121 |
| H | 1.723298 | -0.235897 | -4.329144 |
| H | 1.099964 | -1.792331 | -4.892107 |
| Ni | 1.728205 | -1.274283 | -0.217720 |
| H | 3.829190 | -0.931435 | 1.287216 |
| C | 4.449812 | -1.250412 | 0.431169 |
| N | 3.624246 | -0.720460 | -0.739263 |
| H | 3.486300 | 0.287770 | -0.600202 |
| C | 4.129542 | -0.834986 | -2.087184 |
| O | 4.029480 | 0.133093 | -2.814283 |
| C | 4.696874 | -2.147485 | -2.513971 |
| H | 4.070608 | -2.993953 | -2.211382 |
| H | 4.825480 | -2.135168 | -3.598211 |
| H | 5.678664 | -2.299073 | -2.048021 |
| C | 4.526834 | -2.724346 | 0.427770 |
| H | 5.449784 | -3.276081 | 0.304093 |
| N | 3.434084 | -3.462886 | 0.526094 |
| O | 3.378674 | -4.694379 | 0.515578 |
| O | 2.219276 | -2.831284 | 0.664305 |
| C | 5.801792 | -0.582403 | 0.558405 |
| C | 6.708791 | -1.093211 | 1.494423 |
| C | 6.151167 | 0.566576 | -0.156906 |
| C | 7.936981 | -0.477035 | 1.702829 |
| H | 6.442212 | -1.978172 | 2.071416 |
| C | 7.386004 | 1.179234 | 0.048803 |
| H | 5.479898 | 0.999613 | -0.896849 |
| C | 8.281625 | 0.661150 | 0.977181 |
| H | 8.628576 | -0.890640 | 2.433598 |
| H | 7.644836 | 2.065664 | -0.526382 |
| H | 9.245146 | 1.140520 | 1.135336 |

**VII$_{R1}$**

| | | | |
|---|---|---|---|
| P | -0.011576 | 0.113499 | -1.727715 |
| P | 0.189226 | -0.987431 | 1.077759 |
| C | -2.530108 | -0.310680 | 1.572381 |
| C | -2.654821 | -2.581708 | 0.478993 |
| C | -4.896440 | -3.148983 | 2.248341 |
| H | -4.501327 | -3.928726 | 1.601608 |
| C | -1.399355 | -3.154966 | 0.646591 |
| C | -0.701463 | 3.070817 | -0.346109 |
| C | -2.045696 | 2.715165 | -0.883967 |
| C | 1.116503 | -0.228101 | 2.511809 |
| C | -3.483510 | -3.016796 | -0.604895 |
| C | -2.456926 | 1.396350 | -1.067875 |
| C | -2.927912 | 3.783526 | -1.268064 |
| C | -3.758419 | 1.119672 | -1.552745 |
| H | -4.060156 | 0.078234 | -1.667361 |
| C | -1.301241 | 0.079311 | 0.783040 |
| H | -0.979367 | 1.072043 | 1.132294 |
| C | 0.452930 | 2.749806 | -1.053715 |
| C | 0.403672 | 1.824691 | -2.229570 |
| H | 1.370560 | 1.780187 | -2.744061 |
| H | -0.356493 | 2.124598 | -2.967489 |
| C | -4.802325 | -0.883014 | 3.155635 |
| C | -3.149362 | -1.551579 | 1.435604 |
| C | -3.050085 | -4.099414 | -1.436514 |
| C | 0.699963 | 4.327098 | 1.268366 |
| C | -1.579694 | 0.212717 | -0.735622 |
| H | -2.116073 | -0.698516 | -1.037530 |
| C | -5.921551 | -1.195691 | 3.963880 |
| H | -6.308033 | -0.428528 | 4.633953 |
| C | -5.111118 | 4.527382 | -2.130190 |
| H | -6.106584 | 4.270679 | -2.491130 |
| C | -0.453568 | -2.599860 | 1.667913 |
| H | 0.404016 | -3.263321 | 1.823004 |

| | | | |
|---|---|---|---|
| H | -0.944969 | -2.445264 | 2.639010 |
| C | -4.239672 | 3.476964 | -1.755802 |
| C | -3.855941 | -4.498713 | -2.530089 |
| H | -3.514283 | -5.324333 | -3.153669 |
| C | -1.691741 | 4.064051 | 1.720088 |
| H | -2.674400 | 3.702255 | 1.421135 |
| C | -1.540591 | 4.737305 | 2.910610 |
| H | -2.404604 | 4.899446 | 3.552260 |
| C | -1.809162 | -4.721020 | -1.161649 |
| H | -1.490154 | -5.559194 | -1.780094 |
| C | -4.280302 | -1.870763 | 2.258901 |
| C | -4.631719 | 2.124591 | -1.870990 |
| H | -5.633533 | 1.893482 | -2.231367 |
| C | -4.176687 | 0.382037 | 3.221014 |
| H | -4.582400 | 1.137592 | 3.892693 |
| C | -0.582965 | 3.825586 | 0.867514 |
| C | -5.468159 | -2.787895 | -1.992886 |
| H | -6.405468 | -2.281807 | -2.216086 |
| C | -3.066833 | 0.650528 | 2.463333 |
| H | -2.582688 | 1.626104 | 2.529260 |
| C | 0.814517 | 5.033028 | 2.490801 |
| H | 1.794384 | 5.411961 | 2.780022 |
| C | 1.821262 | 4.085094 | 0.439679 |
| H | 2.790466 | 4.509456 | 0.701857 |
| C | 2.242371 | -1.176562 | 2.930562 |
| H | 2.807746 | -0.725297 | 3.755742 |
| H | 2.954944 | -1.355019 | 2.113791 |
| H | 1.869945 | -2.146593 | 3.283948 |
| C | -3.409951 | 6.146123 | -1.603026 |
| H | -3.084944 | 7.183645 | -1.563818 |
| C | -4.716108 | -2.385787 | -0.912902 |
| H | -5.061874 | -1.566230 | -0.285024 |
| C | -2.543286 | 5.148880 | -1.221211 |
| H | -1.541204 | 5.411871 | -0.891652 |
| C | 1.700805 | 3.299819 | -0.678560 |
| H | 2.573636 | 3.146287 | -1.314489 |
| C | -5.037766 | -3.854659 | -2.809248 |
| H | -5.643037 | -4.165130 | -3.658013 |
| C | -5.976307 | -3.424188 | 3.053839 |
| H | -6.426439 | -4.414561 | 3.029241 |
| C | 1.705923 | 1.096684 | 2.035609 |
| H | 0.935985 | 1.773425 | 1.649580 |
| H | 2.452069 | 0.959820 | 1.240374 |
| H | 2.201980 | 1.611792 | 2.869310 |
| C | -0.249146 | -2.290277 | -3.058440 |
| H | -0.579145 | -2.862014 | -3.936398 |
| H | -0.867260 | -2.606711 | -2.211051 |
| H | 0.794918 | -2.567888 | -2.855173 |
| C | -0.277807 | 5.229166 | 3.300959 |
| H | -0.173279 | 5.766865 | 4.241003 |
| C | -4.710016 | 5.838498 | -2.052123 |
| H | -5.386074 | 6.638562 | -2.346268 |
| C | -1.819075 | -0.486911 | -3.782881 |
| H | -1.967793 | -0.924058 | -4.778688 |
| H | -2.025167 | 0.587895 | -3.862049 |
| H | -2.574721 | -0.931259 | -3.122347 |
| C | -6.502739 | -2.438999 | 3.914126 |
| H | -7.361039 | -2.669562 | 4.541319 |
| C | -0.390780 | -0.795328 | -3.328021 |
| C | -0.993130 | -4.235385 | -0.171699 |
| H | -0.011881 | -4.679834 | -0.006000 |
| C | 0.174117 | 0.032437 | 3.687721 |
| H | -0.355067 | -0.866135 | 4.027394 |
| H | -0.574426 | 0.797986 | 3.450715 |
| H | 0.759656 | 0.406211 | 4.537903 |
| C | 0.606715 | -0.361307 | -4.402878 |
| H | 1.645016 | -0.528405 | -4.097348 |
| H | 0.480045 | 0.689590 | -4.687553 |
| H | 0.426166 | -0.962440 | -5.303024 |
| Ni | 1.435973 | -1.027768 | -0.658850 |
| H | 3.853777 | -0.433613 | 0.276777 |
| C | 4.783113 | -0.121921 | -0.239041 |
| N | 4.533081 | 1.303444 | -0.481018 |
| H | 3.554291 | 1.522249 | -0.630436 |
| C | 5.367581 | 2.232450 | -1.068285 |
| O | 4.907325 | 3.302404 | -1.447775 |
| C | 6.830933 | 1.896302 | -1.161993 |
| H | 7.005684 | 0.888138 | -1.555623 |
| H | 7.314335 | 2.628221 | -1.811214 |
| H | 7.303302 | 1.934734 | -0.173077 |
| C | 4.865181 | -0.968917 | -1.476883 |
| H | 5.792105 | -1.271678 | -1.956729 |
| N | 3.793224 | -1.096831 | -2.275294 |
| O | 3.737993 | -1.778864 | -3.302371 |
| O | 2.685515 | -0.409061 | -1.903442 |
| C | 5.911847 | -0.344736 | 0.750815 |
| C | 6.889060 | -1.329938 | 0.598669 |
| C | 5.942555 | 0.457322 | 1.898560 |

| | | | |
|---|---|---|---|
| C | 7.882316 | -1.499400 | 1.562734 |
| H | 6.899475 | -1.978609 | -0.275564 |
| C | 6.930620 | 0.288787 | 2.859772 |
| H | 5.190458 | 1.237878 | 2.014297 |
| C | 7.908681 | -0.691076 | 2.692928 |
| H | 8.641677 | -2.265285 | 1.419913 |
| H | 6.942532 | 0.928148 | 3.740412 |
| H | 8.688225 | -0.820267 | 3.440641 |
| C | 3.193982 | -3.547045 | 0.171087 |
| C | 2.883867 | -4.790807 | 0.932029 |
| H | 3.134718 | -5.671769 | 0.333124 |
| H | 3.511748 | -4.833665 | 1.828548 |
| H | 1.832905 | -4.830247 | 1.222051 |
| O | 4.442498 | -3.486050 | -0.215047 |
| H | 4.612545 | -2.617346 | -0.702202 |
| O | 2.350198 | -2.667727 | -0.050806 |

**TSIII$_{R1}$**

| | | | |
|---|---|---|---|
| P | 0.040316 | 0.168751 | -1.756523 |
| P | 0.312488 | -0.932135 | 1.064717 |
| C | -2.430559 | -0.382399 | 1.569373 |
| C | -2.467988 | -2.653619 | 0.470590 |
| C | -4.663626 | -3.320888 | 2.266352 |
| H | -4.239915 | -4.082293 | 1.615865 |
| C | -1.184418 | -3.167428 | 0.616418 |
| C | -0.780570 | 3.084842 | -0.348491 |
| C | -2.108286 | 2.666419 | -0.881366 |
| C | 1.185412 | -0.146347 | 2.517393 |
| C | -3.290766 | -3.120763 | -0.604407 |
| C | -2.461390 | 1.330082 | -1.054413 |
| C | -3.041757 | 3.693145 | -1.256551 |
| C | -3.762416 | 0.993897 | -1.501631 |
| H | -4.023078 | -0.060326 | -1.597450 |
| C | -1.224101 | 0.064731 | 0.773419 |
| H | -0.948033 | 1.070650 | 1.123974 |
| C | 0.388160 | 2.824240 | -1.056922 |
| C | 0.376609 | 1.906005 | -2.240431 |
| H | 1.336875 | 1.915125 | -2.770406 |
| H | -0.404326 | 2.177319 | -2.967965 |
| C | -4.662026 | -1.052500 | 3.171700 |
| C | -2.995795 | -1.649183 | 1.436994 |
| C | -2.816813 | -4.174819 | -1.451467 |
| C | 0.555858 | 4.394821 | 1.278395 |
| C | -1.519447 | 0.191284 | -0.741516 |
| H | -2.010434 | -0.744997 | -1.044650 |
| C | -5.759729 | -1.413038 | 3.989247 |
| H | -6.173947 | -0.662841 | 4.661909 |
| C | -5.268775 | 4.336583 | -2.084554 |
| H | -6.259012 | 4.035772 | -2.425154 |
| C | -0.254514 | -2.579881 | 1.633199 |
| H | 0.635154 | -3.202330 | 1.771138 |
| H | -0.745402 | -2.454532 | 2.608618 |
| C | -4.347947 | 3.326804 | -1.716596 |
| C | -3.617022 | -4.602141 | -2.538683 |
| H | -3.243648 | -5.403841 | -3.175244 |
| C | -1.821625 | 4.017010 | 1.720853 |
| H | -2.785420 | 3.612089 | 1.416764 |
| C | -1.705256 | 4.687458 | 2.916919 |
| H | -2.577533 | 4.803334 | 3.557354 |
| C | -1.543999 | -4.738650 | -1.197736 |
| H | -1.194454 | -5.556958 | -1.826347 |
| C | -4.104140 | -2.016912 | 2.271426 |
| C | -4.685179 | 1.957752 | -1.807451 |
| H | -5.684828 | 1.680289 | -2.139960 |
| C | -4.094000 | 0.239735 | 3.229610 |
| H | -4.527698 | 0.978094 | 3.902882 |
| C | -0.700636 | 3.836540 | 0.870245 |
| C | -5.304041 | -2.978457 | -1.962732 |
| H | -6.269024 | -2.518624 | -2.166914 |
| C | -3.004024 | 0.555732 | 2.461877 |
| H | -2.564438 | 1.552263 | 2.522190 |
| C | 0.634424 | 5.096876 | 2.506064 |
| H | 1.594709 | 5.519356 | 2.800489 |
| C | 1.688328 | 4.211952 | 0.450715 |
| H | 2.635869 | 4.679633 | 0.718177 |
| C | 2.339048 | -1.053211 | 2.950286 |
| H | 2.875218 | -0.580836 | 3.783277 |
| H | 3.071288 | -1.210844 | 2.148433 |
| H | 1.992894 | -2.034275 | 3.299520 |
| C | -3.627006 | 6.030023 | -1.602242 |
| H | -3.344969 | 7.080405 | -1.578107 |
| C | -4.557520 | -2.549392 | -0.889635 |
| H | -4.935182 | -1.754408 | -0.248784 |
| C | -2.713535 | 5.073287 | -1.226183 |
| H | -1.718117 | 5.380227 | -0.914660 |
| C | 1.607039 | 3.432033 | -0.675532 |
| H | 2.489325 | 3.327012 | -1.307657 |
| C | -4.832394 | -4.013978 | -2.796257 |

| | | | |
|---|---|---|---|
| H | -5.433004 | -4.345460 | -3.640403 |
| C | -5.724130 | -3.642598 | 3.080414 |
| H | -6.130648 | -4.651725 | 3.059995 |
| C | 1.717550 | 1.208153 | 2.060366 |
| H | 0.920898 | 1.854563 | 1.674410 |
| H | 2.478514 | 1.114559 | 1.274886 |
| H | 2.179462 | 1.736162 | 2.905754 |
| C | -0.185188 | -2.216224 | -3.122914 |
| H | -0.537022 | -2.786214 | -3.993488 |
| H | -0.768860 | -2.550802 | -2.257433 |
| H | 0.868017 | -2.474239 | -2.954140 |
| C | -0.467850 | 5.235189 | 3.314627 |
| H | -0.391300 | 5.770867 | 4.258478 |
| C | -4.920514 | 5.663836 | -2.026027 |
| H | -5.634406 | 6.431901 | -2.316045 |
| C | -1.823984 | -0.451197 | -3.763665 |
| H | -1.990739 | -0.877127 | -4.761402 |
| H | -2.068001 | 0.616987 | -3.818568 |
| H | -2.542166 | -0.932482 | -3.088471 |
| C | -6.286354 | -2.680670 | 3.944425 |
| H | -7.129391 | -2.947446 | 4.578019 |
| C | -0.369076 | -0.722146 | -3.368430 |
| C | -0.738517 | -4.223213 | -0.214182 |
| H | 0.262060 | -4.626456 | -0.058160 |
| C | 0.216903 | 0.067876 | 3.681914 |
| H | -0.290985 | -0.849223 | 4.002807 |
| H | -0.548502 | 0.816986 | 3.447767 |
| H | 0.782410 | 0.444497 | 4.544288 |
| C | 0.550525 | -0.241097 | -4.492463 |
| H | 1.608642 | -0.394931 | -4.266916 |
| H | 0.384737 | 0.814390 | -4.737952 |
| H | 0.314535 | -0.818339 | -5.395535 |
| Ni | 1.544672 | -0.974743 | -0.699092 |
| H | 3.561839 | -0.397194 | 0.136900 |
| C | 4.541647 | -0.071522 | -0.260863 |
| N | 4.316500 | 1.350481 | -0.528545 |
| H | 3.349589 | 1.613670 | -0.688967 |
| C | 5.206075 | 2.315766 | -0.956291 |
| O | 4.785391 | 3.408161 | -1.316207 |
| C | 6.674360 | 1.991415 | -0.907359 |
| H | 6.905877 | 0.980860 | -1.260504 |
| H | 7.208451 | 2.721927 | -1.517542 |
| H | 7.046306 | 2.059596 | 0.122072 |
| C | 4.810459 | -0.898454 | -1.496807 |
| H | 5.811356 | -0.893209 | -1.925466 |
| N | 3.882445 | -0.898855 | -2.503902 |
| O | 4.150150 | -1.161702 | -3.670993 |
| O | 2.606831 | -0.660688 | -2.227486 |
| C | 5.558176 | -0.300559 | 0.841991 |
| C | 6.520673 | -1.310721 | 0.781103 |
| C | 5.511783 | 0.511337 | 1.982650 |
| C | 7.415673 | -1.504222 | 1.831652 |
| H | 6.593153 | -1.956947 | -0.090219 |
| C | 6.401962 | 0.317069 | 3.031750 |
| H | 4.779013 | 1.317471 | 2.028316 |
| C | 7.360261 | -0.693340 | 2.959240 |
| H | 8.165626 | -2.289000 | 1.757416 |
| H | 6.355203 | 0.963290 | 3.906297 |
| H | 8.064693 | -0.840121 | 3.775266 |
| C | 3.329987 | -3.439207 | 0.071187 |
| C | 3.186129 | -4.656909 | 0.924807 |
| H | 3.485348 | -5.552502 | 0.373531 |
| H | 3.869787 | -4.567928 | 1.777549 |
| H | 2.167337 | -4.768957 | 1.301575 |
| O | 4.446843 | -3.373890 | -0.572078 |
| H | 4.592301 | -2.390780 | -1.013117 |
| O | 2.427249 | -2.572650 | 0.048027 |

**VIII$_{R1}$**

| | | | |
|---|---|---|---|
| P | -0.224077 | -0.096121 | -1.800649 |
| P | 0.086716 | -1.054599 | 1.063771 |
| C | -2.553247 | -0.160404 | 1.643426 |
| C | -2.917950 | -2.451545 | 0.643195 |
| C | -5.131772 | -2.743041 | 2.534480 |
| H | -4.830317 | -3.585958 | 1.917407 |
| C | -1.691884 | -3.098046 | 0.759435 |
| C | -0.372178 | 2.975958 | -0.441539 |
| C | -1.781706 | 2.797749 | -0.899527 |
| C | 1.118502 | -0.379267 | 2.462331 |
| C | -3.836296 | -2.884527 | -0.370301 |
| C | -2.400578 | 1.552438 | -0.991449 |
| C | -2.527670 | 3.973891 | -1.257202 |
| C | -3.774796 | 1.464092 | -1.323455 |
| H | -4.238923 | 0.476561 | -1.348858 |
| C | -1.330339 | 0.109375 | 0.797941 |
| H | -0.918646 | 1.077440 | 1.119446 |
| C | 0.701282 | 2.459766 | -1.162454 |
| C | 0.469664 | 1.526189 | -2.309805 |

| | | | |
|---|---|---|---|
| H | 1.401846 | 1.319294 | -2.847072 |
| H | -0.248534 | 1.949324 | -3.028940 |
| C | -4.809925 | -0.453034 | 3.316114 |
| C | -3.283464 | -1.347176 | 1.577012 |
| C | -3.523729 | -4.039899 | -1.158592 |
| C | 1.242677 | 4.130688 | 1.044351 |
| C | -1.679592 | 0.256660 | -0.704866 |
| H | -2.361763 | -0.571432 | -0.952368 |
| C | -5.920604 | -0.624445 | 4.176413 |
| H | -6.212446 | 0.207406 | 4.816504 |
| C | -4.653932 | 5.018832 | -1.927367 |
| H | -5.710061 | 4.907994 | -2.171251 |
| C | -0.656726 | -2.596414 | 1.716536 |
| H | 0.149822 | -3.326350 | 1.850468 |
| H | -1.086863 | -2.374930 | 2.703477 |
| C | -3.915121 | 3.858459 | -1.595486 |
| C | -4.419148 | -4.450190 | -2.174720 |
| H | -4.168620 | -5.333748 | -2.761074 |
| C | -1.134515 | 4.170123 | 1.617497 |
| H | -2.165920 | 3.905993 | 1.388947 |
| C | -0.847166 | 4.885809 | 2.757796 |
| H | -1.652869 | 5.174776 | 3.430082 |
| C | -2.295059 | -4.708946 | -0.948921 |
| H | -2.065896 | -5.590386 | -1.546847 |
| C | -4.407423 | -1.525046 | 2.454735 |
| C | -4.518145 | 2.580435 | -1.597221 |
| H | -5.577859 | 2.496034 | -1.835027 |
| C | -4.076977 | 0.754410 | 3.301147 |
| H | -4.394426 | 1.574193 | 3.944360 |
| C | -0.106469 | 3.765255 | 0.727694 |
| C | -5.866906 | -2.589097 | -1.678742 |
| H | -6.771468 | -2.023584 | -1.893704 |
| C | -2.973624 | 0.884663 | 2.501508 |
| H | -2.402787 | 1.814891 | 2.502692 |
| C | 1.499681 | 4.891244 | 2.210129 |
| H | 2.530198 | 5.168564 | 2.429613 |
| C | 2.292093 | 3.693423 | 0.201443 |
| H | 3.319958 | 4.000336 | 0.397572 |
| C | 2.152858 | -1.443147 | 2.838390 |
| H | 2.859006 | -1.022229 | 3.566128 |
| H | 2.730952 | -1.792503 | 1.974209 |
| H | 1.693166 | -2.322413 | 3.304899 |
| C | -2.681823 | 6.370892 | -1.656858 |
| H | -2.201329 | 7.346140 | -1.702078 |
| C | -5.032055 | -2.179777 | -0.663696 |
| H | -5.285191 | -1.299935 | -0.074084 |
| C | -1.939296 | 5.263973 | -1.317463 |
| H | -0.879454 | 5.380089 | -1.104994 |
| C | 2.026193 | 2.850295 | -0.847222 |
| H | 2.842195 | 2.497143 | -1.476952 |
| C | -5.564709 | -3.738357 | -2.437760 |
| H | -6.237446 | -4.055990 | -3.231529 |
| C | -6.198627 | -2.882260 | 3.391162 |
| H | -6.730717 | -3.830421 | 3.436180 |
| C | 1.803264 | 0.884898 | 1.959871 |
| H | 1.075480 | 1.670726 | 1.730292 |
| H | 2.387714 | 0.706776 | 1.049714 |
| H | 2.470070 | 1.284785 | 2.737335 |
| C | -1.282209 | -2.267648 | -3.113278 |
| H | -1.783593 | -2.694110 | -3.992698 |
| H | -1.984620 | -2.339432 | -2.274201 |
| H | -0.408880 | -2.887287 | -2.882061 |
| C | 0.479897 | 5.257672 | 3.055849 |
| H | 0.693146 | 5.833475 | 3.953942 |
| C | -4.054870 | 6.254286 | -1.952769 |
| H | -4.631897 | 7.139141 | -2.212271 |
| C | -2.135523 | -0.055259 | -3.859336 |
| H | -2.397674 | -0.405557 | -4.866474 |
| H | -1.988834 | 1.030324 | -3.919036 |
| H | -3.003059 | -0.244125 | -3.215915 |
| C | -6.606966 | -1.813123 | 4.214112 |
| H | -7.455986 | -1.936291 | 4.883115 |
| C | -0.896871 | -0.817630 | -3.385659 |
| C | -1.387134 | -4.222889 | -0.043763 |
| H | -0.423525 | -4.715223 | 0.094629 |
| C | 0.251875 | -0.027644 | 3.672702 |
| H | -0.314997 | -0.880808 | 4.063687 |
| H | -0.456793 | 0.779252 | 3.449493 |
| H | 0.902516 | 0.328200 | 4.482522 |
| C | 0.209428 | -0.732461 | -4.440932 |
| H | 1.152633 | -1.175080 | -4.101143 |
| H | 0.403817 | 0.301638 | -4.748558 |
| H | -0.109313 | -1.280943 | -5.336296 |
| Ni | 1.081869 | -1.478137 | -0.780567 |
| H | 4.177100 | -0.564652 | 0.854202 |
| C | 5.107181 | -0.296977 | 0.332904 |
| N | 5.102674 | 1.157332 | 0.316042 |
| H | 4.329521 | 1.602397 | 0.798269 |

| | | | |
|---|---|---|---|
| C | 5.801778 | 2.055849 | -0.465311 |
| O | 5.488951 | 3.237885 | -0.454506 |
| C | 6.929650 | 1.535043 | -1.318280 |
| H | 7.401570 | 0.627528 | -0.927690 |
| H | 6.559525 | 1.330061 | -2.331252 |
| H | 7.682732 | 2.320926 | -1.409045 |
| C | 4.967737 | -0.894189 | -1.073874 |
| H | 5.843223 | -0.766704 | -1.711564 |
| N | 3.819979 | -0.258673 | -1.786630 |
| O | 3.929327 | 0.035461 | -2.969481 |
| O | 2.792220 | -0.070229 | -1.106365 |
| C | 6.253314 | -0.956518 | 1.076917 |
| C | 6.217071 | -2.342188 | 1.271251 |
| C | 7.318122 | -0.227695 | 1.604506 |
| C | 7.237908 | -2.988633 | 1.958313 |
| H | 5.366602 | -2.920108 | 0.900955 |
| C | 8.342067 | -0.875356 | 2.292497 |
| H | 7.337542 | 0.854684 | 1.492698 |
| C | 8.309093 | -2.255224 | 2.464944 |
| H | 7.194269 | -4.066078 | 2.104402 |
| H | 9.167549 | -0.293847 | 2.697813 |
| H | 9.110541 | -2.759290 | 3.000378 |
| C | 2.333160 | -3.318849 | -1.373096 |
| C | 3.031988 | -4.578326 | -1.739450 |
| H | 3.621276 | -4.443452 | -2.650522 |
| H | 3.661422 | -4.933851 | -0.920113 |
| H | 2.284671 | -5.352860 | -1.950887 |
| O | 1.881281 | -2.525292 | -2.261173 |
| H | 4.729376 | -1.963186 | -1.015786 |
| O | 2.142472 | -2.995092 | -0.147131 |

**IX$_{S2}$**

| | | | |
|---|---|---|---|
| P | 0.388689 | -0.687885 | 1.560503 |
| P | 0.813839 | 0.017351 | -1.475101 |
| C | -1.817138 | 1.229114 | -1.398228 |
| C | -0.304663 | 3.062851 | -0.573383 |
| C | -2.082514 | 4.938239 | -1.941132 |
| H | -1.151064 | 5.293423 | -1.506316 |
| C | 0.960719 | 2.709302 | -1.027582 |
| C | -2.209142 | -2.573458 | 0.448225 |
| C | -2.900364 | -1.477925 | 1.190411 |
| C | 0.601843 | -1.050102 | -3.016353 |
| C | -0.427800 | 3.899532 | 0.584271 |
| C | -2.370586 | -0.192249 | 1.299237 |
| C | -4.147103 | -1.775223 | 1.842183 |
| C | -3.096179 | 0.821283 | 1.972094 |
| H | -2.660579 | 1.820715 | 2.025028 |
| C | -0.947560 | 0.142109 | -0.803168 |
| H | -1.381157 | -0.820454 | -1.109231 |
| C | -0.963609 | -3.042842 | 0.858056 |
| C | -0.229725 | -2.368369 | 1.975228 |
| H | 0.631696 | -2.963896 | 2.297563 |
| H | -0.896844 | -2.250026 | 2.838611 |
| C | -3.598963 | 3.116915 | -2.529116 |
| C | -1.518059 | 2.586351 | -1.293048 |
| C | 0.750945 | 4.427002 | 1.205161 |
| C | -2.279931 | -4.376499 | -1.250910 |
| C | -1.019431 | 0.193726 | 0.745256 |
| H | -0.834424 | 1.241288 | 1.021775 |
| C | -4.464050 | 4.070067 | -3.117562 |
| H | -5.386209 | 3.717711 | -3.578557 |
| C | -6.100139 | -1.019642 | 3.138976 |
| H | -6.631512 | -0.207206 | 3.633327 |
| C | 1.105464 | 1.719991 | -2.142251 |
| H | 2.106219 | 1.765735 | -2.588625 |
| H | 0.378001 | 1.915109 | -2.941326 |
| C | -4.864619 | -0.731097 | 2.511225 |
| C | 0.627063 | 5.236372 | 2.359776 |
| H | 1.533870 | 5.637354 | 2.812008 |
| C | -4.046952 | -2.686556 | -1.241461 |
| H | -4.501319 | -1.796287 | -0.809367 |
| C | -4.628298 | -3.288275 | -2.334057 |
| H | -5.535554 | -2.866905 | -2.762632 |
| C | 2.016864 | 4.097398 | 0.666850 |
| H | 2.912737 | 4.519153 | 1.121932 |
| C | -2.385889 | 3.552589 | -1.904655 |
| C | -4.314342 | 0.569516 | 2.545655 |
| H | -4.865320 | 1.363844 | 3.047833 |
| C | -3.900591 | 1.736903 | -2.549152 |
| H | -4.832883 | 1.405647 | -3.005241 |
| C | -2.856174 | -3.201736 | -0.667079 |
| C | -1.766453 | 4.965054 | 2.316306 |
| H | -2.740334 | 5.169036 | 2.757116 |
| C | -3.026980 | 0.826480 | -2.015990 |
| H | -3.262794 | -0.238233 | -2.048271 |
| C | -2.916221 | -4.983925 | -2.359768 |
| H | -2.468233 | -5.879997 | -2.788782 |
| C | -1.065376 | -4.878662 | -0.726611 |

| | | | |
|---|---|---|---|
| H | -0.643069 | -5.788772 | -1.153211 |
| C | 1.903135 | -1.054842 | -3.815628 |
| H | 1.766905 | -1.653906 | -4.725293 |
| H | 2.721707 | -1.514250 | -3.248086 |
| H | 2.207138 | -0.051021 | -4.140998 |
| C | -5.894585 | -3.333474 | 2.512733 |
| H | -6.288343 | -4.347713 | 2.532559 |
| C | -1.681847 | 4.193575 | 1.179066 |
| H | -2.588841 | 3.796997 | 0.726425 |
| C | -4.697385 | -3.082959 | 1.883776 |
| H | -4.159309 | -3.905127 | 1.418844 |
| C | -0.409563 | -4.205608 | 0.272262 |
| H | 0.547521 | -4.565197 | 0.648238 |
| C | -0.604672 | 5.500551 | 2.909282 |
| H | -0.685469 | 6.117990 | 3.801195 |
| C | -2.938736 | 5.838281 | -2.531350 |
| H | -2.678066 | 6.894436 | -2.550022 |
| C | 0.302336 | -2.468066 | -2.545623 |
| H | -0.667054 | -2.533038 | -2.041226 |
| H | 1.058949 | -2.843855 | -1.847249 |
| H | 0.258196 | -3.150348 | -3.405782 |
| C | 1.988475 | -0.410953 | 3.812134 |
| H | 2.076372 | -0.109355 | 4.863911 |
| H | 2.865839 | -0.011319 | 3.288614 |
| H | 2.056384 | -1.506773 | 3.780590 |
| C | -4.063939 | -4.451392 | -2.896807 |
| H | -4.537937 | -4.923639 | -3.754633 |
| C | -6.612637 | -2.293450 | 3.136951 |
| H | -7.561938 | -2.504690 | 3.624642 |
| C | 0.706739 | 1.628704 | 3.091988 |
| H | -0.286195 | 2.057252 | 2.900263 |
| H | 1.374005 | 1.963034 | 2.287781 |
| H | 1.072942 | 2.080502 | 4.023123 |
| C | -4.146592 | 5.406427 | -3.116430 |
| H | -4.817810 | 6.130681 | -3.572885 |
| C | 0.657481 | 0.109702 | 3.250954 |
| C | 2.113926 | 3.244329 | -0.403576 |
| H | 3.094557 | 3.006017 | -0.818375 |
| C | -0.540881 | -0.575552 | -3.913941 |
| H | -0.430697 | 0.461589 | -4.252569 |
| H | -1.517182 | -0.672143 | -3.427232 |
| H | -0.569007 | -1.205470 | -4.813155 |
| C | -0.455831 | -0.245323 | 4.244996 |
| H | -0.391434 | -1.282999 | 4.589677 |
| H | -1.461516 | -0.073199 | 3.844192 |
| H | -0.344243 | 0.389823 | 5.133205 |
| Ni | 2.178718 | -0.571876 | 0.228116 |
| H | 4.112054 | 1.340069 | -1.393165 |
| C | 6.178173 | 0.970546 | 2.110882 |
| C | 5.384116 | 0.778126 | 0.990177 |
| C | 4.952801 | -0.505492 | 0.607378 |
| C | 5.366215 | -1.585241 | 1.399982 |
| C | 6.159751 | -1.393432 | 2.527421 |
| C | 6.567486 | -0.116207 | 2.893278 |
| H | 6.499825 | 1.976659 | 2.372116 |
| H | 5.085189 | 1.652628 | 0.411982 |
| H | 5.080689 | -2.601126 | 1.136622 |
| H | 6.464408 | -2.255057 | 3.117812 |
| H | 7.189637 | 0.033713 | 3.772608 |
| C | 4.022846 | -0.676949 | -0.569593 |
| C | 4.308329 | 0.303999 | -1.671484 |
| N | 4.059595 | -2.050180 | -1.084718 |
| H | 4.876356 | -2.320076 | -1.631252 |
| N | 5.772710 | 0.289093 | -2.110542 |
| O | 6.285450 | -0.821660 | -2.260526 |
| O | 6.322909 | 1.360798 | -2.293815 |
| H | 3.752057 | 0.074442 | -2.582916 |
| H | 2.831209 | 0.432533 | 1.364413 |
| C | 3.305379 | -2.961567 | -0.467260 |
| C | 3.482307 | -4.410677 | -0.770148 |
| H | 2.538322 | -4.819900 | -1.149033 |
| H | 4.270337 | -4.602176 | -1.502599 |
| H | 3.715101 | -4.953950 | 0.151855 |
| O | 2.429741 | -2.578057 | 0.353200 |
| H | 2.523909 | 0.926472 | 0.776904 |

**TSV$_{S2}$**

| | | | |
|---|---|---|---|
| P | 0.391117 | -0.674421 | 1.583083 |
| P | 0.783425 | 0.002043 | -1.489267 |
| C | -1.832505 | 1.223672 | -1.383876 |
| C | -0.327568 | 3.064203 | -0.555717 |
| C | -2.103342 | 4.927994 | -1.946713 |
| H | -1.174501 | 5.287645 | -1.510108 |
| C | 0.942264 | 2.703506 | -0.991792 |
| C | -2.183029 | -2.598353 | 0.501328 |
| C | -2.884380 | -1.500444 | 1.226657 |
| C | 0.559418 | -1.025259 | -3.051273 |
| C | -0.459286 | 3.915282 | 0.591300 |

| | | | |
|---|---|---|---|
| C | -2.384289 | -0.201504 | 1.294592 |
| C | -4.136454 | -1.801313 | 1.867013 |
| C | -3.166504 | 0.826522 | 1.875903 |
| H | -2.765888 | 1.841197 | 1.876570 |
| C | -0.959718 | 0.139074 | -0.789529 |
| H | -1.399925 | -0.824695 | -1.082529 |
| C | -0.933660 | -3.056253 | 0.905835 |
| C | -0.185586 | -2.368806 | 2.007338 |
| H | 0.703417 | -2.943609 | 2.291050 |
| H | -0.815702 | -2.272708 | 2.903747 |
| C | -3.613556 | 3.100063 | -2.529567 |
| C | -1.536104 | 2.581528 | -1.280914 |
| C | 0.715172 | 4.453162 | 1.212776 |
| C | -2.253485 | -4.406184 | -1.192876 |
| C | -1.024805 | 0.193095 | 0.760290 |
| H | -0.847996 | 1.241811 | 1.046061 |
| C | -4.477922 | 4.047612 | -3.128187 |
| H | -5.397265 | 3.690289 | -3.591013 |
| C | -6.136002 | -1.041360 | 3.086211 |
| H | -6.701526 | -0.221960 | 3.528643 |
| C | 1.098682 | 1.716903 | -2.108256 |
| H | 2.104023 | 1.765887 | -2.543031 |
| H | 0.381555 | 1.924208 | -2.913343 |
| C | -4.898470 | -0.746839 | 2.465838 |
| C | 0.583506 | 5.271528 | 2.360370 |
| H | 1.487334 | 5.678436 | 2.813141 |
| C | -4.024394 | -2.721404 | -1.184565 |
| H | -4.479776 | -1.829915 | -0.756409 |
| C | -4.603773 | -3.325343 | -2.277091 |
| H | -5.510776 | -2.905222 | -2.707398 |
| C | 1.984659 | 4.118187 | 0.687965 |
| H | 2.876922 | 4.546588 | 1.143833 |
| C | -2.404071 | 3.542080 | -1.902210 |
| C | -4.392606 | 0.571645 | 2.428229 |
| H | -4.983720 | 1.377906 | 2.861017 |
| C | -3.912915 | 1.719515 | -2.543906 |
| H | -4.842577 | 1.384217 | -3.002358 |
| C | -2.833258 | -3.234005 | -0.608793 |
| C | -1.808209 | 4.989375 | 2.310563 |
| H | -2.784551 | 5.192607 | 2.746127 |
| C | -3.038842 | 0.813841 | -2.003484 |
| H | -3.269666 | -0.252418 | -2.033054 |
| C | -2.889157 | -5.018156 | -2.299394 |
| H | -2.438549 | -5.913388 | -2.727432 |
| C | -1.033334 | -4.898341 | -0.672967 |
| H | -0.607823 | -5.808295 | -1.096642 |
| C | 1.893284 | -1.072440 | -3.795939 |
| H | 1.779277 | -1.667935 | -4.710945 |
| H | 2.678615 | -1.548792 | -3.195281 |
| H | 2.238369 | -0.077600 | -4.107844 |
| C | -5.850242 | -3.376795 | 2.580693 |
| H | -6.214795 | -4.400099 | 2.643365 |
| C | -1.716582 | 4.213209 | 1.177567 |
| H | -2.620891 | 3.815892 | 0.720581 |
| C | -4.649780 | -3.120924 | 1.959887 |
| H | -4.078958 | -3.948191 | 1.545149 |
| C | -0.378402 | -4.220202 | 0.322313 |
| H | 0.581181 | -4.575335 | 0.693709 |
| C | -0.650754 | 5.532530 | 2.904968 |
| H | -0.737316 | 6.154095 | 3.793464 |
| C | -2.958270 | 5.822313 | -2.547428 |
| H | -2.699225 | 6.878732 | -2.572455 |
| C | 0.171844 | -2.438483 | -2.626003 |
| H | -0.826232 | -2.472403 | -2.174870 |
| H | 0.871653 | -2.865384 | -1.899865 |
| H | 0.140911 | -3.097004 | -3.505026 |
| C | 1.621697 | -0.748779 | 4.046845 |
| H | 1.861713 | -0.255915 | 4.997263 |
| H | 2.570213 | -0.906539 | 3.514482 |
| H | 1.195801 | -1.729611 | 4.289599 |
| C | -4.038193 | -4.489009 | -2.837054 |
| H | -4.511593 | -4.963900 | -3.693761 |
| C | -6.609329 | -2.329444 | 3.140709 |
| H | -7.560469 | -2.545156 | 3.622838 |
| C | 1.227355 | 1.534299 | 3.088703 |
| H | 0.631933 | 2.146419 | 2.399656 |
| H | 2.263813 | 1.528498 | 2.732740 |
| H | 1.218938 | 2.047646 | 4.059070 |
| C | -4.162748 | 5.384443 | -3.134992 |
| H | -4.833029 | 6.104342 | -3.599675 |
| C | 0.646763 | 0.131173 | 3.259501 |
| C | 2.090065 | 3.249741 | -0.367883 |
| H | 3.073507 | 3.008180 | -0.771825 |
| C | -0.520371 | -0.475535 | -3.982849 |
| H | -0.330336 | 0.552778 | -4.312027 |
| H | -1.516104 | -0.507776 | -3.528197 |
| H | -0.558633 | -1.099107 | -4.886005 |
| C | -0.672749 | 0.260864 | 4.028522 |

| | | | |
|---|---|---|---|
| H | -1.261830 | -0.663662 | 4.068224 |
| H | -1.311212 | 1.042790 | 3.604186 |
| H | -0.448280 | 0.548326 | 5.063507 |
| Ni | 2.182234 | -0.613284 | 0.256090 |
| H | 4.078109 | 1.379121 | -1.283273 |
| C | 6.235438 | 0.951108 | 2.181808 |
| C | 5.377116 | 0.780057 | 1.105408 |
| C | 5.041825 | -0.501875 | 0.638636 |
| C | 5.600520 | -1.601905 | 1.297655 |
| C | 6.459305 | -1.431264 | 2.381020 |
| C | 6.783295 | -0.156331 | 2.828359 |
| H | 6.480944 | 1.957044 | 2.516669 |
| H | 4.938249 | 1.663605 | 0.638620 |
| H | 5.374397 | -2.613199 | 0.963683 |
| H | 6.882275 | -2.306390 | 2.870567 |
| H | 7.458320 | -0.022683 | 3.670620 |
| C | 4.058684 | -0.658730 | -0.497066 |
| C | 4.292051 | 0.354686 | -1.586372 |
| N | 4.120364 | -2.013304 | -1.063860 |
| H | 4.964256 | -2.263754 | -1.576851 |
| N | 5.745089 | 0.386166 | -2.061499 |
| O | 6.295656 | -0.705216 | -2.219006 |
| O | 6.249597 | 1.476256 | -2.266048 |
| H | 3.719716 | 0.123692 | -2.488740 |
| H | 2.830710 | -0.198207 | 1.501800 |
| C | 3.267995 | -2.938846 | -0.633915 |
| C | 3.450834 | -4.364048 | -1.030919 |
| H | 2.527313 | -4.737322 | -1.487299 |
| H | 4.279198 | -4.511288 | -1.728239 |
| H | 3.631249 | -4.971561 | -0.137322 |
| O | 2.295098 | -2.601214 | 0.094957 |
| H | 2.460817 | 0.737850 | 0.723723 |

**$X_{S2}$**

| | | | |
|---|---|---|---|
| P | 0.417622 | -0.724836 | 1.560795 |
| P | 0.711386 | -0.084832 | -1.562460 |
| C | -1.821457 | 1.241955 | -1.375143 |
| C | -0.202881 | 3.029508 | -0.633168 |
| C | -1.953248 | 4.948855 | -1.975788 |
| H | -1.002271 | 5.278140 | -1.564318 |
| C | 1.031508 | 2.617751 | -1.126357 |
| C | -2.249695 | -2.541484 | 0.632547 |
| C | -2.907095 | -1.390344 | 1.313960 |
| C | 0.421057 | -1.138873 | -3.090196 |
| C | -0.244570 | 3.913878 | 0.494800 |
| C | -2.349954 | -0.113222 | 1.343158 |
| C | -4.168499 | -1.621404 | 1.962937 |
| C | -3.084257 | 0.960760 | 1.905138 |
| H | -2.643161 | 1.957707 | 1.876256 |
| C | -0.982807 | 0.131826 | -0.782721 |
| H | -1.473104 | -0.818669 | -1.034174 |
| C | -1.030718 | -3.040880 | 1.077181 |
| C | -0.265300 | -2.324605 | 2.148078 |
| H | 0.581469 | -2.925935 | 2.499354 |
| H | -0.900481 | -2.108594 | 3.020473 |
| C | -3.543629 | 3.174990 | -2.510110 |
| C | -1.462026 | 2.586665 | -1.298492 |
| C | 0.977534 | 4.436886 | 1.031015 |
| C | -2.360498 | -4.386980 | -1.014640 |
| C | -0.986790 | 0.209409 | 0.767513 |
| H | -0.752077 | 1.251590 | 1.034184 |
| C | -4.385187 | 4.150475 | -3.096469 |
| H | -5.326563 | 3.824117 | -3.537172 |
| C | -6.130284 | -0.747907 | 3.166053 |
| H | -6.660206 | 0.104853 | 3.589280 |
| C | 1.098297 | 1.594744 | -2.217936 |
| H | 2.088311 | 1.574093 | -2.690591 |
| H | 0.364590 | 1.818586 | -3.003205 |
| C | -4.882598 | -0.521077 | 2.537672 |
| C | 0.933955 | 5.281180 | 2.166190 |
| H | 1.872422 | 5.672734 | 2.557330 |
| C | -4.071215 | -2.641725 | -1.072422 |
| H | -4.502385 | -1.727019 | -0.668639 |
| C | -4.653409 | -3.248762 | -2.161978 |
| H | -5.538547 | -2.807541 | -2.616258 |
| C | 2.204913 | 4.061396 | 0.437180 |
| H | 3.132587 | 4.481171 | 0.825352 |
| C | -2.304972 | 3.575582 | -1.910719 |
| C | -4.317954 | 0.772038 | 2.468289 |
| H | -4.870846 | 1.613874 | 2.883550 |
| C | -3.892408 | 1.806169 | -2.517011 |
| H | -4.838418 | 1.504030 | -2.964965 |
| C | -2.907989 | -3.183006 | -0.466787 |
| C | -1.464044 | 5.067632 | 2.257895 |
| H | -2.408099 | 5.311458 | 2.741445 |
| C | -3.046329 | 0.870539 | -1.982405 |
| H | -3.312587 | -0.187811 | -2.007625 |
| C | -2.999423 | -5.000907 | -2.118357 |

| | | | |
|---|---|---|---|
| H | -2.574807 | -5.921486 | -2.518209 |
| C | -1.168979 | -4.911786 | -0.462043 |
| H | -0.769055 | -5.844480 | -0.859575 |
| C | 1.784975 | -1.447421 | -3.711317 |
| H | 1.646165 | -2.068927 | -4.605340 |
| H | 2.441857 | -2.000567 | -3.027281 |
| H | 2.307094 | -0.537976 | -4.037880 |
| C | -5.945405 | -3.105290 | 2.716093 |
| H | -6.353385 | -4.110126 | 2.804127 |
| C | -1.458737 | 4.265174 | 1.139608 |
| H | -2.398454 | 3.889563 | 0.739124 |
| C | -4.736703 | -2.915513 | 2.087992 |
| H | -4.201088 | -3.775561 | 1.693547 |
| C | -0.509461 | -4.239122 | 0.534073 |
| H | 0.430331 | -4.622726 | 0.926977 |
| C | -0.259019 | 5.585228 | 2.776104 |
| H | -0.277495 | 6.224455 | 3.655932 |
| C | -2.787854 | 5.870366 | -2.563670 |
| H | -2.489830 | 6.916002 | -2.602861 |
| C | -0.251708 | -2.439723 | -2.656626 |
| H | -1.314560 | -2.293962 | -2.431367 |
| H | 0.225799 | -2.894063 | -1.779903 |
| H | -0.207759 | -3.166482 | -3.479412 |
| C | 1.713283 | -0.686746 | 4.007985 |
| H | 2.038155 | -0.114483 | 4.885817 |
| H | 2.622453 | -1.024394 | 3.490506 |
| H | 1.171865 | -1.566479 | 4.376427 |
| C | -4.119456 | -4.443357 | -2.687591 |
| H | -4.595387 | -4.920090 | -3.541871 |
| C | -6.657805 | -2.013144 | 3.251613 |
| H | -7.616641 | -2.176387 | 3.738933 |
| C | 1.622805 | 1.469479 | 2.745725 |
| H | 1.108628 | 2.041708 | 1.963869 |
| H | 2.634066 | 1.238746 | 2.387337 |
| H | 1.713919 | 2.124664 | 3.622117 |
| C | -4.021272 | 5.474493 | -3.120004 |
| H | -4.674625 | 6.215633 | -3.575242 |
| C | 0.846170 | 0.210388 | 3.123756 |
| C | 2.227625 | 3.157169 | -0.593471 |
| H | 3.178699 | 2.885397 | -1.052955 |
| C | -0.448693 | -0.438314 | -4.134991 |
| H | 0.049143 | 0.427488 | -4.586303 |
| H | -1.412970 | -0.108625 | -3.731292 |
| H | -0.660595 | -1.145496 | -4.947881 |
| C | -0.422882 | 0.620654 | 3.876428 |
| H | -1.131417 | -0.203335 | 4.028942 |
| H | -0.955494 | 1.427774 | 3.360137 |
| H | -0.142242 | 1.001593 | 4.866465 |
| Ni | 2.060063 | -0.845723 | 0.111122 |
| H | 3.996428 | 1.100566 | -1.381988 |
| C | 5.772522 | 1.147438 | 2.277875 |
| C | 5.005332 | 0.810998 | 1.169707 |
| C | 4.879192 | -0.521763 | 0.751769 |
| C | 5.553092 | -1.503346 | 1.484326 |
| C | 6.322379 | -1.167649 | 2.596045 |
| C | 6.438612 | 0.158065 | 2.999045 |
| H | 5.846547 | 2.190388 | 2.581182 |
| H | 4.461565 | 1.598453 | 0.643155 |
| H | 5.474103 | -2.548732 | 1.190064 |
| H | 6.838697 | -1.952804 | 3.144930 |
| H | 7.040233 | 0.420306 | 3.866196 |
| C | 4.004199 | -0.856785 | -0.430363 |
| C | 4.270896 | 0.067038 | -1.591391 |
| N | 4.184316 | -2.250652 | -0.863701 |
| H | 5.095268 | -2.519429 | -1.230089 |
| N | 5.746943 | 0.133933 | -1.988118 |
| O | 6.391622 | -0.912899 | -1.914740 |
| O | 6.173726 | 1.210440 | -2.369218 |
| H | 3.764059 | -0.257986 | -2.505486 |
| H | 2.598775 | -1.102322 | 1.452151 |
| C | 3.200087 | -3.131430 | -0.738314 |
| C | 3.402275 | -4.551262 | -1.144300 |
| H | 2.580896 | -4.856723 | -1.801578 |
| H | 4.354667 | -4.720705 | -1.652280 |
| H | 3.357725 | -5.194644 | -0.258985 |
| O | 2.076868 | -2.770384 | -0.284619 |
| H | 2.193643 | 0.585087 | 0.296626 |

**TSVI$_{S2}$**

| | | | |
|---|---|---|---|
| P | -0.425632 | 0.784071 | 1.560094 |
| P | -0.615544 | 0.128016 | -1.652778 |
| C | 1.856157 | -1.263619 | -1.324177 |
| C | 0.143859 | -3.000969 | -0.670705 |
| C | 1.895334 | -4.972916 | -1.925992 |
| H | 0.916306 | -5.271410 | -1.559301 |
| C | -1.051419 | -2.562354 | -1.233572 |
| C | 2.327222 | 2.501584 | 0.695636 |
| C | 2.943233 | 1.322128 | 1.366802 |

| | | | | | | | |
|---|---|---|---|---|---|---|---|
| C | -0.238835 | 1.162312 | -3.177341 | C | 3.109799 | 4.970538 | -2.036468 |
| C | 0.102724 | -3.886151 | 0.455966 | H | 2.693392 | 5.896318 | -2.433092 |
| C | 2.328535 | 0.071037 | 1.389656 | C | 1.269979 | 4.886591 | -0.391948 |
| C | 4.202413 | 1.501149 | 2.035340 | H | 0.875194 | 5.820182 | -0.792603 |
| C | 3.003922 | -1.029306 | 1.973270 | C | -1.585269 | 1.506277 | -3.819681 |
| H | 2.519460 | -2.005893 | 1.942878 | H | -1.420236 | 2.094562 | -4.731760 |
| C | 1.023877 | -0.127336 | -0.774939 | H | -2.213125 | 2.111187 | -3.150722 |
| H | 1.551913 | 0.807805 | -1.009201 | H | -2.150737 | 0.612301 | -4.116516 |
| C | 1.121126 | 3.021645 | 1.151920 | C | 6.024607 | 2.911740 | 2.820044 |
| C | 0.354500 | 2.310882 | 2.224004 | H | 6.472171 | 3.899018 | 2.914359 |
| H | -0.445942 | 2.945181 | 2.622579 | C | 1.272712 | -4.273774 | 1.158932 |
| H | 1.008596 | 2.029954 | 3.062591 | H | 2.241040 | -3.926656 | 0.803201 |
| C | 3.564048 | -3.251351 | -2.384793 | C | 4.822034 | 2.770866 | 2.168141 |
| C | 1.448870 | -2.595943 | -1.269419 | H | 4.329561 | 3.650616 | 1.760496 |
| C | -1.159006 | -4.375325 | 0.927720 | C | 0.608628 | 4.222549 | 0.608723 |
| C | 2.460043 | 4.354441 | -0.940354 | H | -0.323274 | 4.618638 | 1.009288 |
| C | 0.964653 | -0.193664 | 0.785585 | C | -0.044785 | -5.559122 | 2.730572 |
| H | 0.689482 | -1.228223 | 1.041979 | H | -0.087785 | -6.199540 | 3.608715 |
| C | 4.400529 | -4.253900 | -2.931397 | C | 2.726811 | -5.921644 | -2.473655 |
| H | 5.371213 | -3.958112 | -3.328117 | H | 2.398008 | -6.957497 | -2.526552 |
| C | 6.101284 | 0.550255 | 3.279734 | C | 0.455185 | 2.447637 | -2.734006 |
| H | 6.585870 | -0.322557 | 3.716441 | H | 1.499516 | 2.271392 | -2.451493 |
| C | -1.026162 | -1.540457 | -2.326828 | H | -0.051962 | 2.933716 | -1.890701 |
| H | -1.994033 | -1.479978 | -2.841912 | H | 0.476112 | 3.160574 | -3.569646 |
| H | -0.270351 | -1.799343 | -3.079224 | C | -1.844598 | 0.743659 | 3.913846 |
| C | 4.857147 | 0.373848 | 2.628093 | H | -2.271821 | 0.173775 | 4.748479 |
| C | -1.196846 | -5.220325 | 2.062714 | H | -2.686846 | 1.140101 | 3.328461 |
| H | -2.164349 | -5.586098 | 2.405437 | H | -1.293681 | 1.587756 | 4.345829 |
| C | 4.159375 | 2.597986 | -0.999034 | C | 4.230639 | 4.410424 | -2.601502 |
| H | 4.583735 | 1.679418 | -0.597051 | H | 4.715760 | 4.890172 | -3.448903 |
| C | 4.753839 | 3.209366 | -2.079621 | C | 6.679481 | 1.792583 | 3.372925 |
| H | 5.640446 | 2.766524 | -2.529469 | H | 7.634164 | 1.917533 | 3.879462 |
| C | -2.342329 | -3.973183 | 0.265973 | C | -1.744039 | -1.400527 | 2.639267 |
| H | -3.298583 | -4.375066 | 0.600298 | H | -1.194508 | -1.998877 | 1.902375 |
| C | 2.287119 | -3.611491 | -1.843537 | H | -2.712410 | -1.128708 | 2.199194 |
| C | 4.234803 | -0.892525 | 2.557926 | H | -1.938215 | -2.048520 | 3.504516 |
| H | 4.740579 | -1.755121 | 2.990524 | C | 3.996670 | -5.565891 | -2.971814 |
| C | 3.955851 | -1.894152 | -2.375568 | H | 4.647024 | -6.327647 | -3.396381 |
| H | 4.930134 | -1.622813 | -2.780700 | C | -0.948946 | -0.174861 | 3.078860 |
| C | 2.995971 | 3.142757 | -0.396565 | C | -2.288063 | -3.075764 | -0.769856 |
| C | 1.199265 | -5.077609 | 2.273298 | H | -3.205311 | -2.797662 | -1.291794 |
| H | 2.110306 | -5.351059 | 2.802051 | C | 0.635897 | 0.428751 | -4.192983 |
| C | 3.117471 | -0.932129 | -1.877241 | H | 0.123530 | -0.423595 | -4.653475 |
| H | 3.417013 | 0.117343 | -1.888962 | H | 1.573639 | 0.068147 | -3.753867 |

| | | | |
|---|---|---|---|
| H | 0.899608 | 1.120672 | -5.004057 |
| C | 0.249540 | -0.640226 | 3.907921 |
| H | 0.961225 | 0.160879 | 4.142220 |
| H | 0.801914 | -1.443045 | 3.405283 |
| H | -0.109538 | -1.046349 | 4.862515 |
| Ni | -1.930661 | 0.945865 | -0.030559 |
| H | -3.718074 | -0.902841 | -1.272748 |
| C | -6.053231 | -1.172851 | 2.098818 |
| C | -5.177092 | -0.760888 | 1.101262 |
| C | -5.039528 | 0.597196 | 0.787233 |
| C | -5.788615 | 1.529992 | 1.507270 |
| C | -6.668775 | 1.115113 | 2.503141 |
| C | -6.807261 | -0.236332 | 2.801438 |
| H | -6.146189 | -2.233102 | 2.325749 |
| H | -4.578436 | -1.507778 | 0.576278 |
| H | -5.684226 | 2.593086 | 1.295890 |
| H | -7.249888 | 1.857268 | 3.046620 |
| H | -7.496146 | -0.559379 | 3.578552 |
| C | -4.034187 | 1.016943 | -0.269705 |
| C | -4.100856 | 0.095921 | -1.472486 |
| N | -4.209186 | 2.408043 | -0.675400 |
| H | -5.129923 | 2.681727 | -1.009276 |
| N | -5.538942 | -0.112905 | -1.961834 |
| O | -6.276868 | 0.870817 | -1.930896 |
| O | -5.834909 | -1.226052 | -2.364184 |
| H | -3.577094 | 0.514867 | -2.336740 |
| H | -3.064921 | 1.102879 | 0.883469 |
| C | -3.171469 | 3.246185 | -0.746416 |
| C | -3.387291 | 4.650988 | -1.194295 |
| H | -2.721815 | 4.866187 | -2.037182 |
| H | -4.418946 | 4.857190 | -1.489164 |
| H | -3.106808 | 5.335863 | -0.387097 |
| O | -2.012895 | 2.863528 | -0.435015 |
| H | -1.890617 | -0.484349 | 0.202520 |

**XI$_{S2}$**

| | | | |
|---|---|---|---|
| P | 0.543242 | -0.781013 | 1.550920 |
| P | 0.513251 | 0.040653 | -1.759380 |
| C | -2.077380 | 1.090433 | -1.234740 |
| C | -0.574311 | 3.011027 | -0.586559 |
| C | -2.595874 | 4.777007 | -1.743944 |
| H | -1.653105 | 5.186221 | -1.388932 |
| C | 0.641394 | 2.737004 | -1.205820 |
| C | -2.092055 | -2.689201 | 0.725971 |

| | | | |
|---|---|---|---|
| C | -2.796608 | -1.580397 | 1.428333 |
| C | 0.187045 | -0.955072 | -3.316738 |
| C | -0.594055 | 3.857672 | 0.569544 |
| C | -2.275479 | -0.287037 | 1.451243 |
| C | -4.029375 | -1.858636 | 2.109290 |
| C | -3.016642 | 0.752015 | 2.065387 |
| H | -2.605825 | 1.761134 | 2.039931 |
| C | -1.085771 | 0.053183 | -0.758107 |
| H | -1.506682 | -0.933007 | -0.995850 |
| C | -0.829732 | -3.091330 | 1.145507 |
| C | -0.137388 | -2.345811 | 2.244733 |
| H | 0.695863 | -2.926157 | 2.657051 |
| H | -0.829198 | -2.109177 | 3.066253 |
| C | -4.050580 | 2.871512 | -2.201698 |
| C | -1.840415 | 2.462482 | -1.154151 |
| C | 0.623558 | 4.457893 | 1.029166 |
| C | -2.043847 | -4.460303 | -1.002594 |
| C | -0.956017 | 0.085840 | 0.808308 |
| H | -0.750635 | 1.136373 | 1.068861 |
| C | -5.015517 | 3.772247 | -2.712537 |
| H | -5.950933 | 3.366324 | -3.096209 |
| C | -5.981667 | -1.060865 | 3.377240 |
| H | -6.522599 | -0.231137 | 3.831238 |
| C | 0.691343 | 1.777503 | -2.352048 |
| H | 1.639503 | 1.860123 | -2.899863 |
| H | -0.119489 | 1.984349 | -3.062739 |
| C | -4.756493 | -0.788565 | 2.722762 |
| C | 0.605566 | 5.257135 | 2.197319 |
| H | 1.540911 | 5.702651 | 2.535325 |
| C | -3.948467 | -2.929072 | -0.927002 |
| H | -4.469432 | -2.091373 | -0.467703 |
| C | -4.498730 | -3.558123 | -2.020056 |
| H | -5.447791 | -3.208869 | -2.422072 |
| C | 1.822222 | 4.213751 | 0.318119 |
| H | 2.739566 | 4.701554 | 0.646653 |
| C | -2.815228 | 3.376380 | -1.681440 |
| C | -4.224656 | 0.518811 | 2.667073 |
| H | -4.783225 | 1.336757 | 3.121019 |
| C | -4.269213 | 1.475905 | -2.216790 |
| H | -5.211470 | 1.092581 | -2.606703 |
| C | -2.708368 | -3.352749 | -0.383004 |
| C | -1.761694 | 4.881807 | 2.444237 |
| H | -2.684817 | 5.053319 | 2.994587 |
| C | -3.302282 | 0.617043 | -1.765195 |

| | | | |
|---|---|---|---|
| H | -3.466538 | -0.460925 | -1.800845 |
| C | -2.648326 | -5.099213 | -2.112134 |
| H | -2.133265 | -5.944388 | -2.568715 |
| C | -0.776428 | -4.858613 | -0.516637 |
| H | -0.272442 | -5.702334 | -0.988176 |
| C | 1.557995 | -1.147098 | -3.973464 |
| H | 1.448329 | -1.704281 | -4.913176 |
| H | 2.236966 | -1.717448 | -3.325552 |
| H | 2.041205 | -0.192017 | -4.222179 |
| C | -5.747350 | -3.403764 | 2.874549 |
| H | -6.128173 | -4.420284 | 2.950719 |
| C | -1.780527 | 4.110307 | 1.304858 |
| H | -2.718695 | 3.685719 | 0.953457 |
| C | -4.558859 | -3.170032 | 2.223316 |
| H | -4.010580 | -4.006196 | 1.795661 |
| C | -0.178437 | -4.176530 | 0.512432 |
| H | 0.810260 | -4.469902 | 0.865101 |
| C | -0.558411 | 5.459589 | 2.899095 |
| H | -0.556415 | 6.069451 | 3.799786 |
| C | -3.548711 | 5.625229 | -2.258414 |
| H | -3.349992 | 6.694350 | -2.298900 |
| C | -0.382886 | -2.312644 | -2.913349 |
| H | -1.435162 | -2.244407 | -2.611333 |
| H | 0.177145 | -2.777964 | -2.090225 |
| H | -0.346593 | -2.998489 | -3.770963 |
| C | 1.973158 | -0.624407 | 3.877911 |
| H | 2.419270 | -0.019540 | 4.677417 |
| H | 2.798917 | -1.041998 | 3.284096 |
| H | 1.440274 | -1.454427 | 4.356543 |
| C | -3.848252 | -4.657500 | -2.617164 |
| H | -4.297651 | -5.153082 | -3.475177 |
| C | -6.474104 | -2.340960 | 3.449177 |
| H | -7.416120 | -2.539163 | 3.956284 |
| C | 1.802994 | 1.471202 | 2.525653 |
| H | 1.211463 | 2.045354 | 1.802024 |
| H | 2.750152 | 1.199001 | 2.036864 |
| H | 2.038321 | 2.141404 | 3.363750 |
| C | -4.775658 | 5.124196 | -2.738465 |
| H | -5.521969 | 5.807437 | -3.137968 |
| C | 1.043512 | 0.247719 | 3.030595 |
| C | 1.831050 | 3.360038 | -0.755424 |
| H | 2.753658 | 3.190245 | -1.312824 |
| C | -0.760299 | -0.259923 | -4.293457 |
| H | -0.319313 | 0.641823 | -4.733721 |
| H | -1.713082 | 0.018177 | -3.826471 |
| H | -0.988521 | -0.943002 | -5.122647 |
| C | -0.150187 | 0.711772 | 3.864303 |
| H | -0.829119 | -0.105469 | 4.139104 |
| H | -0.738274 | 1.475094 | 3.339931 |
| H | 0.209207 | 1.167164 | 4.796729 |
| Ni | 1.732484 | -0.782150 | -0.218021 |
| H | 4.004802 | 1.481417 | -0.678877 |
| C | 7.106256 | 0.923672 | 2.071482 |
| C | 6.167719 | 0.793565 | 1.051837 |
| C | 5.539513 | -0.432980 | 0.821358 |
| C | 5.855743 | -1.519887 | 1.643490 |
| C | 6.791840 | -1.388678 | 2.662292 |
| C | 7.422980 | -0.165486 | 2.876865 |
| H | 7.594694 | 1.882569 | 2.229395 |
| H | 5.942864 | 1.664460 | 0.437572 |
| H | 5.360470 | -2.477713 | 1.481377 |
| H | 7.030318 | -2.244939 | 3.289451 |
| H | 8.159893 | -0.061841 | 3.670015 |
| C | 4.465962 | -0.597760 | -0.230082 |
| C | 4.317801 | 0.566816 | -1.190283 |
| N | 4.578894 | -1.876116 | -0.923140 |
| H | 5.498689 | -2.093453 | -1.289276 |
| N | 5.581864 | 0.890311 | -1.943409 |
| O | 6.386009 | -0.025194 | -2.107898 |
| O | 5.679802 | 2.027610 | -2.379065 |
| H | 3.576471 | 0.344510 | -1.968203 |
| H | 3.503012 | -0.647792 | 0.335248 |
| C | 3.574463 | -2.755530 | -1.087642 |
| C | 3.895968 | -4.079171 | -1.698018 |
| H | 3.166854 | -4.301722 | -2.483317 |
| H | 4.905500 | -4.132345 | -2.113349 |
| H | 3.791533 | -4.860632 | -0.937020 |
| O | 2.394946 | -2.518625 | -0.735886 |
| H | 1.411842 | 0.601331 | 0.088721 |

**TSIV$_{S3}$**

| | | | |
|---|---|---|---|
| P | 0.416588 | -0.014514 | -1.505792 |
| P | 0.075066 | -0.803305 | 1.487424 |
| C | -2.664241 | -0.138480 | 1.350454 |
| C | -2.620497 | -2.470344 | 0.388489 |
| C | -5.204521 | -2.882344 | 1.698696 |
| H | -4.712182 | -3.708682 | 1.192099 |
| C | -1.410695 | -2.993897 | 0.837254 |

| | | | |
|---|---|---|---|
| C | -0.486914 | 3.072125 | -0.409441 |
| C | -1.761709 | 2.692914 | -1.080931 |
| C | 0.623084 | 0.035561 | 3.076733 |
| C | -3.207974 | -3.005580 | -0.804476 |
| C | -2.117753 | 1.356730 | -1.280685 |
| C | -2.616934 | 3.729021 | -1.589798 |
| C | -3.350263 | 1.039959 | -1.901117 |
| H | -3.611287 | -0.011231 | -2.026335 |
| C | -1.303401 | 0.200705 | 0.783528 |
| H | -1.056292 | 1.225319 | 1.090138 |
| C | 0.726449 | 2.636921 | -0.933240 |
| C | 0.741509 | 1.695096 | -2.090436 |
| H | 1.678905 | 1.728459 | -2.643060 |
| H | -0.074262 | 1.918470 | -2.792255 |
| C | -5.217731 | -0.567511 | 2.477828 |
| C | -3.283035 | -1.375248 | 1.155437 |
| C | -2.571970 | -4.102180 | -1.473429 |
| C | 0.768602 | 4.370053 | 1.283226 |
| C | -1.296867 | 0.193533 | -0.760486 |
| H | -1.803269 | -0.729485 | -1.061861 |
| C | -6.487217 | -0.804432 | 3.057427 |
| H | -6.973127 | 0.010377 | 3.592741 |
| C | -4.728429 | 4.402520 | -2.664293 |
| H | -5.680643 | 4.114304 | -3.108409 |
| C | -0.711822 | -2.373795 | 2.004826 |
| H | 0.062411 | -3.025371 | 2.422076 |
| H | -1.422191 | -2.125610 | 2.803269 |
| C | -3.869164 | 3.380816 | -2.193542 |
| C | -3.126202 | -4.600389 | -2.677040 |
| H | -2.629094 | -5.435046 | -3.170277 |
| C | -1.668816 | 4.251673 | 1.449496 |
| H | -2.625404 | 3.900608 | 1.065909 |
| C | -1.627506 | 5.015560 | 2.594375 |
| H | -2.552043 | 5.259096 | 3.114428 |
| C | -1.385476 | -4.648889 | -0.933346 |
| H | -0.918656 | -5.497304 | -1.432460 |
| C | -4.562253 | -1.618528 | 1.757651 |
| C | -4.211931 | 2.017319 | -2.324542 |
| H | -5.165873 | 1.753434 | -2.779931 |
| C | -4.571277 | 0.681117 | 2.613722 |
| H | -5.073619 | 1.479756 | 3.157895 |
| C | -0.480653 | 3.905754 | 0.756054 |
| C | -4.885692 | -2.958287 | -2.568054 |
| H | -5.782019 | -2.516504 | -2.999099 |
| C | -3.324435 | 0.879823 | 2.079996 |
| H | -2.821247 | 1.841333 | 2.200905 |
| C | 0.771843 | 5.179411 | 2.444718 |
| H | 1.728821 | 5.534268 | 2.826544 |
| C | 1.974951 | 3.959327 | 0.666747 |
| H | 2.922489 | 4.323960 | 1.063518 |
| C | 1.958063 | -0.547451 | 3.538466 |
| H | 2.175089 | -0.182510 | 4.550019 |
| H | 2.778596 | -0.204182 | 2.897041 |
| H | 1.972300 | -1.642913 | 3.566092 |
| C | -3.116880 | 6.071795 | -2.025809 |
| H | -2.817847 | 7.117039 | -1.984782 |
| C | -4.380145 | -2.459011 | -1.388728 |
| H | -4.882006 | -1.628810 | -0.894998 |
| C | -2.265623 | 5.103590 | -1.547745 |
| H | -1.301702 | 5.398306 | -1.141254 |
| C | 1.956293 | 3.080040 | -0.386559 |
| H | 2.887124 | 2.734369 | -0.837654 |
| C | -4.257203 | -4.039533 | -3.219741 |
| H | -4.669328 | -4.425962 | -4.149220 |
| C | -6.433625 | -3.083548 | 2.282111 |
| H | -6.901758 | -4.064052 | 2.224300 |
| C | 0.802984 | 1.524814 | 2.801103 |
| H | -0.151341 | 2.059191 | 2.725153 |
| H | 1.373751 | 1.711424 | 1.880445 |
| H | 1.358247 | 1.985542 | 3.628469 |
| C | 0.766001 | -2.447359 | -2.837760 |
| H | 0.503940 | -3.105819 | -3.677391 |
| H | 0.351846 | -2.908330 | -1.930391 |
| H | 1.859849 | -2.446771 | -2.769190 |
| C | -0.398639 | 5.494323 | 3.092963 |
| H | -0.380283 | 6.107497 | 3.991218 |
| C | -4.367566 | 5.724312 | -2.576306 |
| H | -5.034489 | 6.501385 | -2.943531 |
| C | -1.273565 | -1.162996 | -3.487565 |
| H | -1.325810 | -1.688675 | -4.450259 |
| H | -1.752075 | -0.185421 | -3.622755 |
| H | -1.869147 | -1.749446 | -2.778341 |
| C | -7.089803 | -2.034762 | 2.958450 |
| H | -8.064602 | -2.206007 | 3.409771 |
| C | 0.200040 | -1.050040 | -3.091187 |
| C | -0.810565 | -4.091840 | 0.179775 |
| H | 0.109346 | -4.510066 | 0.587901 |
| C | -0.431099 | -0.171422 | 4.168718 |

| | | | |
|---|---|---|---|
| H | -0.458421 | -1.205088 | 4.530402 |
| H | -1.441092 | 0.112884 | 3.847820 |
| H | -0.169176 | 0.463792 | 5.024761 |
| C | 0.951418 | -0.384008 | -4.248172 |
| H | 2.005918 | -0.188050 | -4.036305 |
| H | 0.479579 | 0.556851 | -4.552891 |
| H | 0.916764 | -1.058886 | -5.112774 |
| Ni | 1.634540 | -1.115890 | 0.013319 |
| H | 3.324276 | 0.298413 | 0.607114 |
| C | 4.213743 | -0.263376 | 0.248746 |
| C | 3.814734 | -1.173497 | -0.928576 |
| H | 4.620386 | -1.906251 | -1.072249 |
| N | 3.723084 | -0.528195 | -2.239052 |
| O | 3.939740 | -1.225817 | -3.229576 |
| O | 3.420656 | 0.669517 | -2.282477 |
| N | 4.589663 | -1.124337 | 1.376563 |
| H | 3.797555 | -1.513665 | 1.876305 |
| C | 5.627430 | -2.044812 | 1.449158 |
| C | 6.909560 | -1.761634 | 0.721541 |
| H | 7.626602 | -2.545092 | 0.972007 |
| H | 6.772854 | -1.748946 | -0.366681 |
| H | 7.317910 | -0.782855 | 0.995309 |
| O | 5.476407 | -3.032621 | 2.157630 |
| C | 5.267795 | 0.794840 | -0.020709 |
| C | 6.134411 | 0.775919 | -1.113562 |
| C | 5.386778 | 1.827007 | 0.914679 |
| C | 7.104947 | 1.761710 | -1.264659 |
| H | 6.076752 | -0.017144 | -1.859421 |
| C | 6.354992 | 2.813622 | 0.767331 |
| H | 4.714522 | 1.841174 | 1.775578 |
| C | 7.218428 | 2.782612 | -0.326123 |
| H | 7.772614 | 1.729810 | -2.122808 |
| H | 6.438185 | 3.606701 | 1.508069 |
| H | 7.976387 | 3.553444 | -0.446434 |
| C | 2.587475 | -3.598791 | 0.634405 |
| O | 2.133613 | -2.544227 | 1.208914 |
| O | 2.908708 | -3.595884 | -0.591746 |
| C | 2.797041 | -4.816160 | 1.460537 |
| H | 3.765966 | -4.699067 | 1.964653 |
| H | 2.031621 | -4.909770 | 2.235796 |
| H | 2.831398 | -5.711943 | 0.838060 |
| H | 3.053236 | -2.352344 | -0.844253 |

**TSIV$_{S1}$**

| | | | |
|---|---|---|---|
| P | 0.382783 | -0.293229 | -1.595787 |
| P | 0.119199 | -1.161534 | 1.329846 |
| C | -2.465998 | 0.019841 | 1.431806 |
| C | -2.904569 | -2.273397 | 0.468468 |
| C | -5.370097 | -2.282289 | 2.033367 |
| H | -5.061968 | -3.175716 | 1.495211 |
| C | -1.791338 | -3.043812 | 0.789842 |
| C | 0.256524 | 2.826797 | -0.408133 |
| C | -1.076414 | 2.737974 | -1.071052 |
| C | 0.913745 | -0.504686 | 2.888312 |
| C | -3.698005 | -2.638039 | -0.666896 |
| C | -1.762981 | 1.533458 | -1.218467 |
| C | -1.664294 | 3.945720 | -1.580626 |
| C | -3.068284 | 1.524707 | -1.768431 |
| H | -3.597640 | 0.572816 | -1.831091 |
| C | -1.104434 | 0.118953 | 0.782800 |
| H | -0.640321 | 1.055643 | 1.126756 |
| C | 1.371149 | 2.196829 | -0.952561 |
| C | 1.224734 | 1.253083 | -2.103549 |
| H | 2.200026 | 0.972502 | -2.520436 |
| H | 0.623630 | 1.683426 | -2.919489 |
| C | -4.953834 | 0.009285 | 2.768531 |
| C | -3.286588 | -1.100006 | 1.306948 |
| C | -3.395011 | -3.841609 | -1.382846 |
| C | 1.728609 | 3.834157 | 1.311112 |
| C | -1.197805 | 0.208879 | -0.759396 |
| H | -1.892889 | -0.583224 | -1.077868 |
| C | -6.183760 | -0.025178 | 3.466889 |
| H | -6.486087 | 0.857988 | 4.028380 |
| C | -3.567429 | 5.101690 | -2.630489 |
| H | -4.574235 | 5.052815 | -3.043876 |
| C | -0.868000 | -2.604517 | 1.882635 |
| H | -0.173874 | -3.403384 | 2.169021 |
| H | -1.420183 | -2.302745 | 2.784903 |
| C | -2.982931 | 3.909840 | -2.139722 |
| C | -4.158851 | -4.178028 | -2.526281 |
| H | -3.916079 | -5.095797 | -3.061182 |
| C | -0.690201 | 4.161866 | 1.481202 |
| H | -1.693732 | 4.012142 | 1.086466 |
| C | -0.511023 | 4.874330 | 2.644587 |
| H | -1.375345 | 5.276744 | 3.169394 |
| C | -2.308436 | -4.642246 | -0.957737 |
| H | -2.089929 | -5.564618 | -1.494625 |
| C | -4.534481 | -1.135814 | 2.016503 |

| | | | |
|---|---|---|---|
| C | -3.670329 | 2.676550 | -2.198772 |
| H | -4.681251 | 2.653614 | -2.603841 |
| C | -4.121619 | 1.150583 | 2.804525 |
| H | -4.451670 | 2.028593 | 3.358290 |
| C | 0.415481 | 3.614395 | 0.779528 |
| C | -5.474139 | -2.174990 | -2.264686 |
| H | -6.279721 | -1.531915 | -2.613747 |
| C | -2.909655 | 1.145015 | 2.168296 |
| H | -2.264587 | 2.023792 | 2.210754 |
| C | 1.877882 | 4.589457 | 2.499232 |
| H | 2.882800 | 4.750195 | 2.888779 |
| C | 2.841404 | 3.254798 | 0.657172 |
| H | 3.842420 | 3.438183 | 1.047714 |
| C | 1.599383 | -1.674537 | 3.596487 |
| H | 2.254065 | -1.296957 | 4.392159 |
| H | 2.216358 | -2.276245 | 2.914465 |
| H | 0.878946 | -2.354219 | 4.065479 |
| C | -1.569490 | 6.326296 | -2.079377 |
| H | -1.016785 | 7.263524 | -2.075568 |
| C | -4.760758 | -1.825968 | -1.140697 |
| H | -5.009940 | -0.911091 | -0.605795 |
| C | -0.978435 | 5.188181 | -1.583123 |
| H | 0.037510 | 5.239677 | -1.199363 |
| C | 2.661696 | 2.429257 | -0.423036 |
| H | 3.519424 | 1.961752 | -0.906756 |
| C | -5.174059 | -3.361991 | -2.964743 |
| H | -5.747664 | -3.629064 | -3.849473 |
| C | -6.554908 | -2.288293 | 2.731819 |
| H | -7.172834 | -3.183668 | 2.733930 |
| C | 1.921524 | 0.569065 | 2.493133 |
| H | 1.462414 | 1.329299 | 1.851498 |
| H | 2.788140 | 0.164873 | 1.955793 |
| H | 2.298536 | 1.082012 | 3.388049 |
| C | -0.429689 | -2.588726 | -2.866508 |
| H | -0.832939 | -3.094032 | -3.754581 |
| H | -1.200176 | -2.636387 | -2.088829 |
| H | 0.446273 | -3.149176 | -2.520722 |
| C | 0.783699 | 5.096125 | 3.158127 |
| H | 0.912077 | 5.667498 | 4.074918 |
| C | -2.879675 | 6.290057 | -2.598339 |
| H | -3.336571 | 7.199938 | -2.981589 |
| C | -1.319716 | -0.466114 | -3.813210 |
| H | -1.482425 | -0.894927 | -4.810348 |
| H | -1.209709 | 0.618432 | -3.937431 |
| H | -2.231111 | -0.654489 | -3.232636 |
| C | -6.973435 | -1.149217 | 3.449703 |
| H | -7.914648 | -1.167000 | 3.994965 |
| C | -0.088587 | -1.140714 | -3.199097 |
| C | -1.504912 | -4.231297 | 0.075682 |
| H | -0.642046 | -4.828279 | 0.372132 |
| C | -0.132456 | 0.123519 | 3.811696 |
| H | -0.979911 | -0.538383 | 4.028461 |
| H | -0.529627 | 1.058685 | 3.399600 |
| H | 0.345808 | 0.368664 | 4.768914 |
| C | 1.077538 | -1.057993 | -4.186431 |
| H | 2.001624 | -1.478760 | -3.780458 |
| H | 1.265591 | -0.029118 | -4.516077 |
| H | 0.814809 | -1.637186 | -5.080461 |
| Ni | 1.468313 | -1.607660 | -0.271763 |
| H | 3.346388 | -1.084360 | 0.529049 |
| C | 4.401198 | -1.421588 | 0.412185 |
| N | 4.861419 | -1.621254 | 1.775296 |
| H | 4.155565 | -1.769320 | 2.486934 |
| C | 6.119180 | -2.001966 | 2.226627 |
| O | 6.258516 | -2.313998 | 3.396078 |
| C | 7.266754 | -1.948747 | 1.253361 |
| H | 7.742221 | -0.962173 | 1.307846 |
| H | 8.009335 | -2.690152 | 1.556334 |
| H | 6.988159 | -2.108871 | 0.207166 |
| C | 4.339835 | -2.742225 | -0.321242 |
| H | 5.195241 | -3.410048 | -0.271144 |
| N | 3.675753 | -2.867507 | -1.504546 |
| O | 3.979729 | -3.650439 | -2.399083 |
| O | 2.545685 | -2.171011 | -1.680229 |
| C | 5.121232 | -0.274779 | -0.287507 |
| C | 5.285490 | -0.232006 | -1.674935 |
| C | 5.568261 | 0.809957 | 0.476651 |
| C | 5.875049 | 0.875550 | -2.283125 |
| H | 4.979949 | -1.068317 | -2.300056 |
| C | 6.170525 | 1.906940 | -0.130681 |
| H | 5.449259 | 0.782370 | 1.559171 |
| C | 6.319959 | 1.947810 | -1.515387 |
| H | 6.002023 | 0.887234 | -3.363779 |
| H | 6.526860 | 2.733468 | 0.482208 |
| H | 6.792548 | 2.804116 | -1.991730 |
| H | 2.866580 | -3.125911 | 0.467080 |
| H | 2.061849 | -2.964792 | 0.679365 |

**IX$_{R2}$**

| | | | |
|---|---|---|---|
| P | -0.161101 | -0.832036 | -1.742720 |
| P | -1.034638 | 0.065113 | 1.137699 |
| C | 1.563552 | 1.300036 | 1.392801 |
| C | 0.216942 | 3.077371 | 0.236509 |
| C | 1.669489 | 5.022196 | 1.870963 |
| H | 0.830137 | 5.345786 | 1.259488 |
| C | -1.110503 | 2.723083 | 0.447300 |
| C | 2.304351 | -2.557492 | -0.151886 |
| C | 3.056869 | -1.495042 | -0.881782 |
| C | -1.026783 | -0.840846 | 2.788828 |
| C | 0.562496 | 3.861218 | -0.914879 |
| C | 2.521910 | -0.231410 | -1.127231 |
| C | 4.378788 | -1.797332 | -1.359973 |
| C | 3.315260 | 0.768568 | -1.741391 |
| H | 2.878141 | 1.756807 | -1.893470 |
| C | 0.801012 | 0.177180 | 0.722156 |
| H | 1.185357 | -0.763136 | 1.140368 |
| C | 1.124605 | -3.086657 | -0.668274 |
| C | 0.535743 | -2.525042 | -1.925468 |
| H | -0.261076 | -3.171479 | -2.309442 |
| H | 1.314804 | -2.457577 | -2.696033 |
| C | 3.082618 | 3.247507 | 2.775118 |
| C | 1.272458 | 2.647012 | 1.192757 |
| C | -0.472113 | 4.330863 | -1.787359 |
| C | 2.206802 | -4.228887 | 1.674689 |
| C | 1.096318 | 0.142466 | -0.795972 |
| H | 0.930061 | 1.163982 | -1.161971 |
| C | 3.800080 | 4.231052 | 3.496772 |
| H | 4.625154 | 3.910009 | 4.131877 |
| C | 6.462128 | -1.066310 | -2.451407 |
| H | 7.038990 | -0.267669 | -2.916491 |
| C | -1.488232 | 1.803921 | 1.570309 |
| H | -2.570071 | 1.835844 | 1.760103 |
| H | -0.975108 | 2.082551 | 2.501064 |
| C | 5.158843 | -0.770707 | -1.985530 |
| C | -0.128141 | 5.082420 | -2.936478 |
| H | -0.930064 | 5.437097 | -3.583671 |
| C | 3.949613 | -2.515338 | 1.729217 |
| H | 4.436731 | -1.647117 | 1.289218 |
| C | 4.427642 | -3.038910 | 2.908532 |
| H | 5.284397 | -2.576963 | 3.395178 |
| C | -1.816544 | 4.008149 | -1.492127 |
| H | -2.605999 | 4.392587 | -2.137950 |
| C | 2.000018 | 3.643785 | 1.926014 |
| C | 4.600851 | 0.518134 | -2.140446 |
| H | 5.201094 | 1.302474 | -2.600327 |
| C | 3.404863 | 1.875298 | 2.876947 |
| H | 4.246668 | 1.574178 | 3.499484 |
| C | 2.829199 | -3.087614 | 1.072787 |
| C | 2.209155 | 4.879464 | -2.392401 |
| H | 3.248979 | 5.091258 | -2.633696 |
| C | 2.657062 | 0.936812 | 2.217462 |
| H | 2.899820 | -0.122289 | 2.317939 |
| C | 2.733616 | -4.752088 | 2.880064 |
| H | 2.252385 | -5.624531 | 3.321602 |
| C | 1.064518 | -4.791988 | 1.058130 |
| H | 0.608674 | -5.677364 | 1.502218 |
| C | -2.443374 | -0.803560 | 3.349086 |
| H | -2.470433 | -1.334362 | 4.310134 |
| H | -3.139240 | -1.305442 | 2.670675 |
| H | -2.810873 | 0.214884 | 3.526472 |
| C | 6.209863 | -3.354605 | -1.750487 |
| H | 6.614960 | -4.361930 | -1.677694 |
| C | 1.906612 | 4.161204 | -1.257143 |
| H | 2.709444 | 3.812521 | -0.610440 |
| C | 4.945943 | -3.095767 | -1.274240 |
| H | 4.365160 | -3.904960 | -0.838106 |
| C | 0.522029 | -4.213639 | -0.060890 |
| H | -0.374500 | -4.626823 | -0.521284 |
| C | 1.185397 | 5.350835 | -3.239087 |
| H | 1.437933 | 5.925129 | -4.127753 |
| C | 2.378615 | 5.952648 | 2.594379 |
| H | 2.098292 | 7.002634 | 2.541603 |
| C | -0.630500 | -2.289356 | 2.523079 |
| H | 0.397588 | -2.375038 | 2.154640 |
| H | -1.284861 | -2.770464 | 1.786229 |
| H | -0.683625 | -2.868947 | 3.454932 |
| C | -1.364011 | -0.777616 | -4.238104 |
| H | -1.298783 | -0.534198 | -5.306206 |
| H | -2.327767 | -0.396047 | -3.879963 |
| H | -1.384669 | -1.872461 | -4.153544 |
| C | 3.821075 | -4.171932 | 3.488148 |
| H | 4.212966 | -4.581749 | 4.416504 |
| C | 6.983514 | -2.331541 | -2.335133 |
| H | 7.984707 | -2.549158 | -2.700931 |
| C | -0.308124 | 1.362804 | -3.444999 |
| H | 0.626688 | 1.849677 | -3.137947 |

| | | | |
|---|---|---|---|
| H | -1.096700 | 1.701108 | -2.760223 |
| H | -0.554322 | 1.749218 | -4.442679 |
| C | 3.460106 | 5.559000 | 3.408457 |
| H | 4.015667 | 6.306420 | 3.970876 |
| C | -0.168903 | -0.158096 | -3.502296 |
| C | -2.119031 | 3.210045 | -0.417839 |
| H | -3.153820 | 2.969170 | -0.184803 |
| C | -0.052616 | -0.240626 | 3.800391 |
| H | -0.202569 | 0.832122 | 3.970984 |
| H | 0.991369 | -0.392410 | 3.509175 |
| H | -0.194241 | -0.743389 | 4.766673 |
| C | 1.105429 | -0.512398 | -4.278659 |
| H | 1.153479 | -1.575188 | -4.539744 |
| H | 2.024045 | -0.242814 | -3.745720 |
| H | 1.100535 | 0.044922 | -5.224369 |
| Ni | -2.146230 | -0.726563 | -0.681536 |
| H | -4.308196 | -1.929953 | -2.080229 |
| C | -5.965843 | 0.923985 | 2.631959 |
| C | -5.299716 | -0.090297 | 1.950384 |
| C | -4.789820 | 0.113124 | 0.661574 |
| C | -5.021567 | 1.366266 | 0.069311 |
| C | -5.686764 | 2.380827 | 0.744827 |
| C | -6.155309 | 2.168332 | 2.039023 |
| H | -6.351586 | 0.730605 | 3.630805 |
| H | -5.191539 | -1.057395 | 2.435887 |
| H | -4.665033 | 1.558850 | -0.943308 |
| H | -5.842218 | 3.340644 | 0.255264 |
| H | -6.676759 | 2.959400 | 2.572379 |
| C | -4.089213 | -0.937528 | -0.130983 |
| C | -4.749059 | -1.112551 | -1.496317 |
| N | -4.078715 | -2.251639 | 0.526215 |
| H | -4.960709 | -2.586287 | 0.907700 |
| N | -6.243702 | -1.471256 | -1.420804 |
| O | -6.661700 | -2.021251 | -0.404168 |
| O | -6.906959 | -1.191537 | -2.404947 |
| H | -4.738787 | -0.215942 | -2.116916 |
| H | -2.690295 | 0.129554 | -1.971629 |
| C | -3.210351 | -3.147072 | 0.042619 |
| C | -3.332739 | -4.584754 | 0.414302 |
| H | -2.466091 | -4.873903 | 1.021373 |
| H | -4.242467 | -4.805054 | 0.977969 |
| H | -3.311654 | -5.204842 | -0.487050 |
| O | -2.273189 | -2.749055 | -0.700187 |
| H | -2.504712 | 0.688430 | -1.377043 |

**TSV$_{R2}$**

| | | | |
|---|---|---|---|
| P | -0.183084 | -0.828040 | -1.771935 |
| P | -1.025152 | 0.045697 | 1.159009 |
| C | 1.565212 | 1.258211 | 1.390934 |
| C | 0.255719 | 3.062005 | 0.230463 |
| C | 1.700264 | 4.972275 | 1.917119 |
| H | 0.882045 | 5.313309 | 1.286778 |
| C | -1.076787 | 2.704874 | 0.408362 |
| C | 2.281559 | -2.585145 | -0.246297 |
| C | 3.033713 | -1.506393 | -0.948686 |
| C | -1.002913 | -0.816182 | 2.838434 |
| C | 0.622663 | 3.868914 | -0.898199 |
| C | 2.511313 | -0.230581 | -1.147186 |
| C | 4.361567 | -1.799350 | -1.415346 |
| C | 3.335810 | 0.793610 | -1.674693 |
| H | 2.914960 | 1.794799 | -1.778090 |
| C | 0.797244 | 0.155030 | 0.697260 |
| H | 1.185469 | -0.797763 | 1.078357 |
| C | 1.109106 | -3.116911 | -0.772000 |
| C | 0.492402 | -2.526685 | -2.004400 |
| H | -0.336515 | -3.148875 | -2.361496 |
| H | 1.232101 | -2.463953 | -2.816361 |
| C | 3.065580 | 3.169088 | 2.839088 |
| C | 1.292134 | 2.610517 | 1.199934 |
| C | -0.397075 | 4.359610 | -1.777700 |
| C | 2.184041 | -4.261958 | 1.574746 |
| C | 1.079865 | 0.141488 | -0.824767 |
| H | 0.915175 | 1.164404 | -1.193519 |
| C | 3.776763 | 4.135708 | 3.589595 |
| H | 4.579457 | 3.796676 | 4.243799 |
| C | 6.473359 | -1.041202 | -2.427869 |
| H | 7.069701 | -0.227848 | -2.840121 |
| C | -1.477931 | 1.795727 | 1.532352 |
| H | -2.561637 | 1.836248 | 1.709528 |
| H | -0.971750 | 2.088327 | 2.461506 |
| C | 5.165565 | -0.752328 | -1.971341 |
| C | -0.032631 | 5.135654 | -2.904261 |
| H | -0.823156 | 5.502676 | -3.558482 |
| C | 3.913043 | -2.537043 | 1.646854 |
| H | 4.398502 | -1.664384 | 1.214281 |
| C | 4.376869 | -3.051942 | 2.836024 |
| H | 5.222161 | -2.580675 | 3.333663 |
| C | -1.746208 | 4.027832 | -1.516552 |

| | | | |
|---|---|---|---|
| H | -2.522570 | 4.420588 | -2.173074 |
| C | 2.012016 | 3.589181 | 1.964389 |
| C | 4.627145 | 0.551501 | -2.057463 |
| H | 5.247708 | 1.355677 | -2.451451 |
| C | 3.362185 | 1.791182 | 2.941849 |
| H | 4.176594 | 1.472382 | 3.591621 |
| C | 2.805695 | -3.119104 | 0.977220 |
| C | 2.294243 | 4.924683 | -2.321002 |
| H | 3.337907 | 5.143662 | -2.538078 |
| C | 2.621814 | 0.868469 | 2.250968 |
| H | 2.837494 | -0.196237 | 2.358463 |
| C | 2.698208 | -4.779138 | 2.787955 |
| H | 2.217828 | -5.654554 | 3.224604 |
| C | 1.054222 | -4.833609 | 0.944637 |
| H | 0.603525 | -5.726301 | 1.379104 |
| C | -2.451458 | -1.041427 | 3.262472 |
| H | -2.469926 | -1.518110 | 4.251737 |
| H | -2.987813 | -1.694119 | 2.565883 |
| H | -3.012010 | -0.102002 | 3.341064 |
| C | 6.181798 | -3.358195 | -1.843592 |
| H | 6.574641 | -4.372464 | -1.811138 |
| C | 1.972299 | 4.180332 | -1.208605 |
| H | 2.763377 | 3.819123 | -0.554328 |
| C | 4.912451 | -3.106732 | -1.378001 |
| H | 4.314435 | -3.927631 | -0.988814 |
| C | 0.518630 | -4.258750 | -0.178940 |
| H | -0.370028 | -4.681058 | -0.644062 |
| C | 1.285403 | 5.412409 | -3.176542 |
| H | 1.553251 | 6.005198 | -4.048353 |
| C | 2.402066 | 5.885332 | 2.668874 |
| H | 2.136530 | 6.939360 | 2.620474 |
| C | -0.304657 | -2.166354 | 2.673853 |
| H | 0.786628 | -2.068648 | 2.618251 |
| H | -0.636067 | -2.705933 | 1.776952 |
| H | -0.516048 | -2.800295 | 3.545975 |
| C | -1.032502 | -1.087485 | -4.379150 |
| H | -1.162554 | -0.653083 | -5.378113 |
| H | -2.035390 | -1.268853 | -3.968199 |
| H | -0.535732 | -2.056398 | -4.508647 |
| C | 3.771365 | -4.187757 | 3.410795 |
| H | 4.153046 | -4.591410 | 4.346074 |
| C | 6.976924 | -2.317571 | -2.365081 |
| H | 7.982118 | -2.529626 | -2.723122 |
| C | -0.859086 | 1.258119 | -3.502769 |
| H | -0.396644 | 1.925489 | -2.764731 |
| H | -1.935432 | 1.228236 | -3.301112 |
| H | -0.725402 | 1.727493 | -4.486196 |
| C | 3.456971 | 5.468871 | 3.506373 |
| H | 4.007370 | 6.202785 | 4.091222 |
| C | -0.214643 | -0.127022 | -3.512166 |
| C | -2.068564 | 3.205453 | -0.467789 |
| H | -3.106564 | 2.952181 | -0.264476 |
| C | -0.307898 | -0.009101 | 3.935345 |
| H | -0.847290 | 0.911871 | 4.184010 |
| H | 0.726015 | 0.252591 | 3.692186 |
| H | -0.284027 | -0.616596 | 4.850205 |
| C | 1.197037 | 0.015622 | -4.091933 |
| H | 1.818474 | -0.881365 | -3.982308 |
| H | 1.737419 | 0.850610 | -3.634872 |
| H | 1.115727 | 0.228132 | -5.165684 |
| Ni | -2.164470 | -0.786140 | -0.703372 |
| H | -4.488375 | -2.023661 | -2.106498 |
| C | -5.859586 | 1.006546 | 2.561830 |
| C | -5.230763 | -0.033308 | 1.881757 |
| C | -4.771184 | 0.131919 | 0.569806 |
| C | -4.996608 | 1.374859 | -0.043670 |
| C | -5.626338 | 2.413299 | 0.630119 |
| C | -6.056682 | 2.236970 | 1.943433 |
| H | -6.210697 | 0.843367 | 3.578894 |
| H | -5.110340 | -0.991057 | 2.382676 |
| H | -4.660174 | 1.537387 | -1.069100 |
| H | -5.783034 | 3.364977 | 0.125143 |
| H | -6.552428 | 3.046888 | 2.473102 |
| C | -4.135550 | -0.963985 | -0.221636 |
| C | -4.850068 | -1.146692 | -1.556844 |
| N | -4.167224 | -2.255739 | 0.479715 |
| H | -5.073489 | -2.583158 | 0.808032 |
| N | -6.358210 | -1.386848 | -1.412669 |
| O | -6.757391 | -1.951499 | -0.393777 |
| O | -7.057694 | -1.014742 | -2.338651 |
| H | -4.784826 | -0.282889 | -2.218920 |
| H | -2.654633 | -0.421023 | -2.051997 |
| C | -3.181914 | -3.124030 | 0.262615 |
| C | -3.283529 | -4.516533 | 0.783127 |
| H | -2.462767 | -4.695367 | 1.487953 |
| H | -4.232475 | -4.716646 | 1.286423 |
| H | -3.159532 | -5.230598 | -0.037226 |
| O | -2.146417 | -2.749825 | -0.353510 |

| | | | |
|---|---|---|---|
| H | -2.459671 | 0.505183 | -1.327535 |

**$X_{R2}$**

| | | | |
|---|---|---|---|
| P | -0.229904 | -0.876461 | -1.772779 |
| P | -1.008573 | 0.042288 | 1.178391 |
| C | 1.574824 | 1.209526 | 1.382230 |
| C | 0.279034 | 3.043495 | 0.250983 |
| C | 1.738779 | 4.914286 | 1.961956 |
| H | 0.936187 | 5.274068 | 1.322210 |
| C | -1.059597 | 2.715264 | 0.443913 |
| C | 2.299793 | -2.580893 | -0.328552 |
| C | 3.030964 | -1.477752 | -1.014102 |
| C | -0.986125 | -0.845279 | 2.840717 |
| C | 0.648546 | 3.861340 | -0.867498 |
| C | 2.475452 | -0.213969 | -1.205025 |
| C | 4.364864 | -1.736084 | -1.482054 |
| C | 3.273497 | 0.829481 | -1.735721 |
| H | 2.831627 | 1.821041 | -1.834283 |
| C | 0.795116 | 0.127603 | 0.670039 |
| H | 1.178235 | -0.836518 | 1.026313 |
| C | 1.144323 | -3.127437 | -0.875061 |
| C | 0.531618 | -2.517736 | -2.100273 |
| H | -0.253798 | -3.161992 | -2.512806 |
| H | 1.286109 | -2.371929 | -2.888402 |
| C | 3.067415 | 3.083264 | 2.883314 |
| C | 1.314453 | 2.566063 | 1.209418 |
| C | -0.371462 | 4.388881 | -1.724984 |
| C | 2.197746 | -4.267242 | 1.481503 |
| C | 1.041438 | 0.129560 | -0.857223 |
| H | 0.847164 | 1.148535 | -1.223655 |
| C | 3.776611 | 4.031743 | 3.658491 |
| H | 4.563111 | 3.674547 | 4.322576 |
| C | 6.456775 | -0.924092 | -2.493596 |
| H | 7.032302 | -0.095828 | -2.905685 |
| C | -1.462409 | 1.786976 | 1.552196 |
| H | -2.545924 | 1.821770 | 1.730645 |
| H | -0.958305 | 2.067890 | 2.486151 |
| C | 5.142212 | -0.668729 | -2.036888 |
| C | -0.003327 | 5.166629 | -2.849324 |
| H | -0.793121 | 5.559476 | -3.489180 |
| C | 3.916275 | -2.531986 | 1.576468 |
| H | 4.401049 | -1.655737 | 1.150371 |
| C | 4.370915 | -3.047371 | 2.768820 |
| H | 5.209304 | -2.573555 | 3.275687 |
| C | -1.725630 | 4.105650 | -1.434050 |
| H | -2.502745 | 4.538365 | -2.063771 |
| C | 2.033676 | 3.527048 | 1.996123 |
| C | 4.569267 | 0.620153 | -2.122427 |
| H | 5.167049 | 1.440940 | -2.517595 |
| C | 3.343908 | 1.700503 | 2.976196 |
| H | 4.139598 | 1.363361 | 3.639820 |
| C | 2.818346 | -3.117958 | 0.894967 |
| C | 2.325425 | 4.905815 | -2.291330 |
| H | 3.370605 | 5.110862 | -2.514825 |
| C | 2.607268 | 0.794792 | 2.258857 |
| H | 2.807051 | -0.273372 | 2.358722 |
| C | 2.702646 | -4.784357 | 2.698859 |
| H | 2.223991 | -5.664550 | 3.127790 |
| C | 1.079098 | -4.845674 | 0.837304 |
| H | 0.627138 | -5.739911 | 1.266704 |
| C | -2.434276 | -1.169661 | 3.198592 |
| H | -2.465351 | -1.683046 | 4.168915 |
| H | -2.905829 | -1.824039 | 2.457371 |
| H | -3.051368 | -0.267457 | 3.283901 |
| C | 6.223970 | -3.248223 | -1.909502 |
| H | 6.642996 | -4.251949 | -1.876577 |
| C | 2.000632 | 4.154848 | -1.184640 |
| H | 2.790516 | 3.772418 | -0.541032 |
| C | 4.948066 | -3.029402 | -1.445361 |
| H | 4.370328 | -3.864942 | -1.056646 |
| C | 0.555212 | -4.275526 | -0.294303 |
| H | -0.324488 | -4.705235 | -0.770224 |
| C | 1.317223 | 5.418048 | -3.133677 |
| H | 1.587633 | 6.013408 | -4.002925 |
| C | 2.438375 | 5.809087 | 2.737073 |
| H | 2.186096 | 6.866725 | 2.697631 |
| C | -0.184106 | -2.140374 | 2.697683 |
| H | 0.898314 | -1.959596 | 2.705039 |
| H | -0.433311 | -2.699950 | 1.787032 |
| H | -0.394658 | -2.791755 | 3.556905 |
| C | -1.077899 | -1.093678 | -4.400106 |
| H | -1.275264 | -0.609303 | -5.364511 |
| H | -2.043042 | -1.440731 | -4.006909 |
| H | -0.450873 | -1.970519 | -4.599904 |
| C | 3.765239 | -4.187659 | 3.334742 |
| H | 4.139545 | -4.591638 | 4.272856 |
| C | 6.992535 | -2.187350 | -2.430705 |
| H | 8.002819 | -2.373806 | -2.788706 |

| | | | |
|---|---|---|---|
| C | -1.235471 | 1.177344 | -3.351789 |
| H | -0.857404 | 1.848638 | -2.570342 |
| H | -2.287228 | 0.961700 | -3.126161 |
| H | -1.200428 | 1.729279 | -4.300321 |
| C | 3.473777 | 5.369528 | 3.587192 |
| H | 4.022313 | 6.089131 | 4.191263 |
| C | -0.396059 | -0.092627 | -3.466898 |
| C | -2.053564 | 3.276137 | -0.392699 |
| H | -3.094684 | 3.057566 | -0.169657 |
| C | -0.392852 | 0.004834 | 3.965713 |
| H | -1.027684 | 0.860373 | 4.222286 |
| H | 0.613626 | 0.374204 | 3.742804 |
| H | -0.319711 | -0.617608 | 4.867502 |
| C | 0.975450 | 0.283480 | -4.036442 |
| H | 1.714726 | -0.525113 | -3.979689 |
| H | 1.398035 | 1.159667 | -3.531252 |
| H | 0.858911 | 0.548221 | -5.095024 |
| Ni | -2.104825 | -0.910507 | -0.590711 |
| H | -4.527749 | -1.860831 | -2.272486 |
| C | -5.854388 | 0.760544 | 2.613883 |
| C | -5.247826 | -0.235679 | 1.852017 |
| C | -4.736937 | 0.037714 | 0.578743 |
| C | -4.890570 | 1.342323 | 0.084511 |
| C | -5.499346 | 2.337177 | 0.838398 |
| C | -5.980330 | 2.052738 | 2.115141 |
| H | -6.243663 | 0.515449 | 3.600244 |
| H | -5.176990 | -1.242472 | 2.258916 |
| H | -4.512890 | 1.584611 | -0.909977 |
| H | -5.604555 | 3.338328 | 0.423235 |
| H | -6.461133 | 2.827944 | 2.707214 |
| C | -4.121235 | -1.005732 | -0.306483 |
| C | -4.780840 | -0.975002 | -1.680527 |
| N | -4.278415 | -2.359306 | 0.250456 |
| H | -5.228276 | -2.700037 | 0.389602 |
| N | -6.306396 | -0.996403 | -1.595917 |
| O | -6.805339 | -1.744380 | -0.751277 |
| O | -6.924835 | -0.290581 | -2.373468 |
| H | -4.545788 | -0.086008 | -2.267281 |
| H | -2.429254 | -1.313358 | -1.975368 |
| C | -3.238432 | -3.179027 | 0.334184 |
| C | -3.418108 | -4.577114 | 0.818984 |
| H | -2.709189 | -4.764804 | 1.633099 |
| H | -4.432202 | -4.782552 | 1.169956 |
| H | -3.175060 | -5.281309 | 0.016048 |
| O | -2.081724 | -2.772602 | 0.028778 |
| H | -2.273139 | 0.480637 | -0.949732 |

**TSVI$_{R2}$**

| | | | |
|---|---|---|---|
| P | -0.404795 | -0.883786 | -1.737373 |
| P | -0.966124 | 0.114486 | 1.323183 |
| C | 1.703620 | 1.071574 | 1.353163 |
| C | 0.509945 | 3.024527 | 0.322695 |
| C | 2.245104 | 4.738166 | 1.953271 |
| H | 1.433337 | 5.177288 | 1.377955 |
| C | -0.836385 | 2.782499 | 0.580749 |
| C | 2.145548 | -2.671103 | -0.414238 |
| C | 2.882557 | -1.590008 | -1.128071 |
| C | -0.905839 | -0.774902 | 2.984979 |
| C | 0.876961 | 3.832744 | -0.803949 |
| C | 2.347069 | -0.313476 | -1.296389 |
| C | 4.184634 | -1.882236 | -1.660876 |
| C | 3.138980 | 0.704662 | -1.881446 |
| H | 2.713816 | 1.704368 | -1.967426 |
| C | 0.793437 | 0.069061 | 0.679137 |
| H | 1.126412 | -0.926471 | 0.997025 |
| C | 0.944564 | -3.162140 | -0.914249 |
| C | 0.321709 | -2.531967 | -2.123266 |
| H | -0.483112 | -3.160461 | -2.523381 |
| H | 1.064712 | -2.397192 | -2.923463 |
| C | 3.463642 | 2.781579 | 2.760231 |
| C | 1.555960 | 2.449739 | 1.212674 |
| C | -0.147485 | 4.426842 | -1.611686 |
| C | 2.024158 | -4.339111 | 1.412597 |
| C | 0.943718 | 0.070703 | -0.872077 |
| H | 0.765432 | 1.100578 | -1.217630 |
| C | 4.318270 | 3.651290 | 3.479083 |
| H | 5.117119 | 3.214666 | 4.077793 |
| C | 6.242635 | -1.123908 | -2.778726 |
| H | 6.814518 | -0.311353 | -3.225675 |
| C | -1.248026 | 1.891885 | 1.716970 |
| H | -2.314527 | 2.017278 | 1.951118 |
| H | -0.671334 | 2.136025 | 2.618245 |
| C | 4.957130 | -0.836637 | -2.261522 |
| C | 0.214647 | 5.205411 | -2.737654 |
| H | -0.579451 | 5.650261 | -3.336796 |
| C | 3.849723 | -2.714531 | 1.413793 |
| H | 4.367740 | -1.872412 | 0.959640 |
| C | 4.325614 | -3.250097 | 2.588703 |

| | | | |
|---|---|---|---|
| H | 5.215335 | -2.827060 | 3.051639 |
| C | -1.502069 | 4.209726 | -1.269837 |
| H | -2.279811 | 4.685618 | -1.867026 |
| C | 2.411619 | 3.329348 | 1.956584 |
| C | 4.407794 | 0.462923 | -2.333847 |
| H | 5.000655 | 1.265913 | -2.771188 |
| C | 3.610946 | 1.378276 | 2.831326 |
| H | 4.413790 | 0.960105 | 3.438314 |
| C | 2.686062 | -3.233135 | 0.788586 |
| C | 2.546589 | 4.814826 | -2.281315 |
| H | 3.591384 | 4.967994 | -2.545266 |
| C | 2.742895 | 0.554302 | 2.164529 |
| H | 2.846541 | -0.528000 | 2.247104 |
| C | 2.549828 | -4.877736 | 2.612038 |
| H | 2.035535 | -5.721683 | 3.071373 |
| C | 0.842947 | -4.850738 | 0.826620 |
| H | 0.352565 | -5.706857 | 1.290057 |
| C | -2.354122 | -1.056675 | 3.385154 |
| H | -2.376504 | -1.533618 | 4.374217 |
| H | -2.844688 | -1.733753 | 2.674909 |
| H | -2.955117 | -0.140997 | 3.444596 |
| C | 5.988167 | -3.437901 | -2.166547 |
| H | 6.385701 | -4.450733 | -2.146807 |
| C | 2.228642 | 4.059090 | -1.176212 |
| H | 3.024870 | 3.620567 | -0.578345 |
| C | 4.740204 | -3.188209 | -1.644947 |
| H | 4.164102 | -4.009563 | -1.225105 |
| C | 0.310792 | -4.263534 | -0.292418 |
| H | -0.611257 | -4.645070 | -0.728364 |
| C | 1.533392 | 5.396599 | -3.071004 |
| H | 1.799117 | 5.995260 | -3.939489 |
| C | 3.084571 | 5.555598 | 2.673309 |
| H | 2.930796 | 6.632721 | 2.657032 |
| C | -0.162096 | -2.098967 | 2.806607 |
| H | 0.925878 | -1.962518 | 2.758231 |
| H | -0.482356 | -2.641904 | 1.907771 |
| H | -0.357283 | -2.745325 | 3.673186 |
| C | -1.476084 | -1.031728 | -4.260090 |
| H | -1.749200 | -0.542898 | -5.203697 |
| H | -2.410379 | -1.314516 | -3.753552 |
| H | -0.934014 | -1.950090 | -4.514907 |
| C | 3.674985 | -4.344660 | 3.193947 |
| H | 4.064639 | -4.763563 | 4.119276 |
| C | 6.754243 | -2.397629 | -2.730842 |

| | | | |
|---|---|---|---|
| H | 7.742362 | -2.608094 | -3.134498 |
| C | -1.377299 | 1.245989 | -3.234772 |
| H | -0.862232 | 1.918749 | -2.537303 |
| H | -2.398662 | 1.101177 | -2.858607 |
| H | -1.443298 | 1.764604 | -4.200485 |
| C | 4.139021 | 5.012191 | 3.435594 |
| H | 4.798978 | 5.670795 | 3.996194 |
| C | -0.635297 | -0.076013 | -3.411221 |
| C | -1.831146 | 3.397023 | -0.215796 |
| H | -2.872872 | 3.229193 | 0.046744 |
| C | -0.244135 | 0.053573 | 4.087189 |
| H | -0.846375 | 0.923959 | 4.372002 |
| H | 0.759393 | 0.402059 | 3.818022 |
| H | -0.143760 | -0.571411 | 4.984695 |
| C | 0.696789 | 0.206736 | -4.109000 |
| H | 1.367726 | -0.660355 | -4.146907 |
| H | 1.238573 | 1.025958 | -3.623247 |
| H | 0.503654 | 0.515121 | -5.144754 |
| Ni | -2.134798 | -0.779830 | -0.370829 |
| H | -4.947909 | -1.551536 | -2.456987 |
| C | -5.560201 | 0.877451 | 2.664283 |
| C | -5.115900 | -0.112470 | 1.792862 |
| C | -4.702060 | 0.205246 | 0.494541 |
| C | -4.772717 | 1.546464 | 0.089373 |
| C | -5.220631 | 2.533892 | 0.959045 |
| C | -5.613817 | 2.206372 | 2.255225 |
| H | -5.881160 | 0.600062 | 3.666710 |
| H | -5.098556 | -1.147601 | 2.127067 |
| H | -4.456914 | 1.825901 | -0.916889 |
| H | -5.268977 | 3.566548 | 0.617237 |
| H | -5.972105 | 2.977796 | 2.932670 |
| C | -4.289059 | -0.857912 | -0.490599 |
| C | -5.073718 | -0.688334 | -1.793462 |
| N | -4.500835 | -2.214531 | 0.009200 |
| H | -5.469017 | -2.520376 | 0.088304 |
| N | -6.568286 | -0.591965 | -1.551307 |
| O | -7.045364 | -1.395788 | -0.745679 |
| O | -7.189641 | 0.250782 | -2.171574 |
| H | -4.806446 | 0.219314 | -2.337710 |
| H | -3.095385 | -0.992725 | -1.462988 |
| C | -3.490181 | -3.014464 | 0.352079 |
| C | -3.780757 | -4.379841 | 0.874639 |
| H | -3.294928 | -4.499550 | 1.849308 |
| H | -4.849290 | -4.582586 | 0.979820 |

| | | | |
|---|---|---|---|
| H | -3.342561 | -5.129634 | 0.207856 |
| O | -2.297085 | -2.629568 | 0.244507 |
| H | -1.987036 | 0.619363 | -0.727930 |

**$XI_{R2}$**

| | | | |
|---|---|---|---|
| P | -0.441303 | -0.660108 | -1.846755 |
| P | -0.825160 | 0.329742 | 1.402370 |
| C | 1.947191 | 0.906276 | 1.326086 |
| C | 0.968932 | 3.013172 | 0.354880 |
| C | 2.999282 | 4.471920 | 1.878687 |
| H | 2.241305 | 5.016393 | 1.320578 |
| C | -0.376764 | 2.964138 | 0.707706 |
| C | 1.720883 | -2.879787 | -0.539790 |
| C | 2.692317 | -1.908921 | -1.116019 |
| C | -0.898792 | -0.596151 | 3.036347 |
| C | 1.366095 | 3.782660 | -0.787448 |
| C | 2.368753 | -0.562032 | -1.278342 |
| C | 3.974458 | -2.386869 | -1.551786 |
| C | 3.343515 | 0.330140 | -1.787891 |
| H | 3.079333 | 1.383132 | -1.877882 |
| C | 0.891755 | 0.035732 | 0.682079 |
| H | 1.089730 | -0.998094 | 0.993217 |
| C | 0.518407 | -3.125076 | -1.192869 |
| C | 0.139585 | -2.321739 | -2.398524 |
| H | -0.675759 | -2.800089 | -2.953645 |
| H | 0.990402 | -2.197675 | -3.084357 |
| C | 3.958835 | 2.372159 | 2.672138 |
| C | 1.979067 | 2.293245 | 1.182080 |
| C | 0.384314 | 4.538395 | -1.508320 |
| C | 1.103749 | -4.573525 | 1.156743 |
| C | 1.029344 | 0.025600 | -0.882670 |
| H | 1.029057 | 1.079715 | -1.199393 |
| C | 4.942360 | 3.120271 | 3.362411 |
| H | 5.690053 | 2.580980 | 3.943265 |
| C | 6.210750 | -1.928991 | -2.472751 |
| H | 6.932930 | -1.205056 | -2.848786 |
| C | -0.823413 | 2.113274 | 1.856238 |
| H | -1.841027 | 2.376656 | 2.174565 |
| H | -0.157601 | 2.254349 | 2.717321 |
| C | 4.941059 | -1.460259 | -2.058749 |
| C | 0.773434 | 5.269597 | -2.656227 |
| H | 0.012155 | 5.836032 | -3.192199 |
| C | 3.179388 | -3.316415 | 1.441307 |
| H | 3.889654 | -2.571714 | 1.088074 |
| C | 3.421690 | -3.982796 | 2.620996 |
| H | 4.317683 | -3.753964 | 3.194820 |
| C | -0.960317 | 4.514478 | -1.069917 |
| H | -1.700939 | 5.107893 | -1.605665 |
| C | 2.970516 | 3.053158 | 1.889792 |
| C | 4.594153 | -0.094036 | -2.149525 |
| H | 5.330745 | 0.615304 | -2.525708 |
| C | 3.916054 | 0.962594 | 2.754508 |
| H | 4.671392 | 0.444504 | 3.344737 |
| C | 2.017653 | -3.583606 | 0.671748 |
| C | 3.048896 | 4.528667 | -2.382441 |
| H | 4.082325 | 4.530859 | -2.723406 |
| C | 2.929915 | 0.258782 | 2.114405 |
| H | 2.889079 | -0.827830 | 2.202036 |
| C | 1.392531 | -5.255448 | 2.363313 |
| H | 0.689322 | -6.010803 | 2.713895 |
| C | -0.082876 | -4.827383 | 0.430207 |
| H | -0.767058 | -5.595080 | 0.792728 |
| C | -2.387769 | -0.653142 | 3.394925 |
| H | -2.515802 | -1.089221 | 4.394882 |
| H | -2.944757 | -1.272676 | 2.680727 |
| H | -2.857619 | 0.340381 | 3.404217 |
| C | 5.565164 | -4.187334 | -1.947366 |
| H | 5.805462 | -5.248317 | -1.922793 |
| C | 2.705140 | 3.815635 | -1.256808 |
| H | 3.470228 | 3.266157 | -0.712396 |
| C | 4.326395 | -3.761471 | -1.528134 |
| H | 3.599886 | -4.492937 | -1.182817 |
| C | -0.372868 | -4.106298 | -0.699511 |
| H | -1.296484 | -4.287574 | -1.249880 |
| C | 2.076380 | 5.262579 | -3.092456 |
| H | 2.360976 | 5.825024 | -3.979046 |
| C | 3.961456 | 5.170476 | 2.569891 |
| H | 3.956191 | 6.258396 | 2.546605 |
| C | -0.364582 | -2.013291 | 2.828815 |
| H | 0.731859 | -2.039204 | 2.780546 |
| H | -0.756737 | -2.484854 | 1.917085 |
| H | -0.657455 | -2.643082 | 3.680066 |
| C | -1.415451 | -0.423501 | -4.390341 |
| H | -1.654305 | 0.201079 | -5.260559 |
| H | -2.368050 | -0.724930 | -3.928783 |
| H | -0.919125 | -1.326692 | -4.764284 |
| C | 2.524851 | -4.966049 | 3.086889 |
| H | 2.731997 | -5.493111 | 4.015930 |

| | | | |
|---|---|---|---|
| C | 6.523435 | -3.265070 | -2.414612 |
| H | 7.501139 | -3.615220 | -2.738994 |
| C | -1.200150 | 1.689102 | -3.071857 |
| H | -0.665687 | 2.212549 | -2.270798 |
| H | -2.238703 | 1.554996 | -2.734338 |
| H | -1.208338 | 2.347908 | -3.951172 |
| C | 4.950739 | 4.492626 | 3.311349 |
| H | 5.710089 | 5.057445 | 3.847939 |
| C | -0.535694 | 0.359810 | -3.413406 |
| C | -1.328488 | 3.727892 | -0.009161 |
| H | -2.365963 | 3.697897 | 0.319584 |
| C | -0.121036 | 0.094743 | 4.155574 |
| H | -0.582372 | 1.041641 | 4.458664 |
| H | 0.922749 | 0.292104 | 3.881524 |
| H | -0.110992 | -0.554794 | 5.041188 |
| C | 0.831522 | 0.635437 | -4.037368 |
| H | 1.439440 | -0.268859 | -4.164905 |
| H | 1.411831 | 1.350168 | -3.440073 |
| H | 0.698183 | 1.083416 | -5.031130 |
| Ni | -1.869780 | -0.437356 | -0.280004 |
| H | -5.509500 | -0.791244 | -2.714886 |
| C | -5.772022 | 0.924220 | 2.670743 |
| C | -5.566098 | 0.046000 | 1.609097 |
| C | -4.879822 | 0.466736 | 0.470351 |
| C | -4.407909 | 1.781856 | 0.402496 |
| C | -4.623620 | 2.660239 | 1.458599 |
| C | -5.304703 | 2.232230 | 2.597841 |
| H | -6.307619 | 0.583446 | 3.554156 |
| H | -5.942198 | -0.973741 | 1.673503 |
| H | -3.858973 | 2.115099 | -0.482857 |
| H | -4.266392 | 3.687105 | 1.394282 |
| H | -5.474404 | 2.921494 | 3.421982 |
| C | -4.684417 | -0.443221 | -0.728643 |
| C | -5.602775 | -0.078095 | -1.888405 |
| N | -4.797074 | -1.858329 | -0.410390 |
| H | -5.742333 | -2.228728 | -0.394347 |
| N | -7.055802 | -0.106658 | -1.480739 |
| O | -7.431939 | -1.112648 | -0.870621 |
| O | -7.749368 | 0.845788 | -1.781187 |
| H | -5.409250 | 0.934368 | -2.247607 |
| H | -3.662480 | -0.295787 | -1.143922 |
| C | -3.786552 | -2.575772 | 0.113136 |
| C | -4.076497 | -3.967355 | 0.569754 |
| H | -3.700927 | -4.090302 | 1.591261 |
| H | -5.139100 | -4.221017 | 0.542789 |
| H | -3.528259 | -4.681213 | -0.054548 |
| O | -2.623154 | -2.131507 | 0.212762 |
| H | -1.396469 | 0.909359 | -0.573595 |

**TSIV$_{R3}$**

| | | | |
|---|---|---|---|
| P | 0.459512 | -0.023672 | -1.659366 |
| P | 0.417644 | -0.938508 | 1.373055 |
| C | -2.346936 | -0.300597 | 1.456645 |
| C | -2.284526 | -2.642532 | 0.502033 |
| C | -4.797916 | -3.110691 | 1.912817 |
| H | -4.295362 | -3.932041 | 1.408233 |
| C | -1.057995 | -3.158136 | 0.909831 |
| C | -0.270901 | 2.999658 | -0.430762 |
| C | -1.635726 | 2.645262 | -0.915555 |
| C | 1.143467 | -0.080574 | 2.871612 |
| C | -2.916986 | -3.198835 | -0.659021 |
| C | -2.030601 | 1.320433 | -1.104805 |
| C | -2.543715 | 3.703268 | -1.265772 |
| C | -3.334177 | 1.028661 | -1.573283 |
| H | -3.616897 | -0.017402 | -1.697734 |
| C | -1.027629 | 0.077679 | 0.818618 |
| H | -0.759802 | 1.089684 | 1.163473 |
| C | 0.844576 | 2.620223 | -1.172435 |
| C | 0.707716 | 1.674518 | -2.319167 |
| H | 1.592136 | 1.681298 | -2.960659 |
| H | -0.168064 | 1.902332 | -2.943952 |
| C | -4.860182 | -0.784478 | 2.652142 |
| C | -2.936433 | -1.555708 | 1.291077 |
| C | -2.311424 | -4.314593 | -1.324128 |
| C | 1.234357 | 4.241916 | 1.098517 |
| C | -1.160451 | 0.143131 | -0.721469 |
| H | -1.695347 | -0.769826 | -1.009905 |
| C | -6.108025 | -1.047465 | 3.266692 |
| H | -6.604414 | -0.237101 | 3.799158 |
| C | -4.768907 | 4.420364 | -2.040045 |
| H | -5.771212 | 4.150748 | -2.371430 |
| C | -0.293934 | -2.498116 | 2.013803 |
| H | 0.527627 | -3.130134 | 2.368609 |
| H | -0.938975 | -2.258320 | 2.870228 |
| C | -3.864745 | 3.379997 | -1.716998 |
| C | -2.911936 | -4.836960 | -2.494576 |
| H | -2.435907 | -5.684278 | -2.987295 |
| C | -1.144399 | 4.076871 | 1.647976 |

| | | | |
|---|---|---|---|
| H | -2.151273 | 3.749231 | 1.392392 |
| C | -0.923903 | 4.778716 | 2.811201 |
| H | -1.755559 | 4.987532 | 3.481418 |
| C | -1.109317 | -4.858158 | -0.814401 |
| H | -0.669055 | -5.722843 | -1.309801 |
| C | -4.191116 | -1.827982 | 1.934150 |
| C | -4.233224 | 2.023309 | -1.852670 |
| H | -5.238841 | 1.780663 | -2.194277 |
| C | -4.251178 | 0.486605 | 2.744765 |
| H | -4.766891 | 1.282449 | 3.280345 |
| C | -0.080184 | 3.779183 | 0.757723 |
| C | -4.664194 | -3.189462 | -2.354905 |
| H | -5.577290 | -2.757289 | -2.759496 |
| C | -3.023823 | 0.710763 | 2.179087 |
| H | -2.552613 | 1.691234 | 2.260627 |
| C | 1.420447 | 4.983870 | 2.289322 |
| H | 2.423615 | 5.336072 | 2.528322 |
| C | 2.322623 | 3.912020 | 0.254564 |
| H | 3.318552 | 4.291971 | 0.484338 |
| C | 2.098736 | -1.051437 | 3.567718 |
| H | 2.643692 | -0.521439 | 4.358895 |
| H | 2.851185 | -1.468423 | 2.882458 |
| H | 1.570980 | -1.887031 | 4.041753 |
| C | -3.079907 | 6.061566 | -1.545661 |
| H | -2.772011 | 7.104139 | -1.501819 |
| C | -4.112501 | -2.665090 | -1.207560 |
| H | -4.598749 | -1.829536 | -0.707125 |
| C | -2.181714 | 5.074571 | -1.213767 |
| H | -1.172677 | 5.350923 | -0.919066 |
| C | 2.134092 | 3.099678 | -0.835603 |
| H | 2.978585 | 2.852136 | -1.479771 |
| C | -4.061158 | -4.284852 | -3.007075 |
| H | -4.508647 | -4.690751 | -3.911724 |
| C | -6.005823 | -3.337052 | 2.530156 |
| H | -6.445786 | -4.331849 | 2.501208 |
| C | 1.889201 | 1.181095 | 2.428330 |
| H | 1.374512 | 1.705883 | 1.614098 |
| H | 2.916156 | 0.969498 | 2.105645 |
| H | 1.963896 | 1.887090 | 3.265059 |
| C | 0.447956 | -2.508588 | -2.928820 |
| H | 0.105401 | -3.151742 | -3.751146 |
| H | -0.007092 | -2.888615 | -2.005327 |
| H | 1.536707 | -2.628515 | -2.853998 |
| C | 0.368036 | 5.242109 | 3.135314 |
| H | 0.527381 | 5.805643 | 4.052186 |
| C | -4.390705 | 5.737226 | -1.950315 |
| H | -5.092468 | 6.529215 | -2.203245 |
| C | -1.433569 | -0.992460 | -3.549520 |
| H | -1.586779 | -1.479933 | -4.521602 |
| H | -1.803312 | 0.036783 | -3.638723 |
| H | -2.065407 | -1.526000 | -2.828672 |
| C | -6.676343 | -2.296061 | 3.204167 |
| H | -7.634764 | -2.487377 | 3.681970 |
| C | 0.055304 | -1.059595 | -3.205152 |
| C | -0.484922 | -4.273714 | 0.256833 |
| H | 0.458368 | -4.672894 | 0.629806 |
| C | 0.013481 | 0.330373 | 3.821838 |
| H | -0.662632 | -0.491920 | 4.083947 |
| H | -0.590356 | 1.146105 | 3.406916 |
| H | 0.457165 | 0.699836 | 4.755194 |
| C | 0.847315 | -0.524781 | -4.402353 |
| H | 1.925753 | -0.468001 | -4.229801 |
| H | 0.499934 | 0.465623 | -4.717003 |
| H | 0.692220 | -1.204479 | -5.249851 |
| Ni | 1.817538 | -1.123367 | -0.250928 |
| H | 3.195290 | -0.404685 | 0.750809 |
| C | 4.095985 | -0.521293 | 0.088624 |
| N | 4.465723 | 0.850288 | -0.139978 |
| H | 3.739492 | 1.537783 | 0.038248 |
| C | 5.448576 | 1.350544 | -0.980386 |
| O | 5.475556 | 2.546517 | -1.215516 |
| C | 6.454972 | 0.382503 | -1.541425 |
| H | 6.303289 | -0.663139 | -1.253394 |
| H | 6.442015 | 0.461490 | -2.633464 |
| H | 7.456003 | 0.680777 | -1.214244 |
| C | 3.651598 | -1.363538 | -1.136668 |
| H | 4.268367 | -2.259398 | -1.246367 |
| N | 3.679260 | -0.781331 | -2.503228 |
| O | 3.903873 | -1.583081 | -3.406349 |
| O | 3.460095 | 0.418155 | -2.667450 |
| C | 5.067991 | -1.284686 | 0.983503 |
| C | 5.019468 | -2.678551 | 1.105030 |
| C | 5.958671 | -0.574606 | 1.794708 |
| C | 5.852126 | -3.344906 | 1.999343 |
| H | 4.326169 | -3.273412 | 0.511964 |
| C | 6.793211 | -1.242594 | 2.684644 |
| H | 5.997842 | 0.509702 | 1.720338 |
| C | 6.744851 | -2.629597 | 2.790674 |

| | | | |
|---|---|---|---|
| H | 5.801974 | -4.429172 | 2.071190 |
| H | 7.486870 | -0.671297 | 3.298060 |
| H | 7.399506 | -3.150511 | 3.485746 |
| H | 2.631327 | -2.336768 | -0.672880 |
| H | 2.124904 | -2.478690 | 0.383791 |

**TSIV$_{R1}$**

| | | | |
|---|---|---|---|
| P | 0.393416 | -0.206550 | -1.666973 |
| P | 0.389317 | -1.061263 | 1.271371 |
| C | -2.279453 | -0.122066 | 1.551498 |
| C | -2.573887 | -2.458932 | 0.658937 |
| C | -4.894313 | -2.670082 | 2.427291 |
| H | -4.548666 | -3.541948 | 1.876523 |
| C | -1.367106 | -3.108626 | 0.891179 |
| C | -0.054057 | 2.919417 | -0.513431 |
| C | -1.390071 | 2.638974 | -1.113934 |
| C | 1.263586 | -0.268625 | 2.721426 |
| C | -3.413556 | -2.906977 | -0.411586 |
| C | -1.935432 | 1.359291 | -1.185468 |
| C | -2.131996 | 3.746521 | -1.653140 |
| C | -3.255416 | 1.178238 | -1.666479 |
| H | -3.673481 | 0.170649 | -1.665860 |
| C | -0.987146 | 0.086800 | 0.794550 |
| H | -0.592916 | 1.072038 | 1.083159 |
| C | 1.113981 | 2.431512 | -1.092354 |
| C | 1.060676 | 1.424151 | -2.198756 |
| H | 2.057246 | 1.263098 | -2.635062 |
| H | 0.399048 | 1.758403 | -3.013889 |
| C | -4.637810 | -0.334127 | 3.093695 |
| C | -2.998767 | -1.314904 | 1.514096 |
| C | -3.042478 | -4.069305 | -1.161744 |
| C | 1.331846 | 4.214819 | 1.082517 |
| C | -1.187742 | 0.125433 | -0.739725 |
| H | -1.797685 | -0.752717 | -1.004989 |
| C | -5.798823 | -0.467454 | 3.891733 |
| H | -6.136975 | 0.396108 | 4.463295 |
| C | -4.181072 | 4.620613 | -2.700718 |
| H | -5.186046 | 4.435547 | -3.078488 |
| C | -0.417746 | -2.574268 | 1.922128 |
| H | 0.366833 | -3.300669 | 2.164638 |
| H | -0.936824 | -2.312204 | 2.854741 |
| C | -3.453140 | 3.532590 | -2.162751 |
| C | -3.861975 | -4.490222 | -2.237141 |
| H | -3.568064 | -5.376577 | -2.798429 |
| C | -1.097980 | 4.198181 | 1.363660 |
| H | -2.085405 | 3.884224 | 1.028661 |
| C | -0.972505 | 4.987291 | 2.483940 |
| H | -1.862023 | 5.287624 | 3.034181 |
| C | -1.839088 | -4.741103 | -0.838887 |
| H | -1.561647 | -5.628894 | -1.405866 |
| C | -4.174553 | -1.451381 | 2.325992 |
| C | -4.000436 | 2.230062 | -2.126499 |
| H | -5.016123 | 2.072608 | -2.487533 |
| C | -3.914384 | 0.878663 | 3.045984 |
| H | -4.277045 | 1.733632 | 3.615372 |
| C | 0.042859 | 3.779044 | 0.630991 |
| C | -5.357679 | -2.639603 | -1.849259 |
| H | -6.252930 | -2.085853 | -2.125071 |
| C | -2.762933 | 0.971148 | 2.310991 |
| H | -2.199018 | 1.904921 | 2.294261 |
| C | 1.423166 | 5.041778 | 2.227907 |
| H | 2.409890 | 5.370262 | 2.553137 |
| C | 2.483073 | 3.801180 | 0.372883 |
| H | 3.457234 | 4.185378 | 0.674776 |
| C | 2.230973 | -1.287626 | 3.326551 |
| H | 2.834230 | -0.798972 | 4.102301 |
| H | 2.935316 | -1.700359 | 2.593690 |
| H | 1.705250 | -2.125355 | 3.800146 |
| C | -2.322181 | 6.090666 | -2.281757 |
| H | -1.879006 | 7.082153 | -2.347867 |
| C | -4.592810 | -2.213945 | -0.787812 |
| H | -4.890088 | -1.328284 | -0.229415 |
| C | -1.593662 | 5.056618 | -1.741910 |
| H | -0.580566 | 5.245000 | -1.395140 |
| C | 2.377246 | 2.916888 | -0.670568 |
| H | 3.259228 | 2.626720 | -1.243203 |
| C | -4.994308 | -3.789710 | -2.579626 |
| H | -5.609589 | -4.118383 | -3.414313 |
| C | -6.012321 | -2.770702 | 3.221904 |
| H | -6.541259 | -3.719327 | 3.287572 |
| C | 2.011286 | 0.962435 | 2.217587 |
| H | 1.335977 | 1.676118 | 1.732604 |
| H | 2.808638 | 0.719149 | 1.505136 |
| H | 2.483344 | 1.485477 | 3.060068 |
| C | -0.373530 | -2.513177 | -2.948597 |
| H | -0.801543 | -3.009949 | -3.829837 |
| H | -1.109590 | -2.598947 | -2.139675 |
| H | 0.526233 | -3.062187 | -2.652155 |

| | | | |
|---|---|---|---|
| C | 0.297420 | 5.418201 | 2.920297 |
| H | 0.381034 | 6.049518 | 3.802171 |
| C | -3.631071 | 5.877853 | -2.758534 |
| H | -4.196755 | 6.705865 | -3.180293 |
| C | -1.348871 | -0.415043 | -3.852517 |
| H | -1.498087 | -0.816523 | -4.863114 |
| H | -1.296312 | 0.677755 | -3.937486 |
| H | -2.242118 | -0.668811 | -3.269426 |
| C | -6.477113 | -1.659987 | 3.955619 |
| H | -7.365981 | -1.752819 | 4.575843 |
| C | -0.083927 | -1.049020 | -3.266917 |
| C | -1.006484 | -4.249718 | 0.135045 |
| H | -0.061185 | -4.748052 | 0.353125 |
| C | 0.250508 | 0.175194 | 3.780697 |
| H | -0.418273 | -0.629507 | 4.109157 |
| H | -0.368761 | 1.009957 | 3.432562 |
| H | 0.797749 | 0.525666 | 4.665291 |
| C | 1.069516 | -0.904674 | -4.262983 |
| H | 2.024846 | -1.248889 | -3.856131 |
| H | 1.187475 | 0.129367 | -4.607498 |
| H | 0.847416 | -1.516118 | -5.146367 |
| Ni | 1.601943 | -1.510048 | -0.424938 |
| H | 3.354304 | -0.992254 | 0.620419 |
| C | 4.330155 | -1.015418 | 0.085192 |
| N | 4.167731 | 0.060896 | -0.881878 |
| H | 3.347860 | 0.647721 | -0.765867 |
| C | 4.874013 | 0.332823 | -2.031327 |
| O | 4.462712 | 1.195197 | -2.800406 |
| C | 6.131128 | -0.456578 | -2.267589 |
| H | 5.901858 | -1.406078 | -2.767200 |
| H | 6.780834 | 0.116814 | -2.931221 |
| H | 6.665459 | -0.684846 | -1.337960 |
| C | 4.465912 | -2.396222 | -0.517094 |
| H | 5.438582 | -2.879536 | -0.492403 |
| N | 3.811559 | -2.675466 | -1.698012 |
| O | 4.268181 | -3.403779 | -2.572906 |
| O | 2.585419 | -2.216355 | -1.855223 |
| C | 5.380363 | -0.761390 | 1.147875 |
| C | 5.807184 | -1.802962 | 1.978862 |
| C | 5.849525 | 0.532242 | 1.388163 |
| C | 6.692147 | -1.556491 | 3.023500 |
| H | 5.441127 | -2.816973 | 1.815203 |
| C | 6.739229 | 0.776505 | 2.430342 |
| H | 5.514902 | 1.349640 | 0.749981 |
| C | 7.162530 | -0.265654 | 3.250599 |
| H | 7.018154 | -2.377784 | 3.658265 |
| H | 7.105695 | 1.787228 | 2.598692 |
| H | 7.860300 | -0.073738 | 4.062877 |
| H | 3.164696 | -2.958110 | 0.311168 |
| H | 2.339294 | -2.812797 | 0.535387 |

## (NOAc-H)

**TSII$_{S1}$**

| | | | |
|---|---|---|---|
| P | -0.288748 | -1.034807 | -1.693314 |
| P | -1.251179 | -0.197177 | 1.115750 |
| C | 1.059423 | 1.471696 | 1.349078 |
| C | -0.588495 | 2.953342 | 0.130225 |
| C | 0.455933 | 5.159844 | 1.714744 |
| H | -0.431391 | 5.303315 | 1.102890 |
| C | -1.852790 | 2.407966 | 0.326054 |
| C | 2.380060 | -2.311623 | -0.103659 |
| C | 3.012981 | -1.148025 | -0.791077 |
| C | -1.213676 | -0.999414 | 2.806790 |
| C | -0.361200 | 3.796187 | -1.007482 |
| C | 2.291130 | 0.001515 | -1.114824 |
| C | 4.392925 | -1.238866 | -1.179682 |
| C | 2.936859 | 1.088520 | -1.752972 |
| H | 2.350252 | 1.981333 | -1.978378 |
| C | 0.525480 | 0.210957 | 0.710076 |
| H | 1.056873 | -0.641856 | 1.159431 |
| C | 1.334745 | -2.997922 | -0.714151 |
| C | 0.760803 | -2.500592 | -2.005656 |
| H | 0.140267 | -3.263518 | -2.487525 |
| H | 1.554821 | -2.201508 | -2.702655 |
| C | 2.180304 | 3.712894 | 2.658809 |
| C | 0.514580 | 2.729976 | 1.106853 |
| C | -1.460946 | 4.164500 | -1.848973 |
| C | 2.324386 | -3.966186 | 1.736317 |
| C | 0.818850 | 0.167743 | -0.812511 |
| H | 0.509874 | 1.143387 | -1.211463 |
| C | 2.706505 | 4.837758 | 3.337845 |
| H | 3.580364 | 4.697412 | 3.973140 |
| C | 6.388084 | -0.209708 | -2.191511 |
| H | 6.851259 | 0.654278 | -2.667070 |
| C | -2.096219 | 1.411867 | 1.418339 |
| H | -3.167960 | 1.202091 | 1.533901 |
| H | -1.729724 | 1.783157 | 2.387604 |
| C | 5.025772 | -0.121981 | -1.816414 |

| | | | | | | | |
|---|---|---|---|---|---|---|---|
| C | -1.226427 | 4.976431 | -2.984715 | H | -0.608813 | 1.779712 | -2.852340 |
| H | -2.075447 | 5.254196 | -3.608574 | H | -2.175314 | 1.025714 | -2.494802 |
| C | 3.833991 | -2.049898 | 1.914603 | C | 3.737367 | -3.689134 | 3.692251 |
| H | 4.254340 | -1.136567 | 1.496454 | H | 4.085718 | -4.037172 | 4.662328 |
| C | 4.254895 | -2.496307 | 3.146691 | C | 7.106635 | -1.359708 | -1.976268 |
| H | 5.000207 | -1.928506 | 3.700333 | H | 8.151548 | -1.417727 | -2.273219 |
| C | -2.753416 | 3.686535 | -1.534079 | C | 0.533520 | -0.245961 | -4.263721 |
| H | -3.598636 | 4.004161 | -2.143822 | H | 0.263449 | 0.248573 | -5.205898 |
| C | 1.038804 | 3.869645 | 1.808249 | H | 0.942261 | -1.228990 | -4.522774 |
| C | 4.266988 | 1.040035 | -2.078711 | H | 1.332786 | 0.345804 | -3.799841 |
| H | 4.750908 | 1.889837 | -2.558810 | C | 2.125371 | 6.075806 | 3.208864 |
| C | 2.755322 | 2.430227 | 2.804367 | H | 2.536919 | 6.933204 | 3.737194 |
| H | 3.639716 | 2.315499 | 3.429911 | C | -0.709590 | -0.346428 | -3.377852 |
| C | 2.859061 | -2.763246 | 1.170196 | C | -2.932643 | 2.811288 | -0.493675 |
| C | 1.132009 | 5.016246 | -2.492156 | H | -3.927551 | 2.442361 | -0.251459 |
| H | 2.137447 | 5.340677 | -2.753225 | C | -0.268006 | -0.266251 | 3.760775 |
| C | 2.197731 | 1.345101 | 2.183281 | H | -0.455603 | 0.812669 | 3.827716 |
| H | 2.633681 | 0.354132 | 2.317121 | H | 0.782056 | -0.408439 | 3.481377 |
| C | 2.794689 | -4.407344 | 2.996682 | H | -0.392138 | -0.682393 | 4.768706 |
| H | 2.383570 | -5.328013 | 3.409998 | C | -1.731674 | -1.295517 | -4.009710 |
| C | 1.304931 | -4.662443 | 1.045076 | H | -2.670755 | -1.320639 | -3.440489 |
| H | 0.906287 | -5.579436 | 1.478002 | H | -1.365902 | -2.326228 | -4.086198 |
| C | -2.632893 | -0.986882 | 3.376149 | H | -1.969550 | -0.951383 | -5.024140 |
| H | -2.650112 | -1.558865 | 4.312233 | Ni | -1.980900 | -1.526231 | -0.387567 |
| H | -3.358888 | -1.451983 | 2.695835 | H | -2.866274 | -1.875784 | 0.766822 |
| H | -2.983140 | 0.026583 | 3.607060 | C | -4.206074 | -1.712002 | -0.530792 |
| C | 6.480634 | -2.474340 | -1.381936 | C | -4.400183 | -3.033570 | -0.926960 |
| H | 7.044058 | -3.393219 | -1.232569 | H | -5.314019 | -3.600329 | -0.800746 |
| C | 0.934305 | 4.250117 | -1.366405 | N | -3.357044 | -3.709578 | -1.491584 |
| H | 1.783465 | 3.975559 | -0.743306 | O | -3.456637 | -4.787573 | -2.067184 |
| C | 5.162648 | -2.416438 | -0.993766 | O | -2.166025 | -3.145511 | -1.369705 |
| H | 4.698767 | -3.292570 | -0.547687 | C | -5.219497 | -1.003225 | 0.242625 |
| C | 0.796672 | -4.168961 | -0.129282 | C | -5.897823 | -1.630637 | 1.297800 |
| H | -0.015265 | -4.682767 | -0.642191 | C | -5.536345 | 0.322496 | -0.080169 |
| C | 0.043283 | 5.391456 | -3.306280 | C | -6.867406 | -0.941488 | 2.013900 |
| H | 0.212595 | 6.005917 | -4.187836 | H | -5.638172 | -2.655004 | 1.566140 |
| C | 0.983535 | 6.230989 | 2.397055 | C | -6.511975 | 1.009546 | 0.631638 |
| H | 0.512195 | 7.207851 | 2.310575 | H | -5.026740 | 0.793475 | -0.921159 |
| C | -0.717164 | -2.431599 | 2.634734 | C | -7.175568 | 0.378586 | 1.683050 |
| H | 0.258251 | -2.457442 | 2.134647 | H | -7.384142 | -1.431978 | 2.835623 |
| H | -1.408225 | -3.045537 | 2.042965 | H | -6.761410 | 2.034252 | 0.363615 |
| H | -0.592061 | -2.907294 | 3.616582 | H | -7.936234 | 0.915555 | 2.245446 |
| C | -1.333225 | 1.033710 | -3.201140 | H | -3.636558 | -1.055039 | -1.227975 |
| H | -1.717063 | 1.391709 | -4.165465 | | | | |

**TSIII$_{S1}$**

| | | | |
|---|---|---|---|
| P | -0.759020 | -0.245243 | 1.433265 |
| P | -0.155333 | -1.166463 | -1.398070 |
| C | 2.333780 | 0.216227 | -1.337110 |
| C | 2.888118 | -2.001701 | -0.274757 |
| C | 5.486555 | -1.806844 | -1.608784 |
| H | 5.222482 | -2.707891 | -1.060145 |
| C | 1.858833 | -2.850048 | -0.669981 |
| C | -0.570137 | 2.901420 | 0.238111 |
| C | 0.672970 | 2.832538 | 1.056056 |
| C | -0.948794 | -0.606331 | -2.994196 |
| C | 3.601471 | -2.288466 | 0.935232 |
| C | 1.370475 | 1.644201 | 1.263744 |
| C | 1.158247 | 4.045063 | 1.654921 |
| C | 2.606715 | 1.665730 | 1.955289 |
| H | 3.153666 | 0.729247 | 2.068856 |
| C | 0.923094 | 0.215583 | -0.793472 |
| H | 0.424382 | 1.118608 | -1.172587 |
| C | -1.724922 | 2.229474 | 0.625887 |
| C | -1.704669 | 1.285900 | 1.788923 |
| H | -2.717787 | 0.980847 | 2.080703 |
| H | -1.227188 | 1.743170 | 2.670089 |
| C | 4.914029 | 0.403977 | -2.473250 |
| C | 3.243787 | -0.814822 | -1.105348 |
| C | 3.293168 | -3.479285 | 1.670296 |
| C | -1.845348 | 3.931438 | -1.619496 |
| C | 0.884308 | 0.306504 | 0.751127 |
| H | 1.568673 | -0.468179 | 1.129232 |
| C | 6.198246 | 0.470003 | -3.064758 |
| H | 6.460565 | 1.359586 | -3.636214 |
| C | 2.885102 | 5.235795 | 2.942448 |
| H | 3.838242 | 5.209536 | 3.469622 |
| C | 1.009691 | -2.504438 | -1.854762 |
| H | 0.417752 | -3.364604 | -2.184500 |
| H | 1.616323 | -2.157392 | -2.701703 |
| C | 2.403190 | 4.038157 | 2.362137 |
| C | 3.970050 | -3.739347 | 2.886299 |
| H | 3.722072 | -4.648017 | 3.434146 |
| C | 0.563692 | 4.327145 | -1.476412 |
| H | 1.518175 | 4.180248 | -0.973486 |
| C | 0.505582 | 5.088290 | -2.621419 |
| H | 1.415636 | 5.534077 | -3.018296 |
| C | 2.294793 | -4.353679 | 1.181000 |
| H | 2.073274 | -5.264692 | 1.735907 |
| C | 4.544715 | -0.750248 | -1.709287 |
| C | 3.120435 | 2.825280 | 2.471626 |
| H | 4.078683 | 2.821783 | 2.990067 |
| C | 3.979050 | 1.450514 | -2.632956 |
| H | 4.265611 | 2.333510 | -3.202825 |
| C | -0.602241 | 3.723236 | -0.937166 |
| C | 5.220579 | -1.692672 | 2.652087 |
| H | 5.968205 | -1.002875 | 3.038645 |
| C | 2.724284 | 1.348634 | -2.092737 |
| H | 2.001536 | 2.155062 | -2.230722 |
| C | -1.871976 | 4.734882 | -2.784866 |
| H | -2.827349 | 4.887715 | -3.286278 |
| C | -3.012380 | 3.304992 | -1.124791 |
| H | -3.965132 | 3.479975 | -1.624818 |
| C | -1.794889 | -1.757729 | -3.543955 |
| H | -2.242178 | -1.452009 | -4.498187 |
| H | -2.618695 | -2.022491 | -2.867977 |
| H | -1.204065 | -2.662077 | -3.739057 |
| C | 0.921942 | 6.408062 | 2.189324 |
| H | 0.339999 | 7.326235 | 2.142564 |
| C | 4.587313 | -1.414603 | 1.462541 |
| H | 4.840882 | -0.509373 | 0.913866 |
| C | 0.432974 | 5.263954 | 1.603947 |
| H | -0.533519 | 5.291501 | 1.106701 |
| C | -2.945136 | 2.453701 | -0.052121 |
| H | -3.844490 | 1.956832 | 0.307263 |
| C | 4.911619 | -2.864560 | 3.373097 |
| H | 5.420615 | -3.073682 | 4.311563 |
| C | 6.723333 | -1.716027 | -2.203710 |
| H | 7.424761 | -2.543074 | -2.114197 |
| C | -1.845534 | 0.587286 | -2.683519 |
| H | -1.275995 | 1.451351 | -2.321592 |
| H | -2.608315 | 0.351637 | -1.930190 |
| H | -2.366989 | 0.909419 | -3.595129 |
| C | -0.081253 | -2.500732 | 2.873503 |
| H | 0.285639 | -2.953139 | 3.805010 |
| H | 0.713015 | -2.607871 | 2.125202 |
| H | -0.955239 | -3.070752 | 2.537437 |
| C | -0.722667 | 5.301577 | -3.280679 |
| H | -0.755629 | 5.912774 | -4.179918 |
| C | 2.164097 | 6.401785 | 2.855351 |
| H | 2.542337 | 7.316263 | 3.307299 |
| C | 0.762096 | -0.339838 | 3.789119 |
| H | 0.846529 | -0.731030 | 4.811207 |

| | | | |
|---|---|---|---|
| H | 0.645459 | 0.748601 | 3.863277 |
| H | 1.715489 | -0.550572 | 3.289465 |
| C | 7.090071 | -0.565718 | -2.931881 |
| H | 8.073736 | -0.505641 | -3.392642 |
| C | -0.423674 | -1.032439 | 3.111948 |
| C | 1.575816 | -4.028553 | 0.059393 |
| H | 0.771154 | -4.678054 | -0.284783 |
| C | 0.109629 | -0.199638 | -4.020453 |
| H | 0.760916 | -1.030654 | -4.314256 |
| H | 0.744121 | 0.619916 | -3.663045 |
| H | -0.394417 | 0.152272 | -4.930154 |
| C | -1.653172 | -0.903860 | 4.014188 |
| H | -2.539189 | -1.371835 | 3.578770 |
| H | -1.878031 | 0.140627 | 4.260560 |
| H | -1.436480 | -1.415609 | 4.960553 |
| Ni | -1.594306 | -1.721120 | 0.102901 |
| H | -3.589412 | -1.171522 | -0.781292 |
| C | -4.667495 | -1.397669 | -0.666232 |
| C | -4.804641 | -2.566257 | 0.254495 |
| H | -5.782355 | -3.012018 | 0.424962 |
| N | -4.017054 | -2.690593 | 1.350304 |
| O | -4.256955 | -3.441281 | 2.295025 |
| O | -2.884496 | -1.975859 | 1.438705 |
| C | -5.391954 | -0.137511 | -0.257115 |
| C | -5.649948 | 0.161538 | 1.083963 |
| C | -5.782243 | 0.778831 | -1.238491 |
| C | -6.271475 | 1.358502 | 1.434680 |
| H | -5.373607 | -0.549753 | 1.862877 |
| C | -6.407661 | 1.971890 | -0.890464 |
| H | -5.593612 | 0.548855 | -2.288756 |
| C | -6.648840 | 2.268315 | 0.449833 |
| H | -6.470059 | 1.575837 | 2.482486 |
| H | -6.717920 | 2.668428 | -1.667983 |
| H | -7.142012 | 3.198592 | 0.724214 |
| C | -2.419077 | -4.415535 | -1.290309 |
| O | -1.789756 | -3.476361 | -0.768211 |
| O | -3.713732 | -4.528706 | -1.301970 |
| C | -1.710882 | -5.546229 | -1.958965 |
| H | -2.250514 | -5.857851 | -2.856898 |
| H | -0.680358 | -5.283286 | -2.207140 |
| H | -1.694953 | -6.408552 | -1.282338 |
| H | -4.145344 | -3.789155 | -0.705499 |
| H | -5.001386 | -1.693943 | -1.672350 |

**TSII$_{R1}$**

| | | | |
|---|---|---|---|
| P | 0.473415 | -0.157854 | -1.929493 |
| P | 0.958535 | -0.895530 | 1.034457 |
| C | -1.742943 | -0.156871 | 1.597983 |
| C | -1.967157 | -2.531678 | 0.761973 |
| C | -4.041170 | -2.876767 | 2.787213 |
| H | -3.682766 | -3.734853 | 2.223485 |
| C | -0.698982 | -3.096269 | 0.845344 |
| C | 0.147120 | 2.973231 | -0.751012 |
| C | -1.266931 | 2.679064 | -1.124500 |
| C | 1.815648 | -0.082559 | 2.481083 |
| C | -2.907942 | -3.070158 | -0.176189 |
| C | -1.789377 | 1.386075 | -1.113422 |
| C | -2.115026 | 3.774610 | -1.506187 |
| C | -3.169513 | 1.181782 | -1.358539 |
| H | -3.561157 | 0.165566 | -1.292281 |
| C | -0.557394 | 0.135711 | 0.706248 |
| H | -0.204518 | 1.148504 | 0.953675 |
| C | 1.205666 | 2.459586 | -1.491841 |
| C | 0.945280 | 1.486553 | -2.597203 |
| H | 1.829107 | 1.350929 | -3.232060 |
| H | 0.122210 | 1.822147 | -3.245789 |
| C | -3.911489 | -0.510687 | 3.374596 |
| C | -2.368156 | -1.401587 | 1.648656 |
| C | -2.572405 | -4.252305 | -0.913130 |
| C | 1.780493 | 4.226117 | 0.629079 |
| C | -0.960260 | 0.162643 | -0.787832 |
| H | -1.593179 | -0.722473 | -0.957368 |
| C | -4.966940 | -0.714250 | 4.295067 |
| H | -5.313198 | 0.135653 | 4.882185 |
| C | -4.342358 | 4.619040 | -2.128622 |
| H | -5.397135 | 4.417432 | -2.312681 |
| C | 0.340478 | -2.480228 | 1.727514 |
| H | 1.202309 | -3.145104 | 1.855608 |
| H | -0.062131 | -2.262991 | 2.727762 |
| C | -3.504500 | 3.538369 | -1.763498 |
| C | -3.493781 | -4.764751 | -1.857793 |
| H | -3.224684 | -5.665346 | -2.409004 |
| C | -0.579656 | 4.251143 | 1.269154 |
| H | -1.610633 | 3.951935 | 1.088544 |
| C | -0.274236 | 5.030582 | 2.361601 |
| H | -1.065167 | 5.337018 | 3.043326 |
| C | -1.301171 | -4.844177 | -0.724106 |
| H | -1.050633 | -5.746517 | -1.280668 |

| | | | |
|---|---|---|---|
| C | -3.439180 | -1.608125 | 2.584056 |
| C | -4.007819 | 2.222780 | -1.654741 |
| H | -5.069157 | 2.046745 | -1.825907 |
| C | -3.305068 | 0.756408 | 3.222542 |
| H | -3.683468 | 1.597374 | 3.802449 |
| C | 0.429630 | 3.823064 | 0.369222 |
| C | -5.017547 | -2.958638 | -1.384520 |
| H | -5.963641 | -2.456790 | -1.578211 |
| C | -2.244185 | 0.918041 | 2.372934 |
| H | -1.766804 | 1.894297 | 2.272523 |
| C | 2.055498 | 5.051895 | 1.745742 |
| H | 3.086416 | 5.356501 | 1.924925 |
| C | 2.815907 | 3.739185 | -0.202791 |
| H | 3.840127 | 4.059837 | -0.012077 |
| C | 2.832220 | -1.069779 | 3.054766 |
| H | 3.477189 | -0.551347 | 3.776440 |
| H | 3.486034 | -1.497521 | 2.285991 |
| H | 2.344346 | -1.894697 | 3.587339 |
| C | -2.465493 | 6.123444 | -2.041319 |
| H | -2.061812 | 7.125729 | -2.169149 |
| C | -4.155142 | -2.447779 | -0.441592 |
| H | -4.427255 | -1.548971 | 0.109280 |
| C | -1.628055 | 5.096891 | -1.672384 |
| H | -0.570794 | 5.300296 | -1.520283 |
| C | 2.537452 | 2.846731 | -1.207167 |
| H | 3.340243 | 2.455661 | -1.834965 |
| C | -4.689570 | -4.130729 | -2.096265 |
| H | -5.383039 | -4.528901 | -2.833671 |
| C | -5.058229 | -3.044589 | 3.698016 |
| H | -5.495888 | -4.030710 | 3.838449 |
| C | 2.498037 | 1.163910 | 1.937909 |
| H | 1.770928 | 1.855373 | 1.498807 |
| H | 3.231591 | 0.921156 | 1.162821 |
| H | 3.021421 | 1.701336 | 2.740827 |
| C | -0.405454 | -2.526018 | -2.982487 |
| H | -0.936880 | -3.082516 | -3.766216 |
| H | -1.009756 | -2.596078 | -2.070919 |
| H | 0.554368 | -3.019560 | -2.798845 |
| C | 1.052846 | 5.443730 | 2.599814 |
| H | 1.280100 | 6.068075 | 3.461099 |
| C | -3.838098 | 5.889595 | -2.261636 |
| H | -4.489715 | 6.712582 | -2.547200 |
| C | -1.600668 | -0.501299 | -3.786524 |
| H | -1.915783 | -0.966824 | -4.729389 |

| | | | |
|---|---|---|---|
| H | -1.598138 | 0.584935 | -3.943913 |
| H | -2.369779 | -0.736312 | -3.041184 |
| C | -5.534689 | -1.954826 | 4.454667 |
| H | -6.342000 | -2.101296 | 5.169013 |
| C | -0.232848 | -1.070567 | -3.400276 |
| C | -0.376310 | -4.254248 | 0.099194 |
| H | 0.619274 | -4.685331 | 0.206299 |
| C | 0.829051 | 0.330153 | 3.574845 |
| H | 0.164688 | -0.481227 | 3.896887 |
| H | 0.202853 | 1.174520 | 3.265379 |
| H | 1.397839 | 0.655702 | 4.455613 |
| C | 0.722900 | -0.945834 | -4.588726 |
| H | 1.729072 | -1.306821 | -4.356391 |
| H | 0.786125 | 0.083958 | -4.961003 |
| H | 0.336150 | -1.558689 | -5.412833 |
| Ni | 2.053818 | -1.171517 | -0.782351 |
| H | 3.056983 | -1.856106 | 0.167605 |
| C | 4.446271 | -1.087267 | -0.576402 |
| C | 4.824584 | -1.977457 | -1.583068 |
| H | 5.785200 | -2.473151 | -1.636779 |
| N | 3.941670 | -2.306704 | -2.566237 |
| O | 4.239340 | -2.907348 | -3.593600 |
| O | 2.677834 | -1.979219 | -2.358800 |
| C | 5.366035 | -0.870565 | 0.541380 |
| C | 5.957503 | -1.963068 | 1.193562 |
| C | 5.700162 | 0.428201 | 0.948410 |
| C | 6.850524 | -1.759590 | 2.236274 |
| H | 5.684490 | -2.974367 | 0.891271 |
| C | 6.603773 | 0.630747 | 1.983859 |
| H | 5.260127 | 1.280822 | 0.430420 |
| C | 7.174621 | -0.462117 | 2.633434 |
| H | 7.295730 | -2.612531 | 2.743429 |
| H | 6.867069 | 1.643022 | 2.282462 |
| H | 7.875999 | -0.303438 | 3.449730 |
| H | 3.874148 | -0.190939 | -0.884803 |

## 7. References


[1] a) M. Zhou, D. J. Dong, B. L. Zhu, H. L. Geng, Y. Wang, X. M. Zhang, *Org. Lett.* **2013**, *15*, 5524; b) Q. Z. Yan, M. Liu, D. Y. Kong, G. F. Zi, G. H. Hou, *Chem. Commun*. **2014**, *50*, 12870.

[2] S. K. Li, T. F. Xiao, D. D. Li, X. M. Zhang, *Org. Lett*. **2015**, *17*, 3782.

[3] Zhao Y.; Truhlar D. G. *J. Chem. Phys.* **2006**, *125*, 194101: 1.

[4] a) Ditchfield, R.; Hehre, W. J.; Pople J. A. *J. Chem. Phys.* **1971**, *54*, 724; b) Hehre, W. J.; Ditchfield, R.; Pople J. A. *J. Chem. Phys.* **1972**, *56*, 2257; c) Hariharan, P. C.; Pople J. A. *Theor. Chem. Acc.* **1973**, *28*, 213.

[5] Grimme, S.; Antony, J.; Ehrlich, S. Krieg, H. *J. Chem. Phys.* **2010**, *132*, 154104.

[6] Marenich, A. V.; Cramer, C. J.; Truhlar D. G. *J. Phys. Chem. B*. **2009**, *113*, 6378.

[7] a) Becke, A. D. *J. Chem. Phys.* **1993**, *98*, 5648; b) C, Lee, W, Yang, R. G. Parr, *Phys. Rev. B* **1988**, *37*, 785.

[8] Gaussian 09, Revision D.01, Frisch, M. J.; Trucks, G. W.; Schlegel, H. B.; Scuseria, G. E.; Robb, M. A.; Cheeseman, J. R.; Scalmani, G.; Barone, V.; Mennucci, B.; Petersson, G. A.; Nakatsuji, H.; Caricato, M.; Li, X.; Hratchian, H. P.; Izmaylov, A. F.; Bloino, J.; Zheng, G.; Sonnenberg, J. L.; Hada, M.; Ehara, M.; Toyota, K.; Fukuda, R.; Hasegawa, J.; Ishida, M.; Nakajima, T.; Honda, Y.; Kitao, O.; Nakai, H.; Vreven, T.; Montgomery, J. A., Jr.; Peralta, J. E.; Ogliaro, F.; Bearpark, M.; Heyd, J. J.; Brothers, E.; Kudin, K. N.; Staroverov, V. N.; Kobayashi, R.; Normand, J.; Raghavachari, K.; Rendell, A.; Burant, J. C.; Iyengar, S. S.; Tomasi, J.; Cossi, M.; Rega, N.; Millam, J. M.; Klene, M.; Knox, J. E.; Cross, J. B.; Bakken, V.; Adamo, C.; Jaramillo, J.; Gomperts, R.; Stratmann, R. E.; Yazyev, O.; Austin, A. J.; Cammi, R.; Pomelli, C.; Ochterski, J. W.; Martin, R. L.; Morokuma, K.; Zakrzewski, V. G.; Voth, G. A.; Salvador, P.; Dannenberg, J. J.; Dapprich, S.; Daniels, A. D.; Farkas, Ö.; Foresman, J. B.; Ortiz, J. V.; Cioslowski, J.; Fox, D. J. Gaussian, Inc., Wallingford CT, **2009**.

[9] Legault, C. Y. CYL View, version 1.0 b; Universite de Sherbrooke, Sherbrooke, Quebec, Canada, **2009**; http://www.cylview.org.

[10] Falivene, L.; Credendino, R.; Poater, A.; Petta, A.; Serra, L.; Oliva, R.; Scarano, V.; Cavallo, L. *Organometallics* **2016**, *35*, 2286.

# 8. NMR and HPLC

$^{1}H$ NMR and $^{13}C$ NMR of **2a**

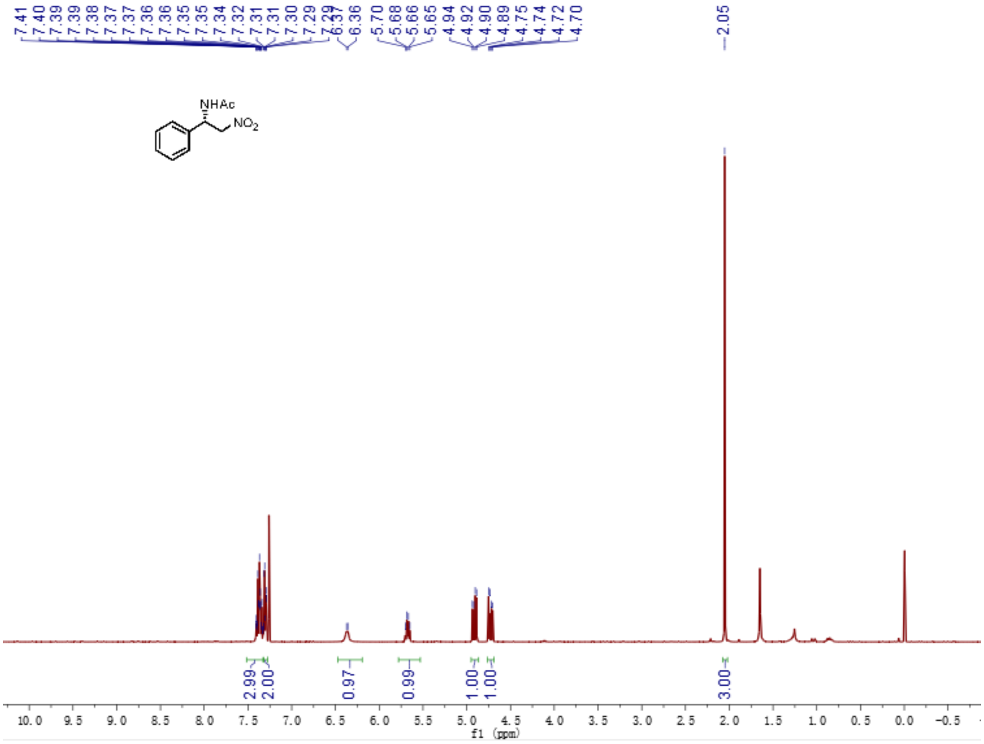


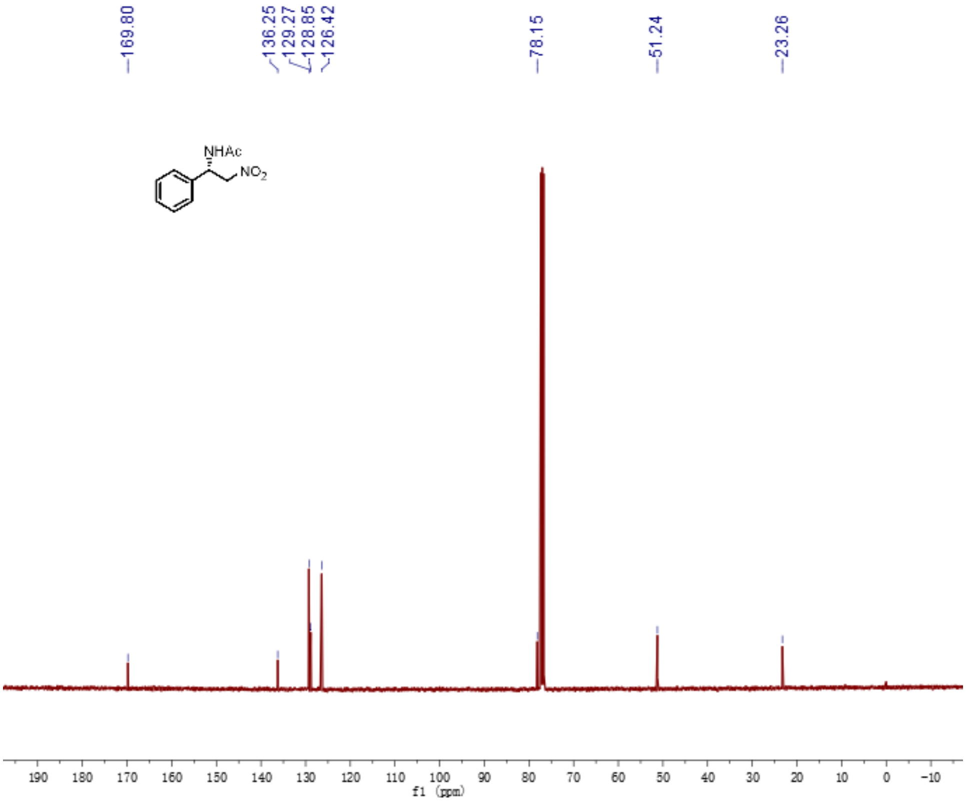

$^{1}$H NMR and $^{13}$C NMR of **2b**

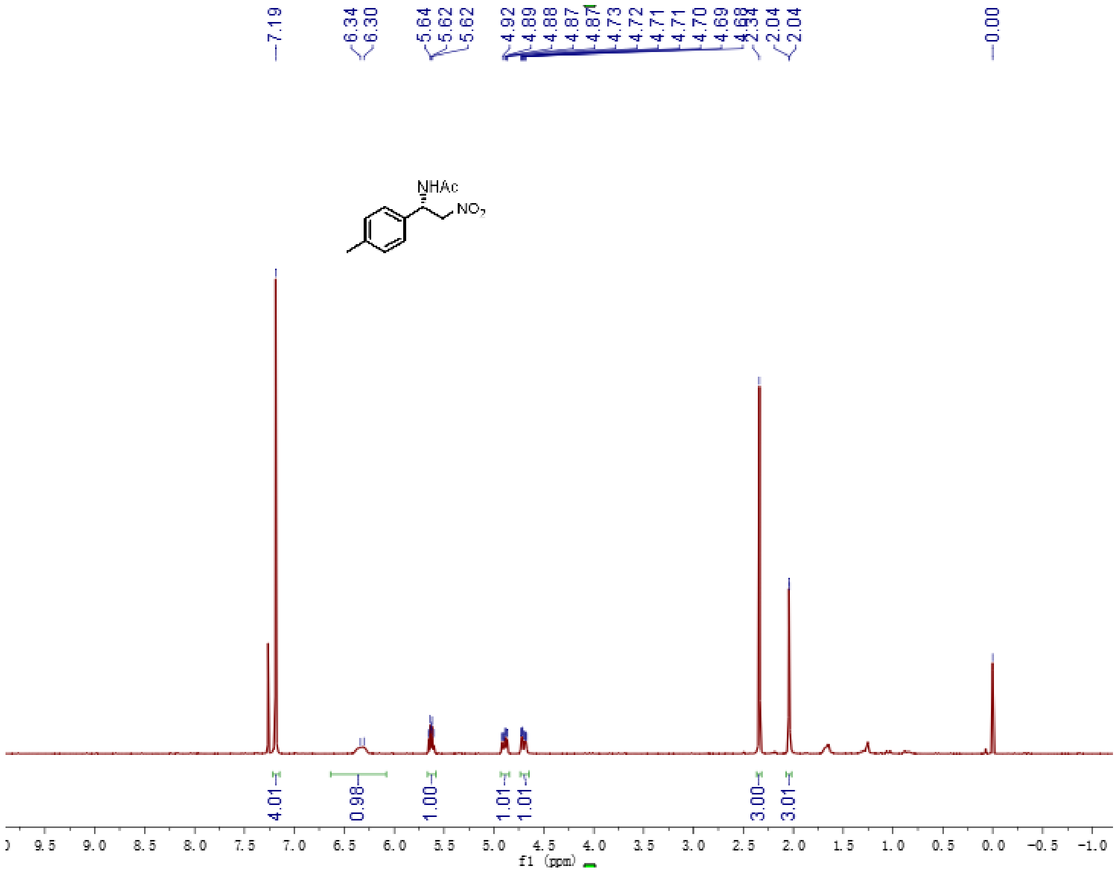


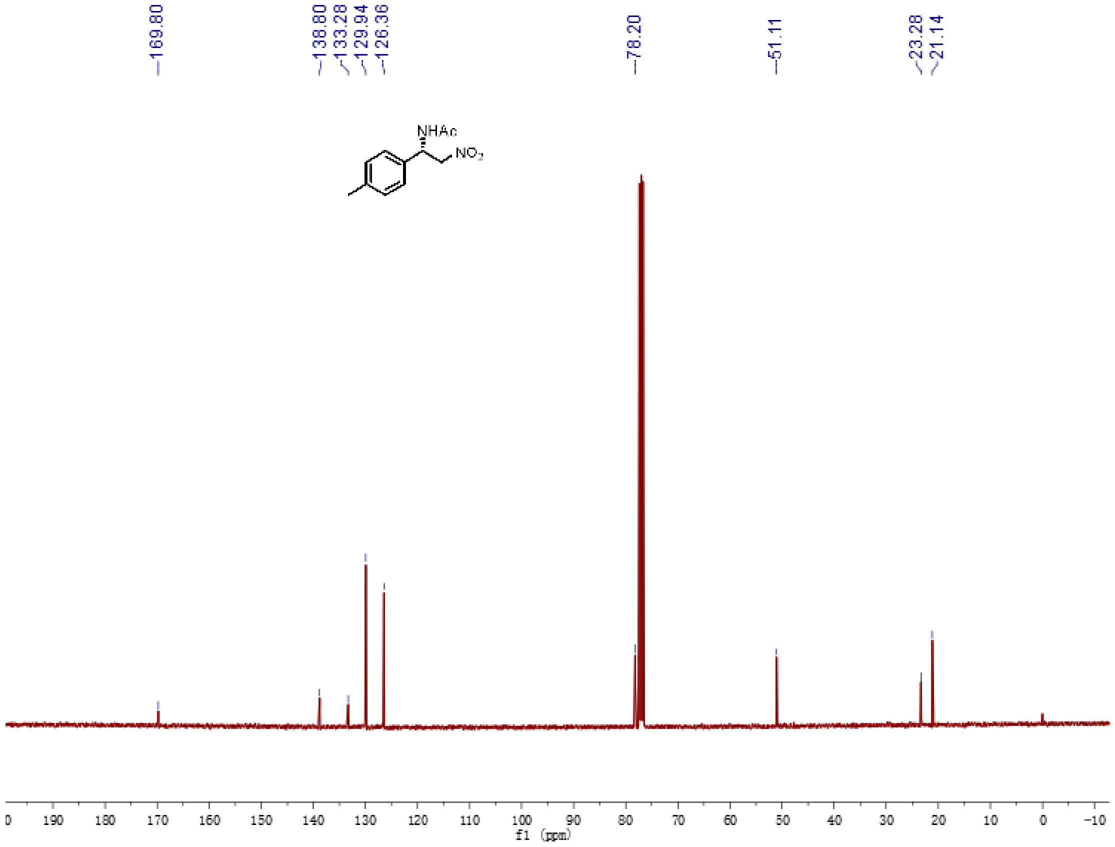

$^{1}$H NMR and $^{13}$C NMR of **2c**

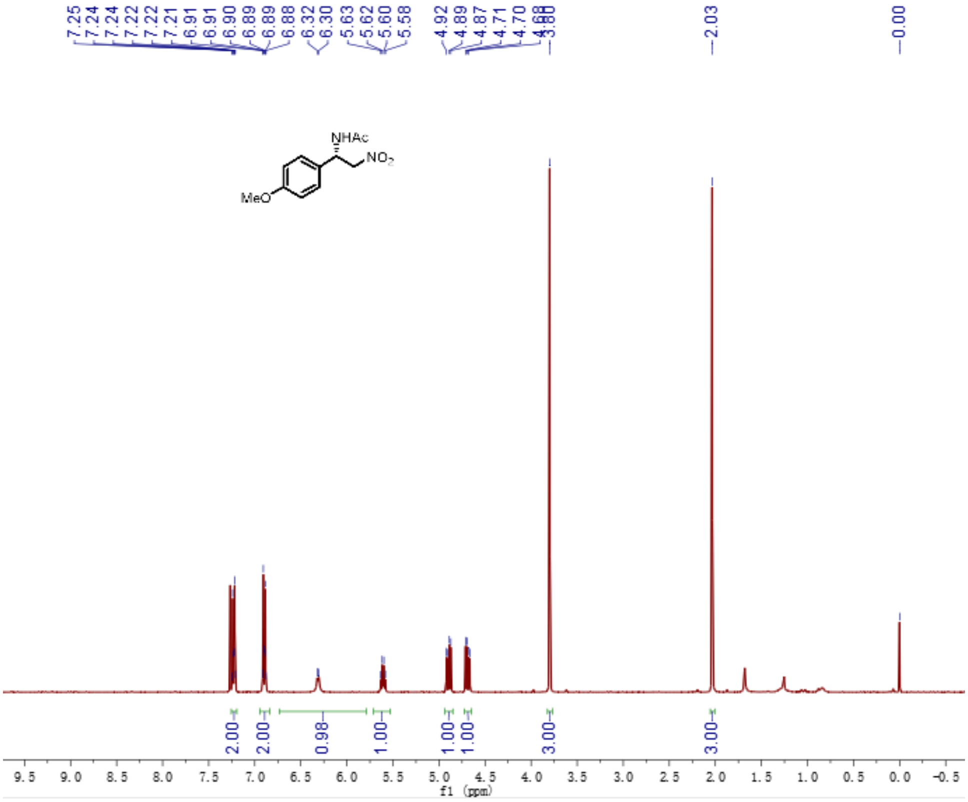


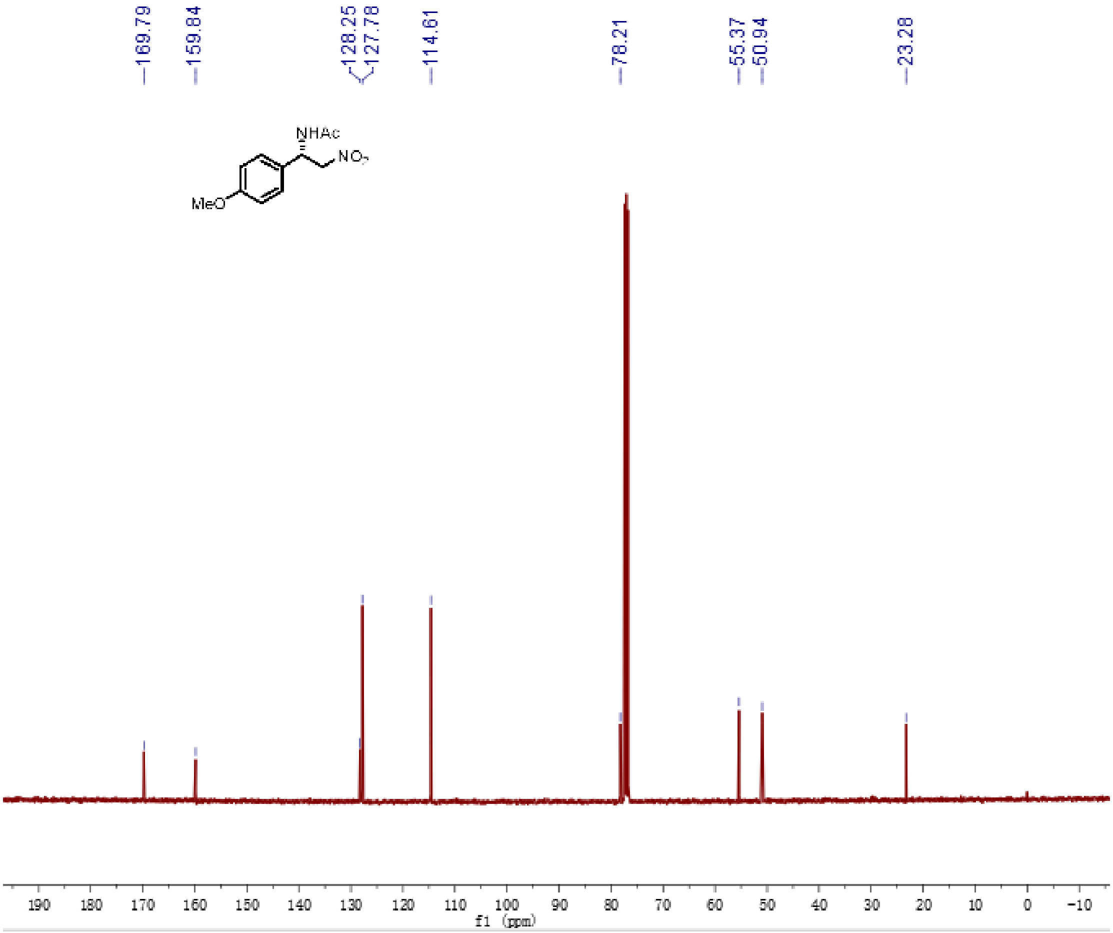

$^{1}$H NMR and $^{13}$C NMR of **2d**

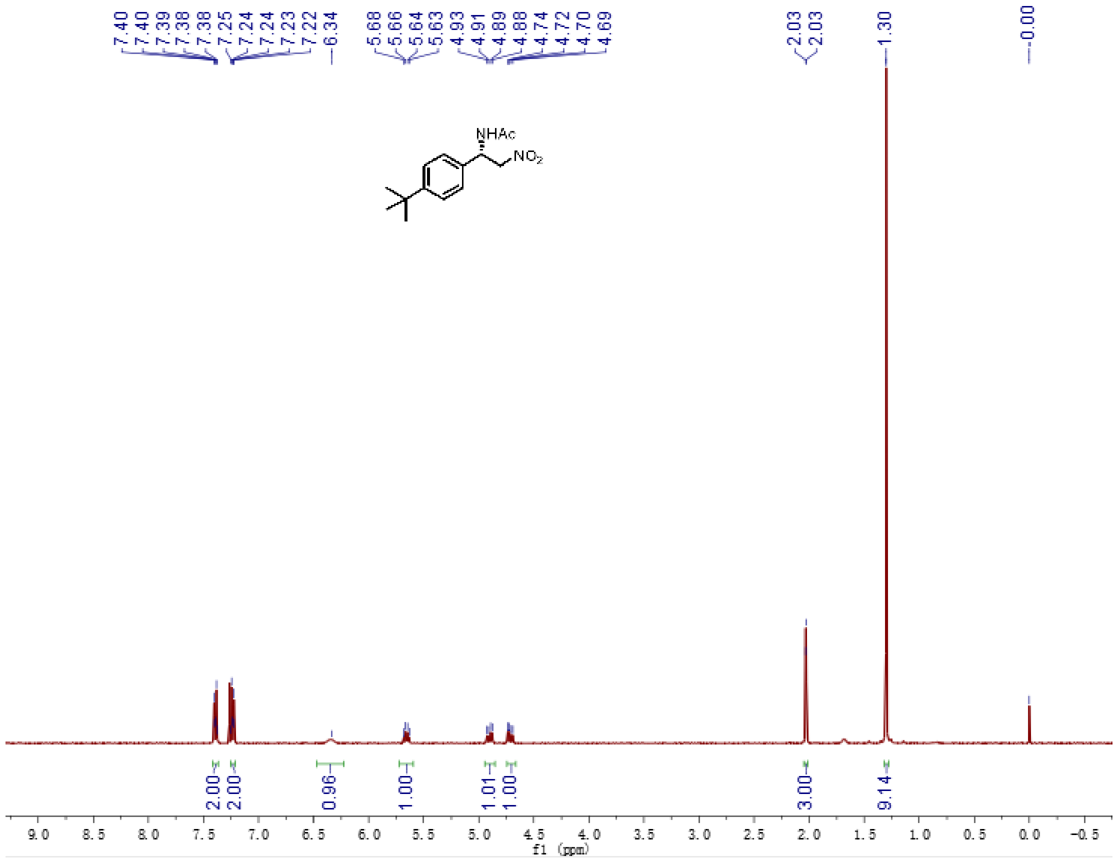

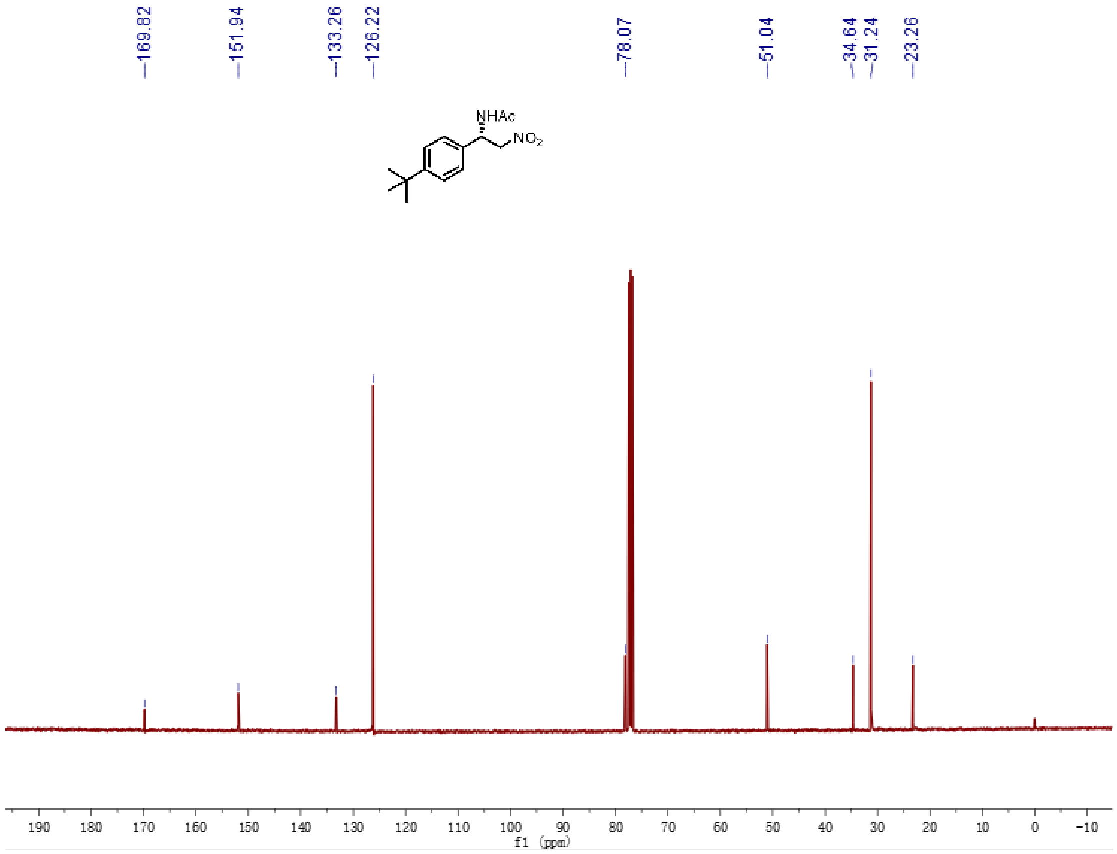

$^{1}H$ NMR and $^{13}C$ NMR of **2e**

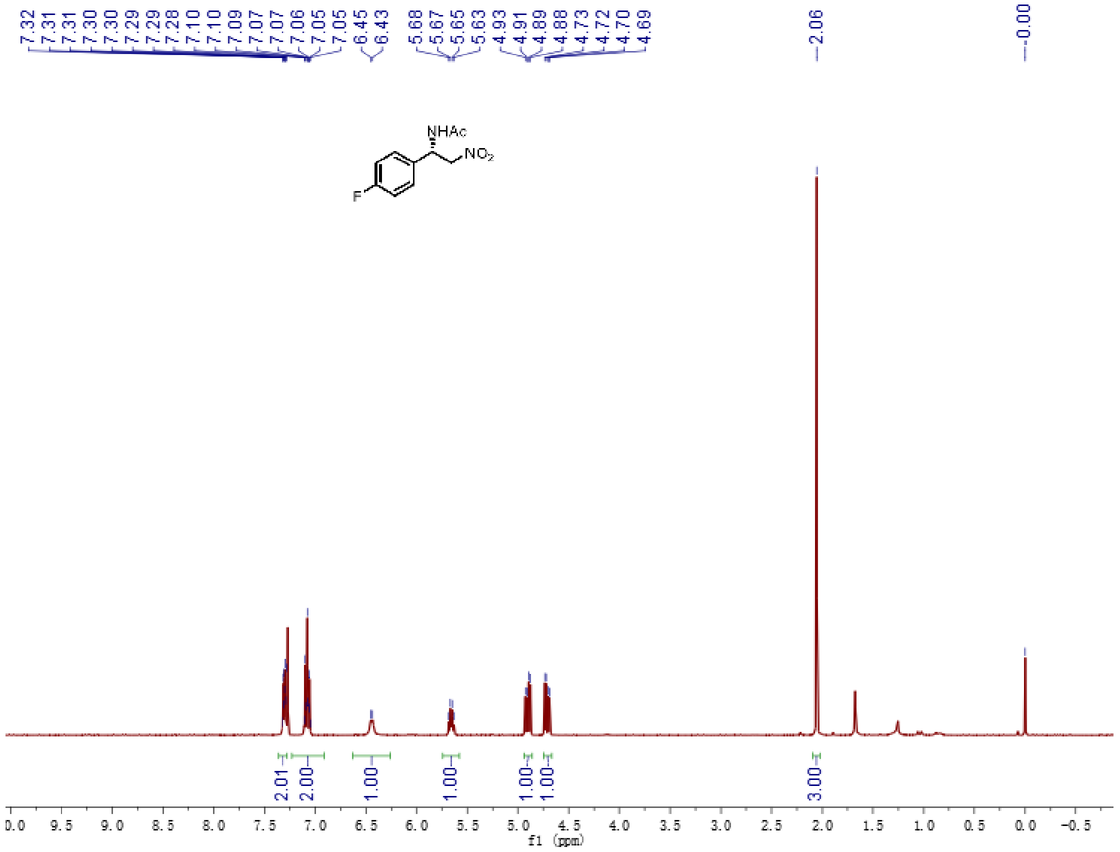

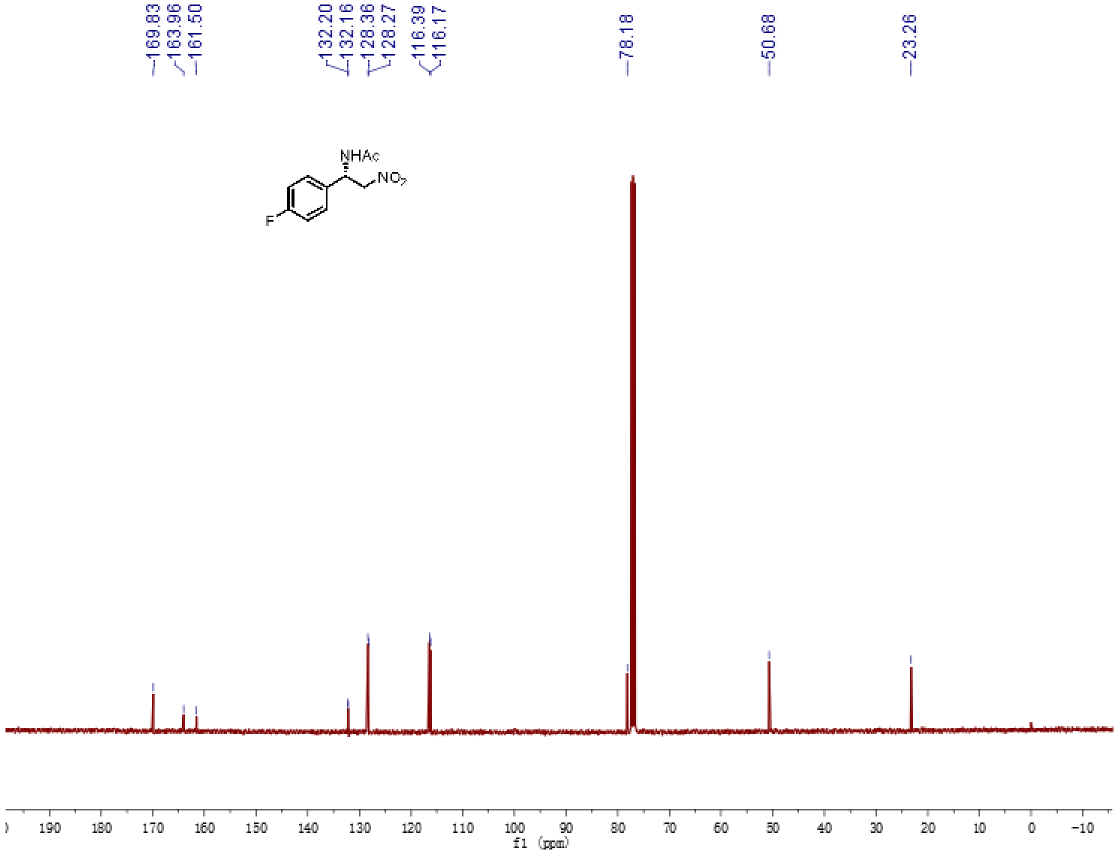

$^{1}$H NMR and $^{13}$C NMR of **2f**

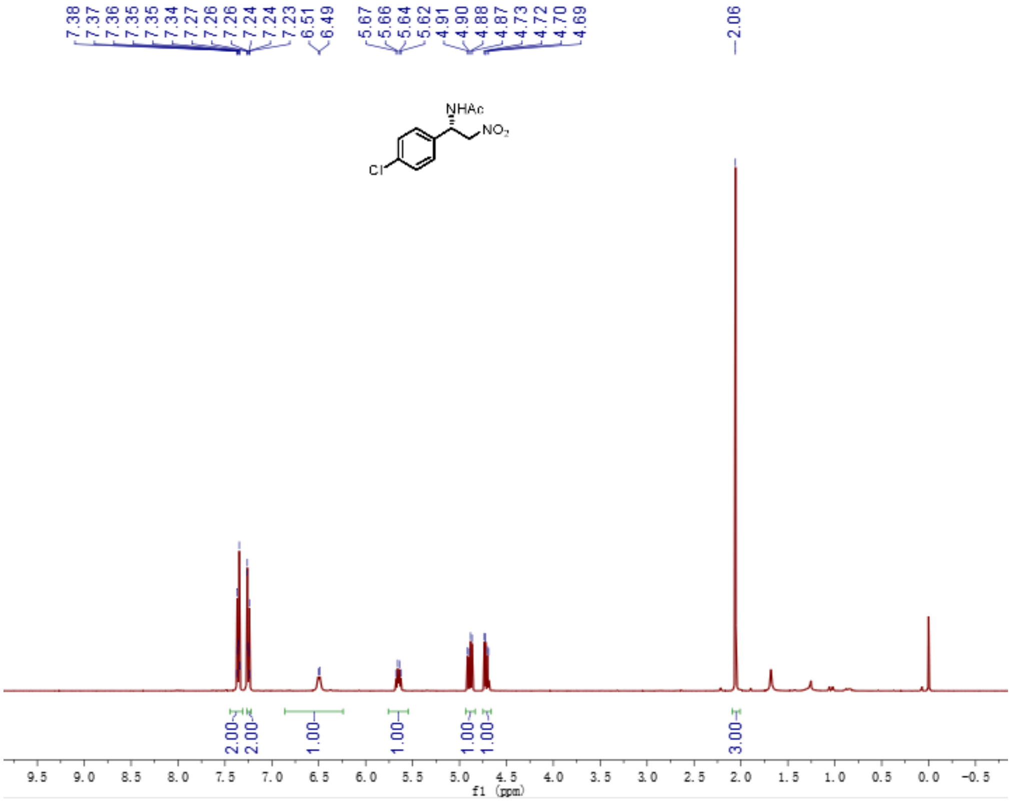

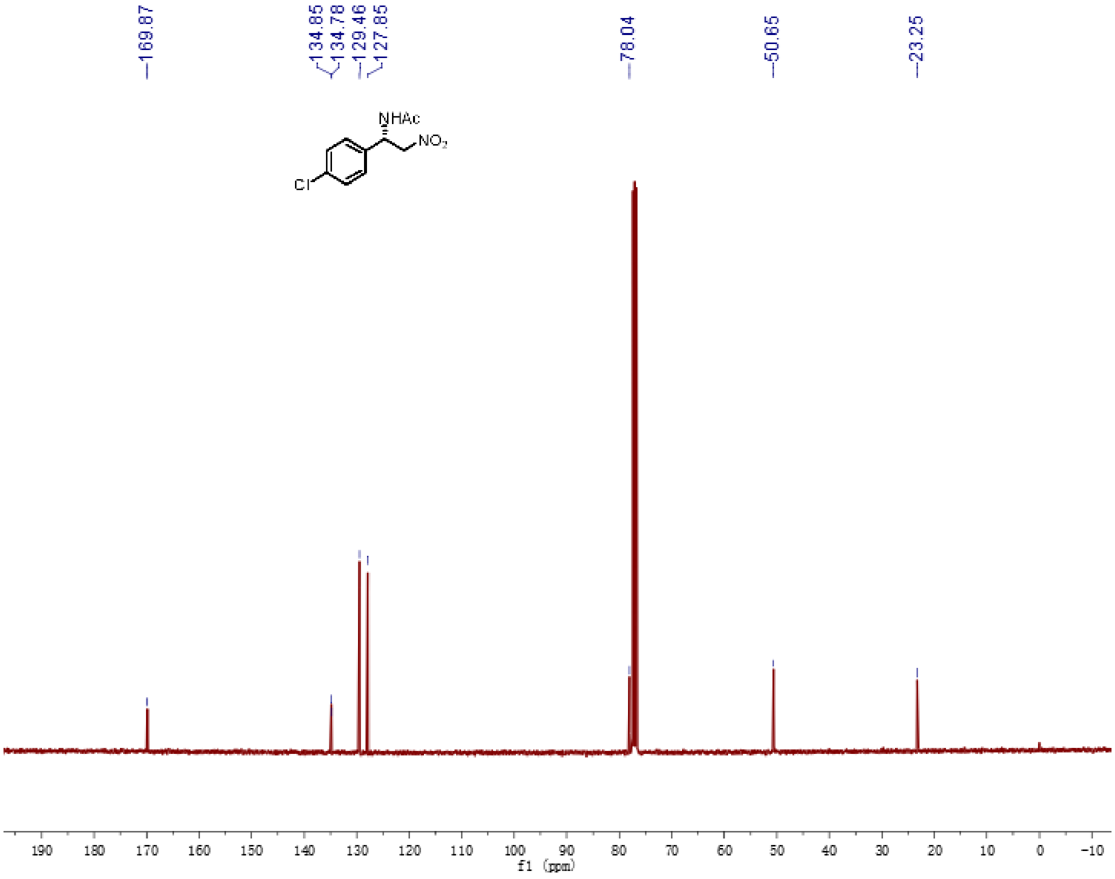

$^{1}H$ NMR and $^{13}C$ NMR of **2g**

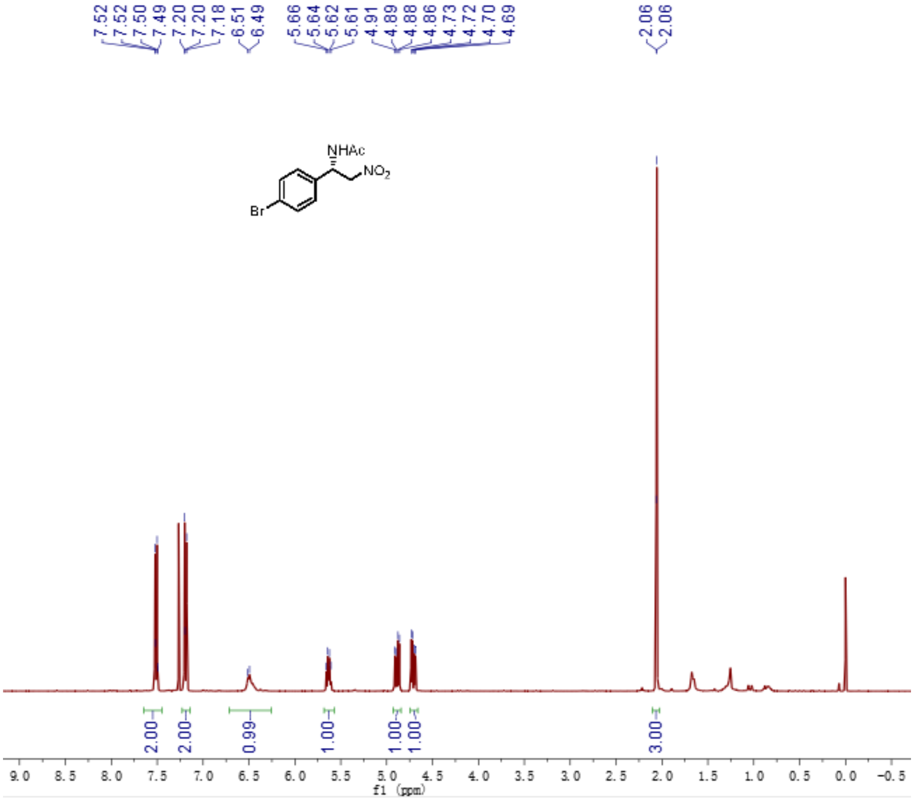


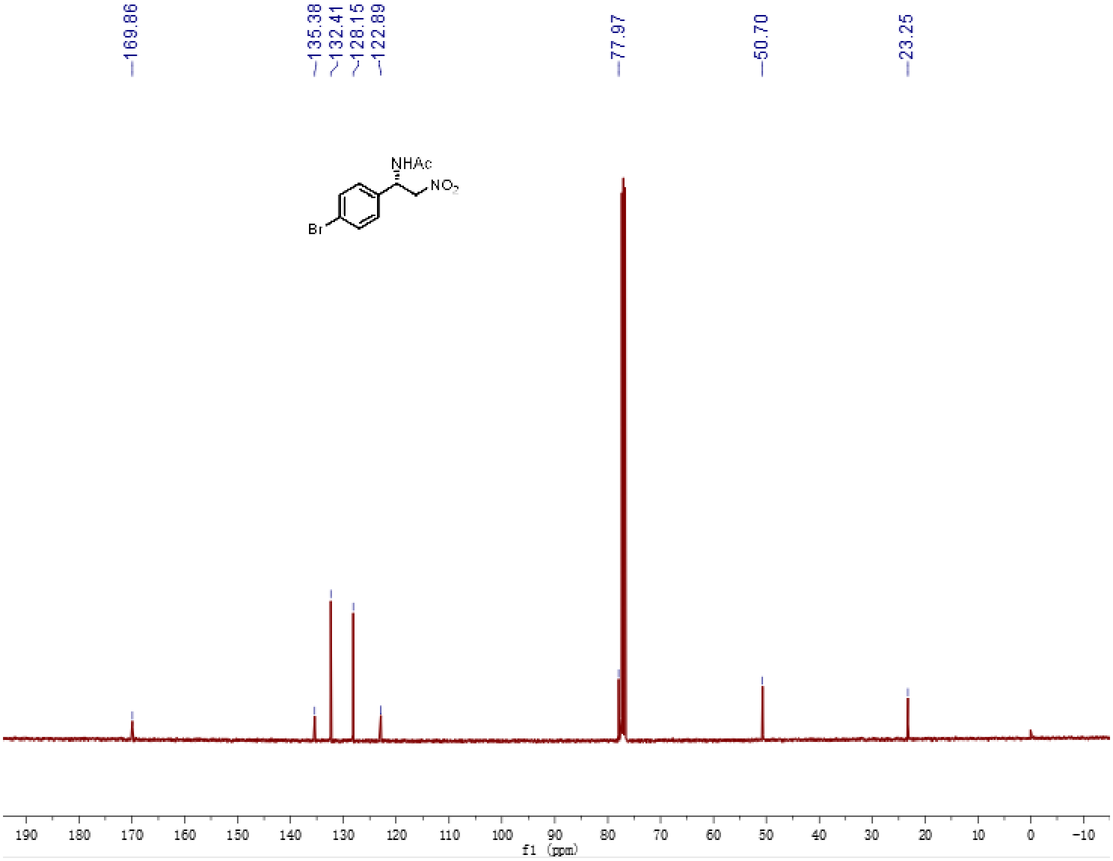

$^{1}H$ NMR and $^{13}C$ NMR of **2h**

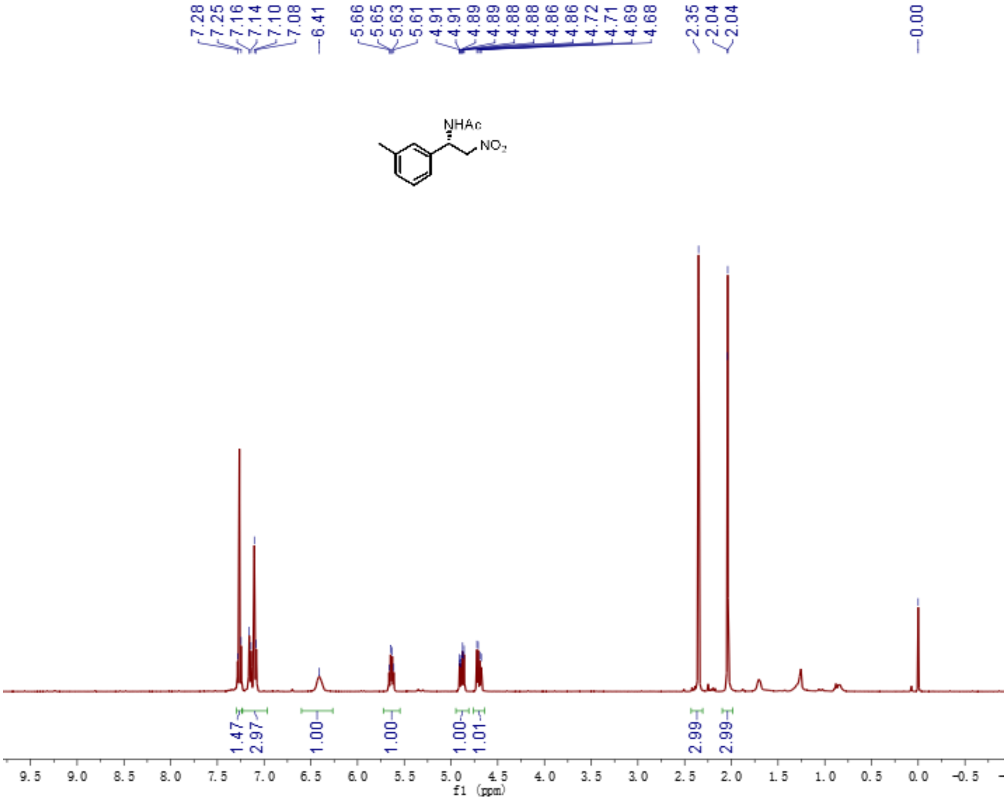


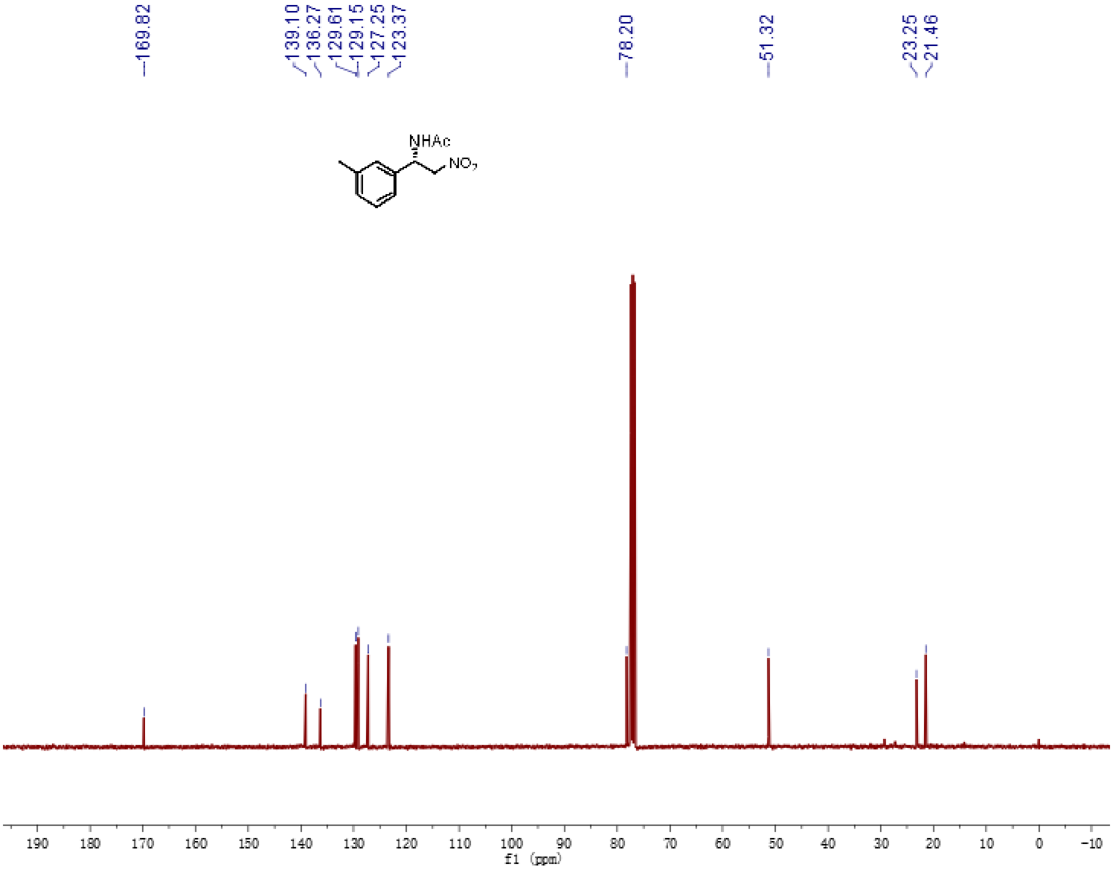

$^{1}H$ NMR and $^{13}C$ NMR of **2i**

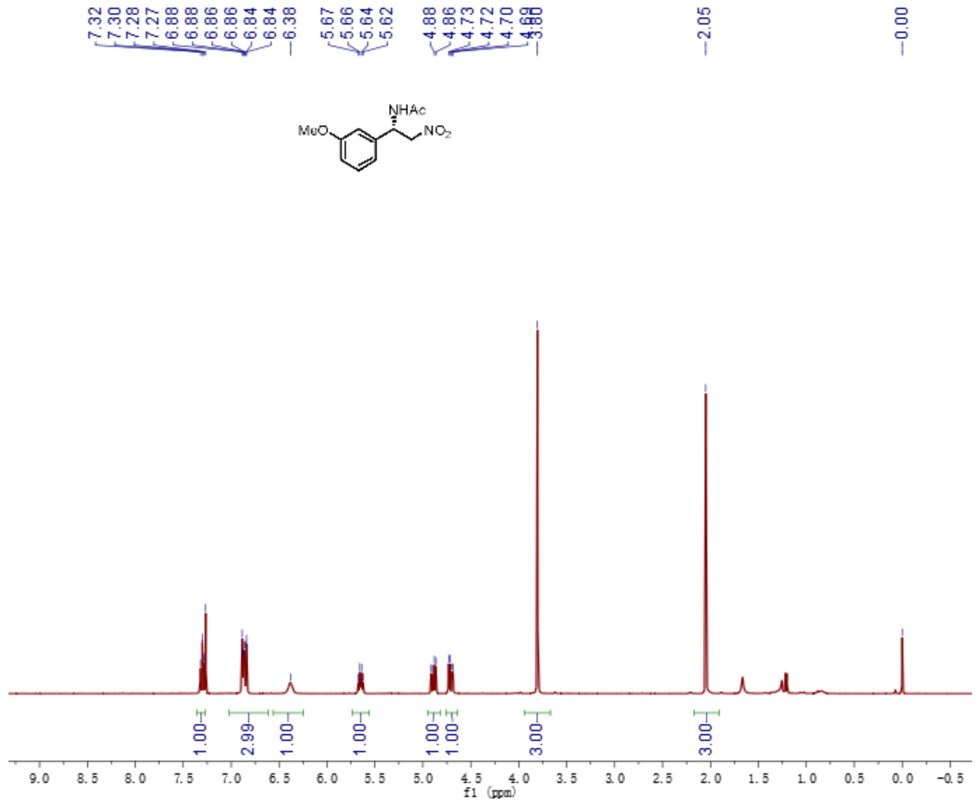


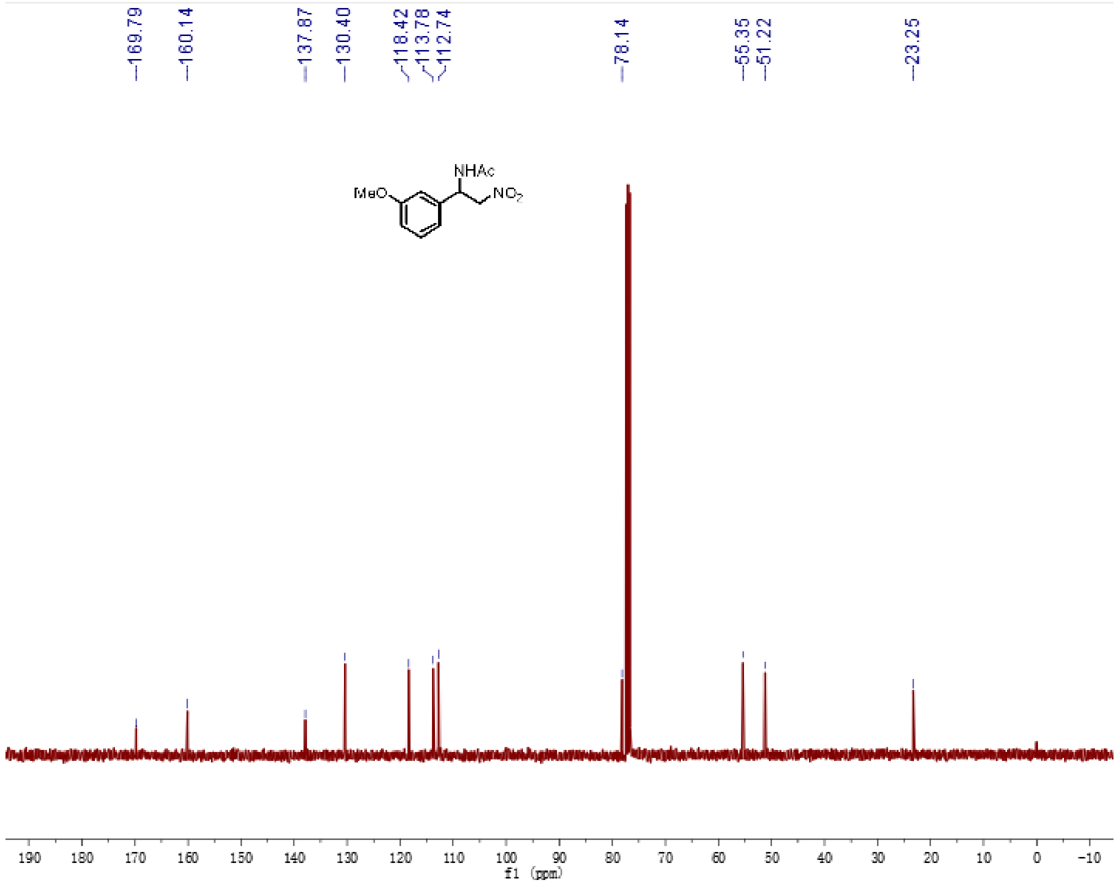

$^1$H NMR and $^{13}$C NMR of **2j**

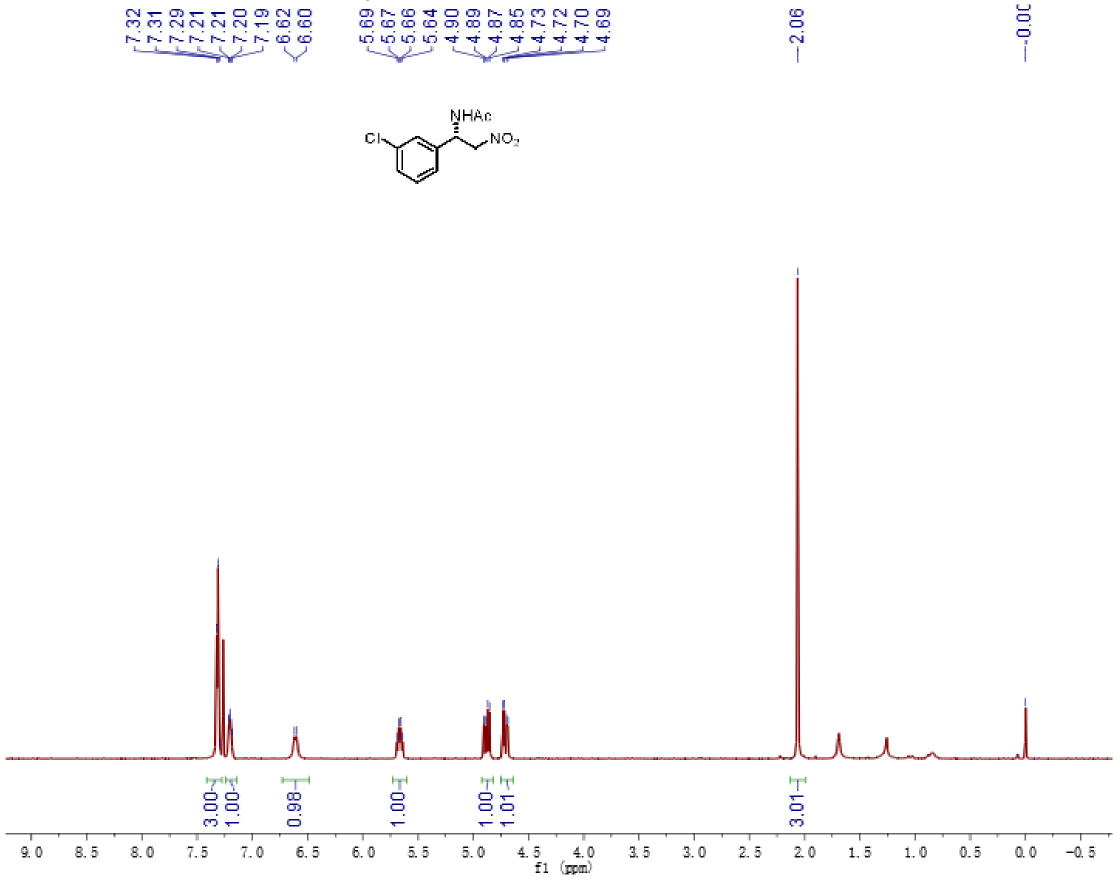


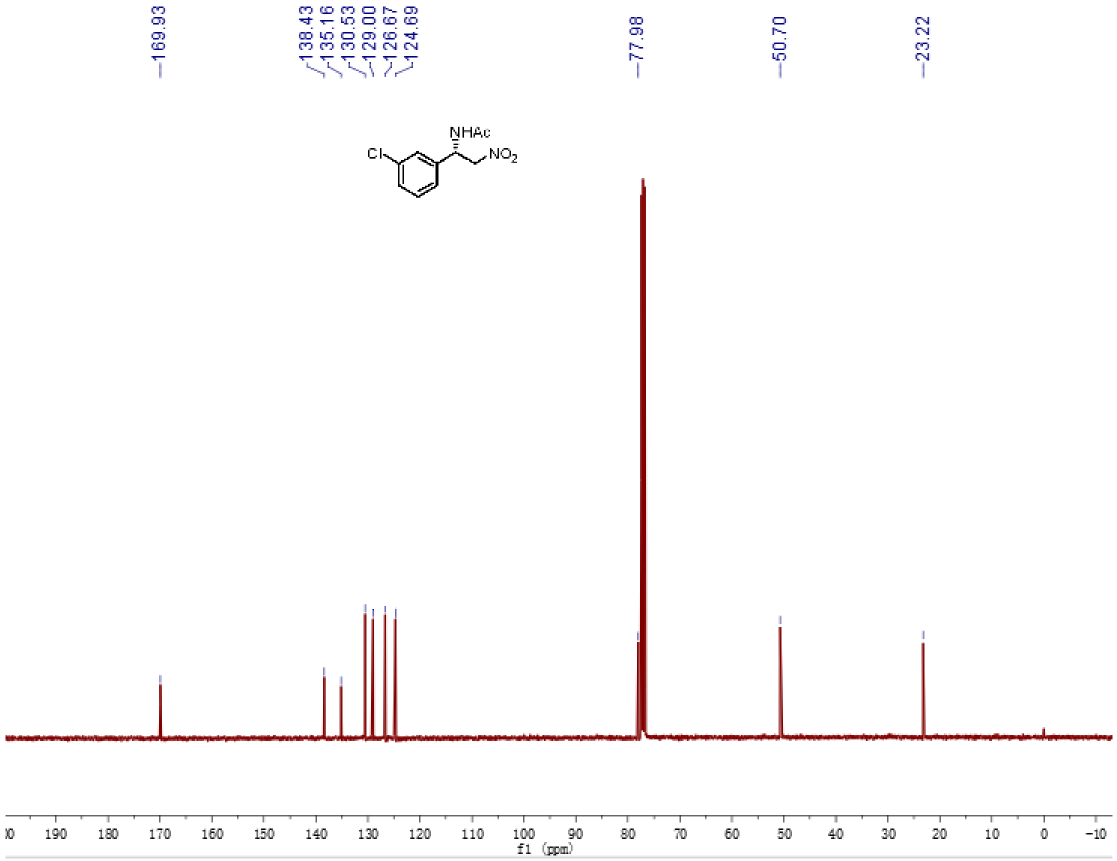

$^{1}$H NMR and $^{13}$C NMR of **2k**

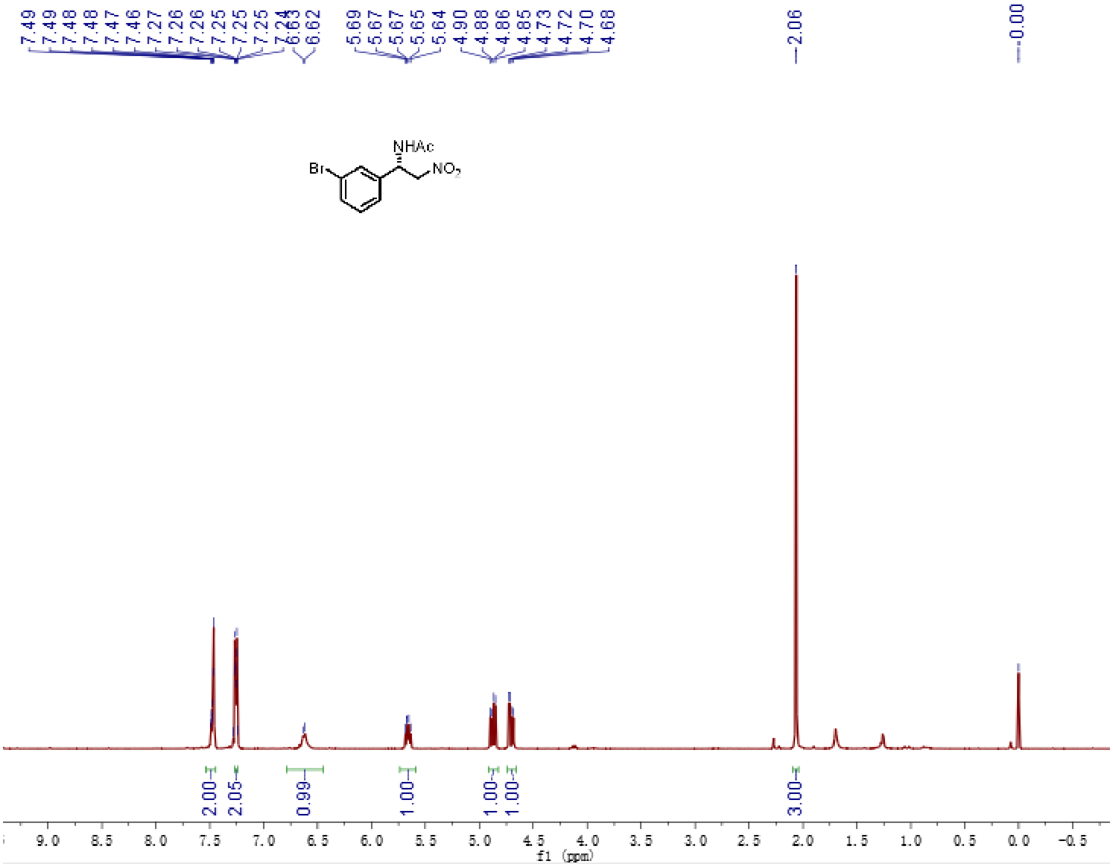

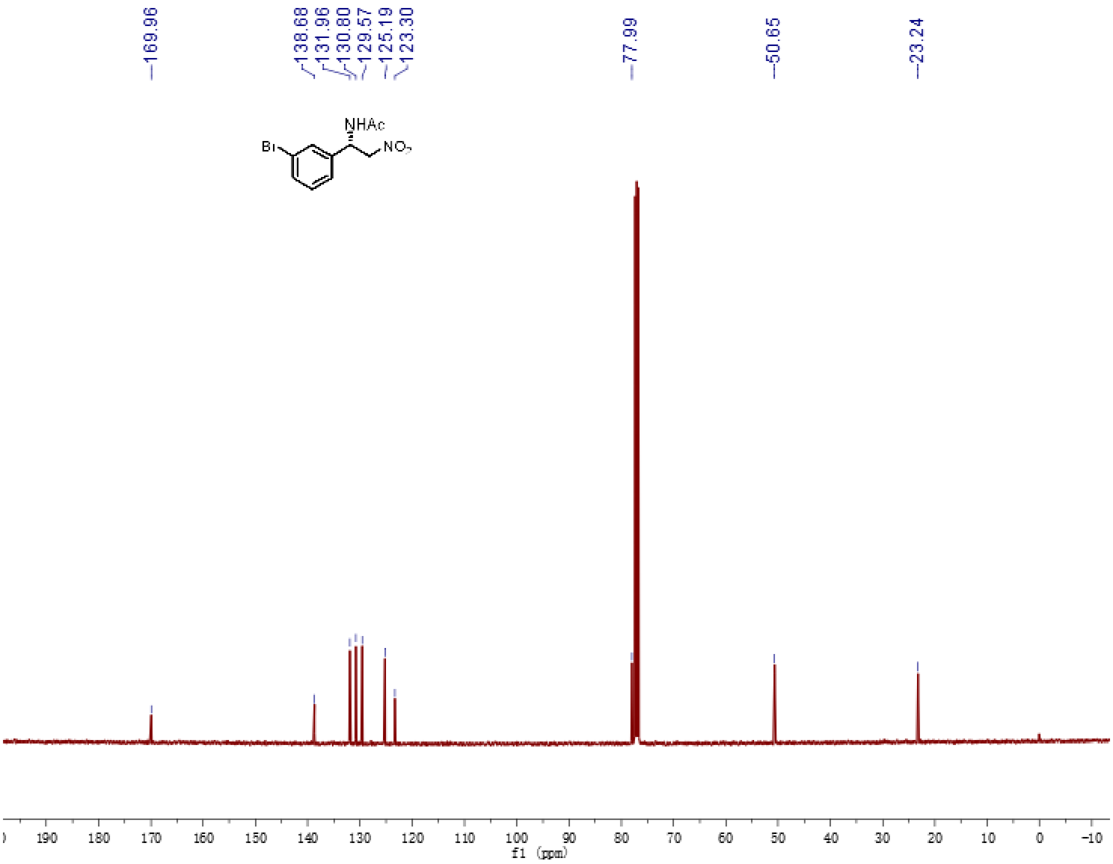

$^{1}$H NMR and $^{13}$C NMR of **2l**

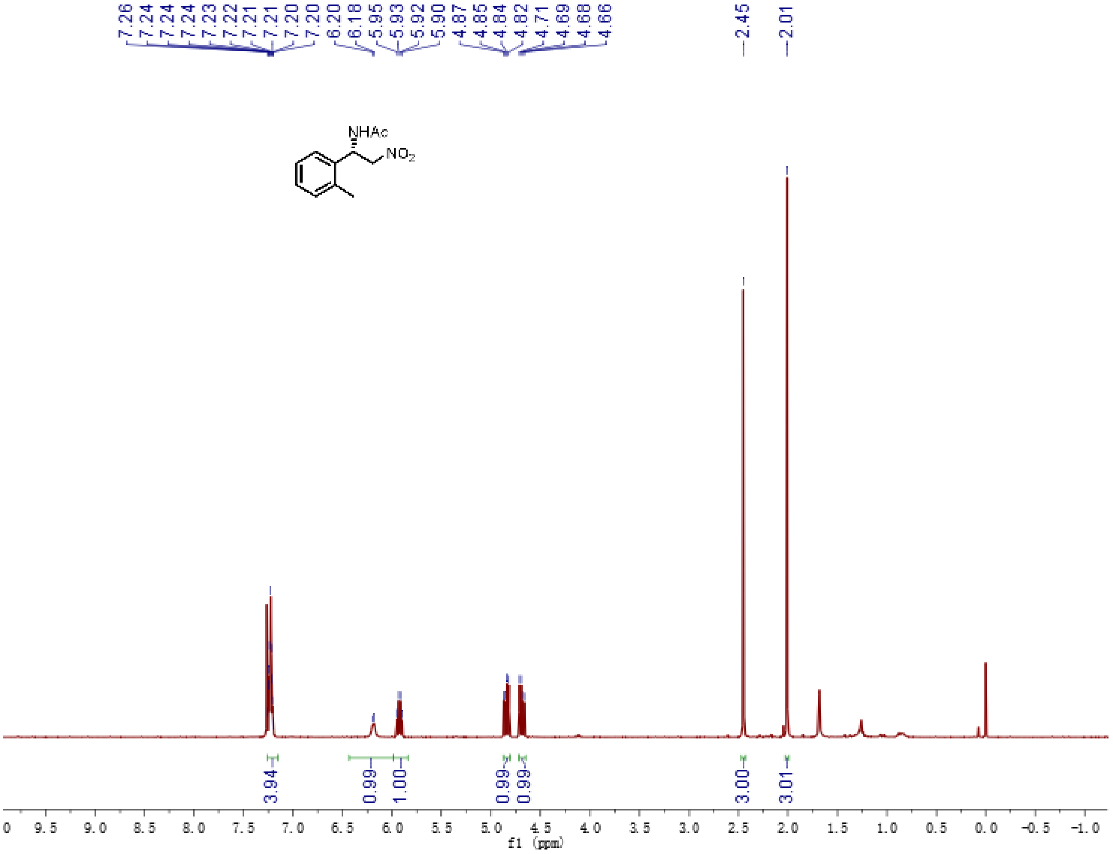


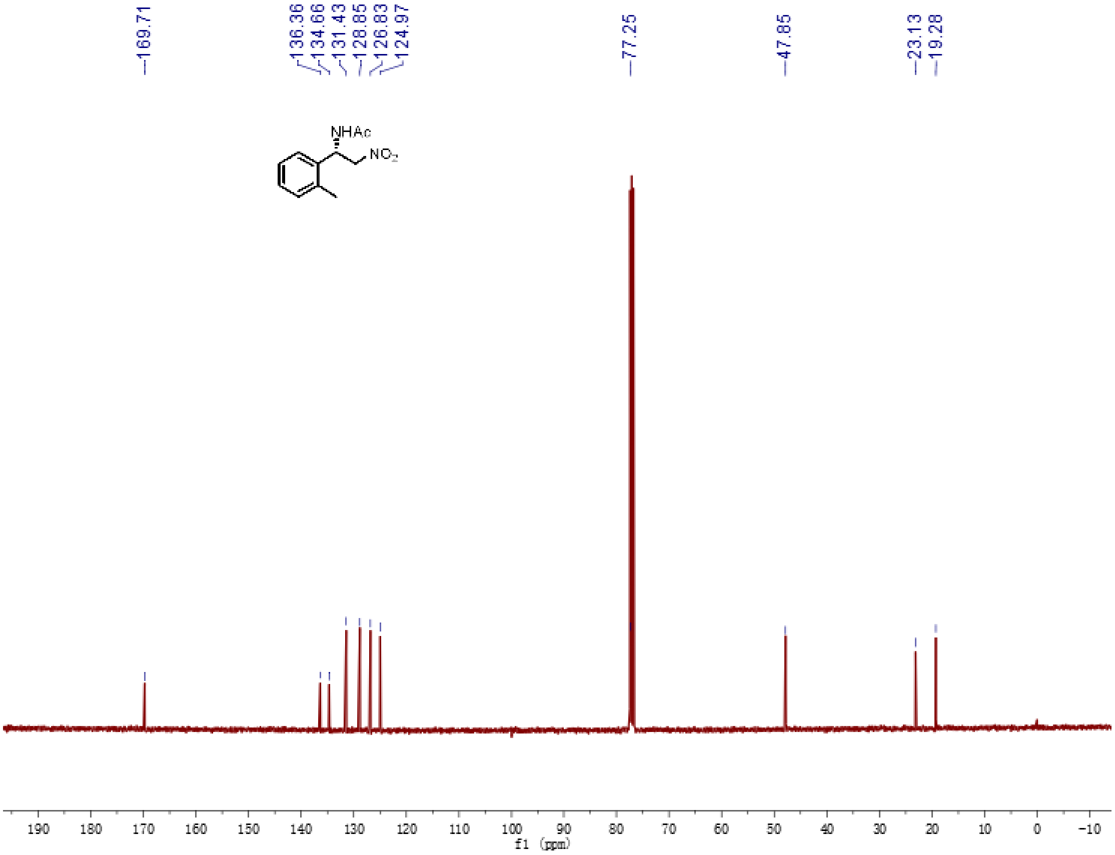

$^{1}$H NMR and $^{13}$C NMR of **2m**

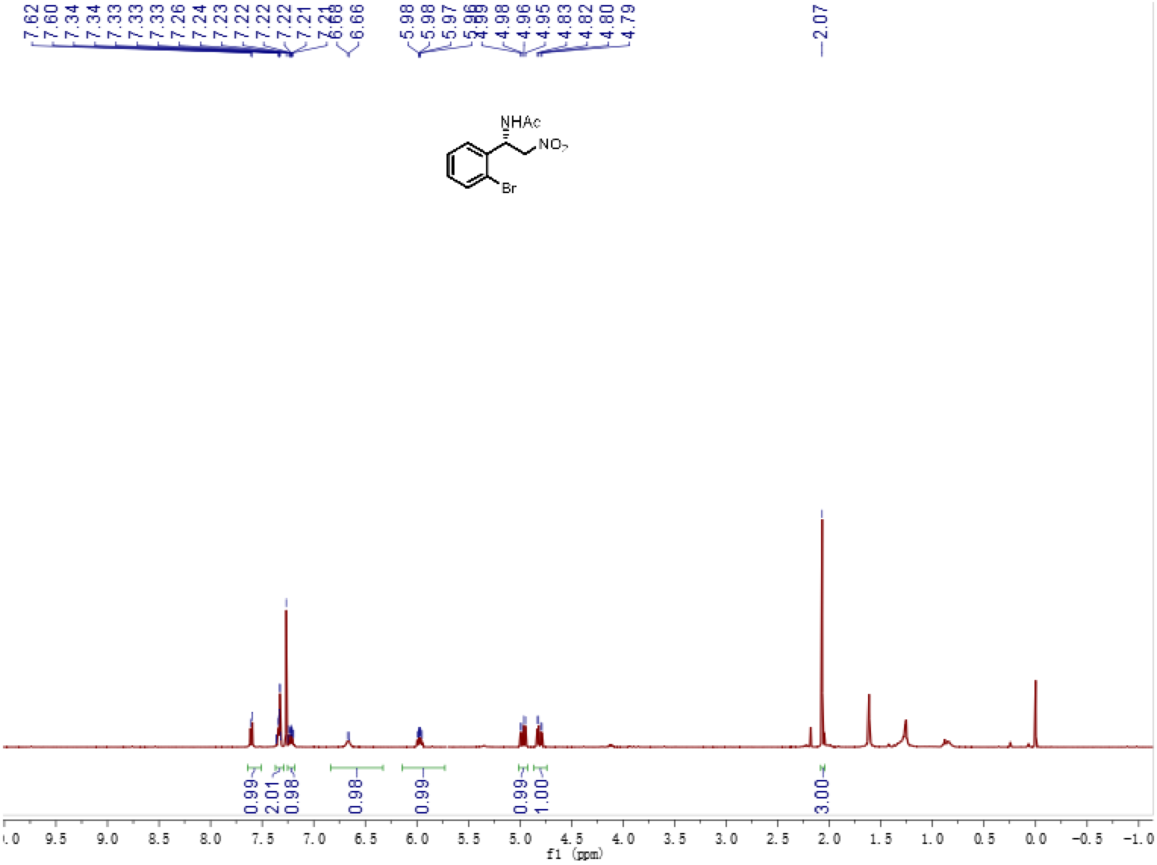


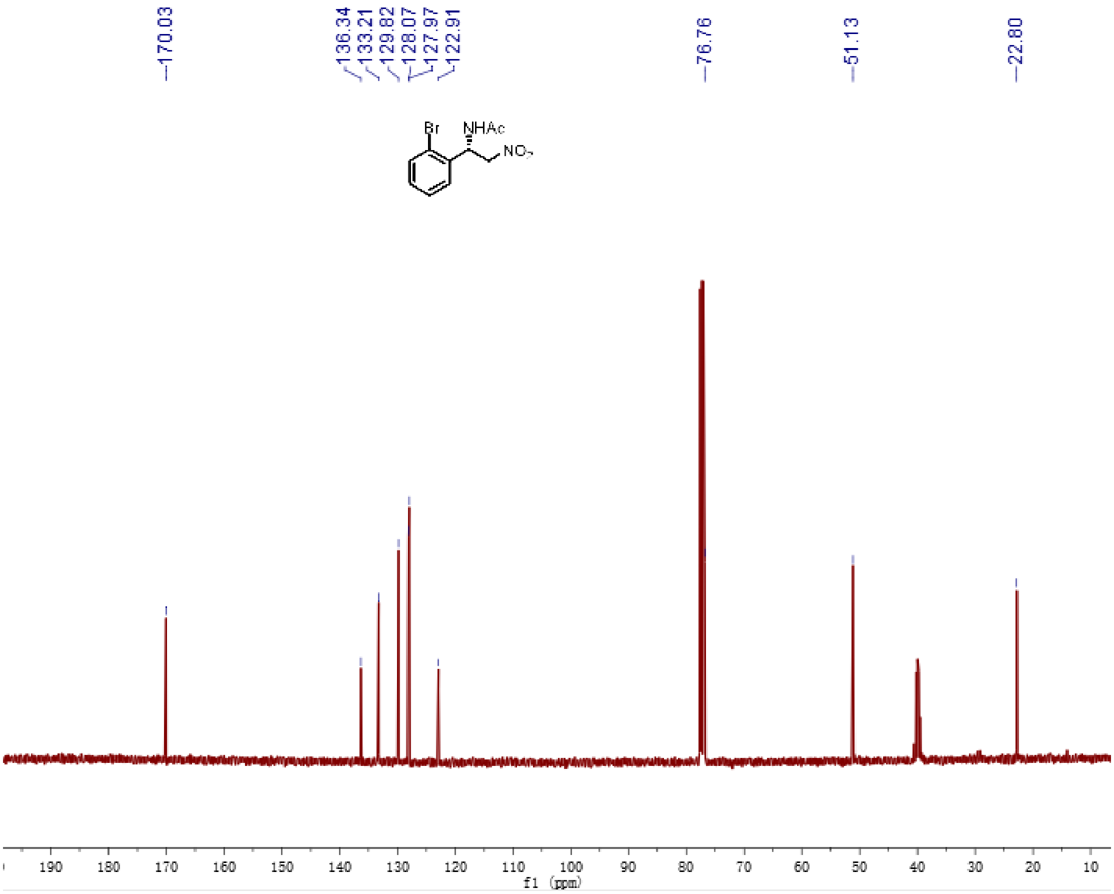

$^{1}$H NMR and $^{13}$C NMR of **2n**

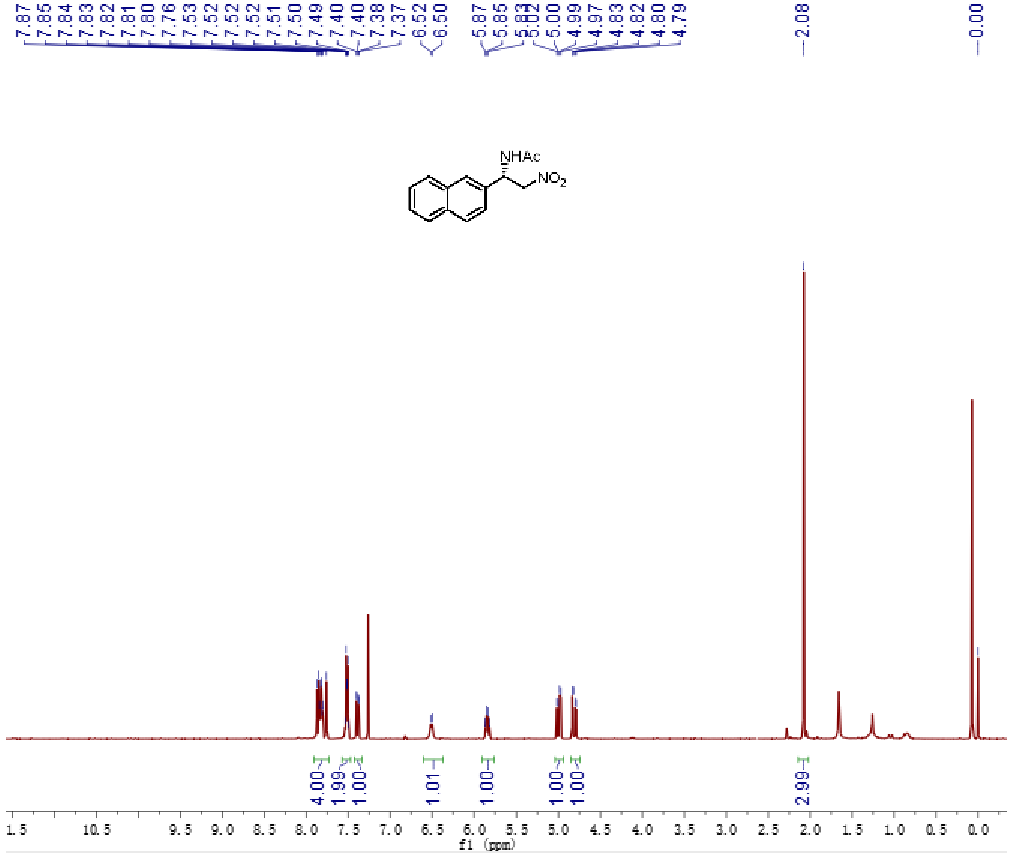


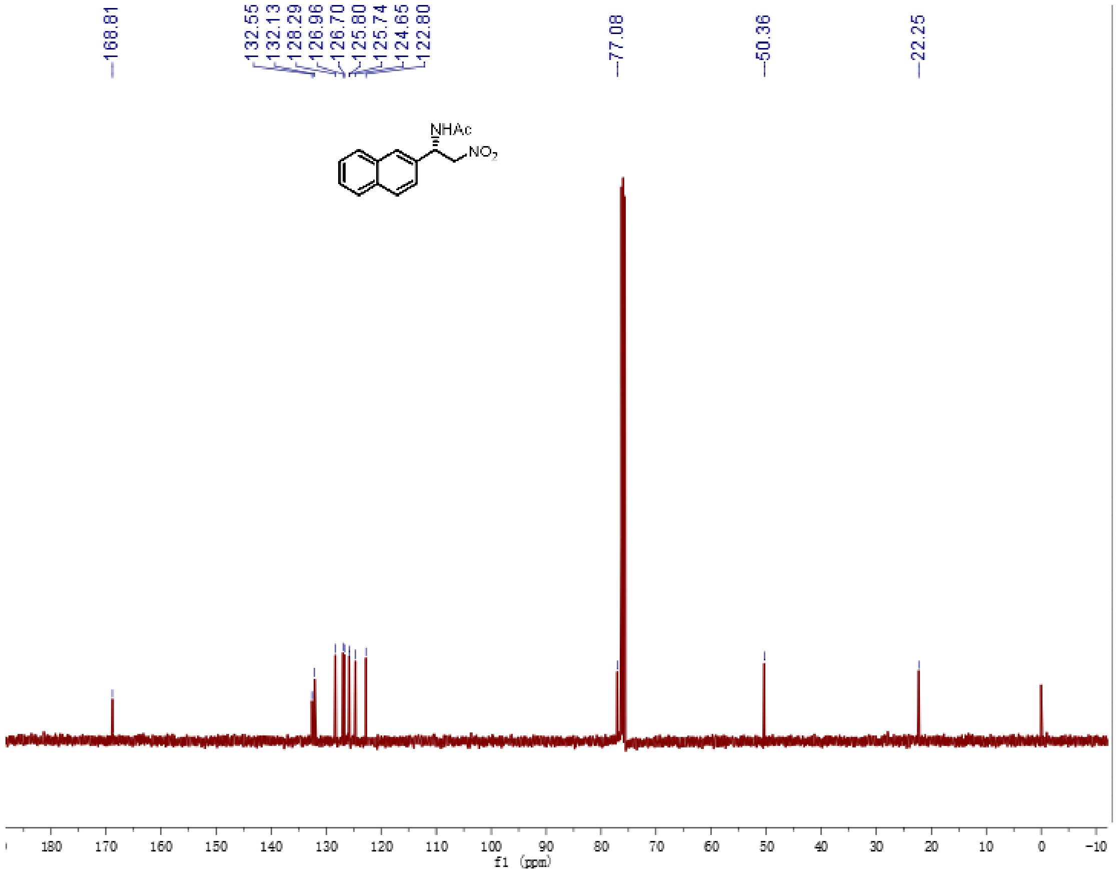

$^{1}$H NMR and $^{13}$C NMR of **2o**

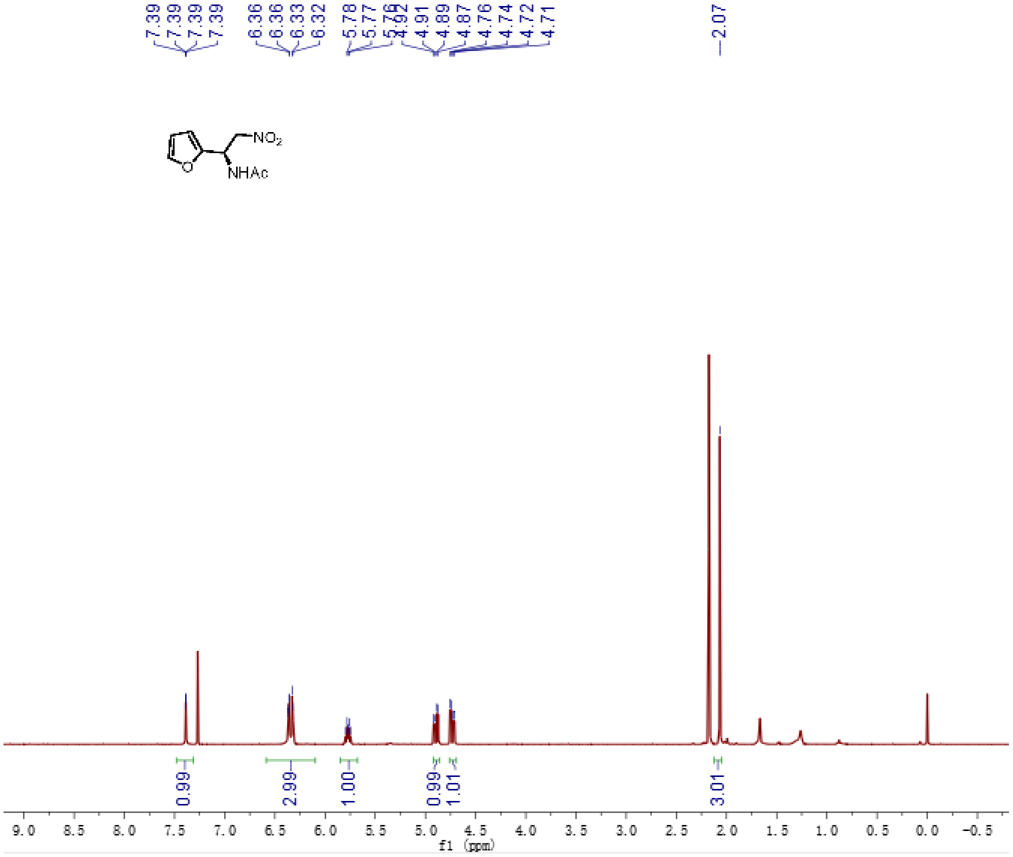


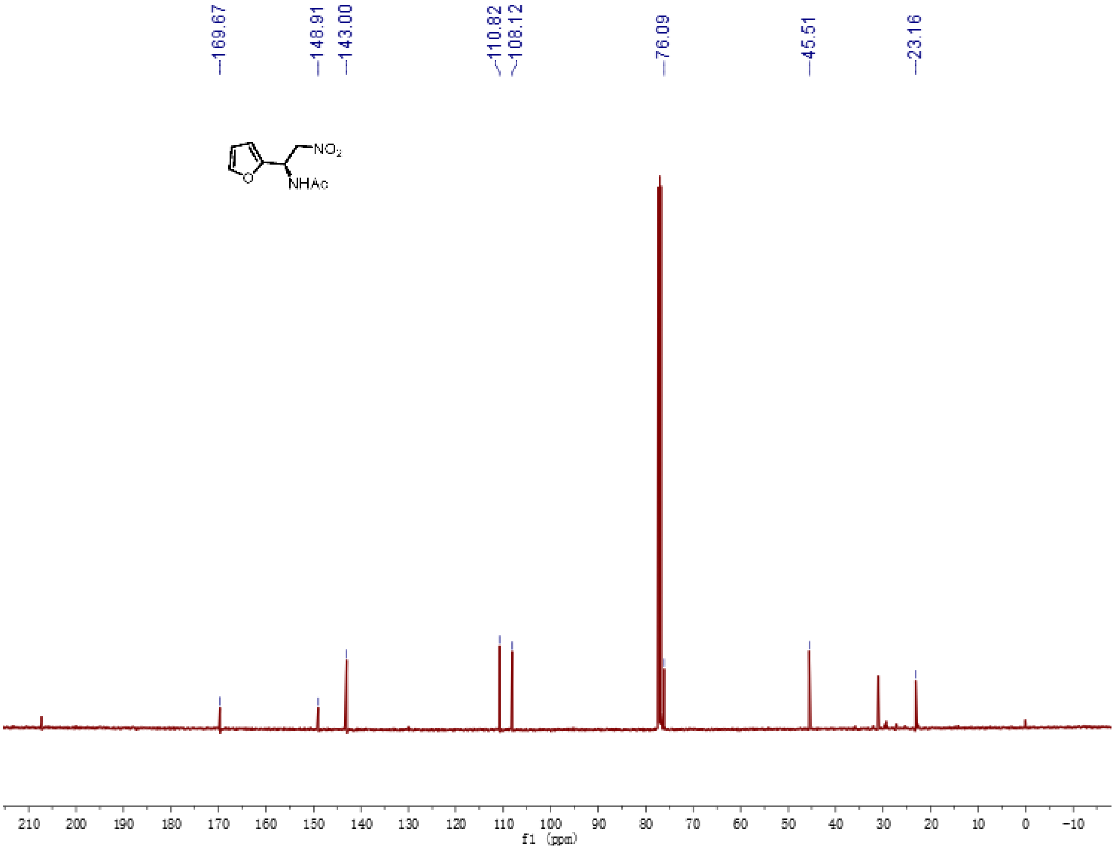

$^1$H NMR and $^{13}$C NMR of **2p**

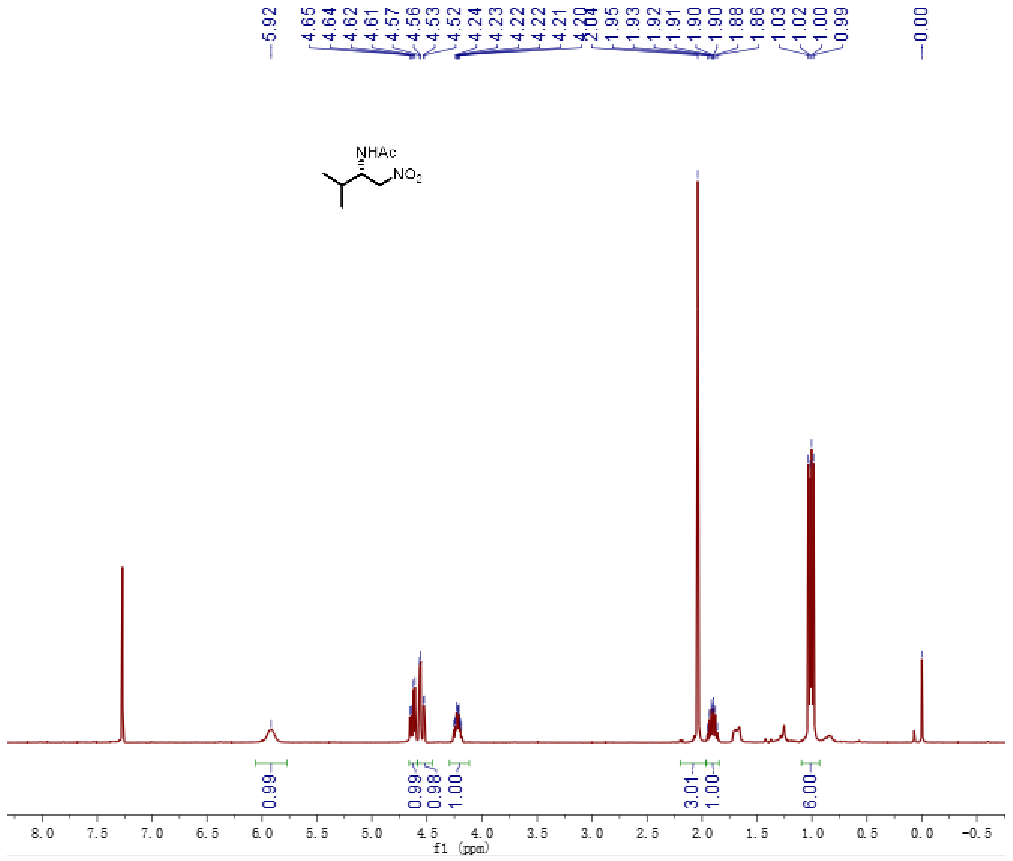


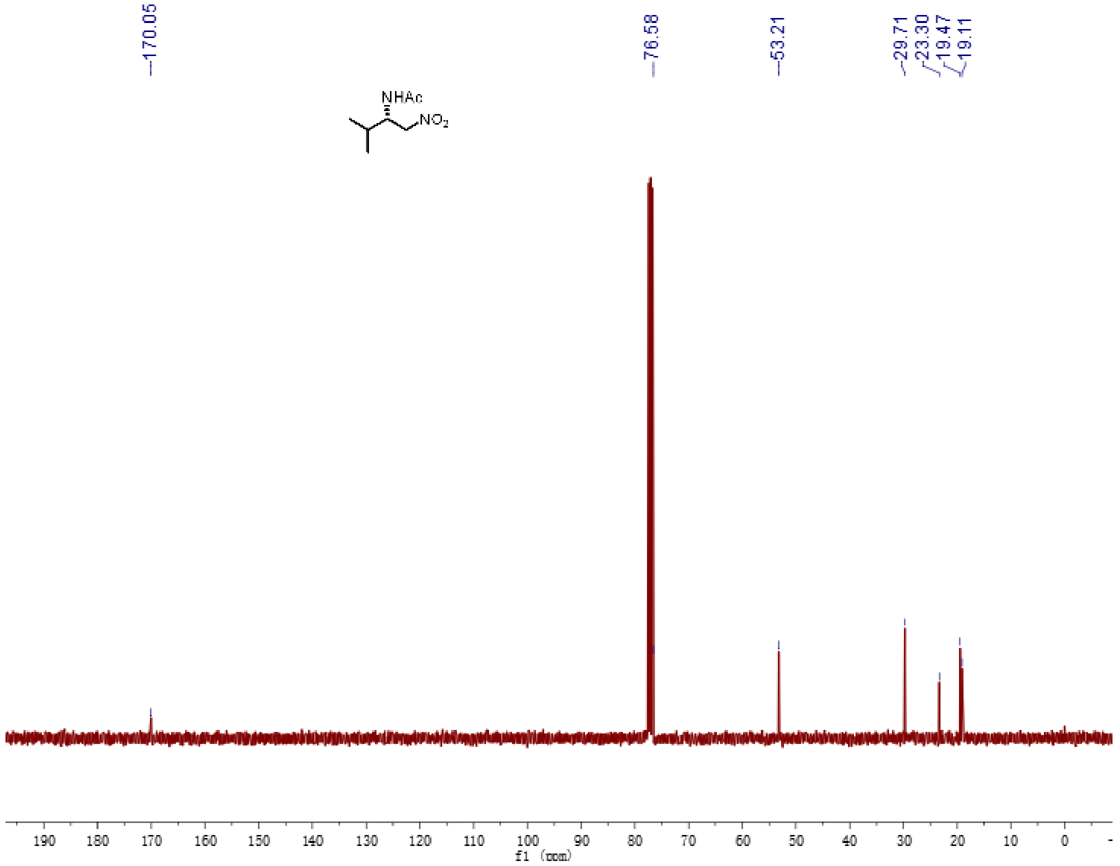

# $^{1}$H NMR and $^{13}$C NMR of **2q**

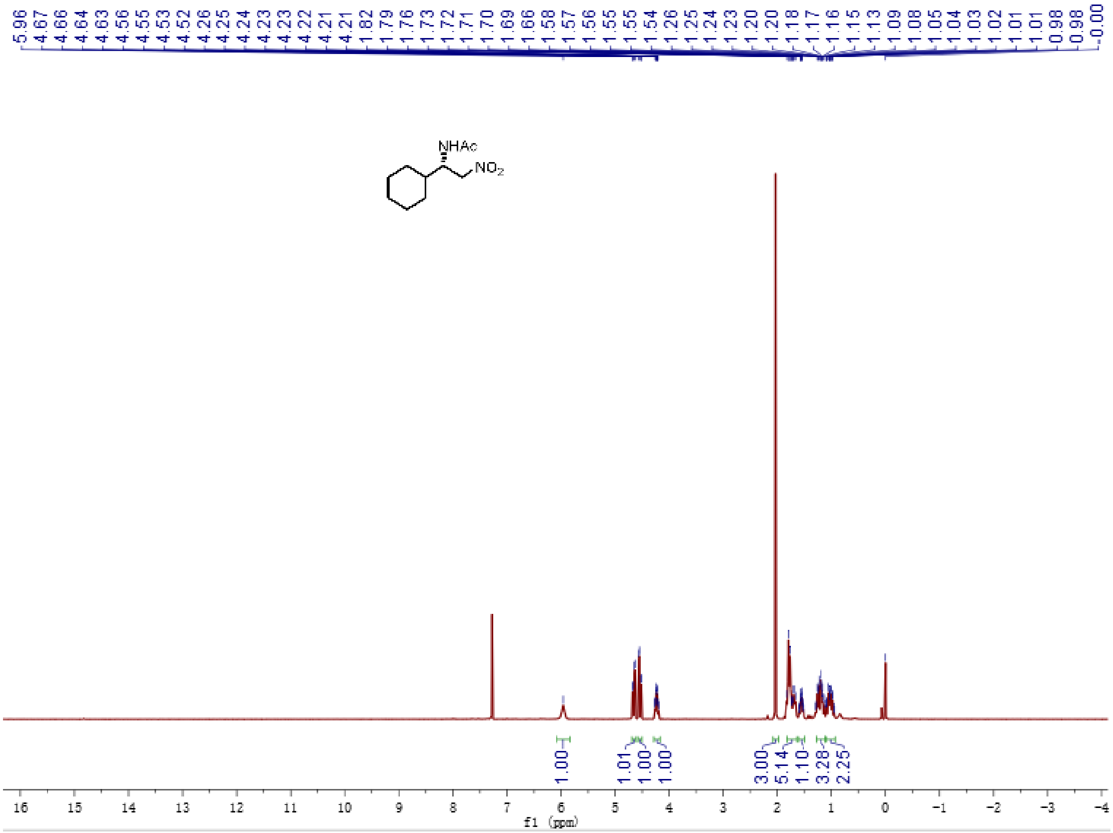


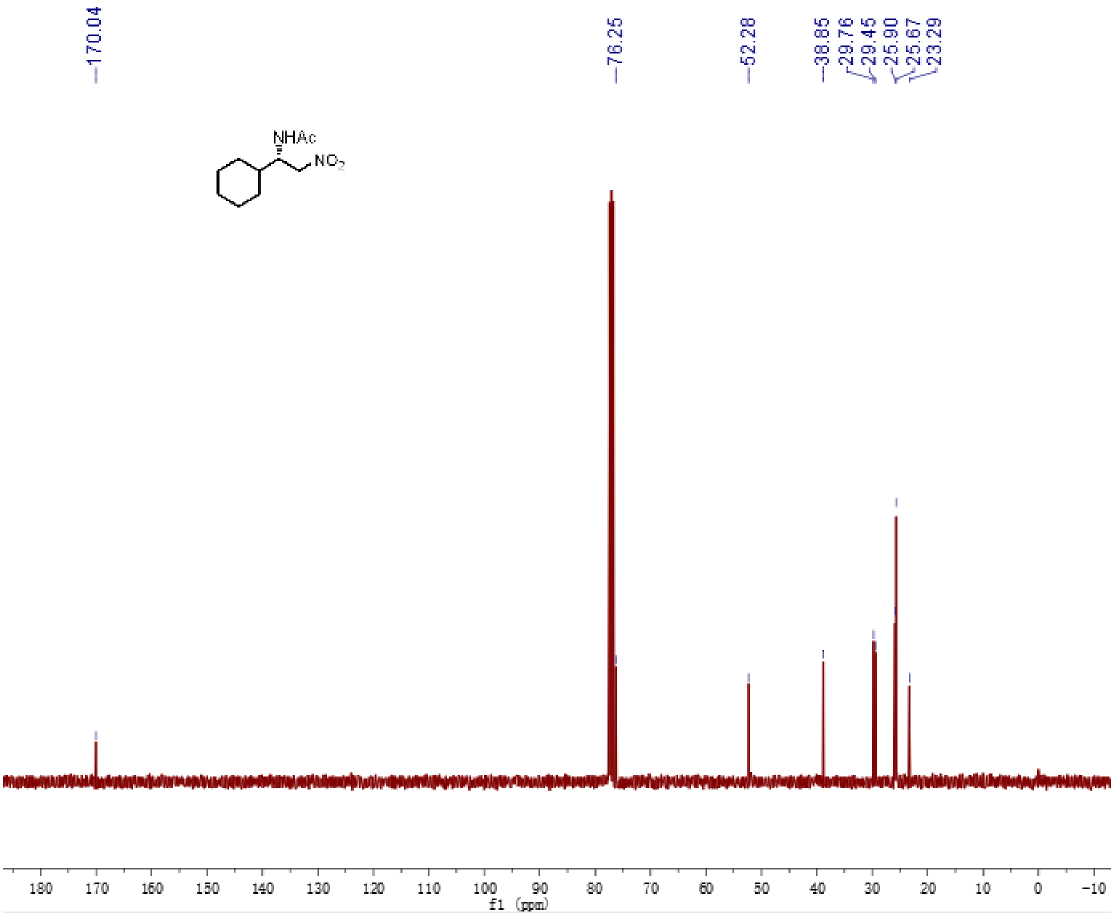

HPLC-2a-**rac**

```
Data File E:\DATA\GWC\GWC16-9\SUBSTRATE- SCOPE 2016-09-23 07-58-46\001-0301.D
Sample Name: biao-rac

    =====================================================================
    Acq. Operator   : SYSTEM                          Seq. Line :   3
    Acq. Instrument : 1260HPLC-VWD                     Location : Vial 1
    Injection Date  : 9/23/2016 8:36:04 AM                  Inj :   1
                                                     Inj Volume : 5.000 µl
    Acq. Method     : E:\DATA\GWC\GWC16-9\SUBSTRATE- SCOPE 2016-09-23 07-58-46\VWD-ADH(1-6)-90
                      -10-210NM-40MIN.M
    Last changed    : 9/23/2016 8:30:50 AM by SYSTEM
    Analysis Method : E:\DATA\GWC\GWC16-9\SUBSTRATE- SCOPE 2016-09-23 07-58-46\VWD-ADH(1-6)-90
                      -10-210NM-40MIN.M (Sequence Method)
    Last changed    : 10/25/2016 7:59:19 PM by SYSTEM
                      (modified after loading)
    Additional Info : Peak(s) manually integrated
```

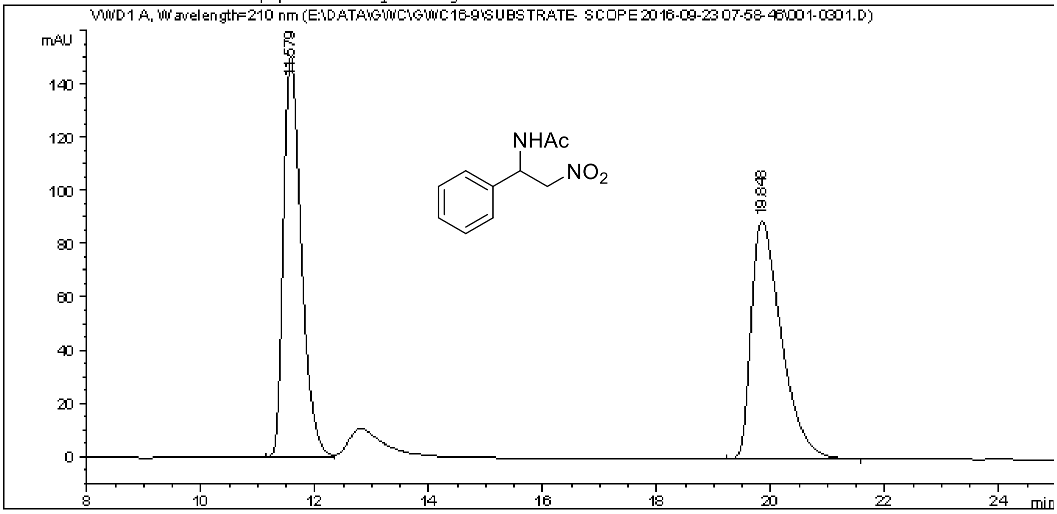


```
    =====================================================================
                             Area Percent Report
    =====================================================================

    Sorted By             :      Signal
    Multiplier            :      1.0000
    Dilution              :      1.0000
    Do not use Multiplier & Dilution Factor with ISTDs


    Signal 1: VWD1 A, Wavelength=210 nm

    Peak RetTime Type  Width     Area      Height     Area
      #   [min]        [min]   [mAU*s]     [mAU]        %
    ----|-------|----|-------|----------|----------|--------|
       1  11.579 BV    0.3331 3282.81226  150.73700  50.0626
       2  19.848 BB    0.5575 3274.59888   89.36766  49.9374

    Totals :                  6557.41113  240.10466

    =====================================================================
                              *** End of Report ***
```

HPLC-2a-**cat**

```
Data File E:\DATA\GWC\GWC16-9\SUBSTRATE- SCOPE 2016-09-23 07-58-46\014-2001.D
Sample Name: biao-cat

    ===================================================================
    Acq. Operator   : SYSTEM                        Seq. Line :  20
    Acq. Instrument : 1260HPLC-VWD                   Location : Vial 14
    Injection Date  : 9/23/2016 2:15:31 PM                Inj :  1
                                                   Inj Volume : 5.000 µl
    Acq. Method     : E:\DATA\GWC\GWC16-9\SUBSTRATE- SCOPE 2016-09-23 07-58-46\VWD-ADH(1-6)-90
                      -10-210NM-40MIN.M
    Last changed    : 9/23/2016 2:18:34 PM by SYSTEM
                      (modified after loading)
    Analysis Method : E:\DATA\GWC\GWC16-9\SUBSTRATE- SCOPE 2016-09-23 07-58-46\VWD-ADH(1-6)-90
                      -10-210NM-40MIN.M (Sequence Method)
    Last changed    : 10/25/2016 8:28:02 PM by SYSTEM
                      (modified after loading)
    Additional Info : Peak(s) manually integrated
```

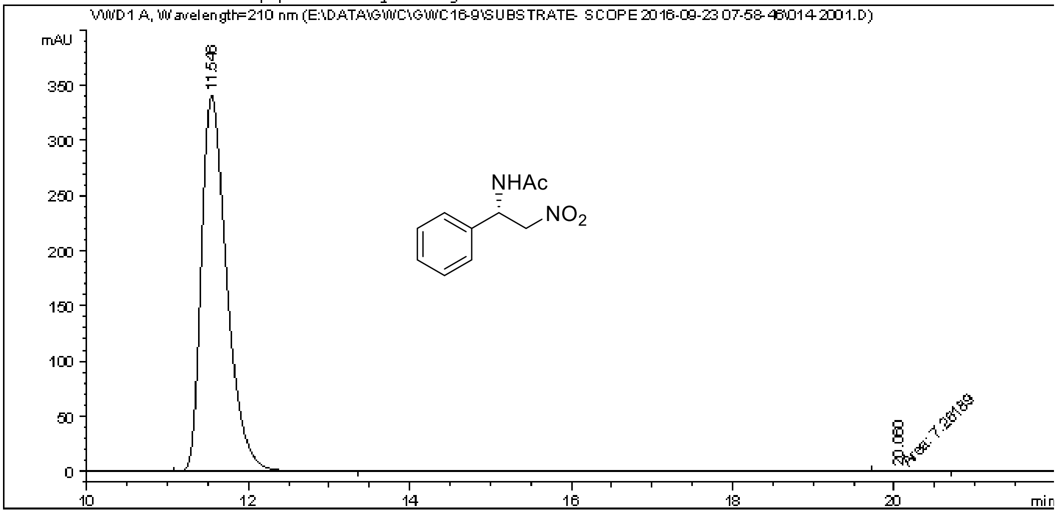


```
    ===================================================================
                             Area Percent Report
    ===================================================================

    Sorted By             :      Signal
    Multiplier            :      1.0000
    Dilution              :      1.0000
    Do not use Multiplier & Dilution Factor with ISTDs


    Signal 1: VWD1 A, Wavelength=210 nm

    Peak RetTime Type  Width     Area      Height     Area
      #   [min]        [min]   [mAU*s]     [mAU]        %
    ----|-------|----|-------|----------|----------|--------|
       1  11.546 BB    0.3353 7448.50586  340.40121  99.9026
       2  20.060 MM    0.4875    7.26189 2.48253e-1   0.0974

    Totals :                  7455.76775  340.64947


    ===================================================================
                             *** End of Report ***
```

HPLC-2b-rac

```
Data File E:\DATA\GWC\GWC16-9\NI-TEMP-2 2016-09-11 19-34-06\024-0501.D
Sample Name: rac

    =====================================================================
    Acq. Operator   : SYSTEM                          Seq. Line :   5
    Acq. Instrument : 1260HPLC-VWD                     Location : Vial 24
    Injection Date  : 9/11/2016 8:48:00 PM                  Inj :   1
                                                     Inj Volume : 5.000 µl
    Acq. Method     : E:\DATA\GWC\GWC16-9\NI-TEMP-2 2016-09-11 19-34-06\VWD-ADH(1-6)-90-10-
                      210NM-20MIN.M
    Last changed    : 9/11/2016 7:34:06 PM by SYSTEM
    Analysis Method : E:\DATA\GWC\GWC16-9\NI-TEMP-2 2016-09-11 19-34-06\VWD-ADH(1-6)-90-10-
                      210NM-20MIN.M (Sequence Method)
    Last changed    : 10/25/2016 9:05:10 PM by SYSTEM
                      (modified after loading)
    Additional Info : Peak(s) manually integrated
```

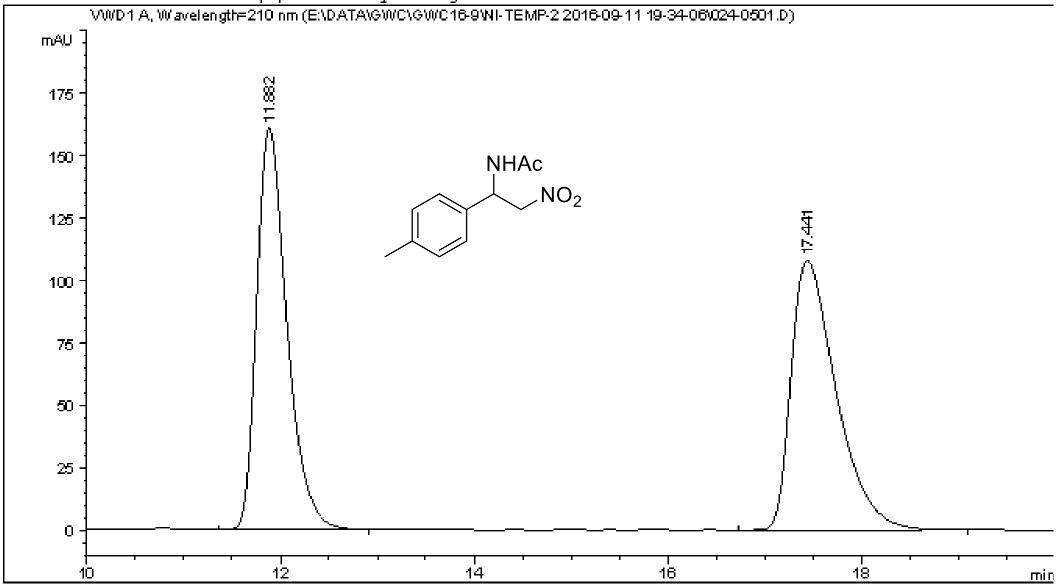


```
    =====================================================================
                             Area Percent Report
    =====================================================================

    Sorted By             :      Signal
    Multiplier            :      1.0000
    Dilution              :      1.0000
    Do not use Multiplier & Dilution Factor with ISTDs


    Signal 1: VWD1 A, Wavelength=210 nm
```

| Peak # | RetTime [min] | Type | Width [min] | Area [mAU*s] | Height [mAU] | Area % |
|---|---|---|---|---|---|---|
| 1 | 11.882 | BB | 0.3322 | 3490.79248 | 160.83640 | 49.9750 |
| 2 | 17.441 | BB | 0.4915 | 3494.28735 | 107.97732 | 50.0250 |

Totals : 6985.07983 268.81371

```
    =====================================================================
                            *** End of Report ***
```

1260HPLC-DAD 10/25/2016 9:05:17 PM SYSTEM Page 1 of 1

HPLC-2b-cat

Data File E:\DATA\GWC\GWC16-9\NI-TEMP-2 2016-09-11 19-34-06\022-0301.D
Sample Name: 4

=====================================================================

```
Acq. Operator   : SYSTEM                          Seq. Line :   3
Acq. Instrument : 1260HPLC-VWD                     Location : Vial 22
Injection Date  : 9/11/2016 8:06:26 PM                  Inj :   1
                                                 Inj Volume : 5.000 µl
Acq. Method     : E:\DATA\GWC\GWC16-9\NI-TEMP-2 2016-09-11 19-34-06\VWD-ADH(1-6)-90-10-
                  210NM-20MIN.M
Last changed    : 9/11/2016 7:34:06 PM by SYSTEM
Analysis Method : E:\DATA\GWC\GWC16-9\NI-TEMP-2 2016-09-11 19-34-06\VWD-ADH(1-6)-90-10-
                  210NM-20MIN.M (Sequence Method)
Last changed    : 10/25/2016 9:05:37 PM by SYSTEM
                  (modified after loading)
Additional Info : Peak(s) manually integrated
```

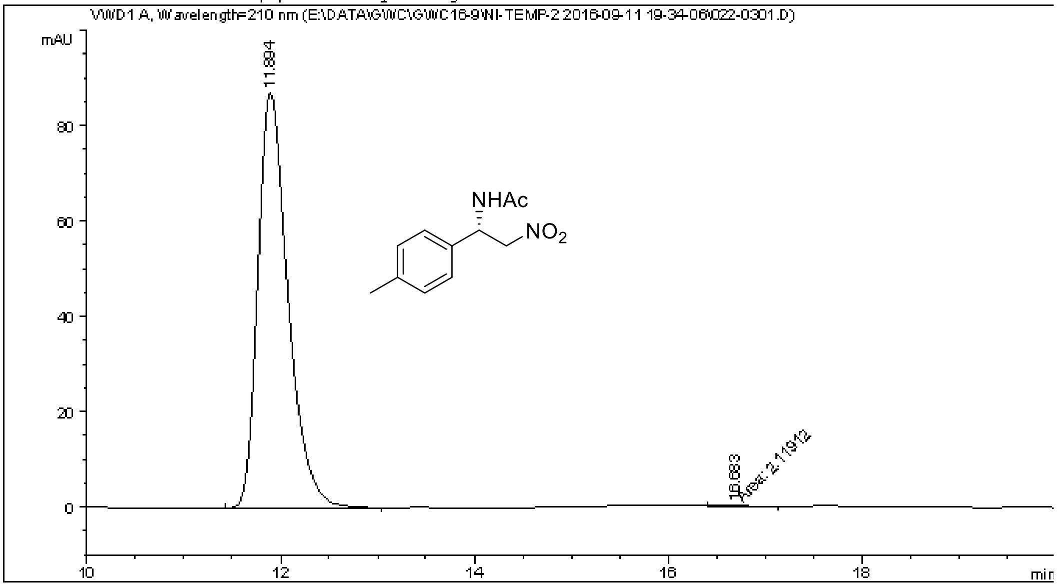


=====================================================================

Area Percent Report

=====================================================================

```
Sorted By             :      Signal
Multiplier            :      1.0000
Dilution              :      1.0000
Do not use Multiplier & Dilution Factor with ISTDs
```

Signal 1: VWD1 A, Wavelength=210 nm

| Peak # | RetTime [min] | Type | Width [min] | Area [mAU*s] | Height [mAU] | Area % |
|---|---|---|---|---|---|---|
| 1 | 11.894 | BB | 0.3290 | 1878.40344 | 86.97512 | 99.8873 |
| 2 | 16.683 | MM | 0.2885 | 2.11912 | 1.22423e-1 | 0.1127 |

Totals : 1880.52256 87.09754

=====================================================================

*** End of Report ***

HPLC-2c-rac

```
Data File E:\DATA\GWC\GWC16-9\SUBSTRATE- SCOPE 2016-09-23 07-58-46\011-1401.D
Sample Name: OMe-rac

    =====================================================================
    Acq. Operator   : SYSTEM                          Seq. Line :  14
    Acq. Instrument : 1260HPLC-VWD                     Location : Vial 11
    Injection Date  : 9/23/2016 12:26:45 PM                  Inj :  1
                                                     Inj Volume : 5.000 µl
    Acq. Method     : E:\DATA\GWC\GWC16-9\SUBSTRATE- SCOPE 2016-09-23 07-58-46\VWD-ADH(1-6)-85
                      -15-210NM-30MIN.M
    Last changed    : 9/23/2016 12:30:57 PM by SYSTEM
                      (modified after loading)
    Analysis Method : E:\DATA\GWC\GWC16-9\SUBSTRATE- SCOPE 2016-09-23 07-58-46\VWD-ADH(1-6)-85
                      -15-210NM-30MIN.M (Sequence Method)
    Last changed    : 10/25/2016 8:14:43 PM by SYSTEM
                      (modified after loading)
    Additional Info : Peak(s) manually integrated
```

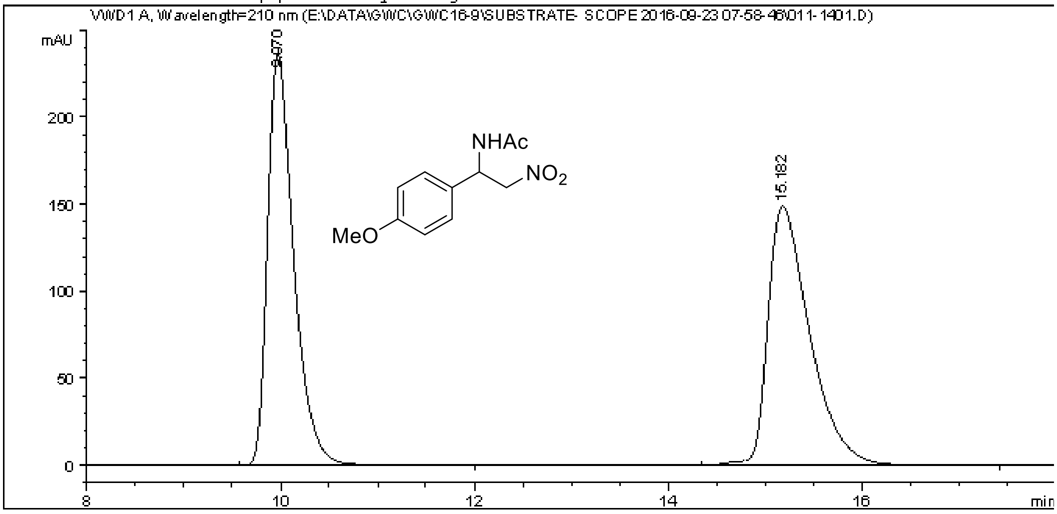


```
    =====================================================================
                             Area Percent Report
    =====================================================================

    Sorted By             :      Signal
    Multiplier            :      1.0000
    Dilution              :      1.0000
    Do not use Multiplier & Dilution Factor with ISTDs


    Signal 1: VWD1 A, Wavelength=210 nm

    Peak RetTime Type  Width     Area      Height     Area
      #   [min]        [min]   [mAU*s]     [mAU]        %
    ----|-------|----|-------|----------|----------|--------|
       1   9.970 BB    0.2851 4435.74561  236.17282  49.8124
       2  15.182 BB    0.4532 4469.14990  148.88206  50.1876

    Totals :                  8904.89551  385.05489

    =====================================================================
                             *** End of Report ***
```

HPLC-2c-cat

```
Data File E:\DATA\GWC\GWC16-9\SUBSTRATE- SCOPE 2016-09-23 07-58-46\022-1701.D
Sample Name: 4-ome-cat

    =====================================================================
    Acq. Operator   : SYSTEM                        Seq. Line :  17
    Acq. Instrument : 1260HPLC-VWD                   Location : Vial 22
    Injection Date  : 9/23/2016 1:23:05 PM                Inj :  1
                                                   Inj Volume : 5.000 µl
    Acq. Method     : E:\DATA\GWC\GWC16-9\SUBSTRATE- SCOPE 2016-09-23 07-58-46\VWD-ADH(1-6)-85
                      -15-210NM-30MIN.M
    Last changed    : 9/23/2016 1:23:17 PM by SYSTEM
                      (modified after loading)
    Analysis Method : E:\DATA\GWC\GWC16-9\SUBSTRATE- SCOPE 2016-09-23 07-58-46\VWD-ADH(1-6)-85
                      -15-210NM-30MIN.M (Sequence Method)
    Last changed    : 10/25/2016 8:25:15 PM by SYSTEM
                      (modified after loading)
    Additional Info : Peak(s) manually integrated
```

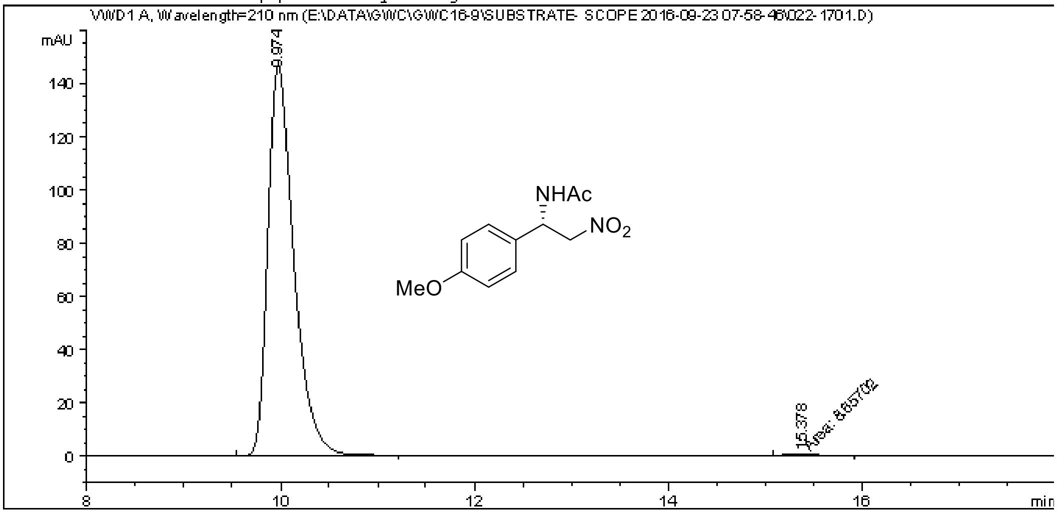


```
    =====================================================================
                             Area Percent Report
    =====================================================================

    Sorted By             :      Signal
    Multiplier            :      1.0000
    Dilution              :      1.0000
    Do not use Multiplier & Dilution Factor with ISTDs


    Signal 1: VWD1 A, Wavelength=210 nm

    Peak RetTime Type  Width     Area      Height     Area
      #   [min]        [min]   [mAU*s]     [mAU]        %
    ----|-------|----|-------|----------|----------|--------|
       1   9.974 BB    0.2836 2728.93823  146.27979  99.6838
       2  15.378 MM    0.4133    8.65702 3.49128e-1   0.3162

    Totals :                  2737.59526  146.62891

    =====================================================================
                             *** End of Report ***
```

HPLC-2d-rac

Data File E:\DATA\GWC\GWC16-9\SUBSTRATE- SCOPE 2016-09-23 07-58-46\007-0501.D
Sample Name: tBu-rac

=====================================================================

Acq. Operator : SYSTEM Seq. Line : 5
Acq. Instrument : 1260HPLC-VWD Location : Vial 7
Injection Date : 9/23/2016 9:32:37 AM Inj : 1
Inj Volume : 5.000 µl
Acq. Method : E:\DATA\GWC\GWC16-9\SUBSTRATE- SCOPE 2016-09-23 07-58-46\VWD-ADH(1-6)-90-10-210NM-40MIN.M
Last changed : 9/23/2016 9:47:19 AM by SYSTEM
(modified after loading)
Analysis Method : E:\DATA\GWC\GWC16-9\SUBSTRATE- SCOPE 2016-09-23 07-58-46\VWD-ADH(1-6)-90-10-210NM-40MIN.M (Sequence Method)
Last changed : 10/25/2016 8:05:00 PM by SYSTEM
(modified after loading)
Additional Info : Peak(s) manually integrated

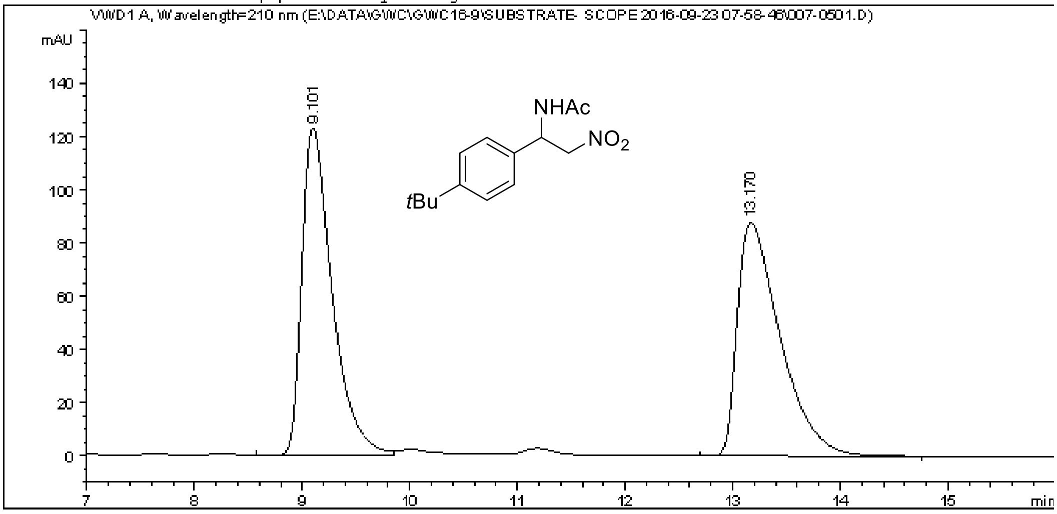


=====================================================================

Area Percent Report

=====================================================================

Sorted By : Signal
Multiplier : 1.0000
Dilution : 1.0000
Do not use Multiplier & Dilution Factor with ISTDs

Signal 1: VWD1 A, Wavelength=210 nm

| Peak # | RetTime [min] | Type | Width [min] | Area [mAU*s] | Height [mAU] | Area % |
|---|---|---|---|---|---|---|
| 1 | 9.101 | BV | 0.2952 | 2405.08936 | 122.98277 | 49.7564 |
| 2 | 13.170 | BB | 0.4168 | 2428.64331 | 87.78138 | 50.2436 |

Totals : 4833.73267 210.76415

=====================================================================

*** End of Report ***

HPLC-2d-cat

Data File E:\DATA\DX\ZXD-32\ZXD-32-34-44-46-AD-AS 2016-09-25 15-35-12\011-0401.D
Sample Name: tBu-cat

=====================================================================

Acq. Operator : SYSTEM Seq. Line : 4
Acq. Instrument : 1260HPLC-VWD Location : Vial 11
Injection Date : 9/25/2016 4:28:15 PM Inj : 1
Inj Volume : 5.000 µl
Acq. Method : E:\DATA\DX\ZXD-32\ZXD-32-34-44-46-AD-AS 2016-09-25 15-35-12\VWD-ADH(1-6)-90-10-210NM-20MIN.M
Last changed : 9/25/2016 3:35:13 PM by SYSTEM
Analysis Method : E:\DATA\DX\ZXD-32\ZXD-32-34-44-46-AD-AS 2016-09-25 15-35-12\VWD-ADH(1-6)-90-10-210NM-20MIN.M (Sequence Method)
Last changed : 12/16/2016 10:16:21 AM by SYSTEM
(modified after loading)
Additional Info : Peak(s) manually integrated

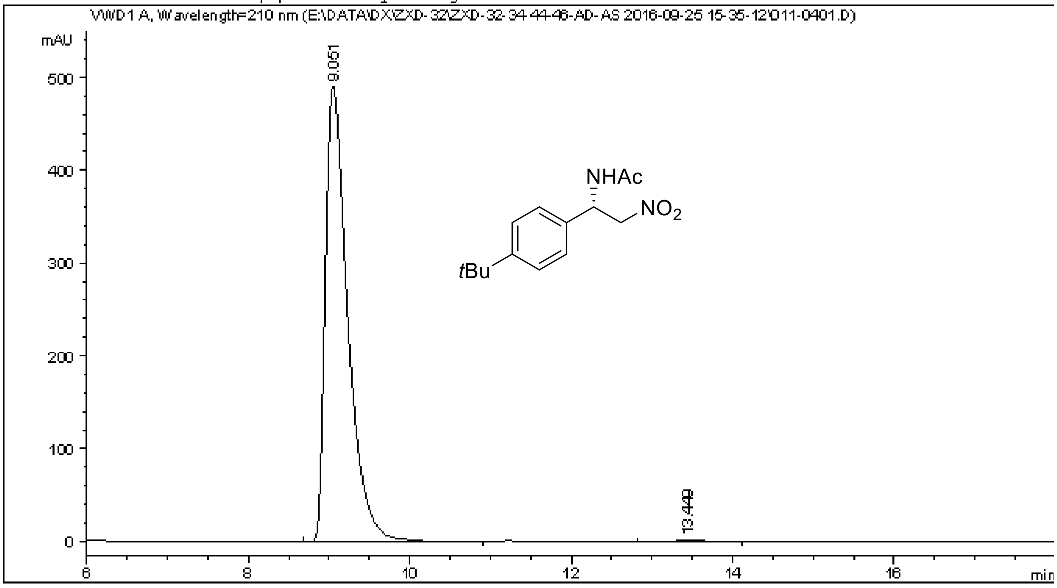


=====================================================================

Area Percent Report

=====================================================================

Sorted By : Signal
Multiplier : 1.0000
Dilution : 1.0000
Do not use Multiplier & Dilution Factor with ISTDs

Signal 1: VWD1 A, Wavelength=210 nm

| Peak # | RetTime [min] | Type | Width [min] | Area [mAU*s] | Height [mAU] | Area % |
|---|---|---|---|---|---|---|
| 1 | 9.051 | BB | 0.2895 | 9401.23535 | 490.67557 | 99.5203 |
| 2 | 13.449 | BB | 0.3760 | 45.31156 | 1.77419 | 0.4797 |
| Totals : | | | | 9446.54691 | 492.44976 | |

=====================================================================

*** End of Report ***

HPLC-2e-rac

Data File E:\DATA\GWC\GWC16-9\SUBSTRATE- SCOPE 2016-09-23 07-58-46\002-0401.D
Sample Name: F-rac

=====================================================================

Acq. Operator : SYSTEM Seq. Line : 4
Acq. Instrument : 1260HPLC-VWD Location : Vial 2
Injection Date : 9/23/2016 9:01:49 AM Inj : 1
Inj Volume : 5.000 µl
Acq. Method : E:\DATA\GWC\GWC16-9\SUBSTRATE- SCOPE 2016-09-23 07-58-46\VWD-ADH(1-6)-90-10-210NM-40MIN.M
Last changed : 9/23/2016 9:17:36 AM by SYSTEM
(modified after loading)
Analysis Method : E:\DATA\GWC\GWC16-9\SUBSTRATE- SCOPE 2016-09-23 07-58-46\VWD-ADH(1-6)-90-10-210NM-40MIN.M (Sequence Method)
Last changed : 10/25/2016 8:02:58 PM by SYSTEM
(modified after loading)
Additional Info : Peak(s) manually integrated

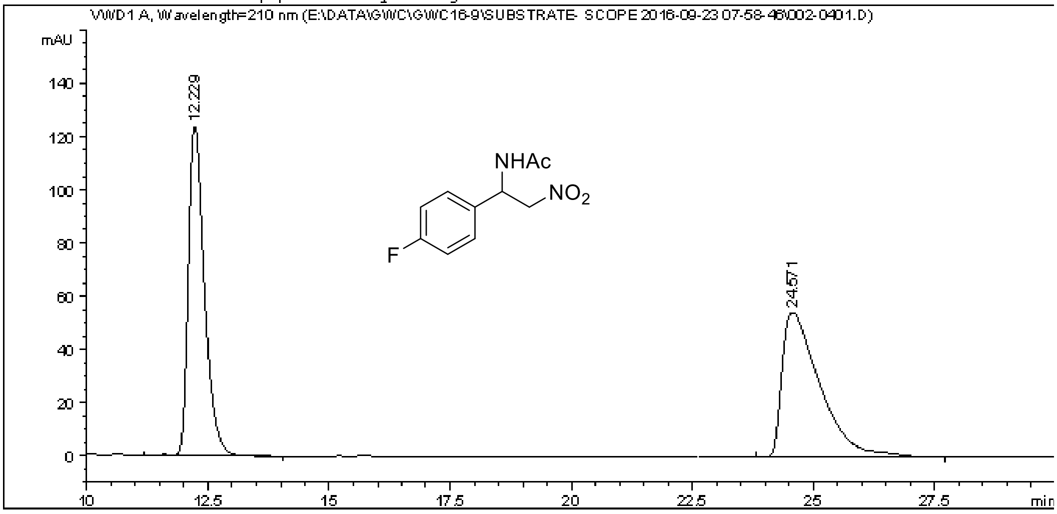


=====================================================================

Area Percent Report

=====================================================================

Sorted By : Signal
Multiplier : 1.0000
Dilution : 1.0000
Do not use Multiplier & Dilution Factor with ISTDs

Signal 1: VWD1 A, Wavelength=210 nm

| Peak # | RetTime [min] | Type | Width [min] | Area [mAU*s] | Height [mAU] | Area % |
|---|---|---|---|---|---|---|
| 1 | 12.229 | BB | 0.3536 | 2873.46655 | 123.86520 | 49.2803 |
| 2 | 24.571 | BB | 0.7853 | 2957.39014 | 54.47705 | 50.7197 |

Totals : 5830.85669 178.34226

=====================================================================

*** End of Report ***

HPLC-2e-cat

Data File E:\DATA\GWC\GWC16-9\SUBSTRATE- SCOPE 2016-09-23 07-58-46\021-2101.D
Sample Name: 4-f-cat

=====================================================================

Acq. Operator : SYSTEM    Seq. Line : 21
Acq. Instrument : 1260HPLC-VWD    Location : Vial 21
Injection Date : 9/23/2016 2:41:47 PM    Inj : 1
Inj Volume : 5.000 µl
Acq. Method : E:\DATA\GWC\GWC16-9\SUBSTRATE- SCOPE 2016-09-23 07-58-46\VWD-ADH(1-6)-90-10-210NM-40MIN.M
Last changed : 9/23/2016 2:42:30 PM by SYSTEM (modified after loading)
Analysis Method : E:\DATA\GWC\GWC16-9\SUBSTRATE- SCOPE 2016-09-23 07-58-46\VWD-ADH(1-6)-90-10-210NM-40MIN.M (Sequence Method)
Last changed : 10/25/2016 8:30:14 PM by SYSTEM (modified after loading)
Additional Info : Peak(s) manually integrated

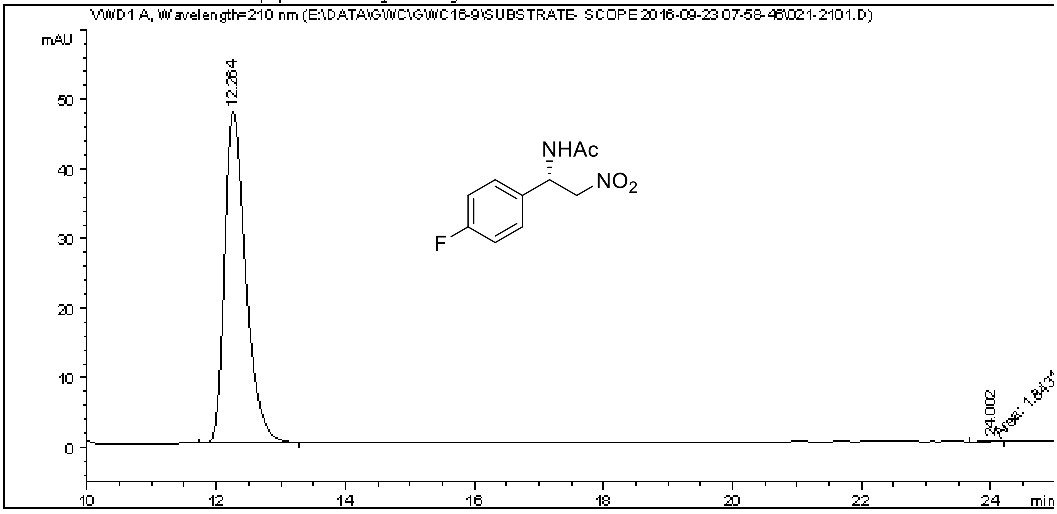


=====================================================================

Area Percent Report

=====================================================================

Sorted By : Signal
Multiplier : 1.0000
Dilution : 1.0000
Do not use Multiplier & Dilution Factor with ISTDs

Signal 1: VWD1 A, Wavelength=210 nm

| Peak # | RetTime [min] | Type | Width [min] | Area [mAU*s] | Height [mAU] | Area % |
|---|---|---|---|---|---|---|
| 1 | 12.264 | BB | 0.3478 | 1087.23499 | 47.55069 | 99.8308 |
| 2 | 24.002 | MM | 0.3073 | 1.84314 | 9.99646e-2 | 0.1692 |

Totals : 1089.07812 47.65066

=====================================================================

*** End of Report ***

HPLC-2f-rac

Data File E:\DATA\GWC\GWC16-9\SUBSTRATE- SCOPE 2016-09-23 07-58-46\003-0901.D
Sample Name: Cl-rac

=====================================================================

Acq. Operator : SYSTEM    Seq. Line : 9
Acq. Instrument : 1260HPLC-VWD    Location : Vial 3
Injection Date : 9/23/2016 10:45:44 AM    Inj : 1
Inj Volume : 5.000 µl
Acq. Method : E:\DATA\GWC\GWC16-9\SUBSTRATE- SCOPE 2016-09-23 07-58-46\VWD-ADH(1-6)-90-10-210NM-40MIN.M
Last changed : 9/23/2016 11:16:28 AM by SYSTEM
(modified after loading)
Analysis Method : E:\DATA\GWC\GWC16-9\SUBSTRATE- SCOPE 2016-09-23 07-58-46\VWD-ADH(1-6)-90-10-210NM-40MIN.M (Sequence Method)
Last changed : 10/25/2016 8:09:49 PM by SYSTEM
(modified after loading)
Additional Info : Peak(s) manually integrated

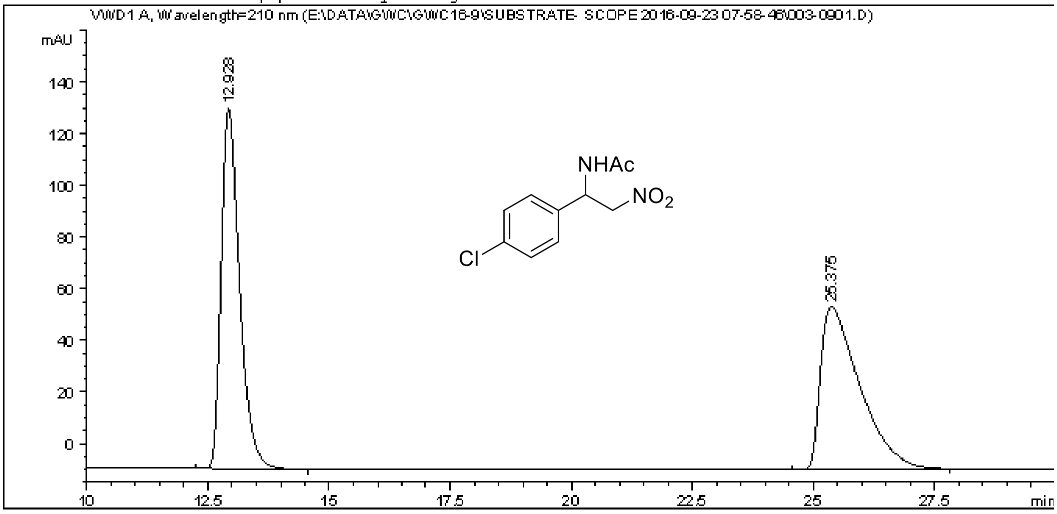


=====================================================================

Area Percent Report

=====================================================================

Sorted By : Signal
Multiplier : 1.0000
Dilution : 1.0000
Do not use Multiplier & Dilution Factor with ISTDs

Signal 1: VWD1 A, Wavelength=210 nm

| Peak # | RetTime [min] | Type | Width [min] | Area [mAU*s] | Height [mAU] | Area % |
|---|---|---|---|---|---|---|
| 1 | 12.928 | BB | 0.4035 | 3685.12231 | 139.38007 | 50.9043 |
| 2 | 25.375 | BB | 0.8318 | 3554.19092 | 62.84177 | 49.0957 |

Totals : 7239.31323 202.22183

=====================================================================

*** End of Report ***

HPLC-2f-cat

```
Data File E:\DATA\GWC\GWC16-9\SUBSTRATE- SCOPE 2016-09-23 07-58-46\023-2201.D
Sample Name: 4-cl-cat

    ===================================================================
    Acq. Operator   : SYSTEM                        Seq. Line :  22
    Acq. Instrument : 1260HPLC-VWD                   Location : Vial 23
    Injection Date  : 9/23/2016 3:12:34 PM                Inj :  1
                                                   Inj Volume : 5.000 µl
    Acq. Method     : E:\DATA\GWC\GWC16-9\SUBSTRATE- SCOPE 2016-09-23 07-58-46\VWD-ADH(1-6)-90
                      -10-210NM-40MIN.M
    Last changed    : 9/23/2016 2:42:30 PM by SYSTEM
    Analysis Method : E:\DATA\GWC\GWC16-9\SUBSTRATE- SCOPE 2016-09-23 07-58-46\VWD-ADH(1-6)-90
                      -10-210NM-40MIN.M (Sequence Method)
    Last changed    : 10/25/2016 8:34:19 PM by SYSTEM
                      (modified after loading)
    Additional Info : Peak(s) manually integrated
```

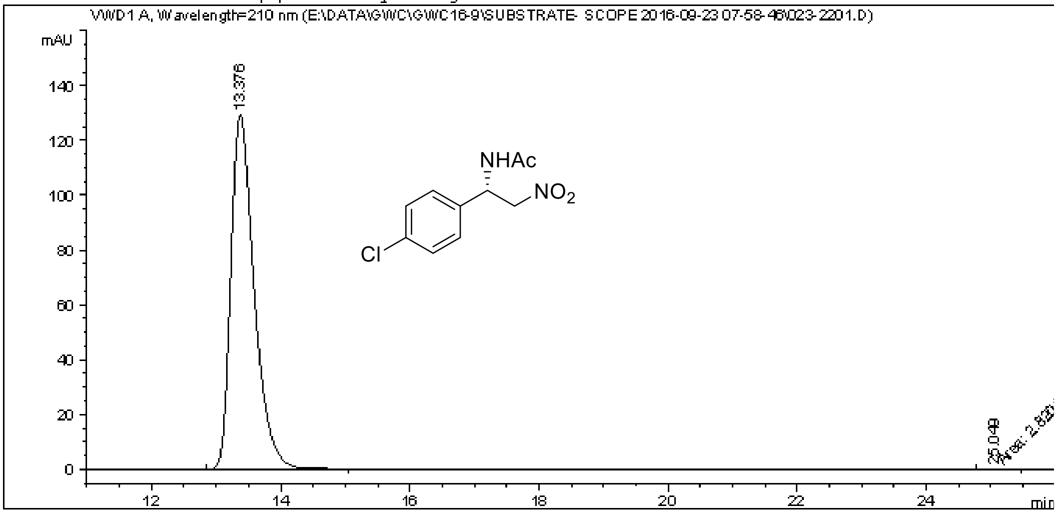


```
    ===================================================================
                          Area Percent Report
    ===================================================================

    Sorted By             :      Signal
    Multiplier            :      1.0000
    Dilution              :      1.0000
    Do not use Multiplier & Dilution Factor with ISTDs


    Signal 1: VWD1 A, Wavelength=210 nm

    Peak RetTime Type  Width     Area      Height     Area
      #   [min]        [min]   [mAU*s]     [mAU]        %
    ----|-------|----|-------|----------|----------|--------|
       1  13.376 BB    0.3811 3233.29492  129.12560  99.9128
       2  25.049 MM    0.4247    2.82047 1.10689e-1   0.0872

    Totals :                  3236.11540  129.23628

    ===================================================================
                           *** End of Report ***
```

HPLC-2g-rac

Data File E:\DATA\GWC\GWC16-9\SUBSTRATE- SCOPE 2016-09-23 07-58-46\004-1101.D
Sample Name: Br-rac

=====================================================================

Acq. Operator : SYSTEM Seq. Line : 11
Acq. Instrument : 1260HPLC-VWD Location : Vial 4
Injection Date : 9/23/2016 11:29:23 AM Inj : 1
Inj Volume : 5.000 µl
Acq. Method : E:\DATA\GWC\GWC16-9\SUBSTRATE- SCOPE 2016-09-23 07-58-46\VWD-ADH(1-6)-85-15-210NM-30MIN.M
Last changed : 9/23/2016 11:47:07 AM by SYSTEM
(modified after loading)
Analysis Method : E:\DATA\GWC\GWC16-9\SUBSTRATE- SCOPE 2016-09-23 07-58-46\VWD-ADH(1-6)-85-15-210NM-30MIN.M (Sequence Method)
Last changed : 10/25/2016 8:10:49 PM by SYSTEM
(modified after loading)
Additional Info : Peak(s) manually integrated

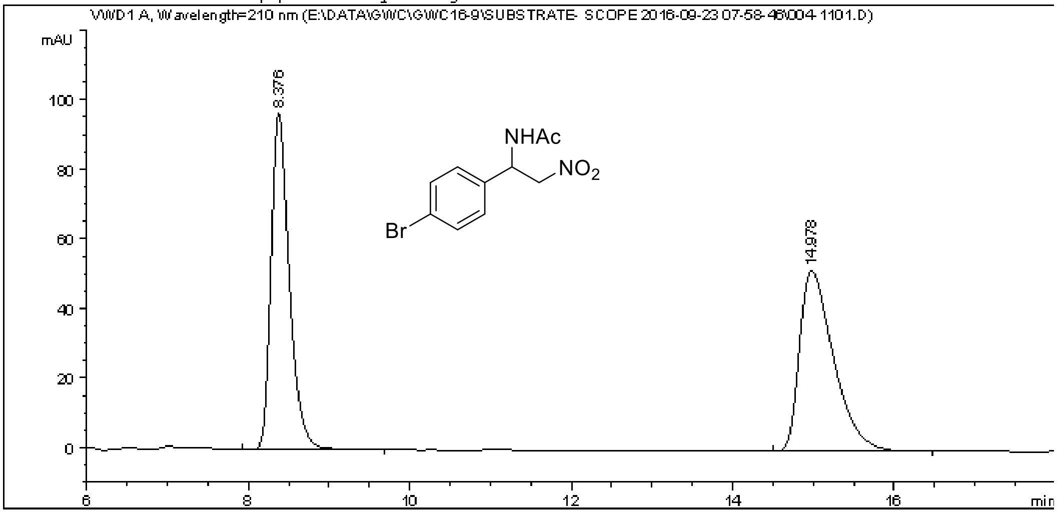


=====================================================================

Area Percent Report

=====================================================================

Sorted By : Signal
Multiplier : 1.0000
Dilution : 1.0000
Do not use Multiplier & Dilution Factor with ISTDs

Signal 1: VWD1 A, Wavelength=210 nm

| Peak # | RetTime [min] | Type | Width [min] | Area [mAU*s] | Height [mAU] | Area % |
|---|---|---|---|---|---|---|
| 1 | 8.376 | BB | 0.2391 | 1517.92566 | 96.70007 | 50.0947 |
| 2 | 14.978 | BB | 0.4456 | 1512.18921 | 51.79261 | 49.9053 |

Totals : 3030.11487 148.49268

=====================================================================

*** End of Report ***

HPLC-2g-cat

Data File E:\DATA\GWC\GWC16-9\SUBSTRATE- SCOPE 2016-09-23 07-58-46\024-1501.D
Sample Name: 4-br-cat

=====================================================================
Acq. Operator : SYSTEM Seq. Line : 15
Acq. Instrument : 1260HPLC-VWD Location : Vial 24
Injection Date : 9/23/2016 12:47:32 PM Inj : 1
Inj Volume : 5.000 µl
Acq. Method : E:\DATA\GWC\GWC16-9\SUBSTRATE- SCOPE 2016-09-23 07-58-46\VWD-ADH(1-6)-85-15-210NM-30MIN.M
Last changed : 9/23/2016 12:30:57 PM by SYSTEM
Analysis Method : E:\DATA\GWC\GWC16-9\SUBSTRATE- SCOPE 2016-09-23 07-58-46\VWD-ADH(1-6)-85-15-210NM-30MIN.M (Sequence Method)
Last changed : 10/25/2016 8:20:39 PM by SYSTEM
(modified after loading)
Additional Info : Peak(s) manually integrated

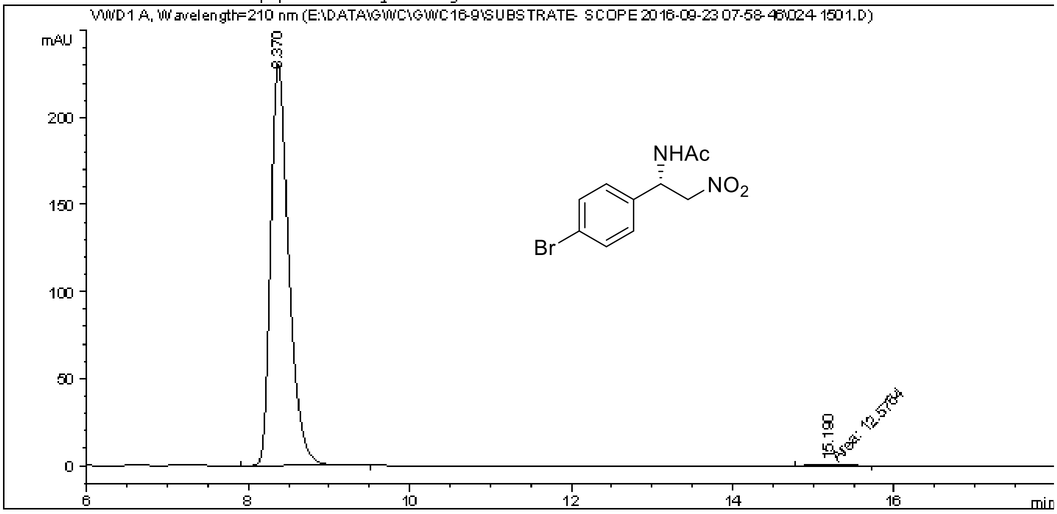


=====================================================================
Area Percent Report
=====================================================================

Sorted By : Signal
Multiplier : 1.0000
Dilution : 1.0000
Do not use Multiplier & Dilution Factor with ISTDs

Signal 1: VWD1 A, Wavelength=210 nm

| Peak # | RetTime [min] | Type | Width [min] | Area [mAU*s] | Height [mAU] | Area % |
|---|---|---|---|---|---|---|
| 1 | 8.370 | BB | 0.2369 | 3599.86133 | 230.84367 | 99.6519 |
| 2 | 15.190 | MM | 0.4652 | 12.57644 | 4.50604e-1 | 0.3481 |

Totals : 3612.43776 231.29428

=====================================================================
*** End of Report ***

HPLC-2h-rac

```
Data File E:\DATA\DX\ZXD-32\ZXD-32-34-44-46-AD-AS 2016-09-24 18-30-10\011-1101.D
Sample Name: 3-ME-rac

    =====================================================================
    Acq. Operator   : SYSTEM                          Seq. Line :  11
    Acq. Instrument : 1260HPLC-VWD                     Location : Vial 11
    Injection Date  : 9/25/2016 12:38:59 AM                 Inj :  1
                                                     Inj Volume : 5.000 µl
    Acq. Method     : E:\DATA\DX\ZXD-32\ZXD-32-34-44-46-AD-AS 2016-09-24 18-30-10\VWD-ADH(1-6)
                      -85-15-210NM-20MIN.M
    Last changed    : 9/24/2016 10:04:57 PM by SYSTEM
    Analysis Method : E:\DATA\DX\ZXD-32\ZXD-32-34-44-46-AD-AS 2016-09-24 18-30-10\VWD-ADH(1-6)
                      -85-15-210NM-20MIN.M (Sequence Method)
    Last changed    : 10/25/2016 8:38:23 PM by SYSTEM
                      (modified after loading)
    Additional Info : Peak(s) manually integrated
```

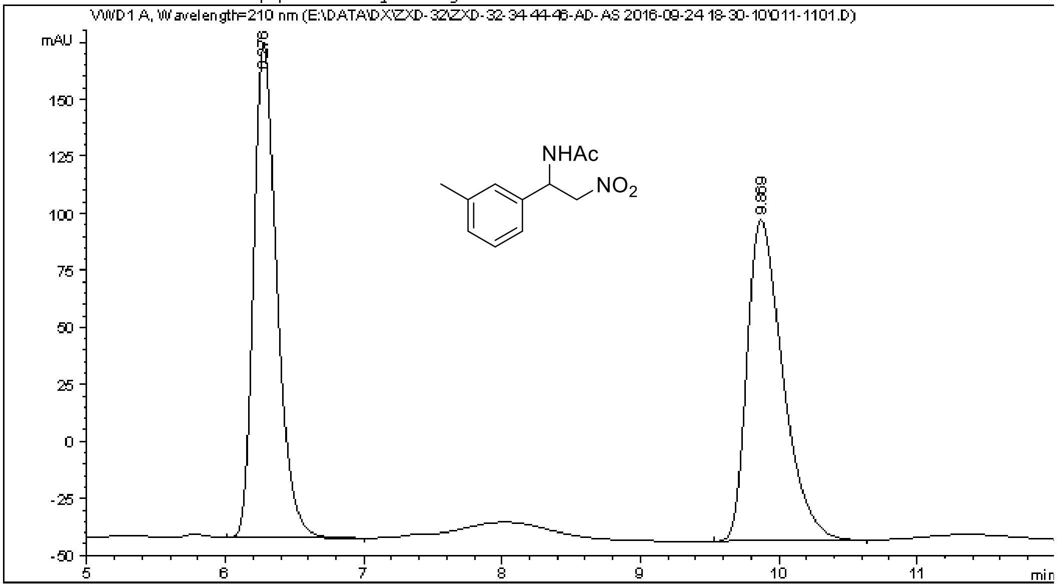


```
    =====================================================================
                             Area Percent Report
    =====================================================================

    Sorted By             :      Signal
    Multiplier            :      1.0000
    Dilution              :      1.0000
    Do not use Multiplier & Dilution Factor with ISTDs


    Signal 1: VWD1 A, Wavelength=210 nm
```

| Peak # | RetTime [min] | Type | Width [min] | Area [mAU*s] | Height [mAU] | Area % |
|---|---|---|---|---|---|---|
| 1 | 6.276 | VB | 0.1799 | 2538.25171 | 216.25807 | 50.0981 |
| 2 | 9.869 | BB | 0.2755 | 2528.31250 | 140.77724 | 49.9019 |

Totals : 5066.56421 357.03531

```
    =====================================================================
                            *** End of Report ***
```

HPLC-2h-cat

Data File E:\DATA\DX\ZXD-32\ZXD-32-34-44-46-AD-AS 2016-09-24 18-30-10\021-1501.D
Sample Name: 3-me-cat

=====================================================================

Acq. Operator : SYSTEM Seq. Line : 15
Acq. Instrument : 1260HPLC-VWD Location : Vial 21
Injection Date : 9/25/2016 2:02:01 AM Inj : 1
Inj Volume : 5.000 µl
Acq. Method : E:\DATA\DX\ZXD-32\ZXD-32-34-44-46-AD-AS 2016-09-24 18-30-10\VWD-ADH(1-6)-85-15-210NM-20MIN.M
Last changed : 9/24/2016 10:04:57 PM by SYSTEM
Analysis Method : E:\DATA\DX\ZXD-32\ZXD-32-34-44-46-AD-AS 2016-09-24 18-30-10\VWD-ADH(1-6)-85-15-210NM-20MIN.M (Sequence Method)
Last changed : 10/25/2016 8:44:33 PM by SYSTEM
(modified after loading)
Additional Info : Peak(s) manually integrated

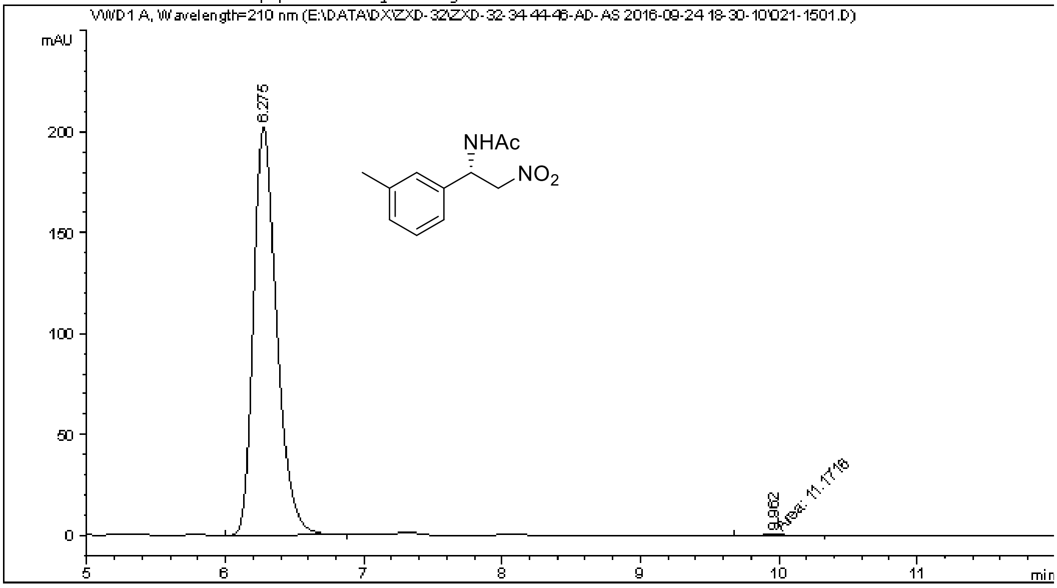


=====================================================================

Area Percent Report

=====================================================================

Sorted By : Signal
Multiplier : 1.0000
Dilution : 1.0000
Do not use Multiplier & Dilution Factor with ISTDs

Signal 1: VWD1 A, Wavelength=210 nm

| Peak # | RetTime [min] | Type | Width [min] | Area [mAU*s] | Height [mAU] | Area % |
|---|---|---|---|---|---|---|
| 1 | 6.275 | BB | 0.1784 | 2362.60815 | 202.04823 | 99.5294 |
| 2 | 9.962 | MM | 0.3113 | 11.17162 | 5.98206e-1 | 0.4706 |

Totals : 2373.77978 202.64644

=====================================================================

*** End of Report ***

HPLC-2i-rac

Data File E:\DATA\DX\ZXD-32\ZXD-32-34-44-46-AD-AS 2016-09-24 18-30-10\012-1201.D
Sample Name: 3-ome-rac

=====================================================================

Acq. Operator : SYSTEM Seq. Line : 12
Acq. Instrument : 1260HPLC-VWD Location : Vial 12
Injection Date : 9/25/2016 12:59:43 AM Inj : 1
Inj Volume : 5.000 µl
Acq. Method : E:\DATA\DX\ZXD-32\ZXD-32-34-44-46-AD-AS 2016-09-24 18-30-10\VWD-ADH(1-6)-85-15-210NM-20MIN.M
Last changed : 9/24/2016 10:04:57 PM by SYSTEM
Analysis Method : E:\DATA\DX\ZXD-32\ZXD-32-34-44-46-AD-AS 2016-09-24 18-30-10\VWD-ADH(1-6)-85-15-210NM-20MIN.M (Sequence Method)
Last changed : 10/25/2016 8:40:38 PM by SYSTEM
(modified after loading)
Additional Info : Peak(s) manually integrated

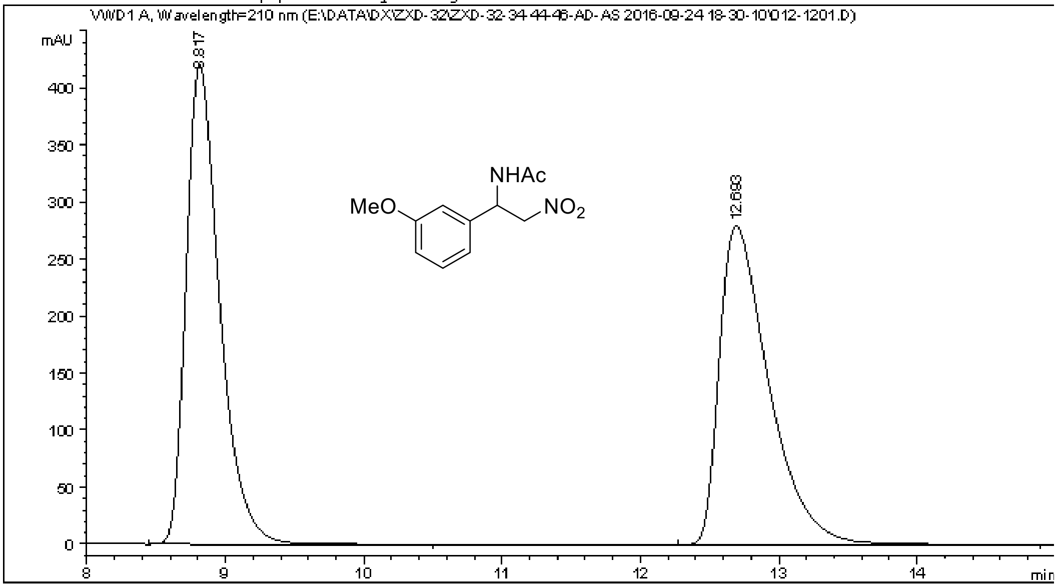


=====================================================================

Area Percent Report

=====================================================================

Sorted By : Signal
Multiplier : 1.0000
Dilution : 1.0000
Do not use Multiplier & Dilution Factor with ISTDs

Signal 1: VWD1 A, Wavelength=210 nm

| Peak # | RetTime [min] | Type | Width [min] | Area [mAU*s] | Height [mAU] | Area % |
|---|---|---|---|---|---|---|
| 1 | 8.817 | BB | 0.2570 | 7102.33496 | 420.51407 | 50.4378 |
| 2 | 12.693 | BB | 0.3772 | 6979.04395 | 279.57132 | 49.5622 |

Totals : 1.40814e4 700.08539

=====================================================================

*** End of Report ***

HPLC-2i-cat

Data File E:\DATA\DX\ZXD-32\ZXD-32-34-44-46-AD-AS 2016-09-24 18-30-10\022-1601.D
Sample Name: 3-ome-cat

```
=====================================================================
Acq. Operator   : SYSTEM                         Seq. Line :  16
Acq. Instrument : 1260HPLC-VWD                    Location : Vial 22
Injection Date  : 9/25/2016 2:22:47 AM                 Inj :  1
                                                Inj Volume : 5.000 µl
Acq. Method     : E:\DATA\DX\ZXD-32\ZXD-32-34-44-46-AD-AS 2016-09-24 18-30-10\VWD-ADH(1-6)
                  -85-15-210NM-20MIN.M
Last changed    : 9/24/2016 10:04:57 PM by SYSTEM
Analysis Method : E:\DATA\DX\ZXD-32\ZXD-32-34-44-46-AD-AS 2016-09-24 18-30-10\VWD-ADH(1-6)
                  -85-15-210NM-20MIN.M (Sequence Method)
Last changed    : 10/25/2016 8:47:10 PM by SYSTEM
                  (modified after loading)
Additional Info : Peak(s) manually integrated
```

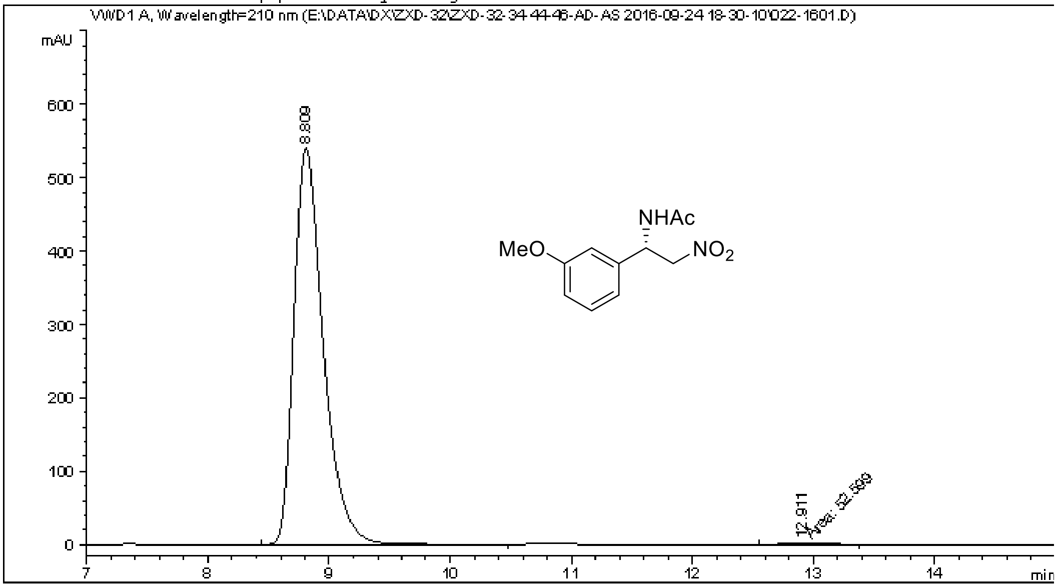


```
=====================================================================
                         Area Percent Report
=====================================================================

Sorted By             :      Signal
Multiplier            :      1.0000
Dilution              :      1.0000
Do not use Multiplier & Dilution Factor with ISTDs


Signal 1: VWD1 A, Wavelength=210 nm
```

| Peak # | RetTime [min] | Type | Width [min] | Area [mAU*s] | Height [mAU] | Area % |
|---|---|---|---|---|---|---|
| 1 | 8.809 | BB | 0.2569 | 9113.59473 | 540.10608 | 99.4262 |
| 2 | 12.911 | MM | 0.3741 | 52.59902 | 2.34333 | 0.5738 |
| Totals : | | | | 9166.19375 | 542.44941 | |

```
=====================================================================
                         *** End of Report ***
```

HPLC-2j-rac

Data File E:\DATA\DX\ZXD-32\ZXD-32-34-44-46-AD-AS 2016-09-24 18-30-10\013-1301.D
Sample Name: 3-cl-rac

=====================================================================

Acq. Operator : SYSTEM　　Seq. Line : 13
Acq. Instrument : 1260HPLC-VWD　　Location : Vial 13
Injection Date : 9/25/2016 1:20:28 AM　　Inj : 1
Inj Volume : 5.000 µl
Acq. Method : E:\DATA\DX\ZXD-32\ZXD-32-34-44-46-AD-AS 2016-09-24 18-30-10\VWD-ADH(1-6)-85-15-210NM-20MIN.M
Last changed : 9/24/2016 10:04:57 PM by SYSTEM
Analysis Method : E:\DATA\DX\ZXD-32\ZXD-32-34-44-46-AD-AS 2016-09-24 18-30-10\VWD-ADH(1-6)-85-15-210NM-20MIN.M (Sequence Method)
Last changed : 10/25/2016 8:41:42 PM by SYSTEM
(modified after loading)
Additional Info : Peak(s) manually integrated

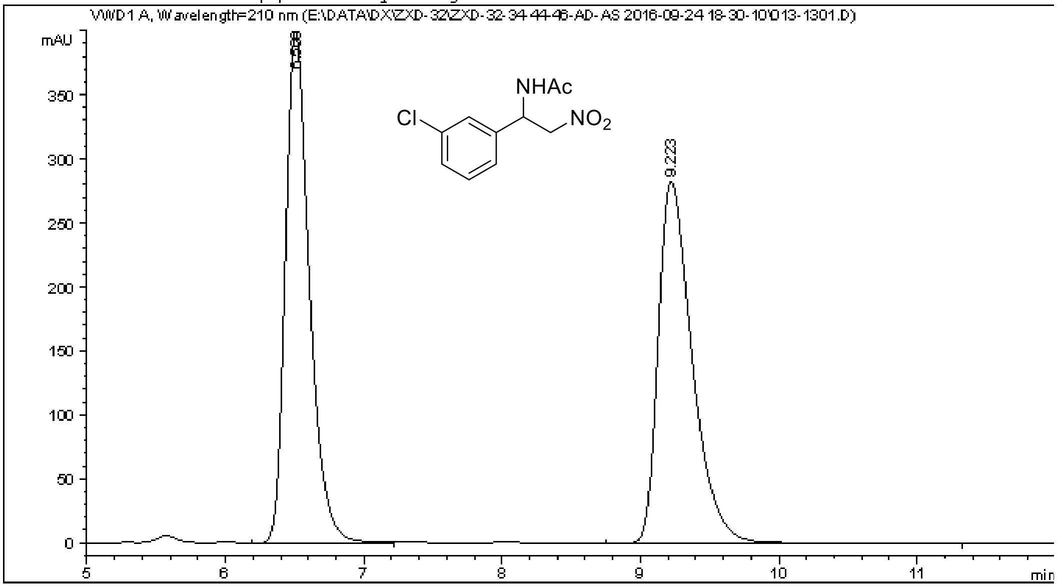


=====================================================================

Area Percent Report

=====================================================================

Sorted By : Signal
Multiplier : 1.0000
Dilution : 1.0000
Do not use Multiplier & Dilution Factor with ISTDs

Signal 1: VWD1 A, Wavelength=210 nm

| Peak # | RetTime [min] | Type | Width [min] | Area [mAU*s] | Height [mAU] | Area % |
|---|---|---|---|---|---|---|
| 1 | 6.509 | BV | 0.1842 | 4849.09326 | 400.60236 | 50.1704 |
| 2 | 9.223 | BB | 0.2598 | 4816.15283 | 282.63071 | 49.8296 |
| Totals : | | | | 9665.24609 | 683.23306 | |

=====================================================================

*** End of Report ***

1260HPLC-DAD 10/25/2016 8:41:46 PM SYSTEM　　Page 1 of 1

HPLC-2j-cat

```
Data File E:\DATA\DX\ZXD-32\ZXD-32-34-44-46-AD-AS 2016-09-24 18-30-10\023-1701.D
Sample Name: 3-cl-cat

    =====================================================================
    Acq. Operator   : SYSTEM                          Seq. Line :  17
    Acq. Instrument : 1260HPLC-VWD                     Location : Vial 23
    Injection Date  : 9/25/2016 2:43:33 AM                  Inj :  1
                                                     Inj Volume : 5.000 µl
    Acq. Method     : E:\DATA\DX\ZXD-32\ZXD-32-34-44-46-AD-AS 2016-09-24 18-30-10\VWD-ADH(1-6)
                      -85-15-210NM-20MIN.M
    Last changed    : 9/24/2016 10:04:57 PM by SYSTEM
    Analysis Method : E:\DATA\DX\ZXD-32\ZXD-32-34-44-46-AD-AS 2016-09-24 18-30-10\VWD-ADH(1-6)
                      -85-15-210NM-20MIN.M (Sequence Method)
    Last changed    : 10/25/2016 8:48:31 PM by SYSTEM
                      (modified after loading)
    Additional Info : Peak(s) manually integrated
```

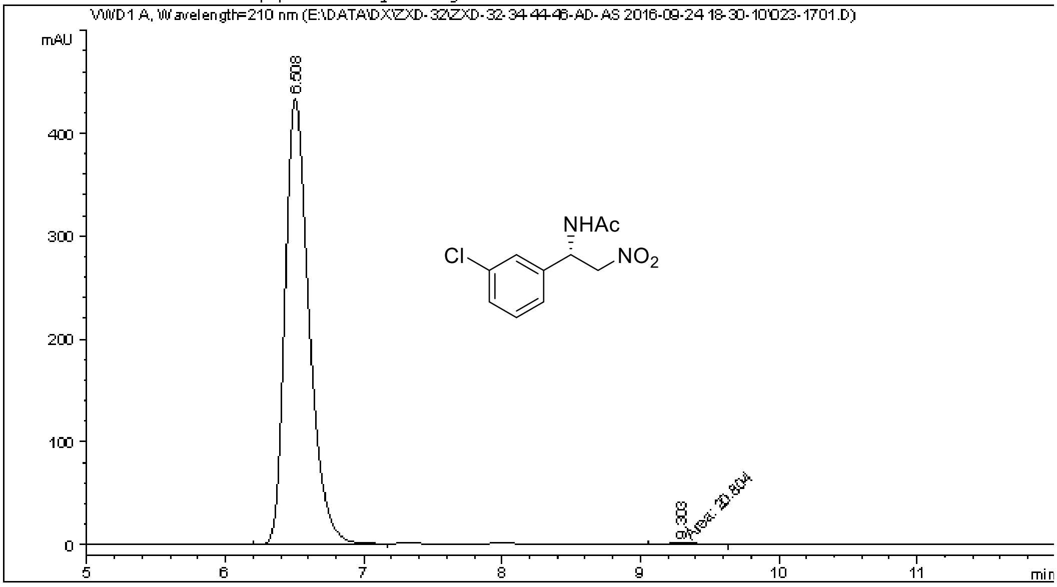


```
    =====================================================================
                             Area Percent Report
    =====================================================================

    Sorted By             :      Signal
    Multiplier            :      1.0000
    Dilution              :      1.0000
    Do not use Multiplier & Dilution Factor with ISTDs


    Signal 1: VWD1 A, Wavelength=210 nm
```

| Peak # | RetTime [min] | Type | Width [min] | Area [mAU*s] | Height [mAU] | Area % |
|---|---|---|---|---|---|---|
| 1 | 6.508 | BB | 0.1846 | 5222.48682 | 433.45673 | 99.6032 |
| 2 | 9.303 | MM | 0.2542 | 20.80397 | 1.36397 | 0.3968 |
| Totals : | | | | 5243.29078 | 434.82070 | |

```
    =====================================================================
                             *** End of Report ***
```

HPLC-2k-rac

```
Data File E:\DATA\GWC\GWC16-10\2-BR AND 3-BR 2016-10-04 09-42-10\001-0501.D
Sample Name: 3-Br-rac

    =====================================================================
    Acq. Operator   : SYSTEM                         Seq. Line :   5
    Acq. Instrument : 1260HPLC-VWD                    Location : Vial 1
    Injection Date  : 10/4/2016 10:56:01 AM                Inj :   1
                                                    Inj Volume : 5.000 µl
    Acq. Method     : E:\DATA\GWC\GWC16-10\2-BR AND 3-BR 2016-10-04 09-42-10\VWD-ADH(1-6)-85-
                      15-210NM-30MIN.M
    Last changed    : 10/4/2016 10:35:17 AM by SYSTEM
    Analysis Method : E:\DATA\GWC\GWC16-10\2-BR AND 3-BR 2016-10-04 09-42-10\VWD-ADH(1-6)-85-
                      15-210NM-30MIN.M (Sequence Method)
    Last changed    : 10/25/2016 8:50:53 PM by SYSTEM
                      (modified after loading)
    Additional Info : Peak(s) manually integrated
```

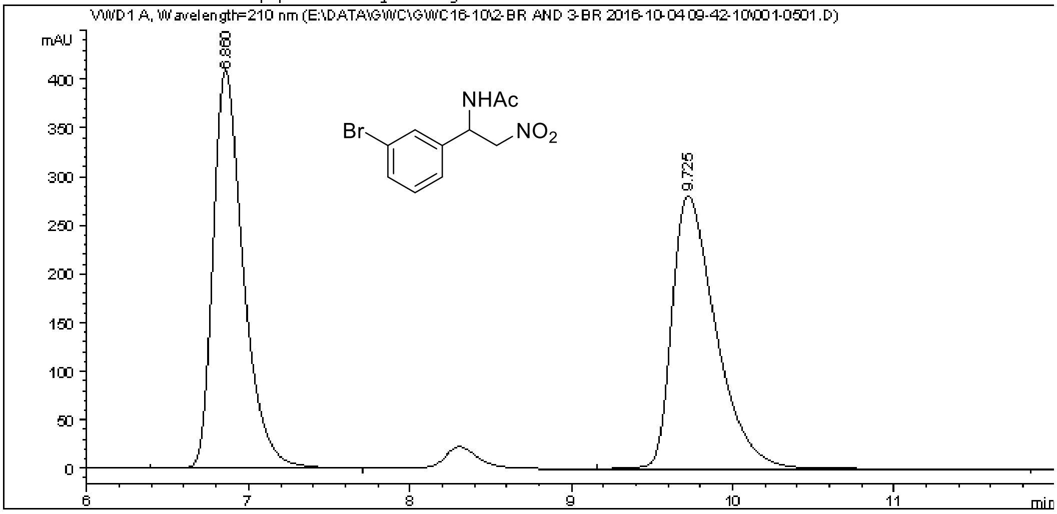


```
    =====================================================================
                             Area Percent Report
    =====================================================================

    Sorted By             :      Signal
    Multiplier            :      1.0000
    Dilution              :      1.0000
    Do not use Multiplier & Dilution Factor with ISTDs


    Signal 1: VWD1 A, Wavelength=210 nm

    Peak RetTime Type  Width     Area      Height     Area
      #   [min]        [min]   [mAU*s]     [mAU]        %
    ----|-------|----|-------|----------|----------|--------|
       1   6.860 BB    0.1975 5363.68115  410.22986  50.1193
       2   9.725 BB    0.2886 5338.14404  281.02087  49.8807

    Totals :                  1.07018e4   691.25073

    =====================================================================
                             *** End of Report ***
```

HPLC-2k-cat

```
Data File E:\DATA\DX\ZXD-32\ZXD-32-34-44-46-AD-AS 2016-09-24 18-30-10\024-1801.D
Sample Name: 3-br-cat

    =====================================================================
    Acq. Operator   : SYSTEM                        Seq. Line :  18
    Acq. Instrument : 1260HPLC-VWD                   Location : Vial 24
    Injection Date  : 9/25/2016 3:04:20 AM                Inj :  1
                                                   Inj Volume : 5.000 µl
    Acq. Method     : E:\DATA\DX\ZXD-32\ZXD-32-34-44-46-AD-AS 2016-09-24 18-30-10\VWD-ADH(1-6)
                      -85-15-210NM-20MIN.M
    Last changed    : 9/24/2016 10:04:57 PM by SYSTEM
    Analysis Method : E:\DATA\DX\ZXD-32\ZXD-32-34-44-46-AD-AS 2016-09-24 18-30-10\VWD-ADH(1-6)
                      -85-15-210NM-20MIN.M (Sequence Method)
    Last changed    : 10/25/2016 9:02:20 PM by SYSTEM
                      (modified after loading)
    Additional Info : Peak(s) manually integrated
```

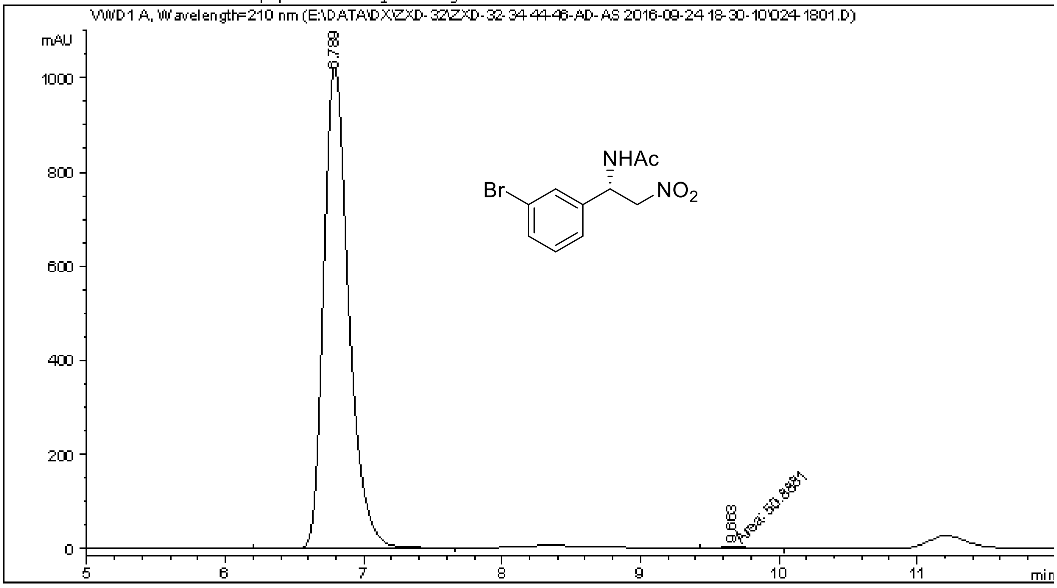


```
    =====================================================================
                             Area Percent Report
    =====================================================================

    Sorted By             :      Signal
    Multiplier            :      1.0000
    Dilution              :      1.0000
    Do not use Multiplier & Dilution Factor with ISTDs


    Signal 1: VWD1 A, Wavelength=210 nm

    Peak RetTime Type  Width     Area      Height     Area
      #   [min]        [min]   [mAU*s]     [mAU]        %
    ----|-------|----|-------|----------|----------|--------|
       1   6.789 BB    0.1915 1.28355e4  1022.09790  99.6051
       2   9.663 MM    0.2817   50.88806    3.01076   0.3949

    Totals :                  1.28863e4  1025.10866


    =====================================================================
                             *** End of Report ***
```

HPLC-2l-rac

Data File E:\DATA\GWC\GWC16-9\SUBSTRATE- SCOPE 2016-09-23 07-58-46\006-1301.D
Sample Name: 2-me-rac

=====================================================================

Acq. Operator : SYSTEM Seq. Line : 13
Acq. Instrument : 1260HPLC-VWD Location : Vial 6
Injection Date : 9/23/2016 12:11:00 PM Inj : 1
Inj Volume : 5.000 µl
Acq. Method : E:\DATA\GWC\GWC16-9\SUBSTRATE- SCOPE 2016-09-23 07-58-46\VWD-ADH(1-6)-85-15-210NM-30MIN.M
Last changed : 9/23/2016 12:20:36 PM by SYSTEM
(modified after loading)
Analysis Method : E:\DATA\GWC\GWC16-9\SUBSTRATE- SCOPE 2016-09-23 07-58-46\VWD-ADH(1-6)-85-15-210NM-30MIN.M (Sequence Method)
Last changed : 10/25/2016 8:13:50 PM by SYSTEM
(modified after loading)
Additional Info : Peak(s) manually integrated

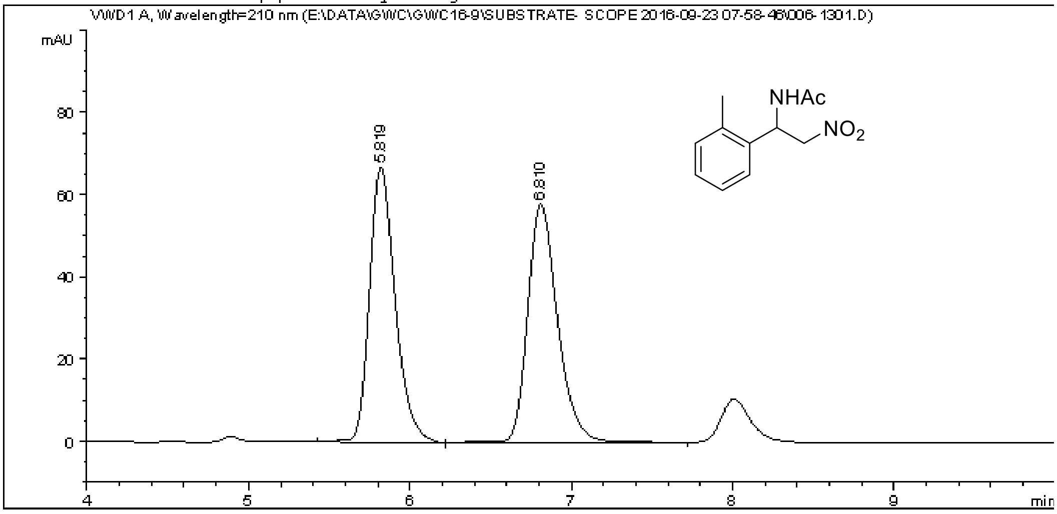


=====================================================================

Area Percent Report

=====================================================================

Sorted By : Signal
Multiplier : 1.0000
Dilution : 1.0000
Do not use Multiplier & Dilution Factor with ISTDs

Signal 1: VWD1 A, Wavelength=210 nm

| Peak # | RetTime [min] | Type | Width [min] | Area [mAU*s] | Height [mAU] | Area % |
|---|---|---|---|---|---|---|
| 1 | 5.819 | BB | 0.1692 | 741.97858 | 67.01857 | 49.7411 |
| 2 | 6.810 | BB | 0.1968 | 749.70227 | 57.99751 | 50.2589 |
| Totals : | | | | 1491.68085 | 125.01608 | |

=====================================================================

*** End of Report ***

HPLC-2l-cat

Data File E:\DATA\GWC\GWC16-9\SUBSTRATE- SCOPE 2016-09-23 07-58-46\015-1601.D
Sample Name: 2-me-cat

=====================================================================

Acq. Operator : SYSTEM Seq. Line : 16
Acq. Instrument : 1260HPLC-VWD Location : Vial 15
Injection Date : 9/23/2016 1:08:19 PM Inj : 1
Inj Volume : 5.000 µl
Acq. Method : E:\DATA\GWC\GWC16-9\SUBSTRATE- SCOPE 2016-09-23 07-58-46\VWD-ADH(1-6)-85-15-210NM-30MIN.M
Last changed : 9/23/2016 1:21:47 PM by SYSTEM (modified after loading)
Analysis Method : E:\DATA\GWC\GWC16-9\SUBSTRATE- SCOPE 2016-09-23 07-58-46\VWD-ADH(1-6)-85-15-210NM-30MIN.M (Sequence Method)
Last changed : 10/25/2016 8:23:45 PM by SYSTEM (modified after loading)
Additional Info : Peak(s) manually integrated

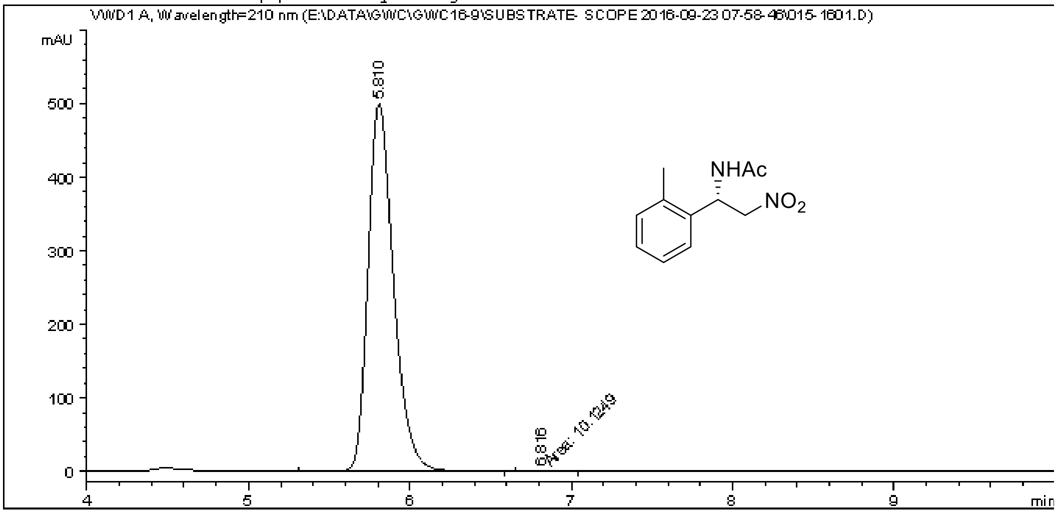


=====================================================================

Area Percent Report

=====================================================================

Sorted By : Signal
Multiplier : 1.0000
Dilution : 1.0000
Do not use Multiplier & Dilution Factor with ISTDs

Signal 1: VWD1 A, Wavelength=210 nm

| Peak # | RetTime [min] | Type | Width [min] | Area [mAU*s] | Height [mAU] | Area % |
|---|---|---|---|---|---|---|
| 1 | 5.810 | BB | 0.1679 | 5471.99609 | 499.22159 | 99.8153 |
| 2 | 6.816 | MM | 0.1899 | 10.12492 | 8.88631e-1 | 0.1847 |

Totals : 5482.12102 500.11022

=====================================================================

*** End of Report ***

HPLC-2m-rac

Data File E:\DATA\GWC\GWC16-10\2-BR AND 3-BR 2016-10-04 09-42-10\002-0701.D
Sample Name: 2-Br-RAC

=====================================================================
Acq. Operator : SYSTEM Seq. Line : 7
Acq. Instrument : 1260HPLC-VWD Location : Vial 2
Injection Date : 10/4/2016 11:22:39 AM Inj : 1
Inj Volume : 5.000 µl
Acq. Method : E:\DATA\GWC\GWC16-10\2-BR AND 3-BR 2016-10-04 09-42-10\VWD-ADH(1-6)-90-10-210NM-40MIN.M
Last changed : 10/4/2016 11:09:48 AM by SYSTEM
Analysis Method : E:\DATA\GWC\GWC16-10\2-BR AND 3-BR 2016-10-04 09-42-10\VWD-ADH(1-6)-90-10-210NM-40MIN.M (Sequence Method)
Last changed : 10/26/2016 10:28:22 AM by SYSTEM
(modified after loading)
Additional Info : Peak(s) manually integrated

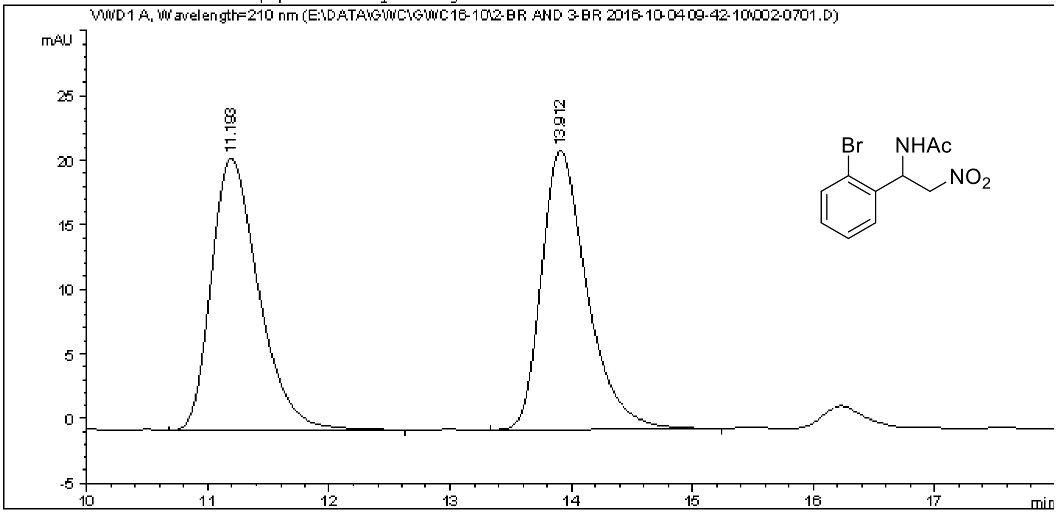


=====================================================================
Area Percent Report
=====================================================================

Sorted By : Signal
Multiplier : 1.0000
Dilution : 1.0000
Do not use Multiplier & Dilution Factor with ISTDs

Signal 1: VWD1 A, Wavelength=210 nm

| Peak # | RetTime [min] | Type | Width [min] | Area [mAU*s] | Height [mAU] | Area % |
|---|---|---|---|---|---|---|
| 1 | 11.193 | BB | 0.4187 | 575.57806 | 21.06749 | 50.3137 |
| 2 | 13.912 | BB | 0.3979 | 568.40088 | 21.60568 | 49.6863 |
| Totals : | | | | 1143.97894 | 42.67317 | |

=====================================================================
*** End of Report ***

HPLC-2m-cat

```
Data File E:\DATA\YHL\YHL-5-204\YHL-5-204-5-6 2016-10-26 13-58-08\006-1701.D
Sample Name: 2

    =====================================================================
    Acq. Operator   : SYSTEM                        Seq. Line :  17
    Acq. Instrument : 1260HPLC-VWD                   Location : Vial 6
    Injection Date  : 10/26/2016 7:41:47 PM               Inj :  1
                                                   Inj Volume : 5.000 µl
    Acq. Method     : E:\DATA\YHL\YHL-5-204\YHL-5-204-5-6 2016-10-26 13-58-08\VWD-ADH(1-6)-90-
                      10-210NM-40MIN.M
    Last changed    : 10/26/2016 2:15:48 PM by SYSTEM
    Analysis Method : E:\DATA\YHL\YHL-5-204\YHL-5-204-5-6 2016-10-26 13-58-08\VWD-ADH(1-6)-90-
                      10-210NM-40MIN.M (Sequence Method)
    Last changed    : 10/27/2016 11:46:18 AM by SYSTEM
                      (modified after loading)
    Additional Info : Peak(s) manually integrated
```

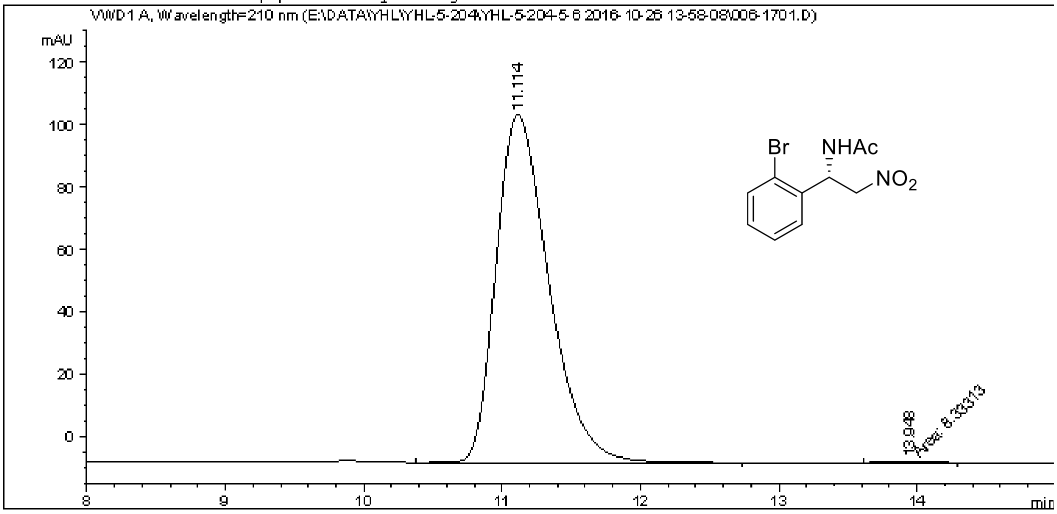


```
    =====================================================================
                             Area Percent Report
    =====================================================================

    Sorted By             :      Signal
    Multiplier            :      1.0000
    Dilution              :      1.0000
    Do not use Multiplier & Dilution Factor with ISTDs


    Signal 1: VWD1 A, Wavelength=210 nm

    Peak RetTime Type  Width     Area      Height     Area
      #   [min]        [min]   [mAU*s]     [mAU]        %
    ----|-------|----|-------|----------|----------|--------|
       1  11.114 BB    0.4091 2962.12622  111.44106  99.7195
       2  13.948 MM    0.3647    8.33313 3.80846e-1  0.2805

    Totals :                  2970.45935  111.82191

    =====================================================================
                             *** End of Report ***
```

HPLC-2n-rac

Data File E:\DATA\GWC\GWC16-9\SUBSTRATE- SCOPE 2016-09-23 07-58-46\005-1201.D
Sample Name: Nai-rac

=====================================================================

Acq. Operator : SYSTEM  Seq. Line : 12
Acq. Instrument : 1260HPLC-VWD  Location : Vial 5
Injection Date : 9/23/2016 11:50:12 AM  Inj : 1
Inj Volume : 2.000 µl
Acq. Method : E:\DATA\GWC\GWC16-9\SUBSTRATE- SCOPE 2016-09-23 07-58-46\VWD-ADH(1-6)-85-15-210NM-10MIN.M
Last changed : 9/23/2016 11:58:04 AM by SYSTEM
(modified after loading)
Analysis Method : E:\DATA\GWC\GWC16-9\SUBSTRATE- SCOPE 2016-09-23 07-58-46\VWD-ADH(1-6)-85-15-210NM-10MIN.M (Sequence Method)
Last changed : 10/25/2016 8:12:23 PM by SYSTEM
(modified after loading)
Additional Info : Peak(s) manually integrated

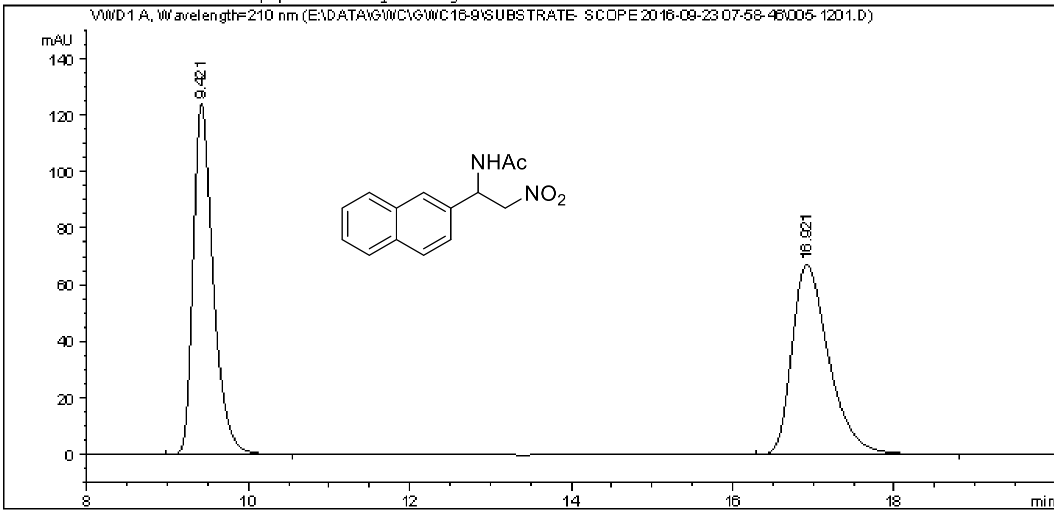


=====================================================================

Area Percent Report

=====================================================================

Sorted By : Signal
Multiplier : 1.0000
Dilution : 1.0000
Do not use Multiplier & Dilution Factor with ISTDs

Signal 1: VWD1 A, Wavelength=210 nm

| Peak # | RetTime [min] | Type | Width [min] | Area [mAU*s] | Height [mAU] | Area % |
|---|---|---|---|---|---|---|
| 1 | 9.421 | BB | 0.2616 | 2143.52905 | 124.06277 | 50.0649 |
| 2 | 16.921 | BB | 0.4825 | 2137.96875 | 67.32954 | 49.9351 |

Totals : 4281.49780 191.39231

=====================================================================

*** End of Report ***

HPLC-2n-cat

```
Data File E:\DATA\GWC\GWC16-9\SUBSTRATE- SCOPE 2016-09-23 07-58-46\026-1801.D
Sample Name: nai-cat

    =====================================================================
    Acq. Operator   : SYSTEM                          Seq. Line :  18
    Acq. Instrument : 1260HPLC-VWD                     Location : Vial 26
    Injection Date  : 9/23/2016 1:43:50 PM                  Inj :   1
                                                    Inj Volume : 5.000 µl
    Acq. Method     : E:\DATA\GWC\GWC16-9\SUBSTRATE- SCOPE 2016-09-23 07-58-46\VWD-ADH(1-6)-85
                      -15-210NM-30MIN.M
    Last changed    : 9/23/2016 1:23:17 PM by SYSTEM
    Analysis Method : E:\DATA\GWC\GWC16-9\SUBSTRATE- SCOPE 2016-09-23 07-58-46\VWD-ADH(1-6)-85
                      -15-210NM-30MIN.M (Sequence Method)
    Last changed    : 10/25/2016 8:26:28 PM by SYSTEM
                      (modified after loading)
    Additional Info : Peak(s) manually integrated
```

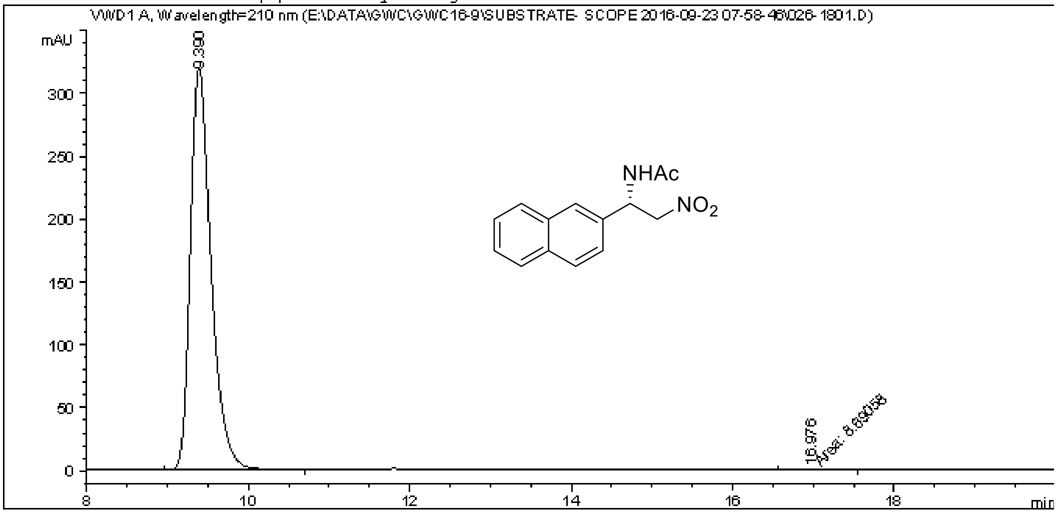


```
    =====================================================================
                         Area Percent Report
    =====================================================================

    Sorted By             :      Signal
    Multiplier            :      1.0000
    Dilution              :      1.0000
    Do not use Multiplier & Dilution Factor with ISTDs


    Signal 1: VWD1 A, Wavelength=210 nm
```

| Peak # | RetTime [min] | Type | Width [min] | Area [mAU*s] | Height [mAU] | Area % |
|---|---|---|---|---|---|---|
| 1 | 9.390 | BB | 0.2708 | 5709.43457 | 320.51248 | 99.8480 |
| 2 | 16.976 | MM | 0.4998 | 8.69058 | 2.89791e-1 | 0.1520 |

| Totals : | | | | 5718.12515 | 320.80227 | |
|---|---|---|---|---|---|---|

```
    =====================================================================
                         *** End of Report ***
```

HPLC-2o-rac

```
Data File E:\DATA\WWL\WWL-3-DKR-DIRATIVE\WWL-3-DKR-DIRATIVE 2016-11-03 12-12-24\042-0601.D
Sample Name: rac

    =====================================================================
    Acq. Operator   : SYSTEM                          Seq. Line :   6
    Acq. Instrument : 1260HPLC-VWD                     Location : Vial 42
    Injection Date  : 11/3/2016 2:02:08 PM                  Inj :   1
                                                    Inj Volume : 5.000 µl
    Acq. Method     : E:\DATA\WWL\WWL-3-DKR-DIRATIVE\WWL-3-DKR-DIRATIVE 2016-11-03 12-12-24
                      \VWD-ADH(1-6)-90-10-210NM-20MIN.M
    Last changed    : 11/3/2016 12:14:22 PM by SYSTEM
    Analysis Method : E:\DATA\WWL\WWL-3-DKR-DIRATIVE\WWL-3-DKR-DIRATIVE 2016-11-03 12-12-24
                      \VWD-ADH(1-6)-90-10-210NM-20MIN.M (Sequence Method)
    Last changed    : 11/3/2016 2:36:58 PM by SYSTEM
                      (modified after loading)
    Additional Info : Peak(s) manually integrated
```

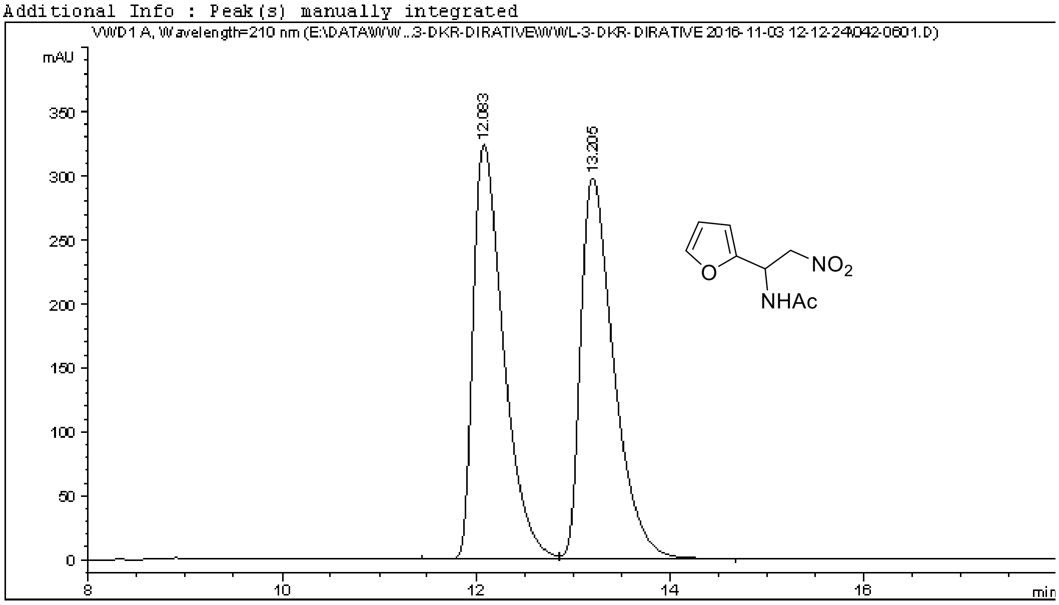


```
    =====================================================================
                             Area Percent Report
    =====================================================================

    Sorted By             :      Signal
    Multiplier            :      1.0000
    Dilution              :      1.0000
    Do not use Multiplier & Dilution Factor with ISTDs


    Signal 1: VWD1 A, Wavelength=210 nm

    Peak RetTime Type  Width     Area      Height     Area
      #   [min]        [min]   [mAU*s]     [mAU]        %
    ----|-------|----|-------|----------|----------|--------|
       1  12.083 BV    0.3320 7048.88770  323.80307  49.8933
       2  13.205 VB    0.3614 7079.03027  297.73203  50.1067

    Totals :                  1.41279e4   621.53510


    =====================================================================
                             *** End of Report ***

1260HPLC-VWD 11/3/2016 2:37:08 PM SYSTEM                                  Page 1 of 1
```

HPLC-2o-cat

```
Data File E:\DATA\WWL\WWL-3-DKR-DIRATIVE\WWL-3-DKR-DIRATIVE 2016-11-03 12-12-24\041-0701.D
Sample Name: cat

    =================================================================
    Acq. Operator   : SYSTEM                        Seq. Line :   7
    Acq. Instrument : 1260HPLC-VWD                   Location : Vial 41
    Injection Date  : 11/3/2016 2:22:54 PM                Inj :   1
                                                   Inj Volume : 5.000 µl
    Acq. Method     : E:\DATA\WWL\WWL-3-DKR-DIRATIVE\WWL-3-DKR-DIRATIVE 2016-11-03 12-12-24
                      \VWD-ADH(1-6)-90-10-210NM-20MIN.M
    Last changed    : 11/3/2016 2:39:26 PM by SYSTEM
                      (modified after loading)
    Analysis Method : E:\DATA\WWL\WWL-3-DKR-DIRATIVE\WWL-3-DKR-DIRATIVE 2016-11-03 12-12-24
                      \VWD-ADH(1-6)-90-10-210NM-20MIN.M (Sequence Method)
    Last changed    : 11/3/2016 2:43:02 PM by SYSTEM
                      (modified after loading)
    Additional Info : Peak(s) manually integrated
```

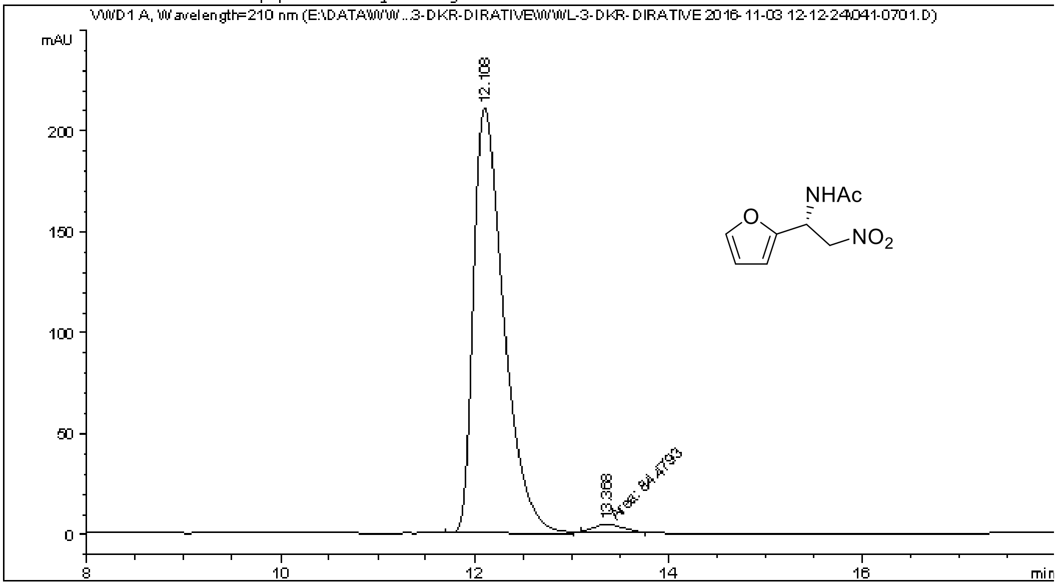


```
    =================================================================
                         Area Percent Report
    =================================================================

    Sorted By             :      Signal
    Multiplier            :      1.0000
    Dilution              :      1.0000
    Do not use Multiplier & Dilution Factor with ISTDs


    Signal 1: VWD1 A, Wavelength=210 nm

    Peak RetTime Type  Width     Area      Height     Area
      #   [min]        [min]   [mAU*s]     [mAU]        %
    ----|-------|----|-------|----------|----------|--------|
       1  12.108 BV    0.3320 4585.45508  210.59842  98.1910
       2  13.368 MM    0.3501   84.47933    4.02145   1.8090

    Totals :                  4669.93441  214.61987

    =================================================================
```

HPLC-2p-rac

Data File E:\DATA\GWC\GWC16-10\ZHI-OTHER 2016-10-19 08-01-51\013-0201.D
Sample Name: zhi-rac

```
=====================================================================
Acq. Operator   : SYSTEM                        Seq. Line :   2
Acq. Instrument : 1260HPLC-VWD                   Location : Vial 13
Injection Date  : 10/19/2016 8:13:24 AM               Inj :   1
                                               Inj Volume : 5.000 µl
Acq. Method     : E:\DATA\GWC\GWC16-10\ZHI-OTHER 2016-10-19 08-01-51\VWD-ADH(1-6)-97-3-0.9
                  -210NM-40MIN.M
Last changed    : 10/19/2016 8:43:09 AM by SYSTEM
                  (modified after loading)
Analysis Method : E:\DATA\GWC\GWC16-10\ZHI-OTHER 2016-10-19 08-01-51\VWD-ADH(1-6)-97-3-0.9
                  -210NM-40MIN.M (Sequence Method)
Last changed    : 10/25/2016 10:51:16 AM by SYSTEM
                  (modified after loading)
Additional Info : Peak(s) manually integrated
```

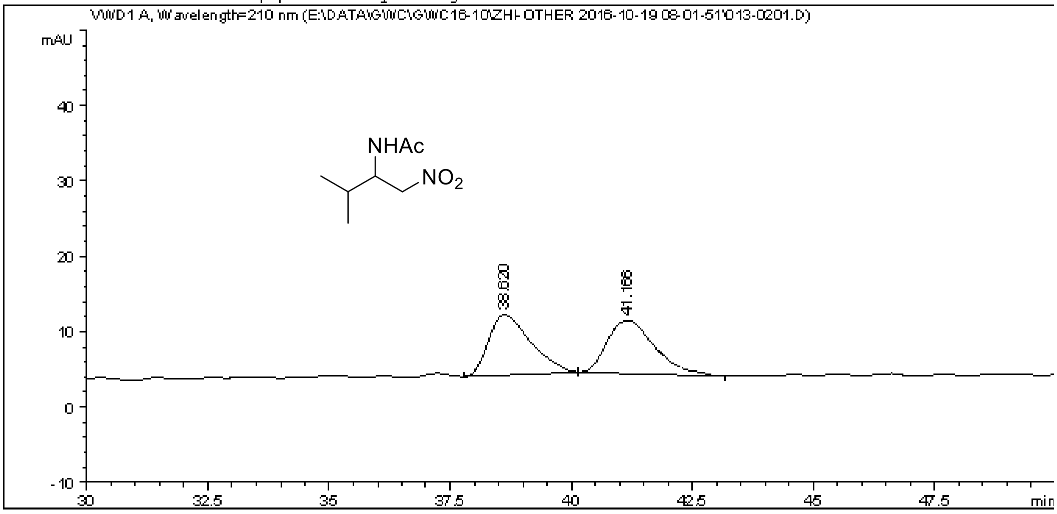


```
=====================================================================
                         Area Percent Report
=====================================================================

Sorted By             :      Signal
Multiplier            :      1.0000
Dilution              :      1.0000
Do not use Multiplier & Dilution Factor with ISTDs


Signal 1: VWD1 A, Wavelength=210 nm

Peak RetTime Type  Width     Area      Height     Area
  #   [min]        [min]   [mAU*s]     [mAU]       %
----|-------|----|-------|----------|----------|--------|
   1  38.620 BB    0.8918  481.31561    7.97812  50.0437
   2  41.166 BB    0.9897  480.47427    7.03635  49.9563

Totals :                   961.78989   15.01447


=====================================================================
                          *** End of Report ***
```

HPLC-2p-cat

Data File E:\DATA\GWC\GWC16-10\ZHI-OTHER 2016-10-19 08-01-51\014-0301.D
Sample Name: ZHI-CAT

=====================================================================

Acq. Operator : SYSTEM Seq. Line : 3
Acq. Instrument : 1260HPLC-VWD Location : Vial 14
Injection Date : 10/19/2016 9:04:09 AM Inj : 1
Inj Volume : 5.000 µl
Acq. Method : E:\DATA\GWC\GWC16-10\ZHI-OTHER 2016-10-19 08-01-51\VWD-ADH(1-6)-97-3-0.9-210NM-40MIN.M
Last changed : 10/19/2016 8:43:09 AM by SYSTEM
Analysis Method : E:\DATA\GWC\GWC16-10\ZHI-OTHER 2016-10-19 08-01-51\VWD-ADH(1-6)-97-3-0.9-210NM-40MIN.M (Sequence Method)
Last changed : 10/25/2016 7:57:42 PM by SYSTEM
(modified after loading)
Additional Info : Peak(s) manually integrated

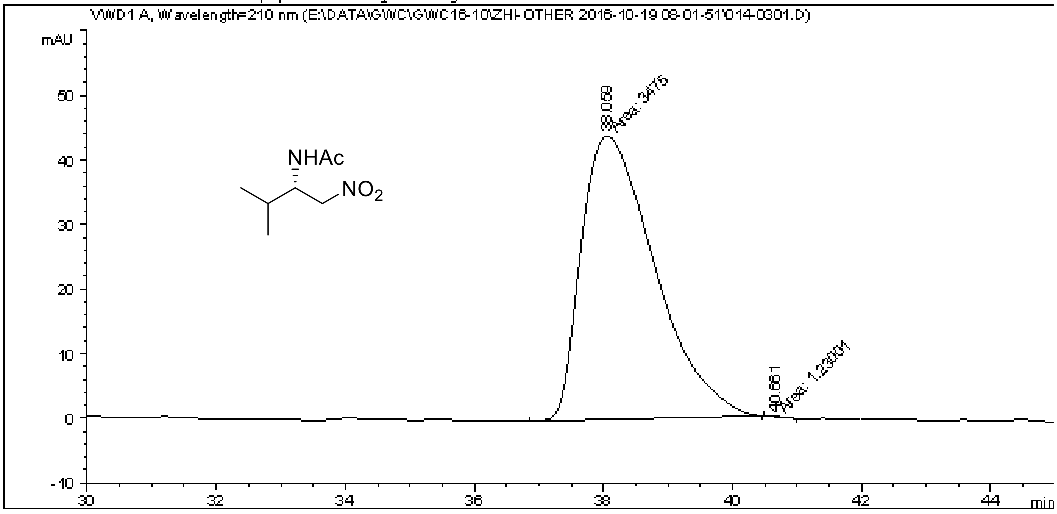


=====================================================================

Area Percent Report

=====================================================================

Sorted By : Signal
Multiplier : 1.0000
Dilution : 1.0000
Do not use Multiplier & Dilution Factor with ISTDs

Signal 1: VWD1 A, Wavelength=210 nm

| Peak # | RetTime [min] | Type | Width [min] | Area [mAU*s] | Height [mAU] | Area % |
|---|---|---|---|---|---|---|
| 1 | 38.059 | MM | 1.3206 | 3474.99585 | 43.85545 | 99.9646 |
| 2 | 40.661 | MM | 0.2434 | 1.23001 | 8.42135e-2 | 0.0354 |

Totals : 3476.22586 43.93966

=====================================================================

*** End of Report ***

HPLC-2q-rac

Data File E:\DATA\GWC\GWC16-9\SUBSTRATE- SCOPE 2016-09-23 07-58-46\013-0701.D
Sample Name: six-huan-rac

=====================================================================

Acq. Operator : SYSTEM  Seq. Line : 7
Acq. Instrument : 1260HPLC-VWD  Location : Vial 13
Injection Date : 9/23/2016 10:14:07 AM  Inj : 1
Inj Volume : 5.000 µl
Acq. Method : E:\DATA\GWC\GWC16-9\SUBSTRATE- SCOPE 2016-09-23 07-58-46\VWD-ADH(1-6)-90-10-210NM-40MIN.M
Last changed : 9/23/2016 9:47:19 AM by SYSTEM
Analysis Method : E:\DATA\GWC\GWC16-9\SUBSTRATE- SCOPE 2016-09-23 07-58-46\VWD-ADH(1-6)-90-10-210NM-40MIN.M (Sequence Method)
Last changed : 10/25/2016 8:07:41 PM by SYSTEM
(modified after loading)
Additional Info : Peak(s) manually integrated

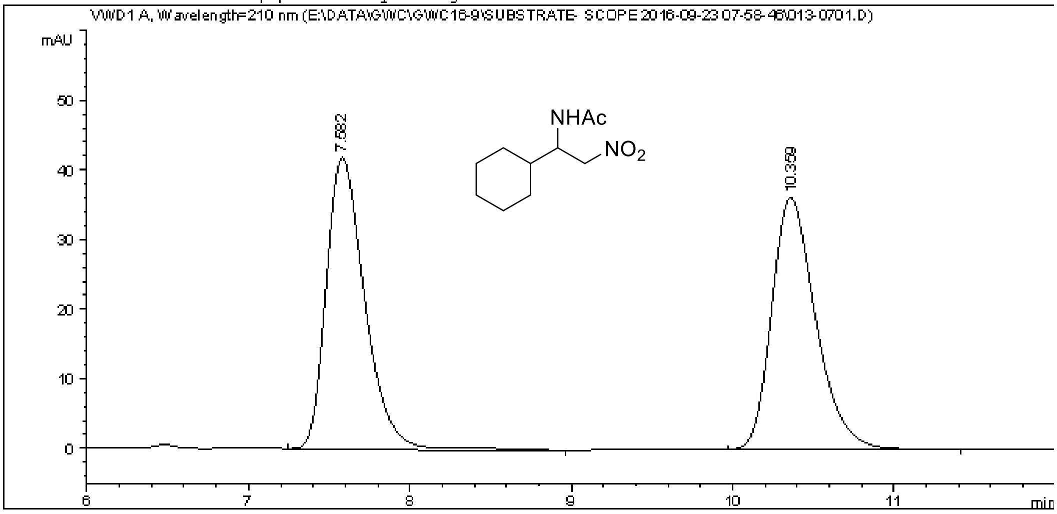


=====================================================================

Area Percent Report

=====================================================================

Sorted By : Signal
Multiplier : 1.0000
Dilution : 1.0000
Do not use Multiplier & Dilution Factor with ISTDs

Signal 1: VWD1 A, Wavelength=210 nm

| Peak # | RetTime [min] | Type | Width [min] | Area [mAU*s] | Height [mAU] | Area % |
|---|---|---|---|---|---|---|
| 1 | 7.582 | BB | 0.2558 | 704.48138 | 41.96710 | 50.7415 |
| 2 | 10.359 | BB | 0.2910 | 683.89142 | 36.11015 | 49.2585 |

Totals : 1388.37280 78.07725

=====================================================================

*** End of Report ***

HPLC-2q-cat

Data File E:\DATA\GWC\GWC16-9\SUBSTRATE- SCOPE 2016-09-23 07-58-46\025-2301.D
Sample Name: sixhuan-cat

=====================================================================

Acq. Operator : SYSTEM　　Seq. Line : 23
Acq. Instrument : 1260HPLC-VWD　　Location : Vial 25
Injection Date : 9/23/2016 3:43:20 PM　　Inj : 1
Inj Volume : 5.000 µl
Acq. Method : E:\DATA\GWC\GWC16-9\SUBSTRATE- SCOPE 2016-09-23 07-58-46\VWD-ADH(1-6)-90-10-210NM-40MIN.M
Last changed : 9/23/2016 3:45:05 PM by SYSTEM (modified after loading)
Analysis Method : E:\DATA\GWC\GWC16-9\SUBSTRATE- SCOPE 2016-09-23 07-58-46\VWD-ADH(1-6)-90-10-210NM-40MIN.M (Sequence Method)
Last changed : 10/25/2016 8:36:03 PM by SYSTEM (modified after loading)
Additional Info : Peak(s) manually integrated

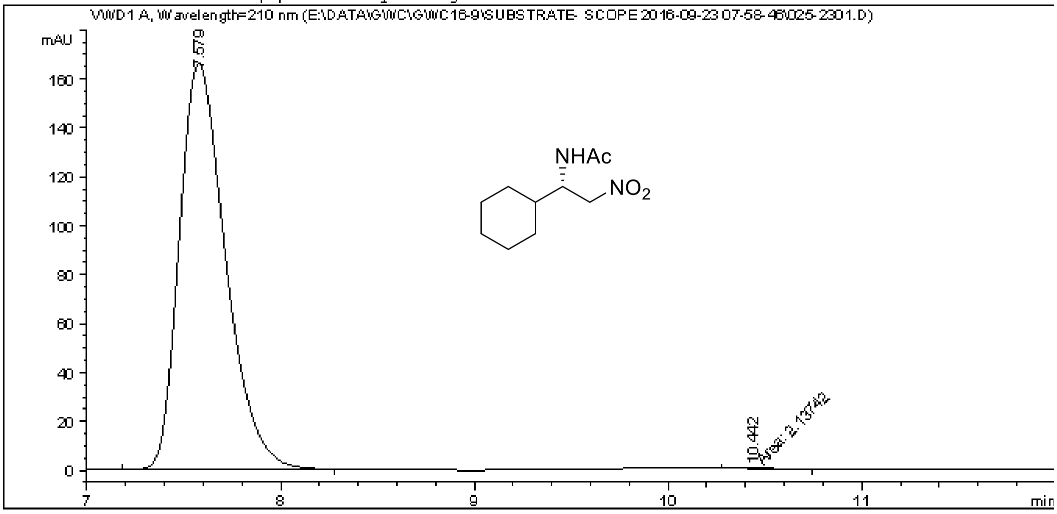


=====================================================================

Area Percent Report

=====================================================================

Sorted By : Signal
Multiplier : 1.0000
Dilution : 1.0000
Do not use Multiplier & Dilution Factor with ISTDs

Signal 1: VWD1 A, Wavelength=210 nm

| Peak # | RetTime [min] | Type | Width [min] | Area [mAU*s] | Height [mAU] | Area % |
|---|---|---|---|---|---|---|
| 1 | 7.579 | BB | 0.2563 | 2750.34399 | 165.97928 | 99.9223 |
| 2 | 10.442 | MM | 0.2352 | 2.13742 | 1.51444e-1 | 0.0777 |

Totals : 2752.48141 166.13072

=====================================================================

*** End of Report ***
